# Shotgun Metagenome Analysis of Two *Schizaphis graminum* Biotypes over Time With and Without Carried Cereal Yellow Dwarf Virus

**DOI:** 10.3390/insects16060554

**Published:** 2025-05-23

**Authors:** Yan M. Crane, Charles F. Crane, Subhashree Subramanyam, Brandon J. Schemerhorn

**Affiliations:** 1 Crop Production and Pest Control Research Unit, United States Department of Agriculture-Agricultural Research Service (USDA-ARS), West Lafayette, IN 47907, USA; yan.crane@usda.gov (Y.M.C.); ccrane@purdue.edu (C.F.C.); subhashree.subramanyam@usda.gov (S.S.); 2 Department of Entomology, Purdue University, West Lafayette, IN 47907, USA; 3 Department of Botany and Plant Pathology, Purdue University, West Lafayette, IN 47907, USA

**Keywords:** *Schizaphis graminum*, RNA-seq, microbiome, CYDV-RPV, gut microflora

## Abstract

The greenbug aphid (*Schizaphis graminum* (Rondani)) is a major pest of wheat and an important vector of wheat viruses. An RNA-seq study was conducted to investigate the microbial effects of two greenbug genotypes, the presence or absence of cereal yellow dwarf virus, and the condition of the wheat host over a 20-day time course of unrestricted greenbug feeding. Messenger RNA reads were mapped to ca. 47,000 bacterial, 1218 archaeal, 14,165 viral, 571 fungal, and 94 protozoan reference or representative genomes, plus greenbug itself and its wheat host. Taxon counts were analyzed with QIIME2 and DESeq2. Distinct early (days 1 through 10) and late (days 15 and 20) communities differed in the abundance of typical enteric genera (*Shigella*, *Escherichia*, *Citrobacter*), which declined in the late community, while the ratio of microbial to greenbug read counts declined 50% and diversity measures increased. The nearly universal aphid endosymbiont, *Buchnera aphidicola*, accounted for less than 25% of the read counts in both communities. There were 302 differentially expressed (populated) genera with respect to early and late dates, while 25 genera differed between the greenbug genotypes and nine differed between carrier and virus-free greenbugs. The late community was likely responding to starvation as the wheat host succumbed to aphid feeding. Our results add to basic knowledge about aphid microbiomes and offer an attractive alternative method to assess insect microbiomes.

## Simple Summary

The greenbug aphid is one of the leading pests of wheat and sorghum in the United States. Messenger RNA was used to inventory the microorganisms in two forms of greenbugs, each with and without a wheat virus, over 20 days. Many of the most common bacteria were similar to those in human feces, while an important aphid-specific bacterium was not as dominant as usually seen in aphids. The overall microbial population declined by 50% from day 5 to day 20, led by a decline in the typical fecal organisms. The greenbug genotype and wheat virus affected the microorganisms less than the collection date. This study adds to basic knowledge about microbes in aphids and the methods used to census these microbes.

## 1. Introduction

Phytophagous insects cohabit with a multitude of microorganisms (the microbiome) that potentially influence their nutrition [1], longevity, reproduction [2], behavior [3], and gene expression [4,5]. The insect can interact with its microbiome in various ways, including, among others, diet, immune and stress responses [6,7,8,9], specific signaling pathways [4], and anatomical adaptations [8], which in turn depend upon insect genetics. The insect also interacts with its host plant individually and as a population. Feeding withdraws nutrients from the plant, disrupts plant tissues, opens the way for plant pathogens to invade [10], and in some cases introduces pathogens [11,12,13], toxins [14], or effectors of hypersensitive response into the plant [15]. Thus, the quality and quantity of the insects’ diet can respond to elapsed time by way of increased insect population density, sustained withdrawal of nutrients, development of symptomatic disease, and ultimately death of the host plant.

Because of their small body size, RNA-seq experiments in insects often sample whole individuals or groups of individuals. In this case, the transcriptome includes transcripts from the microbiome and potentially the immediate environment. With deep sequencing of the target transcriptome, there are enough microbial reads to survey the composition and community structure of the microbiome. Since these transcripts come from throughout the genome, the Silva and UNITE databases of hypervariable rRNA regions are useless for assigning them to organisms, and new methods to count transcripts in whole genomes must be developed. Our previous RNA-seq study of gene expression in greenbug aphids (*Schizaphis graminum* (Rondani)) [16] generated 110 million microbial reads over a time course that included the decline of the host plant under feeding pressure. Here we describe how we counted these reads to whole genomes and how the microbiome responded to greenbug biotype and carried cereal yellow dwarf virus over the time course. We compare the resulting taxonomic abundances to the results of 16S rDNA amplicon sequencing in related aphid species. This appears to be the first report of a microbiome from the greenbug itself.

Greenbug remains a major pest of small grains, especially wheat and sorghum [17], although it is also a generalist pest of grasses [17]. Annual economic loss to greenbug probably exceeded USD 250,000,000 in 1999 [18]. Greenbug outbreaks have decreased since 2000 (https://entomology.k-state.edu/extension/crop-protection/sorghum/greenbug.html, accessed on 7 May 2025), and current estimates of yield loss specific to greenbug are unavailable. Greenbug saliva has been considered toxic to the plant [19], and greenbug is an important vector of yellow-dwarf viruses [20] that cause disease in grasses. The main vectored viruses are barley yellow dwarf virus (BYDV-PAV) and cereal yellow dwarf virus (CYDV-RPV and CYDV-RPS), which collectively were the eighth leading cause of wheat yield reduction in 29 US states and two Canadian provinces in 2024 (https://cropprotectionnetwork.org/publications/wheat-disease-loss-estimates-from-the-united-states-and-ontario-canada-2024, accessed on 7 May 2025), causing about 10% of the loss produced by the leading disease, stripe rust. Historically, these viruses have been considered as closely related luteoviruses, but as more fully sequenced genomes have become available, their perceived relationship has become more tenuous [21]. Currently, they are classified in separate genera (*Polerovirus* and *Luteovirus*) with different arrangements of open reading frames. CYDV is more closely related to other poleroviruses that do not infect grasses [22]. For example, CYDV strains formed a sister clade to potato leaf roll virus with 97% bootstrap support in a maximum likelihood tree of *Polerovirus* based on the translated amino acid sequence of open reading frame two [22].

Amplicon-based studies have dealt with microbiomes in related aphid species, but apparently not *S. graminum* itself; thus, there is a gap in basic knowledge to fill about this important wheat and sorghum pest. Related genera include *Rhopalosiphum*, *Aphis*, *Protaphis*, and more distantly *Acyrtosiphon*, *Myzus*, *Sitobion*, and *Diuraphis* [23]. *Buchnera aphidicola*, the obligate intracellular endosymbiont of almost all aphids, comprised 84.4% of total reads in *S. avenae* [24], at least 98.5% of total reads in six samples of *R. padi* [24], 100% of total reads in *D. noxia* [25], 53% to 94% in five samples of *A*. *gossypii* [26], 86.8% and 99.9% in two color morphs of *A*. *gossypii* [27], 70% to 98% in six collections of *A*. *gossypii* [28], and 95% to 98% in *M*. *persicae* [28]. The percentage of *Buchnera* among 16S amplicons seems not to have been recently reported in *Acyrthosiphon*, but the titer of secondary endosymbiont *Hamiltonella defensa* declines reversibly at low temperature [29]. *Buchnera* was about 100 times as abundant as *Arsenophonus* or *Wolbachia* in *A*. *glycines* as measured by quantitative PCR [30]. Fakhour et al. [25] also reported the secondary symbionts *Regiella insecticola* and *Hamiltonella defensa* in 77.8% of collections of *S. avenae* and free-living genera *Erwinia*, *Pantoea*, *Pseudomonas*, *Acinetobacter*, and *Staphylococcus* in 28.3% of collections of *R*. *padi*. Ma et al. [26] also found *Serratia* to account for 24% of reads from one sample of *A*. *gossypii*. Gallo-Franco et al. [28] found 17.5% of the reads of one collection of *A*. *gossypii* to come from *Pseudomonas*. In contrast to the preponderance of *Buchnera* in the preceding examples, He et al. [31] did not exclude rare taxa and found 1607 genera of bacteria and 37 archaeal taxa in *M. persicae* fed Chinese cabbage, eggplant, pepper, or tobacco, with the greatest diversity of genera on pepper. *Buchnera* comprised only 12.5% to 25.5% of the reads from the solanaceous-fed aphids and was outnumbered by *Pseudomonas* (62% and 69%) with feeding on eggplant or tobacco, and this excess of *Pseudomonas* was upheld with qPCR with genus-specific primers.

During data analysis, it was found that 20 of the 95 samples in our RNA-seq study also contained Rhopalosiphum padi virus (RhPV, not to be confused with CYDV-RPV). RhPV is pathogenic to aphids and reduces their lifespan and fecundity [32]. RhPV can be transmitted to and from host plants and is mobile in host plants [32] without causing visible plant disease. Thus, there was also an opportunity to investigate if RhPV independently affected the greenbug microbiome.

This study was designed to test the effects, if any, of greenbug genotype, CYDV carrier status, time, and incidentally RhPV, on the identity, relative abundance, and diversity metrics of microorganisms in or on the greenbugs. By following the greenbug population through the growth and decline of its host wheat plants, we explored the response of the microbiome to conditions not usually considered in microbiome studies. It also provides an indirect opportunity to compare shotgun with amplicon-based methods to analyze microbiomes, given that amplicon-based analyses have been performed in related aphid species.

## 2. Materials and Methods

### 2.1. Experimental Execution

**Figure 1:**
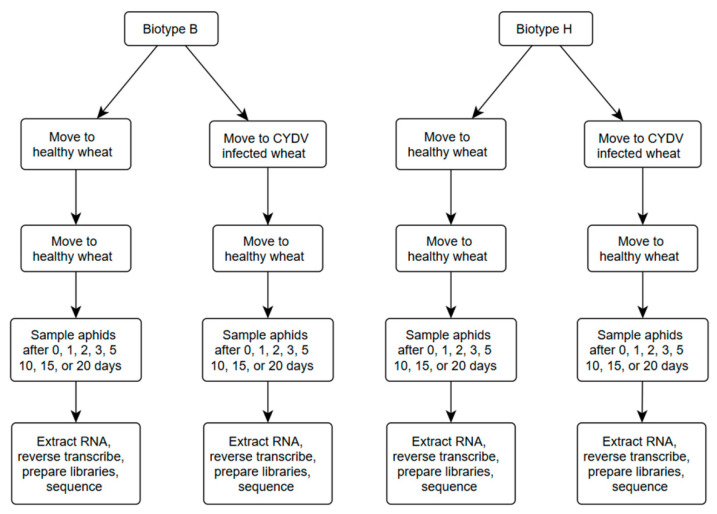
Experimental layout. Two greenbug biotypes, each with and without acquired CYDV, were sampled over eight timepoints from infestation until 20 days later.

Reads were reanalyzed from a previous RNA-seq study of greenbug gene expression in relation to biotype, harbored CYDV-RPV, and time post-infestation [16]. The experimental layout, which is diagrammed in Figure 1, was the same as in Crane et al. [16]. The populations of biotypes B and H descended parthenogenetically from a single individual of each biotype and therefore were genetically uniform. Half of the aphids of each biotype were allowed to acquire CYDV-RPV from infected wheat cv. “Newton” plants, while the other half remained CYDV-RPV-free. A sequence specific to CYDV-RPV was amplifiable only from infected wheat, whereas nine other wheat viruses were not detected [16]. Then the aphids were moved to separate sets of CYDV-RPV-free wheat cv. “Newton” plants that were placed in separate growth chambers, and their population was allowed to increase over 20 days with sampling at days 0, 1, 2, 3, 5, 10, 15, and 20. Three groups of aphids were sampled from separate wheat plants (biological replicates) per combination of biotype, viral carrier status, and timepoint. Each individual wheat plant was sampled only once. Over this time course, the wheat plants with viruliferous aphids developed yellow-dwarf symptoms, indicating successful infection. The CYDV-RPV-specific sequence could be amplified only from the viruliferous aphids [16]. Aphids were flash-frozen by dropping an infested leaf segment into liquid nitrogen, then collecting and counting the aphids. RNA was extracted from each group of aphids with the Qiagen RNeasy Mini kit (Germantown, MD, USA) and stored at −80 °C. Library preparation and Illumina sequencing were performed by Novogene USA (Sacramento, CA, USA). Sequence was obtained for 95 of 96 samples; the failed sample was one replicate of biotype B without CYDV at day 15. The resulting reads are available at the NCBI sequence read archive under project PRJNA981508.

### 2.2. Bioinformatic Processing

**Figure 2:**
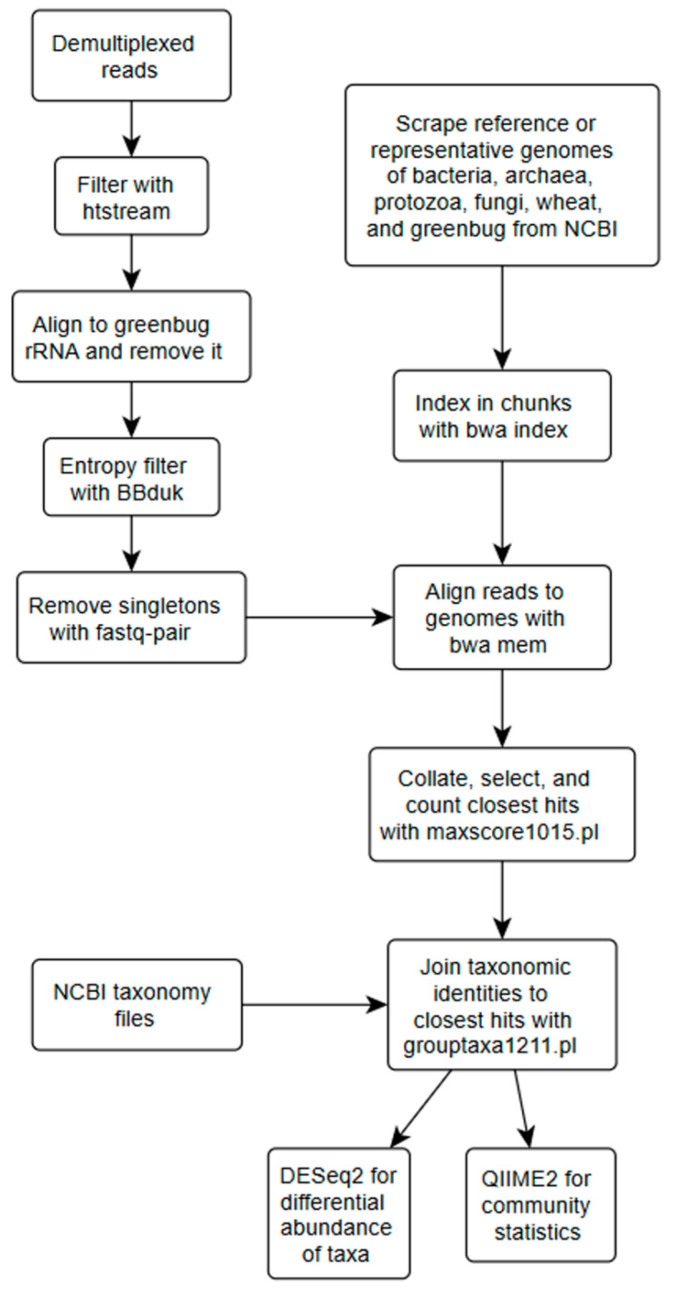
Flowchart of the informatic processing after demultiplexing and initial quality control of reads.

As part of the previous study of greenbug gene expression [16], demultiplexed reads from Novogene USA (Sacramento, CA, USA) were processed, as depicted in Figure 2. Read trimming, quality filtering, and removal of greenbug rRNA, were performed with HTStream ([33]; https://github.com/s4hts/HTStream, accessed on 7 May 2025; Bioinformatics Core, University of California, Davis, CA, USA) without the “superdeduper” step, which otherwise would have removed duplicate reads. This was justified because the library preparation did not involve PCR amplification, so duplicate reads were not expected. HTStream used NCBI Genbank aphid accessions AH003128.2, AB369153.1, AB369137.1, and S50426.1 as rRNA reference sequences. Reads with entropy below 0.7 were removed with BBduk ([34]; https://sourceforge.net/projects/bbmap/, accessed on 7 May 2025; U.S. Department of Energy Joint Genome Institute, Walnut Creek, CA, USA); such reads often included long microsatellites, polyA tails, or long tracts of single nucleotides indicative of low-quality sequencing. Empty or singleton reads left from previous processing were removed with fastq-pair [35]. From here onward, the analysis followed the general strategy of RNAseq, except that whole genomes took the place of gene sequences and taxa took the place of gene names. Reference or representative genomes of ca. 47,000 bacterial, 1218 archaeal, 14,165 viral, 571 fungal, and 94 protozoan taxa, plus greenbug (GCA_020882235.1) and wheat cv. “Chinese Spring” (GCF_018294505.1) were downloaded from NCBI Genomes, split into 63 subsets, and indexed with bwa index [36]. The slowness of bwa indexing even in a high-performing cluster environment necessitated this much splitting of the genomic database. Each read was mapped to each of 63 database chunks with bwa mem [36]. A perl script, maxscore1015.pl (https://zenodo.org/records/13750628; accessed on 11 September 2024), was written to identify the highest scoring hit for each read among the .sam outputs of bwa mem, and the read was credited to that genome. Another perl script, grouptaxa1211.pl (https://zenodo.org/records/13750628; accessed on 11 September 2024), was written to join an NCBI taxonomic identifier to each accession and group accession counts by taxon. Read counts that mapped to wheat and greenbug were ignored. Microbial read counts were structured exactly like gene read counts, and thus DESeq2 [37] was used to detect differential “expression” (population if all taxa were equally transcribed). Read counts at each taxonomic level from genus to phylum were also transformed to biom format by biom convert [38] and imported into a QIIME2 feature table for display and statistical analysis with functions “diversity core-metrics”, “diversity beta-group-significance” [39], “diversity adonis” [40], and “taxa barplot”.

## 3. Results

### 3.1. Overview

**Figure 3:**
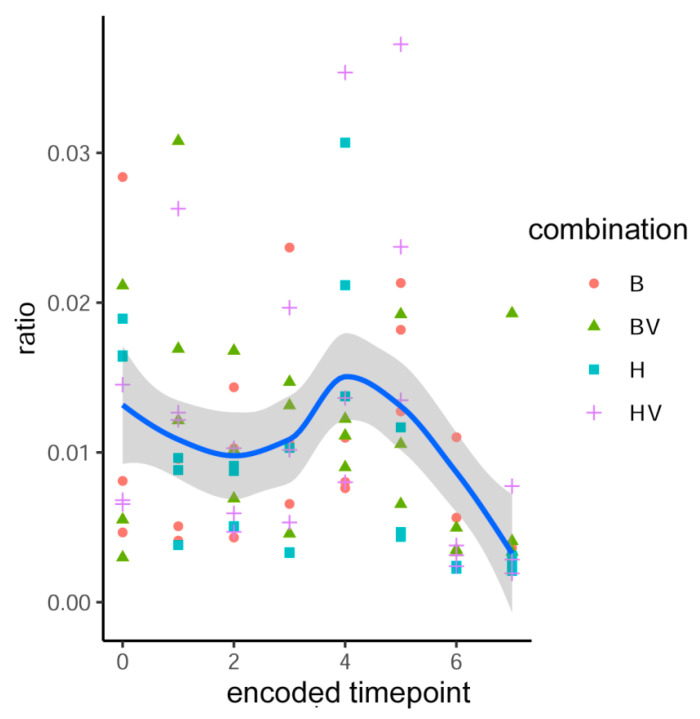
Relationship of total-microbe:greenbug counts ratio to time. Timepoints are encoded as 0, start; 1, one day post infestation; 2, 2 days; 3, 3 days; 4, 5 days; 5, 10 days; 6, 15 days; 7, 20 days post infestation. Combinations of biotype and CYDV-carrier status are encoded as B and BV for biotype B without and with carried CYDV, and H and HV for biotype H without and with carried CYDV. Total microbial counts declined markedly on days 15 and 20, primarily from loss of *Shigella* and *Escherichia* counts. A fitted Loess curve appears in blue.

**Figure 4:**
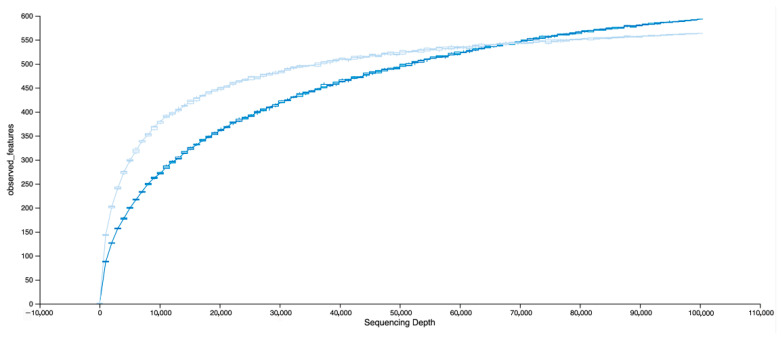
Rarefaction boxplot of counts of genera versus subsample size for 89 samples with at least 100,000 microbial reads. The darker curve is for all samples taken from days 0 through 10. The lighter curve is for days 15 and 20.

**Figure 5:**
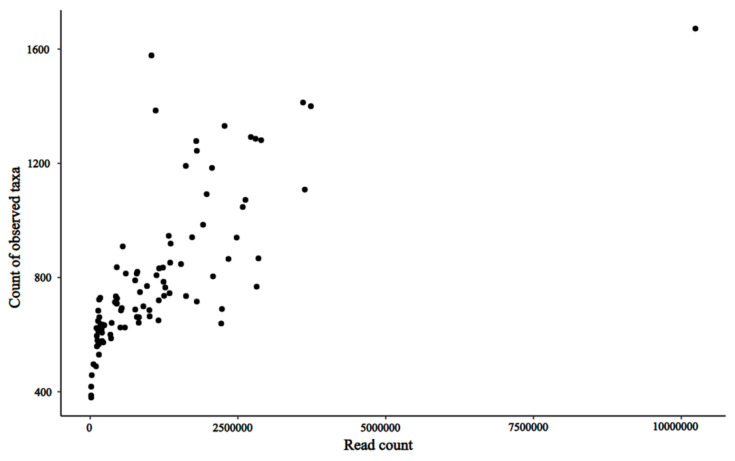
Counts of detected genera versus total read counts per sample.

Over the entire study, 118,059,680 reads (1.233% of total) mapped to wheat, 9,350,093,175 (97.619%) to greenbug, and 110,008,016 (1.149%) to microbes. There were 16,474 to 10,239,120 microbial reads per sample. The ratio of total microbial counts to total greenbug counts peaked at day 5 and declined 50% by day 20 (Figure 3, where the consecutive timepoints are numbered 0 through 7). The microbial reads matched 3833 genera, 836 families, 367 orders, 153 classes, and 71 phyla. Bacteria accounted for 46 of the 50 most abundant genera. While the 30 most abundant genera accounted for 70 to 90% of the reads among samples, the counts indicated a diverse microbiome with representatives from all five kingdoms searched (bacteria, archaea, viruses, fungi, and protozoa). There were 380 to 1672 taxa encountered per sample; the 1672 came from the sample with 10,239,120 microbial reads, but the 380 came from a sample with the third lowest total read count. Over all the replicates, Pearson’s correlation coefficient was 0.735 for taxon count to read count. Rarefaction to 100,000 reads retained 89 of 95 samples, and the counts of observed genera did not reach saturation within that interval when samples were split by biotype, carrier status, presence of RhPV, or relative time (Figure 4). The rarefaction curves were nearly superimposed for splits by biotype, carrier status, and presence of RhPV; they were separated only for splits of samples by relative time. A plot of counts of genera versus total counts of reads (Figure 5) revealed wide variation with continued increase in the number of genera into the millions of reads.

### 3.2. Diversity

**Figure 6:**
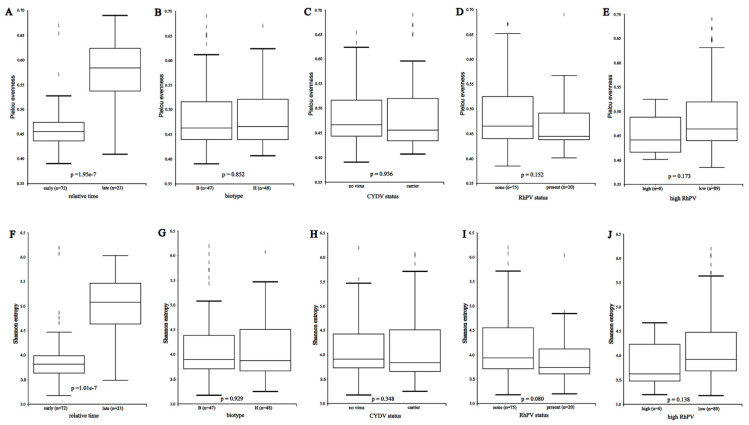
Within-community diversity, as measured by Pielou evenness in panels (**A**–**E**) and Shannon entropy in panels (**F**–**J**). Panels (**A**,**F**) compare collections at days 15 and 20 post-infestation to all earlier collections. Panels (**B**,**G**) compare biotypes B and H. Panels (**C**,**H**) compare CYDV-free and CYDV-carrier conditions. Panels (**D**,**I**) compare the presence with absence of RhPV counts, while panels (**E**,**J**) compare samples with fewer than 100 Rhopalosiphum padi virus counts to samples with more than 100.

For all genera, including *Buchnera*, taxonomic diversity within samples was estimated with Pielou evenness and Shannon entropy after rarefaction to the lowest read count (16,470). The distribution of diversity estimates was displayed as boxplots (Figure 6A–J) with the Kruskal–Wallis *p*-value for pairwise comparisons involving early versus late time (days 0 through 10 versus days 15 and 20), biotypes, CYDV carrier status, RhPV infection, and RhPV infection with more than 100 RhPV counts in the sample (range 127 to 789,356). Only late versus early collection dates differed significantly in either index (Figure 6A,F). Both indices increased as overall microbial abundance decreased on days 15 and 20. The observed evenness values indicated low to moderate within-community diversity with a few abundant genera. The observed Shannon values indicated high to very high within-community diversity because of the large number of rare genera encountered. Increasing the rarefaction limit from 16,470 to 100,000 negligibly affected the evenness or Shannon values, which approached a limit with only 2000 to 4000 reads.

**Figure 7:**
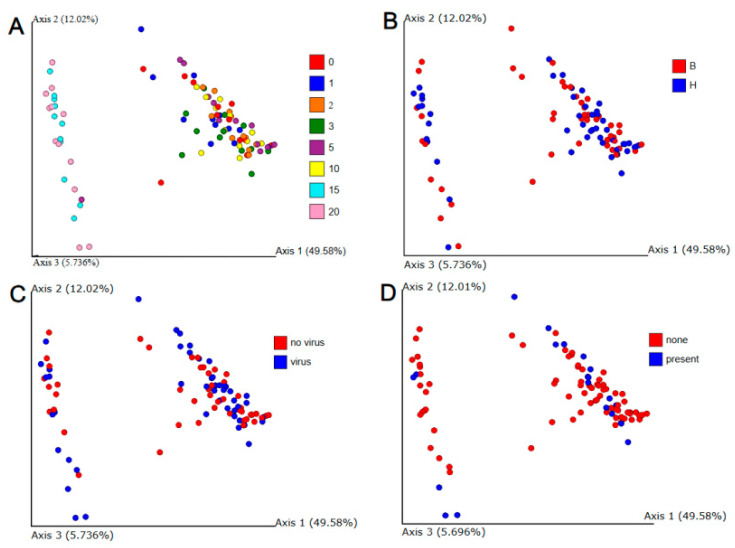
Principal components plot of Bray–Curtis dissimilarities among all individual replicates. Collection dates are encoded in color in panel (**A**). Biotypes are coded in color in panel (**B**), while CYDV carrier status is coded in panel (**C**) and RhPV presence is coded in panel (**D**). The colors are thoroughly interspersed in panels (**B**–**D**).

For all genera, including *Buchnera*, taxonomic diversity between samples was estimated with the Bray–Curtis dissimilarity after rarefaction to 16,470 reads. The first three principal components were plotted (Figure 7) with color-coding for collection dates, biotype, CYDV carrier status, and RhPV infection status. All four panels display the same plot, but only panel A (early versus late collection) shows separation of colors into disjunct groups with a single exception for one sample from day 5. This result also supports a fundamental difference between microbial communities on days 15 and 20 versus earlier samples. Increasing the rarefaction limit from 16,470 to 100,000 eliminated six spots but otherwise negligibly affected the principal components plot. Application of PERMANOVA in Adonis [39] to Bray–Curtis distances also showed that only early versus late time affected the communities significantly, although the interactions of biotype with time and viral carrier status with time were almost significant at *p* = 0.05 (Table 1).

### 3.3. Generic Composition

**Figure 8:**
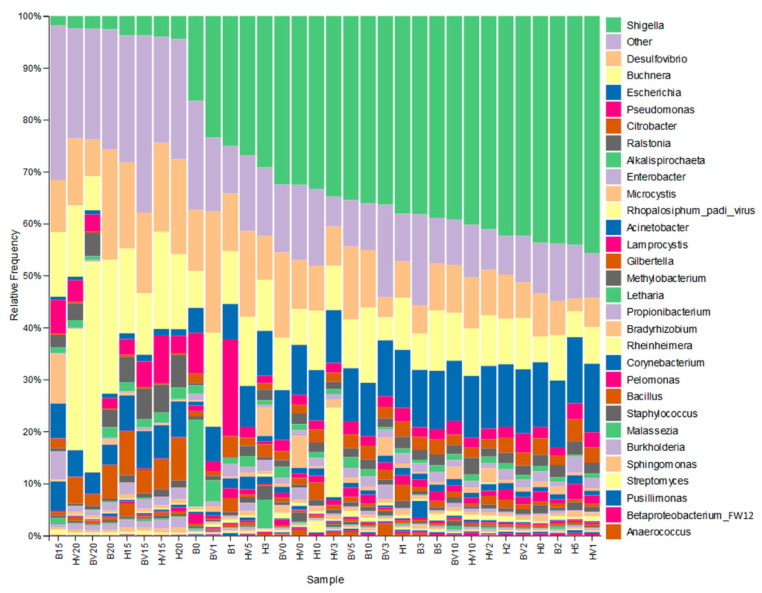
Barplotted relative frequencies of the top 30 microbial genera as defined by total counts over all timepoints and treatments. Samples are encoded B or H for greenbug biotype, V if carrying CYDV-RPV, and 0 to 20 for days post-infestation. “Other” is the sum of counts of all other genera.

The 30 most abundant genera are listed in Figure 8. Supplemental Figures S1–S4 list the top 30 families, orders, classes, and phyla. In Figure 8, “other” is the sum of all other genera, and the size of each bar is proportional to the counts in each combination of biotype, CYDV-carrier status, and collection date. Aside from *Buchnera,* the most abundant genera are typical fecal bacteria that have been reported in vertebrates as well as other insects: *Shigella*, *Desulfovibrio*, *Escherichia*, *Citrobacter*, *Enterobacter*, and *Acinetobacter*. *Pseudomonas* and *Ralstonia* have also been found in mammalian feces. In contrast, *Microcystis* and *Lamprocystis* are photosynthetic, although the latter is anaerobic. A third photosynthetic alga, *Nannochloropsis*, was far less abundant. Figure 8 indicates two distinct communities that differ in the abundance of *Shigella* and *Escherichia*, which decreased on days 15 and 20. These two communities correspond to the disjunct Bray–Curtis clusters in Figure 7.

Taxa varied greatly in counts among samples, even replicates, reflecting seemingly random outbreaks of particular organisms. One measure of such outbreaks is the ratio of the maximum count in any one sample to the total count over all samples. This measure is given in Table 2 for 58 genera that were represented by at least 100,000 reads. The most unevenly encountered genera were the fungi *Mucor* and *Letharia*. For *Mucor*, 98.5% (117,316) of counts came from one replicate of greenbug biotype B harboring CYDV-RPV at 5 days, and no other sample exceeded 250 counts. For *Letharia*, 97.9% (753,389) of counts came from one replicate of greenbug biotype B without harbored CYDV-RPV at 0 days, and five other samples had between 1000 and 5000 counts. Bacterial genera *Fibrisoma* (95.9%), *Variovorax* (88.7%), *Pantoea* (80.9%), and *Serratia* (74.8%) also had more than 70% of their total counts from one sample. At the other extreme, one fungus (*Gilbertella*, 4.3%) and eight bacteria (*Burkholderia* (3.5%) through *Shigella* (5.3%) and *Escherichia* (5.0%)) had no more than 6% of their total counts in any one sample. *Buchnera* was intermediate in stability, with 21% of its total counts in its greatest sample. Count ratios of *Buchnera* to *Schizaphis* varied 35-fold from 0.0002 to 0.0069 among samples. In comparison, the count ratios of *Shigella* to *Schizaphis* varied 290-fold from 0.0000522 to 0.015.

Stability is the opposite of outbreaking, and a set of sufficiently stably abundant genera could constitute a core microbiome if it exists. One measure of stability is *S* = min(*n_i_*/*N_i_*)/max(*n_i_*/*N_i_*), where *n_i_* is the count of the taxon in sample *i* and *N_i_* is the total read count of sample *i*. *S* lies in the interval [0, 1], with higher values denoting greater stability. Supplemental Table S1 lists values of *S* in descending order for 58 genera that have at least 100,000 summed counts over all samples. Even the most stable genera have a ten-fold variation among samples, and therefore, a core microbiome seems not to exist. However, 50 of these genera have non-zero counts in every sample, as reflected in *S* and in the presence across all columns in Figure 8.

Eleven genera of *Enterobacteriaceae* had at least 10,000 total counts. To examine the similarity of their response to that of *Shigella*, the fraction of each one’s counts to total microbial counts was calculated for each sample. Then each fraction was divided by the maximum value of that fraction for that taxon. The mean of such fractions of maximum fractions was then calculated over days 0 through 10 and for days 15 and 20. Table 3 gives the results. *Shigella* and *Escherichia* had the biggest drop, but *Citrobacter*, *Enterobacter*, *Klebsiella*, *Raoultella*, and *Cedecea* also dropped greatly on days 15 and 20. In contrast, *Salmonella*, *Kluyvera*, *Blochmannia*, and *Leclercia* increased on days 15 and 20. Of note, the four most abundant genera of *Enterobacteriaceae* all dropped on days 15 and 20.

Manzano-Marin et al. [41] mentioned eight genera of secondary bacterial symbionts from aphids at large. We detected all of these, although only *Erwinia*, *Serratia*, *Arsenophonus*, *Sodalis*, and *Wolbachia* had more than 10,000 counts. *Fukatsuia*, *Hamiltonella*, and *Regiella* had fewer than 20 counts over the entire study. Many species in *Erwinia*, *Serratia*, and *Sodalis* are free-living and not symbiotic.

Oddly, CYDV-RPV was not detected among reads, even though the wheat plants with viruliferous greenbugs developed yellow dwarf disease. The most abundant virus by counts was Rhopalosiphum padi virus, an aphid pathogen that was detected in 20 samples, of which 18 had CYDV-viruliferous greenbugs that accounted for 99.996% of the RhPV counts. The RhPV-positive samples were split 9:11 between biotypes B and H. The other most abundant insect virus was Diolcogaster facetosa bracovirus (ranked 797th out of all counts). Four bacteriophages that affect *Enterobacteriaceae* were also among the top ten viruses: Shigella phage SfIV (685th), Escherichia phage 500465-1 (831st), Enterobacteria phage DE3 (883rd), and Enterobacteria phage P7 (984th). Overall, there were counts for 301 phage accessions. Many appeared only as a few hundred counts in one sample.

Raw counts of genera were subjected to DESeq2 [37] to identify genera with significant response to time, biotype, or harbored CYDV-RPV. The time comparisons included each timepoint to day 0 and also comparisons between early and late (days 15 and 20) relative time. The results are summarized in Table 4 and appear in full in Supplemental Tables S2–S9. Even though biotype and CYDV did not affect communities overall, individual genera were up- or down-expressed (populated) with both. Some genera responded significantly in more than one comparison and therefore appear more than once in Table 4. In the comparison of timepoints to day 0, there were 299 significantly differentially populated genera, of which 182 were down-populated at late times and 117 were up-populated at later times. In the comparison of late to early relative timepoints (days 15 and 20 versus all previous days), there were 302 significantly differentially populated genera, of which 173 were down-populated and 129 were up-populated. *Shigella*, *Escherichia*, and *Citrobacter* were the most abundant down-populated genera on days 15 and 20, while *Aquabacterium* was a prominent up-populated genus. In the comparison of biotypes, there were 25 significantly differentially populated genera, of which 24 were down-populated in biotype H. The most abundant of these down-populated genera was *Pseudomonas*. In the comparison of CYDV-carrier with CYDV-free greenbugs, there were nine significantly differentially populated genera, of which six Wwere down-populated in CYDV-carriers. *Microvirga* was the most abundant down-populated genus, while *Chryseobacterium* was the most abundant up-populated genus.

## 4. Discussion

Our findings contrast with most rRNA amplicon-based studies in aphids, where *Buchnera* accounts for 70–99% of counts. In our study, *Buchnera* ranked after *Shigella* and *Desulfovibrio* and was just one of several dominant genera. A plausible reason for this discrepancy is the high ploidy of *Buchnera* bacteroids, which have 60 to 200 or more circular genomes per cell [42]. However, *Buchnera* genomes have lost numerous genes during the course of evolution [43], so there are fewer genes being transcribed. PCR amplification of 16S rDNA reflects the copy number of the rRNA genes, which have been retained. We found a high diversity of taxa, and sampling even millions of reads did not fully exhaust the roster of taxa, as evidenced by the rarefaction curves in Figure 4 and their endpoints in Figure 5. The greenbugs and their environment contained a large number of infinitesimally rare, possibly dispersing taxa. The high diversity that we found is most similar to the findings of He et al. [31] in *Myzus persicae* fed on Chinese cabbage, eggplant, tobacco, and pepper. There, *Pseudomonas* outnumbered *Buchnera* on two diets, and *Ralstonia*, *Burkholderia*, *Acinetobacter*, and *Allobaculum* were prominent. All of these genera except *Allobaculum* were among the 30 most abundant genera in greenbugs. However, the *Myzus* microbiome lacked the abundant Enterobacteriaceae (*Shigella*, *Escherichia*, *Citrobacter*, *Klebsiella*) found in greenbugs. Sharawi [44] cultured *Escherichia coli* and single species of *Serratia*, *Bacillus*, *Micrococcus*, and *Staphylococcus* from *Myzus persicae*, indicating the presence of these genera in *Myzus*. All of these bacterial genera were abundant, with more than 85,000 counts in greenbugs. The great preponderance of *Buchnera* in reported aphid microbiomes also implies that the aphid gut lumen is relatively free of bacteria, upholding the finding by Douglas [45] that the gut lumen of *Myzus persicae* was devoid of microorganisms when embedded, sectioned, and examined with light microscopy. However, unconsolidated material can be lost from deparaffinized sections during washing and staining, so confirmation with plastic thick (1 µm) sections (or scanning electron microscopy after freeze-fracturing) is warranted. The transit time of ingested sap through the gut is important in relation to the doubling time of the gut microorganisms. If the transit time is short, then microbial populations might be reduced, although this does not preclude high diversity.

The collection procedure was intended to prevent injury to the greenbugs prior to freezing, so as to avoid wound responses in their transcriptome. Thus, leaf segments bearing greenbugs were frozen, and then the greenbugs were removed. The leaf segment was frozen concomitantly and became brittle, so the greenbug samples were contaminated with wheat and its resident microbiome. This is evident from the percentages of wheat (1.233%) and microbial (1.149%) counts among total counts. There is no spatial information in the counts themselves to indicate which organisms were in the gut, on the exoskeleton, in excreted honeydew, or on/in the leaf. The microbiome that we found was a superset of greenbug and wheat foliar microbiomes and thus was more diverse than microbiomes reported from washed aphids. However, the preponderance of Enterobacteriaceae and *Desulfovibrio* indicates that our greenbug microbiome was mostly from the gut, since these enteric taxa are not abundant in foliar microbiomes. Cernava et al. [46] found Enterobacteriaceae to comprise a minor part of the internal leaf microbiome of *Eruca sativa*, where most read counts from genomic DNA mapped to Pseudomonadales, Burkholderiales, Actinobacteria, and Rhizobiales. However, enteric genera including *Shigella*, *Escherichia*, *Salmonella*, and *Citrobacter* were encountered when the 16S V3-V8 region was amplified with enterobacterial primers Entero-F234 and Entero-R1423. *Buchnera* and *Candidatus Hamiltonella* were also amplified, indicating unacknowledged entrainment of aphids with the sampled leaves. In wheat, Mao et al. [47] found prominent unclassified Alcaligenaceae along with *Lactiplantibacillus*, *Lactobacillus*, *Streptococcus*, and *Pseudomonas*. *Pantoea* was the only genus mentioned from the Enterobacterales.

Our study was designed to investigate the relative effects of greenbug genotype, acquired CYDV-RPV, and time since population establishment, on the diversity and identity of microbes in or on the greenbugs. Greenbug biotypes are defined by their ability to colonize wheat lines with different aphid resistance genes [48]; there is no assurance that different biotypes are genetically close to or distant from one another, and there is no assurance of genetic uniformity within a biotype collected from different locations. However, our study took advantage of the parthenogenetic reproduction of greenbugs to assure genetic uniformity within its chosen biotypes. One weakness in our study was that the presence of CYDV-RPV in the host plants was not confirmed by primer-specific PCR late in the time course. That presence had been confirmed in a previous generation of wheat before the viruliferous greenbugs were placed on healthy wheat [16], and the plants that received viruliferous greenbugs appeared to develop yellow-dwarf disease, but the absence of detected CYDV in the collected greenbugs is concerning. Possibly, the library preparation procedure selected against CYDV, which lacks a polyA tail [49], and allowed recovery of RhPV, which has a polyA tail [50]. The time course spanned the lifespan of wheat under heavy greenbug feeding. The host plants were dying by day 20, and the enteric-depleted community of days 15 and 20 likely resulted from greenbug starvation. This would be especially likely if the previously abundant Enterobacteriaceae had a higher nutritional requirement than the other genera that succeeded them, and it is consistent with the overall microbial population decline on days 15 and 20. However, the differential “expression” of 25 genera between biotypes and nine genera after CYDV acquisition implies that subtle effects might exist for both factors.

The presence of RhPV was unexpected, as was its strong correlation to CYDV acquisition (99.996% of RhPV counts were in the greenbugs that carried CYDV). According to the NCBI taxonomy browser, CYDV and RhPV belong to different orders within the *Pisoniviricetes*, but their genomes have no homology by blastn alignment at an e-value of 1 × 10^−8^. Instead, three hypotheses are plausible: (1) that RhPV was present among the founders of both CYDV-acquiring subpopulations, perhaps having been transmitted via the plant from the aphids that started the CYDV infection in the wheat, since RhPV is mobile within wheat plants [32]; (2) that a diet of CYDV-infected wheat weakens innate immunity to RhPV; and (3) that acquisition of CYDV in itself makes greenbugs more susceptible to RhPV, perhaps by suppressing innate immunity to other viruses. This correlation merits experimental investigation.

Bioinformatic methodology might also have increased the apparent diversity in our study. Some studies explicitly eliminated rare taxa from the analysis of diversity. Most microbiome studies are based on amplification of hypervariable regions in 16S-23S rRNA genes or ITS sequences in 18S rRNA, with merging of overlapping paired-end reads, removal of PCR chimeras, and clustering or denoising to consensus or most abundant similar sequences over some narrow range (e.g., 3%; see [26], and the documentation of QIIME2) of sequence variability. That strategy combats amplification and sequencing error, but it also implicitly assumes that closely related taxa are not present in the community and that amplification is equally effective for all variants. Extensive databases (Silva, UNITE, GreenGenes) exist for such amplicons, many of which come from uncultured organisms. Having mRNA in hand, with no expectation of chimeric reads in the absence of PCR amplification, we chose instead to align each forward or reverse read to all publicly available whole genomes of viruses, bacteria, archaea, fungi, and protists, letting competition among closest hits determine the taxonomic assignment of each read. It is therefore possible that reads derived from conserved genes mapped randomly to multiple taxa, with ties broken in favor of the first of the identical hits. However, genome assemblies are expensive compared to amplicon sequencing and, therefore, might be biased to medically or agriculturally important organisms. The open question is how frequently erroneous reads more closely map to some related organism, which would inflate the number of hit taxa. Contamination from the laboratory or extraction kits can lead to amplification from common environmental bacteria, such as *Ralstonia*, *Afipia*, *Pseudomonas*, *Acinetobacter*, and *Burkholderia*, in blank samples subjected to ca. 30 cycles of PCR [51], but such contamination is swamped out with more than 10^7^ cells in a sample [51] and is not expected to be discernible in unamplified RNA-seq samples. Therefore, “kitome” contamination probably did not affect the apparent presence of genera in our study.

The presence of more than 10,000 counts each of *Erwinia*, *Serratia*, *Arsenophonus*, *Sodalis*, and *Wolbachia* suggests that one or more of these genera might be a secondary symbiont in greenbugs, since these genera contain secondary symbiotic taxa in at least six lineages of aphids [41]. However, *Schizaphis* is not in any of these lineages, and only six of the *Serratia* reads came from *Serratia symbiotica.* In contrast, Ma et al. [26] also detected *Serratia*, *Arsenophonus*, and *Wolbachia* in *Aphis gossypii*, which, like greenbugs, is in the tribe *Aphidini*. Proving endosymbiosis would require demonstrating the presence of the bacterial genome within bacteroids in bacteriocytes. Even if an intracellular relationship does not exist, these and other bacteria might supply B-vitamins from the gut lumen. Another hemolymph-resident bacterium, *Spiroplasma* [52], was scarcely detected with a total of 47 counts. Therefore, *Spiroplasma* is unlikely to be a greenbug symbiont.

RNA is an imperfect proxy for cell counts. Transcription rates and mRNA stability surely vary among genes and taxa, and RNA counts per cell depend on cell volume, which is greater in eukaryotes than in bacteria. There is also a contrast between multicellular, filamentous fungi and unicellular bacteria and archaea. Cell or organism counts based on amplicon reads can be skewed by ploidy variation, as mentioned above. Sequence complexity (entropy) can also affect the range of matching sequences in reference genomes. We had to filter out low-entropy reads (long microsatellites, low-quality runs of single nucleotides) with bbduk because such reads were leading non-specifically to implausible hits (e.g., corals) for greenbugs in a growth chamber. Nevertheless, one of our top 30-ranking genera, *Letharia*, was implausibly present even after entropy filtration. *Letharia* is a lichenized ascomycete that grows on tree branches in the Pacific Northwest of the USA [53]. It is possibly a contaminant that was somehow introduced after sample collection.

In summary, taxon counts obtained from microbial mRNA in greenbugs differed markedly from counts obtained with 16S rDNA amplicons from related aphid species, indicating a much more diverse microbiome with reduced dominance of *Buchnera aphidicola*. It is not obvious why this should be so, although the high ploidy of *Buchnera* probably has a role. It will be difficult to understand the biological significance of this difference until results are obtained with both methods from the same aphid samples. The mRNA method revealed a population collapse of enteric bacteria as the host plants succumbed to aphid feeding. This collapse was more evident because the ratio of microbial to greenbug read counts was available, which would not have been the case with amplicon sequencing.

## Figures and Tables

**Table 1:** Significance of Bray–Curtis distances among communities in response to time, biotype, and harbored CYDV-RPV.

Factor *	r^2^	*p*-Value
rel_time	0.459	0.001
biotype	0.007	0.567
viral_status	0.006	0.690
biotype:rel_time	0.014	0.067
viral_status:rel_time	0.014	0.068
biotype:viral_status	0.010	0.408

* The value of rel_time (relative time) is early (days 0 through 10) or late (days 15 and 20). Viral status is harboring CYDV-RPV or free of CYDV-RPV. Colons indicate interactions. The r^2^ is a correlation coefficient.

**Table 2:** Maximum fraction of counts in a single sample for genera with at least 100,000 reads.

Genera	Maximum Fraction of Counts
*Mucor*	0.9846
*Letharia*	0.9785
*Fibrisoma*	0.9589
*Variovorax*	0.8873
*Pantoea*	0.8093
*Serratia*	0.7483
Rhopalosiphum padi virus	0.5483
*Vibrio*	0.4438
*Malassezia*	0.3932
*Legionella*	0.3622
*Anaerococcus*	0.3426
*Corynebacterium*	0.3395
*Novosphingobium*	0.3354
*Alkalispirochaeta*	0.3174
*Micrococcus*	0.3162
*Desulfovibrio*	0.2465
*Erwinia*	0.2308
*Kocuria*	0.2276
*Nocardioides*	0.2204
*Buchnera*	0.2060
*Pelomonas*	0.1734
*Arthrobacter*	0.1578
*Microcystis*	0.1565
*Streptococcus*	0.1545
*Staphylococcus*	0.1502
*Propionibacterium*	0.1388
*Klebsiella*	0.1315
*Caulobacter*	0.1308
*Brevundimonas*	0.1246
*Clostridium*	0.0996
*Sphingomonas*	0.0869
*Bradyrhizobium*	0.0799
*Microbacterium*	0.0796
*Pseudomonas*	0.0687
*Acidovorax*	0.0679
*Providencia*	0.0610
Betaproteobacterium FWI2	0.0608
*Rheinheimera*	0.0602
*Streptomyces*	0.0590
*Flavobacterium*	0.0585
*Actinomadura*	0.0559
*Afipia*	0.0552
*Stenotrophomonas*	0.0552
*Marinobacterium*	0.0545
*Enterobacter*	0.0532
*Shigella*	0.0530
*Aquabacterium*	0.0506
*Lamprocystis*	0.0506
*Ralstonia*	0.0504
*Escherichia*	0.0497
*Methylobacterium*	0.0480
*Pusillimonas*	0.0465
*Terrisporobacter*	0.0460
*Acinetobacter*	0.0446
*Citrobacter*	0.0445
*Gilbertella*	0.0425
*Bacillus*	0.0384
*Burkholderia*	0.0350

**Table 3:** Response of *Enterobacteriaceae* to time.

Genus	Total Count	Mean Days 0–10	Mean Days 15–20
*Shigella*	36,995,790	0.6859	0.0661
*Escherichia*	10,750,123	0.6825	0.0711
*Citrobacter*	2,335,059	0.4983	0.0670
*Enterobacter*	1,621,481	0.4626	0.0742
*Klebsiella*	264,331	0.5574	0.1801
*Salmonella*	72,442	0.2627	0.3421
*Kluyvera*	41,571	0.1827	0.3337
*Raoultella*	12,306	0.4437	0.0908
*Blochmannia*	11,825	0.0302	0.0770
*Leclercia*	11,269	0.0954	0.3715
*Cedecea*	10,269	0.2655	0.0082

**Table 4:** Genera that responded most strongly to varied factors ^a^.

Taxon	Variable	Direction	Base_Mean	Log2_Fold_Change	Adj_*p*-Value
*Shigella*	time	down	210,548.6	−4.57	8.59 × 10^−136^
*Escherichia*	time	down	61,277.55	−4.5	2.19 × 10^−124^
*Citrobacter*	time	down	14,367.69	−4.19	3.17 × 10^−74^
*Shigella*	rel_time	down	210,548.6	−4.6	1.31 × 10^−146^
*Escherichia*	rel_time	down	61,277.55	−4.48	2.38 × 10^−134^
*Citrobacter*	rel_time	down	14,367.69	−4.07	1.99 × 10^−82^
*Letharia*	biotype	down	146.68	−3.69	1.73 × 10^−7^
*Aequitasia*	biotype	down	109.78	−6.26	1.18 × 10^−5^
*Salipaludibacillus*	biotype	down	74.46	−2.71	1.86 × 10^−5^
*Microvirga*	virus	down	228.96	−2.46	5.66 × 10^−6^
*Salipaludibacillus*	virus	down	74.46	−2.61	4.20 × 10^−5^
*Letharia*	virus	down	101.13	−2.57	6.54 × 10^−4^
*Aquabacterium*	time	Up	1382.08	2.14	3.32 × 10^−68^
*Herbaspirillum*	time	Up	591.39	2.05	1.74 × 10^−55^
*Delftia*	time	Up	511.92	1.54	6.12 × 10^−51^
*Aquabacterium*	rel_time	Up	1382.08	2.3	9.23 × 10^−73^
*Herbaspirillum*	rel_time	Up	591.39	1.85	1.20 × 10^−57^
*Delftia*	rel_time	Up	511.92	1.75	9.20 × 10^−55^
*Microvirga*	biotype	Up	140.13	1.57	2.03 × 10^−3^
*Massilia*	biotype	Up	397.05	0.91	2.09 × 10^−2^
*Porphyromonas*	biotype	Up	55.79	1.09	3.33 × 10^−2^
*Blochmannia*	virus	Up	14.48	5	1.12 × 10^−12^
*Izhakiella*	virus	Up	17.35	5.94	1.83 × 10^−11^
*Chryseobacterium*	virus	Up	293.1	0.91	2.07 × 10^−4^

^a^ Time is time in days, while rel_time is early (days 0–10) or late (days 15 and 20). Down means lower counts with later time, lower counts with biotype H, and lower counts with harbored CYDV-RPV. The listed genera are the three most extreme for each variable and direction of fold-change.

## Data Availability

The Illumina reads reside in NCBI SRA under bioproject PRJNA981508 (www.ncbi.nlm.nih.gov/bioproject/PRJNA981508, URL first accessed 8 June 2023). There are 94 runs from SRR25033616 through SRR25033709. Read counts and scripts have been deposited at https://doi.org/10.5061/dryad.z08kprrp1.

## Supplementary Materials

The following supporting information can be downloaded at: https://www.mdpi.com/article/10.3390/insects16060554/s1, Figure S1. Barplotted relative frequencies of counts of the top 30 microbial families plus “Other” as the sum of all other families. Samples are encoded B or H for greenbug biotype, V if viruliferous, and 0 to 20 for days after infestation. Late depletion of *Enterobacteriaceae* is still evident. Figure S2. Barplotted relative frequencies of counts of the top 30 microbial orders plus “Other” as the sum of all other orders. Samples are encoded B or H for greenbug biotype, V if viruliferous, and 0 to 20 for days after infestation. The *Shigella*-depleted community is less distinct as more genera are grouped under *Enterobacteriales*. Figure S3. Barplotted relative frequencies of counts of the top 30 microbial classes plus “Other” as the sum of all other classes. Samples are encoded B or H for greenbug biotype, V if viruliferous, and 0 to 20 for days after infestation. The *Shigella*-depleted community is even less distinct. Various fungal classes appear when orders are grouped into bacterial classes. Figure S4. Barplotted relative frequencies of counts of the top 30 microbial phyla plus “Other” as the sum of all other phyla. Samples are encoded B or H for greenbug biotype, V if viruliferous, and 0 to 20 for days after infestation. There is a nearly continuous downward gradation in frequency of *Pseudomonadota* at later timepoints. Table S1. Stability and total counts of 58 abundant genera ranked by stability index *S*. Table S2. DESeq2 results for comparison by time, arranged by BH-adjusted *p*-value. Table S3. DESeq2 results for comparison by time, arranged by log_2_ fold change. Table S4. DESeq2 results for comparison by biotype, arranged by BH-adjusted *p*-value. Table S5. DESeq2 results for comparison by biotype, arranged by log_2_ fold change. Table S6. DESeq2 results for comparison by viral carrier status, arranged by BH-adjusted *p*-value. Table S7. DESeq2 results for comparison by viral carrier status, arranged by log_2_ fold change. Table S8. DESeq2 results for comparison by early versus late relative time, arranged by BH-adjusted *p*-value. Table S9. DESeq2 results for comparison of early versus late relative time, arranged by log_2_ fold change.
