# Supplementary material for: Shotgun Metagenome Analysis of Two Schizaphis graminum Biotypes over Time With and Without Carried Cereal Yellow Dwarf Virus
Source: Insects. 2025 May 23;16(6):554. doi: 10.3390/insects16060554 (PMC12193481; doi:10.3390/insects16060554)
Supplement: Supplementary file 1 [file insects-16-00554-s001.zip › Table S1.pdf]

Table S1. Stability and total counts of 58 abundant genera ranked by stability index *S*.

Genus	*S*	Total
Providencia	0.0994	135,115
Bradyrhizobium	0.0913	710,069
Buchnera	0.0707	1,1187,991
Microbacterium	0.0696	189,227
Flavobacterium	0.0569	102,904
Klebsiella	0.0556	264,331
Stenotrophomonas	0.0527	149,542
Acidovorax	0.0456	103,411
Methylobacterium	0.0440	843,421
Sphingomonas	0.0422	307,221
Desulfovibrio	0.0409	11,961,445
Burkholderia	0.0378	319,332
Alkalispirochaeta	0.0363	1,666,962
Rheinheimera	0.0352	694,281
Enterobacter	0.0341	1,621,481
Caulobacter	0.0324	168,885
Actinomadura	0.0321	131,141
Legionella	0.0312	266,771
Pseudomonas	0.0306	3,098,401
Citrobacter	0.0274	2,335,059
Pusillimonas	0.0244	283,278
Bacillus	0.0236	428,815
Afipia	0.0232	160,702
Acinetobacter	0.0213	1,437,672
Shigella	0.0208	36,995,790
Escherichia	0.0202	10,750,123
Brevundimonas	0.0185	165,154
Erwinia	0.0168	134,876
Aquabacterium	0.0157	141,175
Lamprocystis	0.0138	1,326,056
Streptococcus	0.0134	252,825
Pelomonas	0.0129	450,679
Propionibacterium	0.0119	759,362
Arthrobacter	0.0115	103,696
Nocardioides	0.0088	134,781
Ralstonia	0.0087	2,046,817
Corynebacterium	0.0085	589,305
Staphylococcus	0.0081	421,439
Clostridium	0.0071	188,885
Micrococcus	0.0068	121,999
Streptomyces	0.0066	301,603
Vibrio	0.0062	140,085
Microcystis	0.0034	1,474,003
Marinobacterium	0.0030	208,745
Gilbertella	0.0020	1,206,934

Malassezia	0.0019	324,363
Serratia	0.0018	125,750
Pantoea	0.0006	190,407
Novosphingobium	0.0002	120,716
Kocuria	0.0001	100,885
Mucor	0	117,316
Terrisporobacter	0	119,031
Rhopalosiphum padi virus	0	1,439,602
Anaerococcus	0	269,738
Letharia	0	769,927
Fibrisoma	0	104,441
Betaproteobacterium FWI2	0	273,255
Variovorax	0	154,817
