# Supplementary material for: Shotgun Metagenome Analysis of Two Schizaphis graminum Biotypes over Time With and Without Carried Cereal Yellow Dwarf Virus
Source: Insects. 2025 May 23;16(6):554. doi: 10.3390/insects16060554 (PMC12193481; doi:10.3390/insects16060554)
Supplement: Supplementary file 1 [file insects-16-00554-s001.zip › Table S2.pdf]

Table S2. DESeq2 results for comparison by time, arranged by BH-adjusted p-value.

Genus	BaseMean	Log2FC	LFCSE	Padj
Shigella	210548.549	-4.567	0.225	8.589e-136
Escherichia	61277.548	-4.504	0.231	2.186e-124
Citrobacter	14367.687	-4.186	0.275	3.165e-74
Klebsiella	1531.102	-2.847	0.217	3.317e-68
Aquabacterium	1382.076	2.138	0.233	3.317e-68
Terrisporobacter	682.815	-4.583	0.330	3.111e-64
Lamprocystis	7995.423	-4.774	0.342	3.006e-61
Herbaspirillum	591.387	2.055	0.206	1.741e-55
Delftia	511.917	1.537	0.200	6.117e-51
Enterobacter	9658.205	-3.798	0.318	3.461e-48
Pusillimonas	1729.460	-3.117	0.286	6.018e-48
Cedecea	62.532	-5.388	0.541	6.088e-47
Oryzomicrobium	63.319	-5.855	0.609	8.629e-47
Lentisphaera	29.992	-3.433	0.419	1.818e-38
Phytophthora	90.681	-6.648	0.610	4.356e-37
Tannerella	323.303	-3.638	0.384	1.956e-35
Marinobacterium	1297.016	-4.935	0.466	6.710e-33
Actinobacillus	273.019	-4.055	0.421	4.415e-32
Rheinheimera	4037.545	-3.039	0.326	6.446e-32
Raoultella	68.520	-3.271	0.414	7.355e-32
Leptothrix	258.760	2.980	0.425	3.041e-31
Elizabethkingia	205.340	-3.108	0.334	7.336e-31
Serratia	400.268	-2.650	0.497	1.210e-26
Musicola	448.106	-4.622	0.499	7.898e-25
Mycolicibacterium	115.168	2.645	0.440	6.307e-22
Craterilacuibacter	127.435	-5.720	0.740	1.312e-21
Gleimia	229.791	-5.729	0.592	2.253e-21
Ruficoccus	65.274	-3.493	0.494	5.979e-21
Comamonas	377.215	1.916	0.295	1.222e-20
Burkholderia	2610.408	1.256	0.204	1.287e-20
Marinobacter	150.235	-2.559	0.353	1.545e-20
Erwinia	905.375	-3.296	0.425	2.348e-20
Bacteroides	124.758	-2.686	0.334	1.113e-19
Ascochyta	1160.970	4.027	1.097	1.419e-19
Pseudochrobactrum	75.819	3.700	0.642	3.415e-19
Paracidovorax	147.082	1.824	0.459	4.100e-19
Labrys	177.824	4.979	0.685	9.501e-19
Moritella	186.504	-1.919	0.241	6.636e-18
Paraburkholderia	685.653	1.328	0.225	1.969e-17
Parastagonospora	616.798	4.797	0.991	8.231e-17
Enterobacteriaceae_genus	24.824	-1.267	0.420	9.941e-17
Macroventuria	247.054	3.914	1.094	1.422e-16
Seonamhaeicola	12.679	3.557	0.597	1.872e-16
Rhizobacter	51.463	2.433	0.456	2.037e-16
Edwardsiella	89.782	-3.162	0.609	1.563e-15
Facklamia	31.482	-5.287	0.798	1.886e-15
Piscinibacter	118.721	2.678	0.543	6.442e-15
Acinetobacter	11818.492	1.145	0.225	3.006e-14
Fusobacterium	181.144	-1.955	0.318	3.663e-14
Afipia	1012.669	-2.467	0.371	2.260e-13
Allomeiothermus	372.615	-8.433	1.023	6.125e-13
Ochrobactrum	77.175	2.514	0.549	6.511e-13
Moesziomyces	251.864	-6.995	1.616	7.091e-13
Kallotenue	98.738	-8.434	1.048	1.405e-12
Kurthia	83.993	-3.265	0.549	3.549e-12

Allomuricauda	54.976	-3.554	0.549	5.046e-12
Microbispora	62.361	-2.632	0.474	7.156e-12
Betaproteobacterium_FWI2	1603.185	-3.833	0.536	9.863e-12
Anaerococcus	1493.959	-5.874	0.980	1.301e-11
Nitriliruptoraceae_genus	156.786	4.829	1.356	1.576e-11
Spiribacter	38.734	4.083	0.663	1.582e-11
Rubrivivax	73.057	2.800	0.542	4.673e-11
Letharia	104.390	3.601	0.915	6.220e-11
Brevundimonas	1147.754	1.906	0.372	6.872e-11
Rahnella	196.146	-2.688	0.483	9.639e-11
Proteobacteria	45.108	-0.253	1.111	1.128e-10
Agrobacterium	192.449	1.397	0.297	1.182e-10
Zoogloea	42.077	-4.176	0.618	1.908e-10
Bradyrhizobium	4376.605	-1.593	0.278	2.868e-10
Fusarium	242.795	-2.358	1.012	3.911e-10
Yersinia	118.324	-3.091	0.641	7.917e-10
Lysinibacillus	127.976	-4.248	0.627	9.311e-10
Cytobacillus	13.792	8.194	1.643	9.721e-10
Neofusicoccum	135.521	-2.439	0.968	9.881e-10
Murine_type_C_virus	17.101	3.889	0.807	1.104e-09
Armatimonadetes	144.045	-7.030	1.120	1.805e-09
Proteus	179.703	-3.130	0.580	1.980e-09
Kineococcus	53.880	1.224	1.060	2.101e-09
Polyangium	64.134	3.540	0.890	2.294e-09
Alteromonas	44.223	3.169	0.791	2.984e-09
Pseudomonas	23142.274	-1.402	0.379	3.640e-09
Aerococcus	77.226	-2.647	0.475	4.316e-09
Sinirhodobacter	15.801	1.644	0.519	4.698e-09
Lichtheimia	35.429	1.615	0.798	4.787e-09
Lautropia	241.645	-1.840	0.332	4.814e-09
Salipaludibacillus	74.463	-3.633	0.887	7.240e-09
Ensifer	36.688	-3.396	0.535	9.813e-09
Beijerinckiaceae_genus	37.739	5.614	1.115	1.361e-08
Isoptericola	47.485	1.157	0.567	1.473e-08
Acidithiobacillus	129.947	2.545	0.785	1.653e-08
Glycocaulis	61.217	3.835	1.607	2.040e-08
Kosakonia	32.393	-3.408	0.836	2.474e-08
Guillardia	48.159	0.719	1.576	2.989e-08
Fonsecaea	35.534	0.814	1.089	3.383e-08
Bordetella	34.897	3.815	0.793	3.387e-08
Uncultured	130.743	1.243	0.600	3.616e-08
Tumebacillus	31.849	-3.098	0.704	4.201e-08
Polychytrium	132.053	2.524	1.495	7.462e-08
Diaphorobacter	35.714	-2.404	0.653	7.462e-08
Orbilia	26.431	-5.123	1.464	7.471e-08
Novosphingobium	579.919	1.808	0.519	7.656e-08
Stenotrophomonas	1057.148	0.628	0.183	8.242e-08
Mitosporidium	133.751	1.801	1.939	8.255e-08
Westeberhardia	22.020	-1.667	0.725	8.898e-08
Kitasatospora	49.538	-5.197	1.128	1.254e-07
Kalmanozyma	17.246	-3.333	1.454	1.322e-07
Azospirillum	271.311	2.254	0.577	1.444e-07
Ichthyophthirius	11.762	-5.490	0.994	1.465e-07
Glutamicibacter	58.869	-3.955	0.829	1.789e-07
Caulobacter	1070.743	-1.275	0.397	1.939e-07
Leptosphaeria	79.063	4.159	1.310	1.939e-07
Limnobacter	140.336	2.940	0.994	2.127e-07
Mitsuaria	151.580	2.114	0.538	2.491e-07
Peptoniphilus	314.312	-3.040	1.010	2.739e-07

Exophiala	38.352	2.177	0.996	2.746e-07
Clostridium	1001.246	-1.804	0.314	3.251e-07
Alloscardovia	58.836	-3.054	0.714	3.472e-07
Spizellomyces	92.702	1.416	1.737	3.535e-07
Gordonia	106.567	0.744	0.710	3.547e-07
Pelomonas	2109.665	-1.267	0.298	4.408e-07
Fimicolochytrium	92.915	-1.018	1.780	4.408e-07
Roseburia	44.523	2.550	0.612	4.513e-07
Vibrio	744.207	1.586	0.444	4.757e-07
Aeromonas	111.663	2.117	0.497	5.048e-07
Mycobacteroides	34.731	5.000	1.063	5.457e-07
Corynebacterium	3523.431	-0.740	0.598	5.490e-07
Brevibacterium	127.378	1.667	0.697	5.672e-07
Massilia	397.055	0.980	0.485	5.726e-07
Lobosporangium	154.598	2.870	1.861	1.020e-06
Finegoldia	237.843	-2.946	1.098	1.134e-06
Nostoc	400.525	-2.275	0.494	1.287e-06
Corallococcus	181.760	0.405	0.594	1.333e-06
Kocuria	801.833	2.184	0.831	1.362e-06
Acaromyces	130.080	-2.971	1.695	1.362e-06
Prevotella	338.175	-2.505	0.527	1.567e-06
Bacillus	2815.520	-0.577	0.144	1.692e-06
Roseomonas	144.562	1.230	0.671	2.108e-06
Pseudarthrobacter	88.036	1.887	0.894	2.117e-06
Azotobacter	95.528	-3.819	0.789	2.544e-06
Saccharopolyspora	62.086	-0.937	0.371	2.725e-06
Betaproteobacterium_AAP51	11.054	2.906	0.914	2.725e-06
Shewanella	593.636	-0.994	0.247	3.516e-06
Dietzia	166.910	2.605	0.572	3.568e-06
Photorhabdus	73.809	-1.455	0.773	4.000e-06
Trichosporon	145.713	0.802	1.256	4.086e-06
Rugamonas	51.529	-0.929	0.906	4.086e-06
Morococcus	313.297	-2.376	0.475	4.102e-06
Aequitasia	117.988	-0.087	1.687	4.432e-06
Metarhizium	45.258	-5.018	1.164	4.553e-06
Lawsonella	195.241	-2.686	0.711	5.462e-06
Providencia	883.343	-0.899	0.270	8.543e-06
Microcystis	6710.710	-2.281	0.633	9.528e-06
Sphingopyxis	66.886	2.871	0.687	1.083e-05
Tetrahymena	10.703	-6.831	1.365	1.083e-05
Enterococcus	585.175	-0.572	0.227	1.153e-05
Staphylococcus	2906.978	-1.360	0.618	1.192e-05
Saitoella	68.439	2.297	2.193	1.198e-05
Cloacibacterium	93.517	-1.818	0.795	1.373e-05
Thalassiosira	9.266	-5.165	1.090	1.591e-05
Paucilactobacillus	8.021	-4.092	1.085	1.719e-05
Mycotypha	40.609	6.232	1.889	1.743e-05
Tepidiphilus	149.559	-2.114	1.216	2.209e-05
Ornithinimicrobium	139.539	-0.107	0.805	2.209e-05
Heterobasidion	23.873	-6.272	1.527	2.315e-05
Agrococcus	150.918	2.930	1.123	2.338e-05
Aeromicrobium	61.992	0.659	0.773	2.461e-05
Pseudoxanthomonas	97.563	-2.222	0.611	2.503e-05
Propioniciclava	35.109	-3.345	1.058	2.839e-05
Aureococcus	12.830	-7.445	1.639	2.970e-05
Wallemia	78.999	-0.720	1.703	3.444e-05
Nocardioides	882.762	0.398	0.396	3.767e-05
Paludifilum	60.826	-1.823	0.553	3.997e-05
Micrococcus	653.697	1.859	0.552	4.399e-05

Alkalihalobacillus	4.260	3.634	1.008	4.455e-05
Naegleria	20.303	-3.961	0.895	4.636e-05
Planococcus	196.283	-1.747	0.346	4.662e-05
Nesterenkonia	94.391	-1.570	0.597	5.187e-05
Hydrogenophaga	66.646	-2.801	0.689	5.791e-05
Acetobacter	24.983	-1.295	0.724	6.271e-05
Serinicoccus	39.889	5.922	1.495	6.566e-05
Propionimicrobium	84.480	-1.881	0.897	7.767e-05
Frankia	114.940	-2.350	0.506	8.164e-05
Colletotrichum	63.127	-1.868	0.719	8.605e-05
Salinimicrobium	20.796	1.737	0.809	8.963e-05
Hymenobacter	123.295	1.259	0.586	1.033e-04
Pseudooceanicola	112.214	-1.849	0.939	1.182e-04
Meira	243.896	-1.338	1.934	1.497e-04
harvey_murine_sarcoma_virus	1.591	2.107	1.063	1.657e-04
Dermacoccus	44.902	1.197	0.536	1.728e-04
Roseovarius	20.641	2.831	0.633	1.855e-04
Myceligenerans	6.325	-4.429	1.136	1.868e-04
Aspergillus	116.582	-2.592	0.613	1.889e-04
Pseudacidovorax	63.479	-2.086	0.557	2.104e-04
Lutimaribacter	14.713	-6.223	1.123	2.302e-04
Dyadobacter	35.160	0.690	1.031	2.404e-04
Bifidobacterium	80.934	1.823	0.765	2.477e-04
Duganella	59.734	-1.494	0.655	2.777e-04
Undibacterium	178.266	2.219	0.602	3.299e-04
Janthinobacterium	149.394	1.591	0.432	3.476e-04
Abelson	3.843	1.766	0.843	4.372e-04
Dyella	23.540	-2.934	0.673	4.493e-04
Laccaria	12.373	-2.272	1.557	4.908e-04
Clostridiales	35.068	1.283	0.687	5.803e-04
Rhodobacteraceae_genus	44.452	0.455	0.851	6.054e-04
Microvirga	80.646	1.081	0.554	6.965e-04
Arcticiflavibacter	197.563	2.037	0.585	7.391e-04
Dolosigranulum	32.224	0.094	1.410	7.391e-04
Stereum	19.572	-1.643	1.395	7.446e-04
Neisseria	439.766	-1.444	0.379	7.745e-04
Phytobacter	18.093	-3.012	1.013	7.745e-04
Phaeodactylum	12.627	-5.062	1.093	7.745e-04
Apibacter	18.031	-1.964	1.849	8.164e-04
Janibacter	177.517	-0.813	0.422	8.261e-04
Rhodococcus	520.947	-0.375	0.389	8.417e-04
Nanosynbacter	12.801	-5.360	1.246	8.417e-04
Wenzhouxiangella	8.641	-3.881	1.688	8.873e-04
Bowmanella	86.909	-1.109	0.731	9.553e-04
Saprolegnia	9.754	-5.924	1.208	9.753e-04
Didymella	21.350	3.401	1.703	9.877e-04
Nitrosospira	14.651	4.484	2.350	9.877e-04
Pseudoramibacter	123.529	-3.137	1.005	9.979e-04
Wickerhamiella	8.538	3.021	1.210	1.021e-03
Perkinsus	5.227	-4.766	1.155	1.021e-03
Entamoeba	32.922	0.263	0.870	1.046e-03
Paracoccus	735.536	0.792	0.307	1.077e-03
Glarea	9.663	-4.605	1.448	1.077e-03
Paraclostridium	28.339	0.430	1.463	1.096e-03
Lachnospira	17.322	1.734	0.845	1.096e-03
Gemella	167.060	-1.661	0.758	1.149e-03
Lachnoanaerobaculum	14.139	-3.658	0.829	1.149e-03
Telluria	36.691	-0.975	1.059	1.163e-03
Pseudomicrostroma	29.079	1.156	1.472	1.163e-03

Blastomonas	151.122	-1.052	0.527	1.165e-03
Microlunatus	147.679	2.425	0.945	1.165e-03
Aureibaculum	51.891	-2.363	0.675	1.184e-03
Protofrankia	75.565	-2.236	0.730	1.204e-03
Melampsora	15.381	8.993	2.225	1.208e-03
Geobacillus	32.342	-0.149	1.136	1.284e-03
Mucor	14.488	4.190	1.456	1.306e-03
Micromonospora	101.944	-1.322	0.815	1.348e-03
Toxoplasma	250.863	0.485	0.271	1.374e-03
Ruminococcus	45.967	-2.339	0.999	1.400e-03
Pontibacter	44.467	4.060	2.596	1.482e-03
Sedimentitalea	30.138	1.663	0.479	1.688e-03
Postia	23.021	-21.086	1.663	1.725e-03
Rhizoctonia	14.717	-4.604	1.620	1.750e-03
Paeniglutamicibacter	22.306	-0.115	0.779	1.867e-03
Hyphobacterium	32.142	1.973	0.547	1.895e-03
Salmonella	451.456	-1.194	0.292	1.953e-03
Lentilactobacillus	33.253	5.095	1.917	1.984e-03
Desulfosporosinus	5.604	-3.517	1.282	1.984e-03
Hahella	2.380	-1.949	1.313	1.989e-03
Acidaminobacter	12.532	-2.660	1.309	2.055e-03
Agilicoccus	113.618	-2.580	1.036	2.080e-03
Cutaneotrichosporon	17.445	-5.437	1.661	2.108e-03
Mycetohabitans	9.753	0.931	0.805	2.173e-03
Aureobasidium	23.442	-4.263	1.203	2.359e-03
Empedobacter	31.761	1.705	0.670	2.366e-03
Gardnerella	54.022	0.879	1.211	2.398e-03
Propionibacterium	5198.439	0.372	0.518	2.398e-03
Yarrowia	13.249	3.674	1.352	2.601e-03
Methylobacterium	5697.290	-1.311	0.330	2.620e-03
Pseudozyma	25.743	-3.482	1.486	2.629e-03
Hafnia	2.018	-2.295	0.985	2.743e-03
Microbacterium	1220.119	-0.881	0.247	2.758e-03
Punctularia	29.052	-1.932	1.785	2.851e-03
Paracoccidioides	3.115	-1.798	1.481	2.867e-03
Thermus	128.332	-3.263	0.759	2.918e-03
Alcaligenes	8.410	-0.902	1.201	3.057e-03
Plasmopara	14.723	-4.777	1.426	3.222e-03
Secondary	3.884	-3.407	0.960	3.572e-03
Xenorhabdus	3.486	-3.631	0.918	3.759e-03
Pseudoroseomonas	17.498	2.457	1.165	3.804e-03
Rhodobacter	87.074	-1.742	0.461	3.885e-03
Cupidesulfovibrio	28.579	-2.191	0.755	4.111e-03
Flavobacterium	706.963	-1.182	0.282	4.121e-03
Parasaccharibacter	82.051	-0.131	0.393	4.165e-03
Streptosporangium	49.775	0.450	0.436	4.243e-03
Neoantrodia	18.640	-5.255	1.771	4.826e-03
Pantoea	228.056	-0.803	0.268	4.834e-03
Pedobacter	122.644	-1.822	0.500	4.844e-03
Dictyostelium	11.036	-3.185	1.533	5.345e-03
PreXMRV-1_provirus_complete	4.785	2.361	0.882	5.422e-03
Azospira	27.304	-1.872	0.591	5.747e-03
Terrimonas	78.628	-1.140	1.042	5.797e-03
Aquamicrobium	62.955	-3.061	0.631	5.797e-03
Sodalis	1.787	-1.770	0.849	6.077e-03
Cronobacter	1.817	1.599	1.738	6.592e-03
Sporisorium	4.490	-6.120	2.320	6.825e-03
Pseudogymnoascus	14.590	-4.191	1.820	6.890e-03
Curtobacterium	171.096	0.397	0.712	7.488e-03

Bipolaris	14.314	-4.947	1.629	7.729e-03
Adhaeribacter	19.641	0.648	2.121	8.219e-03
Desulfocarbo	2.982	5.760	1.648	8.756e-03
Sporosarcina	5.918	-3.827	1.470	8.768e-03
Acidobacteria	5.876	-4.594	1.790	8.798e-03
Rhodotorula	54.705	-2.008	1.118	9.105e-03
Cutibacterium	357.153	-0.205	0.573	9.506e-03
Shimia	358.445	-0.568	0.557	9.647e-03
Pseudonocardia	81.981	0.707	0.716	1.059e-02
Levilactobacillus	112.639	-3.726	0.941	1.113e-02
Protomyces	15.298	5.293	1.741	1.176e-02
Blastococcus	150.500	0.530	0.716	1.176e-02
Mogibacterium	9.735	-5.500	1.266	1.176e-02
Agarivorans	92.107	-3.593	1.095	1.198e-02
Marichromatium	2.010	-1.702	1.124	1.237e-02
Georgenia	10.823	3.560	1.171	1.258e-02
Murine_osteosarcoma_virus	2.353	2.618	1.206	1.266e-02
Pyrinomonas	9.505	-5.555	1.655	1.268e-02
Metabacillus	4.195	5.295	1.948	1.270e-02
Dysgonamonadaceae_genus	19.860	-5.791	1.468	1.287e-02
Pestalotiopsis	18.985	-4.929	1.072	1.299e-02
Skermanella	35.925	0.202	1.169	1.325e-02
Arsenophonus	1.327	0.101	1.272	1.333e-02
Epilithonimonas	41.936	0.703	0.670	1.398e-02
Seramator	7.459	-5.126	1.440	1.409e-02
Gilbertella	13763.122	0.897	0.433	1.473e-02
Blautia	26.548	0.655	1.131	1.473e-02
Leptospira	69.642	-2.066	0.527	1.502e-02
Leuconostoc	39.177	-0.254	0.745	1.502e-02
Chitinophaga	7.848	-3.474	1.256	1.517e-02
Caballeronia	39.742	0.867	0.427	1.532e-02
Selenomonas	7.222	-3.937	1.145	1.551e-02
Brachybacterium	121.244	2.496	0.760	1.591e-02
Phytoplasma	62.472	-1.667	0.557	1.667e-02
Endocarpon	10.892	0.311	1.352	1.692e-02
Microsporum	6.726	0.080	1.830	1.729e-02
Rubellimicrobium	30.281	1.249	0.998	1.818e-02
Loigolactobacillus	25.133	-5.697	1.823	1.824e-02
Robertmurraya	10.088	-3.672	1.221	1.874e-02
Trametes	7.826	-3.659	1.354	1.962e-02
Quadrisphaera	26.642	4.438	1.723	1.966e-02
Zymoseptoria	45.638	-0.413	0.759	1.971e-02
Fulvia	6.635	-2.235	1.378	1.978e-02
Williamsia	113.724	-0.340	0.844	1.984e-02
Rhodofomes	8.148	-3.803	1.686	2.030e-02
Veillonella	194.159	-2.203	0.626	2.079e-02
Amaricoccus	20.347	3.457	1.028	2.094e-02
Bacterium	36.502	0.682	0.440	2.114e-02
Suillus	38.317	-0.080	1.769	2.190e-02
Paraconexibacter	6.607	-1.599	2.078	2.204e-02
Picosynechococcus	10.813	0.718	1.300	2.240e-02
Mycena	39.124	4.656	1.508	2.250e-02
Aliidiomarina	6.184	-1.719	1.225	2.329e-02
Paenimyroides	6.925	3.685	1.712	2.442e-02
Caldifermentibacillus	6.086	-5.370	1.983	2.455e-02
Sulfolobus	2.237	1.880	0.864	2.520e-02
Domibacillus	6.862	-5.234	2.159	2.579e-02
Luteolibacter	24.040	-2.381	0.772	2.610e-02
Anoxybacillus	61.693	-2.178	1.357	2.656e-02

Leifsonia	76.614	1.021	0.711	2.707e-02
Neobacillus	54.975	0.675	0.547	2.707e-02
Cercospora	17.883	-2.570	1.117	2.745e-02
Saccharothrix	13.589	2.969	1.020	2.745e-02
Aphanomyces	5.189	-4.389	1.206	2.785e-02
Belnapia	18.847	2.582	1.137	2.791e-02
Leclercia	58.127	0.734	0.514	2.881e-02
Frigoribacterium	39.917	-1.867	1.392	3.081e-02
Chromohalobacter	65.014	-1.015	0.400	3.193e-02
Chelativorans	10.320	-5.587	1.543	3.230e-02
Fibrisoma	18.035	-2.690	1.027	3.274e-02
Aureimonas	24.555	-1.263	0.999	3.300e-02
Photobacterium	11.366	-4.481	1.028	3.543e-02
Alkaliphilus	9.787	-1.139	2.738	3.551e-02
Phenylobacterium	73.211	-0.630	0.733	3.592e-02
Desulfitobacterium	6.033	-2.674	1.541	3.622e-02
Roseateles	324.440	0.674	0.322	3.755e-02
Trichoderma	47.821	-2.342	1.264	3.755e-02
Mycoplasma	2.586	-1.696	1.752	3.809e-02
Marinifilum	38.998	0.939	0.484	3.823e-02
Mus_musculus_mobilized_virus	23.218	1.980	0.612	3.901e-02
Rhodanobacter	6.156	-3.199	1.273	3.933e-02
Pauljensenia	155.649	-2.862	0.829	3.976e-02
Eikenella	13.478	-1.393	1.212	4.000e-02
Pectobacterium	3.690	0.451	1.120	4.000e-02
Wolbachia	68.134	-3.042	0.788	4.057e-02
Paucibacter	116.341	-0.212	0.435	4.068e-02
Segatella	9.943	-3.961	1.203	4.102e-02
Marinilabiliaceae_genus	4.842	-1.812	1.591	4.137e-02
Lactobacillus	101.572	0.210	0.375	4.221e-02
Thermoanaerobacterium	15.500	-5.405	1.820	4.248e-02
Pseudocercospora	12.922	-3.095	1.321	4.248e-02
Frigidibacter	67.900	1.415	0.527	4.333e-02
Reyranella	10.238	-4.023	1.138	4.386e-02
Spleen_focus-forming_virus	3.983	2.472	0.887	4.552e-02
Zychaea	18.213	4.670	2.400	4.569e-02
Arthrobacter	653.862	0.101	0.426	4.577e-02
Moniliophthora	4.949	-0.327	1.780	4.577e-02
Campylobacter	36.232	1.270	0.522	4.582e-02
Batrachochytrium	121.131	0.503	3.053	4.604e-02
Human_adenovirus_2	5.562	-4.157	1.491	4.604e-02
Clavispora	11.624	5.250	2.516	4.618e-02
Gamsiella	11.897	-0.924	1.999	4.641e-02
Chromobacterium	7.322	-1.716	0.963	4.753e-02
Westerdykella	4.874	2.086	1.669	4.862e-02
Babesia	6.351	-4.498	1.301	5.014e-02
Plasmodium	260.330	0.580	0.313	5.015e-02
Niastella	47.268	0.608	2.189	5.091e-02
Pararhodobacter	7.548	1.139	1.541	5.093e-02
Morganella	1.022	0.643	1.250	5.093e-02
Alkalispirochaeta	9160.805	-1.027	0.447	5.157e-02
Apiotrichum	11.988	-3.372	1.454	5.348e-02
Salipiger	55.657	-0.107	0.553	5.602e-02
Phyllobacterium	39.527	1.225	1.001	5.602e-02
Nannochloropsis	2.594	-4.694	2.222	5.606e-02
Penicillium	43.107	-0.184	0.677	5.812e-02
Gloeophyllum	30.331	-20.925	2.039	5.846e-02
Oceanimonas	3.842	2.554	1.403	5.882e-02
Duffyella	6.269	-2.028	1.536	5.979e-02

Thiolapillus	20.111	-2.138	1.463	6.238e-02
Delta	4.266	2.795	1.102	6.251e-02
Trematosphaeria	6.493	3.702	1.823	6.320e-02
Bifidobacteriaceae_genus	10.863	-0.054	1.729	6.329e-02
Citricoccus	39.723	0.844	1.021	6.422e-02
Oribacterium	27.835	-1.741	1.246	6.422e-02
Haemophilus	217.469	-0.029	0.518	6.426e-02
Deinococcus	166.493	0.547	0.290	6.426e-02
Pleionea	5.060	-0.541	1.552	6.447e-02
Aureispira	3.069	-3.028	1.511	6.447e-02
Thermothelomyces	4.242	1.006	2.892	6.572e-02
Methylorubrum	265.529	-0.453	0.423	6.717e-02
Psychrobacter	113.554	0.525	0.558	6.786e-02
Fictibacillus	387.233	-1.534	0.716	6.843e-02
Gallibacter	30.774	-3.828	1.697	6.872e-02
Beggiatoa	32.646	-0.298	0.463	6.991e-02
Shinella	19.684	0.133	1.014	6.991e-02
Kribbella	9.265	0.350	1.169	7.026e-02
Rubrobacter	26.883	-0.048	1.330	7.138e-02
Methylomonas	1.727	2.937	1.137	7.272e-02
Panacagrimonas	4.713	-5.171	2.521	7.384e-02
Sugiyamaella	3.235	4.645	2.218	7.632e-02
Lachnospiraceae_genus	22.274	-0.856	0.854	7.779e-02
Granulicatella	28.531	-0.713	0.867	7.899e-02
Erythrobacter	135.550	1.342	0.430	8.150e-02
Leptolyngbya	41.961	-1.410	0.774	8.150e-02
Bosea	160.841	0.919	0.358	8.204e-02
Mannheimia	5.616	-1.645	1.037	8.204e-02
Nodosilinea	25.845	0.521	1.453	8.873e-02
Pseudenterobacter	0.693	0.425	1.235	9.134e-02
Phanerochaete	8.549	-5.830	1.574	9.141e-02
Burkholderiales	19.138	1.077	0.812	9.309e-02
Pleomorphomonas	12.823	-4.695	2.233	9.329e-02
Podospora	0.954	-2.823	1.516	9.342e-02
Intestinirhabdus	0.996	-1.373	0.926	9.351e-02
Faecalibacterium	6.785	-2.256	1.561	9.368e-02
Emergencia	2.121	4.743	2.489	9.585e-02
Serpula	13.896	-1.363	2.232	9.605e-02
Aedoeadaptatus	14.710	-17.768	2.887	9.764e-02
Algiphilus	4.070	-4.096	1.642	9.801e-02
Aurantimonas	8.757	-1.931	1.076	1.035e-01
Rhizophagus	4.450	1.791	1.887	1.035e-01
Aquitalea	1.946	-2.497	1.520	1.062e-01
Rothia	222.042	-0.174	0.456	1.076e-01
Paraferrimonas	4.903	-1.982	1.281	1.076e-01
Amorphotheca	13.824	-1.412	1.445	1.222e-01
Paludibacterium	3.956	-3.377	1.541	1.248e-01
Mollisia	7.237	-5.855	1.678	1.275e-01
Dichomitus	24.638	2.603	1.266	1.281e-01
Congregibacter	1.218	-0.821	1.892	1.320e-01
Dioszegia	17.319	-6.448	1.841	1.331e-01
Mesorhizobium	79.330	-0.114	0.367	1.357e-01
Diplodia	6.906	-2.277	1.646	1.362e-01
Schizophyllum	63.317	0.768	1.659	1.368e-01
Nocardia	68.412	0.700	0.434	1.471e-01
Aequorivita	205.552	0.690	0.419	1.483e-01
Chitinimonas	19.480	-3.775	1.439	1.487e-01
Planomicrobium	4.483	4.593	2.725	1.501e-01
Kwoniella	10.704	-1.875	1.845	1.521e-01

Turicibacter	6.744	-0.043	2.503	1.523e-01
Oceanospirillum	4.069	-1.722	1.248	1.526e-01
Stutzerimonas	48.535	2.784	0.955	1.528e-01
Coraliihabitans	7.777	-2.460	1.253	1.540e-01
Tetrasphaera	13.715	-1.064	1.317	1.550e-01
Legionella	1332.136	-0.156	0.455	1.554e-01
Segetibacter	3.587	1.854	2.354	1.574e-01
Marinomonas	19.694	-0.245	1.955	1.577e-01
Phialophora	3.186	0.462	1.692	1.577e-01
Caldibacillus	8.783	-2.390	1.744	1.577e-01
Rhizobium	451.252	-0.391	0.308	1.622e-01
Roseibacterium	113.257	-1.539	0.885	1.623e-01
Endosymbiont	5.833	-1.100	1.164	1.729e-01
Polaribacter	8.861	-0.597	0.835	1.753e-01
Thermicanus	19.342	-3.103	1.739	1.757e-01
Bavariicoccus	60.548	-0.454	0.604	1.758e-01
Modestobacter	77.884	-0.782	0.522	1.758e-01
Tissierellia	6.176	-1.426	2.312	1.758e-01
Pelorhabdus	4.813	-1.733	1.384	1.758e-01
Polynucleobacter	4.388	1.548	1.866	1.758e-01
Schaalia	24.329	-3.571	0.935	1.808e-01
Oceanibium	5.401	0.689	1.297	1.813e-01
Acytostelium	1.048	-3.365	1.634	1.815e-01
Pseudoduganella	10.110	0.428	1.522	1.833e-01
Dothidotthia	2.037	0.732	2.213	1.833e-01
Alcanivorax	267.141	0.898	0.441	1.834e-01
Advenella	5.711	5.994	2.006	1.834e-01
Betaproteobacterium_JGI	8.316	0.679	0.801	1.901e-01
Mycobacterium	323.882	-0.621	0.359	1.910e-01
Rhizorhapis	25.863	1.617	0.949	1.942e-01
Sphingosinicella	13.654	1.454	1.637	1.945e-01
Winogradskyella	5.235	0.316	0.730	1.959e-01
Phycomyces	1.988	-1.807	2.169	1.964e-01
Boeremia	20.842	2.635	1.579	1.966e-01
Methylovulum	39.553	1.002	0.908	1.996e-01
Xanthomonadaceae_genus	5.216	-4.621	1.746	2.059e-01
Lentibacillus	34.801	-0.848	0.514	2.063e-01
Bartonella	9.625	-0.917	1.070	2.063e-01
Rhabdonatronobacter	170.476	-1.127	0.469	2.073e-01
Porphyromonas	64.376	-0.138	0.753	2.073e-01
Ramlibacter	69.721	0.576	0.726	2.073e-01
Methylocystis	2.390	-3.450	1.708	2.073e-01
Limnohabitans	19.440	-0.661	0.657	2.088e-01
Acaricomes	2.882	3.317	1.680	2.100e-01
Moraxella	437.707	-0.481	0.479	2.113e-01
Luteibacter	4.401	-3.731	1.634	2.113e-01
FBR_murine_osteosarcoma_virus	1.393	2.230	1.154	2.124e-01
Conchiformibius	9.870	-3.659	1.685	2.134e-01
Devosia	86.884	0.380	0.716	2.169e-01
Salinicoccus	15.586	-0.533	1.216	2.169e-01
Lacticaseibacillus	6.204	-3.051	1.379	2.169e-01
Oceanobacillus	36.670	1.525	0.547	2.181e-01
Spirosoma	14.170	-0.694	1.245	2.186e-01
Tissierella	290.882	0.564	0.364	2.244e-01
Human_endogenous_retrovirus	5.191	1.132	1.041	2.285e-01
Arsukibacterium	2.520	-1.633	2.490	2.285e-01
Porphyromonadaceae_genus	12.235	-7.042	1.315	2.309e-01
Tepidimonas	33.038	1.023	0.914	2.326e-01
Rhodopirellula	20.297	-2.428	0.828	2.326e-01

Rhodoferax	44.739	-0.228	0.616	2.326e-01
Pectinatus	4.713	-2.394	1.415	2.326e-01
Luteimonas	107.126	-0.795	1.100	2.345e-01
Ectobacillus	119.344	0.159	0.684	2.354e-01
Acuticoccus	38.672	1.050	0.483	2.354e-01
Salinicola	3.874	-3.462	1.169	2.354e-01
Salinarimonas	4.201	0.454	0.700	2.354e-01
Schlegelella	25.036	2.328	0.835	2.389e-01
Rummeliibacillus	3.322	-3.540	1.640	2.398e-01
Solihabitans	25.142	0.752	0.605	2.406e-01
Colwellia	7.776	-1.574	1.010	2.409e-01
Aliiruegeria	16.063	-2.500	1.144	2.411e-01
Azohydromonas	22.510	0.337	1.039	2.411e-01
Neohortaea	3.383	-4.843	1.995	2.411e-01
Phaeosphaeria	6.620	5.102	2.841	2.411e-01
Acidiplasma	17.926	-0.060	0.657	2.414e-01
Halomonas	158.342	0.173	0.510	2.426e-01
Tamlana	6.081	0.021	0.885	2.430e-01
Anaeroglobus	4.318	-3.773	1.549	2.466e-01
Microcoleus	16.615	2.000	1.062	2.483e-01
Pararhizobium	14.351	-4.313	1.394	2.550e-01
Gluconacetobacter	6.748	-2.670	1.200	2.550e-01
Marmoricola	34.294	0.655	1.044	2.588e-01
Paenacidovorax	3.180	2.589	1.892	2.588e-01
Methylopila	6.130	-1.907	1.722	2.593e-01
Phycicoccus	66.900	-0.781	0.495	2.659e-01
Parainfluenza_virus_5	10.436	-23.274	2.263	2.659e-01
Nakamurella	30.160	-0.198	1.018	2.672e-01
Weissella	7.985	0.479	1.566	2.672e-01
Didymosphaeria	48.534	0.515	1.260	2.744e-01
Anaerotruncus	1.791	-3.275	1.894	2.781e-01
Algoriphagus	22.246	1.529	0.640	2.796e-01
Mammaliicoccus	12.984	1.203	1.416	2.855e-01
Virgibacillus	35.407	-1.095	0.526	2.881e-01
Ligilactobacillus	18.282	1.291	1.291	2.883e-01
Chromatium	57.450	0.249	0.403	2.932e-01
Variovorax	87.909	-0.758	0.385	2.946e-01
Radiomyces	1.508	5.282	2.437	2.946e-01
Thermomonas	34.874	0.749	0.508	2.946e-01
Thioflexithrix	10.033	-0.705	0.882	2.957e-01
Moraxellaceae_genus	14.760	1.131	1.080	2.977e-01
Pontibacillus	32.118	-20.807	2.560	3.001e-01
Microbacteriaceae_genus	4.368	-5.215	2.359	3.077e-01
Ehrlichia	3.296	-0.948	2.111	3.077e-01
Saccharibacteria	4.408	-2.968	1.973	3.078e-01
Zasmidium	2.768	-1.190	1.755	3.098e-01
Halomicroarcula	1.134	-0.222	2.061	3.098e-01
Motilimonas	7.460	-2.328	1.128	3.137e-01
Terrabacter	109.045	0.786	0.454	3.165e-01
Luteitalea	16.785	1.481	1.927	3.165e-01
Sphaerotilus	3.359	-3.460	1.904	3.184e-01
Thermalbibacter	6.902	-5.443	2.020	3.222e-01
Curvibacter	191.180	0.297	0.285	3.319e-01
Enterobacteria_phage_RTP_virus	2.620	-2.213	1.823	3.319e-01
Jatrophihabitans	13.273	-2.468	2.066	3.327e-01
Abyssicoccus	5.009	2.444	2.161	3.327e-01
Oceanitalea	2.776	3.068	2.903	3.329e-01
Leptotrichia	40.411	-1.088	0.650	3.349e-01
Lactococcus	112.493	-1.830	0.780	3.351e-01

Haematobacter	4.763	3.670	2.286	3.351e-01
Halovibrio	10.136	-2.088	1.227	3.413e-01
Actinokineospora	59.425	-1.201	1.107	3.448e-01
Prevotellaceae_genus	5.849	-0.873	1.320	3.455e-01
Oxalobacteraceae_genus	5.859	-3.218	1.875	3.455e-01
Halochromatium	4.224	3.013	1.258	3.455e-01
Homoserinimonas	5.839	-2.858	1.498	3.460e-01
Pseudoalteromonas	365.269	-0.086	0.397	3.465e-01
Oleiphilus	1.965	4.513	1.521	3.465e-01
Filomicrobium	3.341	-4.568	2.160	3.493e-01
Sparassis	5.100	-1.201	1.459	3.592e-01
Thermomicrobium	3.852	-4.140	1.740	3.592e-01
Trypanosoma	18.124	-0.078	1.141	3.595e-01
Synchytrium	4.292	2.316	2.462	3.598e-01
Kushneria	78.067	-0.088	0.513	3.646e-01
Exiguobacterium	51.512	-0.041	0.663	3.652e-01
Mameliella	2.131	-0.389	2.258	3.703e-01
Xanthobacter	8.431	-0.188	1.366	3.721e-01
Sphingomonadaceae_genus	3.430	5.602	2.747	3.781e-01
Acidothermus	3.350	1.029	2.938	3.821e-01
Gallaecimonas	2.804	4.874	1.962	3.828e-01
Crenobacter	1.823	-0.483	1.904	3.894e-01
Myxococcus	31.942	-0.079	0.520	3.922e-01
Ruegeria	9.018	1.059	1.355	3.922e-01
Rathayibacter	27.104	-3.080	1.250	3.933e-01
Beutenbergia	4.200	2.937	2.140	3.969e-01
Caldimonas	7.771	2.472	1.753	3.985e-01
Proteus_phage_VB_PmiS-Isfahan_virus	1.743	-0.419	1.118	3.994e-01
Mesonia	2.400	-2.361	1.415	4.000e-01
Acidovorax	744.728	0.061	0.211	4.008e-01
Bacidia	8.750	-3.718	1.935	4.008e-01
Halococcus	1.068	-2.258	1.781	4.008e-01
Dactylellina	2.854	-4.354	2.678	4.017e-01
Desulfovibrio	71087.628	-0.705	0.437	4.049e-01
Oceanicola	3.175	2.100	2.074	4.049e-01
Enterobacteria_phage_phi80_virus	4.380	-3.850	1.798	4.076e-01
Limosilactobacillus	14.701	0.229	0.857	4.096e-01
Stomatobaculum	7.662	-4.352	1.627	4.096e-01
Euzebya	1.314	-0.319	2.612	4.184e-01
Aquincola	21.141	1.702	1.055	4.204e-01
Cryptosporidium	15.505	-2.038	0.981	4.207e-01
Salinibacterium	8.132	-1.646	1.258	4.213e-01
Lasiodiplodia	27.457	-0.666	0.687	4.292e-01
Jannaschia	2.987	0.800	0.948	4.297e-01
Halobacteriovorax	2.262	2.252	1.311	4.297e-01
Xanthocytophaga	2.018	0.041	2.447	4.334e-01
Gulosibacter	3.895	-2.505	2.117	4.339e-01
Mycobacteriaceae_genus	41.727	0.707	0.443	4.420e-01
Fibroporia	7.444	-3.185	2.220	4.420e-01
Cokeromyces	2.224	0.471	1.001	4.421e-01
Halorubrum	12.131	-0.978	0.837	4.467e-01
Amnimonas	9.718	3.192	2.142	4.593e-01
Malassezia	1094.910	-0.783	0.520	4.619e-01
Candida	5.852	1.371	1.213	4.619e-01
Gaiella	11.733	-5.809	1.960	4.619e-01
Kluyvera	247.288	0.266	0.366	4.658e-01
Streptomyces	1952.590	-0.206	0.288	4.661e-01
Acidihalobacter	28.945	-1.965	1.043	4.661e-01
Sphaerulina	10.884	-0.018	1.311	4.661e-01

Neomicrococcus	8.418	-0.117	1.469	4.661e-01
Pelagivirga	2.684	1.972	1.243	4.661e-01
Fannyhessea	5.504	-4.840	2.313	4.670e-01
Paenalcaligenes	0.760	3.066	1.867	4.703e-01
Actinomyces	520.914	-0.050	0.468	4.707e-01
Microdochium	4.060	-3.473	1.552	4.722e-01
Geodermatophilus	37.591	1.379	1.080	4.726e-01
Gemmobacter	16.490	0.015	1.305	4.728e-01
Zhihengliuella	3.336	1.127	2.763	4.732e-01
TM7	7.135	-3.806	1.373	4.790e-01
Actinosynnema	3.106	2.489	1.724	4.816e-01
Planctomonas	7.195	-2.026	1.831	4.869e-01
Lentzea	21.126	-0.454	0.598	4.872e-01
Thermosipho	4.351	0.397	0.884	4.872e-01
Thauera	36.207	0.486	0.463	4.873e-01
Xylophilus	6.954	0.732	1.010	4.889e-01
Tabrizicola	4.823	2.171	1.662	4.889e-01
Enhydrobacter	51.501	-0.753	0.554	4.958e-01
Qipengyuania	57.461	-0.712	0.781	4.959e-01
Peredibacter	28.482	-2.134	1.152	4.995e-01
Chrysosporum	14.164	2.128	1.431	5.026e-01
Collimonas	9.925	-0.665	1.416	5.040e-01
Sphingobacterium	129.321	-1.326	0.602	5.043e-01
Glaciibacter	18.801	-2.827	1.287	5.099e-01
Methylobrevis	19.272	1.145	0.811	5.099e-01
Patulibacter	14.300	-1.629	1.609	5.099e-01
Capillimicrobium	2.546	2.356	2.487	5.099e-01
Propionicimonas	3.302	-2.210	2.215	5.099e-01
Thecamonas	2.728	-3.269	2.688	5.099e-01
Tricharina	3.354	-1.712	2.422	5.108e-01
Rickettsia	2.139	1.612	2.768	5.112e-01
Entotheonella	5.670	-2.348	1.449	5.125e-01
Tardiphaga	3.851	1.002	1.497	5.147e-01
Ideonella	12.997	0.140	1.089	5.170e-01
Rhizorhabdus	68.045	0.346	0.575	5.205e-01
Pimelobacter	6.825	-2.338	1.482	5.214e-01
Pseudophaeobacter	5.590	2.430	1.557	5.214e-01
Saezia	2.274	2.150	1.752	5.240e-01
Cyclobacterium	9.163	-0.486	0.730	5.263e-01
Eimeria	1.388	1.424	2.077	5.291e-01
Scytonema	22.749	1.139	0.940	5.410e-01
Solibacillus	6.613	-0.667	1.287	5.422e-01
Methylovorus	3.354	-0.040	1.631	5.493e-01
Buchnera	70221.134	-0.152	0.362	5.494e-01
Hephaestia	3.618	5.554	2.427	5.494e-01
Algibacillus	1.251	1.918	1.804	5.494e-01
Qaidamihabitans	7.592	-0.692	2.238	5.545e-01
Mixta	7.632	-1.011	1.122	5.549e-01
Atlantibacter	14.246	1.015	0.525	5.549e-01
Nitrobacter	5.995	1.777	1.282	5.549e-01
Terriglobus	2.580	-1.463	2.584	5.549e-01
Limimaricola	3.153	0.778	2.677	5.581e-01
Lewinella	0.693	0.868	1.262	5.591e-01
Kytococcus	23.988	-1.001	0.976	5.604e-01
Lelliottia	2.324	-3.146	1.333	5.607e-01
Lactiplantibacillus	3.228	1.086	1.070	5.607e-01
Alkalicoccobacillus	0.836	-2.031	1.588	5.629e-01
Dermabacter	5.429	1.775	1.754	5.708e-01
Parafrankia	2.734	0.416	2.162	5.708e-01

Acidipropionibacterium	23.248	0.181	1.160	5.718e-01
Levyella	10.500	-4.422	2.289	5.718e-01
Tepidicella	11.802	0.752	1.429	5.720e-01
Alkanindiges	7.099	2.314	1.459	5.720e-01
Streptacidiphilus	2.022	2.168	3.129	5.720e-01
Thyridium	8.234	-0.360	2.179	5.722e-01
Buttiauxella	0.847	2.059	1.921	5.722e-01
Labilibaculum	7.087	0.187	0.986	5.726e-01
Alicycliphilus	12.250	-1.406	0.910	5.736e-01
Thalassotalea	1.950	0.440	1.945	5.736e-01
Methanotrophic	6.831	-4.544	1.644	5.743e-01
Aphanizomenon	21.520	0.647	0.514	5.757e-01
Nevskia	7.692	-1.909	2.104	5.782e-01
Pyrenophora	3.967	1.013	1.632	5.813e-01
Chaetomium	2.173	2.782	2.143	5.820e-01
Brettanomyces	5.223	5.921	2.880	5.826e-01
Coprobacillus	2.469	-1.270	2.444	5.826e-01
Methylophilus	8.689	-2.013	1.074	5.860e-01
Dorea	18.063	2.131	1.165	5.887e-01
Clavibacter	6.175	-4.392	1.916	5.888e-01
Paenibacillus	158.355	-0.088	0.360	5.910e-01
Trichophyton	4.702	1.624	2.602	5.912e-01
Drepanopeziza	2.702	-3.389	2.197	5.941e-01
Pasteurella	1.606	-0.681	1.354	5.941e-01
Mangrovicoccus	8.916	1.414	1.861	5.954e-01
Enterobacteria_phage_vB_EcoS_IME542_virus	2.844	-2.878	2.104	5.954e-01
Pedomonas	5.502	0.777	2.103	5.967e-01
Extensimonas	3.609	-3.452	2.063	5.967e-01
Collinsella	12.119	-0.811	1.630	5.970e-01
Burkholderiaceae_genus	42.128	-1.053	0.672	5.970e-01
Azonexus	3.481	0.897	2.124	5.970e-01
Fretibacterium	3.170	-2.624	2.226	5.978e-01
Polymorphobacter	8.366	-2.075	1.719	5.997e-01
Mixia	2.238	2.002	2.356	5.997e-01
Thiobacillus	5.354	0.977	1.522	5.997e-01
Halalkalibacterium	3.247	-3.441	1.797	5.997e-01
Coniosporium	2.486	1.243	2.861	5.997e-01
Atopobium	11.220	-2.034	1.269	6.026e-01
Cryptococcus	7.682	-1.857	1.585	6.031e-01
Talaromyces	8.852	-1.505	1.371	6.056e-01
Motilibacter	2.373	5.039	2.939	6.091e-01
Izhakiella	7.982	1.469	1.554	6.097e-01
Coniophora	16.488	1.091	2.095	6.097e-01
Firmicutes	2.935	0.574	2.448	6.109e-01
Brevibacillus	1.713	-2.150	1.917	6.123e-01
Dechloromonas	8.894	1.358	1.441	6.146e-01
Methylomicrobium	16.552	1.206	1.104	6.146e-01
Kineosporia	6.745	2.347	1.982	6.146e-01
Moorena	7.922	-1.679	1.677	6.209e-01
Paludisphaera	3.132	-3.658	1.737	6.254e-01
Diaporthe	8.192	-0.894	2.179	6.254e-01
Verrucosispora	2.100	-0.157	1.901	6.254e-01
Oceanicella	2.913	-1.647	1.742	6.254e-01
Rhizopus	1.764	-3.861	2.957	6.256e-01
Baudoinia	14.011	-1.409	0.980	6.256e-01
Rosenbergiella	1.423	-1.717	1.431	6.256e-01
Sphingorhabdus	2.961	-2.589	2.443	6.256e-01
Comamonadaceae_genus	99.630	0.419	0.466	6.267e-01
Proteiniclasticum	1.269	-1.983	1.721	6.301e-01

Ruminococcaceae_genus	14.329	1.306	0.737	6.307e-01
Dialister	5.744	-3.477	1.961	6.307e-01
Sphaerobacter	12.579	2.288	2.384	6.307e-01
Pseudorivibacter	2.168	0.151	2.713	6.307e-01
Actinomycetospora	14.912	0.278	0.966	6.333e-01
Pneumocystis	8.386	0.338	2.374	6.333e-01
Kockovaella	3.506	-1.567	2.013	6.333e-01
Rhodovulum	1.619	-2.924	2.725	6.333e-01
Austwickia	3.652	-0.273	2.316	6.336e-01
Phreatobacter	2.827	-2.698	1.843	6.340e-01
Muribaculaceae_genus	0.949	1.667	2.611	6.340e-01
Methylococcus	3.490	2.024	1.433	6.341e-01
Seohaeicola	2.294	2.280	2.616	6.363e-01
Gemmata	2.366	-2.281	3.082	6.391e-01
Gayadomonas	2.348	-1.450	1.106	6.392e-01
Psychromicrobium	11.336	-0.458	0.604	6.405e-01
Eleftheria	9.525	-0.264	1.243	6.531e-01
Tepidiforma	2.948	2.762	3.039	6.532e-01
Exserohilum	4.737	-4.775	2.467	6.557e-01
Simian_virus_40	1.423	-2.083	1.684	6.557e-01
Alloprevotella	42.819	-1.395	0.944	6.571e-01
Azoarcus	4.010	2.239	1.680	6.581e-01
Ezakiella	3.186	1.612	3.441	6.623e-01
Cellulomonas	41.380	0.926	0.663	6.651e-01
Parasphingorhabdus	1.615	1.646	2.439	6.712e-01
Terrihabitans	6.726	3.378	3.434	6.727e-01
Puccinia	4.914	-3.237	1.582	6.727e-01
Alishewanella	16.028	0.027	1.296	6.749e-01
Parvimonas	4.539	-1.993	1.646	6.761e-01
Taibaiella	8.236	0.039	1.468	6.774e-01
Okeania	8.897	-3.053	1.600	6.804e-01
Methylosarcina	17.576	0.774	1.126	6.815e-01
Dysgonomonas	10.780	-1.244	0.875	6.831e-01
Aggregatibacter	18.736	-1.369	0.867	6.844e-01
Methylosinus	1.704	-3.917	2.273	6.844e-01
Hyaloscypha	3.026	-0.827	1.777	6.844e-01
Castellaniella	1.632	-2.418	2.980	6.844e-01
Pinisolibacter	11.572	0.301	0.554	6.856e-01
Streptococcus	1476.802	-0.693	0.580	6.883e-01
Leptomonas	3.765	-20.879	2.172	6.884e-01
Altererythrobacter	7.197	1.156	1.930	6.889e-01
Sulfitobacter	6.435	0.732	0.906	6.900e-01
Fomitiporia	6.233	0.084	1.508	6.902e-01
Emericellopsis	3.316	2.310	2.958	6.907e-01
Mucilaginibacter	56.373	-0.906	0.594	6.943e-01
Hyphomicrobium	34.815	-0.176	0.579	6.943e-01
Escherichia_phage_phiV10_virus	2.464	-3.690	2.031	6.943e-01
Scandinavium	0.642	-0.516	1.311	6.943e-01
Abditibacterium	5.942	-4.221	1.952	6.949e-01
Daldinia	10.587	-3.322	2.199	6.972e-01
Frateuria	2.007	-3.263	1.594	6.972e-01
Parerythrobacter	3.953	-2.629	2.762	7.034e-01
Frondihabitans	1.716	-3.578	2.500	7.036e-01
Rhodomicrobium	7.805	0.744	0.915	7.036e-01
Parasphingopyxis	20.694	0.381	1.335	7.054e-01
Thermococcus	14.111	0.547	0.818	7.054e-01
Gryllotalpicola	3.043	0.325	2.068	7.085e-01
Emticicia	4.218	-4.347	2.819	7.146e-01
Nitrosomonas	5.354	1.515	1.557	7.160e-01

Sphingobium	158.875	-0.031	0.384	7.263e-01
Epithele	1.800	-2.699	2.344	7.272e-01
Chroococcidiopsis	4.006	-3.229	1.936	7.275e-01
Haladaptatus	2.468	-0.605	1.665	7.290e-01
Aurantiacibacter	6.751	-3.110	1.656	7.305e-01
Acanthamoeba	49.409	-0.328	1.340	7.313e-01
Singulisphaera	3.765	-1.716	1.684	7.313e-01
Rhabdothermincola	15.023	0.913	1.851	7.314e-01
Aplosporella	8.752	-0.187	1.518	7.375e-01
Gammaproteobacteria	3.215	-2.918	2.001	7.410e-01
Sporichthya	7.913	0.880	2.206	7.416e-01
Sediminibacterium	6.108	-0.603	2.402	7.416e-01
Rhodospirillum	1.657	1.668	2.706	7.416e-01
Sagittula	4.544	3.936	2.468	7.448e-01
Parageobacillus	3.984	-0.873	2.831	7.529e-01
Natronorubrum	1.146	1.028	3.104	7.529e-01
Desertimonas	18.398	2.020	1.900	7.574e-01
Paenirhodobacter	23.041	-0.485	0.699	7.688e-01
UNVERIFIED_CONTAM:	2.089	-1.982	2.620	7.688e-01
Noviherbaspirillum	23.982	-0.839	1.354	7.703e-01
Arcobacter	3.620	1.675	1.635	7.719e-01
Vallicoccus	2.969	4.772	2.695	7.741e-01
Cellvibrio	9.111	1.142	1.528	7.741e-01
Neurospora	2.010	-3.039	3.766	7.755e-01
Phaeoacremonium	1.292	1.801	1.170	7.758e-01
Thiohalocapsa	35.128	0.524	0.710	7.773e-01
Roseisolibacter	3.503	-0.252	2.212	7.829e-01
Roseicella	5.364	0.015	1.982	7.829e-01
Psychrosphaera	4.364	0.102	1.233	7.829e-01
Gullanella	1.395	3.541	2.061	7.841e-01
Sphingomonas	2004.426	0.262	0.256	7.876e-01
Dacryopinax	1.891	0.799	2.903	7.876e-01
Rhodocyclaceae_genus	4.038	-1.089	1.779	7.876e-01
Kirsten_murine_sarcoma_virus	1.366	4.359	3.302	7.876e-01
Enterobacteria_phage_T4_virus	2.981	-3.448	2.302	7.894e-01
Blochmannia	16.307	0.064	1.409	7.914e-01
Aliterella	15.423	-1.165	2.266	7.914e-01
Aquihabitans	4.924	-1.451	2.139	7.914e-01
Archangium	2.621	1.190	1.587	7.914e-01
Tsukamurella	8.402	4.441	2.522	7.947e-01
Candidate	14.431	-0.275	0.710	7.990e-01
Nitrotoga	4.783	-0.617	1.153	7.990e-01
Siccirubricoccus	2.442	2.354	2.765	8.048e-01
Enterobacteria_phage_vB_EcoS_ACG-M12_virus	1.666	-3.051	2.346	8.052e-01
Penaeicola	1.825	2.076	1.805	8.080e-01
Desarmillaria	3.626	-2.011	1.696	8.103e-01
Kaistia	3.343	-3.546	2.015	8.106e-01
Chryseobacterium	293.101	-0.329	0.409	8.129e-01
Pleomorpha	4.781	1.114	1.890	8.141e-01
Iamia	6.518	-3.259	2.289	8.141e-01
Sandaracinobacteroides	10.046	-1.792	1.673	8.174e-01
Glaesserella	0.891	-1.309	1.856	8.189e-01
Eutypa	3.140	-2.980	1.757	8.227e-01
Leyella	1.037	-2.648	2.705	8.290e-01
Planctomyces	2.081	-2.496	2.164	8.305e-01
Chelatococcus	4.009	-2.775	1.832	8.334e-01
Pyruvatibacter	1.139	0.860	1.522	8.341e-01
Leishmania	4.184	-4.792	2.037	8.359e-01
Metasolibacillus	1.157	-1.964	2.615	8.387e-01

Vulcaniibacterium	1.978	3.578	3.367	8.387e-01
Amycolatopsis	5.663	-0.982	1.038	8.459e-01
Brachymonas	13.279	0.743	0.880	8.469e-01
Hammondia	7.312	-0.264	0.969	8.470e-01
Pelosinus	4.274	-3.508	1.820	8.470e-01
Garicola	5.439	0.171	2.948	8.482e-01
Minimicrobia	2.704	-2.851	2.055	8.499e-01
Hoylesella	15.245	-1.694	1.136	8.503e-01
Tolypothrix	11.518	-2.619	1.773	8.503e-01
Planctopirus	0.764	-3.410	3.101	8.503e-01
Primorskyibacter	0.613	0.445	1.925	8.516e-01
Filobasidium	18.977	-0.194	1.049	8.525e-01
Jeotgalibacillus	1.208	-1.231	2.216	8.549e-01
Gramella	1.113	1.685	1.764	8.560e-01
Piscicoccus	2.025	-0.252	3.037	8.667e-01
Pseudoflavonifractor	1.639	-0.080	2.099	8.691e-01
Paracandidimonas	1.505	5.777	4.069	8.714e-01
Halopseudomonas	2.577	-0.907	1.746	8.727e-01
Aphanothece	13.799	0.405	0.858	8.727e-01
Botrytis	2.262	1.614	2.235	8.727e-01
Rhodoplanes	8.916	-0.534	1.121	8.727e-01
Pseudonocardiaceae_genus	2.564	-0.881	3.494	8.727e-01
Globicatella	2.180	-3.239	3.854	8.727e-01
Angustibacter	5.041	0.128	2.346	8.737e-01
Herbiconiux	8.764	-5.309	2.137	8.748e-01
Thalassolituus	1.194	-0.081	2.258	8.751e-01
Yaniella	2.635	2.584	2.542	8.752e-01
Paecilomyces	5.744	1.159	1.867	8.790e-01
Rhizobiales	18.512	1.250	0.909	8.801e-01
Flavihumibacter	2.570	-2.573	1.735	8.801e-01
Magnetospirillum	1.981	0.818	1.934	8.801e-01
Methylocella	3.834	-0.177	2.525	8.801e-01
Gellertiella	2.769	2.474	3.331	8.801e-01
Jeotgalicoccus	20.422	-0.102	0.744	8.804e-01
Glaciecola	6.760	-0.990	0.740	8.836e-01
Solimonas	5.862	-1.021	1.733	8.836e-01
Putridiphycobacter	2.985	-0.650	1.604	8.886e-01
Pelagibacterium	4.466	0.378	2.242	8.886e-01
Anaeromyxobacter	2.178	1.126	2.686	8.886e-01
Pasteurellaceae_genus	2.823	0.138	1.862	8.886e-01
Arsenicicoccus	2.165	0.402	2.041	8.886e-01
Megasphaera	26.909	2.384	1.435	8.888e-01
Cereibacter	5.029	-0.650	1.587	8.888e-01
Mediterraneibacter	9.994	-0.009	0.856	8.889e-01
Cryptosporangium	3.758	1.222	2.661	8.889e-01
Flexivirga	2.311	1.523	2.242	8.889e-01
Cryptomonas	1.703	0.872	2.687	8.889e-01
Fluoribacter	203.940	0.429	0.542	8.905e-01
Algibacter	5.117	0.347	1.124	8.905e-01
Fimbriimonas	2.579	2.208	2.334	8.908e-01
Filifactor	2.916	4.126	2.083	8.908e-01
Desulfuromonas	1.691	4.475	3.109	8.908e-01
Caldilinea	2.122	-0.022	3.452	8.917e-01
Actinoplanes	35.115	1.461	0.724	8.920e-01
Naasia	3.058	-1.332	2.346	8.924e-01
Oceaniovalibus	4.279	0.946	1.428	9.011e-01
Blastomyces	1.591	-2.061	1.781	9.011e-01
Solobacterium	7.141	-1.941	1.407	9.011e-01
Pseudaminobacter	1.415	3.119	3.391	9.011e-01

Tetragenococcus	9.450	-0.186	1.735	9.029e-01
Gluconobacter	9.302	-2.004	1.291	9.031e-01
Gemmiger	2.088	4.136	3.073	9.031e-01
Yonghaparkia	1.044	-2.395	3.138	9.031e-01
Lacipirellula	1.585	4.864	4.102	9.031e-01
Eremococcus	4.175	-4.128	1.843	9.042e-01
Phaeobacter	1.043	-1.136	1.756	9.046e-01
Citromicrobium	6.385	-0.272	1.680	9.064e-01
Tatumella	7.235	-2.365	1.234	9.076e-01
Alpha	67.476	0.707	0.566	9.076e-01
Brucella	13.265	0.393	0.859	9.138e-01
Gemmatimonas	2.909	-3.048	2.681	9.138e-01
Polaromonas	23.598	-0.451	0.606	9.153e-01
Dermatobacter	1.264	5.213	3.634	9.214e-01
Tsuneonella	1.670	2.953	3.442	9.259e-01
Klenkia	7.551	-1.614	1.498	9.264e-01
Geminicoccus	65.368	-0.321	0.463	9.278e-01
Marinithermofilum	29.006	0.317	0.526	9.279e-01
Carnobacterium	19.889	-0.026	0.969	9.292e-01
Grosmannia	2.498	-3.796	3.119	9.292e-01
Acetobacterium	3.427	-2.965	2.020	9.292e-01
Xenophilus	37.016	0.383	0.651	9.304e-01
Desemzia	16.346	-0.329	1.030	9.321e-01
Peptostreptococcus	6.314	-2.561	1.377	9.321e-01
Parapedobacter	0.896	0.602	2.781	9.321e-01
Herbinix	3.656	-4.836	2.954	9.354e-01
Haliangium	2.382	1.612	3.351	9.369e-01
Riemerella	4.363	-2.034	1.842	9.369e-01
Hallella	4.170	-3.995	1.911	9.369e-01
Psychromonas	1.967	-1.502	1.422	9.369e-01
Aminobacter	1.243	1.438	1.999	9.440e-01
Synechocystis	12.589	0.414	1.252	9.446e-01
Cardiobacterium	13.923	-0.557	1.156	9.446e-01
Heyndrickxia	2.903	-3.767	2.937	9.446e-01
Diolcogaster_facetosa_bracovirus	12.306	-1.837	1.143	9.446e-01
Bhargavaea	9.142	-0.966	1.227	9.446e-01
Rhodospirillaceae_genus	1.528	-0.216	2.787	9.446e-01
Agromyces	13.851	-0.594	0.829	9.454e-01
Bergeriella	2.718	2.238	3.406	9.480e-01
Fluviicola	6.282	-3.921	1.744	9.490e-01
Maribellus	40.625	-1.066	0.698	9.507e-01
Phocaeicola	15.829	-1.233	1.244	9.507e-01
Streptoalloteichus	1.391	0.256	2.820	9.507e-01
Insolitispirillum	2.216	-0.928	2.391	9.507e-01
Paracnuella	0.644	1.015	2.806	9.606e-01
Salinispora	1.822	3.628	2.936	9.639e-01
Rickettsiella	1.563	0.615	1.636	9.639e-01
Rudaea	1.559	-2.178	1.506	9.648e-01
Alteribacter	6.236	-0.058	0.835	9.679e-01
Cellulosimicrobium	4.701	2.717	2.527	9.679e-01
Calothrix	2.801	1.005	1.869	9.711e-01
Phaeovulum	30.793	0.356	0.623	9.711e-01
Ilyonectria	2.369	0.244	2.307	9.711e-01
Pluralibacter	1.241	2.523	2.868	9.745e-01
Meiothermus	30.189	-1.526	1.127	9.762e-01
Loktanella	12.504	-0.474	0.537	9.762e-01
Pyxidicoccus	1.544	-0.878	2.401	9.762e-01
Rhodospirillales	0.991	0.326	3.671	9.762e-01
Linderina	1.155	0.964	2.270	9.762e-01

Tomitella	1.592	-1.551	2.837	9.773e-01
Hoeflea	3.595	0.758	1.948	9.820e-01
Pleurotus	1.535	-0.110	2.248	9.849e-01
Brochothrix	2.027	-0.187	2.806	9.866e-01
Actibacterium	0.985	-3.240	2.823	9.866e-01
Rufibacter	2.548	-2.858	3.730	9.866e-01
Eremomyces	1.147	-4.139	4.484	9.866e-01
Methylibium	9.563	0.211	1.675	9.908e-01
Plantactinospora	1.201	-0.057	2.666	9.917e-01
Ferruginibacter	1.198	-1.726	2.890	9.946e-01
Croceibacterium	1.140	1.156	2.688	9.946e-01
Cyberlindnera	3.470	-16.381	2.687	9.946e-01
Hemiselmis	1.714	1.612	3.491	9.946e-01
Wickerhamomyces	2.767	1.772	2.963	9.946e-01
Alternaria	98.393	0.028	0.457	9.953e-01
Pseudorhizobium	3.035	1.912	2.291	9.953e-01
Collibacillus	3.224	-18.897	4.372	9.962e-01
Romboutsia	3.447	0.770	1.496	9.962e-01
Ralstonia	14254.930	0.229	0.322	9.966e-01
Actinomadura	901.640	-0.007	0.300	9.966e-01
Asticcacaulis	114.652	0.342	0.805	9.966e-01
Dickeya	1.888	-0.483	0.911	9.966e-01
Amnibacterium	9.282	-0.853	1.606	9.966e-01
Abiotrophia	14.388	-0.630	1.243	9.966e-01
Leucothrix	10.440	0.215	0.966	9.966e-01
Fredinandcohnia	6.991	-0.228	1.203	9.966e-01
Chondromyces	2.094	-3.992	3.434	9.966e-01
Agreia	1.710	-0.027	4.227	9.966e-01
Gloeocapsa	4.503	-1.185	2.024	9.966e-01
Falsirhodobacter	4.108	2.039	2.316	9.966e-01
Marinilactibacillus	3.912	-4.158	2.253	9.966e-01
Sandaracinus	3.521	-17.499	3.385	9.966e-01
Pseudoglutamicibacter	1.363	0.772	2.738	9.966e-01
Saccharomyces	1.294	1.295	3.628	9.966e-01
Candidatus	2.000	1.910	3.144	9.966e-01
Sordaria	2.130	-2.963	2.554	9.966e-01
Starkeya	1.085	1.486	2.705	9.966e-01
Mycetocola	2.282	1.548	2.621	9.966e-01
Propionicicella	2.147	1.892	2.614	9.966e-01
Afifella	2.232	3.505	3.754	9.966e-01
Theileria	0.473	-1.772	2.405	9.966e-01
Hubei_permutotetra-like_virus	2.130	0.157	3.949	9.966e-01
Thioalkalivibrio	1.217	-0.247	2.829	9.966e-01
Butyrivibrio	0.998	-2.149	3.644	9.966e-01
Conexibacter	10.106	0.585	1.375	9.974e-01
Ancylomarina	7.570	0.251	1.125	9.974e-01
Gamma	2.276	-0.827	2.274	9.974e-01
Methylocapsa	1.039	-1.468	2.520	9.974e-01
Cupriavidus	310.513	-0.366	0.403	1.000e+00
Xanthomonas	75.563	-0.278	0.590	1.000e+00
Achromobacter	178.806	0.278	0.262	1.000e+00
Lysobacter	182.469	-0.267	0.429	1.000e+00
Falsiroseomonas	137.429	0.108	0.507	1.000e+00
Halalkalibacter	112.475	0.037	0.606	1.000e+00
Allobacillus	31.886	-0.022	0.733	1.000e+00
Sporolactobacillus	110.272	0.025	0.325	1.000e+00
Solirubrobacter	18.530	0.878	1.108	1.000e+00
Methyloversatilis	103.042	-0.015	0.556	1.000e+00
Actinotalea	13.336	0.734	1.407	1.000e+00

Schizosaccharomyces	2.310	1.964	2.297	1.000e+00
Flavisolibacter	0.705	1.030	2.768	1.000e+00
Ethanoligenens	1.222	-0.247	5.527	1.000e+00
Capnocytophaga	41.728	0.195	0.611	1.000e+00
Cnuella	0.966	2.067	4.140	1.000e+00
Weizmannia	1.405	-17.180	5.316	1.000e+00
Aquisphaera	1.528	-1.323	2.658	1.000e+00
Tautonia	1.472	0.890	3.632	1.000e+00
Pandoraea	33.582	0.765	0.632	1.000e+00
Scedosporium	0.752	-1.937	4.132	1.000e+00
Latilactobacillus	2.108	1.468	2.759	1.000e+00
Fenollaria	0.551	-2.092	3.826	1.000e+00
Rhodopseudomonas	31.634	-0.597	0.720	1.000e+00
Parabacteroides	28.004	0.228	0.687	1.000e+00
Tessaracoccus	17.444	1.374	1.291	1.000e+00
Atopomonas	30.225	-0.256	0.570	1.000e+00
Promicromonospora	0.868	1.334	4.107	1.000e+00
Flaviaesturariibacter	0.912	2.172	5.412	1.000e+00
Ogataea	1.139	-1.486	3.132	1.000e+00
Humisphaera	2.628	-1.705	3.017	1.000e+00
Leucobacter	21.363	-0.080	0.816	1.000e+00
Kingella	8.778	-0.744	1.066	1.000e+00
Puia	26.661	-0.634	0.600	1.000e+00
Caulobacteraceae_genus	0.901	0.121	5.003	1.000e+00
Prosthecobacter	3.876	0.744	2.127	1.000e+00
Ectothiorhodospira	16.455	1.149	0.727	1.000e+00
Eubacterium	18.755	-0.158	0.814	1.000e+00
Kaistella	17.043	0.342	0.964	1.000e+00
Arachnia	16.113	-0.729	1.130	1.000e+00
Calidithermus	19.574	-0.303	1.083	1.000e+00
Varibaculum	3.474	-0.146	2.076	1.000e+00
Lachnellula	2.618	-1.109	1.609	1.000e+00
Ilumatobacter	3.893	-1.259	2.228	1.000e+00
Labilithrix	4.216	-1.123	2.171	1.000e+00
Crocosphaera	13.832	1.091	0.798	1.000e+00
Lacrimispora	0.872	2.197	4.166	1.000e+00
Terracoccus	15.641	-0.636	1.607	1.000e+00
Methylotenera	11.956	0.050	1.305	1.000e+00
Treponema	11.715	-0.824	1.068	1.000e+00
UNVERIFIED_ORG:	13.458	1.304	0.971	1.000e+00
Gilliamella	0.563	1.024	2.716	1.000e+00
Ottowia	13.988	-0.213	0.979	1.000e+00
Pediococcus	0.682	-1.055	2.352	1.000e+00
Xylanimonas	2.036	0.464	1.951	1.000e+00
Kinneretia	11.622	0.188	1.028	1.000e+00
Macrococcus	3.332	1.028	1.469	1.000e+00
Mobilicoccus	15.933	0.301	1.142	1.000e+00
Glaciihabitans	0.993	-2.394	3.339	1.000e+00
Pseudokineococcus	6.411	-2.198	2.569	1.000e+00
Agathobacter	7.239	1.488	2.070	1.000e+00
Pseudoclavibacter	10.937	-0.200	1.248	1.000e+00
Winkia	10.745	-0.274	1.596	1.000e+00
Pyrococcus	12.784	0.648	1.162	1.000e+00
Barnesiella	9.605	0.571	0.746	1.000e+00
Actinobaculum	7.638	0.594	1.719	1.000e+00
Baekduia	2.199	-0.405	2.386	1.000e+00
Thermohydrogenium	1.212	-1.645	5.354	1.000e+00
Aquimarina	1.034	-2.607	4.817	1.000e+00
Vagococcus	0.490	1.612	5.799	1.000e+00

Knoellia	9.989	1.260	1.421	1.000e+00
Zimmermannella	7.604	-1.737	1.566	1.000e+00
Psilocybe	1.438	-2.255	1.858	1.000e+00
Nocardiopsis	8.273	0.853	2.000	1.000e+00
Conyzicola	1.343	-2.192	2.629	1.000e+00
Bdellovibrio	0.992	-1.973	2.750	1.000e+00
Miltoncostaea	3.182	0.565	3.075	1.000e+00
Niabella	0.539	-0.399	4.711	1.000e+00
Besnoitia	7.844	0.451	0.867	1.000e+00
Pseudogemmobacter	0.782	-1.001	5.785	1.000e+00
Truepera	5.824	-3.274	2.361	1.000e+00
Micropruina	6.588	2.283	1.995	1.000e+00
Enterovirga	5.894	-1.565	1.981	1.000e+00
Chlorogloea	2.108	2.709	3.075	1.000e+00
Planktothrix	6.905	-0.518	1.274	1.000e+00
Cadophora	1.438	-2.245	2.433	1.000e+00
Actinomarinicola	1.651	-1.146	2.745	1.000e+00
Yimella	7.431	1.815	1.479	1.000e+00
Sporothrix	0.672	-1.109	3.692	1.000e+00
Pseudorhodoferax	8.912	0.694	1.320	1.000e+00
Olsenella	7.411	0.597	1.628	1.000e+00
Coprococcus	2.444	0.696	2.993	1.000e+00
Lacibacter	0.974	0.360	5.794	1.000e+00
Pseudopropionibacterium	6.216	1.063	1.418	1.000e+00
Coleofasciculus	1.102	1.058	2.824	1.000e+00
Renibacterium	7.693	0.605	1.627	1.000e+00
Tolumonas	0.755	-1.318	5.081	1.000e+00
Frankineae	1.069	-1.162	5.359	1.000e+00
Minicystis	1.842	-2.907	3.872	1.000e+00
Hydrogenophilus	2.147	0.168	3.468	1.000e+00
Brenneria	3.075	0.668	0.846	1.000e+00
Sanguibacter	1.792	-1.027	2.776	1.000e+00
Pedococcus	6.046	0.604	1.462	1.000e+00
Pengzhenrongella	1.108	2.195	3.519	1.000e+00
Solemya	6.351	0.751	1.373	1.000e+00
Longimicrobium	6.592	2.649	2.066	1.000e+00
Roseobacter	0.876	2.625	2.933	1.000e+00
Pigmentiphaga	5.865	0.587	1.487	1.000e+00
Chryseomicrobium	0.641	-1.932	3.964	1.000e+00
Barrientosiimonas	8.157	-1.276	2.124	1.000e+00
Anatilimnocola	0.489	4.665	5.780	1.000e+00
Fortiea	3.806	-4.173	2.685	1.000e+00
Microcella	4.645	-1.700	1.757	1.000e+00
Myxococcales	1.400	0.531	4.796	1.000e+00
Paraprevotella	1.819	-0.258	2.568	1.000e+00
Fervidibacillus	2.133	0.175	4.561	1.000e+00
Negativicoccus	0.611	-3.984	5.800	1.000e+00
Chloroploca	0.537	1.612	5.799	1.000e+00
Thalassobius	5.691	1.743	1.494	1.000e+00
Actinobacteria	3.730	-20.887	3.372	1.000e+00
Porphyrobacter	4.254	0.235	1.270	1.000e+00
Pirellula	0.574	0.346	5.137	1.000e+00
Peptostreptococcaceae_genus	4.076	-0.296	1.791	1.000e+00
Galbitalea	2.705	-0.988	2.710	1.000e+00
Naumannella	5.114	-1.998	1.777	1.000e+00
Vogesella	4.571	0.391	1.896	1.000e+00
Plantibacter	4.413	-2.420	2.018	1.000e+00
Cohnella	3.253	0.179	1.447	1.000e+00
Sinorhizobium	5.189	0.148	1.622	1.000e+00

Flectobacillus	1.051	0.076	3.041	1.000e+00
Glaciimonas	3.779	2.883	2.049	1.000e+00
Catenulispora	0.821	1.612	5.799	1.000e+00
Peribacillus	4.798	-0.158	1.893	1.000e+00
Pannonibacter	1.432	1.321	3.211	1.000e+00
Mangrovibacillus	3.321	0.946	2.539	1.000e+00
Labilibacter	4.141	-1.689	1.733	1.000e+00
Azomonas	2.713	-2.167	1.893	1.000e+00
Ancylobacter	5.043	-0.522	1.966	1.000e+00
Acetivibrio	1.359	2.751	4.780	1.000e+00
Saccharibacillus	2.894	-2.236	2.819	1.000e+00
Synechococcus	0.880	-0.873	3.226	1.000e+00
Pisolithus	0.864	0.774	1.907	1.000e+00
Aliarcobacter	1.118	4.346	5.782	1.000e+00
Actinobacterium	1.262	4.961	3.661	1.000e+00
Flavimobilis	2.525	1.976	2.233	1.000e+00
Azorhizobium	2.897	-0.303	1.968	1.000e+00
Fusibacter	1.572	-2.865	3.781	1.000e+00
Megamonas	3.586	0.021	2.183	1.000e+00
Huaxiibacter	3.774	-2.151	1.955	1.000e+00
Nosocomiicoccus	0.953	2.926	3.955	1.000e+00
Roseococcus	1.486	0.967	2.985	1.000e+00
Evansella	0.774	-2.287	5.776	1.000e+00
Geodermatophilaceae_genus	0.845	-2.310	3.827	1.000e+00
Neorhizobium	3.954	0.534	2.067	1.000e+00
Prolinoborus	3.096	0.829	1.716	1.000e+00
Sutterella	3.974	0.386	1.270	1.000e+00
Hyalangium	1.005	4.910	5.219	1.000e+00
Catenibacterium	1.044	-1.626	4.350	1.000e+00
Pseudofrankia	0.563	1.612	5.703	1.000e+00
Hoyosella	1.254	1.612	5.220	1.000e+00
Marisediminicola	0.852	-2.393	5.589	1.000e+00
Ruania	3.365	1.480	2.270	1.000e+00
Mongoliimonas	0.486	0.782	5.796	1.000e+00
Snodgrassella	4.153	1.270	2.529	1.000e+00
Acidisphaera	2.480	-3.195	3.027	1.000e+00
Fimbriiglobus	3.126	1.572	2.603	1.000e+00
Fusicatenibacter	1.772	1.158	3.159	1.000e+00
Paracaedibacter	1.572	1.143	3.729	1.000e+00
Protaetiibacter	2.115	0.952	2.578	1.000e+00
Cryphonectria	1.076	-0.938	2.886	1.000e+00
Fonticella	1.327	-2.313	5.240	1.000e+00
Grimontella	3.770	-0.598	1.507	1.000e+00
Ciceribacter	3.272	1.157	1.646	1.000e+00
Caenimonas	0.820	-1.874	3.015	1.000e+00
Wolinella	0.625	1.612	4.932	1.000e+00
Herpetosiphon	1.350	2.804	3.708	1.000e+00
Flaviflexus	0.626	1.029	4.120	1.000e+00
Stappia	1.579	2.816	2.937	1.000e+00
Asinibacterium	2.381	-1.294	1.988	1.000e+00
Chthoniobacter	2.198	-0.490	3.240	1.000e+00
Catonella	3.258	-0.730	1.987	1.000e+00
Aestuariimicrobium	3.255	0.954	2.056	1.000e+00
Brasilonema	1.716	-1.488	3.255	1.000e+00
Laetiporus	0.632	1.612	5.799	1.000e+00
Mycosynbacter	1.754	-0.569	2.758	1.000e+00
Flavipsychrobacter	0.537	-1.067	5.784	1.000e+00
Aliicoccus	0.581	2.199	5.794	1.000e+00
Herminiimonas	2.008	-1.753	2.845	1.000e+00

Aliihoeflea	3.673	0.177	2.931	1.000e+00
Xinfangfangia	1.197	3.161	3.359	1.000e+00
Lancefieldella	4.026	0.996	2.720	1.000e+00
Acetanaerobacterium	0.693	2.643	4.195	1.000e+00
Paraglaciecola	2.599	1.319	1.328	1.000e+00
Vitreoscilla	1.415	1.612	3.583	1.000e+00
Lactovum	3.125	0.296	2.276	1.000e+00
Pseudomassariella	0.529	-2.652	5.775	1.000e+00
Friedmanniella	3.725	3.315	2.381	1.000e+00
Oscillibacter	0.732	3.395	5.790	1.000e+00
Desulforhabdus	2.687	1.006	2.267	1.000e+00
Alloiococcus	1.164	2.359	2.873	1.000e+00
Saccharomonospora	2.321	1.345	2.034	1.000e+00
Xylella	2.546	-0.610	1.275	1.000e+00
Mobiluncus	3.137	1.342	2.228	1.000e+00
Wielerella	1.083	0.097	4.551	1.000e+00
Perlucidibaca	2.185	2.122	2.085	1.000e+00
Inhella	1.300	-3.187	3.515	1.000e+00
Gemmatirosa	2.401	-0.161	3.430	1.000e+00
Adlercreutzia	0.609	1.612	4.525	1.000e+00
Alsobacter	2.441	1.593	2.596	1.000e+00
Cystobacter	0.999	0.108	4.133	1.000e+00
Nitrolancea	1.083	-2.344	4.029	1.000e+00
Tuber	2.943	-2.512	2.674	1.000e+00
Propionibacteriaceae_genus	1.387	0.620	3.016	1.000e+00
Oxalicibacterium	0.983	1.961	3.246	1.000e+00
Microthrix	0.483	1.612	5.799	1.000e+00
Luteococcus	1.151	1.325	4.530	1.000e+00
Demequina	2.047	2.718	2.267	1.000e+00
Komagataeibacter	1.405	-0.138	3.354	1.000e+00
Marasmius	2.889	1.030	2.158	1.000e+00
Auritidibacter	1.179	-0.925	5.785	1.000e+00
Amniculibacterium	0.863	2.077	5.768	1.000e+00
Jiangella	1.073	-2.043	3.599	1.000e+00
Alistipes	0.973	1.180	3.805	1.000e+00
Thermacetogenium	1.244	-2.189	5.777	1.000e+00
Nitratireductor	1.522	-0.201	2.161	1.000e+00
Richelia	0.517	-2.093	4.151	1.000e+00
Gallintestinimicrobium	0.653	-2.222	5.777	1.000e+00
Drechmeria	0.756	1.297	3.445	1.000e+00
Goekera	0.470	0.006	5.792	1.000e+00
Pectobacterium_phage_CBB_virus	0.902	0.315	3.564	1.000e+00
Bacteriovorax	1.730	-2.436	3.024	1.000e+00
Methanothermobacter	0.537	-1.523	5.781	1.000e+00
Actinophytocola	1.747	-2.594	3.432	1.000e+00
Desulfogranum	1.068	-1.872	3.375	1.000e+00
Neoarthrinium	1.024	-0.823	3.333	1.000e+00
Anabaena	1.596	1.629	2.653	1.000e+00
Gemmataceae_genus	0.636	-0.672	5.104	1.000e+00
Hansschlegelia	2.156	-1.252	2.905	1.000e+00
Peptococcus	0.489	1.612	5.799	1.000e+00
Lichenibacterium	2.044	-1.875	3.084	1.000e+00
Alloalcanivorax	2.425	-0.476	2.061	1.000e+00
Saccharophagus	0.820	-1.507	4.009	1.000e+00
Hydrocarboniphaga	1.904	-1.725	2.584	1.000e+00
Roseitranquillus	1.921	2.483	4.003	1.000e+00
Calidifontibacter	0.577	-1.227	5.781	1.000e+00
Dissoconium	0.726	-3.278	5.773	1.000e+00
Quisquiliibacterium	0.686	1.612	4.915	1.000e+00

Endobacter	3.493	2.523	2.508	1.000e+00
Melaminivora	1.423	0.509	3.223	1.000e+00
Mycoavidus	1.316	3.513	3.797	1.000e+00
Thiomonas	1.233	1.544	2.856	1.000e+00
Millisia	1.115	2.472	4.853	1.000e+00
Faecalicatena	0.820	-0.830	3.443	1.000e+00
Flavobacteriaceae_genus	0.778	-0.711	3.121	1.000e+00
Tychonema	1.638	-2.928	2.436	1.000e+00
Nannocystis	1.346	1.612	4.195	1.000e+00
Coprinopsis	1.019	1.264	3.509	1.000e+00
Sclerotinia	1.417	2.067	2.756	1.000e+00
Verticillium	0.616	-1.073	3.976	1.000e+00
Halobacillus	0.605	-0.702	2.756	1.000e+00
Sneathia	1.805	4.137	4.738	1.000e+00
Cryobacterium	0.921	-0.468	4.997	1.000e+00
Aquabacter	1.821	-2.821	2.565	1.000e+00
Verrucomicrobia	1.670	-2.716	3.485	1.000e+00
Thermincola	0.911	-1.743	5.467	1.000e+00
Purpureocillium	1.627	-2.861	5.761	1.000e+00
Paramagnetospirillum	1.385	-3.074	3.263	1.000e+00
Roseicitreum	1.809	1.655	2.688	1.000e+00
Robbsia	0.852	1.287	2.984	1.000e+00
Arenimonas	1.463	0.258	2.957	1.000e+00
Pilimelia	0.808	3.809	3.346	1.000e+00
Ustilago	1.359	-3.076	2.869	1.000e+00
Schneideria	0.970	0.572	3.414	1.000e+00
Thermogemmata	0.790	1.617	5.608	1.000e+00
Azovibrio	1.084	0.379	3.337	1.000e+00
Minwuia	0.824	0.560	5.795	1.000e+00
Ignavibacterium	0.501	1.612	5.799	1.000e+00
Veillonellaceae_genus	0.824	4.650	5.388	1.000e+00
Intrasporangium	1.177	2.834	4.116	1.000e+00
Desulfotomaculum	0.503	1.612	5.799	1.000e+00
Desertibacillus	1.140	-2.018	3.540	1.000e+00
Propioniferax	0.646	1.612	5.799	1.000e+00
Meyerozyma	1.367	1.995	4.447	1.000e+00
Beijerinckia	0.593	-1.119	5.089	1.000e+00
Myroides	0.746	-0.351	5.729	1.000e+00
Simplicispira	1.925	-0.819	2.019	1.000e+00
Stigmatella	0.634	-2.597	5.775	1.000e+00
Ewingella	0.845	-0.571	5.767	1.000e+00
Klugiella	0.607	-0.350	5.789	1.000e+00
Arcticibacter	0.589	0.218	3.331	1.000e+00
Phototrophicus	0.608	-1.167	5.717	1.000e+00
Pochonia	0.554	-2.358	3.464	1.000e+00
Plastoroseomonas	0.749	0.322	5.045	1.000e+00
Branchiibius	0.578	0.256	5.770	1.000e+00
Parasutterella	0.525	-1.919	3.021	1.000e+00
Truncatella	1.191	-2.744	3.207	1.000e+00
Aaosphaeria	0.697	-0.463	5.490	1.000e+00
Bryobacter	0.568	1.612	5.799	1.000e+00
Puniceibacterium	0.603	-1.930	4.298	1.000e+00
Oscillatoria	1.378	-1.241	2.613	1.000e+00
Immundisolibacter	1.554	-0.703	2.628	1.000e+00
Rhodoligotrophos	1.096	0.796	3.876	1.000e+00
Bacteroidetes	1.272	1.674	4.403	1.000e+00
Crenalkalicoccus	0.619	1.645	5.011	1.000e+00
Andreesenia	0.839	1.373	5.798	1.000e+00
Catellicoccus	1.455	1.612	4.287	1.000e+00

Microvirgula	1.737	-0.271	3.758	1.000e+00
Rhodoblastus	0.592	1.612	4.141	1.000e+00
Peptoclostridium	0.768	1.133	4.568	1.000e+00
Aestuariibaculum	1.183	-1.123	2.489	1.000e+00
Maridesulfovibrio	0.477	4.584	5.779	1.000e+00
Oerskovia	0.479	1.884	5.798	1.000e+00
Phormidium	0.945	-0.800	3.550	1.000e+00
Helcococcus	0.691	1.612	5.799	1.000e+00
Glycomyces	1.173	-2.187	4.099	1.000e+00
Acidiphilium	0.973	0.813	3.353	1.000e+00
Steroidobacter	1.439	1.782	2.884	1.000e+00
Hankyongella	1.344	-0.407	5.266	1.000e+00
Oligoflexus	0.666	1.612	5.799	1.000e+00
Nonomuraea	1.439	-1.233	2.505	1.000e+00
Allosphingosinicella	0.640	2.482	3.794	1.000e+00
Nanoperiomorbus	1.569	-1.014	3.258	1.000e+00
Pseudolabrys	0.699	0.679	3.688	1.000e+00
Erythrobacteraceae_genus	1.717	2.614	2.703	1.000e+00
Phytohabitans	0.770	1.324	5.798	1.000e+00
Parvularcula	2.074	1.344	3.156	1.000e+00
Halophilic	1.183	-2.607	3.613	1.000e+00
Alkalibacterium	1.571	4.462	3.347	1.000e+00
Salifodinibacter	1.878	-2.592	3.676	1.000e+00
Catellatospora	0.854	1.612	5.799	1.000e+00
Stakelama	0.615	3.646	3.750	1.000e+00
Nitrosocosmicus	2.028	1.612	4.329	1.000e+00
Ureibacillus	0.798	-1.060	3.498	1.000e+00
Fastidiosipila	0.578	1.612	5.799	1.000e+00
Carideicomes	0.567	-0.209	2.782	1.000e+00
Aliidongia	0.963	0.479	3.574	1.000e+00
Caldovatus	0.480	0.826	4.200	1.000e+00
Butyricicoccus	0.747	1.144	4.002	1.000e+00
Granulicella	0.826	-3.001	5.533	1.000e+00
Gregarina	0.881	1.324	5.798	1.000e+00
Dokdonella	0.875	4.538	4.415	1.000e+00
Kickxella	0.850	-2.459	4.230	1.000e+00
Aromatoleum	0.470	1.646	5.798	1.000e+00
Lampropedia	1.054	3.312	3.679	1.000e+00
Niveispirillum	1.317	0.550	3.489	1.000e+00
Rivularia	0.796	-1.376	4.511	1.000e+00
Nanosynsacchari	0.896	-1.091	3.276	1.000e+00
Nitrososphaera	1.353	5.072	5.778	1.000e+00
Actinoallomurus	0.960	3.080	3.902	1.000e+00
Ramularia	0.493	-2.603	5.775	1.000e+00
Rehaibacterium	0.635	0.635	5.591	1.000e+00
Auraticoccus	1.430	4.273	5.744	1.000e+00
Halteromyces	0.521	3.692	3.906	1.000e+00
Ustilaginoidea	1.194	3.938	3.719	1.000e+00
Nocardioidaceae_genus	0.671	1.612	5.799	1.000e+00
Cordyceps	0.785	1.839	4.286	1.000e+00
Micavibrio	0.622	-2.699	5.027	1.000e+00
Methyloglobulus	0.746	-0.144	3.053	1.000e+00
Emiliania	0.907	-0.419	2.635	1.000e+00
Oligella	1.158	-0.352	5.390	1.000e+00
Lachnoclostridium	1.274	1.612	4.637	1.000e+00
Limobrevibacterium	0.674	-3.112	3.692	1.000e+00
Embleya	1.484	4.622	3.360	1.000e+00
Idiomarina	0.677	-1.008	5.785	1.000e+00
Paeniroseomonas	0.513	-1.120	4.253	1.000e+00

Paraphaeosphaeria	1.127	-1.310	5.367	1.000e+00
Subtercola	1.074	-1.631	4.060	1.000e+00
Methylophaga	1.029	1.003	2.580	1.000e+00
Paracraurococcus	0.696	-0.925	4.240	1.000e+00
Hyphomonas	0.992	1.842	3.933	1.000e+00
Faecalimonas	1.189	-0.636	3.521	1.000e+00
Actinoalloteichus	0.689	-0.396	2.979	1.000e+00
Pseudorhodoplanes	0.900	-2.173	2.744	1.000e+00
Mumia	1.011	1.884	4.396	1.000e+00
Laspinema	0.879	-0.413	4.602	1.000e+00
Aeribacillus	0.675	1.612	5.799	1.000e+00
Aquimonas	1.574	0.159	4.835	1.000e+00
Isoalcanivorax	1.062	-0.079	4.306	1.000e+00
Aquibium	0.920	-0.900	4.085	1.000e+00
Dankookia	0.909	-0.868	3.299	1.000e+00
Acidisoma	1.128	0.586	3.690	1.000e+00
Xanthomarina	0.828	-2.474	3.539	1.000e+00
Allocoleopsis	0.836	1.612	4.166	1.000e+00
Parvibaculum	1.055	3.630	3.004	1.000e+00
Microterricola	0.789	-3.434	4.791	1.000e+00
Anaerosphaera	0.551	3.497	5.775	1.000e+00
Saliphagus	1.041	-2.645	2.523	1.000e+00
Labedella	0.902	-2.214	4.461	1.000e+00
Frischella	1.199	0.186	1.969	1.000e+00
Pichia	1.167	-2.937	2.874	1.000e+00
Wigglesworthia	1.349	1.052	1.810	1.000e+00
Hypoxylon	1.453	0.172	3.211	1.000e+00
Chiayiivirga	1.205	-2.135	4.691	1.000e+00
Vibrionimonas	0.863	-2.473	4.378	1.000e+00
Krasilnikoviella	0.643	4.974	5.779	1.000e+00
Gulbenkiania	0.803	-2.290	4.371	1.000e+00
Scardovia	0.474	-2.595	5.775	1.000e+00
Acidocella	1.112	-2.456	3.149	1.000e+00
Chloroflexi	0.505	3.106	3.468	1.000e+00
Faunimonas	1.650	-1.277	5.611	1.000e+00
Zobellella	1.165	0.679	4.043	1.000e+00
Mycoplasmopsis	1.529	-3.156	2.402	1.000e+00
Liquorilactobacillus	0.867	3.825	4.009	1.000e+00
Fuscibacter	0.854	0.358	5.351	1.000e+00
Intrasporangiaceae_genus	0.940	-0.847	2.820	1.000e+00
Sandarakinorhabdus	1.376	-0.242	4.394	1.000e+00
Urbifossiella	0.741	-3.175	5.435	1.000e+00
Lignipirellula	0.509	0.149	5.770	1.000e+00
Slackia	0.631	0.573	4.740	1.000e+00
Aquariibacter	0.913	-0.940	2.907	1.000e+00
Haematomicrobium	1.756	1.668	4.730	1.000e+00
Gallionella	0.693	-1.870	2.154	1.000e+00
Puteibacter	1.005	2.979	2.504	1.000e+00
Mesobacillus	0.966	-0.662	3.342	1.000e+00
Calidifontimicrobium	0.522	4.166	5.783	1.000e+00
Thiofilum	1.021	-1.510	5.045	1.000e+00
Oryzibacter	0.481	0.454	5.768	1.000e+00
Segnochrobactrum	1.097	4.643	4.807	1.000e+00
Debaryomyces	0.735	-1.090	4.997	1.000e+00
Spirilliplanes	0.975	5.707	5.776	1.000e+00
Malikia	0.578	-1.985	5.176	1.000e+00
Nigerium	0.539	1.612	5.799	1.000e+00
Pleurocapsa	1.217	1.216	3.827	1.000e+00
Histoplasma	0.752	-0.021	2.723	1.000e+00

Pelobacter	1.250	4.440	3.700	1.000e+00
Escherichia_phage_vB_EcoS_ESCO41_virus	1.030	-2.697	3.136	1.000e+00
Dysosmobacter	0.526	-2.421	5.776	1.000e+00
Morchella	0.641	1.612	5.799	1.000e+00
Rugosimonospora	1.487	1.612	5.799	1.000e+00
Sporomusaceae_genus	0.491	1.612	2.945	1.000e+00
Mitsuokella	0.467	0.782	5.796	1.000e+00
Polysphondylium	0.471	1.420	5.798	1.000e+00
Scleromatobacter	0.808	3.687	5.777	1.000e+00
Cucurbitaria	0.669	0.117	3.705	1.000e+00
Occultella	0.470	4.699	5.780	1.000e+00
Nitrospira	0.933	-0.461	4.766	1.000e+00
Ornithinicoccus	1.709	-1.392	5.502	1.000e+00
Halovulum	1.008	4.312	3.542	1.000e+00
Microbacter	0.938	1.818	3.897	1.000e+00
Croceicoccus	0.549	1.326	3.470	1.000e+00
Frigoriflavimonas	0.492	1.612	5.249	1.000e+00
Hydrobacter	0.593	2.818	5.041	1.000e+00
Thermoleophilum	0.477	-0.478	5.788	1.000e+00
Larkinella	0.768	-0.691	3.707	1.000e+00
Saccharimonas	0.553	-2.157	5.157	1.000e+00
Sinomonas	1.516	1.191	5.798	1.000e+00
Escherichia_phage_DTL_virus	0.756	-2.577	4.597	1.000e+00
Coccidioides	0.857	0.062	2.660	1.000e+00
Simonsiella	0.622	0.081	5.640	1.000e+00
Jeotgalibaca	1.008	0.738	4.496	1.000e+00
BeAn_58058_virus	0.620	2.309	2.460	1.000e+00
Aestuariivirga	0.693	-2.493	5.522	1.000e+00
Nannizzia	1.520	0.904	2.759	1.000e+00
Haliea	0.489	0.872	3.246	1.000e+00
Siphonobacter	0.858	0.490	5.795	1.000e+00
Shouchella	0.581	1.612	5.799	1.000e+00
Ktedonobacter	0.818	5.087	5.778	1.000e+00
Anaerobiospirillum	0.687	-2.829	5.774	1.000e+00
Roseibium	0.641	-2.513	3.780	1.000e+00
Paenarthrobacter	0.590	0.546	5.745	1.000e+00
Betaproteobacterium_AAP65	0.934	-1.708	2.432	1.000e+00
Rhodocyclus	0.572	-1.477	3.482	1.000e+00
Mesomycoplasma	0.933	-3.444	4.794	1.000e+00
Elstera	0.783	4.352	5.553	1.000e+00
Haloferax	0.578	0.203	5.673	1.000e+00
Brooklawnia	1.148	2.573	4.968	1.000e+00
Neptunicoccus	0.662	5.168	5.778	1.000e+00
Limnobaculum	0.794	2.070	2.440	1.000e+00
Argonema	0.523	3.666	5.788	1.000e+00
Weeksella	0.627	1.612	5.799	1.000e+00
Camelimonas	0.500	-1.554	5.781	1.000e+00
Inquilinus	0.544	0.033	3.468	1.000e+00
Filamentous	0.917	-2.119	4.077	1.000e+00
Ferrovum	0.818	-1.988	5.601	1.000e+00
Propionibacterium_phage_PHL116M00_virus	0.473	-0.554	5.788	1.000e+00
Paenisporosarcina	1.633	-5.561	5.800	1.000e+00
Salegentibacter	0.779	-0.316	5.789	1.000e+00
Penicilliopsis	0.668	-2.108	5.776	1.000e+00
Propionibacterium_phage_PAD20_virus	0.688	4.699	5.780	1.000e+00
Helicobacter	0.471	-2.222	5.777	1.000e+00
Verticiella	1.122	1.612	5.126	1.000e+00
Dongia	0.909	1.325	3.895	1.000e+00
Tersicoccus	1.047	5.033	5.774	1.000e+00

Holdemanella	0.771	4.840	4.487	1.000e+00
Betaproteobacterium_AAP121	0.906	-2.398	2.360	1.000e+00
Jaminaea	0.480	-3.018	5.774	1.000e+00
Enterocloster	0.679	3.154	4.427	1.000e+00
Wenjunlia	0.648	1.612	5.581	1.000e+00
Rhodocyclales	0.940	5.040	5.179	1.000e+00
Phialemonium	0.746	-1.767	5.137	1.000e+00
Thermaerobacter	0.642	1.612	5.718	1.000e+00
Phaseolus_vulgaris_endornavirus	1.016	5.836	5.776	1.000e+00
Defluviimonas	0.700	-2.623	3.506	1.000e+00
Type-C_symbiont_of_Plautia_stali	0.212	1.612	5.799	NA
Sodalis-like	0.331	1.330	2.281	NA
Bacteria	0.118	0.796	2.281	NA
Type-D_symbiont_of_Plautia_stali	0.011	1.324	5.798	NA
Type-F_symbiont_of_Plautia_stali	0.110	1.421	5.798	NA
Endozoicomonas	0.368	3.290	2.978	NA
Profftia	0.070	1.612	5.799	NA
Plautia	0.323	0.689	3.793	NA
Type-E_symbiont_of_Plautia_stali	0.036	2.150	5.798	NA
Annandia	0.084	0.922	5.797	NA
Trabulsiella	0.462	-0.064	1.254	NA
Pseudactinotalea	0.117	1.612	5.799	NA
Mangrovibacter	0.437	-0.052	1.531	NA
Thiothrix	0.405	0.380	5.770	NA
Mikella	0.000	0.000	0.000	NA
Ishikawaella	0.000	0.000	0.000	NA
Hafniaceae_genus	0.000	0.000	0.000	NA
Bathymodiolus	0.021	0.825	5.796	NA
Hoaglandella	0.398	1.612	5.799	NA
Enteractinococcus	0.398	0.782	5.796	NA
Superficieibacter	0.163	0.277	2.671	NA
Alicyclobacillus	0.391	1.368	5.795	NA
Shigella_phage_SfIV_virus	0.000	0.000	0.000	NA
Moranella	0.333	1.324	5.798	NA
Zygosaccharomyces	0.127	2.172	5.798	NA
Kuraishia	0.224	-1.531	5.781	NA
Chthonobacter	0.205	-1.214	5.783	NA
Mediannikoviicoccus	0.000	0.000	0.000	NA
Isosphaera	0.330	0.782	5.796	NA
Tachikawaea	0.009	1.612	5.799	NA
Aggregicoccus	0.236	3.866	5.786	NA
Oscillochloris	0.293	1.715	5.776	NA
Faecalibacillus	0.089	1.612	5.799	NA
Escherichia_phage_500465-1_virus	0.000	0.000	0.000	NA
Niallia	0.268	1.373	5.570	NA
Umbelopsis	0.027	1.022	5.797	NA
Enterobacteria_phage_DE3_virus	0.000	0.000	0.000	NA
Viridilinea	0.240	3.686	5.788	NA
Terribacillus	0.063	0.674	5.796	NA
Arthroderma	0.451	2.151	2.864	NA
Tenebrionibacter	0.227	0.161	2.154	NA
Zafaria	0.027	1.612	5.799	NA
Winslowiella	0.287	1.612	5.799	NA
Oryzihumus	0.119	-1.084	5.784	NA
Cobetia	0.433	-0.982	3.319	NA
Thermobacillus	0.214	1.022	5.797	NA
Chitinophagaceae_genus	0.335	1.420	5.798	NA
Berkiella	0.057	1.066	5.797	NA
Rubrivirga	0.461	1.612	5.799	NA

Yoonia	0.283	2.172	5.798	NA
Fervidobacterium	0.190	1.612	5.799	NA
Enterobacteria_phage_P7_virus	0.000	0.000	0.000	NA
Moorella	0.340	1.612	5.799	NA
Acidiferrimicrobium	0.300	0.812	4.769	NA
Escherichia_phage_RCS47_virus	0.000	0.000	0.000	NA
Armatimonas	0.268	0.718	5.796	NA
Ilyomonas	0.448	-3.009	5.774	NA
Thermodesulfomicrobium	0.020	1.612	5.799	NA
Planifilum	0.365	1.612	5.799	NA
SsRNA_phage_SRR5466369_2_virus	0.058	1.612	5.799	NA
Aciditerrimonas	0.325	0.213	4.781	NA
Carbonactinospora	0.255	-0.405	5.789	NA
Lonsdalea	0.189	1.036	2.193	NA
Parasegetibacter	0.335	1.775	5.799	NA
Alterileibacterium	0.156	1.612	5.799	NA
Lagierella	0.000	0.000	0.000	NA
Sorangium	0.422	-2.460	5.776	NA
Uruburuella	0.038	1.612	5.799	NA
Acidiluteibacter	0.200	1.324	5.798	NA
Neofamilia	0.005	1.612	5.799	NA
Franconibacter	0.453	1.029	2.908	NA
Riesia	0.026	1.612	5.799	NA
Hirsutella	0.112	1.612	5.799	NA
Companilactobacillus	0.288	1.612	5.799	NA
Pseudosporangium	0.338	3.922	5.786	NA
Cereal_yellow_dwarf_virus	0.223	-1.783	5.779	NA
Tenebrionicola	0.031	1.612	5.799	NA
Rhizobiaceae_genus	0.018	0.782	5.796	NA
Rhabdobacter	0.000	0.000	0.000	NA
Kallipyga	0.000	0.000	0.000	NA
Phascolarctobacterium	0.268	-0.224	5.790	NA
Pajaroellobacter	0.393	1.612	5.799	NA
Escherichia_phage_500465-2_virus	0.000	0.000	0.000	NA
Escherichia_virus_Lambda_2G7b	0.000	0.000	0.000	NA
Type-B_symbiont_of_Plautia_stali	0.070	1.374	5.798	NA
Doolittlea	0.005	1.324	5.798	NA
Zymomonas	0.330	1.612	5.799	NA
Photodesmus	0.007	1.324	5.798	NA
Parachlamydia	0.456	-0.644	5.787	NA
Dubosiella	0.074	0.220	5.794	NA
Dactylosporangium	0.221	-1.854	5.779	NA
Pseudohongiella	0.062	1.612	5.799	NA
Agaricicola	0.028	1.884	5.798	NA
Pedosphaera	0.186	1.612	5.799	NA
Erysipelothrix	0.043	1.612	5.799	NA
Flintibacter	0.137	1.612	5.799	NA
Zavarzinella	0.260	1.612	5.799	NA
Pinibacter	0.256	1.612	5.799	NA
Escherichia_phage_TL-2011b_virus	0.000	0.000	0.000	NA
Enterobacteria_phage_f1_virus	0.008	1.324	5.798	NA
Pseudobdellovibrio	0.385	-1.939	5.259	NA
Sandaracinobacter	0.244	0.241	5.794	NA
Hassallia	0.102	1.612	5.799	NA
Siccibacter	0.129	0.881	5.796	NA
Defluviicoccus	0.374	-2.054	5.778	NA
Arthromitus	0.148	1.612	5.799	NA
Thermoanaerobacter	0.395	1.612	5.799	NA
Qingrenia	0.304	1.612	5.799	NA

Sphingosinithalassobacter	0.325	1.325	4.308	NA
Escherichia_virus_Lambda_4A7	0.000	0.000	0.000	NA
Macromonas	0.075	1.612	5.799	NA
Micrococcaceae_genus	0.386	-1.284	5.143	NA
Sinisalibacter	0.394	-0.601	5.400	NA
Centipeda	0.032	0.813	5.796	NA
Paraflavisolibacter	0.061	1.421	5.798	NA
Prauserella	0.035	1.612	5.799	NA
Anaerotardibacter	0.125	1.421	5.798	NA
Planctomycetes	0.410	-0.182	5.343	NA
Mariluticola	0.153	1.612	5.799	NA
Pusillibacter	0.000	0.000	0.000	NA
Rectinema	0.000	0.000	0.000	NA
Anaerobutyricum	0.398	1.612	5.799	NA
Propylenella	0.014	0.782	5.796	NA
Geoalkalibacter	0.121	1.612	5.799	NA
Lawsonibacter	0.311	-0.653	5.785	NA
Plectonema	0.287	-1.436	5.781	NA
Escherichia_phage_Lambda_ev099_virus	0.000	0.000	0.000	NA
Klebsiella_phage_4_virus	0.000	0.000	0.000	NA
Wenxinia	0.272	0.444	5.452	NA
Planobispora	0.083	0.457	5.795	NA
Mycoplana	0.174	1.612	5.799	NA
Kordiimonas	0.361	1.324	5.798	NA
Neptuniibacter	0.092	1.612	5.799	NA
Stx2-converting_phage_1717_virus	0.000	0.000	0.000	NA
Planomonospora	0.303	1.884	5.798	NA
Fischerella	0.349	0.767	5.796	NA
Anaeromassilibacillus	0.075	1.324	5.798	NA
Cetobacterium	0.000	0.000	0.000	NA
Parachlamydiaceae_genus	0.346	-1.639	5.780	NA
Actirhodobacter	0.195	0.741	5.794	NA
Escherichia_phage_Cartapus_virus	0.000	0.000	0.000	NA
Rhodocista	0.302	2.067	4.242	NA
Coprothermobacter	0.251	-2.158	5.777	NA
Schumannella	0.440	2.486	5.797	NA
Coriobacteriaceae_genus	0.340	1.612	5.799	NA
Baumannia	0.277	1.612	5.799	NA
Cecembia	0.302	1.373	5.798	NA
Xylaria	0.460	1.325	4.551	NA
Polymorphum	0.458	-0.270	4.396	NA
Reticulibacter	0.388	1.612	5.799	NA
Bacteroidales	0.451	0.232	4.625	NA
Thermorudis	0.237	1.324	5.798	NA
Granulicoccus	0.329	1.668	5.779	NA
Dinghuibacter	0.264	1.612	5.799	NA
SsRNA_phage_SRR5466337_3_virus	0.000	0.000	0.000	NA
Zeimonas	0.426	-1.774	5.778	NA
Lamprobacter	0.388	0.590	3.024	NA
Escherichia_phage_Lambda_ev207_virus	0.000	0.000	0.000	NA
Escherichia_virus_Lambda_1H12	0.000	0.000	0.000	NA
Solirhodobacter	0.058	1.612	5.799	NA
Tropicibacter	0.000	0.000	0.000	NA
Proteiniphilum	0.024	1.612	5.799	NA
Variibacter	0.081	1.022	5.797	NA
Enterobacteria_phage_T7_virus	0.002	1.421	5.798	NA
Pseudocnuella	0.005	1.612	5.799	NA
Anaerobacillus	0.305	-1.386	5.782	NA
Formosimonas	0.082	2.172	5.798	NA

Dongshaea	0.131	0.305	5.794	NA
Desulfofundulus	0.368	3.979	5.785	NA
Thermosinus	0.439	-1.152	5.784	NA
Youxingia	0.215	1.421	5.798	NA
Stx2-converting_phage_Stx2a_WGPS2_virus	0.000	0.000	0.000	NA
Flavonifractor	0.231	-1.819	5.779	NA
Ruoffia	0.404	-0.414	5.396	NA
Paraflavitalea	0.310	-1.203	5.783	NA
Durotheca	0.146	1.612	5.799	NA
Holdemania	0.350	1.612	5.799	NA
Moheibacter	0.373	-0.350	5.789	NA
Sabulicella	0.126	1.612	5.799	NA
Agathobaculum	0.083	1.612	5.799	NA
Escherichia_phage_Lambda_ev243_virus	0.000	0.000	0.000	NA
Petrimonas	0.193	2.820	5.795	NA
Geomicrobium	0.127	0.186	5.786	NA
Coriobacteriales	0.346	-2.221	5.777	NA
Neoactinobaculum	0.366	-1.702	5.780	NA
Falsochrobactrum	0.395	1.373	5.798	NA
Couchioplanes	0.000	0.000	0.000	NA
Yeguia	0.064	1.612	5.799	NA
Variimorphobacter	0.190	1.612	5.799	NA
Citreicoccus	0.220	1.016	5.797	NA
Serpentinimonas	0.382	1.373	5.798	NA
Ferribacterium	0.267	1.324	5.798	NA
Miniimonas	0.445	-0.139	5.791	NA
Pelagerythrobacter	0.364	1.612	5.799	NA
Erysipelotrichaceae_genus	0.333	-2.320	5.776	NA
Chlamydia	0.204	1.324	5.798	NA
Aridibaculum	0.190	0.635	5.795	NA
Allostreptomyces	0.331	1.612	5.799	NA
Lentihominibacter	0.331	3.028	5.790	NA
Escherichia_virus_Lambda_2H10	0.000	0.000	0.000	NA
Lederbergia	0.161	0.301	5.794	NA
Methanothrix	0.009	1.612	5.799	NA
Catenuloplanes	0.057	1.612	5.799	NA
Rouxiella	0.459	-1.934	5.056	NA
Protochlamydia	0.417	-0.350	5.789	NA
Tuwongella	0.369	1.612	5.799	NA
Hydromonas	0.010	1.612	5.799	NA
Soleaferrea	0.148	1.612	5.799	NA
Thermobrachium	0.226	1.612	5.799	NA
Amygdalobacter	0.251	-1.488	5.781	NA
Rodentibacter	0.334	1.612	4.864	NA
Sphingomonas-like	0.439	-0.803	2.873	NA
Gynurincola	0.054	1.612	5.799	NA
Lipomyces	0.316	-1.639	5.779	NA
Mariprofundus	0.313	1.612	5.799	NA
Salicibibacter	0.239	-0.234	5.790	NA
Rhodovibrio	0.439	-1.531	5.781	NA
Acidobacteriaceae_genus	0.311	0.347	5.794	NA
Nanogingivalis	0.227	1.018	4.891	NA
Acidimicrobium	0.273	1.612	5.799	NA
Runella	0.324	0.187	5.793	NA
Lindgomyces	0.223	-0.619	5.787	NA
Fodinicola	0.339	1.612	5.799	NA
Fontibacillus	0.077	-0.600	5.787	NA
Enterobacteria_phage_YYZ-2008_virus	0.009	1.612	5.799	NA
Elioraea	0.452	1.884	5.239	NA

Quatrionicoccus	0.245	1.324	5.798	NA
Virgisporangium	0.184	-0.327	5.789	NA
Capsulimonas	0.344	-0.264	5.452	NA
Nisaea	0.072	1.612	5.799	NA
Pseudovibrio	0.453	2.069	5.786	NA
Deferrisoma	0.000	0.000	0.000	NA
Papillibacter	0.041	0.029	5.793	NA
Rickettsiales	0.188	1.612	5.799	NA
Neoroseomonas	0.161	1.324	5.798	NA
Tahibacter	0.454	-1.866	3.922	NA
Propionibacterium_phage_PHL041M10_virus	0.037	0.301	5.794	NA
Aff.	0.263	1.612	5.799	NA
Anaerostipes	0.192	1.163	4.988	NA
Chloroflexia	0.066	1.612	5.799	NA
Propionibacterium_phage_PHL301M00_virus	0.225	1.612	5.799	NA
Trujillella	0.198	1.884	5.798	NA
Atlanticothrix	0.147	1.324	5.798	NA
Hominisplanchenecus	0.033	1.612	5.799	NA
Vescimonas	0.025	1.612	5.799	NA
Thermaurantiacus	0.224	1.612	5.799	NA
Provencibacterium	0.230	1.612	5.799	NA
Teredinibacter	0.038	0.635	5.795	NA
Pseudidiomarina	0.129	1.884	5.231	NA
Rhodophyticola	0.082	-0.717	5.786	NA
Cucumibacter	0.022	1.612	5.799	NA
Falseniella	0.389	-0.404	5.789	NA
Parapusillimonas	0.200	0.093	5.793	NA
Falcatimonas	0.017	0.680	5.796	NA
Lujinxingia	0.358	-0.029	4.183	NA
Acidaminococcus	0.185	2.288	5.789	NA
Geomonas	0.050	1.612	5.799	NA
Alkalihalophilus	0.129	1.612	5.799	NA
Pyricularia	0.300	0.730	5.794	NA
Humibacter	0.118	0.299	5.794	NA
Phytoactinopolyspora	0.463	-0.206	5.790	NA
Neokomagataea	0.056	1.612	5.799	NA
Drancourtella	0.234	1.612	5.799	NA
Escherichia_phage_D6_virus	0.000	0.000	0.000	NA
Desulfoscipio	0.435	0.029	5.793	NA
Neglectibacter	0.247	-0.148	3.332	NA
Zoogloeaceae_genus	0.238	-0.405	5.789	NA
Vampirovibrio	0.259	-1.221	5.783	NA
Pseudodesulfovibrio	0.057	1.612	5.799	NA
Allorhizobium	0.157	-0.287	5.790	NA
Pseudaestuariivita	0.239	0.162	5.793	NA
Macellibacteroides	0.431	1.612	5.799	NA
Caenispirillum	0.400	-2.120	5.777	NA
Hydrotalea	0.322	-0.248	5.790	NA
Desnuesiella	0.296	1.612	5.799	NA
Yinghuangia	0.394	0.147	3.891	NA
Enterobacteria_phage_Sf6_virus	0.000	0.000	0.000	NA
Methyloradius	0.301	1.920	5.798	NA
Robiginitalea	0.271	-1.543	5.781	NA
Usitatibacter	0.008	1.066	5.797	NA
Haloechinothrix	0.329	1.066	5.797	NA
Calorimonas	0.022	1.612	5.799	NA
Tistrella	0.157	-1.151	5.784	NA
Sphaerisporangium	0.405	1.612	5.799	NA
Nioella	0.006	1.612	5.799	NA

Chloroflexales	0.074	1.612	5.799	NA
Arenivirga	0.339	-1.346	5.782	NA
Pelistega	0.239	1.612	5.799	NA
Arboricoccus	0.363	2.820	5.795	NA
Propionispora	0.201	1.612	5.799	NA
Odoribacter	0.272	-1.818	5.779	NA
Miniphocaeibacter	0.144	0.469	5.795	NA
Betaproteobacteria	0.088	1.612	5.799	NA
Albitalea	0.203	-1.258	5.783	NA
Trichococcus	0.181	-0.189	5.791	NA
Tetzosporium	0.333	-2.348	5.776	NA
Aquirhabdus	0.376	4.438	5.782	NA
Methylocaldum	0.238	0.590	4.853	NA
Viridibacillus	0.463	1.228	5.208	NA
Aggregatilinea	0.100	0.872	5.796	NA
Fontimonas	0.265	-1.692	5.780	NA
Aceticella	0.332	1.612	5.799	NA
Flagellatimonas	0.061	-0.401	5.789	NA
Gloeothece	0.000	0.000	0.000	NA
Silanimonas	0.294	3.944	5.785	NA
Atopococcus	0.184	1.612	5.799	NA
Prosthecomicrobium	0.437	-0.574	5.753	NA
Propionibacterium_phage_PHL117M01_virus	0.268	1.612	5.799	NA
Carboxylicivirga	0.110	-0.601	5.787	NA
Thermopolyspora	0.034	1.612	5.799	NA
Rubricoccus	0.152	1.612	5.799	NA
Tepidanaerobacter	0.405	1.612	5.799	NA
Sulfuriferula	0.013	1.612	5.799	NA
Idiomarinaceae_genus	0.023	1.374	5.798	NA
Chryseosolibacter	0.194	1.612	5.799	NA
Komarekiella	0.170	1.612	5.799	NA
Pelovirga	0.002	1.612	5.799	NA
Pirellulimonas	0.144	-0.104	5.791	NA
Arachidicoccus	0.202	1.163	5.798	NA
Sulfuricystis	0.204	1.373	5.798	NA
Luteipulveratus	0.361	1.324	5.798	NA
Chryseolinea	0.170	1.612	5.799	NA
Pontibrevibacter	0.195	1.612	5.799	NA
Hanamia	0.253	1.612	5.799	NA
Grimontia	0.335	-0.356	1.736	NA
Thioclava	0.439	-2.443	5.776	NA
Propionibacterium_phage_SKKY_virus	0.362	3.319	5.791	NA
Citreimonas	0.088	1.612	5.799	NA
Planococcaceae_genus	0.288	-1.419	5.782	NA
Caldicellulosiruptor	0.237	1.022	5.797	NA
Hartmannibacter	0.457	1.612	5.799	NA
Pseudescherichia	0.062	0.674	5.796	NA
Akanthomyces	0.281	-1.620	5.780	NA
Plasticicumulans	0.004	1.612	5.799	NA
Escherichia_phage_520873_virus	0.000	0.000	0.000	NA
Bergeyella	0.183	0.767	5.796	NA
Stenoxybacter	0.289	1.612	5.799	NA
Cronobacter_phage_vB_CsaM_GAP32_virus	0.325	-0.601	5.787	NA
Plesiocystis	0.041	0.041	5.793	NA
Torulaspora	0.428	1.612	5.799	NA
Sulfuritalea	0.033	0.301	5.794	NA
Streptobacillus	0.298	1.612	5.799	NA
Viadribacter	0.000	0.000	0.000	NA
Propionibacterium_phage_P100D_virus	0.085	1.374	5.798	NA

Asanoa	0.412	1.612	5.799	NA
Petrotoga	0.426	-2.232	5.777	NA
Salinisphaera	0.371	0.358	2.159	NA
Chloroflexus	0.309	1.612	5.799	NA
Buchananella	0.056	1.612	5.799	NA
Frisingicoccus	0.052	0.091	5.793	NA
Pseudanabaena	0.388	0.909	5.797	NA
Effusibacillus	0.123	1.612	5.799	NA
Blattabacterium	0.399	0.657	5.795	NA
Corticibacterium	0.417	1.612	5.799	NA
Paramesorhizobium	0.152	0.301	5.794	NA
Betaproteobacterium_AAP99	0.003	1.612	5.799	NA
Oceanotoga	0.000	0.000	0.000	NA
Salmonella_phage_SJ46_virus	0.000	0.000	0.000	NA
Roseibaca	0.208	1.032	5.797	NA
Allofustis	0.129	-0.104	5.791	NA
Syntrophomonas	0.141	-0.171	5.791	NA
Paludicola	0.000	0.000	0.000	NA
Kaustia	0.000	0.000	0.000	NA
Pararobbsia	0.017	1.374	5.798	NA
Melittangium	0.138	1.612	5.799	NA
Robinsoniella	0.031	1.612	5.799	NA
