# Supplementary material for: Shotgun Metagenome Analysis of Two Schizaphis graminum Biotypes over Time With and Without Carried Cereal Yellow Dwarf Virus
Source: Insects. 2025 May 23;16(6):554. doi: 10.3390/insects16060554 (PMC12193481; doi:10.3390/insects16060554)
Supplement: Supplementary file 1 [file insects-16-00554-s001.zip › Table S3.pdf]

Table S3. DESeq2 results for comparison by time, arranged by log_2 fold change.

Genus	BaseMean	Log2FC	LFCSE	Padj
Parainfluenza_virus_5	10.436	-23.274	2.263	2.659e-01
Postia	23.021	-21.086	1.663	1.725e-03
Gloeophyllum	30.331	-20.925	2.039	5.846e-02
Actinobacteria	3.730	-20.887	3.372	1.000e+00
Leptomonas	3.765	-20.879	2.172	6.884e-01
Pontibacillus	32.118	-20.807	2.560	3.001e-01
Collibacillus	3.224	-18.897	4.372	9.962e-01
Aedoeadaptatus	14.710	-17.768	2.887	9.764e-02
Sandaracinus	3.521	-17.499	3.385	9.966e-01
Weizmannia	1.405	-17.180	5.316	1.000e+00
Cyberlindnera	3.470	-16.381	2.687	9.946e-01
Kallotenue	98.738	-8.434	1.048	1.405e-12
Allomeiothermus	372.615	-8.433	1.023	6.125e-13
Aureococcus	12.830	-7.445	1.639	2.970e-05
Porphyromonadaceae_genus	12.235	-7.042	1.315	2.309e-01
Armatimonadetes	144.045	-7.030	1.120	1.805e-09
Moesziomyces	251.864	-6.995	1.616	7.091e-13
Tetrahymena	10.703	-6.831	1.365	1.083e-05
Phytophthora	90.681	-6.648	0.610	4.356e-37
Dioszegia	17.319	-6.448	1.841	1.331e-01
Heterobasidion	23.873	-6.272	1.527	2.315e-05
Lutimaribacter	14.713	-6.223	1.123	2.302e-04
Sporisorium	4.490	-6.120	2.320	6.825e-03
Saprolegnia	9.754	-5.924	1.208	9.753e-04
Anaerococcus	1493.959	-5.874	0.980	1.301e-11
Oryzomicrobium	63.319	-5.855	0.609	8.629e-47
Mollisia	7.237	-5.855	1.678	1.275e-01
Phanerochaete	8.549	-5.830	1.574	9.141e-02
Gaiella	11.733	-5.809	1.960	4.619e-01
Dysgonamonadaceae_genus	19.860	-5.791	1.468	1.287e-02
Gleimia	229.791	-5.729	0.592	2.253e-21
Craterilacuibacter	127.435	-5.720	0.740	1.312e-21
Loigolactobacillus	25.133	-5.697	1.823	1.824e-02
Chelativorans	10.320	-5.587	1.543	3.230e-02
Paenisporosarcina	1.633	-5.561	5.800	1.000e+00
Pyrinomonas	9.505	-5.555	1.655	1.268e-02
Mogibacterium	9.735	-5.500	1.266	1.176e-02
Ichthyophthirius	11.762	-5.490	0.994	1.465e-07
Thermalbibacter	6.902	-5.443	2.020	3.222e-01
Cutaneotrichosporon	17.445	-5.437	1.661	2.108e-03
Thermoanaerobacterium	15.500	-5.405	1.820	4.248e-02
Cedecea	62.532	-5.388	0.541	6.088e-47
Caldifermentibacillus	6.086	-5.370	1.983	2.455e-02
Nanosynbacter	12.801	-5.360	1.246	8.417e-04
Herbiconiux	8.764	-5.309	2.137	8.748e-01
Facklamia	31.482	-5.287	0.798	1.886e-15
Neoantrodia	18.640	-5.255	1.771	4.826e-03
Domibacillus	6.862	-5.234	2.159	2.579e-02
Microbacteriaceae_genus	4.368	-5.215	2.359	3.077e-01
Kitasatospora	49.538	-5.197	1.128	1.254e-07
Panacagrimonas	4.713	-5.171	2.521	7.384e-02
Thalassiosira	9.266	-5.165	1.090	1.591e-05
Seramator	7.459	-5.126	1.440	1.409e-02
Orbilia	26.431	-5.123	1.464	7.471e-08
Phaeodactylum	12.627	-5.062	1.093	7.745e-04

Metarhizium	45.258	-5.018	1.164	4.553e-06
Bipolaris	14.314	-4.947	1.629	7.729e-03
Marinobacterium	1297.016	-4.935	0.466	6.710e-33
Pestalotiopsis	18.985	-4.929	1.072	1.299e-02
Neohortaea	3.383	-4.843	1.995	2.411e-01
Fannyhessea	5.504	-4.840	2.313	4.670e-01
Herbinix	3.656	-4.836	2.954	9.354e-01
Leishmania	4.184	-4.792	2.037	8.359e-01
Plasmopara	14.723	-4.777	1.426	3.222e-03
Exserohilum	4.737	-4.775	2.467	6.557e-01
Lamprocystis	7995.423	-4.774	0.342	3.006e-61
Perkinsus	5.227	-4.766	1.155	1.021e-03
Pleomorphomonas	12.823	-4.695	2.233	9.329e-02
Nannochloropsis	2.594	-4.694	2.222	5.606e-02
Musicola	448.106	-4.622	0.499	7.898e-25
Xanthomonadaceae_genus	5.216	-4.621	1.746	2.059e-01
Glarea	9.663	-4.605	1.448	1.077e-03
Rhizoctonia	14.717	-4.604	1.620	1.750e-03
Acidobacteria	5.876	-4.594	1.790	8.798e-03
Terrisporobacter	682.815	-4.583	0.330	3.111e-64
Filomicrobium	3.341	-4.568	2.160	3.493e-01
Shigella	210548.549	-4.567	0.225	8.589e-136
Methanotrophic	6.831	-4.544	1.644	5.743e-01
Escherichia	61277.548	-4.504	0.231	2.186e-124
Babesia	6.351	-4.498	1.301	5.014e-02
Photobacterium	11.366	-4.481	1.028	3.543e-02
Myceligenerans	6.325	-4.429	1.136	1.868e-04
Levyella	10.500	-4.422	2.289	5.718e-01
Clavibacter	6.175	-4.392	1.916	5.888e-01
Aphanomyces	5.189	-4.389	1.206	2.785e-02
Dactylellina	2.854	-4.354	2.678	4.017e-01
Stomatobaculum	7.662	-4.352	1.627	4.096e-01
Emticicia	4.218	-4.347	2.819	7.146e-01
Pararhizobium	14.351	-4.313	1.394	2.550e-01
Aureobasidium	23.442	-4.263	1.203	2.359e-03
Lysinibacillus	127.976	-4.248	0.627	9.311e-10
Abditibacterium	5.942	-4.221	1.952	6.949e-01
Pseudogymnoascus	14.590	-4.191	1.820	6.890e-03
Citrobacter	14367.687	-4.186	0.275	3.165e-74
Zoogloea	42.077	-4.176	0.618	1.908e-10
Fortiea	3.806	-4.173	2.685	1.000e+00
Marinilactibacillus	3.912	-4.158	2.253	9.966e-01
Human_adenovirus_2	5.562	-4.157	1.491	4.604e-02
Thermomicrobium	3.852	-4.140	1.740	3.592e-01
Eremomyces	1.147	-4.139	4.484	9.866e-01
Eremococcus	4.175	-4.128	1.843	9.042e-01
Algiphilus	4.070	-4.096	1.642	9.801e-02
Paucilactobacillus	8.021	-4.092	1.085	1.719e-05
Actinobacillus	273.019	-4.055	0.421	4.415e-32
Reyranella	10.238	-4.023	1.138	4.386e-02
Hallella	4.170	-3.995	1.911	9.369e-01
Chondromyces	2.094	-3.992	3.434	9.966e-01
Negativicoccus	0.611	-3.984	5.800	1.000e+00
Naegleria	20.303	-3.961	0.895	4.636e-05
Segatella	9.943	-3.961	1.203	4.102e-02
Glutamicibacter	58.869	-3.955	0.829	1.789e-07
Selenomonas	7.222	-3.937	1.145	1.551e-02
Fluviicola	6.282	-3.921	1.744	9.490e-01
Methylosinus	1.704	-3.917	2.273	6.844e-01

Wenzhouxiangella	8.641	-3.881	1.688	8.873e-04
Rhizopus	1.764	-3.861	2.957	6.256e-01
Enterobacteria_phage_phi80_virus	4.380	-3.850	1.798	4.076e-01
Betaproteobacterium_FWI2	1603.185	-3.833	0.536	9.863e-12
Gallibacter	30.774	-3.828	1.697	6.872e-02
Sporosarcina	5.918	-3.827	1.470	8.768e-03
Azotobacter	95.528	-3.819	0.789	2.544e-06
TM7	7.135	-3.806	1.373	4.790e-01
Rhodofomes	8.148	-3.803	1.686	2.030e-02
Enterobacter	9658.205	-3.798	0.318	3.461e-48
Grosmannia	2.498	-3.796	3.119	9.292e-01
Chitinimonas	19.480	-3.775	1.439	1.487e-01
Anaeroglobus	4.318	-3.773	1.549	2.466e-01
Heyndrickxia	2.903	-3.767	2.937	9.446e-01
Luteibacter	4.401	-3.731	1.634	2.113e-01
Levilactobacillus	112.639	-3.726	0.941	1.113e-02
Bacidia	8.750	-3.718	1.935	4.008e-01
Escherichia_phage_phiV10_virus	2.464	-3.690	2.031	6.943e-01
Robertmurraya	10.088	-3.672	1.221	1.874e-02
Conchiformibius	9.870	-3.659	1.685	2.134e-01
Trametes	7.826	-3.659	1.354	1.962e-02
Paludisphaera	3.132	-3.658	1.737	6.254e-01
Lachnoanaerobaculum	14.139	-3.658	0.829	1.149e-03
Tannerella	323.303	-3.638	0.384	1.956e-35
Salipaludibacillus	74.463	-3.633	0.887	7.240e-09
Xenorhabdus	3.486	-3.631	0.918	3.759e-03
Agarivorans	92.107	-3.593	1.095	1.198e-02
Frondihabitans	1.716	-3.578	2.500	7.036e-01
Schaalia	24.329	-3.571	0.935	1.808e-01
Allomuricauda	54.976	-3.554	0.549	5.046e-12
Kaistia	3.343	-3.546	2.015	8.106e-01
Rummeliibacillus	3.322	-3.540	1.640	2.398e-01
Desulfosporosinus	5.604	-3.517	1.282	1.984e-03
Pelosinus	4.274	-3.508	1.820	8.470e-01
Ruficoccus	65.274	-3.493	0.494	5.979e-21
Pseudozyma	25.743	-3.482	1.486	2.629e-03
Dialister	5.744	-3.477	1.961	6.307e-01
Chitinophaga	7.848	-3.474	1.256	1.517e-02
Microdochium	4.060	-3.473	1.552	4.722e-01
Salinicola	3.874	-3.462	1.169	2.354e-01
Sphaerotilus	3.359	-3.460	1.904	3.184e-01
Extensimonas	3.609	-3.452	2.063	5.967e-01
Methylocystis	2.390	-3.450	1.708	2.073e-01
Enterobacteria_phage_T4_virus	2.981	-3.448	2.302	7.894e-01
Mesomycoplasma	0.933	-3.444	4.794	1.000e+00
Halalkalibacterium	3.247	-3.441	1.797	5.997e-01
Microterricola	0.789	-3.434	4.791	1.000e+00
Lentisphaera	29.992	-3.433	0.419	1.818e-38
Planctopirus	0.764	-3.410	3.101	8.503e-01
Kosakonia	32.393	-3.408	0.836	2.474e-08
Secondary	3.884	-3.407	0.960	3.572e-03
Ensifer	36.688	-3.396	0.535	9.813e-09
Drepanopeziza	2.702	-3.389	2.197	5.941e-01
Paludibacterium	3.956	-3.377	1.541	1.248e-01
Apiotrichum	11.988	-3.372	1.454	5.348e-02
Acytostelium	1.048	-3.365	1.634	1.815e-01
Propioniciclava	35.109	-3.345	1.058	2.839e-05
Kalmanozyma	17.246	-3.333	1.454	1.322e-07
Daldinia	10.587	-3.322	2.199	6.972e-01

Erwinia	905.375	-3.296	0.425	2.348e-20
Dissoconium	0.726	-3.278	5.773	1.000e+00
Anaerotruncus	1.791	-3.275	1.894	2.781e-01
Truepera	5.824	-3.274	2.361	1.000e+00
Raoultella	68.520	-3.271	0.414	7.355e-32
Thecamonas	2.728	-3.269	2.688	5.099e-01
Kurthia	83.993	-3.265	0.549	3.549e-12
Frateuria	2.007	-3.263	1.594	6.972e-01
Thermus	128.332	-3.263	0.759	2.918e-03
Iamia	6.518	-3.259	2.289	8.141e-01
Actibacterium	0.985	-3.240	2.823	9.866e-01
Globicatella	2.180	-3.239	3.854	8.727e-01
Puccinia	4.914	-3.237	1.582	6.727e-01
Chroococcidiopsis	4.006	-3.229	1.936	7.275e-01
Oxalobacteraceae_genus	5.859	-3.218	1.875	3.455e-01
Rhodanobacter	6.156	-3.199	1.273	3.933e-02
Acidisphaera	2.480	-3.195	3.027	1.000e+00
Inhella	1.300	-3.187	3.515	1.000e+00
Fibroporia	7.444	-3.185	2.220	4.420e-01
Dictyostelium	11.036	-3.185	1.533	5.345e-03
Urbifossiella	0.741	-3.175	5.435	1.000e+00
Edwardsiella	89.782	-3.162	0.609	1.563e-15
Mycoplasmopsis	1.529	-3.156	2.402	1.000e+00
Lelliottia	2.324	-3.146	1.333	5.607e-01
Pseudoramibacter	123.529	-3.137	1.005	9.979e-04
Proteus	179.703	-3.130	0.580	1.980e-09
Pusillimonas	1729.460	-3.117	0.286	6.018e-48
Limobrevibacterium	0.674	-3.112	3.692	1.000e+00
Aurantiacibacter	6.751	-3.110	1.656	7.305e-01
Elizabethkingia	205.340	-3.108	0.334	7.336e-31
Thermicanus	19.342	-3.103	1.739	1.757e-01
Tumebacillus	31.849	-3.098	0.704	4.201e-08
Pseudocercospora	12.922	-3.095	1.321	4.248e-02
Yersinia	118.324	-3.091	0.641	7.917e-10
Rathayibacter	27.104	-3.080	1.250	3.933e-01
Ustilago	1.359	-3.076	2.869	1.000e+00
Paramagnetospirillum	1.385	-3.074	3.263	1.000e+00
Aquamicrobium	62.955	-3.061	0.631	5.797e-03
Alloscardovia	58.836	-3.054	0.714	3.472e-07
Okeania	8.897	-3.053	1.600	6.804e-01
Enterobacteria_phage_vB_EcoS_ACG-M12_virus	1.666	-3.051	2.346	8.052e-01
Lacticaseibacillus	6.204	-3.051	1.379	2.169e-01
Gemmatimonas	2.909	-3.048	2.681	9.138e-01
Wolbachia	68.134	-3.042	0.788	4.057e-02
Peptoniphilus	314.312	-3.040	1.010	2.739e-07
Rheinheimera	4037.545	-3.039	0.326	6.446e-32
Neurospora	2.010	-3.039	3.766	7.755e-01
Aureispira	3.069	-3.028	1.511	6.447e-02
Jaminaea	0.480	-3.018	5.774	1.000e+00
Phytobacter	18.093	-3.012	1.013	7.745e-04
Ilyomonas	0.448	-3.009	5.774	NA
Granulicella	0.826	-3.001	5.533	1.000e+00
Eutypa	3.140	-2.980	1.757	8.227e-01
Acaromyces	130.080	-2.971	1.695	1.362e-06
Saccharibacteria	4.408	-2.968	1.973	3.078e-01
Acetobacterium	3.427	-2.965	2.020	9.292e-01
Sordaria	2.130	-2.963	2.554	9.966e-01
Finegoldia	237.843	-2.946	1.098	1.134e-06
Pichia	1.167	-2.937	2.874	1.000e+00

Dyella	23.540	-2.934	0.673	4.493e-04
Tychonema	1.638	-2.928	2.436	1.000e+00
Rhodovulum	1.619	-2.924	2.725	6.333e-01
Gammaproteobacteria	3.215	-2.918	2.001	7.410e-01
Minicystis	1.842	-2.907	3.872	1.000e+00
Enterobacteria_phage_vB_EcoS_IME542_virus	2.844	-2.878	2.104	5.954e-01
Fusibacter	1.572	-2.865	3.781	1.000e+00
Pauljensenia	155.649	-2.862	0.829	3.976e-02
Purpureocillium	1.627	-2.861	5.761	1.000e+00
Rufibacter	2.548	-2.858	3.730	9.866e-01
Homoserinimonas	5.839	-2.858	1.498	3.460e-01
Minimicrobia	2.704	-2.851	2.055	8.499e-01
Klebsiella	1531.102	-2.847	0.217	3.317e-68
Anaerobiospirillum	0.687	-2.829	5.774	1.000e+00
Glaciibacter	18.801	-2.827	1.287	5.099e-01
Podospora	0.954	-2.823	1.516	9.342e-02
Aquabacter	1.821	-2.821	2.565	1.000e+00
Hydrogenophaga	66.646	-2.801	0.689	5.791e-05
Chelatococcus	4.009	-2.775	1.832	8.334e-01
Truncatella	1.191	-2.744	3.207	1.000e+00
Verrucomicrobia	1.670	-2.716	3.485	1.000e+00
Epithele	1.800	-2.699	2.344	7.272e-01
Micavibrio	0.622	-2.699	5.027	1.000e+00
Phreatobacter	2.827	-2.698	1.843	6.340e-01
Escherichia_phage_vB_EcoS_ESCO41_virus	1.030	-2.697	3.136	1.000e+00
Fibrisoma	18.035	-2.690	1.027	3.274e-02
Rahnella	196.146	-2.688	0.483	9.639e-11
Lawsonella	195.241	-2.686	0.711	5.462e-06
Bacteroides	124.758	-2.686	0.334	1.113e-19
Desulfitobacterium	6.033	-2.674	1.541	3.622e-02
Gluconacetobacter	6.748	-2.670	1.200	2.550e-01
Acidaminobacter	12.532	-2.660	1.309	2.055e-03
Pseudomassariella	0.529	-2.652	5.775	1.000e+00
Serratia	400.268	-2.650	0.497	1.210e-26
Leyella	1.037	-2.648	2.705	8.290e-01
Aerococcus	77.226	-2.647	0.475	4.316e-09
Saliphagus	1.041	-2.645	2.523	1.000e+00
Microbispora	62.361	-2.632	0.474	7.156e-12
Parerythrobacter	3.953	-2.629	2.762	7.034e-01
Fretibacterium	3.170	-2.624	2.226	5.978e-01
Defluviimonas	0.700	-2.623	3.506	1.000e+00
Tolypothrix	11.518	-2.619	1.773	8.503e-01
Aquimarina	1.034	-2.607	4.817	1.000e+00
Halophilic	1.183	-2.607	3.613	1.000e+00
Ramularia	0.493	-2.603	5.775	1.000e+00
Stigmatella	0.634	-2.597	5.775	1.000e+00
Scardovia	0.474	-2.595	5.775	1.000e+00
Actinophytocola	1.747	-2.594	3.432	1.000e+00
Salifodinibacter	1.878	-2.592	3.676	1.000e+00
Aspergillus	116.582	-2.592	0.613	1.889e-04
Sphingorhabdus	2.961	-2.589	2.443	6.256e-01
Agilicoccus	113.618	-2.580	1.036	2.080e-03
Escherichia_phage_DTL_virus	0.756	-2.577	4.597	1.000e+00
Flavihumibacter	2.570	-2.573	1.735	8.801e-01
Cercospora	17.883	-2.570	1.117	2.745e-02
Peptostreptococcus	6.314	-2.561	1.377	9.321e-01
Marinobacter	150.235	-2.559	0.353	1.545e-20
Roseibium	0.641	-2.513	3.780	1.000e+00
Tuber	2.943	-2.512	2.674	1.000e+00

Prevotella	338.175	-2.505	0.527	1.567e-06
Gulosibacter	3.895	-2.505	2.117	4.339e-01
Aliiruegeria	16.063	-2.500	1.144	2.411e-01
Aquitalea	1.946	-2.497	1.520	1.062e-01
Planctomyces	2.081	-2.496	2.164	8.305e-01
Aestuariivirga	0.693	-2.493	5.522	1.000e+00
Xanthomarina	0.828	-2.474	3.539	1.000e+00
Vibrionimonas	0.863	-2.473	4.378	1.000e+00
Jatrophihabitans	13.273	-2.468	2.066	3.327e-01
Afipia	1012.669	-2.467	0.371	2.260e-13
Sorangium	0.422	-2.460	5.776	NA
Coraliihabitans	7.777	-2.460	1.253	1.540e-01
Kickxella	0.850	-2.459	4.230	1.000e+00
Acidocella	1.112	-2.456	3.149	1.000e+00
Thioclava	0.439	-2.443	5.776	NA
Neofusicoccum	135.521	-2.439	0.968	9.881e-10
Bacteriovorax	1.730	-2.436	3.024	1.000e+00
Rhodopirellula	20.297	-2.428	0.828	2.326e-01
Dysosmobacter	0.526	-2.421	5.776	1.000e+00
Plantibacter	4.413	-2.420	2.018	1.000e+00
Castellaniella	1.632	-2.418	2.980	6.844e-01
Diaphorobacter	35.714	-2.404	0.653	7.462e-08
Betaproteobacterium_AAP121	0.906	-2.398	2.360	1.000e+00
Yonghaparkia	1.044	-2.395	3.138	9.031e-01
Glaciihabitans	0.993	-2.394	3.339	1.000e+00
Pectinatus	4.713	-2.394	1.415	2.326e-01
Marisediminicola	0.852	-2.393	5.589	1.000e+00
Caldibacillus	8.783	-2.390	1.744	1.577e-01
Luteolibacter	24.040	-2.381	0.772	2.610e-02
Morococcus	313.297	-2.376	0.475	4.102e-06
Tatumella	7.235	-2.365	1.234	9.076e-01
Aureibaculum	51.891	-2.363	0.675	1.184e-03
Mesonia	2.400	-2.361	1.415	4.000e-01
Pochonia	0.554	-2.358	3.464	1.000e+00
Fusarium	242.795	-2.358	1.012	3.911e-10
Frankia	114.940	-2.350	0.506	8.164e-05
Tetzosporium	0.333	-2.348	5.776	NA
Entotheonella	5.670	-2.348	1.449	5.125e-01
Nitrolancea	1.083	-2.344	4.029	1.000e+00
Trichoderma	47.821	-2.342	1.264	3.755e-02
Ruminococcus	45.967	-2.339	0.999	1.400e-03
Pimelobacter	6.825	-2.338	1.482	5.214e-01
Motilimonas	7.460	-2.328	1.128	3.137e-01
Erysipelotrichaceae_genus	0.333	-2.320	5.776	NA
Fonticella	1.327	-2.313	5.240	1.000e+00
Geodermatophilaceae_genus	0.845	-2.310	3.827	1.000e+00
Hafnia	2.018	-2.295	0.985	2.743e-03
Gulbenkiania	0.803	-2.290	4.371	1.000e+00
Evansella	0.774	-2.287	5.776	1.000e+00
Gemmata	2.366	-2.281	3.082	6.391e-01
Microcystis	6710.710	-2.281	0.633	9.528e-06
Diplodia	6.906	-2.277	1.646	1.362e-01
Nostoc	400.525	-2.275	0.494	1.287e-06
Laccaria	12.373	-2.272	1.557	4.908e-04
Halococcus	1.068	-2.258	1.781	4.008e-01
Faecalibacterium	6.785	-2.256	1.561	9.368e-02
Psilocybe	1.438	-2.255	1.858	1.000e+00
Cadophora	1.438	-2.245	2.433	1.000e+00
Protofrankia	75.565	-2.236	0.730	1.204e-03

Saccharibacillus	2.894	-2.236	2.819	1.000e+00
Fulvia	6.635	-2.235	1.378	1.978e-02
Petrotoga	0.426	-2.232	5.777	NA
Helicobacter	0.471	-2.222	5.777	1.000e+00
Pseudoxanthomonas	97.563	-2.222	0.611	2.503e-05
Gallintestinimicrobium	0.653	-2.222	5.777	1.000e+00
Coriobacteriales	0.346	-2.221	5.777	NA
Labedella	0.902	-2.214	4.461	1.000e+00
Enterobacteria_phage_RTP_virus	2.620	-2.213	1.823	3.319e-01
Propionicimonas	3.302	-2.210	2.215	5.099e-01
Veillonella	194.159	-2.203	0.626	2.079e-02
Pseudokineococcus	6.411	-2.198	2.569	1.000e+00
Conyzicola	1.343	-2.192	2.629	1.000e+00
Cupidesulfovibrio	28.579	-2.191	0.755	4.111e-03
Thermacetogenium	1.244	-2.189	5.777	1.000e+00
Glycomyces	1.173	-2.187	4.099	1.000e+00
Rudaea	1.559	-2.178	1.506	9.648e-01
Anoxybacillus	61.693	-2.178	1.357	2.656e-02
Pseudorhodoplanes	0.900	-2.173	2.744	1.000e+00
Azomonas	2.713	-2.167	1.893	1.000e+00
Coprothermobacter	0.251	-2.158	5.777	NA
Saccharimonas	0.553	-2.157	5.157	1.000e+00
Huaxiibacter	3.774	-2.151	1.955	1.000e+00
Brevibacillus	1.713	-2.150	1.917	6.123e-01
Butyrivibrio	0.998	-2.149	3.644	9.966e-01
Thiolapillus	20.111	-2.138	1.463	6.238e-02
Chiayiivirga	1.205	-2.135	4.691	1.000e+00
Peredibacter	28.482	-2.134	1.152	4.995e-01
Caenispirillum	0.400	-2.120	5.777	NA
Filamentous	0.917	-2.119	4.077	1.000e+00
Tepidiphilus	149.559	-2.114	1.216	2.209e-05
Penicilliopsis	0.668	-2.108	5.776	1.000e+00
Richelia	0.517	-2.093	4.151	1.000e+00
Fenollaria	0.551	-2.092	3.826	1.000e+00
Halovibrio	10.136	-2.088	1.227	3.413e-01
Pseudacidovorax	63.479	-2.086	0.557	2.104e-04
Simian_virus_40	1.423	-2.083	1.684	6.557e-01
Polymorphobacter	8.366	-2.075	1.719	5.997e-01
Leptospira	69.642	-2.066	0.527	1.502e-02
Blastomyces	1.591	-2.061	1.781	9.011e-01
Defluviicoccus	0.374	-2.054	5.778	NA
Jiangella	1.073	-2.043	3.599	1.000e+00
Cryptosporidium	15.505	-2.038	0.981	4.207e-01
Atopobium	11.220	-2.034	1.269	6.026e-01
Riemerella	4.363	-2.034	1.842	9.369e-01
Alkalicoccobacillus	0.836	-2.031	1.588	5.629e-01
Duffyella	6.269	-2.028	1.536	5.979e-02
Planctomonas	7.195	-2.026	1.831	4.869e-01
Desertibacillus	1.140	-2.018	3.540	1.000e+00
Methylophilus	8.689	-2.013	1.074	5.860e-01
Desarmillaria	3.626	-2.011	1.696	8.103e-01
Rhodotorula	54.705	-2.008	1.118	9.105e-03
Gluconobacter	9.302	-2.004	1.291	9.031e-01
Naumannella	5.114	-1.998	1.777	1.000e+00
Parvimonas	4.539	-1.993	1.646	6.761e-01
Ferrovum	0.818	-1.988	5.601	1.000e+00
Malikia	0.578	-1.985	5.176	1.000e+00
Proteiniclasticum	1.269	-1.983	1.721	6.301e-01
Paraferrimonas	4.903	-1.982	1.281	1.076e-01

UNVERIFIED_CONTAM:	2.089	-1.982	2.620	7.688e-01
Bdellovibrio	0.992	-1.973	2.750	1.000e+00
Acidihalobacter	28.945	-1.965	1.043	4.661e-01
Metasolibacillus	1.157	-1.964	2.615	8.387e-01
Apibacter	18.031	-1.964	1.849	8.164e-04
Fusobacterium	181.144	-1.955	0.318	3.663e-14
Hahella	2.380	-1.949	1.313	1.989e-03
Solobacterium	7.141	-1.941	1.407	9.011e-01
Pseudobdellovibrio	0.385	-1.939	5.259	NA
Scedosporium	0.752	-1.937	4.132	1.000e+00
Rouxiella	0.459	-1.934	5.056	NA
Chryseomicrobium	0.641	-1.932	3.964	1.000e+00
Punctularia	29.052	-1.932	1.785	2.851e-03
Aurantimonas	8.757	-1.931	1.076	1.035e-01
Puniceibacterium	0.603	-1.930	4.298	1.000e+00
Moritella	186.504	-1.919	0.241	6.636e-18
Parasutterella	0.525	-1.919	3.021	1.000e+00
Nevskia	7.692	-1.909	2.104	5.782e-01
Methylopila	6.130	-1.907	1.722	2.593e-01
Propionimicrobium	84.480	-1.881	0.897	7.767e-05
Lichenibacterium	2.044	-1.875	3.084	1.000e+00
Kwoniella	10.704	-1.875	1.845	1.521e-01
Caenimonas	0.820	-1.874	3.015	1.000e+00
Desulfogranum	1.068	-1.872	3.375	1.000e+00
Azospira	27.304	-1.872	0.591	5.747e-03
Gallionella	0.693	-1.870	2.154	1.000e+00
Colletotrichum	63.127	-1.868	0.719	8.605e-05
Frigoribacterium	39.917	-1.867	1.392	3.081e-02
Tahibacter	0.454	-1.866	3.922	NA
Cryptococcus	7.682	-1.857	1.585	6.031e-01
Dactylosporangium	0.221	-1.854	5.779	NA
Pseudooceanicola	112.214	-1.849	0.939	1.182e-04
Lautropia	241.645	-1.840	0.332	4.814e-09
Diolcogaster_facetosa_bracovirus	12.306	-1.837	1.143	9.446e-01
Lactococcus	112.493	-1.830	0.780	3.351e-01
Paludifilum	60.826	-1.823	0.553	3.997e-05
Pedobacter	122.644	-1.822	0.500	4.844e-03
Flavonifractor	0.231	-1.819	5.779	NA
Odoribacter	0.272	-1.818	5.779	NA
Cloacibacterium	93.517	-1.818	0.795	1.373e-05
Marinilabiliaceae_genus	4.842	-1.812	1.591	4.137e-02
Phycomyces	1.988	-1.807	2.169	1.964e-01
Clostridium	1001.246	-1.804	0.314	3.251e-07
Paracoccidioides	3.115	-1.798	1.481	2.867e-03
Sandaracinobacteroides	10.046	-1.792	1.673	8.174e-01
Cereal_yellow_dwarf_virus	0.223	-1.783	5.779	NA
Zeimonas	0.426	-1.774	5.778	NA
Theileria	0.473	-1.772	2.405	9.966e-01
Sodalis	1.787	-1.770	0.849	6.077e-03
Phialemonium	0.746	-1.767	5.137	1.000e+00
Herminiimonas	2.008	-1.753	2.845	1.000e+00
Planococcus	196.283	-1.747	0.346	4.662e-05
Thermincola	0.911	-1.743	5.467	1.000e+00
Rhodobacter	87.074	-1.742	0.461	3.885e-03
Oribacterium	27.835	-1.741	1.246	6.422e-02
Zimmermannella	7.604	-1.737	1.566	1.000e+00
Pelorhabdus	4.813	-1.733	1.384	1.758e-01
Ferruginibacter	1.198	-1.726	2.890	9.946e-01
Hydrocarboniphaga	1.904	-1.725	2.584	1.000e+00

Oceanospirillum	4.069	-1.722	1.248	1.526e-01
Aliidiomarina	6.184	-1.719	1.225	2.329e-02
Rosenbergiella	1.423	-1.717	1.431	6.256e-01
Singulisphaera	3.765	-1.716	1.684	7.313e-01
Chromobacterium	7.322	-1.716	0.963	4.753e-02
Tricharina	3.354	-1.712	2.422	5.108e-01
Betaproteobacterium_AAP65	0.934	-1.708	2.432	1.000e+00
Humisphaera	2.628	-1.705	3.017	1.000e+00
Neoactinobaculum	0.366	-1.702	5.780	NA
Marichromatium	2.010	-1.702	1.124	1.237e-02
Microcella	4.645	-1.700	1.757	1.000e+00
Mycoplasma	2.586	-1.696	1.752	3.809e-02
Hoylesella	15.245	-1.694	1.136	8.503e-01
Fontimonas	0.265	-1.692	5.780	NA
Labilibacter	4.141	-1.689	1.733	1.000e+00
Moorena	7.922	-1.679	1.677	6.209e-01
Phytoplasma	62.472	-1.667	0.557	1.667e-02
Westeberhardia	22.020	-1.667	0.725	8.898e-08
Gemella	167.060	-1.661	0.758	1.149e-03
Oceanicella	2.913	-1.647	1.742	6.254e-01
Salinibacterium	8.132	-1.646	1.258	4.213e-01
Mannheimia	5.616	-1.645	1.037	8.204e-02
Thermohydrogenium	1.212	-1.645	5.354	1.000e+00
Stereum	19.572	-1.643	1.395	7.446e-04
Lipomyces	0.316	-1.639	5.779	NA
Parachlamydiaceae_genus	0.346	-1.639	5.780	NA
Arsukibacterium	2.520	-1.633	2.490	2.285e-01
Subtercola	1.074	-1.631	4.060	1.000e+00
Patulibacter	14.300	-1.629	1.609	5.099e-01
Catenibacterium	1.044	-1.626	4.350	1.000e+00
Akanthomyces	0.281	-1.620	5.780	NA
Klenkia	7.551	-1.614	1.498	9.264e-01
Paraconexibacter	6.607	-1.599	2.078	2.204e-02
Bradyrhizobium	4376.605	-1.593	0.278	2.868e-10
Colwellia	7.776	-1.574	1.010	2.409e-01
Nesterenkonia	94.391	-1.570	0.597	5.187e-05
Kockovaella	3.506	-1.567	2.013	6.333e-01
Enterovirga	5.894	-1.565	1.981	1.000e+00
Camelimonas	0.500	-1.554	5.781	1.000e+00
Tomitella	1.592	-1.551	2.837	9.773e-01
Robiginitalea	0.271	-1.543	5.781	NA
Roseibacterium	113.257	-1.539	0.885	1.623e-01
Fictibacillus	387.233	-1.534	0.716	6.843e-02
Kuraishia	0.224	-1.531	5.781	NA
Rhodovibrio	0.439	-1.531	5.781	NA
Meiothermus	30.189	-1.526	1.127	9.762e-01
Methanothermobacter	0.537	-1.523	5.781	1.000e+00
Thiofilum	1.021	-1.510	5.045	1.000e+00
Saccharophagus	0.820	-1.507	4.009	1.000e+00
Talaromyces	8.852	-1.505	1.371	6.056e-01
Psychromonas	1.967	-1.502	1.422	9.369e-01
Duganella	59.734	-1.494	0.655	2.777e-04
Brasilonema	1.716	-1.488	3.255	1.000e+00
Amygdalobacter	0.251	-1.488	5.781	NA
Ogataea	1.139	-1.486	3.132	1.000e+00
Rhodocyclus	0.572	-1.477	3.482	1.000e+00
Methylocapsa	1.039	-1.468	2.520	9.974e-01
Terriglobus	2.580	-1.463	2.584	5.549e-01
Photorhabdus	73.809	-1.455	0.773	4.000e-06

Aquihabitans	4.924	-1.451	2.139	7.914e-01
Gayadomonas	2.348	-1.450	1.106	6.392e-01
Neisseria	439.766	-1.444	0.379	7.745e-04
Plectonema	0.287	-1.436	5.781	NA
Tissierellia	6.176	-1.426	2.312	1.758e-01
Planococcaceae_genus	0.288	-1.419	5.782	NA
Amorphotheca	13.824	-1.412	1.445	1.222e-01
Leptolyngbya	41.961	-1.410	0.774	8.150e-02
Baudoinia	14.011	-1.409	0.980	6.256e-01
Alicycliphilus	12.250	-1.406	0.910	5.736e-01
Pseudomonas	23142.274	-1.402	0.379	3.640e-09
Alloprevotella	42.819	-1.395	0.944	6.571e-01
Eikenella	13.478	-1.393	1.212	4.000e-02
Ornithinicoccus	1.709	-1.392	5.502	1.000e+00
Anaerobacillus	0.305	-1.386	5.782	NA
Rivularia	0.796	-1.376	4.511	1.000e+00
Intestinirhabdus	0.996	-1.373	0.926	9.351e-02
Aggregatibacter	18.736	-1.369	0.867	6.844e-01
Serpula	13.896	-1.363	2.232	9.605e-02
Staphylococcus	2906.978	-1.360	0.618	1.192e-05
Arenivirga	0.339	-1.346	5.782	NA
Meira	243.896	-1.338	1.934	1.497e-04
Naasia	3.058	-1.332	2.346	8.924e-01
Sphingobacterium	129.321	-1.326	0.602	5.043e-01
Aquisphaera	1.528	-1.323	2.658	1.000e+00
Micromonospora	101.944	-1.322	0.815	1.348e-03
Tolumonas	0.755	-1.318	5.081	1.000e+00
Methylobacterium	5697.290	-1.311	0.330	2.620e-03
Paraphaeosphaeria	1.127	-1.310	5.367	1.000e+00
Glaesserella	0.891	-1.309	1.856	8.189e-01
Acetobacter	24.983	-1.295	0.724	6.271e-05
Asinibacterium	2.381	-1.294	1.988	1.000e+00
Micrococcaceae_genus	0.386	-1.284	5.143	NA
Faunimonas	1.650	-1.277	5.611	1.000e+00
Barrientosiimonas	8.157	-1.276	2.124	1.000e+00
Caulobacter	1070.743	-1.275	0.397	1.939e-07
Coprobacillus	2.469	-1.270	2.444	5.826e-01
Pelomonas	2109.665	-1.267	0.298	4.408e-07
Enterobacteriaceae_genus	24.824	-1.267	0.420	9.941e-17
Aureimonas	24.555	-1.263	0.999	3.300e-02
Ilumatobacter	3.893	-1.259	2.228	1.000e+00
Albitalea	0.203	-1.258	5.783	NA
Hansschlegelia	2.156	-1.252	2.905	1.000e+00
Dysgonomonas	10.780	-1.244	0.875	6.831e-01
Oscillatoria	1.378	-1.241	2.613	1.000e+00
Phocaeicola	15.829	-1.233	1.244	9.507e-01
Nonomuraea	1.439	-1.233	2.505	1.000e+00
Jeotgalibacillus	1.208	-1.231	2.216	8.549e-01
Calidifontibacter	0.577	-1.227	5.781	1.000e+00
Vampirovibrio	0.259	-1.221	5.783	NA
Chthonobacter	0.205	-1.214	5.783	NA
Paraflavitalea	0.310	-1.203	5.783	NA
Sparassis	5.100	-1.201	1.459	3.592e-01
Actinokineospora	59.425	-1.201	1.107	3.448e-01
Salmonella	451.456	-1.194	0.292	1.953e-03
Zasmidium	2.768	-1.190	1.755	3.098e-01
Gloeocapsa	4.503	-1.185	2.024	9.966e-01
Flavobacterium	706.963	-1.182	0.282	4.121e-03
Phototrophicus	0.608	-1.167	5.717	1.000e+00

Aliterella	15.423	-1.165	2.266	7.914e-01
Frankineae	1.069	-1.162	5.359	1.000e+00
Thermosinus	0.439	-1.152	5.784	NA
Tistrella	0.157	-1.151	5.784	NA
Actinomarinicola	1.651	-1.146	2.745	1.000e+00
Terrimonas	78.628	-1.140	1.042	5.797e-03
Alkaliphilus	9.787	-1.139	2.738	3.551e-02
Phaeobacter	1.043	-1.136	1.756	9.046e-01
Rhabdonatronobacter	170.476	-1.127	0.469	2.073e-01
Labilithrix	4.216	-1.123	2.171	1.000e+00
Aestuariibaculum	1.183	-1.123	2.489	1.000e+00
Paeniroseomonas	0.513	-1.120	4.253	1.000e+00
Beijerinckia	0.593	-1.119	5.089	1.000e+00
Lachnellula	2.618	-1.109	1.609	1.000e+00
Bowmanella	86.909	-1.109	0.731	9.553e-04
Sporothrix	0.672	-1.109	3.692	1.000e+00
Endosymbiont	5.833	-1.100	1.164	1.729e-01
Virgibacillus	35.407	-1.095	0.526	2.881e-01
Nanosynsacchari	0.896	-1.091	3.276	1.000e+00
Debaryomyces	0.735	-1.090	4.997	1.000e+00
Rhodocyclaceae_genus	4.038	-1.089	1.779	7.876e-01
Leptotrichia	40.411	-1.088	0.650	3.349e-01
Oryzihumus	0.119	-1.084	5.784	NA
Verticillium	0.616	-1.073	3.976	1.000e+00
Flavipsychrobacter	0.537	-1.067	5.784	1.000e+00
Maribellus	40.625	-1.066	0.698	9.507e-01
Tetrasphaera	13.715	-1.064	1.317	1.550e-01
Ureibacillus	0.798	-1.060	3.498	1.000e+00
Pediococcus	0.682	-1.055	2.352	1.000e+00
Burkholderiaceae_genus	42.128	-1.053	0.672	5.970e-01
Blastomonas	151.122	-1.052	0.527	1.165e-03
Alkalispirochaeta	9160.805	-1.027	0.447	5.157e-02
Sanguibacter	1.792	-1.027	2.776	1.000e+00
Solimonas	5.862	-1.021	1.733	8.836e-01
Fimicolochytrium	92.915	-1.018	1.780	4.408e-07
Chromohalobacter	65.014	-1.015	0.400	3.193e-02
Nanoperiomorbus	1.569	-1.014	3.258	1.000e+00
Mixta	7.632	-1.011	1.122	5.549e-01
Idiomarina	0.677	-1.008	5.785	1.000e+00
Kytococcus	23.988	-1.001	0.976	5.604e-01
Pseudogemmobacter	0.782	-1.001	5.785	1.000e+00
Shewanella	593.636	-0.994	0.247	3.516e-06
Glaciecola	6.760	-0.990	0.740	8.836e-01
Galbitalea	2.705	-0.988	2.710	1.000e+00
Amycolatopsis	5.663	-0.982	1.038	8.459e-01
Cobetia	0.433	-0.982	3.319	NA
Halorubrum	12.131	-0.978	0.837	4.467e-01
Telluria	36.691	-0.975	1.059	1.163e-03
Bhargavaea	9.142	-0.966	1.227	9.446e-01
Ehrlichia	3.296	-0.948	2.111	3.077e-01
Aquariibacter	0.913	-0.940	2.907	1.000e+00
Cryphonectria	1.076	-0.938	2.886	1.000e+00
Saccharopolyspora	62.086	-0.937	0.371	2.725e-06
Rugamonas	51.529	-0.929	0.906	4.086e-06
Insolitispirillum	2.216	-0.928	2.391	9.507e-01
Auritidibacter	1.179	-0.925	5.785	1.000e+00
Paracraurococcus	0.696	-0.925	4.240	1.000e+00
Gamsiella	11.897	-0.924	1.999	4.641e-02
Bartonella	9.625	-0.917	1.070	2.063e-01

Halopseudomonas	2.577	-0.907	1.746	8.727e-01
Mucilaginibacter	56.373	-0.906	0.594	6.943e-01
Alcaligenes	8.410	-0.902	1.201	3.057e-03
Aquibium	0.920	-0.900	4.085	1.000e+00
Providencia	883.343	-0.899	0.270	8.543e-06
Diaporthe	8.192	-0.894	2.179	6.254e-01
Pseudonocardiaceae_genus	2.564	-0.881	3.494	8.727e-01
Microbacterium	1220.119	-0.881	0.247	2.758e-03
Pyxidicoccus	1.544	-0.878	2.401	9.762e-01
Parageobacillus	3.984	-0.873	2.831	7.529e-01
Synechococcus	0.880	-0.873	3.226	1.000e+00
Prevotellaceae_genus	5.849	-0.873	1.320	3.455e-01
Dankookia	0.909	-0.868	3.299	1.000e+00
Lachnospiraceae_genus	22.274	-0.856	0.854	7.779e-02
Amnibacterium	9.282	-0.853	1.606	9.966e-01
Lentibacillus	34.801	-0.848	0.514	2.063e-01
Intrasporangiaceae_genus	0.940	-0.847	2.820	1.000e+00
Noviherbaspirillum	23.982	-0.839	1.354	7.703e-01
Faecalicatena	0.820	-0.830	3.443	1.000e+00
Gamma	2.276	-0.827	2.274	9.974e-01
Hyaloscypha	3.026	-0.827	1.777	6.844e-01
Treponema	11.715	-0.824	1.068	1.000e+00
Neoarthrinium	1.024	-0.823	3.333	1.000e+00
Congregibacter	1.218	-0.821	1.892	1.320e-01
Simplicispira	1.925	-0.819	2.019	1.000e+00
Janibacter	177.517	-0.813	0.422	8.261e-04
Collinsella	12.119	-0.811	1.630	5.970e-01
Sphingomonas-like	0.439	-0.803	2.873	NA
Pantoea	228.056	-0.803	0.268	4.834e-03
Phormidium	0.945	-0.800	3.550	1.000e+00
Luteimonas	107.126	-0.795	1.100	2.345e-01
Malassezia	1094.910	-0.783	0.520	4.619e-01
Modestobacter	77.884	-0.782	0.522	1.758e-01
Phycicoccus	66.900	-0.781	0.495	2.659e-01
Variovorax	87.909	-0.758	0.385	2.946e-01
Enhydrobacter	51.501	-0.753	0.554	4.958e-01
Kingella	8.778	-0.744	1.066	1.000e+00
Corynebacterium	3523.431	-0.740	0.598	5.490e-07
Catonella	3.258	-0.730	1.987	1.000e+00
Arachnia	16.113	-0.729	1.130	1.000e+00
Wallemia	78.999	-0.720	1.703	3.444e-05
Rhodophyticola	0.082	-0.717	5.786	NA
Granulicatella	28.531	-0.713	0.867	7.899e-02
Qipengyuania	57.461	-0.712	0.781	4.959e-01
Flavobacteriaceae_genus	0.778	-0.711	3.121	1.000e+00
Thioflexithrix	10.033	-0.705	0.882	2.957e-01
Desulfovibrio	71087.628	-0.705	0.437	4.049e-01
Immundisolibacter	1.554	-0.703	2.628	1.000e+00
Halobacillus	0.605	-0.702	2.756	1.000e+00
Spirosoma	14.170	-0.694	1.245	2.186e-01
Streptococcus	1476.802	-0.693	0.580	6.883e-01
Qaidamihabitans	7.592	-0.692	2.238	5.545e-01
Larkinella	0.768	-0.691	3.707	1.000e+00
Pasteurella	1.606	-0.681	1.354	5.941e-01
Gemmataceae_genus	0.636	-0.672	5.104	1.000e+00
Solibacillus	6.613	-0.667	1.287	5.422e-01
Lasiodiplodia	27.457	-0.666	0.687	4.292e-01
Collimonas	9.925	-0.665	1.416	5.040e-01
Mesobacillus	0.966	-0.662	3.342	1.000e+00

Limnohabitans	19.440	-0.661	0.657	2.088e-01
Lawsonibacter	0.311	-0.653	5.785	NA
Putridiphycobacter	2.985	-0.650	1.604	8.886e-01
Cereibacter	5.029	-0.650	1.587	8.888e-01
Parachlamydia	0.456	-0.644	5.787	NA
Faecalimonas	1.189	-0.636	3.521	1.000e+00
Terracoccus	15.641	-0.636	1.607	1.000e+00
Puia	26.661	-0.634	0.600	1.000e+00
Phenylobacterium	73.211	-0.630	0.733	3.592e-02
Abiotrophia	14.388	-0.630	1.243	9.966e-01
Mycobacterium	323.882	-0.621	0.359	1.910e-01
Lindgomyces	0.223	-0.619	5.787	NA
Nitrotoga	4.783	-0.617	1.153	7.990e-01
Xylella	2.546	-0.610	1.275	1.000e+00
Haladaptatus	2.468	-0.605	1.665	7.290e-01
Sediminibacterium	6.108	-0.603	2.402	7.416e-01
Sinisalibacter	0.394	-0.601	5.400	NA
Cronobacter_phage_vB_CsaM_GAP32_virus	0.325	-0.601	5.787	NA
Carboxylicivirga	0.110	-0.601	5.787	NA
Fontibacillus	0.077	-0.600	5.787	NA
Grimontella	3.770	-0.598	1.507	1.000e+00
Polaribacter	8.861	-0.597	0.835	1.753e-01
Rhodopseudomonas	31.634	-0.597	0.720	1.000e+00
Agromyces	13.851	-0.594	0.829	9.454e-01
Bacillus	2815.520	-0.577	0.144	1.692e-06
Prosthecomicrobium	0.437	-0.574	5.753	NA
Enterococcus	585.175	-0.572	0.227	1.153e-05
Ewingella	0.845	-0.571	5.767	1.000e+00
Mycosynbacter	1.754	-0.569	2.758	1.000e+00
Shimia	358.445	-0.568	0.557	9.647e-03
Cardiobacterium	13.923	-0.557	1.156	9.446e-01
Propionibacterium_phage_PHL116M00_virus	0.473	-0.554	5.788	1.000e+00
Pleionea	5.060	-0.541	1.552	6.447e-02
Rhodoplanes	8.916	-0.534	1.121	8.727e-01
Salinicoccus	15.586	-0.533	1.216	2.169e-01
Ancylobacter	5.043	-0.522	1.966	1.000e+00
Planktothrix	6.905	-0.518	1.274	1.000e+00
Scandinavium	0.642	-0.516	1.311	6.943e-01
Chthoniobacter	2.198	-0.490	3.240	1.000e+00
Cyclobacterium	9.163	-0.486	0.730	5.263e-01
Paenirhodobacter	23.041	-0.485	0.699	7.688e-01
Dickeya	1.888	-0.483	0.911	9.966e-01
Crenobacter	1.823	-0.483	1.904	3.894e-01
Moraxella	437.707	-0.481	0.479	2.113e-01
Thermoleophilum	0.477	-0.478	5.788	1.000e+00
Alloalcanivorax	2.425	-0.476	2.061	1.000e+00
Loktanella	12.504	-0.474	0.537	9.762e-01
Cryobacterium	0.921	-0.468	4.997	1.000e+00
Aaosphaeria	0.697	-0.463	5.490	1.000e+00
Nitrospira	0.933	-0.461	4.766	1.000e+00
Psychromicrobium	11.336	-0.458	0.604	6.405e-01
Lentzea	21.126	-0.454	0.598	4.872e-01
Bavariicoccus	60.548	-0.454	0.604	1.758e-01
Methylorubrum	265.529	-0.453	0.423	6.717e-02
Polaromonas	23.598	-0.451	0.606	9.153e-01
Emiliania	0.907	-0.419	2.635	1.000e+00
Proteus_phage_VB_PmiS-Isfahan_virus	1.743	-0.419	1.118	3.994e-01
Ruoffia	0.404	-0.414	5.396	NA
Zymoseptoria	45.638	-0.413	0.759	1.971e-02

Laspinema	0.879	-0.413	4.602	1.000e+00
Hankyongella	1.344	-0.407	5.266	1.000e+00
Baekduia	2.199	-0.405	2.386	1.000e+00
Carbonactinospora	0.255	-0.405	5.789	NA
Zoogloeaceae_genus	0.238	-0.405	5.789	NA
Falseniella	0.389	-0.404	5.789	NA
Flagellatimonas	0.061	-0.401	5.789	NA
Niabella	0.539	-0.399	4.711	1.000e+00
Actinoalloteichus	0.689	-0.396	2.979	1.000e+00
Rhizobium	451.252	-0.391	0.308	1.622e-01
Mameliella	2.131	-0.389	2.258	3.703e-01
Rhodococcus	520.947	-0.375	0.389	8.417e-04
Cupriavidus	310.513	-0.366	0.403	1.000e+00
Thyridium	8.234	-0.360	2.179	5.722e-01
Grimontia	0.335	-0.356	1.736	NA
Oligella	1.158	-0.352	5.390	1.000e+00
Myroides	0.746	-0.351	5.729	1.000e+00
Moheibacter	0.373	-0.350	5.789	NA
Klugiella	0.607	-0.350	5.789	1.000e+00
Protochlamydia	0.417	-0.350	5.789	NA
Williamsia	113.724	-0.340	0.844	1.984e-02
Desemzia	16.346	-0.329	1.030	9.321e-01
Chryseobacterium	293.101	-0.329	0.409	8.129e-01
Acanthamoeba	49.409	-0.328	1.340	7.313e-01
Moniliophthora	4.949	-0.327	1.780	4.577e-02
Virgisporangium	0.184	-0.327	5.789	NA
Geminicoccus	65.368	-0.321	0.463	9.278e-01
Euzebya	1.314	-0.319	2.612	4.184e-01
Salegentibacter	0.779	-0.316	5.789	1.000e+00
Calidithermus	19.574	-0.303	1.083	1.000e+00
Azorhizobium	2.897	-0.303	1.968	1.000e+00
Beggiatoa	32.646	-0.298	0.463	6.991e-02
Peptostreptococcaceae_genus	4.076	-0.296	1.791	1.000e+00
Allorhizobium	0.157	-0.287	5.790	NA
Xanthomonas	75.563	-0.278	0.590	1.000e+00
Candidate	14.431	-0.275	0.710	7.990e-01
Winkia	10.745	-0.274	1.596	1.000e+00
Austwickia	3.652	-0.273	2.316	6.336e-01
Citromicrobium	6.385	-0.272	1.680	9.064e-01
Microvirgula	1.737	-0.271	3.758	1.000e+00
Polymorphum	0.458	-0.270	4.396	NA
Lysobacter	182.469	-0.267	0.429	1.000e+00
Eleftheria	9.525	-0.264	1.243	6.531e-01
Hammondia	7.312	-0.264	0.969	8.470e-01
Capsulimonas	0.344	-0.264	5.452	NA
Paraprevotella	1.819	-0.258	2.568	1.000e+00
Atopomonas	30.225	-0.256	0.570	1.000e+00
Leuconostoc	39.177	-0.254	0.745	1.502e-02
Proteobacteria	45.108	-0.253	1.111	1.128e-10
Roseisolibacter	3.503	-0.252	2.212	7.829e-01
Piscicoccus	2.025	-0.252	3.037	8.667e-01
Hydrotalea	0.322	-0.248	5.790	NA
Ethanoligenens	1.222	-0.247	5.527	1.000e+00
Thioalkalivibrio	1.217	-0.247	2.829	9.966e-01
Marinomonas	19.694	-0.245	1.955	1.577e-01
Sandarakinorhabdus	1.376	-0.242	4.394	1.000e+00
Salicibibacter	0.239	-0.234	5.790	NA
Rhodoferax	44.739	-0.228	0.616	2.326e-01
Fredinandcohnia	6.991	-0.228	1.203	9.966e-01

Phascolarctobacterium	0.268	-0.224	5.790	NA
Halomicroarcula	1.134	-0.222	2.061	3.098e-01
Rhodospirillaceae_genus	1.528	-0.216	2.787	9.446e-01
Ottowia	13.988	-0.213	0.979	1.000e+00
Paucibacter	116.341	-0.212	0.435	4.068e-02
Carideicomes	0.567	-0.209	2.782	1.000e+00
Streptomyces	1952.590	-0.206	0.288	4.661e-01
Phytoactinopolyspora	0.463	-0.206	5.790	NA
Cutibacterium	357.153	-0.205	0.573	9.506e-03
Nitratireductor	1.522	-0.201	2.161	1.000e+00
Pseudoclavibacter	10.937	-0.200	1.248	1.000e+00
Nakamurella	30.160	-0.198	1.018	2.672e-01
Filobasidium	18.977	-0.194	1.049	8.525e-01
Trichococcus	0.181	-0.189	5.791	NA
Xanthobacter	8.431	-0.188	1.366	3.721e-01
Aplosporella	8.752	-0.187	1.518	7.375e-01
Brochothrix	2.027	-0.187	2.806	9.866e-01
Tetragenococcus	9.450	-0.186	1.735	9.029e-01
Penicillium	43.107	-0.184	0.677	5.812e-02
Planctomycetes	0.410	-0.182	5.343	NA
Methylocella	3.834	-0.177	2.525	8.801e-01
Hyphomicrobium	34.815	-0.176	0.579	6.943e-01
Rothia	222.042	-0.174	0.456	1.076e-01
Syntrophomonas	0.141	-0.171	5.791	NA
Gemmatirosa	2.401	-0.161	3.430	1.000e+00
Eubacterium	18.755	-0.158	0.814	1.000e+00
Peribacillus	4.798	-0.158	1.893	1.000e+00
Verrucosispora	2.100	-0.157	1.901	6.254e-01
Legionella	1332.136	-0.156	0.455	1.554e-01
Buchnera	70221.134	-0.152	0.362	5.494e-01
Geobacillus	32.342	-0.149	1.136	1.284e-03
Neglectibacter	0.247	-0.148	3.332	NA
Varibaculum	3.474	-0.146	2.076	1.000e+00
Methyloglobulus	0.746	-0.144	3.053	1.000e+00
Miniimonas	0.445	-0.139	5.791	NA
Komagataeibacter	1.405	-0.138	3.354	1.000e+00
Porphyromonas	64.376	-0.138	0.753	2.073e-01
Parasaccharibacter	82.051	-0.131	0.393	4.165e-03
Neomicrococcus	8.418	-0.117	1.469	4.661e-01
Paeniglutamicibacter	22.306	-0.115	0.779	1.867e-03
Mesorhizobium	79.330	-0.114	0.367	1.357e-01
Pleurotus	1.535	-0.110	2.248	9.849e-01
Ornithinimicrobium	139.539	-0.107	0.805	2.209e-05
Salipiger	55.657	-0.107	0.553	5.602e-02
Pirellulimonas	0.144	-0.104	5.791	NA
Allofustis	0.129	-0.104	5.791	NA
Jeotgalicoccus	20.422	-0.102	0.744	8.804e-01
Kushneria	78.067	-0.088	0.513	3.646e-01
Paenibacillus	158.355	-0.088	0.360	5.910e-01
Aequitasia	117.988	-0.087	1.687	4.432e-06
Pseudoalteromonas	365.269	-0.086	0.397	3.465e-01
Thalassolituus	1.194	-0.081	2.258	8.751e-01
Pseudoflavonifractor	1.639	-0.080	2.099	8.691e-01
Leucobacter	21.363	-0.080	0.816	1.000e+00
Suillus	38.317	-0.080	1.769	2.190e-02
Isoalcanivorax	1.062	-0.079	4.306	1.000e+00
Myxococcus	31.942	-0.079	0.520	3.922e-01
Trypanosoma	18.124	-0.078	1.141	3.595e-01
Trabulsiella	0.462	-0.064	1.254	NA

Acidiplasma	17.926	-0.060	0.657	2.414e-01
Alteribacter	6.236	-0.058	0.835	9.679e-01
Plantactinospora	1.201	-0.057	2.666	9.917e-01
Bifidobacteriaceae_genus	10.863	-0.054	1.729	6.329e-02
Mangrovibacter	0.437	-0.052	1.531	NA
Actinomyces	520.914	-0.050	0.468	4.707e-01
Rubrobacter	26.883	-0.048	1.330	7.138e-02
Turicibacter	6.744	-0.043	2.503	1.523e-01
Exiguobacterium	51.512	-0.041	0.663	3.652e-01
Methylovorus	3.354	-0.040	1.631	5.493e-01
Sphingobium	158.875	-0.031	0.384	7.263e-01
Haemophilus	217.469	-0.029	0.518	6.426e-02
Lujinxingia	0.358	-0.029	4.183	NA
Agreia	1.710	-0.027	4.227	9.966e-01
Carnobacterium	19.889	-0.026	0.969	9.292e-01
Caldilinea	2.122	-0.022	3.452	8.917e-01
Allobacillus	31.886	-0.022	0.733	1.000e+00
Histoplasma	0.752	-0.021	2.723	1.000e+00
Sphaerulina	10.884	-0.018	1.311	4.661e-01
Methyloversatilis	103.042	-0.015	0.556	1.000e+00
Mediterraneibacter	9.994	-0.009	0.856	8.889e-01
Actinomadura	901.640	-0.007	0.300	9.966e-01
Mikella	0.000	0.000	0.000	NA
Ishikawaella	0.000	0.000	0.000	NA
Hafniaceae_genus	0.000	0.000	0.000	NA
Shigella_phage_SfIV_virus	0.000	0.000	0.000	NA
Mediannikoviicoccus	0.000	0.000	0.000	NA
Escherichia_phage_500465-1_virus	0.000	0.000	0.000	NA
Enterobacteria_phage_DE3_virus	0.000	0.000	0.000	NA
Enterobacteria_phage_P7_virus	0.000	0.000	0.000	NA
Escherichia_phage_RCS47_virus	0.000	0.000	0.000	NA
Lagierella	0.000	0.000	0.000	NA
Rhabdobacter	0.000	0.000	0.000	NA
Kallipyga	0.000	0.000	0.000	NA
Escherichia_phage_500465-2_virus	0.000	0.000	0.000	NA
Escherichia_virus_Lambda_2G7b	0.000	0.000	0.000	NA
Escherichia_phage_TL-2011b_virus	0.000	0.000	0.000	NA
Escherichia_virus_Lambda_4A7	0.000	0.000	0.000	NA
Pusillibacter	0.000	0.000	0.000	NA
Rectinema	0.000	0.000	0.000	NA
Escherichia_phage_Lambda_ev099_virus	0.000	0.000	0.000	NA
Klebsiella_phage_4_virus	0.000	0.000	0.000	NA
Stx2-converting_phage_1717_virus	0.000	0.000	0.000	NA
Cetobacterium	0.000	0.000	0.000	NA
Escherichia_phage_Cartapus_virus	0.000	0.000	0.000	NA
SsRNA_phage_SRR5466337_3_virus	0.000	0.000	0.000	NA
Escherichia_phage_Lambda_ev207_virus	0.000	0.000	0.000	NA
Escherichia_virus_Lambda_1H12	0.000	0.000	0.000	NA
Tropicibacter	0.000	0.000	0.000	NA
Stx2-converting_phage_Stx2a_WGPS2_virus	0.000	0.000	0.000	NA
Escherichia_phage_Lambda_ev243_virus	0.000	0.000	0.000	NA
Couchioplanes	0.000	0.000	0.000	NA
Escherichia_virus_Lambda_2H10	0.000	0.000	0.000	NA
Deferrisoma	0.000	0.000	0.000	NA
Escherichia_phage_D6_virus	0.000	0.000	0.000	NA
Enterobacteria_phage_Sf6_virus	0.000	0.000	0.000	NA
Gloeothece	0.000	0.000	0.000	NA
Escherichia_phage_520873_virus	0.000	0.000	0.000	NA
Viadribacter	0.000	0.000	0.000	NA

Oceanotoga	0.000	0.000	0.000	NA
Salmonella_phage_SJ46_virus	0.000	0.000	0.000	NA
Paludicola	0.000	0.000	0.000	NA
Kaustia	0.000	0.000	0.000	NA
Goekera	0.470	0.006	5.792	1.000e+00
Gemmobacter	16.490	0.015	1.305	4.728e-01
Roseicella	5.364	0.015	1.982	7.829e-01
Tamlana	6.081	0.021	0.885	2.430e-01
Megamonas	3.586	0.021	2.183	1.000e+00
Sporolactobacillus	110.272	0.025	0.325	1.000e+00
Alishewanella	16.028	0.027	1.296	6.749e-01
Alternaria	98.393	0.028	0.457	9.953e-01
Desulfoscipio	0.435	0.029	5.793	NA
Papillibacter	0.041	0.029	5.793	NA
Inquilinus	0.544	0.033	3.468	1.000e+00
Halalkalibacter	112.475	0.037	0.606	1.000e+00
Taibaiella	8.236	0.039	1.468	6.774e-01
Xanthocytophaga	2.018	0.041	2.447	4.334e-01
Plesiocystis	0.041	0.041	5.793	NA
Methylotenera	11.956	0.050	1.305	1.000e+00
Acidovorax	744.728	0.061	0.211	4.008e-01
Coccidioides	0.857	0.062	2.660	1.000e+00
Blochmannia	16.307	0.064	1.409	7.914e-01
Flectobacillus	1.051	0.076	3.041	1.000e+00
Microsporum	6.726	0.080	1.830	1.729e-02
Simonsiella	0.622	0.081	5.640	1.000e+00
Fomitiporia	6.233	0.084	1.508	6.902e-01
Frisingicoccus	0.052	0.091	5.793	NA
Parapusillimonas	0.200	0.093	5.793	NA
Dolosigranulum	32.224	0.094	1.410	7.391e-04
Wielerella	1.083	0.097	4.551	1.000e+00
Arthrobacter	653.862	0.101	0.426	4.577e-02
Arsenophonus	1.327	0.101	1.272	1.333e-02
Psychrosphaera	4.364	0.102	1.233	7.829e-01
Cystobacter	0.999	0.108	4.133	1.000e+00
Falsiroseomonas	137.429	0.108	0.507	1.000e+00
Cucurbitaria	0.669	0.117	3.705	1.000e+00
Caulobacteraceae_genus	0.901	0.121	5.003	1.000e+00
Angustibacter	5.041	0.128	2.346	8.737e-01
Shinella	19.684	0.133	1.014	6.991e-02
Pasteurellaceae_genus	2.823	0.138	1.862	8.886e-01
Ideonella	12.997	0.140	1.089	5.170e-01
Yinghuangia	0.394	0.147	3.891	NA
Sinorhizobium	5.189	0.148	1.622	1.000e+00
Lignipirellula	0.509	0.149	5.770	1.000e+00
Pseudorivibacter	2.168	0.151	2.713	6.307e-01
Hubei_permutotetra-like_virus	2.130	0.157	3.949	9.966e-01
Aquimonas	1.574	0.159	4.835	1.000e+00
Ectobacillus	119.344	0.159	0.684	2.354e-01
Tenebrionibacter	0.227	0.161	2.154	NA
Pseudaestuariivita	0.239	0.162	5.793	NA
Hydrogenophilus	2.147	0.168	3.468	1.000e+00
Garicola	5.439	0.171	2.948	8.482e-01
Hypoxylon	1.453	0.172	3.211	1.000e+00
Halomonas	158.342	0.173	0.510	2.426e-01
Fervidibacillus	2.133	0.175	4.561	1.000e+00
Aliihoeflea	3.673	0.177	2.931	1.000e+00
Cohnella	3.253	0.179	1.447	1.000e+00
Acidipropionibacterium	23.248	0.181	1.160	5.718e-01

Geomicrobium	0.127	0.186	5.786	NA
Frischella	1.199	0.186	1.969	1.000e+00
Runella	0.324	0.187	5.793	NA
Labilibaculum	7.087	0.187	0.986	5.726e-01
Kinneretia	11.622	0.188	1.028	1.000e+00
Capnocytophaga	41.728	0.195	0.611	1.000e+00
Skermanella	35.925	0.202	1.169	1.325e-02
Haloferax	0.578	0.203	5.673	1.000e+00
Lactobacillus	101.572	0.210	0.375	4.221e-02
Methylibium	9.563	0.211	1.675	9.908e-01
Aciditerrimonas	0.325	0.213	4.781	NA
Leucothrix	10.440	0.215	0.966	9.966e-01
Arcticibacter	0.589	0.218	3.331	1.000e+00
Dubosiella	0.074	0.220	5.794	NA
Parabacteroides	28.004	0.228	0.687	1.000e+00
Ralstonia	14254.930	0.229	0.322	9.966e-01
Limosilactobacillus	14.701	0.229	0.857	4.096e-01
Bacteroidales	0.451	0.232	4.625	NA
Porphyrobacter	4.254	0.235	1.270	1.000e+00
Sandaracinobacter	0.244	0.241	5.794	NA
Ilyonectria	2.369	0.244	2.307	9.711e-01
Chromatium	57.450	0.249	0.403	2.932e-01
Ancylomarina	7.570	0.251	1.125	9.974e-01
Branchiibius	0.578	0.256	5.770	1.000e+00
Streptoalloteichus	1.391	0.256	2.820	9.507e-01
Arenimonas	1.463	0.258	2.957	1.000e+00
Sphingomonas	2004.426	0.262	0.256	7.876e-01
Entamoeba	32.922	0.263	0.870	1.046e-03
Kluyvera	247.288	0.266	0.366	4.658e-01
Superficieibacter	0.163	0.277	2.671	NA
Actinomycetospora	14.912	0.278	0.966	6.333e-01
Achromobacter	178.806	0.278	0.262	1.000e+00
Lactovum	3.125	0.296	2.276	1.000e+00
Curvibacter	191.180	0.297	0.285	3.319e-01
Humibacter	0.118	0.299	5.794	NA
Mobilicoccus	15.933	0.301	1.142	1.000e+00
Lederbergia	0.161	0.301	5.794	NA
Pinisolibacter	11.572	0.301	0.554	6.856e-01
Paramesorhizobium	0.152	0.301	5.794	NA
Propionibacterium_phage_PHL041M10_virus	0.037	0.301	5.794	NA
Sulfuritalea	0.033	0.301	5.794	NA
Dongshaea	0.131	0.305	5.794	NA
Endocarpon	10.892	0.311	1.352	1.692e-02
Pectobacterium_phage_CBB_virus	0.902	0.315	3.564	1.000e+00
Winogradskyella	5.235	0.316	0.730	1.959e-01
Marinithermofilum	29.006	0.317	0.526	9.279e-01
Plastoroseomonas	0.749	0.322	5.045	1.000e+00
Gryllotalpicola	3.043	0.325	2.068	7.085e-01
Rhodospirillales	0.991	0.326	3.671	9.762e-01
Azohydromonas	22.510	0.337	1.039	2.411e-01
Pneumocystis	8.386	0.338	2.374	6.333e-01
Kaistella	17.043	0.342	0.964	1.000e+00
Asticcacaulis	114.652	0.342	0.805	9.966e-01
Pirellula	0.574	0.346	5.137	1.000e+00
Rhizorhabdus	68.045	0.346	0.575	5.205e-01
Algibacter	5.117	0.347	1.124	8.905e-01
Acidobacteriaceae_genus	0.311	0.347	5.794	NA
Kribbella	9.265	0.350	1.169	7.026e-02
Phaeovulum	30.793	0.356	0.623	9.711e-01

Salinisphaera	0.371	0.358	2.159	NA
Fuscibacter	0.854	0.358	5.351	1.000e+00
Lacibacter	0.974	0.360	5.794	1.000e+00
Propionibacterium	5198.439	0.372	0.518	2.398e-03
Pelagibacterium	4.466	0.378	2.242	8.886e-01
Azovibrio	1.084	0.379	3.337	1.000e+00
Thiothrix	0.405	0.380	5.770	NA
Devosia	86.884	0.380	0.716	2.169e-01
Parasphingopyxis	20.694	0.381	1.335	7.054e-01
Xenophilus	37.016	0.383	0.651	9.304e-01
Sutterella	3.974	0.386	1.270	1.000e+00
Vogesella	4.571	0.391	1.896	1.000e+00
Brucella	13.265	0.393	0.859	9.138e-01
Thermosipho	4.351	0.397	0.884	4.872e-01
Curtobacterium	171.096	0.397	0.712	7.488e-03
Nocardioides	882.762	0.398	0.396	3.767e-05
Arsenicicoccus	2.165	0.402	2.041	8.886e-01
Aphanothece	13.799	0.405	0.858	8.727e-01
Corallococcus	181.760	0.405	0.594	1.333e-06
Synechocystis	12.589	0.414	1.252	9.446e-01
Parafrankia	2.734	0.416	2.162	5.708e-01
Comamonadaceae_genus	99.630	0.419	0.466	6.267e-01
Pseudenterobacter	0.693	0.425	1.235	9.134e-02
Pseudoduganella	10.110	0.428	1.522	1.833e-01
Fluoribacter	203.940	0.429	0.542	8.905e-01
Paraclostridium	28.339	0.430	1.463	1.096e-03
Thalassotalea	1.950	0.440	1.945	5.736e-01
Wenxinia	0.272	0.444	5.452	NA
Primorskyibacter	0.613	0.445	1.925	8.516e-01
Streptosporangium	49.775	0.450	0.436	4.243e-03
Besnoitia	7.844	0.451	0.867	1.000e+00
Pectobacterium	3.690	0.451	1.120	4.000e-02
Oryzibacter	0.481	0.454	5.768	1.000e+00
Salinarimonas	4.201	0.454	0.700	2.354e-01
Rhodobacteraceae_genus	44.452	0.455	0.851	6.054e-04
Planobispora	0.083	0.457	5.795	NA
Phialophora	3.186	0.462	1.692	1.577e-01
Xylanimonas	2.036	0.464	1.951	1.000e+00
Miniphocaeibacter	0.144	0.469	5.795	NA
Cokeromyces	2.224	0.471	1.001	4.421e-01
Weissella	7.985	0.479	1.566	2.672e-01
Aliidongia	0.963	0.479	3.574	1.000e+00
Toxoplasma	250.863	0.485	0.271	1.374e-03
Thauera	36.207	0.486	0.463	4.873e-01
Siphonobacter	0.858	0.490	5.795	1.000e+00
Batrachochytrium	121.131	0.503	3.053	4.604e-02
Melaminivora	1.423	0.509	3.223	1.000e+00
Didymosphaeria	48.534	0.515	1.260	2.744e-01
Nodosilinea	25.845	0.521	1.453	8.873e-02
Thiohalocapsa	35.128	0.524	0.710	7.773e-01
Psychrobacter	113.554	0.525	0.558	6.786e-02
Blastococcus	150.500	0.530	0.716	1.176e-02
Myxococcales	1.400	0.531	4.796	1.000e+00
Neorhizobium	3.954	0.534	2.067	1.000e+00
Paenarthrobacter	0.590	0.546	5.745	1.000e+00
Thermococcus	14.111	0.547	0.818	7.054e-01
Deinococcus	166.493	0.547	0.290	6.426e-02
Niveispirillum	1.317	0.550	3.489	1.000e+00
Minwuia	0.824	0.560	5.795	1.000e+00

Tissierella	290.882	0.564	0.364	2.244e-01
Miltoncostaea	3.182	0.565	3.075	1.000e+00
Barnesiella	9.605	0.571	0.746	1.000e+00
Schneideria	0.970	0.572	3.414	1.000e+00
Slackia	0.631	0.573	4.740	1.000e+00
Firmicutes	2.935	0.574	2.448	6.109e-01
Ramlibacter	69.721	0.576	0.726	2.073e-01
Plasmodium	260.330	0.580	0.313	5.015e-02
Conexibacter	10.106	0.585	1.375	9.974e-01
Acidisoma	1.128	0.586	3.690	1.000e+00
Pigmentiphaga	5.865	0.587	1.487	1.000e+00
Methylocaldum	0.238	0.590	4.853	NA
Lamprobacter	0.388	0.590	3.024	NA
Actinobaculum	7.638	0.594	1.719	1.000e+00
Olsenella	7.411	0.597	1.628	1.000e+00
Parapedobacter	0.896	0.602	2.781	9.321e-01
Pedococcus	6.046	0.604	1.462	1.000e+00
Renibacterium	7.693	0.605	1.627	1.000e+00
Niastella	47.268	0.608	2.189	5.091e-02
Rickettsiella	1.563	0.615	1.636	9.639e-01
Propionibacteriaceae_genus	1.387	0.620	3.016	1.000e+00
Stenotrophomonas	1057.148	0.628	0.183	8.242e-08
Rehaibacterium	0.635	0.635	5.591	1.000e+00
Aridibaculum	0.190	0.635	5.795	NA
Teredinibacter	0.038	0.635	5.795	NA
Morganella	1.022	0.643	1.250	5.093e-02
Aphanizomenon	21.520	0.647	0.514	5.757e-01
Pyrococcus	12.784	0.648	1.162	1.000e+00
Adhaeribacter	19.641	0.648	2.121	8.219e-03
Blautia	26.548	0.655	1.131	1.473e-02
Marmoricola	34.294	0.655	1.044	2.588e-01
Blattabacterium	0.399	0.657	5.795	NA
Aeromicrobium	61.992	0.659	0.773	2.461e-05
Brenneria	3.075	0.668	0.846	1.000e+00
Pseudescherichia	0.062	0.674	5.796	NA
Terribacillus	0.063	0.674	5.796	NA
Roseateles	324.440	0.674	0.322	3.755e-02
Neobacillus	54.975	0.675	0.547	2.707e-02
Pseudolabrys	0.699	0.679	3.688	1.000e+00
Betaproteobacterium_JGI	8.316	0.679	0.801	1.901e-01
Zobellella	1.165	0.679	4.043	1.000e+00
Falcatimonas	0.017	0.680	5.796	NA
Bacterium	36.502	0.682	0.440	2.114e-02
Plautia	0.323	0.689	3.793	NA
Oceanibium	5.401	0.689	1.297	1.813e-01
Dyadobacter	35.160	0.690	1.031	2.404e-04
Aequorivita	205.552	0.690	0.419	1.483e-01
Pseudorhodoferax	8.912	0.694	1.320	1.000e+00
Coprococcus	2.444	0.696	2.993	1.000e+00
Nocardia	68.412	0.700	0.434	1.471e-01
Epilithonimonas	41.936	0.703	0.670	1.398e-02
Mycobacteriaceae_genus	41.727	0.707	0.443	4.420e-01
Alpha	67.476	0.707	0.566	9.076e-01
Pseudonocardia	81.981	0.707	0.716	1.059e-02
Armatimonas	0.268	0.718	5.796	NA
Picosynechococcus	10.813	0.718	1.300	2.240e-02
Guillardia	48.159	0.719	1.576	2.989e-08
Pyricularia	0.300	0.730	5.794	NA
Dothidotthia	2.037	0.732	2.213	1.833e-01

Sulfitobacter	6.435	0.732	0.906	6.900e-01
Xylophilus	6.954	0.732	1.010	4.889e-01
Actinotalea	13.336	0.734	1.407	1.000e+00
Leclercia	58.127	0.734	0.514	2.881e-02
Jeotgalibaca	1.008	0.738	4.496	1.000e+00
Actirhodobacter	0.195	0.741	5.794	NA
Brachymonas	13.279	0.743	0.880	8.469e-01
Gordonia	106.567	0.744	0.710	3.547e-07
Prosthecobacter	3.876	0.744	2.127	1.000e+00
Rhodomicrobium	7.805	0.744	0.915	7.036e-01
Thermomonas	34.874	0.749	0.508	2.946e-01
Solemya	6.351	0.751	1.373	1.000e+00
Solihabitans	25.142	0.752	0.605	2.406e-01
Tepidicella	11.802	0.752	1.429	5.720e-01
Hoeflea	3.595	0.758	1.948	9.820e-01
Pandoraea	33.582	0.765	0.632	1.000e+00
Fischerella	0.349	0.767	5.796	NA
Bergeyella	0.183	0.767	5.796	NA
Schizophyllum	63.317	0.768	1.659	1.368e-01
Romboutsia	3.447	0.770	1.496	9.962e-01
Pseudoglutamicibacter	1.363	0.772	2.738	9.966e-01
Pisolithus	0.864	0.774	1.907	1.000e+00
Methylosarcina	17.576	0.774	1.126	6.815e-01
Pedomonas	5.502	0.777	2.103	5.967e-01
Limimaricola	3.153	0.778	2.677	5.581e-01
Mitsuokella	0.467	0.782	5.796	1.000e+00
Mongoliimonas	0.486	0.782	5.796	1.000e+00
Isosphaera	0.330	0.782	5.796	NA
Enteractinococcus	0.398	0.782	5.796	NA
Rhizobiaceae_genus	0.018	0.782	5.796	NA
Propylenella	0.014	0.782	5.796	NA
Terrabacter	109.045	0.786	0.454	3.165e-01
Paracoccus	735.536	0.792	0.307	1.077e-03
Bacteria	0.118	0.796	2.281	NA
Rhodoligotrophos	1.096	0.796	3.876	1.000e+00
Dacryopinax	1.891	0.799	2.903	7.876e-01
Jannaschia	2.987	0.800	0.948	4.297e-01
Trichosporon	145.713	0.802	1.256	4.086e-06
Acidiferrimicrobium	0.300	0.812	4.769	NA
Centipeda	0.032	0.813	5.796	NA
Acidiphilium	0.973	0.813	3.353	1.000e+00
Fonsecaea	35.534	0.814	1.089	3.383e-08
Magnetospirillum	1.981	0.818	1.934	8.801e-01
Bathymodiolus	0.021	0.825	5.796	NA
Caldovatus	0.480	0.826	4.200	1.000e+00
Prolinoborus	3.096	0.829	1.716	1.000e+00
Citricoccus	39.723	0.844	1.021	6.422e-02
Nocardiopsis	8.273	0.853	2.000	1.000e+00
Pyruvatibacter	1.139	0.860	1.522	8.341e-01
Caballeronia	39.742	0.867	0.427	1.532e-02
Lewinella	0.693	0.868	1.262	5.591e-01
Cryptomonas	1.703	0.872	2.687	8.889e-01
Haliea	0.489	0.872	3.246	1.000e+00
Aggregatilinea	0.100	0.872	5.796	NA
Solirubrobacter	18.530	0.878	1.108	1.000e+00
Gardnerella	54.022	0.879	1.211	2.398e-03
Sporichthya	7.913	0.880	2.206	7.416e-01
Siccibacter	0.129	0.881	5.796	NA
Tautonia	1.472	0.890	3.632	1.000e+00

Azonexus	3.481	0.897	2.124	5.970e-01
Gilbertella	13763.122	0.897	0.433	1.473e-02
Alcanivorax	267.141	0.898	0.441	1.834e-01
Nannizzia	1.520	0.904	2.759	1.000e+00
Pseudanabaena	0.388	0.909	5.797	NA
Rhabdothermincola	15.023	0.913	1.851	7.314e-01
Bosea	160.841	0.919	0.358	8.204e-02
Annandia	0.084	0.922	5.797	NA
Cellulomonas	41.380	0.926	0.663	6.651e-01
Mycetohabitans	9.753	0.931	0.805	2.173e-03
Marinifilum	38.998	0.939	0.484	3.823e-02
Mangrovibacillus	3.321	0.946	2.539	1.000e+00
Oceaniovalibus	4.279	0.946	1.428	9.011e-01
Protaetiibacter	2.115	0.952	2.578	1.000e+00
Aestuariimicrobium	3.255	0.954	2.056	1.000e+00
Linderina	1.155	0.964	2.270	9.762e-01
Roseococcus	1.486	0.967	2.985	1.000e+00
Thiobacillus	5.354	0.977	1.522	5.997e-01
Massilia	397.055	0.980	0.485	5.726e-07
Lancefieldella	4.026	0.996	2.720	1.000e+00
Tardiphaga	3.851	1.002	1.497	5.147e-01
Methylovulum	39.553	1.002	0.908	1.996e-01
Methylophaga	1.029	1.003	2.580	1.000e+00
Calothrix	2.801	1.005	1.869	9.711e-01
Desulforhabdus	2.687	1.006	2.267	1.000e+00
Thermothelomyces	4.242	1.006	2.892	6.572e-02
Pyrenophora	3.967	1.013	1.632	5.813e-01
Paracnuella	0.644	1.015	2.806	9.606e-01
Atlantibacter	14.246	1.015	0.525	5.549e-01
Citreicoccus	0.220	1.016	5.797	NA
Nanogingivalis	0.227	1.018	4.891	NA
Leifsonia	76.614	1.021	0.711	2.707e-02
Caldicellulosiruptor	0.237	1.022	5.797	NA
Thermobacillus	0.214	1.022	5.797	NA
Variibacter	0.081	1.022	5.797	NA
Umbelopsis	0.027	1.022	5.797	NA
Tepidimonas	33.038	1.023	0.914	2.326e-01
Gilliamella	0.563	1.024	2.716	1.000e+00
Natronorubrum	1.146	1.028	3.104	7.529e-01
Macrococcus	3.332	1.028	1.469	1.000e+00
Acidothermus	3.350	1.029	2.938	3.821e-01
Franconibacter	0.453	1.029	2.908	NA
Flaviflexus	0.626	1.029	4.120	1.000e+00
Flavisolibacter	0.705	1.030	2.768	1.000e+00
Marasmius	2.889	1.030	2.158	1.000e+00
Roseibaca	0.208	1.032	5.797	NA
Lonsdalea	0.189	1.036	2.193	NA
Acuticoccus	38.672	1.050	0.483	2.354e-01
Wigglesworthia	1.349	1.052	1.810	1.000e+00
Coleofasciculus	1.102	1.058	2.824	1.000e+00
Ruegeria	9.018	1.059	1.355	3.922e-01
Pseudopropionibacterium	6.216	1.063	1.418	1.000e+00
Haloechinothrix	0.329	1.066	5.797	NA
Berkiella	0.057	1.066	5.797	NA
Usitatibacter	0.008	1.066	5.797	NA
Burkholderiales	19.138	1.077	0.812	9.309e-02
Microvirga	80.646	1.081	0.554	6.965e-04
Lactiplantibacillus	3.228	1.086	1.070	5.607e-01
Coniophora	16.488	1.091	2.095	6.097e-01

Crocosphaera	13.832	1.091	0.798	1.000e+00
Pleomorpha	4.781	1.114	1.890	8.141e-01
Anaeromyxobacter	2.178	1.126	2.686	8.886e-01
Zhihengliuella	3.336	1.127	2.763	4.732e-01
Moraxellaceae_genus	14.760	1.131	1.080	2.977e-01
Human_endogenous_retrovirus	5.191	1.132	1.041	2.285e-01
Peptoclostridium	0.768	1.133	4.568	1.000e+00
Scytonema	22.749	1.139	0.940	5.410e-01
Pararhodobacter	7.548	1.139	1.541	5.093e-02
Cellvibrio	9.111	1.142	1.528	7.741e-01
Paracaedibacter	1.572	1.143	3.729	1.000e+00
Butyricicoccus	0.747	1.144	4.002	1.000e+00
Methylobrevis	19.272	1.145	0.811	5.099e-01
Acinetobacter	11818.492	1.145	0.225	3.006e-14
Ectothiorhodospira	16.455	1.149	0.727	1.000e+00
Pseudomicrostroma	29.079	1.156	1.472	1.163e-03
Altererythrobacter	7.197	1.156	1.930	6.889e-01
Croceibacterium	1.140	1.156	2.688	9.946e-01
Isoptericola	47.485	1.157	0.567	1.473e-08
Ciceribacter	3.272	1.157	1.646	1.000e+00
Fusicatenibacter	1.772	1.158	3.159	1.000e+00
Paecilomyces	5.744	1.159	1.867	8.790e-01
Anaerostipes	0.192	1.163	4.988	NA
Arachidicoccus	0.202	1.163	5.798	NA
Alistipes	0.973	1.180	3.805	1.000e+00
Archangium	2.621	1.190	1.587	7.914e-01
Sinomonas	1.516	1.191	5.798	1.000e+00
Dermacoccus	44.902	1.197	0.536	1.728e-04
Mammaliicoccus	12.984	1.203	1.416	2.855e-01
Methylomicrobium	16.552	1.206	1.104	6.146e-01
Pleurocapsa	1.217	1.216	3.827	1.000e+00
Cryptosporangium	3.758	1.222	2.661	8.889e-01
Kineococcus	53.880	1.224	1.060	2.101e-09
Phyllobacterium	39.527	1.225	1.001	5.602e-02
Viridibacillus	0.463	1.228	5.208	NA
Roseomonas	144.562	1.230	0.671	2.108e-06
Coniosporium	2.486	1.243	2.861	5.997e-01
Uncultured	130.743	1.243	0.600	3.616e-08
Rubellimicrobium	30.281	1.249	0.998	1.818e-02
Rhizobiales	18.512	1.250	0.909	8.801e-01
Burkholderia	2610.408	1.256	0.204	1.287e-20
Hymenobacter	123.295	1.259	0.586	1.033e-04
Knoellia	9.989	1.260	1.421	1.000e+00
Coprinopsis	1.019	1.264	3.509	1.000e+00
Campylobacter	36.232	1.270	0.522	4.582e-02
Snodgrassella	4.153	1.270	2.529	1.000e+00
Clostridiales	35.068	1.283	0.687	5.803e-04
Robbsia	0.852	1.287	2.984	1.000e+00
Ligilactobacillus	18.282	1.291	1.291	2.883e-01
Saccharomyces	1.294	1.295	3.628	9.966e-01
Drechmeria	0.756	1.297	3.445	1.000e+00
UNVERIFIED_ORG:	13.458	1.304	0.971	1.000e+00
Ruminococcaceae_genus	14.329	1.306	0.737	6.307e-01
Paraglaciecola	2.599	1.319	1.328	1.000e+00
Pannonibacter	1.432	1.321	3.211	1.000e+00
Gregarina	0.881	1.324	5.798	1.000e+00
Phytohabitans	0.770	1.324	5.798	1.000e+00
Luteipulveratus	0.361	1.324	5.798	NA
Kordiimonas	0.361	1.324	5.798	NA

Ferribacterium	0.267	1.324	5.798	NA
Thermorudis	0.237	1.324	5.798	NA
Quatrionicoccus	0.245	1.324	5.798	NA
Chlamydia	0.204	1.324	5.798	NA
Moranella	0.333	1.324	5.798	NA
Atlanticothrix	0.147	1.324	5.798	NA
Neoroseomonas	0.161	1.324	5.798	NA
Acidiluteibacter	0.200	1.324	5.798	NA
Anaeromassilibacillus	0.075	1.324	5.798	NA
Type-D_symbiont_of_Plautia_stali	0.011	1.324	5.798	NA
Enterobacteria_phage_f1_virus	0.008	1.324	5.798	NA
Photodesmus	0.007	1.324	5.798	NA
Doolittlea	0.005	1.324	5.798	NA
Luteococcus	1.151	1.325	4.530	1.000e+00
Xylaria	0.460	1.325	4.551	NA
Sphingosinithalassobacter	0.325	1.325	4.308	NA
Dongia	0.909	1.325	3.895	1.000e+00
Croceicoccus	0.549	1.326	3.470	1.000e+00
Paraburkholderia	685.653	1.328	0.225	1.969e-17
Sodalis-like	0.331	1.330	2.281	NA
Promicromonospora	0.868	1.334	4.107	1.000e+00
Erythrobacter	135.550	1.342	0.430	8.150e-02
Mobiluncus	3.137	1.342	2.228	1.000e+00
Parvularcula	2.074	1.344	3.156	1.000e+00
Saccharomonospora	2.321	1.345	2.034	1.000e+00
Dechloromonas	8.894	1.358	1.441	6.146e-01
Alicyclobacillus	0.391	1.368	5.795	NA
Candida	5.852	1.371	1.213	4.619e-01
Serpentinimonas	0.382	1.373	5.798	NA
Cecembia	0.302	1.373	5.798	NA
Andreesenia	0.839	1.373	5.798	1.000e+00
Niallia	0.268	1.373	5.570	NA
Falsochrobactrum	0.395	1.373	5.798	NA
Sulfuricystis	0.204	1.373	5.798	NA
Propionibacterium_phage_P100D_virus	0.085	1.374	5.798	NA
Type-B_symbiont_of_Plautia_stali	0.070	1.374	5.798	NA
Pararobbsia	0.017	1.374	5.798	NA
Idiomarinaceae_genus	0.023	1.374	5.798	NA
Tessaracoccus	17.444	1.374	1.291	1.000e+00
Geodermatophilus	37.591	1.379	1.080	4.726e-01
Agrobacterium	192.449	1.397	0.297	1.182e-10
Mangrovicoccus	8.916	1.414	1.861	5.954e-01
Frigidibacter	67.900	1.415	0.527	4.333e-02
Spizellomyces	92.702	1.416	1.737	3.535e-07
Chitinophagaceae_genus	0.335	1.420	5.798	NA
Polysphondylium	0.471	1.420	5.798	1.000e+00
Youxingia	0.215	1.421	5.798	NA
Anaerotardibacter	0.125	1.421	5.798	NA
Type-F_symbiont_of_Plautia_stali	0.110	1.421	5.798	NA
Paraflavisolibacter	0.061	1.421	5.798	NA
Enterobacteria_phage_T7_virus	0.002	1.421	5.798	NA
Eimeria	1.388	1.424	2.077	5.291e-01
Aminobacter	1.243	1.438	1.999	9.440e-01
Sphingosinicella	13.654	1.454	1.637	1.945e-01
Actinoplanes	35.115	1.461	0.724	8.920e-01
Latilactobacillus	2.108	1.468	2.759	1.000e+00
Izhakiella	7.982	1.469	1.554	6.097e-01
Ruania	3.365	1.480	2.270	1.000e+00
Luteitalea	16.785	1.481	1.927	3.165e-01

Starkeya	1.085	1.486	2.705	9.966e-01
Agathobacter	7.239	1.488	2.070	1.000e+00
Nitrosomonas	5.354	1.515	1.557	7.160e-01
Flexivirga	2.311	1.523	2.242	8.889e-01
Oceanobacillus	36.670	1.525	0.547	2.181e-01
Algoriphagus	22.246	1.529	0.640	2.796e-01
Delftia	511.917	1.537	0.200	6.117e-51
Thiomonas	1.233	1.544	2.856	1.000e+00
Mycetocola	2.282	1.548	2.621	9.966e-01
Polynucleobacter	4.388	1.548	1.866	1.758e-01
Fimbriiglobus	3.126	1.572	2.603	1.000e+00
Vibrio	744.207	1.586	0.444	4.757e-07
Janthinobacterium	149.394	1.591	0.432	3.476e-04
Alsobacter	2.441	1.593	2.596	1.000e+00
Cronobacter	1.817	1.599	1.738	6.592e-03
Morchella	0.641	1.612	5.799	1.000e+00
Thermaerobacter	0.642	1.612	5.718	1.000e+00
Nocardioidaceae_genus	0.671	1.612	5.799	1.000e+00
Rugosimonospora	1.487	1.612	5.799	1.000e+00
Helcococcus	0.691	1.612	5.799	1.000e+00
Bryobacter	0.568	1.612	5.799	1.000e+00
Oligoflexus	0.666	1.612	5.799	1.000e+00
Hoaglandella	0.398	1.612	5.799	NA
Pseudofrankia	0.563	1.612	5.703	1.000e+00
Catellatospora	0.854	1.612	5.799	1.000e+00
Torulaspora	0.428	1.612	5.799	NA
Hoyosella	1.254	1.612	5.220	1.000e+00
Catenulispora	0.821	1.612	5.799	1.000e+00
Chloroflexus	0.309	1.612	5.799	NA
Wenjunlia	0.648	1.612	5.581	1.000e+00
Peptococcus	0.489	1.612	5.799	1.000e+00
Asanoa	0.412	1.612	5.799	NA
Fastidiosipila	0.578	1.612	5.799	1.000e+00
Propioniferax	0.646	1.612	5.799	1.000e+00
Reticulibacter	0.388	1.612	5.799	NA
Desulfotomaculum	0.503	1.612	5.799	1.000e+00
Nigerium	0.539	1.612	5.799	1.000e+00
Frigoriflavimonas	0.492	1.612	5.249	1.000e+00
Rubrivirga	0.461	1.612	5.799	NA
Fodinicola	0.339	1.612	5.799	NA
Ignavibacterium	0.501	1.612	5.799	1.000e+00
Pajaroellobacter	0.393	1.612	5.799	NA
Laetiporus	0.632	1.612	5.799	1.000e+00
Corticibacterium	0.417	1.612	5.799	NA
Vagococcus	0.490	1.612	5.799	1.000e+00
Nitrosocosmicus	2.028	1.612	4.329	1.000e+00
Macellibacteroides	0.431	1.612	5.799	NA
Hartmannibacter	0.457	1.612	5.799	NA
Zymomonas	0.330	1.612	5.799	NA
Anaerobutyricum	0.398	1.612	5.799	NA
Propionibacterium_phage_PHL301M00_virus	0.225	1.612	5.799	NA
Type-C_symbiont_of_Plautia_stali	0.212	1.612	5.799	NA
Aff.	0.263	1.612	5.799	NA
Nannocystis	1.346	1.612	4.195	1.000e+00
Qingrenia	0.304	1.612	5.799	NA
Coriobacteriaceae_genus	0.340	1.612	5.799	NA
Tuwongella	0.369	1.612	5.799	NA
Thermaurantiacus	0.224	1.612	5.799	NA
Thermoanaerobacter	0.395	1.612	5.799	NA

Fervidobacterium	0.190	1.612	5.799	NA
Allostreptomyces	0.331	1.612	5.799	NA
Stenoxybacter	0.289	1.612	5.799	NA
Zavarzinella	0.260	1.612	5.799	NA
Rickettsia	2.139	1.612	2.768	5.112e-01
Streptobacillus	0.298	1.612	5.799	NA
Lachnoclostridium	1.274	1.612	4.637	1.000e+00
Sphaerisporangium	0.405	1.612	5.799	NA
Moorella	0.340	1.612	5.799	NA
Tepidanaerobacter	0.405	1.612	5.799	NA
Propionispora	0.201	1.612	5.799	NA
Ezakiella	3.186	1.612	3.441	6.623e-01
Rickettsiales	0.188	1.612	5.799	NA
Drancourtella	0.234	1.612	5.799	NA
Allocoleopsis	0.836	1.612	4.166	1.000e+00
Chryseosolibacter	0.194	1.612	5.799	NA
Verticiella	1.122	1.612	5.126	1.000e+00
Holdemania	0.350	1.612	5.799	NA
Microthrix	0.483	1.612	5.799	1.000e+00
Haliangium	2.382	1.612	3.351	9.369e-01
Catellicoccus	1.455	1.612	4.287	1.000e+00
Planifilum	0.365	1.612	5.799	NA
Companilactobacillus	0.288	1.612	5.799	NA
Hemiselmis	1.714	1.612	3.491	9.946e-01
Mariprofundus	0.313	1.612	5.799	NA
Soleaferrea	0.148	1.612	5.799	NA
Durotheca	0.146	1.612	5.799	NA
Komarekiella	0.170	1.612	5.799	NA
Pontibrevibacter	0.195	1.612	5.799	NA
Provencibacterium	0.230	1.612	5.799	NA
Mariluticola	0.153	1.612	5.799	NA
Pedosphaera	0.186	1.612	5.799	NA
Pinibacter	0.256	1.612	5.799	NA
Quisquiliibacterium	0.686	1.612	4.915	1.000e+00
Atopococcus	0.184	1.612	5.799	NA
Chloroploca	0.537	1.612	5.799	1.000e+00
Rodentibacter	0.334	1.612	4.864	NA
Propionibacterium_phage_PHL117M01_virus	0.268	1.612	5.799	NA
Shouchella	0.581	1.612	5.799	1.000e+00
Aceticella	0.332	1.612	5.799	NA
Wolinella	0.625	1.612	4.932	1.000e+00
Adlercreutzia	0.609	1.612	4.525	1.000e+00
Aeribacillus	0.675	1.612	5.799	1.000e+00
Vitreoscilla	1.415	1.612	3.583	1.000e+00
Melittangium	0.138	1.612	5.799	NA
Weeksella	0.627	1.612	5.799	1.000e+00
Winslowiella	0.287	1.612	5.799	NA
Dinghuibacter	0.264	1.612	5.799	NA
Alkalihalophilus	0.129	1.612	5.799	NA
Sporomusaceae_genus	0.491	1.612	2.945	1.000e+00
Betaproteobacteria	0.088	1.612	5.799	NA
Desnuesiella	0.296	1.612	5.799	NA
Variimorphobacter	0.190	1.612	5.799	NA
Chryseolinea	0.170	1.612	5.799	NA
Pseudactinotalea	0.117	1.612	5.799	NA
Baumannia	0.277	1.612	5.799	NA
Hanamia	0.253	1.612	5.799	NA
Pelagerythrobacter	0.364	1.612	5.799	NA
Flintibacter	0.137	1.612	5.799	NA

Sabulicella	0.126	1.612	5.799	NA
Effusibacillus	0.123	1.612	5.799	NA
Mycoplana	0.174	1.612	5.799	NA
Rhodoblastus	0.592	1.612	4.141	1.000e+00
Thermobrachium	0.226	1.612	5.799	NA
Pseudohongiella	0.062	1.612	5.799	NA
Pelistega	0.239	1.612	5.799	NA
Arthromitus	0.148	1.612	5.799	NA
Acidimicrobium	0.273	1.612	5.799	NA
Neptuniibacter	0.092	1.612	5.799	NA
Agathobaculum	0.083	1.612	5.799	NA
Rubricoccus	0.152	1.612	5.799	NA
Nisaea	0.072	1.612	5.799	NA
Citreimonas	0.088	1.612	5.799	NA
Yeguia	0.064	1.612	5.799	NA
Macromonas	0.075	1.612	5.799	NA
Chloroflexales	0.074	1.612	5.799	NA
Neokomagataea	0.056	1.612	5.799	NA
Chloroflexia	0.066	1.612	5.799	NA
Geomonas	0.050	1.612	5.799	NA
Faecalibacillus	0.089	1.612	5.799	NA
Profftia	0.070	1.612	5.799	NA
Solirhodobacter	0.058	1.612	5.799	NA
Hirsutella	0.112	1.612	5.799	NA
Catenuloplanes	0.057	1.612	5.799	NA
Erysipelothrix	0.043	1.612	5.799	NA
Gynurincola	0.054	1.612	5.799	NA
Alterileibacterium	0.156	1.612	5.799	NA
Prauserella	0.035	1.612	5.799	NA
Robinsoniella	0.031	1.612	5.799	NA
Tenebrionicola	0.031	1.612	5.799	NA
Uruburuella	0.038	1.612	5.799	NA
Thermopolyspora	0.034	1.612	5.799	NA
Vescimonas	0.025	1.612	5.799	NA
Geoalkalibacter	0.121	1.612	5.799	NA
Buchananella	0.056	1.612	5.799	NA
Riesia	0.026	1.612	5.799	NA
SsRNA_phage_SRR5466369_2_virus	0.058	1.612	5.799	NA
Proteiniphilum	0.024	1.612	5.799	NA
Thermodesulfomicrobium	0.020	1.612	5.799	NA
Cucumibacter	0.022	1.612	5.799	NA
Calorimonas	0.022	1.612	5.799	NA
Hassallia	0.102	1.612	5.799	NA
Pseudodesulfovibrio	0.057	1.612	5.799	NA
Sulfuriferula	0.013	1.612	5.799	NA
Hominisplanchenecus	0.033	1.612	5.799	NA
Tachikawaea	0.009	1.612	5.799	NA
Methanothrix	0.009	1.612	5.799	NA
Neofamilia	0.005	1.612	5.799	NA
Pseudocnuella	0.005	1.612	5.799	NA
Zafaria	0.027	1.612	5.799	NA
Enterobacteria_phage_YYZ-2008_virus	0.009	1.612	5.799	NA
Plasticicumulans	0.004	1.612	5.799	NA
Betaproteobacterium_AAP99	0.003	1.612	5.799	NA
Nioella	0.006	1.612	5.799	NA
Hydromonas	0.010	1.612	5.799	NA
Pelovirga	0.002	1.612	5.799	NA
Botrytis	2.262	1.614	2.235	8.727e-01
Lichtheimia	35.429	1.615	0.798	4.787e-09

Rhizorhapis	25.863	1.617	0.949	1.942e-01
Thermogemmata	0.790	1.617	5.608	1.000e+00
Trichophyton	4.702	1.624	2.602	5.912e-01
Anabaena	1.596	1.629	2.653	1.000e+00
Sinirhodobacter	15.801	1.644	0.519	4.698e-09
Crenalkalicoccus	0.619	1.645	5.011	1.000e+00
Aromatoleum	0.470	1.646	5.798	1.000e+00
Parasphingorhabdus	1.615	1.646	2.439	6.712e-01
Roseicitreum	1.809	1.655	2.688	1.000e+00
Sedimentitalea	30.138	1.663	0.479	1.688e-03
Brevibacterium	127.378	1.667	0.697	5.672e-07
Muribaculaceae_genus	0.949	1.667	2.611	6.340e-01
Haematomicrobium	1.756	1.668	4.730	1.000e+00
Rhodospirillum	1.657	1.668	2.706	7.416e-01
Granulicoccus	0.329	1.668	5.779	NA
Bacteroidetes	1.272	1.674	4.403	1.000e+00
Arcobacter	3.620	1.675	1.635	7.719e-01
Gramella	1.113	1.685	1.764	8.560e-01
Aquincola	21.141	1.702	1.055	4.204e-01
Empedobacter	31.761	1.705	0.670	2.366e-03
Oscillochloris	0.293	1.715	5.776	NA
Lachnospira	17.322	1.734	0.845	1.096e-03
Salinimicrobium	20.796	1.737	0.809	8.963e-05
Thalassobius	5.691	1.743	1.494	1.000e+00
Abelson	3.843	1.766	0.843	4.372e-04
Wickerhamomyces	2.767	1.772	2.963	9.946e-01
Dermabacter	5.429	1.775	1.754	5.708e-01
Parasegetibacter	0.335	1.775	5.799	NA
Nitrobacter	5.995	1.777	1.282	5.549e-01
Steroidobacter	1.439	1.782	2.884	1.000e+00
Rhizophagus	4.450	1.791	1.887	1.035e-01
Mitosporidium	133.751	1.801	1.939	8.255e-08
Phaeoacremonium	1.292	1.801	1.170	7.758e-01
Novosphingobium	579.919	1.808	0.519	7.656e-08
Yimella	7.431	1.815	1.479	1.000e+00
Microbacter	0.938	1.818	3.897	1.000e+00
Bifidobacterium	80.934	1.823	0.765	2.477e-04
Paracidovorax	147.082	1.824	0.459	4.100e-19
Cordyceps	0.785	1.839	4.286	1.000e+00
Hyphomonas	0.992	1.842	3.933	1.000e+00
Segetibacter	3.587	1.854	2.354	1.574e-01
Micrococcus	653.697	1.859	0.552	4.399e-05
Sulfolobus	2.237	1.880	0.864	2.520e-02
Mumia	1.011	1.884	4.396	1.000e+00
Elioraea	0.452	1.884	5.239	NA
Pseudidiomarina	0.129	1.884	5.231	NA
Oerskovia	0.479	1.884	5.798	1.000e+00
Planomonospora	0.303	1.884	5.798	NA
Trujillella	0.198	1.884	5.798	NA
Agaricicola	0.028	1.884	5.798	NA
Pseudarthrobacter	88.036	1.887	0.894	2.117e-06
Propionicicella	2.147	1.892	2.614	9.966e-01
Brevundimonas	1147.754	1.906	0.372	6.872e-11
Candidatus	2.000	1.910	3.144	9.966e-01
Pseudorhizobium	3.035	1.912	2.291	9.953e-01
Comamonas	377.215	1.916	0.295	1.222e-20
Algibacillus	1.251	1.918	1.804	5.494e-01
Methyloradius	0.301	1.920	5.798	NA
Oxalicibacterium	0.983	1.961	3.246	1.000e+00

Schizosaccharomyces	2.310	1.964	2.297	1.000e+00
Pelagivirga	2.684	1.972	1.243	4.661e-01
Hyphobacterium	32.142	1.973	0.547	1.895e-03
Flavimobilis	2.525	1.976	2.233	1.000e+00
Mus_musculus_mobilized_virus	23.218	1.980	0.612	3.901e-02
Meyerozyma	1.367	1.995	4.447	1.000e+00
Microcoleus	16.615	2.000	1.062	2.483e-01
Mixia	2.238	2.002	2.356	5.997e-01
Desertimonas	18.398	2.020	1.900	7.574e-01
Methylococcus	3.490	2.024	1.433	6.341e-01
Arcticiflavibacter	197.563	2.037	0.585	7.391e-04
Falsirhodobacter	4.108	2.039	2.316	9.966e-01
Herbaspirillum	591.387	2.055	0.206	1.741e-55
Buttiauxella	0.847	2.059	1.921	5.722e-01
Sclerotinia	1.417	2.067	2.756	1.000e+00
Cnuella	0.966	2.067	4.140	1.000e+00
Rhodocista	0.302	2.067	4.242	NA
Pseudovibrio	0.453	2.069	5.786	NA
Limnobaculum	0.794	2.070	2.440	1.000e+00
Penaeicola	1.825	2.076	1.805	8.080e-01
Amniculibacterium	0.863	2.077	5.768	1.000e+00
Westerdykella	4.874	2.086	1.669	4.862e-02
Oceanicola	3.175	2.100	2.074	4.049e-01
harvey_murine_sarcoma_virus	1.591	2.107	1.063	1.657e-04
Mitsuaria	151.580	2.114	0.538	2.491e-07
Aeromonas	111.663	2.117	0.497	5.048e-07
Perlucidibaca	2.185	2.122	2.085	1.000e+00
Chrysosporum	14.164	2.128	1.431	5.026e-01
Dorea	18.063	2.131	1.165	5.887e-01
Aquabacterium	1382.076	2.138	0.233	3.317e-68
Saezia	2.274	2.150	1.752	5.240e-01
Type-E_symbiont_of_Plautia_stali	0.036	2.150	5.798	NA
Arthroderma	0.451	2.151	2.864	NA
Streptacidiphilus	2.022	2.168	3.129	5.720e-01
Tabrizicola	4.823	2.171	1.662	4.889e-01
Flaviaesturariibacter	0.912	2.172	5.412	1.000e+00
Yoonia	0.283	2.172	5.798	NA
Zygosaccharomyces	0.127	2.172	5.798	NA
Formosimonas	0.082	2.172	5.798	NA
Exophiala	38.352	2.177	0.996	2.746e-07
Kocuria	801.833	2.184	0.831	1.362e-06
Pengzhenrongella	1.108	2.195	3.519	1.000e+00
Lacrimispora	0.872	2.197	4.166	1.000e+00
Aliicoccus	0.581	2.199	5.794	1.000e+00
Fimbriimonas	2.579	2.208	2.334	8.908e-01
Undibacterium	178.266	2.219	0.602	3.299e-04
FBR_murine_osteosarcoma_virus	1.393	2.230	1.154	2.124e-01
Bergeriella	2.718	2.238	3.406	9.480e-01
Azoarcus	4.010	2.239	1.680	6.581e-01
Halobacteriovorax	2.262	2.252	1.311	4.297e-01
Azospirillum	271.311	2.254	0.577	1.444e-07
Seohaeicola	2.294	2.280	2.616	6.363e-01
Micropruina	6.588	2.283	1.995	1.000e+00
Sphaerobacter	12.579	2.288	2.384	6.307e-01
Acidaminococcus	0.185	2.288	5.789	NA
Saitoella	68.439	2.297	2.193	1.198e-05
BeAn_58058_virus	0.620	2.309	2.460	1.000e+00
Emericellopsis	3.316	2.310	2.958	6.907e-01
Alkanindiges	7.099	2.314	1.459	5.720e-01

Synchytrium	4.292	2.316	2.462	3.598e-01
Schlegelella	25.036	2.328	0.835	2.389e-01
Kineosporia	6.745	2.347	1.982	6.146e-01
Siccirubricoccus	2.442	2.354	2.765	8.048e-01
Capillimicrobium	2.546	2.356	2.487	5.099e-01
Alloiococcus	1.164	2.359	2.873	1.000e+00
PreXMRV-1_provirus_complete	4.785	2.361	0.882	5.422e-03
Megasphaera	26.909	2.384	1.435	8.888e-01
Microlunatus	147.679	2.425	0.945	1.165e-03
Pseudophaeobacter	5.590	2.430	1.557	5.214e-01
Rhizobacter	51.463	2.433	0.456	2.037e-16
Abyssicoccus	5.009	2.444	2.161	3.327e-01
Pseudoroseomonas	17.498	2.457	1.165	3.804e-03
Millisia	1.115	2.472	4.853	1.000e+00
Spleen_focus-forming_virus	3.983	2.472	0.887	4.552e-02
Caldimonas	7.771	2.472	1.753	3.985e-01
Gellertiella	2.769	2.474	3.331	8.801e-01
Allosphingosinicella	0.640	2.482	3.794	1.000e+00
Roseitranquillus	1.921	2.483	4.003	1.000e+00
Schumannella	0.440	2.486	5.797	NA
Actinosynnema	3.106	2.489	1.724	4.816e-01
Brachybacterium	121.244	2.496	0.760	1.591e-02
Ochrobactrum	77.175	2.514	0.549	6.511e-13
Endobacter	3.493	2.523	2.508	1.000e+00
Pluralibacter	1.241	2.523	2.868	9.745e-01
Polychytrium	132.053	2.524	1.495	7.462e-08
Acidithiobacillus	129.947	2.545	0.785	1.653e-08
Roseburia	44.523	2.550	0.612	4.513e-07
Oceanimonas	3.842	2.554	1.403	5.882e-02
Brooklawnia	1.148	2.573	4.968	1.000e+00
Belnapia	18.847	2.582	1.137	2.791e-02
Yaniella	2.635	2.584	2.542	8.752e-01
Paenacidovorax	3.180	2.589	1.892	2.588e-01
Dichomitus	24.638	2.603	1.266	1.281e-01
Dietzia	166.910	2.605	0.572	3.568e-06
Erythrobacteraceae_genus	1.717	2.614	2.703	1.000e+00
Murine_osteosarcoma_virus	2.353	2.618	1.206	1.266e-02
Roseobacter	0.876	2.625	2.933	1.000e+00
Boeremia	20.842	2.635	1.579	1.966e-01
Acetanaerobacterium	0.693	2.643	4.195	1.000e+00
Mycolicibacterium	115.168	2.645	0.440	6.307e-22
Longimicrobium	6.592	2.649	2.066	1.000e+00
Piscinibacter	118.721	2.678	0.543	6.442e-15
Chlorogloea	2.108	2.709	3.075	1.000e+00
Cellulosimicrobium	4.701	2.717	2.527	9.679e-01
Demequina	2.047	2.718	2.267	1.000e+00
Acetivibrio	1.359	2.751	4.780	1.000e+00
Tepidiforma	2.948	2.762	3.039	6.532e-01
Chaetomium	2.173	2.782	2.143	5.820e-01
Stutzerimonas	48.535	2.784	0.955	1.528e-01
Delta	4.266	2.795	1.102	6.251e-02
Rubrivivax	73.057	2.800	0.542	4.673e-11
Herpetosiphon	1.350	2.804	3.708	1.000e+00
Stappia	1.579	2.816	2.937	1.000e+00
Hydrobacter	0.593	2.818	5.041	1.000e+00
Arboricoccus	0.363	2.820	5.795	NA
Petrimonas	0.193	2.820	5.795	NA
Roseovarius	20.641	2.831	0.633	1.855e-04
Intrasporangium	1.177	2.834	4.116	1.000e+00

Lobosporangium	154.598	2.870	1.861	1.020e-06
Sphingopyxis	66.886	2.871	0.687	1.083e-05
Glaciimonas	3.779	2.883	2.049	1.000e+00
Betaproteobacterium_AAP51	11.054	2.906	0.914	2.725e-06
Nosocomiicoccus	0.953	2.926	3.955	1.000e+00
Agrococcus	150.918	2.930	1.123	2.338e-05
Methylomonas	1.727	2.937	1.137	7.272e-02
Beutenbergia	4.200	2.937	2.140	3.969e-01
Limnobacter	140.336	2.940	0.994	2.127e-07
Tsuneonella	1.670	2.953	3.442	9.259e-01
Saccharothrix	13.589	2.969	1.020	2.745e-02
Puteibacter	1.005	2.979	2.504	1.000e+00
Leptothrix	258.760	2.980	0.425	3.041e-31
Halochromatium	4.224	3.013	1.258	3.455e-01
Wickerhamiella	8.538	3.021	1.210	1.021e-03
Lentihominibacter	0.331	3.028	5.790	NA
Paenalcaligenes	0.760	3.066	1.867	4.703e-01
Oceanitalea	2.776	3.068	2.903	3.329e-01
Actinoallomurus	0.960	3.080	3.902	1.000e+00
Chloroflexi	0.505	3.106	3.468	1.000e+00
Pseudaminobacter	1.415	3.119	3.391	9.011e-01
Enterocloster	0.679	3.154	4.427	1.000e+00
Xinfangfangia	1.197	3.161	3.359	1.000e+00
Alteromonas	44.223	3.169	0.791	2.984e-09
Amnimonas	9.718	3.192	2.142	4.593e-01
Endozoicomonas	0.368	3.290	2.978	NA
Lampropedia	1.054	3.312	3.679	1.000e+00
Friedmanniella	3.725	3.315	2.381	1.000e+00
Acaricomes	2.882	3.317	1.680	2.100e-01
Propionibacterium_phage_SKKY_virus	0.362	3.319	5.791	NA
Terrihabitans	6.726	3.378	3.434	6.727e-01
Oscillibacter	0.732	3.395	5.790	1.000e+00
Didymella	21.350	3.401	1.703	9.877e-04
Amaricoccus	20.347	3.457	1.028	2.094e-02
Anaerosphaera	0.551	3.497	5.775	1.000e+00
Afifella	2.232	3.505	3.754	9.966e-01
Mycoavidus	1.316	3.513	3.797	1.000e+00
Polyangium	64.134	3.540	0.890	2.294e-09
Gullanella	1.395	3.541	2.061	7.841e-01
Seonamhaeicola	12.679	3.557	0.597	1.872e-16
Georgenia	10.823	3.560	1.171	1.258e-02
Vulcaniibacterium	1.978	3.578	3.367	8.387e-01
Letharia	104.390	3.601	0.915	6.220e-11
Salinispora	1.822	3.628	2.936	9.639e-01
Parvibaculum	1.055	3.630	3.004	1.000e+00
Alkalihalobacillus	4.260	3.634	1.008	4.455e-05
Stakelama	0.615	3.646	3.750	1.000e+00
Argonema	0.523	3.666	5.788	1.000e+00
Haematobacter	4.763	3.670	2.286	3.351e-01
Yarrowia	13.249	3.674	1.352	2.601e-03
Paenimyroides	6.925	3.685	1.712	2.442e-02
Viridilinea	0.240	3.686	5.788	NA
Scleromatobacter	0.808	3.687	5.777	1.000e+00
Halteromyces	0.521	3.692	3.906	1.000e+00
Pseudochrobactrum	75.819	3.700	0.642	3.415e-19
Trematosphaeria	6.493	3.702	1.823	6.320e-02
Pilimelia	0.808	3.809	3.346	1.000e+00
Bordetella	34.897	3.815	0.793	3.387e-08
Liquorilactobacillus	0.867	3.825	4.009	1.000e+00

Glycocaulis	61.217	3.835	1.607	2.040e-08
Aggregicoccus	0.236	3.866	5.786	NA
Murine_type_C_virus	17.101	3.889	0.807	1.104e-09
Macroventuria	247.054	3.914	1.094	1.422e-16
Pseudosporangium	0.338	3.922	5.786	NA
Sagittula	4.544	3.936	2.468	7.448e-01
Ustilaginoidea	1.194	3.938	3.719	1.000e+00
Silanimonas	0.294	3.944	5.785	NA
Desulfofundulus	0.368	3.979	5.785	NA
Ascochyta	1160.970	4.027	1.097	1.419e-19
Pontibacter	44.467	4.060	2.596	1.482e-03
Spiribacter	38.734	4.083	0.663	1.582e-11
Filifactor	2.916	4.126	2.083	8.908e-01
Gemmiger	2.088	4.136	3.073	9.031e-01
Sneathia	1.805	4.137	4.738	1.000e+00
Leptosphaeria	79.063	4.159	1.310	1.939e-07
Calidifontimicrobium	0.522	4.166	5.783	1.000e+00
Mucor	14.488	4.190	1.456	1.306e-03
Auraticoccus	1.430	4.273	5.744	1.000e+00
Halovulum	1.008	4.312	3.542	1.000e+00
Aliarcobacter	1.118	4.346	5.782	1.000e+00
Elstera	0.783	4.352	5.553	1.000e+00
Kirsten_murine_sarcoma_virus	1.366	4.359	3.302	7.876e-01
Aquirhabdus	0.376	4.438	5.782	NA
Quadrisphaera	26.642	4.438	1.723	1.966e-02
Pelobacter	1.250	4.440	3.700	1.000e+00
Tsukamurella	8.402	4.441	2.522	7.947e-01
Alkalibacterium	1.571	4.462	3.347	1.000e+00
Desulfuromonas	1.691	4.475	3.109	8.908e-01
Nitrosospira	14.651	4.484	2.350	9.877e-04
Oleiphilus	1.965	4.513	1.521	3.465e-01
Dokdonella	0.875	4.538	4.415	1.000e+00
Maridesulfovibrio	0.477	4.584	5.779	1.000e+00
Planomicrobium	4.483	4.593	2.725	1.501e-01
Embleya	1.484	4.622	3.360	1.000e+00
Segnochrobactrum	1.097	4.643	4.807	1.000e+00
Sugiyamaella	3.235	4.645	2.218	7.632e-02
Veillonellaceae_genus	0.824	4.650	5.388	1.000e+00
Mycena	39.124	4.656	1.508	2.250e-02
Anatilimnocola	0.489	4.665	5.780	1.000e+00
Zychaea	18.213	4.670	2.400	4.569e-02
Propionibacterium_phage_PAD20_virus	0.688	4.699	5.780	1.000e+00
Occultella	0.470	4.699	5.780	1.000e+00
Emergencia	2.121	4.743	2.489	9.585e-02
Vallicoccus	2.969	4.772	2.695	7.741e-01
Parastagonospora	616.798	4.797	0.991	8.231e-17
Nitriliruptoraceae_genus	156.786	4.829	1.356	1.576e-11
Holdemanella	0.771	4.840	4.487	1.000e+00
Lacipirellula	1.585	4.864	4.102	9.031e-01
Gallaecimonas	2.804	4.874	1.962	3.828e-01
Hyalangium	1.005	4.910	5.219	1.000e+00
Actinobacterium	1.262	4.961	3.661	1.000e+00
Krasilnikoviella	0.643	4.974	5.779	1.000e+00
Labrys	177.824	4.979	0.685	9.501e-19
Mycobacteroides	34.731	5.000	1.063	5.457e-07
Tersicoccus	1.047	5.033	5.774	1.000e+00
Motilibacter	2.373	5.039	2.939	6.091e-01
Rhodocyclales	0.940	5.040	5.179	1.000e+00
Nitrososphaera	1.353	5.072	5.778	1.000e+00

Ktedonobacter	0.818	5.087	5.778	1.000e+00
Lentilactobacillus	33.253	5.095	1.917	1.984e-03
Phaeosphaeria	6.620	5.102	2.841	2.411e-01
Neptunicoccus	0.662	5.168	5.778	1.000e+00
Dermatobacter	1.264	5.213	3.634	9.214e-01
Clavispora	11.624	5.250	2.516	4.618e-02
Radiomyces	1.508	5.282	2.437	2.946e-01
Protomyces	15.298	5.293	1.741	1.176e-02
Metabacillus	4.195	5.295	1.948	1.270e-02
Hephaestia	3.618	5.554	2.427	5.494e-01
Sphingomonadaceae_genus	3.430	5.602	2.747	3.781e-01
Beijerinckiaceae_genus	37.739	5.614	1.115	1.361e-08
Spirilliplanes	0.975	5.707	5.776	1.000e+00
Desulfocarbo	2.982	5.760	1.648	8.756e-03
Paracandidimonas	1.505	5.777	4.069	8.714e-01
Phaseolus_vulgaris_endornavirus	1.016	5.836	5.776	1.000e+00
Brettanomyces	5.223	5.921	2.880	5.826e-01
Serinicoccus	39.889	5.922	1.495	6.566e-05
Advenella	5.711	5.994	2.006	1.834e-01
Mycotypha	40.609	6.232	1.889	1.743e-05
Cytobacillus	13.792	8.194	1.643	9.721e-10
Melampsora	15.381	8.993	2.225	1.208e-03
