# Supplementary material for: Shotgun Metagenome Analysis of Two Schizaphis graminum Biotypes over Time With and Without Carried Cereal Yellow Dwarf Virus
Source: Insects. 2025 May 23;16(6):554. doi: 10.3390/insects16060554 (PMC12193481; doi:10.3390/insects16060554)
Supplement: Supplementary file 1 [file insects-16-00554-s001.zip › Table S4.pdf]

Table S4. DESeq2 results for comparison by biotype, arranged by BH-adjusted p-value.

Genus	BaseMean	Log2FC	LFCSE	Padj
Letharia	146.679	-3.690	0.527	1.728e-07
Aequitasia	109.785	-6.263	0.905	1.182e-05
Salipaludibacillus	74.463	-2.707	0.477	1.859e-05
Salipiger	55.657	-1.402	0.266	3.845e-05
Fimicolochytrium	88.813	-6.632	1.020	6.071e-05
Batrachochytrium	112.118	-28.293	1.368	8.685e-05
Polaribacter	19.839	-2.434	0.477	1.463e-04
Agilicoccus	113.618	-2.542	0.521	3.349e-04
Pseudomonas	23142.274	-0.967	0.216	7.413e-04
Proteobacteria	45.108	-3.137	0.666	8.664e-04
Melampsora	13.623	-7.249	1.176	1.076e-03
Pseudooceanicola	54.236	-1.751	0.400	1.157e-03
Macroventuria	247.054	-3.156	0.692	1.429e-03
Parastagonospora	616.798	-2.742	0.620	1.614e-03
Acidithiobacillus	129.947	-1.908	0.445	1.614e-03
Streptomyces	1952.590	-0.566	0.137	1.771e-03
Mycotypha	36.618	-5.142	1.091	1.904e-03
Microvirga	140.126	1.571	0.378	2.032e-03
Wickerhamiella	12.358	-2.927	0.688	2.391e-03
Rhabdonatronobacter	170.476	-0.912	0.227	2.419e-03
Meira	91.815	-4.482	0.990	2.929e-03
Ascochyta	1160.970	-2.988	0.710	3.059e-03
Hydrogenophaga	66.646	-1.429	0.360	3.207e-03
Propioniciclava	35.109	-2.243	0.566	4.092e-03
Leyella	5.334	-4.171	1.105	8.117e-03
Azotobacter	95.528	-1.536	0.422	1.037e-02
Qipengyuania	57.461	-1.356	0.380	1.176e-02
Thiolapillus	20.111	-2.661	0.719	1.176e-02
Terrimonas	47.737	-1.756	0.490	1.199e-02
Guillardia	48.159	-3.820	0.980	1.199e-02
Novosphingobium	521.937	-0.970	0.279	1.345e-02
Psychrobacter	113.554	-0.963	0.280	1.551e-02
Saccharothrix	13.589	-1.821	0.527	1.551e-02
Nodosilinea	25.845	-2.605	0.738	1.654e-02
Leptosphaeria	79.063	-2.699	0.768	1.954e-02
Mucor	15.110	-2.761	0.805	2.024e-02
Massilia	397.055	0.906	0.274	2.093e-02
Glutamicibacter	58.869	-1.529	0.469	2.468e-02
Phyllobacterium	39.527	-1.694	0.511	2.468e-02
Sporosarcina	5.918	-2.407	0.745	2.468e-02
Clostridiales	35.068	-1.211	0.371	2.496e-02
Mitsuaria	151.580	-0.974	0.305	2.809e-02
Rhodobacteraceae_genus	44.452	-1.471	0.458	2.857e-02
Protofrankia	75.565	-1.221	0.385	2.904e-02
Polychytrium	132.053	-3.028	0.903	3.014e-02
Colwellia	7.776	-1.565	0.497	3.185e-02
Loigolactobacillus	25.133	-3.116	0.941	3.332e-02
Porphyromonas	55.787	1.087	0.350	3.332e-02
Neofusicoccum	52.004	-1.451	0.467	3.490e-02
Aeromonas	84.822	-0.651	0.219	4.397e-02
Spizellomyces	92.702	-3.477	1.072	4.529e-02
Corallococcus	123.324	-0.762	0.257	4.537e-02
Cloacibacterium	93.517	1.307	0.439	4.751e-02
Didymella	21.350	-2.978	0.967	4.913e-02
Wallemia	78.999	-3.044	0.992	5.744e-02

Paeniglutamicibacter	22.306	-1.192	0.408	5.744e-02
Dorea	18.063	-1.659	0.575	5.744e-02
Anoxybacillus	29.382	1.784	0.616	6.320e-02
Apibacter	8.111	2.969	0.986	6.810e-02
Boeremia	20.842	-2.314	0.804	6.810e-02
Mollisia	7.237	2.274	0.796	7.367e-02
Mixta	10.290	-1.601	0.569	7.569e-02
Blautia	17.234	-1.467	0.530	7.569e-02
Rhizorhapis	25.863	-1.317	0.478	7.569e-02
Mammaliicoccus	12.984	1.951	0.702	8.419e-02
Devosia	74.074	-0.904	0.335	8.422e-02
Mitosporidium	133.751	-3.722	1.268	9.076e-02
Trichoderma	47.821	1.768	0.653	9.398e-02
Trichosporon	145.713	-1.948	0.721	9.555e-02
Acaromyces	26.363	-2.366	0.867	9.576e-02
Uncultured	130.743	-0.921	0.349	9.576e-02
Epilithonimonas	41.936	-0.916	0.353	1.012e-01
Mycena	39.124	-2.131	0.797	1.035e-01
Arcticiflavibacter	197.563	0.809	0.316	1.068e-01
Stereum	19.599	1.969	0.746	1.068e-01
Algiphilus	9.411	-2.242	0.882	1.068e-01
Okeania	8.897	-1.939	0.754	1.068e-01
Picosynechococcus	10.813	1.808	0.687	1.068e-01
Suillus	38.317	-2.515	0.948	1.097e-01
Endosymbiont	5.833	-1.397	0.566	1.104e-01
Agarivorans	92.107	-1.426	0.574	1.295e-01
Lobosporangium	154.598	-3.056	1.161	1.295e-01
Pseudarthrobacter	55.707	1.114	0.446	1.295e-01
Filobasidium	18.977	1.247	0.503	1.295e-01
Hymenobacter	123.295	-0.787	0.322	1.314e-01
Chrysosporum	14.164	-1.746	0.707	1.314e-01
Pseudoalteromonas	365.269	-0.482	0.200	1.377e-01
Protomyces	14.504	-2.668	0.946	1.573e-01
Solirubrobacter	30.724	-1.434	0.602	1.617e-01
Aeromicrobium	36.608	-0.794	0.339	1.661e-01
Hyphobacterium	32.142	0.686	0.295	1.661e-01
Fusarium	142.957	-1.322	0.563	1.673e-01
Dialister	5.744	2.180	0.907	1.726e-01
Sphaerobacter	12.579	-2.814	1.164	1.772e-01
Geobacillus	32.342	-1.444	0.618	1.781e-01
Lentilactobacillus	33.253	-2.626	1.097	1.842e-01
Kocuria	483.512	-0.920	0.407	1.894e-01
Trematosphaeria	6.493	-2.157	0.982	1.952e-01
Anaerococcus	995.459	1.235	0.549	2.016e-01
Pseudomicrostroma	29.079	-1.879	0.830	2.079e-01
Finegoldia	126.567	1.236	0.558	2.102e-01
Aplosporella	8.752	1.667	0.747	2.102e-01
Stomatobaculum	7.662	1.685	0.758	2.102e-01
Entotheonella	5.670	-1.560	0.721	2.102e-01
Legionella	1332.136	-0.508	0.234	2.121e-01
Robertmurraya	10.088	-1.323	0.616	2.244e-01
Ectobacillus	119.344	-0.752	0.349	2.252e-01
Rhabdothermincola	6.601	-1.721	0.826	2.252e-01
Veillonella	194.159	-0.696	0.328	2.341e-01
Niastella	47.268	-2.653	1.185	2.341e-01
Caulobacter	1070.743	0.488	0.232	2.409e-01
Dichomitus	29.347	-1.440	0.673	2.409e-01
Acanthamoeba	49.409	-1.420	0.667	2.453e-01
Pantoea	249.985	-0.369	0.178	2.488e-01

Campylobacter	38.374	0.587	0.283	2.488e-01
Undibacterium	178.266	0.688	0.331	2.494e-01
Pontibacillus	12.762	-2.875	1.243	2.512e-01
Phenylobacterium	73.211	-0.789	0.382	2.556e-01
Alkalispirochaeta	9160.805	-0.477	0.234	2.617e-01
Tepidiphilus	66.163	1.234	0.599	2.650e-01
Dysgonomonas	10.780	0.876	0.434	2.702e-01
Dechloromonas	8.894	-1.432	0.718	2.702e-01
Ornithinimicrobium	139.539	-0.903	0.448	2.775e-01
Saitoella	68.439	-3.084	1.433	2.775e-01
Penicillium	43.107	-0.710	0.356	2.790e-01
Carnobacterium	19.889	-0.947	0.477	2.790e-01
Oceanibium	5.401	1.264	0.639	2.790e-01
Microsporum	6.726	-2.034	1.033	2.798e-01
Limnobacter	140.336	1.157	0.577	2.810e-01
Serpula	11.118	2.372	1.146	2.827e-01
Myceligenerans	6.325	-1.072	0.572	3.035e-01
Facklamia	31.482	-0.762	0.397	3.053e-01
Moraxella	437.707	-0.471	0.245	3.068e-01
Micromonospora	92.552	0.822	0.426	3.068e-01
Paucibacter	116.341	-0.437	0.230	3.166e-01
Cutaneotrichosporon	11.862	-1.660	0.870	3.166e-01
Saprolegnia	7.421	-1.061	0.567	3.214e-01
Schizophyllum	63.317	-1.665	0.867	3.275e-01
Lawsonella	184.540	0.731	0.389	3.285e-01
Allomuricauda	54.976	-0.632	0.338	3.285e-01
Acetobacter	16.394	0.615	0.329	3.285e-01
Phytoplasma	68.366	0.568	0.307	3.396e-01
Curtobacterium	149.150	0.669	0.361	3.416e-01
Pelomonas	2109.665	-0.322	0.177	3.471e-01
Micrococcus	532.483	-0.491	0.269	3.471e-01
Ramlibacter	69.721	-0.681	0.372	3.471e-01
Zychaea	9.011	-2.348	1.281	3.471e-01
Alishewanella	18.469	1.211	0.658	3.507e-01
Alteromonas	44.223	-0.882	0.485	3.507e-01
Barrientosiimonas	8.157	-2.051	1.005	3.507e-01
Rhodopirellula	17.595	-0.703	0.395	3.713e-01
Desulfovibrio	71087.628	-0.394	0.222	3.750e-01
Amnibacterium	9.282	1.400	0.779	3.750e-01
Mediterraneibacter	9.994	0.738	0.422	3.820e-01
Luteolibacter	24.040	-0.694	0.395	4.023e-01
Acuticoccus	38.672	0.413	0.249	4.070e-01
Diolcogaster_facetosa_bracovirus	12.306	-0.986	0.563	4.070e-01
Pneumocystis	8.386	-2.046	1.198	4.096e-01
Nakamurella	30.160	-0.897	0.520	4.110e-01
Providencia	883.343	-0.267	0.157	4.139e-01
Roseomonas	144.562	-0.648	0.383	4.192e-01
Jatrophihabitans	6.706	-1.691	1.007	4.192e-01
Domibacillus	6.862	-1.928	1.169	4.192e-01
Aliidiomarina	6.184	0.977	0.575	4.192e-01
Talaromyces	8.852	-1.108	0.673	4.209e-01
Aspergillus	116.582	-0.562	0.337	4.293e-01
Saccharopolyspora	62.086	0.345	0.210	4.312e-01
Delftia	511.917	0.316	0.191	4.393e-01
Collimonas	9.925	1.159	0.701	4.397e-01
Nitriliruptoraceae_genus	156.786	-1.506	0.900	4.398e-01
Mycobacteroides	34.731	1.065	0.639	4.398e-01
Variovorax	102.492	-0.399	0.244	4.449e-01
Azonexus	5.356	-1.898	1.067	4.471e-01

Cellvibrio	6.798	1.179	0.718	4.483e-01
Trametes	13.922	-1.240	0.764	4.490e-01
Mycolicibacterium	115.168	0.516	0.317	4.490e-01
Conexibacter	13.631	-1.166	0.715	4.490e-01
Kitasatospora	20.269	-0.888	0.554	4.490e-01
Punctularia	12.219	1.452	0.882	4.490e-01
Aliiruegeria	16.063	-0.919	0.570	4.490e-01
Zymoseptoria	38.563	-0.609	0.381	4.561e-01
Lutimaribacter	14.713	-0.870	0.549	4.561e-01
Iamia	6.518	-1.770	1.081	4.561e-01
Alloscardovia	83.257	-0.703	0.443	4.585e-01
Ralstonia	14254.930	0.253	0.160	4.610e-01
Citricoccus	29.626	-0.785	0.497	4.610e-01
Tolypothrix	8.797	-1.287	0.846	4.829e-01
Enterococcus	638.409	0.256	0.168	4.856e-01
Lysobacter	182.469	-0.321	0.210	4.856e-01
Paenibacillus	158.355	-0.278	0.182	4.856e-01
Nesterenkonia	69.289	0.413	0.270	4.856e-01
Leptospira	69.642	0.420	0.274	4.856e-01
Acidihalobacter	28.945	-0.794	0.517	4.856e-01
Lachnoanaerobaculum	14.139	0.610	0.399	4.856e-01
Actinobaculum	7.638	-1.286	0.828	4.856e-01
Mycobacterium	323.882	-0.283	0.186	4.876e-01
Gamsiella	5.792	-1.581	1.087	4.876e-01
Kineosporia	6.745	-1.650	0.995	4.876e-01
Rhizobacter	51.463	0.459	0.306	4.912e-01
Weissella	5.780	-1.122	0.782	4.920e-01
Planctomonas	7.195	-1.581	0.920	5.084e-01
Pseudocercospora	7.879	0.936	0.628	5.087e-01
Nocardioides	721.043	-0.227	0.154	5.177e-01
Sphaerulina	8.660	-0.947	0.649	5.199e-01
Caldifermentibacillus	8.825	1.609	1.076	5.249e-01
Ectothiorhodospira	16.455	-0.519	0.358	5.327e-01
Alternaria	98.393	-0.329	0.227	5.331e-01
Brevibacterium	75.382	-0.453	0.314	5.378e-01
Burkholderiaceae_genus	42.128	0.478	0.335	5.378e-01
Lachnospira	14.399	-0.630	0.434	5.378e-01
Fibrisoma	20.446	0.745	0.524	5.388e-01
Neisseria	439.766	0.295	0.209	5.388e-01
Thermicanus	22.909	1.310	0.914	5.388e-01
Heterobasidion	24.973	1.214	0.851	5.388e-01
Roseibacterium	113.257	-0.644	0.456	5.388e-01
Agrococcus	150.918	0.926	0.642	5.388e-01
Gemmobacter	16.490	-0.942	0.660	5.388e-01
Apiotrichum	11.988	-1.094	0.770	5.388e-01
Patulibacter	9.106	1.061	0.737	5.388e-01
Methanotrophic	6.831	1.106	0.778	5.388e-01
Tsukamurella	5.879	1.834	1.257	5.388e-01
Alicycliphilus	12.250	0.622	0.443	5.396e-01
Rhizobium	451.252	0.224	0.160	5.468e-01
Didymosphaeria	32.344	-0.838	0.601	5.474e-01
Georgenia	10.823	-0.927	0.634	5.588e-01
Yersinia	118.324	-0.527	0.383	5.592e-01
Janthinobacterium	149.394	0.332	0.242	5.613e-01
Vibrio	638.107	0.305	0.224	5.623e-01
Faecalibacterium	9.066	-1.076	0.804	5.623e-01
Ideonella	12.997	-0.755	0.557	5.623e-01
Methylomicrobium	16.552	-0.772	0.561	5.623e-01
Mogibacterium	9.735	0.843	0.622	5.667e-01

Haemophilus	188.386	-0.321	0.238	5.672e-01
Peptoniphilus	199.711	0.702	0.520	5.686e-01
Aphanizomenon	21.520	-0.333	0.262	5.686e-01
Thermococcus	14.111	-0.543	0.406	5.697e-01
Buchnera	70221.134	-0.246	0.184	5.738e-01
Corynebacterium	1916.937	0.299	0.225	5.738e-01
Lysinibacillus	127.976	-0.487	0.367	5.738e-01
Methylovulum	39.553	-0.629	0.471	5.738e-01
Paludifilum	42.859	0.293	0.223	5.814e-01
Eleftheria	9.525	-0.819	0.622	5.814e-01
Paraburkholderia	685.653	0.209	0.160	5.878e-01
Sporolactobacillus	110.272	-0.206	0.158	5.892e-01
Pseudonocardia	81.981	0.498	0.383	5.948e-01
Blastococcus	188.669	-0.528	0.411	6.026e-01
Exiguobacterium	51.512	-0.435	0.338	6.026e-01
Adhaeribacter	6.285	1.472	1.122	6.026e-01
Gilbertella	13763.122	0.295	0.232	6.057e-01
Parasaccharibacter	82.051	-0.271	0.214	6.057e-01
Chitinimonas	12.648	-0.876	0.678	6.057e-01
Ochrobactrum	77.175	-0.441	0.349	6.171e-01
Acidaminobacter	7.508	-0.828	0.660	6.171e-01
Modestobacter	77.884	-0.335	0.269	6.215e-01
Alloprevotella	42.819	-0.590	0.474	6.215e-01
Aureimonas	24.555	-0.633	0.508	6.215e-01
Geodermatophilus	37.591	-0.685	0.551	6.229e-01
Lentisphaera	29.992	0.323	0.264	6.229e-01
Leptotrichia	40.411	0.404	0.329	6.308e-01
Planococcus	196.283	0.237	0.194	6.326e-01
Reyranella	10.238	-0.686	0.568	6.326e-01
Gemella	167.060	0.501	0.413	6.358e-01
Labrys	96.739	-0.463	0.382	6.358e-01
Dyadobacter	16.940	-0.562	0.465	6.358e-01
Thermomonas	34.874	-0.314	0.261	6.358e-01
Knoellia	9.989	0.838	0.688	6.358e-01
Chelativorans	10.320	-1.018	0.801	6.358e-01
Fibroporia	6.292	1.336	1.078	6.358e-01
Caballeronia	39.742	-0.275	0.228	6.375e-01
Aureibaculum	51.891	-0.438	0.367	6.378e-01
Fomitiporia	9.122	0.948	0.785	6.378e-01
Leptolyngbya	41.961	-0.476	0.402	6.422e-01
Kribbella	6.342	-0.665	0.562	6.430e-01
Dermacoccus	44.902	0.357	0.302	6.438e-01
Glycocaulis	61.217	-1.302	1.088	6.438e-01
Taibaiella	11.521	-0.937	0.779	6.438e-01
Segatella	9.943	-0.670	0.576	6.438e-01
Erwinia	905.375	-0.347	0.297	6.472e-01
Rugamonas	25.020	-0.485	0.414	6.472e-01
Oceanobacillus	36.670	0.325	0.283	6.472e-01
Collinsella	12.119	-0.986	0.826	6.473e-01
Roseovarius	20.641	0.401	0.352	6.479e-01
Paenimyroides	6.925	1.116	0.955	6.513e-01
Phanerochaete	11.292	-0.935	0.813	6.515e-01
Chryseobacterium	293.101	-0.236	0.206	6.544e-01
Dietzia	166.910	-0.377	0.327	6.544e-01
Frigidibacter	67.900	0.323	0.281	6.544e-01
Mucilaginibacter	56.373	-0.337	0.298	6.618e-01
Amnimonas	7.614	1.244	1.076	6.618e-01
Methylobacterium	5697.290	0.201	0.182	6.715e-01
Rubellimicrobium	30.281	0.594	0.533	6.715e-01

Nocardia	68.412	-0.249	0.225	6.715e-01
Scytonema	22.749	-0.534	0.481	6.715e-01
Exophiala	31.121	0.639	0.575	6.715e-01
Pseudoroseomonas	11.878	-0.679	0.590	6.715e-01
Candidate	14.431	0.391	0.350	6.715e-01
Belnapia	18.847	0.682	0.612	6.715e-01
Citromicrobium	6.385	-0.888	0.819	6.715e-01
Neomicrococcus	5.484	-0.725	0.687	6.715e-01
Gryllotalpicola	6.324	1.109	0.994	6.715e-01
Salmonella	451.456	-0.175	0.161	6.765e-01
Xanthomonas	75.563	-0.319	0.293	6.765e-01
Fluoribacter	203.940	-0.297	0.273	6.765e-01
Lentzea	21.126	-0.322	0.300	6.765e-01
Rhizoctonia	10.173	-0.892	0.827	6.765e-01
Sinorhizobium	5.189	-0.819	0.800	6.765e-01
Bradyrhizobium	4376.605	0.188	0.177	6.852e-01
Burkholderia	2610.408	0.161	0.151	6.852e-01
Moesziomyces	19.781	-0.959	0.900	6.852e-01
Lautropia	241.645	0.218	0.204	6.852e-01
Duganella	52.030	0.360	0.339	6.852e-01
Rathayibacter	27.104	0.678	0.635	6.852e-01
Peredibacter	28.482	-0.605	0.571	6.852e-01
Quadrisphaera	13.743	-0.934	0.865	6.852e-01
Salinicoccus	15.586	-0.688	0.636	6.852e-01
Ilumatobacter	9.966	-1.190	1.092	6.852e-01
Loktanella	12.504	-0.277	0.262	6.852e-01
Methylosarcina	17.576	-0.607	0.571	6.864e-01
Aliterella	5.985	-1.022	1.020	6.899e-01
Cutibacterium	314.810	0.297	0.285	6.958e-01
Deinococcus	166.493	-0.160	0.154	7.002e-01
Plasmodium	260.330	-0.171	0.166	7.037e-01
Xylophilus	6.954	0.507	0.505	7.046e-01
Zoogloea	42.077	-0.354	0.349	7.056e-01
Spiribacter	38.734	0.426	0.420	7.138e-01
Sphingobacterium	129.321	-0.308	0.306	7.151e-01
Phaeodactylum	12.627	-0.524	0.525	7.151e-01
Mus_musculus_mobilized_virus	23.218	0.314	0.324	7.151e-01
Marinomonas	8.179	0.933	0.927	7.156e-01
Thermalbibacter	6.902	1.006	1.000	7.156e-01
Mycetohabitans	9.753	-0.446	0.449	7.156e-01
Afipia	1012.669	0.241	0.243	7.161e-01
Morococcus	313.297	0.270	0.272	7.161e-01
Meiothermus	30.189	0.556	0.557	7.161e-01
Cryptosporidium	15.505	-0.491	0.491	7.161e-01
Pauljensenia	155.649	0.428	0.434	7.205e-01
Betaproteobacterium_FWI2	1603.185	0.331	0.338	7.222e-01
Leucobacter	21.363	0.386	0.394	7.222e-01
Naegleria	20.303	0.474	0.488	7.258e-01
Virgibacillus	35.407	-0.254	0.268	7.273e-01
Pseudoramibacter	88.151	0.491	0.507	7.302e-01
Sphingopyxis	66.886	-0.381	0.394	7.315e-01
Pseudacidovorax	63.479	0.292	0.308	7.368e-01
Bifidobacterium	47.754	0.323	0.339	7.368e-01
Dyella	23.540	-0.331	0.348	7.368e-01
Tepidicella	11.802	0.700	0.730	7.368e-01
Prevotella	338.175	-0.284	0.303	7.416e-01
Parabacteroides	28.004	0.319	0.340	7.416e-01
Tetragenococcus	7.371	0.797	0.837	7.416e-01
Solemya	6.351	-0.598	0.670	7.449e-01

Pandoraea	33.582	0.293	0.314	7.460e-01
Tessaracoccus	17.444	-0.594	0.640	7.460e-01
Megasphaera	17.752	0.623	0.666	7.460e-01
Ottowia	13.988	-0.441	0.475	7.460e-01
Babesia	6.351	-0.567	0.647	7.460e-01
Curvibacter	191.180	-0.134	0.147	7.616e-01
Rhodococcus	464.902	0.163	0.182	7.674e-01
Paenirhodobacter	23.041	0.308	0.351	7.674e-01
Aurantiacibacter	6.751	-0.674	0.793	7.674e-01
Sinirhodobacter	15.801	0.119	0.331	7.674e-01
Nevskia	5.606	0.960	1.059	7.674e-01
Clavispora	11.624	-1.314	1.465	7.674e-01
Aquabacterium	1382.076	0.215	0.243	7.697e-01
Bacteroides	124.758	-0.203	0.232	7.697e-01
Westeberhardia	22.020	-0.361	0.417	7.697e-01
Schlegelella	25.036	-0.382	0.435	7.697e-01
Daldinia	6.504	0.930	1.046	7.697e-01
Herbiconiux	5.773	0.882	0.980	7.697e-01
Rheinheimera	4037.545	-0.228	0.261	7.734e-01
Gluconobacter	9.302	0.547	0.635	7.786e-01
Kluyvera	247.288	-0.161	0.188	7.796e-01
Metarhizium	16.962	0.472	0.545	7.796e-01
Besnoitia	7.844	0.358	0.429	7.796e-01
Capnocytophaga	41.728	0.256	0.302	7.886e-01
Kaistella	17.043	-0.404	0.478	7.902e-01
Terrabacter	109.045	0.196	0.235	7.905e-01
Sphingosinicella	7.978	-0.637	0.777	7.905e-01
Abiotrophia	14.388	-0.511	0.608	7.912e-01
Brevundimonas	1147.754	-0.195	0.236	7.928e-01
Methylorubrum	226.898	0.141	0.170	7.928e-01
Brachybacterium	121.244	0.336	0.407	7.928e-01
Synechocystis	16.400	-0.547	0.661	7.928e-01
Comamonadaceae_genus	99.630	-0.197	0.238	7.928e-01
Polaromonas	23.598	0.246	0.299	7.928e-01
Phocaeicola	15.829	-0.511	0.616	7.928e-01
Photobacterium	11.366	-0.394	0.475	7.928e-01
Chromobacterium	7.322	-0.405	0.511	7.928e-01
Klebsiella	1531.102	-0.184	0.226	7.958e-01
Salinibacterium	8.132	-0.486	0.620	7.958e-01
Xanthobacter	6.049	-0.494	0.647	7.958e-01
Limosilactobacillus	14.701	-0.344	0.433	7.999e-01
Malassezia	1176.135	0.227	0.284	8.058e-01
Pseudoxanthomonas	75.899	-0.239	0.299	8.058e-01
Tissierella	290.882	0.149	0.190	8.110e-01
Peptostreptococcus	6.314	0.528	0.669	8.112e-01
Planktothrix	6.905	-0.492	0.626	8.120e-01
Endocarpon	5.929	-0.544	0.656	8.144e-01
Algoriphagus	22.246	-0.259	0.331	8.146e-01
Propionibacterium	5198.439	0.220	0.283	8.151e-01
Atopomonas	30.225	0.214	0.279	8.151e-01
Diaporthe	5.499	-0.916	1.081	8.180e-01
Bartonella	9.625	-0.428	0.556	8.191e-01
Seonamhaeicola	12.679	0.286	0.416	8.191e-01
Leclercia	50.086	-0.178	0.234	8.218e-01
Dictyostelium	8.462	0.612	0.801	8.218e-01
Polymorphobacter	8.366	-0.930	0.838	8.218e-01
Janibacter	151.607	0.138	0.186	8.218e-01
Geminicoccus	65.368	-0.167	0.231	8.218e-01
Marinifilum	38.998	-0.193	0.259	8.218e-01

Methylotenera	11.956	0.481	0.648	8.218e-01
Tetrahymena	5.684	0.508	0.665	8.218e-01
Barnesiella	9.605	-0.271	0.370	8.218e-01
Pinisolibacter	11.572	0.153	0.279	8.218e-01
Moorena	7.922	-0.602	0.836	8.218e-01
Motilimonas	7.460	0.391	0.534	8.218e-01
Mannheimia	5.616	-0.366	0.527	8.218e-01
Winogradskyella	5.235	0.021	0.380	8.218e-01
Cryptococcus	8.210	-0.600	0.814	8.242e-01
Coraliihabitans	7.777	0.427	0.587	8.323e-01
Desemzia	16.346	-0.370	0.510	8.323e-01
Allomeiothermus	372.615	0.418	0.599	8.378e-01
Bavariicoccus	65.404	-0.227	0.323	8.378e-01
Propionimicrobium	58.601	0.317	0.452	8.378e-01
Rhodobacter	87.074	-0.174	0.249	8.378e-01
Alpha	67.476	-0.198	0.284	8.378e-01
Rhodoferax	44.739	-0.226	0.317	8.378e-01
Noviherbaspirillum	23.982	-0.482	0.684	8.378e-01
Empedobacter	31.761	0.260	0.370	8.378e-01
Solihabitans	20.875	0.147	0.262	8.378e-01
Pimelobacter	6.825	-0.501	0.711	8.378e-01
Alkanindiges	5.454	-0.528	0.703	8.378e-01
Betaproteobacterium_AAP51	11.054	0.383	0.547	8.378e-01
Sulfitobacter	6.435	0.330	0.458	8.378e-01
Lichtheimia	16.959	-0.253	0.371	8.393e-01
Tamlana	6.081	-0.298	0.453	8.394e-01
Pedobacter	122.644	-0.184	0.268	8.416e-01
Nanosynbacter	12.801	0.431	0.634	8.442e-01
Asticcacaulis	114.652	-0.274	0.403	8.469e-01
Levyella	7.688	0.766	1.127	8.469e-01
Tepidimonas	39.113	0.334	0.495	8.491e-01
Herbaspirillum	591.387	0.136	0.203	8.512e-01
Piscinibacter	118.721	0.240	0.360	8.512e-01
Bacterium	36.502	-0.129	0.236	8.512e-01
Rhodotorula	32.855	-0.364	0.551	8.530e-01
Pararhodobacter	5.193	-0.515	0.787	8.530e-01
Yimella	7.431	-0.461	0.728	8.541e-01
Bacillus	2979.064	0.071	0.120	8.558e-01
Clostridium	1001.246	-0.109	0.188	8.558e-01
Stenotrophomonas	1057.148	-0.065	0.113	8.558e-01
Arthrobacter	566.114	-0.108	0.183	8.558e-01
Shewanella	593.636	0.095	0.146	8.558e-01
Enterobacteriaceae_genus	24.824	0.158	0.285	8.558e-01
Aequorivita	187.348	0.117	0.193	8.558e-01
Azospirillum	271.311	0.196	0.341	8.558e-01
Agrobacterium	192.449	0.106	0.190	8.558e-01
Leptothrix	258.760	0.200	0.333	8.558e-01
Armatimonadetes	144.045	0.421	0.646	8.558e-01
Thermus	128.332	0.250	0.409	8.558e-01
Levilactobacillus	89.361	0.288	0.471	8.558e-01
Lactococcus	112.493	0.242	0.400	8.558e-01
Gordonia	74.952	-0.214	0.359	8.558e-01
Acidipropionibacterium	19.534	-0.333	0.559	8.558e-01
Baudoinia	11.960	-0.306	0.468	8.558e-01
Kosakonia	21.067	-0.224	0.373	8.558e-01
Granulicatella	27.984	0.256	0.436	8.558e-01
Enhydrobacter	51.501	-0.171	0.282	8.558e-01
Mycobacteriaceae_genus	41.727	0.121	0.226	8.558e-01
Ensifer	36.688	-0.178	0.309	8.558e-01

Gallibacter	13.089	-0.497	0.786	8.558e-01
Thauera	36.207	-0.149	0.237	8.558e-01
Ligilactobacillus	14.208	0.384	0.636	8.558e-01
Kineococcus	53.880	0.400	0.673	8.558e-01
Lasiodiplodia	27.457	-0.218	0.352	8.558e-01
Postia	8.620	0.462	0.792	8.558e-01
Parasphingopyxis	15.233	0.369	0.633	8.558e-01
Azohydromonas	15.191	0.296	0.480	8.558e-01
Fluviicola	6.799	0.496	0.835	8.558e-01
Desertimonas	10.044	0.540	0.887	8.558e-01
Cercospora	12.008	0.335	0.540	8.558e-01
Eubacterium	18.755	-0.236	0.399	8.558e-01
Methylobrevis	19.272	0.243	0.411	8.558e-01
Dioszegia	17.319	-0.538	0.938	8.558e-01
Burkholderiales	19.138	-0.261	0.425	8.558e-01
Gaiella	11.733	0.560	0.963	8.558e-01
Conchiformibius	7.052	0.486	0.786	8.558e-01
Cyclobacterium	9.163	0.180	0.363	8.558e-01
Agathobacter	7.239	-0.611	1.010	8.558e-01
Aureococcus	5.453	-0.501	0.845	8.558e-01
Pyrococcus	12.784	-0.329	0.576	8.558e-01
Murine_type_C_virus	8.935	0.192	0.401	8.558e-01
Glarea	5.933	-0.419	0.716	8.558e-01
Homoserinimonas	5.839	0.439	0.745	8.558e-01
Aphanomyces	5.189	0.387	0.589	8.558e-01
Solimonas	5.862	0.496	0.866	8.558e-01
Gleimia	229.791	-0.223	0.395	8.610e-01
Tatumella	7.235	-0.342	0.602	8.610e-01
Polyangium	64.134	-0.309	0.546	8.610e-01
Pseudozyma	9.591	-0.397	0.717	8.611e-01
Aureobasidium	12.765	0.328	0.584	8.611e-01
Staphylococcus	2266.839	0.157	0.310	8.686e-01
Comamonas	377.215	0.111	0.215	8.686e-01
Roseateles	324.440	0.092	0.174	8.686e-01
Sphingobium	158.875	0.099	0.194	8.686e-01
Halomonas	158.342	-0.133	0.265	8.686e-01
Entamoeba	33.993	-0.238	0.478	8.686e-01
Proteus	179.703	-0.178	0.348	8.686e-01
Luteimonas	79.273	-0.273	0.533	8.686e-01
Rhizorhabdus	68.045	-0.153	0.294	8.686e-01
Phycicoccus	66.900	-0.134	0.256	8.686e-01
Leifsonia	76.614	0.209	0.381	8.686e-01
Thiohalocapsa	29.701	0.165	0.326	8.686e-01
Skermanella	18.493	0.270	0.538	8.686e-01
Roseburia	27.841	-0.140	0.260	8.686e-01
Beggiatoa	32.646	0.125	0.242	8.686e-01
Tumebacillus	31.849	0.201	0.384	8.686e-01
Lachnospiraceae_genus	22.274	-0.240	0.446	8.686e-01
Aquincola	15.393	-0.238	0.491	8.686e-01
Coniophora	9.959	-0.517	1.022	8.686e-01
Shinella	19.684	-0.278	0.541	8.686e-01
Ruminococcaceae_genus	14.329	0.197	0.374	8.686e-01
Sandaracinobacteroides	6.329	0.394	0.760	8.686e-01
Paucilactobacillus	8.021	0.281	0.552	8.686e-01
Pyrenophora	5.376	-0.404	0.878	8.686e-01
Betaproteobacterium_JGI	8.316	-0.215	0.423	8.686e-01
Amycolatopsis	5.663	-0.254	0.507	8.686e-01
Pigmentiphaga	5.865	-0.353	0.740	8.686e-01
Desulfitobacterium	6.033	0.402	0.774	8.686e-01

Thiobacillus	5.354	-0.358	0.767	8.686e-01
Human_adenovirus_2	5.562	-0.373	0.748	8.686e-01
Terracoccus	15.641	-0.391	0.781	8.699e-01
Streptococcus	1476.802	0.146	0.295	8.714e-01
Enterovirga	5.894	-0.432	0.951	8.717e-01
Bowmanella	57.320	-0.160	0.328	8.719e-01
Labilibaculum	7.087	-0.250	0.488	8.719e-01
Bifidobacteriaceae_genus	8.416	0.444	0.910	8.739e-01
Alteribacter	6.236	-0.178	0.408	8.739e-01
Sphingomonas	2004.426	-0.062	0.130	8.751e-01
Shimia	358.445	0.143	0.301	8.751e-01
Chromatium	57.450	0.099	0.208	8.751e-01
Azospira	27.304	0.131	0.311	8.751e-01
Limnohabitans	19.440	0.157	0.341	8.751e-01
Ichthyophthirius	11.762	0.230	0.507	8.751e-01
Atlantibacter	14.246	0.126	0.266	8.751e-01
Micropruina	6.588	-0.452	0.968	8.751e-01
Abditibacterium	5.942	0.464	0.961	8.751e-01
Rubrivivax	73.057	0.154	0.340	8.784e-01
Psychromicrobium	11.336	-0.125	0.299	8.784e-01
Desulfosporosinus	5.604	-0.266	0.662	8.784e-01
Salinimicrobium	20.796	0.181	0.463	8.789e-01
Kwoniella	6.912	-0.426	0.935	8.789e-01
Edwardsiella	64.525	-0.148	0.329	8.826e-01
Orbilia	10.608	0.383	0.849	8.826e-01
Brucella	13.265	0.193	0.432	8.834e-01
Phaeovulum	30.793	-0.137	0.313	8.841e-01
Halovibrio	10.136	-0.280	0.622	8.841e-01
Aphanothece	13.799	0.187	0.427	8.852e-01
Moritella	186.504	0.069	0.166	8.863e-01
Moraxellaceae_genus	14.760	-0.243	0.557	8.863e-01
Aurantimonas	8.757	-0.232	0.547	8.863e-01
Acidovorax	744.728	0.046	0.109	8.871e-01
Rubrobacter	16.449	-0.268	0.634	8.871e-01
Dysgonamonadaceae_genus	13.608	0.307	0.721	8.871e-01
Solobacterium	7.141	-0.267	0.678	8.871e-01
Caldimonas	5.324	-0.362	0.854	8.871e-01
Marinobacter	150.235	-0.104	0.245	8.880e-01
Colletotrichum	46.845	0.147	0.355	8.880e-01
Gardnerella	26.116	0.242	0.583	8.880e-01
Sedimentitalea	30.138	0.030	0.268	8.880e-01
Thioflexithrix	10.033	0.180	0.450	8.880e-01
Thalassobius	5.691	-0.284	0.746	8.880e-01
Spirosoma	11.388	0.249	0.611	8.916e-01
Microbacterium	1220.119	0.055	0.137	8.934e-01
Hoylesella	15.245	-0.221	0.557	8.934e-01
Nocardiopsis	6.413	0.397	0.966	8.934e-01
Hyphomicrobium	34.815	0.116	0.292	8.960e-01
Klenkia	7.551	0.270	0.725	8.960e-01
Nostoc	288.872	0.078	0.198	8.994e-01
Cedecea	62.532	-0.154	0.404	9.036e-01
Lentibacillus	34.801	0.096	0.264	9.036e-01
Ruminococcus	28.348	-0.183	0.485	9.070e-01
Cupriavidus	310.513	0.074	0.202	9.108e-01
Microlunatus	62.692	-0.149	0.410	9.108e-01
Aggregatibacter	18.736	0.151	0.427	9.108e-01
Amorphotheca	7.737	-0.244	0.696	9.108e-01
Kallotenue	98.738	0.211	0.578	9.112e-01
Marinithermofilum	29.006	0.091	0.264	9.112e-01

Achromobacter	178.806	-0.046	0.132	9.151e-01
Mobilicoccus	15.933	-0.204	0.572	9.151e-01
Olsenella	7.411	0.289	0.798	9.151e-01
Agromyces	13.851	-0.146	0.415	9.154e-01
Alcanivorax	267.141	0.078	0.230	9.168e-01
Serinicoccus	13.203	0.259	0.757	9.168e-01
Luteitalea	7.610	0.317	0.901	9.168e-01
Winkia	10.745	0.268	0.772	9.168e-01
Nitrobacter	5.995	-0.201	0.659	9.168e-01
Musicola	448.106	-0.119	0.355	9.189e-01
Diaphorobacter	35.714	0.124	0.379	9.189e-01
Hammondia	7.312	-0.126	0.477	9.189e-01
Bhargavaea	9.142	0.177	0.600	9.189e-01
Flavobacterium	706.963	0.049	0.155	9.195e-01
Rahnella	196.146	-0.098	0.300	9.195e-01
Candida	5.852	0.012	0.625	9.195e-01
Jeotgalicoccus	20.422	0.108	0.374	9.195e-01
Kurthia	83.993	0.107	0.347	9.195e-01
Neobacillus	44.891	0.070	0.242	9.195e-01
Actinomycetospora	16.751	0.152	0.510	9.195e-01
Myxococcus	31.942	0.015	0.261	9.195e-01
Plasmopara	6.866	-0.197	0.683	9.195e-01
Rhodopseudomonas	31.634	0.114	0.351	9.195e-01
Acidiplasma	17.926	0.103	0.333	9.195e-01
Pararhizobium	9.293	-0.202	0.650	9.195e-01
UNVERIFIED_ORG:	13.458	-0.143	0.482	9.195e-01
Brachymonas	13.279	0.143	0.443	9.195e-01
Microcoleus	16.615	0.128	0.551	9.195e-01
Zimmermannella	7.604	-0.240	0.768	9.195e-01
Rhodoplanes	8.916	-0.162	0.556	9.195e-01
Alcaligenes	8.410	0.198	0.605	9.195e-01
Gluconacetobacter	6.748	-0.154	0.574	9.195e-01
Mangrovicoccus	5.222	0.278	0.898	9.195e-01
Serratia	233.668	-0.074	0.250	9.201e-01
Pedococcus	6.046	-0.193	0.703	9.201e-01
Lacticaseibacillus	6.204	0.206	0.664	9.213e-01
Thermoanaerobacterium	21.481	0.273	0.953	9.276e-01
Terrisporobacter	682.815	0.088	0.323	9.310e-01
Mesorhizobium	79.330	-0.052	0.194	9.310e-01
Paraclostridium	10.370	0.192	0.695	9.310e-01
Chitinophaga	7.848	0.170	0.609	9.310e-01
Pseudoclavibacter	10.937	-0.168	0.612	9.310e-01
Enterobacter	9658.205	-0.076	0.288	9.315e-01
Elizabethkingia	205.340	-0.071	0.269	9.315e-01
Fusobacterium	181.144	0.053	0.212	9.315e-01
Wolbachia	68.134	-0.105	0.407	9.315e-01
Isoptericola	31.305	0.019	0.237	9.315e-01
Neoantrodia	9.542	0.226	0.874	9.315e-01
Pseudokineococcus	5.519	0.354	1.238	9.315e-01
Seramator	7.459	-0.171	0.736	9.315e-01
Thalassiosira	9.266	0.139	0.575	9.315e-01
Bosea	160.841	0.032	0.189	9.317e-01
Amaricoccus	20.347	-0.143	0.558	9.317e-01
Porphyromonadaceae_genus	12.235	-0.154	0.625	9.330e-01
Beijerinckiaceae_genus	37.739	-0.172	0.701	9.345e-01
Parainfluenza_virus_5	6.913	0.293	1.137	9.345e-01
Erythrobacter	135.550	-0.032	0.227	9.345e-01
Halalkalibacter	112.475	-0.071	0.304	9.345e-01
Marmoricola	34.294	-0.124	0.544	9.345e-01

Microbispora	62.361	-0.061	0.302	9.345e-01
Maribellus	40.625	0.074	0.345	9.345e-01
Prevotellaceae_genus	6.860	-0.134	0.676	9.345e-01
Puia	26.661	-0.050	0.296	9.345e-01
Pestalotiopsis	18.985	-0.111	0.520	9.345e-01
Crocosphaera	13.832	0.088	0.398	9.345e-01
Bipolaris	7.199	0.179	0.781	9.345e-01
Pseudorhodoferax	8.912	0.151	0.650	9.345e-01
Renibacterium	7.693	-0.215	0.785	9.345e-01
Arachnia	16.113	-0.121	0.551	9.350e-01
Williamsia	113.724	0.100	0.447	9.361e-01
Selenomonas	7.222	0.089	0.547	9.361e-01
Actinomyces	520.914	-0.053	0.241	9.375e-01
Pusillimonas	1729.460	-0.055	0.260	9.385e-01
Photorhabdus	47.052	0.059	0.368	9.385e-01
Falsiroseomonas	137.429	0.049	0.254	9.385e-01
Chromohalobacter	65.014	0.036	0.210	9.385e-01
Xenophilus	37.016	-0.063	0.327	9.385e-01
Fonsecaea	17.304	-0.119	0.587	9.385e-01
Solibacillus	6.613	-0.097	0.649	9.385e-01
Ruegeria	9.018	0.123	0.689	9.407e-01
Longimicrobium	6.592	0.218	1.017	9.407e-01
Actinoplanes	35.115	0.070	0.366	9.411e-01
Stutzerimonas	32.547	-0.086	0.442	9.411e-01
Tetrasphaera	10.317	-0.132	0.666	9.411e-01
Methylibium	9.563	0.163	0.830	9.411e-01
Citrobacter	14367.687	-0.054	0.290	9.419e-01
Aerococcus	77.226	0.050	0.287	9.419e-01
Raoultella	68.520	-0.053	0.319	9.419e-01
Dermabacter	5.640	-0.140	0.884	9.419e-01
Pyrinomonas	9.505	0.160	0.879	9.419e-01
Escherichia	61277.548	0.053	0.298	9.458e-01
Frankia	114.940	0.048	0.284	9.458e-01
Cupidesulfovibrio	28.579	0.064	0.402	9.458e-01
Ancylomarina	7.570	0.037	0.560	9.458e-01
Methyloversatilis	103.042	-0.045	0.276	9.506e-01
Eikenella	9.058	-0.089	0.566	9.512e-01
Kushneria	83.057	0.003	0.276	9.514e-01
Oryzomicrobium	63.319	-0.064	0.414	9.514e-01
Leuconostoc	27.068	-0.051	0.320	9.514e-01
Actinokineospora	43.083	-0.080	0.529	9.557e-01
Algibacter	5.117	0.019	0.550	9.557e-01
Kingella	8.778	-0.017	0.513	9.572e-01
Trypanosoma	14.029	0.042	0.551	9.572e-01
Lactobacillus	101.572	-0.028	0.200	9.583e-01
Rothia	240.951	-0.033	0.256	9.597e-01
Craterilacuibacter	127.435	-0.066	0.492	9.597e-01
Blochmannia	14.483	-0.081	0.704	9.597e-01
Pseudochrobactrum	75.819	0.055	0.452	9.597e-01
Kytococcus	23.988	0.061	0.491	9.597e-01
Methylophilus	8.689	-0.059	0.539	9.597e-01
Human_endogenous_retrovirus	5.191	0.059	0.536	9.597e-01
Shigella	210548.549	0.038	0.302	9.618e-01
Frigoribacterium	15.155	0.075	0.629	9.654e-01
Tannerella	323.303	-0.034	0.322	9.665e-01
Phytophthora	90.681	-0.045	0.427	9.665e-01
Ruficoccus	65.274	0.036	0.352	9.665e-01
Cardiobacterium	13.923	0.064	0.565	9.665e-01
Fredinandcohnia	6.991	-0.059	0.605	9.665e-01

Rhodomicrobium	7.805	0.028	0.460	9.665e-01
Nitrosomonas	5.354	0.119	0.802	9.665e-01
Telluria	20.994	-0.041	0.509	9.666e-01
Rhizobiales	18.512	-0.014	0.457	9.669e-01
Halorubrum	12.131	0.019	0.426	9.669e-01
Rhodanobacter	6.156	-0.064	0.631	9.669e-01
Cytobacillus	13.792	0.112	1.179	9.682e-01
Marinobacterium	1297.016	-0.034	0.360	9.683e-01
Leucothrix	10.440	0.015	0.474	9.683e-01
Toxoplasma	250.863	0.012	0.152	9.712e-01
Allobacillus	31.886	-0.025	0.362	9.712e-01
Dolosigranulum	11.557	0.055	0.681	9.712e-01
Glaciecola	6.760	-0.019	0.360	9.712e-01
Pseudopropionibacterium	6.216	0.016	0.682	9.712e-01
Perkinsus	5.227	0.081	0.585	9.712e-01
Paracidovorax	147.082	-0.018	0.324	9.726e-01
Oribacterium	16.999	-0.034	0.590	9.726e-01
Calidithermus	19.574	0.040	0.538	9.726e-01
Microcystis	7129.664	-0.022	0.366	9.735e-01
Actinobacillus	273.019	0.016	0.342	9.735e-01
Izhakiella	15.037	0.009	0.840	9.735e-01
Actinotalea	15.791	0.040	0.700	9.735e-01
Aquamicrobium	62.955	-0.014	0.335	9.735e-01
Gloeophyllum	13.463	-0.068	0.985	9.735e-01
Schaalia	24.329	-0.005	0.464	9.735e-01
Glaciibacter	18.801	0.039	0.656	9.735e-01
Treponema	11.715	-0.011	0.512	9.735e-01
Atopobium	11.220	0.034	0.646	9.735e-01
Kinneretia	11.622	-0.004	0.507	9.735e-01
Yarrowia	13.249	0.043	0.776	9.735e-01
Methylopila	6.130	-0.060	0.842	9.735e-01
Cellulomonas	41.380	-0.011	0.339	9.738e-01
Paracoccus	735.536	0.005	0.172	9.739e-01
Blastomonas	151.122	-0.007	0.289	9.739e-01
Streptosporangium	49.775	0.005	0.237	9.739e-01
Phytobacter	11.988	-0.016	0.451	9.748e-01
Actinomadura	901.640	0.006	0.151	9.756e-01
Acinetobacter	11818.492	0.005	0.154	9.802e-01
Bordetella	18.960	0.011	0.384	9.802e-01
TM7	7.135	-0.012	0.647	9.802e-01
Lamprocystis	7995.423	0.005	0.330	9.890e-01
Fictibacillus	317.200	-0.005	0.347	9.890e-01
Arsenophonus	1.327	0.340	0.616	NA
Type-C_symbiont_of_Plautia_stali	0.212	0.094	2.743	NA
Sodalis-like	0.331	-0.411	0.977	NA
Bacteria	0.118	1.194	0.927	NA
Rhizopus	1.598	-2.494	1.482	NA
Type-D_symbiont_of_Plautia_stali	2.107	-3.637	2.900	NA
Motilibacter	2.373	0.738	1.451	NA
Type-F_symbiont_of_Plautia_stali	0.400	-0.627	2.906	NA
Chaetomium	2.688	0.038	1.050	NA
Endozoicomonas	0.368	1.410	1.263	NA
Turicibacter	4.462	-1.962	1.133	NA
Profftia	0.070	1.695	2.912	NA
Plautia	0.323	-0.884	1.579	NA
Type-E_symbiont_of_Plautia_stali	0.036	0.975	2.913	NA
Annandia	0.084	1.144	2.073	NA
Schizosaccharomyces	3.316	-0.456	1.135	NA
Aedoeadaptatus	2.659	0.153	1.331	NA

Desarmillaria	3.626	-0.157	0.811	NA
Flavisolibacter	1.622	-0.907	1.236	NA
Cronobacter	1.550	-0.481	0.946	NA
Emericellopsis	3.788	-1.491	1.494	NA
Hafnia	2.018	0.482	0.468	NA
Sodalis	1.787	-0.324	0.400	NA
Paludisphaera	3.132	0.552	0.835	NA
Trabulsiella	0.462	-0.166	0.595	NA
Singulisphaera	4.308	-0.352	0.833	NA
Phreatobacter	3.203	0.503	0.877	NA
Tissierellia	3.515	-0.516	1.140	NA
Ethanoligenens	2.089	3.800	1.808	NA
Phycomyces	1.988	-0.616	1.142	NA
Chelatococcus	4.743	0.752	0.909	NA
Lelliottia	2.324	0.436	0.625	NA
Cnuella	0.966	1.439	1.736	NA
Weizmannia	1.405	-0.632	2.213	NA
Aquisphaera	1.528	0.114	1.228	NA
Radiomyces	1.508	-2.269	1.183	NA
Tautonia	3.902	-1.686	1.544	NA
Lactiplantibacillus	4.583	-0.343	0.544	NA
Scedosporium	0.752	-0.570	1.715	NA
Thermothelomyces	0.628	-0.204	1.341	NA
Paracnuella	0.644	0.041	1.253	NA
Herbinix	4.473	0.907	1.377	NA
Latilactobacillus	2.108	-0.228	1.306	NA
Sugiyamaella	3.235	-2.523	1.171	NA
Pseudactinotalea	0.117	-0.017	2.911	NA
Mangrovibacter	0.437	0.366	0.663	NA
Fenollaria	1.816	-1.331	1.548	NA
Thiothrix	0.796	3.819	2.423	NA
Calothrix	3.097	0.840	0.924	NA
Pleurotus	2.254	-0.687	1.093	NA
Mikella	0.000	0.000	0.000	NA
Gullanella	0.959	-0.583	1.008	NA
Fulvia	3.461	-0.726	0.670	NA
Promicromonospora	1.177	-0.101	1.694	NA
Flaviaesturariibacter	0.706	2.229	2.252	NA
Wenzhouxiangella	3.178	-0.561	0.810	NA
Phialophora	4.587	-0.599	0.944	NA
Dacryopinax	2.313	-0.122	1.332	NA
Heyndrickxia	3.581	2.607	1.327	NA
Ogataea	1.444	0.554	1.314	NA
Humisphaera	4.666	-2.195	1.303	NA
Planctomyces	2.081	-0.094	1.041	NA
Caulobacteraceae_genus	1.255	0.559	1.906	NA
Prosthecobacter	3.876	-0.848	0.998	NA
Kalmanozyma	4.191	-1.000	0.795	NA
Ishikawaella	0.000	0.000	0.000	NA
Paracoccidioides	3.834	-1.339	0.830	NA
Haliangium	2.455	-0.052	1.475	NA
Xenorhabdus	3.486	-0.182	0.422	NA
Dickeya	1.888	0.754	0.418	NA
Phaeoacremonium	2.333	1.314	0.659	NA
Halopseudomonas	3.486	-1.101	0.873	NA
Hafniaceae_genus	0.000	0.000	0.000	NA
Gammaproteobacteria	2.722	0.844	0.976	NA
Oceanospirillum	3.681	-0.162	0.599	NA
Varibaculum	3.474	-0.182	0.971	NA

Pseudogymnoascus	4.453	0.849	0.936	NA
Lachnellula	2.618	0.116	0.755	NA
Pleomorphomonas	4.145	-1.856	1.137	NA
Labilithrix	4.216	-0.555	1.034	NA
Methylosinus	0.957	-1.034	1.065	NA
Caldibacillus	3.835	0.826	0.811	NA
Lacrimispora	0.872	0.204	1.722	NA
Grosmannia	0.859	1.432	1.527	NA
Pluralibacter	1.241	-2.166	1.300	NA
Pseudoduganella	4.710	-0.855	0.686	NA
Flavihumibacter	2.570	0.958	0.824	NA
Pectobacterium	2.621	0.933	0.525	NA
Putridiphycobacter	3.881	-0.826	0.793	NA
Brettanomyces	3.564	-3.069	1.398	NA
Bathymodiolus	0.021	1.511	2.913	NA
Segetibacter	3.596	0.412	1.245	NA
Arsukibacterium	0.805	0.452	1.168	NA
Hoaglandella	0.398	0.235	2.161	NA
Microdochium	4.060	-0.303	0.752	NA
Gilliamella	0.563	1.731	1.151	NA
Aquihabitans	4.214	0.031	1.078	NA
Rhodofomes	4.456	0.068	0.807	NA
Enteractinococcus	1.267	0.726	2.201	NA
Pediococcus	1.064	-0.638	1.088	NA
Terrihabitans	1.239	-1.844	2.024	NA
Xylanimonas	3.351	-1.018	0.961	NA
Superficieibacter	0.163	1.674	1.121	NA
Rosenbergiella	1.613	0.284	0.697	NA
Sporichthya	4.613	-0.218	1.039	NA
Intestinirhabdus	0.996	-0.137	0.421	NA
Alicyclobacillus	0.445	-0.146	2.171	NA
Shigella_phage_SfIV_virus	0.000	0.000	0.000	NA
Macrococcus	3.784	0.553	0.724	NA
Limimaricola	3.153	-0.145	1.378	NA
Jeotgalibacillus	1.208	0.527	1.048	NA
Oceaniovalibus	4.526	-0.065	0.713	NA
Moranella	0.333	2.383	2.499	NA
Botrytis	2.262	0.977	1.080	NA
Pleomorpha	4.324	-0.823	0.905	NA
Laccaria	3.822	0.277	0.732	NA
Blastomyces	2.516	-1.274	0.903	NA
Glaciihabitans	1.333	-0.426	1.414	NA
Zygosaccharomyces	0.127	1.001	2.912	NA
Bacidia	3.842	0.412	0.895	NA
Oxalobacteraceae_genus	2.465	-0.657	0.872	NA
Vallicoccus	2.925	-0.930	1.250	NA
Gemmatimonas	2.554	-0.072	1.311	NA
Thyridium	3.802	-0.500	1.045	NA
Naasia	4.140	1.541	1.070	NA
Kuraishia	0.224	-0.632	2.908	NA
Chthonobacter	0.278	-0.379	2.908	NA
Baekduia	3.134	-1.513	1.092	NA
Mediannikoviicoccus	0.103	0.092	2.911	NA
Isosphaera	0.656	-2.009	2.817	NA
Thermohydrogenium	1.410	-0.878	1.891	NA
Aquimarina	1.439	0.591	1.785	NA
Qaidamihabitans	5.033	0.734	1.111	NA
Tachikawaea	0.009	1.267	2.913	NA
Vagococcus	0.603	-0.277	2.388	NA

Frondihabitans	4.024	2.183	1.264	NA
Aggregicoccus	0.395	0.653	2.908	NA
Pontibacter	1.507	-1.803	1.399	NA
Psilocybe	1.438	1.496	0.847	NA
Brevibacillus	1.631	-0.097	0.933	NA
Conyzicola	1.172	-0.404	1.196	NA
Acidobacteria	2.858	0.131	0.904	NA
Bdellovibrio	0.865	0.453	1.323	NA
Miltoncostaea	4.350	0.327	1.330	NA
Eutypa	3.140	1.531	0.826	NA
Niabella	0.539	1.429	1.785	NA
Pseudogemmobacter	1.029	-2.649	2.239	NA
Truepera	3.557	-0.461	1.073	NA
Roseisolibacter	3.912	0.629	1.081	NA
Oscillochloris	0.443	0.989	2.907	NA
Diplodia	4.466	-0.398	0.826	NA
Chlorogloea	2.792	1.293	1.313	NA
Faecalibacillus	0.308	-0.051	2.907	NA
Advenella	1.895	2.022	0.937	NA
Altererythrobacter	4.874	0.499	0.920	NA
Capillimicrobium	2.546	-1.212	1.239	NA
Propionicimonas	2.047	1.186	1.047	NA
Microbacteriaceae_genus	3.298	1.303	1.166	NA
Pelagibacterium	4.466	-1.970	1.070	NA
Cadophora	1.997	1.639	1.097	NA
Ilyonectria	2.634	-0.769	1.094	NA
Actinomarinicola	1.651	1.242	1.262	NA
Escherichia_phage_500465-1_virus	0.000	0.000	0.000	NA
Sporothrix	1.005	-0.562	1.574	NA
Pyxidicoccus	1.671	0.755	1.132	NA
Euzebya	0.588	0.491	1.258	NA
Coprococcus	3.166	-0.442	1.352	NA
Salinispora	1.822	2.402	1.343	NA
Lacibacter	2.147	-1.665	2.352	NA
Emticicia	2.070	1.526	1.459	NA
Gemmiger	1.474	-0.032	1.398	NA
Coleofasciculus	1.811	0.004	1.256	NA
Mixia	2.238	-1.416	1.175	NA
Tolumonas	0.873	0.558	1.896	NA
Frankineae	1.201	-2.384	1.945	NA
Paraconexibacter	1.837	0.069	1.112	NA
Niallia	0.268	0.425	1.972	NA
Umbelopsis	0.027	1.161	2.913	NA
Minicystis	1.137	-0.273	1.766	NA
Fimbriimonas	2.579	0.402	1.145	NA
Hydrogenophilus	2.399	0.209	1.510	NA
Brenneria	3.075	-0.368	0.412	NA
Buttiauxella	1.130	1.340	0.969	NA
Exserohilum	3.437	-1.325	1.211	NA
Sanguibacter	2.066	-0.155	1.259	NA
Ferruginibacter	1.535	0.500	1.236	NA
Pengzhenrongella	0.868	1.059	1.528	NA
Enterobacteria_phage_DE3_virus	0.000	0.000	0.000	NA
Sediminibacterium	2.811	0.260	1.146	NA
Riemerella	3.291	-0.729	0.870	NA
Saccharibacteria	4.408	-1.524	0.971	NA
Fannyhessea	4.422	-1.928	1.146	NA
Angustibacter	5.041	-1.037	1.134	NA
Pedomonas	4.318	0.092	1.071	NA

Duffyella	4.145	0.071	0.742	NA
Cokeromyces	2.224	-0.270	0.486	NA
Roseobacter	0.876	-1.524	1.293	NA
Sporisorium	1.536	-0.068	1.287	NA
Chroococcidiopsis	2.687	0.374	0.889	NA
Chryseomicrobium	0.799	-1.296	1.565	NA
Clavibacter	4.588	0.812	0.911	NA
Viridilinea	0.240	0.133	2.911	NA
Terribacillus	0.063	1.544	2.913	NA
Pelorhabdus	4.813	-0.889	0.709	NA
Arthroderma	0.451	0.409	1.193	NA
Tardiphaga	3.851	0.688	0.740	NA
Pleionea	5.060	-0.541	0.758	NA
Paraferrimonas	4.903	-0.616	0.649	NA
Tenebrionibacter	0.381	0.046	0.980	NA
Anatilimnocola	0.615	-0.189	2.905	NA
Fortiea	2.157	0.404	1.307	NA
Oleiphilus	2.361	1.931	0.732	NA
Luteibacter	4.401	-0.300	0.812	NA
Zafaria	0.027	1.333	2.913	NA
Polynucleobacter	2.817	1.292	0.908	NA
Winslowiella	0.287	0.174	2.911	NA
Parerythrobacter	4.253	-2.611	1.200	NA
Microcella	4.645	0.204	0.850	NA
Myxococcales	0.658	-0.431	2.170	NA
Coprobacillus	2.469	-0.849	1.204	NA
Pseudophaeobacter	4.670	0.362	0.759	NA
Roseicella	3.466	-1.063	0.945	NA
Parageobacillus	1.709	1.047	1.399	NA
Leptomonas	2.477	-0.298	1.068	NA
Oryzihumus	0.030	1.345	2.913	NA
Cobetia	0.433	-0.948	1.360	NA
Paraprevotella	2.071	0.734	1.136	NA
Fervidibacillus	1.878	0.646	2.065	NA
Thermobacillus	0.214	-0.060	2.910	NA
Chondromyces	1.551	0.877	1.653	NA
Panacagrimonas	3.396	0.704	1.411	NA
Negativicoccus	0.811	0.553	2.907	NA
Chloroploca	0.537	2.652	2.908	NA
Chitinophagaceae_genus	0.335	0.538	2.643	NA
Actinobacteria	1.870	-0.875	1.596	NA
Yaniella	2.635	-0.307	1.267	NA
Berkiella	0.057	0.700	2.912	NA
Hephaestia	2.393	-0.201	1.203	NA
Parvimonas	4.539	0.811	0.795	NA
Rubrivirga	0.978	-0.461	2.152	NA
Zasmidium	1.710	-1.682	0.851	NA
Yoonia	0.398	2.587	2.576	NA
Nitrosospira	2.562	0.412	1.531	NA
Porphyrobacter	4.254	-0.836	0.617	NA
Fervidobacterium	0.190	1.692	2.910	NA
Hallella	4.170	-1.898	0.870	NA
Nitrotoga	4.783	0.366	0.573	NA
Paecilomyces	3.711	0.249	0.900	NA
Puccinia	4.914	-0.425	0.786	NA
Pseudorhizobium	3.953	0.200	1.093	NA
Beutenbergia	4.200	1.596	1.087	NA
Enterobacteria_phage_P7_virus	0.000	0.000	0.000	NA
Pirellula	0.574	-1.041	1.906	NA

Croceibacterium	1.140	-0.801	1.208	NA
Peptostreptococcaceae_genus	4.076	-0.544	0.877	NA
Sparassis	5.100	-0.616	0.753	NA
Halococcus	1.219	-0.501	0.883	NA
Thecamonas	1.583	-1.660	1.298	NA
Galbitalea	2.897	0.976	1.201	NA
Naumannella	5.114	0.095	0.849	NA
Vogesella	4.571	-0.733	0.914	NA
Moorella	0.340	-1.092	2.906	NA
Acidiferrimicrobium	0.742	-0.062	1.654	NA
Tepidiforma	4.308	-0.756	1.404	NA
Agreia	0.629	0.593	2.010	NA
Plantibacter	4.413	1.832	0.922	NA
Cohnella	3.253	-0.447	0.692	NA
Westerdykella	4.874	-2.098	0.893	NA
Oceanitalea	1.478	-0.361	1.538	NA
Gloeocapsa	4.503	-1.303	0.958	NA
Gulosibacter	3.895	0.525	1.084	NA
Flectobacillus	1.269	-0.757	1.291	NA
Glaciimonas	3.779	-0.063	0.987	NA
Ezakiella	1.319	-0.131	1.834	NA
Catenulispora	1.373	-2.466	2.255	NA
Peribacillus	4.798	-0.432	0.922	NA
Acidothermus	1.557	0.612	1.616	NA
Pectinatus	4.713	0.125	0.697	NA
Pannonibacter	2.009	0.793	1.350	NA
Mangrovibacillus	2.743	1.132	1.195	NA
Xanthocytophaga	2.896	0.959	1.142	NA
Xanthomonadaceae_genus	3.912	-0.206	0.868	NA
Labilibacter	4.141	-1.083	0.829	NA
Azomonas	2.713	-1.023	0.877	NA
Paludibacterium	3.956	-0.095	0.770	NA
Ancylobacter	5.043	0.415	0.938	NA
Azoarcus	4.010	-0.806	0.836	NA
Pelosinus	4.274	0.464	0.860	NA
Escherichia_phage_RCS47_virus	0.000	0.000	0.000	NA
Acetobacterium	3.427	-0.428	0.955	NA
Acetivibrio	0.767	1.935	2.085	NA
Falsirhodobacter	4.108	-0.176	1.133	NA
Thermomicrobium	3.852	0.303	0.854	NA
Neohortaea	3.383	-0.885	1.015	NA
Austwickia	2.277	-0.029	1.152	NA
Saccharibacillus	2.894	-1.077	1.308	NA
Synechococcus	0.674	-0.539	1.454	NA
Pisolithus	0.864	-0.375	0.892	NA
Archangium	2.807	0.526	0.793	NA
Sphaerotilus	3.359	-1.596	0.936	NA
Marinilactibacillus	3.912	0.615	1.067	NA
Enterobacteria_phage_phi80_virus	4.380	-0.239	0.881	NA
Firmicutes	1.459	-1.027	1.155	NA
Aliarcobacter	1.396	-0.047	2.169	NA
Cereibacter	5.029	0.216	0.784	NA
Actinobacterium	1.502	-1.980	1.536	NA
Flavimobilis	3.494	-1.579	1.055	NA
Salinicola	3.874	-0.982	0.549	NA
Azorhizobium	2.897	0.254	0.917	NA
Anaeroglobus	4.318	1.387	0.750	NA
Fusibacter	1.572	0.564	1.684	NA
Megamonas	3.586	0.122	1.011	NA

Armatimonas	0.426	0.027	2.337	NA
Huaxiibacter	3.774	0.264	0.922	NA
Ilyomonas	0.580	-1.829	2.904	NA
Cellulosimicrobium	4.141	1.691	1.241	NA
Nosocomiicoccus	1.844	-3.186	1.489	NA
Rhodocyclaceae_genus	4.038	-1.133	0.877	NA
Roseococcus	1.779	0.686	1.349	NA
Sagittula	2.597	0.160	1.212	NA
Evansella	0.719	1.525	2.215	NA
Pseudonocardiaceae_genus	1.243	-1.134	1.614	NA
Salinarimonas	4.201	-0.219	0.369	NA
Tricharina	1.265	0.180	1.198	NA
Methylovorus	2.833	0.963	0.830	NA
Yonghaparkia	1.392	0.328	1.371	NA
Geodermatophilaceae_genus	1.091	0.660	1.547	NA
Synchytrium	4.292	-2.183	1.287	NA
Thermodesulfomicrobium	0.020	1.059	2.913	NA
Eremococcus	4.175	0.427	0.870	NA
Neorhizobium	3.954	-0.461	0.971	NA
Prolinoborus	3.096	-1.348	0.811	NA
Planifilum	0.365	1.203	2.912	NA
Sutterella	3.974	0.868	0.603	NA
SsRNA_phage_SRR5466369_2_virus	0.058	1.331	2.913	NA
Sandaracinus	1.493	0.308	1.607	NA
Hyalangium	1.433	-1.828	1.979	NA
Catenibacterium	0.871	0.826	1.927	NA
Aciditerrimonas	0.899	-0.746	1.677	NA
Collibacillus	0.630	-0.279	2.591	NA
Carbonactinospora	0.476	-0.561	2.043	NA
Pseudofrankia	0.563	-1.026	2.133	NA
Extensimonas	3.609	0.797	1.008	NA
Hoyosella	1.261	-0.294	2.189	NA
Marisediminicola	0.599	1.104	2.346	NA
Ruania	2.191	-0.126	1.105	NA
Mongoliimonas	0.377	-1.267	2.906	NA
Snodgrassella	4.153	0.475	1.187	NA
Acidisphaera	2.480	-1.295	1.411	NA
Fimbriiglobus	2.293	0.124	1.247	NA
Rhizophagus	4.450	-2.274	1.003	NA
Moniliophthora	4.340	1.788	0.875	NA
Fusicatenibacter	1.772	1.359	1.362	NA
Halalkalibacterium	3.247	0.878	0.865	NA
Marinilabiliaceae_genus	2.283	0.229	0.781	NA
Halochromatium	4.224	0.684	0.638	NA
Paracaedibacter	1.572	2.275	1.556	NA
Haladaptatus	2.468	0.040	0.784	NA
Lonsdalea	0.189	0.473	0.910	NA
Protaetiibacter	2.315	1.279	1.160	NA
Cryptosporangium	3.758	-0.619	1.285	NA
Streptacidiphilus	1.421	1.044	1.478	NA
Tsuneonella	2.101	0.897	1.391	NA
Zhihengliuella	2.204	1.273	1.432	NA
Parasegetibacter	0.335	2.005	2.910	NA
Oceanicola	3.328	-0.026	1.042	NA
Cryphonectria	1.076	0.900	1.329	NA
Thermosipho	4.351	-0.012	0.422	NA
Alterileibacterium	0.250	0.727	2.910	NA
Delta	4.266	0.068	0.590	NA
Alkalihalobacillus	3.555	-0.452	0.564	NA

Parafrankia	2.734	-1.508	1.064	NA
Fonticella	0.702	-0.403	2.352	NA
Actinosynnema	3.106	-0.179	0.849	NA
Tabrizicola	3.196	-0.476	0.818	NA
Grimontella	3.770	-0.303	0.731	NA
Ehrlichia	2.744	1.262	1.077	NA
Alkaliphilus	2.459	0.543	1.497	NA
Leishmania	4.184	0.537	0.980	NA
Lagierella	0.000	0.000	0.000	NA
Ciceribacter	3.850	0.007	0.808	NA
Jannaschia	2.279	-0.191	0.406	NA
Filifactor	2.916	0.659	0.988	NA
Sorangium	0.948	0.683	2.191	NA
Arcobacter	3.620	-0.641	0.823	NA
Aureispira	3.069	0.725	0.764	NA
Gemmata	0.949	1.473	1.532	NA
Parasphingorhabdus	2.643	-1.935	1.079	NA
Caenimonas	1.434	-1.600	1.270	NA
Wolinella	0.895	0.438	1.842	NA
Herpetosiphon	1.735	-1.063	1.593	NA
Fretibacterium	3.170	0.575	1.088	NA
Flaviflexus	0.920	-1.145	1.605	NA
PreXMRV-1_provirus_complete	4.785	0.081	0.493	NA
Romboutsia	3.447	-0.252	0.741	NA
Stappia	1.931	-1.318	1.285	NA
Asinibacterium	2.381	-0.928	0.912	NA
Uruburuella	0.311	2.981	2.910	NA
Acidiluteibacter	0.402	3.087	2.910	NA
Chthoniobacter	1.949	-0.355	1.502	NA
Trichophyton	4.702	-1.603	1.342	NA
Psychrosphaera	4.364	-0.644	0.624	NA
Catonella	3.258	0.139	0.927	NA
Enterobacteria_phage_RTP_virus	2.620	-0.801	0.902	NA
Aestuariimicrobium	3.255	0.443	0.974	NA
Brasilonema	1.390	1.003	1.488	NA
Enterobacteria_phage_T4_virus	2.981	0.260	1.115	NA
Neofamilia	0.005	1.275	2.913	NA
Laetiporus	0.632	3.645	2.909	NA
Mycosynbacter	1.754	-0.386	1.267	NA
Terriglobus	1.251	-0.113	1.209	NA
Enterobacteria_phage_vB_EcoS_IME542_virus	2.043	0.821	0.992	NA
Flavipsychrobacter	0.537	1.655	2.379	NA
Aliicoccus	0.581	0.084	2.112	NA
Kockovaella	3.506	-0.482	1.017	NA
Epithele	1.370	-1.051	1.137	NA
Spleen_focus-forming_virus	3.983	0.657	0.463	NA
Cyberlindnera	2.022	0.072	1.231	NA
Pseudoglutamicibacter	1.363	0.711	1.328	NA
Herminiimonas	2.008	0.592	1.293	NA
Franconibacter	0.683	0.078	1.247	NA
Paenacidovorax	3.180	0.402	0.968	NA
Aquitalea	0.973	0.516	0.718	NA
Aliihoeflea	3.063	-1.153	1.370	NA
Xinfangfangia	1.289	-0.113	1.410	NA
Riesia	0.026	1.164	2.913	NA
Hirsutella	0.227	-0.208	2.910	NA
Magnetospirillum	1.981	-0.854	0.942	NA
Filomicrobium	2.369	0.267	1.076	NA
Escherichia_phage_phiV10_virus	2.464	0.005	0.984	NA

Companilactobacillus	0.505	0.294	2.342	NA
Lancefieldella	4.241	1.264	1.230	NA
Secondary	3.884	-0.262	0.460	NA
Pseudosporangium	0.338	2.891	2.911	NA
Cereal_yellow_dwarf_virus	0.223	0.138	2.909	NA
Tenebrionicola	0.031	1.638	2.913	NA
Anaerotruncus	0.786	1.021	0.867	NA
Acetanaerobacterium	0.863	-1.924	1.572	NA
Sphingomonadaceae_genus	2.729	-0.716	1.415	NA
Paraglaciecola	2.599	-0.154	0.645	NA
Vitreoscilla	1.803	1.667	1.449	NA
Rummeliibacillus	3.322	-0.483	0.827	NA
Lactovum	3.125	1.485	1.040	NA
Pseudomassariella	0.665	0.319	2.206	NA
Friedmanniella	3.725	-0.068	1.161	NA
Oscillibacter	1.351	-0.640	2.046	NA
Desulforhabdus	2.687	1.416	1.054	NA
Alloiococcus	1.164	1.125	1.275	NA
Minimicrobia	2.704	0.838	0.971	NA
Rhizobiaceae_genus	0.018	1.128	2.913	NA
Rhabdobacter	0.000	0.000	0.000	NA
Saccharomonospora	2.446	-0.307	0.957	NA
Kallipyga	0.000	0.000	0.000	NA
Xylella	2.546	0.080	0.607	NA
Kaistia	3.343	-0.167	0.970	NA
Phascolarctobacterium	0.627	-1.915	2.511	NA
Drepanopeziza	2.702	1.168	1.066	NA
Mobiluncus	3.137	0.725	1.034	NA
Wielerella	1.083	1.496	1.796	NA
Perlucidibaca	1.576	-0.115	0.957	NA
Anaeromyxobacter	2.178	-0.379	1.257	NA
Hoeflea	3.595	0.440	0.942	NA
Inhella	1.220	1.076	1.575	NA
Gemmatirosa	1.497	1.327	1.672	NA
Adlercreutzia	0.609	-1.407	1.827	NA
Brochothrix	2.027	-1.592	1.320	NA
Alsobacter	2.441	-1.478	1.202	NA
Cystobacter	1.340	-1.250	1.527	NA
Saccharomyces	1.469	1.885	1.556	NA
Abelson	3.843	-0.023	0.474	NA
Methylocystis	2.390	-0.413	0.865	NA
Candidatus	2.663	-0.731	1.349	NA
Abyssicoccus	5.009	0.850	1.137	NA
Nitrolancea	1.364	-3.061	1.548	NA
Tuber	2.943	-0.861	1.233	NA
Actibacterium	0.985	-1.527	1.322	NA
Propionibacteriaceae_genus	1.663	0.020	1.306	NA
Sordaria	1.077	0.667	1.189	NA
Pasteurellaceae_genus	2.823	0.210	0.906	NA
Oxalicibacterium	1.729	-3.145	1.271	NA
Aminobacter	2.429	-1.402	0.927	NA
Pajaroellobacter	0.393	0.747	2.720	NA
Flexivirga	2.311	0.092	1.101	NA
Nannochloropsis	1.721	2.016	1.244	NA
Verrucosispora	2.100	-2.105	0.909	NA
Escherichia_phage_500465-2_virus	0.000	0.000	0.000	NA
Microthrix	0.483	1.147	2.907	NA
Methylocella	3.329	0.774	1.268	NA
Oceanicella	2.913	-0.060	0.879	NA

Luteococcus	0.817	1.234	1.972	NA
Methylococcus	3.490	0.321	0.716	NA
Escherichia_virus_Lambda_2G7b	0.000	0.000	0.000	NA
Hahella	2.380	-1.044	0.731	NA
Sphingorhabdus	2.961	-1.143	1.178	NA
Demequina	2.047	0.192	1.077	NA
Type-B_symbiont_of_Plautia_stali	0.070	0.947	2.374	NA
Doolittlea	0.005	1.333	2.913	NA
Zymomonas	0.330	-0.275	2.908	NA
Photodesmus	0.007	1.485	2.913	NA
Parachlamydia	0.212	1.709	2.910	NA
Komagataeibacter	1.071	1.153	1.494	NA
Starkeya	0.752	0.732	1.222	NA
Marasmius	3.117	1.017	0.981	NA
Auritidibacter	1.274	2.828	2.728	NA
Thalassotalea	1.950	0.330	0.944	NA
Gallaecimonas	1.280	0.731	0.941	NA
Pseudoflavonifractor	1.639	1.450	0.978	NA
Dubosiella	0.165	0.412	2.910	NA
Amniculibacterium	0.863	0.755	2.885	NA
Jiangella	1.073	0.651	1.579	NA
Dactylosporangium	0.676	-0.195	2.902	NA
Dactylellina	1.676	1.295	1.378	NA
Alistipes	1.236	0.304	1.513	NA
Thermacetogenium	0.690	2.058	2.675	NA
Nitratireductor	1.522	0.970	0.963	NA
Pseudohongiella	0.062	0.510	2.912	NA
Richelia	0.517	-0.321	1.756	NA
Lacipirellula	1.839	-1.101	1.765	NA
Gallintestinimicrobium	0.837	0.259	2.604	NA
Drechmeria	0.521	0.011	1.588	NA
Goekera	0.470	0.594	2.600	NA
Agaricicola	0.028	1.426	2.913	NA
Pectobacterium_phage_CBB_virus	1.674	1.565	1.366	NA
Bacteriovorax	1.730	-0.946	1.372	NA
Phaeosphaeria	3.618	-1.981	1.463	NA
Methanothermobacter	0.156	-0.274	2.910	NA
Pedosphaera	0.186	0.156	2.910	NA
Acaricomes	2.228	-0.365	0.855	NA
Erysipelothrix	0.043	0.669	2.912	NA
Pseudorivibacter	1.087	1.065	1.402	NA
Actinophytocola	2.028	-0.155	1.497	NA
Desulfogranum	1.068	-0.017	1.474	NA
Hyaloscypha	3.026	1.702	0.842	NA
Neoarthrinium	0.839	2.938	1.371	NA
Mycetocola	0.892	0.631	1.247	NA
Metabacillus	4.195	1.116	1.057	NA
Flintibacter	0.071	0.392	2.912	NA
Anabaena	1.596	-0.162	1.201	NA
Gemmataceae_genus	1.373	-1.320	1.927	NA
Cryptomonas	1.703	0.296	1.244	NA
Hansschlegelia	1.485	1.175	1.426	NA
Peptococcus	0.489	0.472	2.905	NA
Lichenibacterium	2.044	0.612	1.401	NA
Alloalcanivorax	2.425	-0.368	0.980	NA
Zavarzinella	0.480	-0.136	2.704	NA
Saccharophagus	0.820	-2.263	1.662	NA
Pinibacter	0.256	-0.361	2.708	NA
Escherichia_phage_TL-2011b_virus	0.000	0.000	0.000	NA

Enterobacteria_phage_f1_virus	0.008	1.333	2.913	NA
Pseudobdellovibrio	0.295	0.912	2.268	NA
Sandaracinobacter	0.418	-0.181	2.908	NA
Pelagivirga	2.684	-1.127	0.614	NA
Proteus_phage_VB_PmiS-Isfahan_virus	1.743	0.138	0.531	NA
Hydrocarboniphaga	1.904	-0.964	1.169	NA
Roseitranquillus	0.614	1.002	2.111	NA
Halomicroarcula	0.764	-0.253	1.039	NA
Calidifontibacter	0.577	1.601	2.133	NA
Hassallia	0.102	1.584	2.913	NA
Siccibacter	0.129	0.166	2.302	NA
Dissoconium	0.190	0.295	2.912	NA
Quisquiliibacterium	0.886	-0.834	1.847	NA
Halobacteriovorax	1.982	0.499	0.590	NA
Pasteurella	1.152	0.018	0.576	NA
Endobacter	3.493	1.592	1.154	NA
Defluviicoccus	0.374	0.287	2.346	NA
Propionicicella	2.147	-0.774	1.250	NA
Arthromitus	0.148	2.379	2.912	NA
Melaminivora	2.129	-1.179	1.348	NA
Thermoanaerobacter	0.489	-0.341	2.906	NA
Mycoavidus	0.989	0.176	1.741	NA
Thiomonas	1.166	-0.340	1.299	NA
Hemiselmis	0.919	0.660	1.731	NA
Millisia	1.115	0.082	2.033	NA
Qingrenia	0.304	-0.056	2.907	NA
Piscicoccus	2.025	-0.242	1.477	NA
Oceanimonas	1.818	-1.275	0.642	NA
Desulfocarbo	2.982	-0.204	0.930	NA
Faecalicatena	0.820	1.282	1.499	NA
Flavobacteriaceae_genus	0.778	0.232	1.418	NA
Sphingosinithalassobacter	0.506	-0.094	1.571	NA
Tychonema	1.638	-0.394	1.131	NA
Nannocystis	1.346	-0.042	1.770	NA
Rhodovulum	0.928	0.651	1.323	NA
Escherichia_virus_Lambda_4A7	0.000	0.000	0.000	NA
Macromonas	0.175	1.140	2.910	NA
Coprinopsis	1.211	-0.760	1.518	NA
Micrococcaceae_genus	0.443	-0.188	1.833	NA
Arsenicicoccus	2.165	0.078	1.004	NA
Mameliella	2.131	0.243	1.124	NA
Wickerhamomyces	2.172	1.912	1.531	NA
Bergeriella	1.836	3.530	1.464	NA
Penaeicola	2.047	-0.469	0.893	NA
Sclerotinia	1.417	1.176	1.259	NA
Verticillium	0.616	0.418	1.621	NA
harvey_murine_sarcoma_virus	0.947	0.263	0.459	NA
Rufibacter	1.023	0.124	1.630	NA
Frateuria	2.007	0.015	0.768	NA
Halobacillus	0.907	-1.092	1.153	NA
Sneathia	0.975	-0.321	2.318	NA
Cryobacterium	1.015	1.082	1.789	NA
Sinisalibacter	0.721	-0.551	1.738	NA
Centipeda	0.032	1.529	2.913	NA
Paraflavisolibacter	0.061	1.437	2.913	NA
Gayadomonas	2.348	-0.223	0.537	NA
Sulfolobus	2.237	0.265	0.452	NA
Prauserella	0.186	0.012	2.909	NA
Siccirubricoccus	2.442	-1.657	1.349	NA

Aquabacter	1.491	0.725	1.211	NA
Verrucomicrobia	1.240	1.594	1.728	NA
Thermincola	0.633	3.147	2.249	NA
Purpureocillium	1.627	0.766	2.264	NA
Haematobacter	4.763	0.613	1.196	NA
Alkalicoccobacillus	0.836	0.110	0.756	NA
Rhodospirillaceae_genus	1.528	-0.420	1.334	NA
Paramagnetospirillum	1.385	-0.215	1.448	NA
Roseicitreum	1.865	2.378	1.176	NA
Robbsia	0.852	0.654	1.318	NA
Neurospora	0.742	0.802	2.185	NA
Arenimonas	1.644	0.187	1.335	NA
Anaerotardibacter	0.374	-0.114	2.020	NA
UNVERIFIED_CONTAM:	2.089	-0.883	1.237	NA
Pilimelia	1.515	-1.177	1.361	NA
Ustilago	1.359	0.056	1.302	NA
Schneideria	1.783	3.071	1.374	NA
Thermogemmata	0.679	0.068	2.165	NA
Caldilinea	1.616	0.991	1.735	NA
Streptoalloteichus	1.111	0.760	1.300	NA
Azovibrio	1.264	0.299	1.452	NA
Minwuia	0.928	-0.345	2.606	NA
Methylomonas	1.557	0.175	0.579	NA
Ignavibacterium	0.501	0.030	2.906	NA
Scandinavium	0.642	0.597	0.602	NA
Veillonellaceae_genus	0.639	2.692	2.295	NA
Intrasporangium	1.145	-1.805	1.702	NA
Rhodospirillum	1.216	0.795	1.343	NA
Desulfotomaculum	0.503	-0.980	2.286	NA
Marichromatium	2.010	-0.913	0.579	NA
Desertibacillus	1.178	2.169	1.518	NA
Planctomycetes	0.540	-0.036	1.889	NA
Propioniferax	0.646	0.395	2.889	NA
Meyerozyma	1.589	0.701	1.808	NA
Beijerinckia	0.400	-0.303	2.188	NA
Gamma	2.482	-1.226	1.066	NA
Myroides	1.084	1.679	1.844	NA
Mariluticola	0.153	0.786	2.911	NA
Garicola	1.949	-0.781	1.462	NA
Afifella	1.377	0.365	1.754	NA
Coniosporium	2.486	0.447	1.447	NA
Pusillibacter	0.000	0.000	0.000	NA
Simplicispira	1.925	-0.680	0.924	NA
Psychromonas	1.967	-0.446	0.649	NA
Stigmatella	0.779	-1.563	2.509	NA
Saezia	2.274	-1.852	0.824	NA
Rectinema	0.081	0.273	2.912	NA
Ewingella	0.845	-0.222	2.346	NA
Anaerobutyricum	0.119	1.135	2.911	NA
Klugiella	0.270	-0.022	2.908	NA
Arcticibacter	0.736	-1.662	1.282	NA
Propylenella	0.014	1.199	2.913	NA
Proteiniclasticum	1.099	-0.222	0.844	NA
Seohaeicola	2.294	-0.102	1.308	NA
Enterobacteria_phage_vB_EcoS_ACG-M12_virus	1.320	1.196	1.126	NA
Phototrophicus	0.608	0.152	2.176	NA
Geoalkalibacter	0.121	1.611	2.913	NA
Tomitella	1.442	1.783	1.325	NA
Pochonia	0.554	0.059	1.501	NA

Plastoroseomonas	0.699	-1.273	1.971	NA
Lawsonibacter	0.311	0.791	2.236	NA
Globicatella	1.145	2.816	1.807	NA
Plectonema	0.287	1.425	2.911	NA
Murine_osteosarcoma_virus	2.353	0.316	0.661	NA
Branchiibius	0.731	-1.186	2.296	NA
Parasutterella	0.614	0.118	1.304	NA
Plantactinospora	0.899	0.556	1.272	NA
Insolitispirillum	2.216	-0.242	1.117	NA
Truncatella	0.941	0.859	1.466	NA
Mesonia	2.526	-0.077	0.687	NA
Aaosphaeria	0.407	-0.994	2.298	NA
Bryobacter	0.568	0.530	2.900	NA
Puniceibacterium	0.603	1.256	1.674	NA
Oscillatoria	1.378	-0.730	1.144	NA
Immundisolibacter	1.554	-1.363	1.198	NA
Rhodoligotrophos	1.096	-0.343	1.626	NA
Escherichia_phage_Lambda_ev099_virus	0.000	0.000	0.000	NA
Bacteroidetes	1.362	-0.267	1.761	NA
Crenalkalicoccus	0.619	0.798	1.981	NA
Andreesenia	0.720	0.819	2.561	NA
Klebsiella_phage_4_virus	0.000	0.000	0.000	NA
Catellicoccus	0.496	1.174	2.192	NA
Microvirgula	1.559	1.550	1.663	NA
Rhodoblastus	1.061	2.686	1.498	NA
Wenxinia	0.425	0.597	1.856	NA
Planobispora	0.083	1.186	2.912	NA
Pyruvatibacter	1.367	-0.371	0.755	NA
Mycoplana	0.055	1.408	2.913	NA
Peptoclostridium	0.886	0.308	1.720	NA
Aestuariibaculum	1.315	-0.149	1.152	NA
Kordiimonas	0.368	1.682	2.323	NA
Maridesulfovibrio	0.477	2.929	2.909	NA
Neptuniibacter	0.092	0.177	2.911	NA
Stx2-converting_phage_1717_virus	0.000	0.000	0.000	NA
Planomonospora	0.421	-0.474	2.135	NA
Fischerella	0.349	-0.930	2.906	NA
Theileria	0.680	-1.358	1.110	NA
Anaeromassilibacillus	0.075	0.609	2.912	NA
Oerskovia	0.479	1.447	2.565	NA
Rudaea	1.559	0.303	0.732	NA
Phormidium	0.945	0.073	1.512	NA
Helcococcus	0.691	-1.261	2.294	NA
Glycomyces	0.654	1.677	1.959	NA
Cetobacterium	0.000	0.000	0.000	NA
Acidiphilium	0.973	1.851	1.428	NA
Steroidobacter	1.439	-2.527	1.199	NA
Hankyongella	1.396	-1.870	1.978	NA
Parachlamydiaceae_genus	0.261	2.708	2.911	NA
Actirhodobacter	0.376	0.044	2.703	NA
Methylocapsa	1.039	0.029	1.172	NA
Escherichia_phage_Cartapus_virus	0.000	0.000	0.000	NA
Rhodocista	0.302	0.231	1.675	NA
Hubei_permutotetra-like_virus	2.387	2.377	1.642	NA
Coprothermobacter	0.362	2.946	2.910	NA
Oligoflexus	0.666	-0.427	2.903	NA
Dothidotthia	2.037	-1.866	1.162	NA
Nonomuraea	1.439	-0.218	1.156	NA
Allosphingosinicella	0.640	1.316	1.592	NA

Schumannella	0.440	0.398	2.096	NA
Coriobacteriaceae_genus	0.483	1.743	2.387	NA
Nanoperiomorbus	1.710	0.924	1.345	NA
Pseudolabrys	0.793	-0.161	1.546	NA
Castellaniella	1.632	-0.800	1.429	NA
Baumannia	0.277	2.027	2.912	NA
Erythrobacteraceae_genus	1.392	0.079	1.312	NA
Phytohabitans	0.324	-0.624	2.907	NA
Parvularcula	2.074	-0.055	1.428	NA
Halophilic	1.709	-2.303	1.383	NA
Alkalibacterium	1.404	1.361	1.575	NA
Salifodinibacter	1.878	-0.863	1.627	NA
Cecembia	0.302	-0.091	2.198	NA
Catellatospora	0.854	-1.253	2.498	NA
Xylaria	0.604	-0.318	1.744	NA
Stakelama	0.615	0.034	1.575	NA
Polymorphum	0.621	-0.463	1.679	NA
Reticulibacter	0.566	-1.807	2.839	NA
Rickettsiella	1.563	-0.508	0.769	NA
Nitrosocosmicus	2.149	0.768	1.739	NA
Planctopirus	0.483	0.151	1.462	NA
Ureibacillus	0.798	1.238	1.446	NA
Fastidiosipila	0.389	2.492	2.911	NA
Carideicomes	0.567	0.319	1.159	NA
Aliidongia	0.759	1.064	1.591	NA
Caldovatus	0.480	-0.860	1.662	NA
Butyricicoccus	0.662	0.676	1.635	NA
Bacteroidales	0.732	-0.623	1.620	NA
Emergencia	2.121	-1.175	1.367	NA
Thermorudis	0.331	-0.348	2.769	NA
Rickettsia	0.876	-0.481	1.308	NA
Granulicoccus	0.329	0.957	2.658	NA
Granulicella	0.707	1.102	2.147	NA
Dinghuibacter	0.335	-0.142	2.909	NA
SsRNA_phage_SRR5466337_3_virus	0.000	0.000	0.000	NA
Gregarina	0.341	-0.214	2.755	NA
Zeimonas	0.839	-0.410	1.737	NA
Dokdonella	1.240	-2.254	1.579	NA
Kickxella	0.434	1.253	1.986	NA
Lamprobacter	0.388	-0.642	1.286	NA
Escherichia_phage_Lambda_ev207_virus	0.000	0.000	0.000	NA
Paracandidimonas	0.784	0.286	2.108	NA
Aromatoleum	0.470	-1.455	2.089	NA
Escherichia_virus_Lambda_1H12	0.000	0.000	0.000	NA
Solirhodobacter	0.058	1.944	2.912	NA
Lampropedia	1.172	-0.616	1.543	NA
Niveispirillum	2.188	0.394	1.438	NA
FBR_murine_osteosarcoma_virus	1.393	-0.004	0.615	NA
Tropicibacter	0.000	0.000	0.000	NA
Rivularia	0.796	1.199	1.852	NA
Nanosynsacchari	0.896	-0.322	1.442	NA
Nitrososphaera	1.719	1.781	2.017	NA
Actinoallomurus	1.044	1.722	1.595	NA
Proteiniphilum	0.111	1.771	2.912	NA
Ramularia	0.158	1.868	2.913	NA
Glaesserella	0.891	0.564	0.900	NA
Rehaibacterium	0.697	1.454	1.973	NA
Auraticoccus	1.408	-2.309	1.933	NA
Halteromyces	0.737	2.403	1.510	NA

Ustilaginoidea	1.348	-1.816	1.537	NA
Nocardioidaceae_genus	0.542	-0.637	2.637	NA
Cordyceps	0.869	-1.883	1.654	NA
Micavibrio	0.558	1.534	1.988	NA
Variibacter	0.081	1.547	2.912	NA
Enterobacteria_phage_T7_virus	0.002	1.344	2.913	NA
Methyloglobulus	0.550	2.373	1.455	NA
Emiliania	0.907	-1.270	1.152	NA
Oligella	1.088	0.645	2.110	NA
Dermatobacter	1.264	-1.168	1.663	NA
Pseudocnuella	0.005	1.464	2.913	NA
Lachnoclostridium	1.068	0.452	2.191	NA
Anaerobacillus	0.305	0.465	2.907	NA
Formosimonas	0.082	1.584	2.913	NA
Dongshaea	0.131	0.426	2.910	NA
Limobrevibacterium	0.486	0.710	1.742	NA
Embleya	1.575	-0.297	1.420	NA
Desulfofundulus	0.440	0.869	2.908	NA
Thermosinus	0.296	1.492	2.909	NA
Idiomarina	0.506	0.635	2.617	NA
Youxingia	0.114	0.462	2.912	NA
Desulfuromonas	1.691	1.367	1.462	NA
Stx2-converting_phage_Stx2a_WGPS2_virus	0.000	0.000	0.000	NA
Flavonifractor	0.146	1.544	2.912	NA
Paeniroseomonas	0.513	1.424	1.721	NA
Phaeobacter	1.043	-0.109	0.831	NA
Paraphaeosphaeria	0.700	-0.654	2.634	NA
Thioalkalivibrio	1.056	-0.096	1.278	NA
Subtercola	0.944	2.314	1.823	NA
Ruoffia	0.404	1.269	1.951	NA
Paraflavitalea	0.310	-0.060	2.908	NA
Crenobacter	1.097	-0.336	0.886	NA
Methylophaga	0.781	-0.311	1.192	NA
Eremomyces	0.488	-1.613	2.075	NA
Paracraurococcus	0.968	-2.536	1.535	NA
Hyphomonas	1.185	-0.348	1.672	NA
Faecalimonas	1.081	2.310	1.603	NA
Actinoalloteichus	0.689	0.051	1.327	NA
Durotheca	0.146	2.080	2.911	NA
Rhodospirillales	0.991	0.162	1.671	NA
Holdemania	0.350	-0.637	2.906	NA
Butyrivibrio	0.998	-0.843	1.650	NA
Moheibacter	0.373	0.873	2.632	NA
Sabulicella	0.024	1.582	2.913	NA
Agathobaculum	0.011	1.332	2.913	NA
Escherichia_phage_Lambda_ev243_virus	0.000	0.000	0.000	NA
Petrimonas	0.089	1.128	2.913	NA
Pseudorhodoplanes	0.962	-0.035	1.241	NA
Geomicrobium	0.127	0.103	2.911	NA
Coriobacteriales	0.250	1.603	2.910	NA
Mumia	0.814	-1.930	1.832	NA
Laspinema	0.879	2.503	1.789	NA
Natronorubrum	1.146	0.954	1.406	NA
Aeribacillus	0.369	0.662	2.911	NA
Aquimonas	0.678	-0.452	1.984	NA
Gellertiella	2.769	3.779	1.432	NA
Isoalcanivorax	1.062	0.505	1.740	NA
Neoactinobaculum	0.622	1.740	2.131	NA
Eimeria	1.027	-0.150	1.030	NA

Aquibium	0.920	0.048	1.674	NA
Dankookia	1.052	-1.385	1.354	NA
Falsochrobactrum	0.395	2.790	2.849	NA
Acidisoma	1.255	0.670	1.477	NA
Couchioplanes	0.000	0.000	0.000	NA
Yeguia	0.064	0.437	2.912	NA
Variimorphobacter	0.190	-0.219	2.909	NA
Citreicoccus	0.220	-0.626	2.909	NA
Serpentinimonas	0.193	0.041	2.910	NA
Ferribacterium	0.267	-0.829	2.908	NA
Xanthomarina	0.968	-1.423	1.485	NA
Miniimonas	0.445	-1.425	2.329	NA
Pelagerythrobacter	0.638	-1.790	2.311	NA
Allocoleopsis	0.836	-0.130	1.755	NA
Parvibaculum	0.863	2.483	1.310	NA
Microterricola	0.560	2.381	2.139	NA
Algibacillus	1.251	-1.242	0.893	NA
Simian_virus_40	1.059	0.844	0.770	NA
Anaerosphaera	0.551	0.654	2.179	NA
Saliphagus	1.228	-1.840	1.087	NA
Labedella	0.708	1.970	1.975	NA
Erysipelotrichaceae_genus	0.086	1.511	2.912	NA
Frischella	1.199	0.812	0.898	NA
Pichia	0.685	0.424	1.347	NA
Morganella	1.022	-0.037	0.556	NA
Chlamydia	0.283	1.453	2.804	NA
Aridibaculum	0.190	0.773	2.912	NA
Allostreptomyces	0.218	2.548	2.911	NA
Lentihominibacter	0.331	0.499	2.148	NA
Wigglesworthia	1.349	0.319	0.840	NA
Escherichia_virus_Lambda_2H10	0.000	0.000	0.000	NA
Hypoxylon	1.453	0.992	1.441	NA
Chiayiivirga	0.821	-1.129	2.163	NA
Vibrionimonas	0.863	0.840	1.853	NA
Krasilnikoviella	0.109	0.042	2.911	NA
Lederbergia	0.161	0.770	2.805	NA
Gulbenkiania	0.803	1.668	1.737	NA
Methanothrix	0.009	1.267	2.913	NA
Catenuloplanes	0.189	-0.462	2.909	NA
Scardovia	0.474	2.240	2.908	NA
Acidocella	0.541	0.465	1.509	NA
Chloroflexi	0.627	0.940	1.421	NA
Linderina	1.155	0.357	1.056	NA
Rouxiella	0.368	-0.565	1.999	NA
Faunimonas	0.206	1.836	2.760	NA
Zobellella	1.254	-0.560	1.614	NA
Protochlamydia	0.307	1.993	2.909	NA
Mycoplasmopsis	1.529	0.203	1.110	NA
Tuwongella	0.369	1.225	2.909	NA
Hydromonas	0.010	1.491	2.913	NA
Soleaferrea	0.148	0.631	2.910	NA
Liquorilactobacillus	0.992	0.183	1.643	NA
Thermobrachium	0.000	0.000	0.000	NA
Fuscibacter	0.854	2.068	2.074	NA
Amygdalobacter	0.251	0.628	2.909	NA
Rodentibacter	0.334	-0.463	1.810	NA
Sphingomonas-like	0.586	-0.669	1.222	NA
Intrasporangiaceae_genus	0.940	0.952	1.257	NA
Gynurincola	0.054	0.653	2.912	NA

Sandarakinorhabdus	1.376	-0.098	1.916	NA
Mycoplasma	1.308	0.428	0.808	NA
Lipomyces	0.225	0.129	2.780	NA
Mariprofundus	0.047	0.696	2.912	NA
Salicibibacter	0.350	0.569	2.907	NA
Urbifossiella	0.741	1.174	2.158	NA
Metasolibacillus	1.157	-1.169	1.239	NA
Lignipirellula	0.509	-1.650	2.560	NA
Slackia	0.879	0.033	1.782	NA
Rhodovibrio	0.716	-1.226	1.882	NA
Acidobacteriaceae_genus	0.311	-0.975	2.346	NA
Nanogingivalis	0.227	0.800	1.918	NA
Aquariibacter	0.913	-1.139	1.253	NA
Haematomicrobium	1.237	3.051	2.092	NA
Acidimicrobium	0.273	2.179	2.911	NA
Kirsten_murine_sarcoma_virus	1.366	-0.305	1.604	NA
Runella	0.324	-0.247	2.100	NA
Lindgomyces	0.470	-1.018	2.381	NA
Gallionella	0.436	0.375	0.997	NA
Fodinicola	0.558	-1.796	2.272	NA
Fontibacillus	0.151	2.366	2.911	NA
Puteibacter	1.005	0.423	1.141	NA
Mesobacillus	1.096	1.307	1.358	NA
Enterobacteria_phage_YYZ-2008_virus	0.009	1.491	2.913	NA
Vulcaniibacterium	2.155	0.100	1.585	NA
Calidifontimicrobium	0.522	0.085	2.907	NA
Elioraea	0.452	-1.427	1.918	NA
Quatrionicoccus	0.245	0.962	2.909	NA
Thiofilum	1.021	2.540	1.916	NA
Virgisporangium	0.184	-0.117	2.213	NA
Capsulimonas	0.520	-1.432	1.732	NA
Nisaea	0.072	0.722	2.912	NA
Oryzibacter	0.481	-0.551	2.172	NA
Segnochrobactrum	1.097	1.525	2.009	NA
Debaryomyces	1.396	2.979	1.687	NA
Spirilliplanes	0.536	0.009	2.910	NA
Malikia	0.578	-0.368	1.967	NA
Nigerium	0.539	2.525	2.301	NA
Pleurocapsa	1.217	-0.446	1.667	NA
Thalassolituus	1.194	-0.827	1.048	NA
Pseudovibrio	0.453	-0.058	2.100	NA
Histoplasma	0.752	0.101	1.238	NA
Deferrisoma	0.000	0.000	0.000	NA
Pelobacter	1.522	1.911	1.571	NA
Escherichia_phage_vB_EcoS_ESCO41_virus	1.030	1.282	1.384	NA
Papillibacter	0.041	1.817	2.913	NA
Acytostelium	1.048	0.817	0.857	NA
Rickettsiales	0.188	1.079	2.516	NA
Dysosmobacter	0.322	0.474	2.333	NA
Morchella	0.641	-1.029	2.376	NA
Neoroseomonas	0.161	0.228	2.910	NA
Tahibacter	0.454	0.852	1.547	NA
Propionibacterium_phage_PHL041M10_virus	0.119	0.630	2.911	NA
Rugosimonospora	0.807	-1.145	2.833	NA
Aff.	0.263	1.739	2.910	NA
Pseudaminobacter	1.415	2.064	1.493	NA
Sporomusaceae_genus	0.444	0.813	1.285	NA
Anaerostipes	0.356	-1.090	1.639	NA
Chloroflexia	0.169	2.520	2.911	NA

Mitsuokella	0.467	-0.786	2.623	NA
Propionibacterium_phage_PHL301M00_virus	0.111	1.997	2.912	NA
Pseudenterobacter	0.515	0.947	0.535	NA
Trujillella	0.289	-0.274	2.454	NA
Atlanticothrix	0.252	-0.783	2.390	NA
Hominisplanchenecus	0.033	0.816	2.913	NA
Vescimonas	0.148	-0.108	2.910	NA
Thermaurantiacus	0.224	0.987	2.910	NA
Provencibacterium	0.082	1.422	2.912	NA
Teredinibacter	0.038	0.728	2.913	NA
Pseudidiomarina	0.129	1.316	1.832	NA
Polysphondylium	0.588	3.379	2.230	NA
Rhodophyticola	0.082	0.653	2.912	NA
Cucumibacter	0.022	1.128	2.913	NA
Planomicrobium	3.609	3.550	1.388	NA
Scleromatobacter	0.808	-0.293	2.396	NA
Cucurbitaria	0.958	-1.849	1.491	NA
Falseniella	0.389	1.748	2.339	NA
Parapusillimonas	0.200	0.729	2.911	NA
Falcatimonas	0.017	1.481	2.913	NA
Lujinxingia	0.179	0.948	1.921	NA
Occultella	0.037	0.908	2.913	NA
Nitrospira	0.572	-0.027	1.933	NA
Ornithinicoccus	0.159	0.074	2.830	NA
Halovulum	0.908	1.893	1.606	NA
Microbacter	0.671	0.695	1.702	NA
Croceicoccus	0.401	-0.404	1.631	NA
Parapedobacter	0.475	0.830	1.355	NA
Acidaminococcus	0.185	0.210	2.910	NA
Frigoriflavimonas	0.201	0.797	2.275	NA
Geomonas	0.050	0.626	2.912	NA
Alkalihalophilus	0.036	1.656	2.913	NA
Hydrobacter	0.593	0.215	1.968	NA
Thermoleophilum	0.347	1.642	2.673	NA
Pyricularia	0.300	-0.483	2.360	NA
Humibacter	0.118	0.166	2.911	NA
Phytoactinopolyspora	0.463	-0.920	2.400	NA
Paenalcaligenes	0.760	1.763	0.902	NA
Neokomagataea	0.142	-0.187	2.910	NA
Drancourtella	0.234	1.636	2.911	NA
Larkinella	0.768	0.339	1.602	NA
Escherichia_phage_D6_virus	0.000	0.000	0.000	NA
Desulfoscipio	0.565	0.786	2.174	NA
Saccharimonas	0.553	0.863	1.947	NA
Neglectibacter	0.247	0.866	1.373	NA
Sinomonas	0.052	0.855	2.913	NA
Zoogloeaceae_genus	0.238	0.180	2.908	NA
Escherichia_phage_DTL_virus	0.756	-1.076	1.846	NA
Vampirovibrio	0.259	-0.806	2.908	NA
Coccidioides	0.857	-0.746	1.180	NA
Gramella	1.113	0.085	0.807	NA
Pseudodesulfovibrio	0.057	1.384	2.913	NA
Simonsiella	0.622	1.521	2.129	NA
Allorhizobium	0.157	0.465	2.910	NA
Pseudaestuariivita	0.064	1.305	2.912	NA
Macellibacteroides	0.431	1.150	2.135	NA
Caenispirillum	0.400	-1.339	2.906	NA
Jeotgalibaca	0.938	2.262	1.820	NA
Hydrotalea	0.322	0.161	2.628	NA

Desnuesiella	0.296	2.406	2.911	NA
BeAn_58058_virus	0.384	0.204	1.118	NA
Yinghuangia	0.547	-0.517	1.514	NA
Congregibacter	0.755	0.444	0.985	NA
Enterobacteria_phage_Sf6_virus	0.000	0.000	0.000	NA
Methyloradius	0.198	1.880	2.912	NA
Robiginitalea	0.271	-0.481	2.675	NA
Usitatibacter	0.008	1.501	2.913	NA
Haloechinothrix	0.147	1.631	2.737	NA
Calorimonas	0.022	0.962	2.913	NA
Aestuariivirga	0.693	1.846	2.151	NA
Tistrella	0.240	0.517	2.909	NA
Sphaerisporangium	0.405	-1.133	2.602	NA
Nannizzia	1.520	1.040	1.257	NA
Haliea	0.570	-0.276	1.354	NA
Siphonobacter	1.123	1.562	2.100	NA
Nioella	0.006	1.333	2.913	NA
Shouchella	0.581	2.178	2.684	NA
Chloroflexales	0.074	2.051	2.912	NA
Arenivirga	0.339	2.010	2.910	NA
Ktedonobacter	0.419	0.020	2.905	NA
Anaerobiospirillum	0.376	0.379	2.611	NA
Pelistega	0.345	-0.369	2.909	NA
Arboricoccus	0.363	1.194	2.359	NA
Roseibium	0.811	-1.404	1.499	NA
Propionispora	0.201	0.731	2.909	NA
Odoribacter	0.475	-1.211	2.341	NA
Miniphocaeibacter	0.034	1.530	2.913	NA
Paenarthrobacter	0.392	0.860	2.384	NA
Betaproteobacteria	0.088	1.109	2.912	NA
Betaproteobacterium_AAP65	0.934	2.706	1.031	NA
Albitalea	0.203	-0.130	2.786	NA
Trichococcus	0.181	-0.365	2.800	NA
Rhodocyclus	0.572	0.752	1.518	NA
Tetzosporium	0.333	-0.592	2.907	NA
Mesomycoplasma	0.350	0.528	2.354	NA
Elstera	0.637	-1.961	2.261	NA
Haloferax	0.284	1.041	2.711	NA
Brooklawnia	0.606	-0.557	2.133	NA
Aquirhabdus	0.124	2.019	2.912	NA
Neptunicoccus	0.118	0.797	2.911	NA
Methylocaldum	0.238	1.942	1.730	NA
Limnobaculum	0.794	1.763	1.069	NA
Viridibacillus	0.463	1.289	1.978	NA
Aggregatilinea	0.100	0.361	2.911	NA
Primorskyibacter	0.613	-0.469	0.908	NA
Fontimonas	0.265	1.236	2.787	NA
Aceticella	0.332	1.000	2.909	NA
Argonema	0.523	1.821	2.375	NA
Flagellatimonas	0.126	-0.084	2.910	NA
Weeksella	0.627	-0.529	2.908	NA
Gloeothece	0.000	0.000	0.000	NA
Camelimonas	0.584	-0.755	2.158	NA
Silanimonas	0.294	-0.308	2.910	NA
Inquilinus	0.544	-1.202	1.328	NA
Atopococcus	0.111	2.118	2.912	NA
Prosthecomicrobium	0.437	0.023	2.085	NA
Propionibacterium_phage_PHL117M01_virus	0.268	0.094	2.909	NA
Carboxylicivirga	0.110	0.270	2.912	NA

Thermopolyspora	0.034	0.975	2.913	NA
Filamentous	0.701	-0.170	1.784	NA
Rubricoccus	0.152	1.803	2.912	NA
Ferrovum	0.569	0.387	2.388	NA
Tepidanaerobacter	0.405	-1.342	2.906	NA
Sulfuriferula	0.273	2.568	2.910	NA
Idiomarinaceae_genus	0.023	1.481	2.913	NA
Chryseosolibacter	0.194	0.326	2.909	NA
Komarekiella	0.170	0.072	2.910	NA
Pelovirga	0.002	1.356	2.913	NA
Pirellulimonas	0.144	2.141	2.911	NA
Propionibacterium_phage_PHL116M00_virus	0.473	3.374	2.484	NA
Paenisporosarcina	0.629	1.101	2.911	NA
Salegentibacter	0.151	0.706	2.910	NA
Muribaculaceae_genus	0.814	1.490	1.314	NA
Arachidicoccus	0.202	1.314	2.911	NA
Sulfuricystis	0.442	-1.260	2.585	NA
Luteipulveratus	0.361	0.579	2.611	NA
Chryseolinea	0.170	1.194	2.912	NA
Pontibrevibacter	0.084	0.323	2.912	NA
Hanamia	0.253	2.802	2.911	NA
Penicilliopsis	0.668	0.473	2.312	NA
Podospora	0.781	1.677	0.751	NA
Grimontia	0.335	0.894	0.758	NA
Thioclava	0.294	0.222	2.319	NA
Propionibacterium_phage_SKKY_virus	0.362	1.689	2.909	NA
Citreimonas	0.088	0.216	2.911	NA
Propionibacterium_phage_PAD20_virus	0.255	0.796	2.910	NA
Planococcaceae_genus	0.288	1.293	2.909	NA
Caldicellulosiruptor	0.237	0.473	2.908	NA
Hartmannibacter	0.288	0.221	2.907	NA
Helicobacter	0.471	-0.923	2.189	NA
Verticiella	0.592	1.629	2.364	NA
Pseudescherichia	0.062	1.532	2.912	NA
Akanthomyces	0.281	-0.001	2.907	NA
Plasticicumulans	0.111	2.172	2.912	NA
Dongia	0.909	-0.676	1.656	NA
Escherichia_phage_520873_virus	0.000	0.000	0.000	NA
Bergeyella	0.183	0.495	2.865	NA
Tersicoccus	1.189	-2.231	2.151	NA
Stenoxybacter	0.289	2.480	2.911	NA
Cronobacter_phage_vB_CsaM_GAP32_virus	0.325	1.400	2.686	NA
Plesiocystis	0.041	1.053	2.913	NA
Holdemanella	0.771	-0.455	1.823	NA
Torulaspora	0.428	-0.041	2.905	NA
Sulfuritalea	0.033	1.128	2.913	NA
Streptobacillus	0.298	-0.969	2.907	NA
Viadribacter	0.000	0.000	0.000	NA
Betaproteobacterium_AAP121	0.906	2.550	1.004	NA
Jaminaea	0.197	-0.246	2.910	NA
Propionibacterium_phage_P100D_virus	0.085	0.764	2.912	NA
Asanoa	0.412	0.747	2.907	NA
Enterocloster	0.679	1.073	1.796	NA
Petrotoga	0.426	1.209	2.144	NA
Wenjunlia	0.648	-0.875	2.090	NA
Salinisphaera	0.252	0.317	0.902	NA
Lewinella	0.693	0.800	0.582	NA
Chloroflexus	0.443	0.729	2.435	NA
Buchananella	0.056	1.451	2.913	NA

Rhodocyclales	0.737	2.362	2.276	NA
Frisingicoccus	0.052	0.585	2.912	NA
Pseudanabaena	0.202	-0.528	2.909	NA
Effusibacillus	0.123	0.134	2.911	NA
Blattabacterium	0.399	0.279	2.140	NA
Corticibacterium	0.584	2.378	1.975	NA
Paramesorhizobium	0.152	1.232	2.912	NA
Betaproteobacterium_AAP99	0.003	1.333	2.913	NA
Oceanotoga	0.000	0.000	0.000	NA
Salmonella_phage_SJ46_virus	0.000	0.000	0.000	NA
Roseibaca	0.208	1.760	2.515	NA
Allofustis	0.129	0.728	2.911	NA
Phialemonium	0.746	1.442	1.994	NA
Thermaerobacter	0.441	0.991	2.394	NA
Phaseolus_vulgaris_endornavirus	0.000	0.000	0.000	NA
Defluviimonas	0.700	-1.874	1.449	NA
Syntrophomonas	0.225	1.160	2.909	NA
Paludicola	0.000	0.000	0.000	NA
Kaustia	0.000	0.000	0.000	NA
Pararobbsia	0.017	1.498	2.913	NA
Melittangium	0.138	-0.144	2.910	NA
Robinsoniella	0.031	0.984	2.913	NA
