# Supplementary material for: Shotgun Metagenome Analysis of Two Schizaphis graminum Biotypes over Time With and Without Carried Cereal Yellow Dwarf Virus
Source: Insects. 2025 May 23;16(6):554. doi: 10.3390/insects16060554 (PMC12193481; doi:10.3390/insects16060554)
Supplement: Supplementary file 1 [file insects-16-00554-s001.zip › Table S5.pdf]

Table S5. DESeq2 results for comparison by biotype, arranged by log_2 fold change.

Genus	BaseMean	Log2FC	LFCSE	Padj
Batrachochytrium	112.118	-28.293	1.368	8.685e-05
Melampsora	13.623	-7.249	1.176	1.076e-03
Fimicolochytrium	88.813	-6.632	1.020	6.071e-05
Aequitasia	109.785	-6.263	0.905	1.182e-05
Mycotypha	36.618	-5.142	1.091	1.904e-03
Meira	91.815	-4.482	0.990	2.929e-03
Leyella	5.334	-4.171	1.105	8.117e-03
Guillardia	48.159	-3.820	0.980	1.199e-02
Mitosporidium	133.751	-3.722	1.268	9.076e-02
Letharia	146.679	-3.690	0.527	1.728e-07
Type-D_symbiont_of_Plautia_stali	2.107	-3.637	2.900	NA
Spizellomyces	92.702	-3.477	1.072	4.529e-02
Nosocomiicoccus	1.844	-3.186	1.489	NA
Macroventuria	247.054	-3.156	0.692	1.429e-03
Oxalicibacterium	1.729	-3.145	1.271	NA
Proteobacteria	45.108	-3.137	0.666	8.664e-04
Loigolactobacillus	25.133	-3.116	0.941	3.332e-02
Saitoella	68.439	-3.084	1.433	2.775e-01
Brettanomyces	3.564	-3.069	1.398	NA
Nitrolancea	1.364	-3.061	1.548	NA
Lobosporangium	154.598	-3.056	1.161	1.295e-01
Wallemia	78.999	-3.044	0.992	5.744e-02
Polychytrium	132.053	-3.028	0.903	3.014e-02
Ascochyta	1160.970	-2.988	0.710	3.059e-03
Didymella	21.350	-2.978	0.967	4.913e-02
Wickerhamiella	12.358	-2.927	0.688	2.391e-03
Pontibacillus	12.762	-2.875	1.243	2.512e-01
Sphaerobacter	12.579	-2.814	1.164	1.772e-01
Mucor	15.110	-2.761	0.805	2.024e-02
Parastagonospora	616.798	-2.742	0.620	1.614e-03
Salipaludibacillus	74.463	-2.707	0.477	1.859e-05
Leptosphaeria	79.063	-2.699	0.768	1.954e-02
Protomyces	14.504	-2.668	0.946	1.573e-01
Thiolapillus	20.111	-2.661	0.719	1.176e-02
Niastella	47.268	-2.653	1.185	2.341e-01
Pseudogemmobacter	1.029	-2.649	2.239	NA
Lentilactobacillus	33.253	-2.626	1.097	1.842e-01
Parerythrobacter	4.253	-2.611	1.200	NA
Nodosilinea	25.845	-2.605	0.738	1.654e-02
Agilicoccus	113.618	-2.542	0.521	3.349e-04
Paracraurococcus	0.968	-2.536	1.535	NA
Steroidobacter	1.439	-2.527	1.199	NA
Sugiyamaella	3.235	-2.523	1.171	NA
Suillus	38.317	-2.515	0.948	1.097e-01
Rhizopus	1.598	-2.494	1.482	NA
Catenulispora	1.373	-2.466	2.255	NA
Polaribacter	19.839	-2.434	0.477	1.463e-04
Sporosarcina	5.918	-2.407	0.745	2.468e-02
Frankineae	1.201	-2.384	1.945	NA
Acaromyces	26.363	-2.366	0.867	9.576e-02
Zychaea	9.011	-2.348	1.281	3.471e-01
Boeremia	20.842	-2.314	0.804	6.810e-02
Auraticoccus	1.408	-2.309	1.933	NA
Halophilic	1.709	-2.303	1.383	NA
Rhizophagus	4.450	-2.274	1.003	NA

Radiomyces	1.508	-2.269	1.183	NA
Saccharophagus	0.820	-2.263	1.662	NA
Dokdonella	1.240	-2.254	1.579	NA
Propioniciclava	35.109	-2.243	0.566	4.092e-03
Algiphilus	9.411	-2.242	0.882	1.068e-01
Tersicoccus	1.189	-2.231	2.151	NA
Humisphaera	4.666	-2.195	1.303	NA
Synchytrium	4.292	-2.183	1.287	NA
Pluralibacter	1.241	-2.166	1.300	NA
Trematosphaeria	6.493	-2.157	0.982	1.952e-01
Mycena	39.124	-2.131	0.797	1.035e-01
Verrucosispora	2.100	-2.105	0.909	NA
Westerdykella	4.874	-2.098	0.893	NA
Barrientosiimonas	8.157	-2.051	1.005	3.507e-01
Pneumocystis	8.386	-2.046	1.198	4.096e-01
Microsporum	6.726	-2.034	1.033	2.798e-01
Isosphaera	0.656	-2.009	2.817	NA
Phaeosphaeria	3.618	-1.981	1.463	NA
Actinobacterium	1.502	-1.980	1.536	NA
Pelagibacterium	4.466	-1.970	1.070	NA
Turicibacter	4.462	-1.962	1.133	NA
Elstera	0.637	-1.961	2.261	NA
Trichosporon	145.713	-1.948	0.721	9.555e-02
Okeania	8.897	-1.939	0.754	1.068e-01
Parasphingorhabdus	2.643	-1.935	1.079	NA
Mumia	0.814	-1.930	1.832	NA
Domibacillus	6.862	-1.928	1.169	4.192e-01
Fannyhessea	4.422	-1.928	1.146	NA
Acetanaerobacterium	0.863	-1.924	1.572	NA
Phascolarctobacterium	0.627	-1.915	2.511	NA
Acidithiobacillus	129.947	-1.908	0.445	1.614e-03
Azonexus	5.356	-1.898	1.067	4.471e-01
Hallella	4.170	-1.898	0.870	NA
Cordyceps	0.869	-1.883	1.654	NA
Pseudomicrostroma	29.079	-1.879	0.830	2.079e-01
Defluviimonas	0.700	-1.874	1.449	NA
Hankyongella	1.396	-1.870	1.978	NA
Dothidotthia	2.037	-1.866	1.162	NA
Pleomorphomonas	4.145	-1.856	1.137	NA
Saezia	2.274	-1.852	0.824	NA
Cucurbitaria	0.958	-1.849	1.491	NA
Terrihabitans	1.239	-1.844	2.024	NA
Saliphagus	1.228	-1.840	1.087	NA
Ilyomonas	0.580	-1.829	2.904	NA
Hyalangium	1.433	-1.828	1.979	NA
Saccharothrix	13.589	-1.821	0.527	1.551e-02
Ustilaginoidea	1.348	-1.816	1.537	NA
Reticulibacter	0.566	-1.807	2.839	NA
Intrasporangium	1.145	-1.805	1.702	NA
Pontibacter	1.507	-1.803	1.399	NA
Fodinicola	0.558	-1.796	2.272	NA
Pelagerythrobacter	0.638	-1.790	2.311	NA
Iamia	6.518	-1.770	1.081	4.561e-01
Terrimonas	47.737	-1.756	0.490	1.199e-02
Pseudooceanicola	54.236	-1.751	0.400	1.157e-03
Chrysosporum	14.164	-1.746	0.707	1.314e-01
Rhabdothermincola	6.601	-1.721	0.826	2.252e-01
Phyllobacterium	39.527	-1.694	0.511	2.468e-02
Jatrophihabitans	6.706	-1.691	1.007	4.192e-01

Tautonia	3.902	-1.686	1.544	NA
Zasmidium	1.710	-1.682	0.851	NA
Schizophyllum	63.317	-1.665	0.867	3.275e-01
Lacibacter	2.147	-1.665	2.352	NA
Arcticibacter	0.736	-1.662	1.282	NA
Thecamonas	1.583	-1.660	1.298	NA
Cutaneotrichosporon	11.862	-1.660	0.870	3.166e-01
Dorea	18.063	-1.659	0.575	5.744e-02
Siccirubricoccus	2.442	-1.657	1.349	NA
Kineosporia	6.745	-1.650	0.995	4.876e-01
Lignipirellula	0.509	-1.650	2.560	NA
Eremomyces	0.488	-1.613	2.075	NA
Trichophyton	4.702	-1.603	1.342	NA
Mixta	10.290	-1.601	0.569	7.569e-02
Caenimonas	1.434	-1.600	1.270	NA
Sphaerotilus	3.359	-1.596	0.936	NA
Brochothrix	2.027	-1.592	1.320	NA
Planctomonas	7.195	-1.581	0.920	5.084e-01
Gamsiella	5.792	-1.581	1.087	4.876e-01
Flavimobilis	3.494	-1.579	1.055	NA
Colwellia	7.776	-1.565	0.497	3.185e-02
Stigmatella	0.779	-1.563	2.509	NA
Entotheonella	5.670	-1.560	0.721	2.102e-01
Azotobacter	95.528	-1.536	0.422	1.037e-02
Glutamicibacter	58.869	-1.529	0.469	2.468e-02
Actibacterium	0.985	-1.527	1.322	NA
Roseobacter	0.876	-1.524	1.293	NA
Saccharibacteria	4.408	-1.524	0.971	NA
Baekduia	3.134	-1.513	1.092	NA
Parafrankia	2.734	-1.508	1.064	NA
Nitriliruptoraceae_genus	156.786	-1.506	0.900	4.398e-01
Emericellopsis	3.788	-1.491	1.494	NA
Alsobacter	2.441	-1.478	1.202	NA
Rhodobacteraceae_genus	44.452	-1.471	0.458	2.857e-02
Blautia	17.234	-1.467	0.530	7.569e-02
Aromatoleum	0.470	-1.455	2.089	NA
Neofusicoccum	52.004	-1.451	0.467	3.490e-02
Geobacillus	32.342	-1.444	0.618	1.781e-01
Dichomitus	29.347	-1.440	0.673	2.409e-01
Solirubrobacter	30.724	-1.434	0.602	1.617e-01
Capsulimonas	0.520	-1.432	1.732	NA
Dechloromonas	8.894	-1.432	0.718	2.702e-01
Hydrogenophaga	66.646	-1.429	0.360	3.207e-03
Elioraea	0.452	-1.427	1.918	NA
Agarivorans	92.107	-1.426	0.574	1.295e-01
Miniimonas	0.445	-1.425	2.329	NA
Xanthomarina	0.968	-1.423	1.485	NA
Acanthamoeba	49.409	-1.420	0.667	2.453e-01
Mixia	2.238	-1.416	1.175	NA
Adlercreutzia	0.609	-1.407	1.827	NA
Roseibium	0.811	-1.404	1.499	NA
Aminobacter	2.429	-1.402	0.927	NA
Salipiger	55.657	-1.402	0.266	3.845e-05
Endosymbiont	5.833	-1.397	0.566	1.104e-01
Dankookia	1.052	-1.385	1.354	NA
Immundisolibacter	1.554	-1.363	1.198	NA
Theileria	0.680	-1.358	1.110	NA
Qipengyuania	57.461	-1.356	0.380	1.176e-02
Prolinoborus	3.096	-1.348	0.811	NA

Tepidanaerobacter	0.405	-1.342	2.906	NA
Paracoccidioides	3.834	-1.339	0.830	NA
Caenispirillum	0.400	-1.339	2.906	NA
Fenollaria	1.816	-1.331	1.548	NA
Exserohilum	3.437	-1.325	1.211	NA
Robertmurraya	10.088	-1.323	0.616	2.244e-01
Fusarium	142.957	-1.322	0.563	1.673e-01
Gemmataceae_genus	1.373	-1.320	1.927	NA
Stappia	1.931	-1.318	1.285	NA
Rhizorhapis	25.863	-1.317	0.478	7.569e-02
Clavispora	11.624	-1.314	1.465	7.674e-01
Gloeocapsa	4.503	-1.303	0.958	NA
Glycocaulis	61.217	-1.302	1.088	6.438e-01
Chryseomicrobium	0.799	-1.296	1.565	NA
Acidisphaera	2.480	-1.295	1.411	NA
Tolypothrix	8.797	-1.287	0.846	4.829e-01
Actinobaculum	7.638	-1.286	0.828	4.856e-01
Oceanimonas	1.818	-1.275	0.642	NA
Blastomyces	2.516	-1.274	0.903	NA
Plastoroseomonas	0.699	-1.273	1.971	NA
Emiliania	0.907	-1.270	1.152	NA
Mongoliimonas	0.377	-1.267	2.906	NA
Helcococcus	0.691	-1.261	2.294	NA
Sulfuricystis	0.442	-1.260	2.585	NA
Catellatospora	0.854	-1.253	2.498	NA
Cystobacter	1.340	-1.250	1.527	NA
Algibacillus	1.251	-1.242	0.893	NA
Trametes	13.922	-1.240	0.764	4.490e-01
Gamma	2.482	-1.226	1.066	NA
Rhodovibrio	0.716	-1.226	1.882	NA
Protofrankia	75.565	-1.221	0.385	2.904e-02
Capillimicrobium	2.546	-1.212	1.239	NA
Odoribacter	0.475	-1.211	2.341	NA
Clostridiales	35.068	-1.211	0.371	2.496e-02
Inquilinus	0.544	-1.202	1.328	NA
Paeniglutamicibacter	22.306	-1.192	0.408	5.744e-02
Ilumatobacter	9.966	-1.190	1.092	6.852e-01
Branchiibius	0.731	-1.186	2.296	NA
Melaminivora	2.129	-1.179	1.348	NA
Pilimelia	1.515	-1.177	1.361	NA
Emergencia	2.121	-1.175	1.367	NA
Metasolibacillus	1.157	-1.169	1.239	NA
Dermatobacter	1.264	-1.168	1.663	NA
Conexibacter	13.631	-1.166	0.715	4.490e-01
Aliihoeflea	3.063	-1.153	1.370	NA
Rugosimonospora	0.807	-1.145	2.833	NA
Flaviflexus	0.920	-1.145	1.605	NA
Sphingorhabdus	2.961	-1.143	1.178	NA
Aquariibacter	0.913	-1.139	1.253	NA
Pseudonocardiaceae_genus	1.243	-1.134	1.614	NA
Rhodocyclaceae_genus	4.038	-1.133	0.877	NA
Sphaerisporangium	0.405	-1.133	2.602	NA
Chiayiivirga	0.821	-1.129	2.163	NA
Pelagivirga	2.684	-1.127	0.614	NA
Weissella	5.780	-1.122	0.782	4.920e-01
Talaromyces	8.852	-1.108	0.673	4.209e-01
Halopseudomonas	3.486	-1.101	0.873	NA
Lacipirellula	1.839	-1.101	1.765	NA
Apiotrichum	11.988	-1.094	0.770	5.388e-01

Moorella	0.340	-1.092	2.906	NA
Halobacillus	0.907	-1.092	1.153	NA
Anaerostipes	0.356	-1.090	1.639	NA
Labilibacter	4.141	-1.083	0.829	NA
Saccharibacillus	2.894	-1.077	1.308	NA
Faecalibacterium	9.066	-1.076	0.804	5.623e-01
Escherichia_phage_DTL_virus	0.756	-1.076	1.846	NA
Myceligenerans	6.325	-1.072	0.572	3.035e-01
Roseicella	3.466	-1.063	0.945	NA
Herpetosiphon	1.735	-1.063	1.593	NA
Saprolegnia	7.421	-1.061	0.567	3.214e-01
Epithele	1.370	-1.051	1.137	NA
Hahella	2.380	-1.044	0.731	NA
Pirellula	0.574	-1.041	1.906	NA
Angustibacter	5.041	-1.037	1.134	NA
Methylosinus	0.957	-1.034	1.065	NA
Morchella	0.641	-1.029	2.376	NA
Firmicutes	1.459	-1.027	1.155	NA
Pseudofrankia	0.563	-1.026	2.133	NA
Azomonas	2.713	-1.023	0.877	NA
Aliterella	5.985	-1.022	1.020	6.899e-01
Lindgomyces	0.470	-1.018	2.381	NA
Xylanimonas	3.351	-1.018	0.961	NA
Chelativorans	10.320	-1.018	0.801	6.358e-01
Kalmanozyma	4.191	-1.000	0.795	NA
Aaosphaeria	0.407	-0.994	2.298	NA
Collinsella	12.119	-0.986	0.826	6.473e-01
Diolcogaster_facetosa_bracovirus	12.306	-0.986	0.563	4.070e-01
Salinicola	3.874	-0.982	0.549	NA
Desulfotomaculum	0.503	-0.980	2.286	NA
Acidobacteriaceae_genus	0.311	-0.975	2.346	NA
Mitsuaria	151.580	-0.974	0.305	2.809e-02
Novosphingobium	521.937	-0.970	0.279	1.345e-02
Streptobacillus	0.298	-0.969	2.907	NA
Pseudomonas	23142.274	-0.967	0.216	7.413e-04
Hydrocarboniphaga	1.904	-0.964	1.169	NA
Psychrobacter	113.554	-0.963	0.280	1.551e-02
Moesziomyces	19.781	-0.959	0.900	6.852e-01
Cobetia	0.433	-0.948	1.360	NA
Carnobacterium	19.889	-0.947	0.477	2.790e-01
Sphaerulina	8.660	-0.947	0.649	5.199e-01
Bacteriovorax	1.730	-0.946	1.372	NA
Gemmobacter	16.490	-0.942	0.660	5.388e-01
Taibaiella	11.521	-0.937	0.779	6.438e-01
Phanerochaete	11.292	-0.935	0.813	6.515e-01
Quadrisphaera	13.743	-0.934	0.865	6.852e-01
Fischerella	0.349	-0.930	2.906	NA
Polymorphobacter	8.366	-0.930	0.838	8.218e-01
Vallicoccus	2.925	-0.930	1.250	NA
Asinibacterium	2.381	-0.928	0.912	NA
Georgenia	10.823	-0.927	0.634	5.588e-01
Helicobacter	0.471	-0.923	2.189	NA
Uncultured	130.743	-0.921	0.349	9.576e-02
Kocuria	483.512	-0.920	0.407	1.894e-01
Phytoactinopolyspora	0.463	-0.920	2.400	NA
Aliiruegeria	16.063	-0.919	0.570	4.490e-01
Epilithonimonas	41.936	-0.916	0.353	1.012e-01
Diaporthe	5.499	-0.916	1.081	8.180e-01
Marichromatium	2.010	-0.913	0.579	NA

Rhabdonatronobacter	170.476	-0.912	0.227	2.419e-03
Flavisolibacter	1.622	-0.907	1.236	NA
Devosia	74.074	-0.904	0.335	8.422e-02
Ornithinimicrobium	139.539	-0.903	0.448	2.775e-01
Nakamurella	30.160	-0.897	0.520	4.110e-01
Rhizoctonia	10.173	-0.892	0.827	6.765e-01
Pelorhabdus	4.813	-0.889	0.709	NA
Kitasatospora	20.269	-0.888	0.554	4.490e-01
Citromicrobium	6.385	-0.888	0.819	6.715e-01
Neohortaea	3.383	-0.885	1.015	NA
Plautia	0.323	-0.884	1.579	NA
UNVERIFIED_CONTAM:	2.089	-0.883	1.237	NA
Alteromonas	44.223	-0.882	0.485	3.507e-01
Thermohydrogenium	1.410	-0.878	1.891	NA
Chitinimonas	12.648	-0.876	0.678	6.057e-01
Actinobacteria	1.870	-0.875	1.596	NA
Wenjunlia	0.648	-0.875	2.090	NA
Lutimaribacter	14.713	-0.870	0.549	4.561e-01
Salifodinibacter	1.878	-0.863	1.627	NA
Tuber	2.943	-0.861	1.233	NA
Caldovatus	0.480	-0.860	1.662	NA
Pseudoduganella	4.710	-0.855	0.686	NA
Magnetospirillum	1.981	-0.854	0.942	NA
Coprobacillus	2.469	-0.849	1.204	NA
Prosthecobacter	3.876	-0.848	0.998	NA
Butyrivibrio	0.998	-0.843	1.650	NA
Didymosphaeria	32.344	-0.838	0.601	5.474e-01
Porphyrobacter	4.254	-0.836	0.617	NA
Quisquiliibacterium	0.886	-0.834	1.847	NA
Ferribacterium	0.267	-0.829	2.908	NA
Acidaminobacter	7.508	-0.828	0.660	6.171e-01
Thalassolituus	1.194	-0.827	1.048	NA
Putridiphycobacter	3.881	-0.826	0.793	NA
Pleomorpha	4.324	-0.823	0.905	NA
Eleftheria	9.525	-0.819	0.622	5.814e-01
Sinorhizobium	5.189	-0.819	0.800	6.765e-01
Vampirovibrio	0.259	-0.806	2.908	NA
Azoarcus	4.010	-0.806	0.836	NA
Enterobacteria_phage_RTP_virus	2.620	-0.801	0.902	NA
Croceibacterium	1.140	-0.801	1.208	NA
Castellaniella	1.632	-0.800	1.429	NA
Acidihalobacter	28.945	-0.794	0.517	4.856e-01
Aeromicrobium	36.608	-0.794	0.339	1.661e-01
Phenylobacterium	73.211	-0.789	0.382	2.556e-01
Hymenobacter	123.295	-0.787	0.322	1.314e-01
Mitsuokella	0.467	-0.786	2.623	NA
Citricoccus	29.626	-0.785	0.497	4.610e-01
Atlanticothrix	0.252	-0.783	2.390	NA
Garicola	1.949	-0.781	1.462	NA
Propionicicella	2.147	-0.774	1.250	NA
Methylomicrobium	16.552	-0.772	0.561	5.623e-01
Ilyonectria	2.634	-0.769	1.094	NA
Corallococcus	123.324	-0.762	0.257	4.537e-02
Facklamia	31.482	-0.762	0.397	3.053e-01
Coprinopsis	1.211	-0.760	1.518	NA
Flectobacillus	1.269	-0.757	1.291	NA
Tepidiforma	4.308	-0.756	1.404	NA
Ideonella	12.997	-0.755	0.557	5.623e-01
Camelimonas	0.584	-0.755	2.158	NA

Ectobacillus	119.344	-0.752	0.349	2.252e-01
Aciditerrimonas	0.899	-0.746	1.677	NA
Coccidioides	0.857	-0.746	1.180	NA
Vogesella	4.571	-0.733	0.914	NA
Candidatus	2.663	-0.731	1.349	NA
Oscillatoria	1.378	-0.730	1.144	NA
Riemerella	3.291	-0.729	0.870	NA
Fulvia	3.461	-0.726	0.670	NA
Neomicrococcus	5.484	-0.725	0.687	6.715e-01
Sphingomonadaceae_genus	2.729	-0.716	1.415	NA
Penicillium	43.107	-0.710	0.356	2.790e-01
Alloscardovia	83.257	-0.703	0.443	4.585e-01
Rhodopirellula	17.595	-0.703	0.395	3.713e-01
Veillonella	194.159	-0.696	0.328	2.341e-01
Luteolibacter	24.040	-0.694	0.395	4.023e-01
Salinicoccus	15.586	-0.688	0.636	6.852e-01
Pleurotus	2.254	-0.687	1.093	NA
Reyranella	10.238	-0.686	0.568	6.326e-01
Geodermatophilus	37.591	-0.685	0.551	6.229e-01
Ramlibacter	69.721	-0.681	0.372	3.471e-01
Simplicispira	1.925	-0.680	0.924	NA
Pseudoroseomonas	11.878	-0.679	0.590	6.715e-01
Dongia	0.909	-0.676	1.656	NA
Aurantiacibacter	6.751	-0.674	0.793	7.674e-01
Segatella	9.943	-0.670	0.576	6.438e-01
Sphingomonas-like	0.586	-0.669	1.222	NA
Kribbella	6.342	-0.665	0.562	6.430e-01
Oxalobacteraceae_genus	2.465	-0.657	0.872	NA
Paraphaeosphaeria	0.700	-0.654	2.634	NA
Aeromonas	84.822	-0.651	0.219	4.397e-02
Roseomonas	144.562	-0.648	0.383	4.192e-01
Roseibacterium	113.257	-0.644	0.456	5.388e-01
Psychrosphaera	4.364	-0.644	0.624	NA
Lamprobacter	0.388	-0.642	1.286	NA
Arcobacter	3.620	-0.641	0.823	NA
Oscillibacter	1.351	-0.640	2.046	NA
Pediococcus	1.064	-0.638	1.088	NA
Sphingosinicella	7.978	-0.637	0.777	7.905e-01
Nocardioidaceae_genus	0.542	-0.637	2.637	NA
Holdemania	0.350	-0.637	2.906	NA
Aureimonas	24.555	-0.633	0.508	6.215e-01
Weizmannia	1.405	-0.632	2.213	NA
Kuraishia	0.224	-0.632	2.908	NA
Allomuricauda	54.976	-0.632	0.338	3.285e-01
Lachnospira	14.399	-0.630	0.434	5.378e-01
Methylovulum	39.553	-0.629	0.471	5.738e-01
Type-F_symbiont_of_Plautia_stali	0.400	-0.627	2.906	NA
Citreicoccus	0.220	-0.626	2.909	NA
Phytohabitans	0.324	-0.624	2.907	NA
Bacteroidales	0.732	-0.623	1.620	NA
Cryptosporangium	3.758	-0.619	1.285	NA
Lampropedia	1.172	-0.616	1.543	NA
Sparassis	5.100	-0.616	0.753	NA
Paraferrimonas	4.903	-0.616	0.649	NA
Phycomyces	1.988	-0.616	1.142	NA
Agathobacter	7.239	-0.611	1.010	8.558e-01
Zymoseptoria	38.563	-0.609	0.381	4.561e-01
Methylosarcina	17.576	-0.607	0.571	6.864e-01
Peredibacter	28.482	-0.605	0.571	6.852e-01

Moorena	7.922	-0.602	0.836	8.218e-01
Cryptococcus	8.210	-0.600	0.814	8.242e-01
Phialophora	4.587	-0.599	0.944	NA
Solemya	6.351	-0.598	0.670	7.449e-01
Tessaracoccus	17.444	-0.594	0.640	7.460e-01
Tetzosporium	0.333	-0.592	2.907	NA
Alloprevotella	42.819	-0.590	0.474	6.215e-01
Gullanella	0.959	-0.583	1.008	NA
Scedosporium	0.752	-0.570	1.715	NA
Babesia	6.351	-0.567	0.647	7.460e-01
Streptomyces	1952.590	-0.566	0.137	1.771e-03
Rouxiella	0.368	-0.565	1.999	NA
Sporothrix	1.005	-0.562	1.574	NA
Dyadobacter	16.940	-0.562	0.465	6.358e-01
Aspergillus	116.582	-0.562	0.337	4.293e-01
Wenzhouxiangella	3.178	-0.561	0.810	NA
Carbonactinospora	0.476	-0.561	2.043	NA
Zobellella	1.254	-0.560	1.614	NA
Brooklawnia	0.606	-0.557	2.133	NA
Labilithrix	4.216	-0.555	1.034	NA
Oryzibacter	0.481	-0.551	2.172	NA
Sinisalibacter	0.721	-0.551	1.738	NA
Synechocystis	16.400	-0.547	0.661	7.928e-01
Peptostreptococcaceae_genus	4.076	-0.544	0.877	NA
Endocarpon	5.929	-0.544	0.656	8.144e-01
Thermococcus	14.111	-0.543	0.406	5.697e-01
Pleionea	5.060	-0.541	0.758	NA
Synechococcus	0.674	-0.539	1.454	NA
Dioszegia	17.319	-0.538	0.938	8.558e-01
Scytonema	22.749	-0.534	0.481	6.715e-01
Weeksella	0.627	-0.529	2.908	NA
Pseudanabaena	0.202	-0.528	2.909	NA
Blastococcus	188.669	-0.528	0.411	6.026e-01
Alkanindiges	5.454	-0.528	0.703	8.378e-01
Yersinia	118.324	-0.527	0.383	5.592e-01
Phaeodactylum	12.627	-0.524	0.525	7.151e-01
Ectothiorhodospira	16.455	-0.519	0.358	5.327e-01
Coniophora	9.959	-0.517	1.022	8.686e-01
Yinghuangia	0.547	-0.517	1.514	NA
Tissierellia	3.515	-0.516	1.140	NA
Pararhodobacter	5.193	-0.515	0.787	8.530e-01
Abiotrophia	14.388	-0.511	0.608	7.912e-01
Phocaeicola	15.829	-0.511	0.616	7.928e-01
Legionella	1332.136	-0.508	0.234	2.121e-01
Rickettsiella	1.563	-0.508	0.769	NA
Halococcus	1.219	-0.501	0.883	NA
Aureococcus	5.453	-0.501	0.845	8.558e-01
Pimelobacter	6.825	-0.501	0.711	8.378e-01
Thyridium	3.802	-0.500	1.045	NA
Gallibacter	13.089	-0.497	0.786	8.558e-01
Xanthobacter	6.049	-0.494	0.647	7.958e-01
Planktothrix	6.905	-0.492	0.626	8.120e-01
Micrococcus	532.483	-0.491	0.269	3.471e-01
Cryptosporidium	15.505	-0.491	0.491	7.161e-01
Lysinibacillus	127.976	-0.487	0.367	5.738e-01
Salinibacterium	8.132	-0.486	0.620	7.958e-01
Rugamonas	25.020	-0.485	0.414	6.472e-01
Rummeliibacillus	3.322	-0.483	0.827	NA
Pyricularia	0.300	-0.483	2.360	NA

Noviherbaspirillum	23.982	-0.482	0.684	8.378e-01
Pseudoalteromonas	365.269	-0.482	0.200	1.377e-01
Kockovaella	3.506	-0.482	1.017	NA
Robiginitalea	0.271	-0.481	2.675	NA
Rickettsia	0.876	-0.481	1.308	NA
Cronobacter	1.550	-0.481	0.946	NA
Alkalispirochaeta	9160.805	-0.477	0.234	2.617e-01
Leptolyngbya	41.961	-0.476	0.402	6.422e-01
Tabrizicola	3.196	-0.476	0.818	NA
Planomonospora	0.421	-0.474	2.135	NA
Moraxella	437.707	-0.471	0.245	3.068e-01
Penaeicola	2.047	-0.469	0.893	NA
Primorskyibacter	0.613	-0.469	0.908	NA
Labrys	96.739	-0.463	0.382	6.358e-01
Polymorphum	0.621	-0.463	1.679	NA
Rodentibacter	0.334	-0.463	1.810	NA
Catenuloplanes	0.189	-0.462	2.909	NA
Yimella	7.431	-0.461	0.728	8.541e-01
Neorhizobium	3.954	-0.461	0.971	NA
Truepera	3.557	-0.461	1.073	NA
Rubrivirga	0.978	-0.461	2.152	NA
Schizosaccharomyces	3.316	-0.456	1.135	NA
Holdemanella	0.771	-0.455	1.823	NA
Brevibacterium	75.382	-0.453	0.314	5.378e-01
Micropruina	6.588	-0.452	0.968	8.751e-01
Alkalihalobacillus	3.555	-0.452	0.564	NA
Aquimonas	0.678	-0.452	1.984	NA
Cohnella	3.253	-0.447	0.692	NA
Psychromonas	1.967	-0.446	0.649	NA
Mycetohabitans	9.753	-0.446	0.449	7.156e-01
Pleurocapsa	1.217	-0.446	1.667	NA
Coprococcus	3.166	-0.442	1.352	NA
Ochrobactrum	77.175	-0.441	0.349	6.171e-01
Ottowia	13.988	-0.441	0.475	7.460e-01
Aureibaculum	51.891	-0.438	0.367	6.378e-01
Paucibacter	116.341	-0.437	0.230	3.166e-01
Exiguobacterium	51.512	-0.435	0.338	6.026e-01
Peribacillus	4.798	-0.432	0.922	NA
Enterovirga	5.894	-0.432	0.951	8.717e-01
Myxococcales	0.658	-0.431	2.170	NA
Acetobacterium	3.427	-0.428	0.955	NA
Bartonella	9.625	-0.428	0.556	8.191e-01
Oligoflexus	0.666	-0.427	2.903	NA
Kwoniella	6.912	-0.426	0.935	8.789e-01
Glaciihabitans	1.333	-0.426	1.414	NA
Puccinia	4.914	-0.425	0.786	NA
Rhodospirillaceae_genus	1.528	-0.420	1.334	NA
Glarea	5.933	-0.419	0.716	8.558e-01
Methylocystis	2.390	-0.413	0.865	NA
Sodalis-like	0.331	-0.411	0.977	NA
Zeimonas	0.839	-0.410	1.737	NA
Chromobacterium	7.322	-0.405	0.511	7.928e-01
Croceicoccus	0.401	-0.404	1.631	NA
Kaistella	17.043	-0.404	0.478	7.902e-01
Conyzicola	1.172	-0.404	1.196	NA
Pyrenophora	5.376	-0.404	0.878	8.686e-01
Fonticella	0.702	-0.403	2.352	NA
Variovorax	102.492	-0.399	0.244	4.449e-01
Diplodia	4.466	-0.398	0.826	NA

Pseudozyma	9.591	-0.397	0.717	8.611e-01
Tychonema	1.638	-0.394	1.131	NA
Photobacterium	11.366	-0.394	0.475	7.928e-01
Desulfovibrio	71087.628	-0.394	0.222	3.750e-01
Terracoccus	15.641	-0.391	0.781	8.699e-01
Mycosynbacter	1.754	-0.386	1.267	NA
Schlegelella	25.036	-0.382	0.435	7.697e-01
Sphingopyxis	66.886	-0.381	0.394	7.315e-01
Chthonobacter	0.278	-0.379	2.908	NA
Anaeromyxobacter	2.178	-0.379	1.257	NA
Dietzia	166.910	-0.377	0.327	6.544e-01
Pisolithus	0.864	-0.375	0.892	NA
Human_adenovirus_2	5.562	-0.373	0.748	8.686e-01
Pyruvatibacter	1.367	-0.371	0.755	NA
Desemzia	16.346	-0.370	0.510	8.323e-01
Pantoea	249.985	-0.369	0.178	2.488e-01
Pelistega	0.345	-0.369	2.909	NA
Brenneria	3.075	-0.368	0.412	NA
Alloalcanivorax	2.425	-0.368	0.980	NA
Malikia	0.578	-0.368	1.967	NA
Mannheimia	5.616	-0.366	0.527	8.218e-01
Acaricomes	2.228	-0.365	0.855	NA
Trichococcus	0.181	-0.365	2.800	NA
Rhodotorula	32.855	-0.364	0.551	8.530e-01
Caldimonas	5.324	-0.362	0.854	8.871e-01
Pinibacter	0.256	-0.361	2.708	NA
Westeberhardia	22.020	-0.361	0.417	7.697e-01
Oceanitalea	1.478	-0.361	1.538	NA
Thiobacillus	5.354	-0.358	0.767	8.686e-01
Chthoniobacter	1.949	-0.355	1.502	NA
Zoogloea	42.077	-0.354	0.349	7.056e-01
Pigmentiphaga	5.865	-0.353	0.740	8.686e-01
Singulisphaera	4.308	-0.352	0.833	NA
Hyphomonas	1.185	-0.348	1.672	NA
Thermorudis	0.331	-0.348	2.769	NA
Erwinia	905.375	-0.347	0.297	6.472e-01
Minwuia	0.928	-0.345	2.606	NA
Limosilactobacillus	14.701	-0.344	0.433	7.999e-01
Rhodoligotrophos	1.096	-0.343	1.626	NA
Lactiplantibacillus	4.583	-0.343	0.544	NA
Tatumella	7.235	-0.342	0.602	8.610e-01
Thermoanaerobacter	0.489	-0.341	2.906	NA
Thiomonas	1.166	-0.340	1.299	NA
Mucilaginibacter	56.373	-0.337	0.298	6.618e-01
Crenobacter	1.097	-0.336	0.886	NA
Modestobacter	77.884	-0.335	0.269	6.215e-01
Aphanizomenon	21.520	-0.333	0.262	5.686e-01
Acidipropionibacterium	19.534	-0.333	0.559	8.558e-01
Dyella	23.540	-0.331	0.348	7.368e-01
Alternaria	98.393	-0.329	0.227	5.331e-01
Pyrococcus	12.784	-0.329	0.576	8.558e-01
Sodalis	1.787	-0.324	0.400	NA
Nanosynsacchari	0.896	-0.322	1.442	NA
Lentzea	21.126	-0.322	0.300	6.765e-01
Pelomonas	2109.665	-0.322	0.177	3.471e-01
Lysobacter	182.469	-0.321	0.210	4.856e-01
Richelia	0.517	-0.321	1.756	NA
Sneathia	0.975	-0.321	2.318	NA
Haemophilus	188.386	-0.321	0.238	5.672e-01

Xanthomonas	75.563	-0.319	0.293	6.765e-01
Xylaria	0.604	-0.318	1.744	NA
Thermomonas	34.874	-0.314	0.261	6.358e-01
Methylophaga	0.781	-0.311	1.192	NA
Polyangium	64.134	-0.309	0.546	8.610e-01
Sphingobacterium	129.321	-0.308	0.306	7.151e-01
Silanimonas	0.294	-0.308	2.910	NA
Saccharomonospora	2.446	-0.307	0.957	NA
Yaniella	2.635	-0.307	1.267	NA
Baudoinia	11.960	-0.306	0.468	8.558e-01
Kirsten_murine_sarcoma_virus	1.366	-0.305	1.604	NA
Microdochium	4.060	-0.303	0.752	NA
Beijerinckia	0.400	-0.303	2.188	NA
Grimontella	3.770	-0.303	0.731	NA
Luteibacter	4.401	-0.300	0.812	NA
Leptomonas	2.477	-0.298	1.068	NA
Tamlana	6.081	-0.298	0.453	8.394e-01
Embleya	1.575	-0.297	1.420	NA
Fluoribacter	203.940	-0.297	0.273	6.765e-01
Hoyosella	1.261	-0.294	2.189	NA
Scleromatobacter	0.808	-0.293	2.396	NA
Prevotella	338.175	-0.284	0.303	7.416e-01
Thalassobius	5.691	-0.284	0.746	8.880e-01
Mycobacterium	323.882	-0.283	0.186	4.876e-01
Halovibrio	10.136	-0.280	0.622	8.841e-01
Collibacillus	0.630	-0.279	2.591	NA
Paenibacillus	158.355	-0.278	0.182	4.856e-01
Shinella	19.684	-0.278	0.541	8.686e-01
Loktanella	12.504	-0.277	0.262	6.852e-01
Vagococcus	0.603	-0.277	2.388	NA
Haliea	0.570	-0.276	1.354	NA
Caballeronia	39.742	-0.275	0.228	6.375e-01
Zymomonas	0.330	-0.275	2.908	NA
Asticcacaulis	114.652	-0.274	0.403	8.469e-01
Trujillella	0.289	-0.274	2.454	NA
Methanothermobacter	0.156	-0.274	2.910	NA
Luteimonas	79.273	-0.273	0.533	8.686e-01
Minicystis	1.137	-0.273	1.766	NA
Barnesiella	9.605	-0.271	0.370	8.218e-01
Parasaccharibacter	82.051	-0.271	0.214	6.057e-01
Cokeromyces	2.224	-0.270	0.486	NA
Rubrobacter	16.449	-0.268	0.634	8.871e-01
Solobacterium	7.141	-0.267	0.678	8.871e-01
Bacteroidetes	1.362	-0.267	1.761	NA
Providencia	883.343	-0.267	0.157	4.139e-01
Desulfosporosinus	5.604	-0.266	0.662	8.784e-01
Secondary	3.884	-0.262	0.460	NA
Burkholderiales	19.138	-0.261	0.425	8.558e-01
Algoriphagus	22.246	-0.259	0.331	8.146e-01
Amycolatopsis	5.663	-0.254	0.507	8.686e-01
Virgibacillus	35.407	-0.254	0.268	7.273e-01
Lichtheimia	16.959	-0.253	0.371	8.393e-01
Halomicroarcula	0.764	-0.253	1.039	NA
Romboutsia	3.447	-0.252	0.741	NA
Labilibaculum	7.087	-0.250	0.488	8.719e-01
Nocardia	68.412	-0.249	0.225	6.715e-01
Runella	0.324	-0.247	2.100	NA
Jaminaea	0.197	-0.246	2.910	NA
Buchnera	70221.134	-0.246	0.184	5.738e-01

Amorphotheca	7.737	-0.244	0.696	9.108e-01
Moraxellaceae_genus	14.760	-0.243	0.557	8.863e-01
Insolitispirillum	2.216	-0.242	1.117	NA
Piscicoccus	2.025	-0.242	1.477	NA
Lachnospiraceae_genus	22.274	-0.240	0.446	8.686e-01
Zimmermannella	7.604	-0.240	0.768	9.195e-01
Enterobacteria_phage_phi80_virus	4.380	-0.239	0.881	NA
Pseudoxanthomonas	75.899	-0.239	0.299	8.058e-01
Entamoeba	33.993	-0.238	0.478	8.686e-01
Aquincola	15.393	-0.238	0.491	8.686e-01
Eubacterium	18.755	-0.236	0.399	8.558e-01
Chryseobacterium	293.101	-0.236	0.206	6.544e-01
Aurantimonas	8.757	-0.232	0.547	8.863e-01
Rheinheimera	4037.545	-0.228	0.261	7.734e-01
Latilactobacillus	2.108	-0.228	1.306	NA
Nocardioides	721.043	-0.227	0.154	5.177e-01
Bavariicoccus	65.404	-0.227	0.323	8.378e-01
Rhodoferax	44.739	-0.226	0.317	8.378e-01
Kosakonia	21.067	-0.224	0.373	8.558e-01
Gayadomonas	2.348	-0.223	0.537	NA
Gleimia	229.791	-0.223	0.395	8.610e-01
Ewingella	0.845	-0.222	2.346	NA
Proteiniclasticum	1.099	-0.222	0.844	NA
Hoylesella	15.245	-0.221	0.557	8.934e-01
Salinarimonas	4.201	-0.219	0.369	NA
Variimorphobacter	0.190	-0.219	2.909	NA
Sporichthya	4.613	-0.218	1.039	NA
Lasiodiplodia	27.457	-0.218	0.352	8.558e-01
Nonomuraea	1.439	-0.218	1.156	NA
Paramagnetospirillum	1.385	-0.215	1.448	NA
Betaproteobacterium_JGI	8.316	-0.215	0.423	8.686e-01
Renibacterium	7.693	-0.215	0.785	9.345e-01
Gregarina	0.341	-0.214	2.755	NA
Gordonia	74.952	-0.214	0.359	8.558e-01
Hirsutella	0.227	-0.208	2.910	NA
Xanthomonadaceae_genus	3.912	-0.206	0.868	NA
Sporolactobacillus	110.272	-0.206	0.158	5.892e-01
Mobilicoccus	15.933	-0.204	0.572	9.151e-01
Thermothelomyces	0.628	-0.204	1.341	NA
Desulfocarbo	2.982	-0.204	0.930	NA
Bacteroides	124.758	-0.203	0.232	7.697e-01
Pararhizobium	9.293	-0.202	0.650	9.195e-01
Hephaestia	2.393	-0.201	1.203	NA
Nitrobacter	5.995	-0.201	0.659	9.168e-01
Alpha	67.476	-0.198	0.284	8.378e-01
Plasmopara	6.866	-0.197	0.683	9.195e-01
Comamonadaceae_genus	99.630	-0.197	0.238	7.928e-01
Brevundimonas	1147.754	-0.195	0.236	7.928e-01
Dactylosporangium	0.676	-0.195	2.902	NA
Marinifilum	38.998	-0.193	0.259	8.218e-01
Pedococcus	6.046	-0.193	0.703	9.201e-01
Jannaschia	2.279	-0.191	0.406	NA
Anatilimnocola	0.615	-0.189	2.905	NA
Micrococcaceae_genus	0.443	-0.188	1.833	NA
Neokomagataea	0.142	-0.187	2.910	NA
Pedobacter	122.644	-0.184	0.268	8.416e-01
Klebsiella	1531.102	-0.184	0.226	7.958e-01
Ruminococcus	28.348	-0.183	0.485	9.070e-01
Xenorhabdus	3.486	-0.182	0.422	NA

Varibaculum	3.474	-0.182	0.971	NA
Sandaracinobacter	0.418	-0.181	2.908	NA
Actinosynnema	3.106	-0.179	0.849	NA
Proteus	179.703	-0.178	0.348	8.686e-01
Alteribacter	6.236	-0.178	0.408	8.739e-01
Leclercia	50.086	-0.178	0.234	8.218e-01
Ensifer	36.688	-0.178	0.309	8.558e-01
Falsirhodobacter	4.108	-0.176	1.133	NA
Salmonella	451.456	-0.175	0.161	6.765e-01
Rhodobacter	87.074	-0.174	0.249	8.378e-01
Beijerinckiaceae_genus	37.739	-0.172	0.701	9.345e-01
Enhydrobacter	51.501	-0.171	0.282	8.558e-01
Plasmodium	260.330	-0.171	0.166	7.037e-01
Seramator	7.459	-0.171	0.736	9.315e-01
Filamentous	0.701	-0.170	1.784	NA
Pseudoclavibacter	10.937	-0.168	0.612	9.310e-01
Geminicoccus	65.368	-0.167	0.231	8.218e-01
Kaistia	3.343	-0.167	0.970	NA
Trabulsiella	0.462	-0.166	0.595	NA
Oceanospirillum	3.681	-0.162	0.599	NA
Rhodoplanes	8.916	-0.162	0.556	9.195e-01
Anabaena	1.596	-0.162	1.201	NA
Kluyvera	247.288	-0.161	0.188	7.796e-01
Pseudolabrys	0.793	-0.161	1.546	NA
Bowmanella	57.320	-0.160	0.328	8.719e-01
Deinococcus	166.493	-0.160	0.154	7.002e-01
Desarmillaria	3.626	-0.157	0.811	NA
Actinophytocola	2.028	-0.155	1.497	NA
Sanguibacter	2.066	-0.155	1.259	NA
Porphyromonadaceae_genus	12.235	-0.154	0.625	9.330e-01
Cedecea	62.532	-0.154	0.404	9.036e-01
Paraglaciecola	2.599	-0.154	0.645	NA
Gluconacetobacter	6.748	-0.154	0.574	9.195e-01
Rhizorhabdus	68.045	-0.153	0.294	8.686e-01
Eimeria	1.027	-0.150	1.030	NA
Microlunatus	62.692	-0.149	0.410	9.108e-01
Aestuariibaculum	1.315	-0.149	1.152	NA
Thauera	36.207	-0.149	0.237	8.558e-01
Edwardsiella	64.525	-0.148	0.329	8.826e-01
Agromyces	13.851	-0.146	0.415	9.154e-01
Alicyclobacillus	0.445	-0.146	2.171	NA
Limimaricola	3.153	-0.145	1.378	NA
Melittangium	0.138	-0.144	2.910	NA
UNVERIFIED_ORG:	13.458	-0.143	0.482	9.195e-01
Amaricoccus	20.347	-0.143	0.558	9.317e-01
Dinghuibacter	0.335	-0.142	2.909	NA
Dermabacter	5.640	-0.140	0.884	9.419e-01
Roseburia	27.841	-0.140	0.260	8.686e-01
Phaeovulum	30.793	-0.137	0.313	8.841e-01
Intestinirhabdus	0.996	-0.137	0.421	NA
Zavarzinella	0.480	-0.136	2.704	NA
Phycicoccus	66.900	-0.134	0.256	8.686e-01
Prevotellaceae_genus	6.860	-0.134	0.676	9.345e-01
Curvibacter	191.180	-0.134	0.147	7.616e-01
Halomonas	158.342	-0.133	0.265	8.686e-01
Tetrasphaera	10.317	-0.132	0.666	9.411e-01
Ezakiella	1.319	-0.131	1.834	NA
Allocoleopsis	0.836	-0.130	1.755	NA
Albitalea	0.203	-0.130	2.786	NA

Bacterium	36.502	-0.129	0.236	8.512e-01
Ruania	2.191	-0.126	1.105	NA
Hammondia	7.312	-0.126	0.477	9.189e-01
Psychromicrobium	11.336	-0.125	0.299	8.784e-01
Marmoricola	34.294	-0.124	0.544	9.345e-01
Dacryopinax	2.313	-0.122	1.332	NA
Arachnia	16.113	-0.121	0.551	9.350e-01
Fonsecaea	17.304	-0.119	0.587	9.385e-01
Musicola	448.106	-0.119	0.355	9.189e-01
Virgisporangium	0.184	-0.117	2.213	NA
Perlucidibaca	1.576	-0.115	0.957	NA
Anaerotardibacter	0.374	-0.114	2.020	NA
Xinfangfangia	1.289	-0.113	1.410	NA
Terriglobus	1.251	-0.113	1.209	NA
Pestalotiopsis	18.985	-0.111	0.520	9.345e-01
Phaeobacter	1.043	-0.109	0.831	NA
Clostridium	1001.246	-0.109	0.188	8.558e-01
Arthrobacter	566.114	-0.108	0.183	8.558e-01
Vescimonas	0.148	-0.108	2.910	NA
Wolbachia	68.134	-0.105	0.407	9.315e-01
Marinobacter	150.235	-0.104	0.245	8.880e-01
Seohaeicola	2.294	-0.102	1.308	NA
Promicromonospora	1.177	-0.101	1.694	NA
Rahnella	196.146	-0.098	0.300	9.195e-01
Sandarakinorhabdus	1.376	-0.098	1.916	NA
Solibacillus	6.613	-0.097	0.649	9.385e-01
Brevibacillus	1.631	-0.097	0.933	NA
Thioalkalivibrio	1.056	-0.096	1.278	NA
Paludibacterium	3.956	-0.095	0.770	NA
Sphingosinithalassobacter	0.506	-0.094	1.571	NA
Planctomyces	2.081	-0.094	1.041	NA
Cecembia	0.302	-0.091	2.198	NA
Eikenella	9.058	-0.089	0.566	9.512e-01
Stutzerimonas	32.547	-0.086	0.442	9.411e-01
Flagellatimonas	0.126	-0.084	2.910	NA
Blochmannia	14.483	-0.081	0.704	9.597e-01
Actinokineospora	43.083	-0.080	0.529	9.557e-01
Mesonia	2.526	-0.077	0.687	NA
Enterobacter	9658.205	-0.076	0.288	9.315e-01
Serratia	233.668	-0.074	0.250	9.201e-01
Gemmatimonas	2.554	-0.072	1.311	NA
Halalkalibacter	112.475	-0.071	0.304	9.345e-01
Elizabethkingia	205.340	-0.071	0.269	9.315e-01
Friedmanniella	3.725	-0.068	1.161	NA
Sporisorium	1.536	-0.068	1.287	NA
Gloeophyllum	13.463	-0.068	0.985	9.735e-01
Craterilacuibacter	127.435	-0.066	0.492	9.597e-01
Stenotrophomonas	1057.148	-0.065	0.113	8.558e-01
Oceaniovalibus	4.526	-0.065	0.713	NA
Oryzomicrobium	63.319	-0.064	0.414	9.514e-01
Rhodanobacter	6.156	-0.064	0.631	9.669e-01
Xenophilus	37.016	-0.063	0.327	9.385e-01
Glaciimonas	3.779	-0.063	0.987	NA
Acidiferrimicrobium	0.742	-0.062	1.654	NA
Sphingomonas	2004.426	-0.062	0.130	8.751e-01
Microbispora	62.361	-0.061	0.302	9.345e-01
Thermobacillus	0.214	-0.060	2.910	NA
Paraflavitalea	0.310	-0.060	2.908	NA
Methylopila	6.130	-0.060	0.842	9.735e-01

Oceanicella	2.913	-0.060	0.879	NA
Methylophilus	8.689	-0.059	0.539	9.597e-01
Fredinandcohnia	6.991	-0.059	0.605	9.665e-01
Pseudovibrio	0.453	-0.058	2.100	NA
Qingrenia	0.304	-0.056	2.907	NA
Parvularcula	2.074	-0.055	1.428	NA
Pusillimonas	1729.460	-0.055	0.260	9.385e-01
Citrobacter	14367.687	-0.054	0.290	9.419e-01
Raoultella	68.520	-0.053	0.319	9.419e-01
Actinomyces	520.914	-0.053	0.241	9.375e-01
Haliangium	2.455	-0.052	1.475	NA
Mesorhizobium	79.330	-0.052	0.194	9.310e-01
Leuconostoc	27.068	-0.051	0.320	9.514e-01
Faecalibacillus	0.308	-0.051	2.907	NA
Puia	26.661	-0.050	0.296	9.345e-01
Aliarcobacter	1.396	-0.047	2.169	NA
Achromobacter	178.806	-0.046	0.132	9.151e-01
Phytophthora	90.681	-0.045	0.427	9.665e-01
Methyloversatilis	103.042	-0.045	0.276	9.506e-01
Nannocystis	1.346	-0.042	1.770	NA
Telluria	20.994	-0.041	0.509	9.666e-01
Torulaspora	0.428	-0.041	2.905	NA
Morganella	1.022	-0.037	0.556	NA
Planctomycetes	0.540	-0.036	1.889	NA
Pseudorhodoplanes	0.962	-0.035	1.241	NA
Tannerella	323.303	-0.034	0.322	9.665e-01
Oribacterium	16.999	-0.034	0.590	9.726e-01
Marinobacterium	1297.016	-0.034	0.360	9.683e-01
Rothia	240.951	-0.033	0.256	9.597e-01
Erythrobacter	135.550	-0.032	0.227	9.345e-01
Gemmiger	1.474	-0.032	1.398	NA
Austwickia	2.277	-0.029	1.152	NA
Lactobacillus	101.572	-0.028	0.200	9.583e-01
Nitrospira	0.572	-0.027	1.933	NA
Oceanicola	3.328	-0.026	1.042	NA
Allobacillus	31.886	-0.025	0.362	9.712e-01
Abelson	3.843	-0.023	0.474	NA
Microcystis	7129.664	-0.022	0.366	9.735e-01
Klugiella	0.270	-0.022	2.908	NA
Glaciecola	6.760	-0.019	0.360	9.712e-01
Paracidovorax	147.082	-0.018	0.324	9.726e-01
Kingella	8.778	-0.017	0.513	9.572e-01
Pseudactinotalea	0.117	-0.017	2.911	NA
Desulfogranum	1.068	-0.017	1.474	NA
Phytobacter	11.988	-0.016	0.451	9.748e-01
Rhizobiales	18.512	-0.014	0.457	9.669e-01
Aquamicrobium	62.955	-0.014	0.335	9.735e-01
TM7	7.135	-0.012	0.647	9.802e-01
Thermosipho	4.351	-0.012	0.422	NA
Treponema	11.715	-0.011	0.512	9.735e-01
Cellulomonas	41.380	-0.011	0.339	9.738e-01
Blastomonas	151.122	-0.007	0.289	9.739e-01
Fictibacillus	317.200	-0.005	0.347	9.890e-01
Schaalia	24.329	-0.005	0.464	9.735e-01
FBR_murine_osteosarcoma_virus	1.393	-0.004	0.615	NA
Kinneretia	11.622	-0.004	0.507	9.735e-01
Akanthomyces	0.281	-0.001	2.907	NA
Mikella	0.000	0.000	0.000	NA
Ishikawaella	0.000	0.000	0.000	NA

Hafniaceae_genus	0.000	0.000	0.000	NA
Shigella_phage_SfIV_virus	0.000	0.000	0.000	NA
Escherichia_phage_500465-1_virus	0.000	0.000	0.000	NA
Enterobacteria_phage_DE3_virus	0.000	0.000	0.000	NA
Enterobacteria_phage_P7_virus	0.000	0.000	0.000	NA
Escherichia_phage_RCS47_virus	0.000	0.000	0.000	NA
Lagierella	0.000	0.000	0.000	NA
Rhabdobacter	0.000	0.000	0.000	NA
Kallipyga	0.000	0.000	0.000	NA
Escherichia_phage_500465-2_virus	0.000	0.000	0.000	NA
Escherichia_virus_Lambda_2G7b	0.000	0.000	0.000	NA
Escherichia_phage_TL-2011b_virus	0.000	0.000	0.000	NA
Escherichia_virus_Lambda_4A7	0.000	0.000	0.000	NA
Pusillibacter	0.000	0.000	0.000	NA
Escherichia_phage_Lambda_ev099_virus	0.000	0.000	0.000	NA
Klebsiella_phage_4_virus	0.000	0.000	0.000	NA
Stx2-converting_phage_1717_virus	0.000	0.000	0.000	NA
Cetobacterium	0.000	0.000	0.000	NA
Escherichia_phage_Cartapus_virus	0.000	0.000	0.000	NA
SsRNA_phage_SRR5466337_3_virus	0.000	0.000	0.000	NA
Escherichia_phage_Lambda_ev207_virus	0.000	0.000	0.000	NA
Escherichia_virus_Lambda_1H12	0.000	0.000	0.000	NA
Tropicibacter	0.000	0.000	0.000	NA
Stx2-converting_phage_Stx2a_WGPS2_virus	0.000	0.000	0.000	NA
Escherichia_phage_Lambda_ev243_virus	0.000	0.000	0.000	NA
Couchioplanes	0.000	0.000	0.000	NA
Escherichia_virus_Lambda_2H10	0.000	0.000	0.000	NA
Thermobrachium	0.000	0.000	0.000	NA
Deferrisoma	0.000	0.000	0.000	NA
Escherichia_phage_D6_virus	0.000	0.000	0.000	NA
Enterobacteria_phage_Sf6_virus	0.000	0.000	0.000	NA
Gloeothece	0.000	0.000	0.000	NA
Escherichia_phage_520873_virus	0.000	0.000	0.000	NA
Viadribacter	0.000	0.000	0.000	NA
Oceanotoga	0.000	0.000	0.000	NA
Salmonella_phage_SJ46_virus	0.000	0.000	0.000	NA
Phaseolus_vulgaris_endornavirus	0.000	0.000	0.000	NA
Paludicola	0.000	0.000	0.000	NA
Kaustia	0.000	0.000	0.000	NA
Kushneria	83.057	0.003	0.276	9.514e-01
Coleofasciculus	1.811	0.004	1.256	NA
Lamprocystis	7995.423	0.005	0.330	9.890e-01
Acinetobacter	11818.492	0.005	0.154	9.802e-01
Paracoccus	735.536	0.005	0.172	9.739e-01
Streptosporangium	49.775	0.005	0.237	9.739e-01
Escherichia_phage_phiV10_virus	2.464	0.005	0.984	NA
Actinomadura	901.640	0.006	0.151	9.756e-01
Ciceribacter	3.850	0.007	0.808	NA
Izhakiella	15.037	0.009	0.840	9.735e-01
Spirilliplanes	0.536	0.009	2.910	NA
Bordetella	18.960	0.011	0.384	9.802e-01
Drechmeria	0.521	0.011	1.588	NA
Prauserella	0.186	0.012	2.909	NA
Toxoplasma	250.863	0.012	0.152	9.712e-01
Candida	5.852	0.012	0.625	9.195e-01
Myxococcus	31.942	0.015	0.261	9.195e-01
Frateuria	2.007	0.015	0.768	NA
Leucothrix	10.440	0.015	0.474	9.683e-01
Pseudopropionibacterium	6.216	0.016	0.682	9.712e-01

Actinobacillus	273.019	0.016	0.342	9.735e-01
Pasteurella	1.152	0.018	0.576	NA
Algibacter	5.117	0.019	0.550	9.557e-01
Halorubrum	12.131	0.019	0.426	9.669e-01
Isoptericola	31.305	0.019	0.237	9.315e-01
Ktedonobacter	0.419	0.020	2.905	NA
Propionibacteriaceae_genus	1.663	0.020	1.306	NA
Winogradskyella	5.235	0.021	0.380	8.218e-01
Prosthecomicrobium	0.437	0.023	2.085	NA
Armatimonas	0.426	0.027	2.337	NA
Rhodomicrobium	7.805	0.028	0.460	9.665e-01
Methylocapsa	1.039	0.029	1.172	NA
Sedimentitalea	30.138	0.030	0.268	8.880e-01
Ignavibacterium	0.501	0.030	2.906	NA
Aquihabitans	4.214	0.031	1.078	NA
Bosea	160.841	0.032	0.189	9.317e-01
Slackia	0.879	0.033	1.782	NA
Stakelama	0.615	0.034	1.575	NA
Atopobium	11.220	0.034	0.646	9.735e-01
Chromohalobacter	65.014	0.036	0.210	9.385e-01
Ruficoccus	65.274	0.036	0.352	9.665e-01
Ancylomarina	7.570	0.037	0.560	9.458e-01
Shigella	210548.549	0.038	0.302	9.618e-01
Chaetomium	2.688	0.038	1.050	NA
Glaciibacter	18.801	0.039	0.656	9.735e-01
Calidithermus	19.574	0.040	0.538	9.726e-01
Haladaptatus	2.468	0.040	0.784	NA
Actinotalea	15.791	0.040	0.700	9.735e-01
Serpentinimonas	0.193	0.041	2.910	NA
Paracnuella	0.644	0.041	1.253	NA
Krasilnikoviella	0.109	0.042	2.911	NA
Trypanosoma	14.029	0.042	0.551	9.572e-01
Yarrowia	13.249	0.043	0.776	9.735e-01
Actirhodobacter	0.376	0.044	2.703	NA
Acidovorax	744.728	0.046	0.109	8.871e-01
Tenebrionibacter	0.381	0.046	0.980	NA
Frankia	114.940	0.048	0.284	9.458e-01
Aquibium	0.920	0.048	1.674	NA
Falsiroseomonas	137.429	0.049	0.254	9.385e-01
Flavobacterium	706.963	0.049	0.155	9.195e-01
Aerococcus	77.226	0.050	0.287	9.419e-01
Actinoalloteichus	0.689	0.051	1.327	NA
Escherichia	61277.548	0.053	0.298	9.458e-01
Fusobacterium	181.144	0.053	0.212	9.315e-01
Dolosigranulum	11.557	0.055	0.681	9.712e-01
Microbacterium	1220.119	0.055	0.137	8.934e-01
Pseudochrobactrum	75.819	0.055	0.452	9.597e-01
Ustilago	1.359	0.056	1.302	NA
Photorhabdus	47.052	0.059	0.368	9.385e-01
Pochonia	0.554	0.059	1.501	NA
Human_endogenous_retrovirus	5.191	0.059	0.536	9.597e-01
Kytococcus	23.988	0.061	0.491	9.597e-01
Cardiobacterium	13.923	0.064	0.565	9.665e-01
Cupidesulfovibrio	28.579	0.064	0.402	9.458e-01
Rhodofomes	4.456	0.068	0.807	NA
Delta	4.266	0.068	0.590	NA
Thermogemmata	0.679	0.068	2.165	NA
Paraconexibacter	1.837	0.069	1.112	NA
Moritella	186.504	0.069	0.166	8.863e-01

Neobacillus	44.891	0.070	0.242	9.195e-01
Actinoplanes	35.115	0.070	0.366	9.411e-01
Bacillus	2979.064	0.071	0.120	8.558e-01
Duffyella	4.145	0.071	0.742	NA
Komarekiella	0.170	0.072	2.910	NA
Cyberlindnera	2.022	0.072	1.231	NA
Phormidium	0.945	0.073	1.512	NA
Maribellus	40.625	0.074	0.345	9.345e-01
Ornithinicoccus	0.159	0.074	2.830	NA
Cupriavidus	310.513	0.074	0.202	9.108e-01
Frigoribacterium	15.155	0.075	0.629	9.654e-01
Nostoc	288.872	0.078	0.198	8.994e-01
Alcanivorax	267.141	0.078	0.230	9.168e-01
Arsenicicoccus	2.165	0.078	1.004	NA
Franconibacter	0.683	0.078	1.247	NA
Erythrobacteraceae_genus	1.392	0.079	1.312	NA
Xylella	2.546	0.080	0.607	NA
Perkinsus	5.227	0.081	0.585	9.712e-01
PreXMRV-1_provirus_complete	4.785	0.081	0.493	NA
Millisia	1.115	0.082	2.033	NA
Aliicoccus	0.581	0.084	2.112	NA
Gramella	1.113	0.085	0.807	NA
Calidifontimicrobium	0.522	0.085	2.907	NA
Crocosphaera	13.832	0.088	0.398	9.345e-01
Terrisporobacter	682.815	0.088	0.323	9.310e-01
Selenomonas	7.222	0.089	0.547	9.361e-01
Marinithermofilum	29.006	0.091	0.264	9.112e-01
Roseateles	324.440	0.092	0.174	8.686e-01
Flexivirga	2.311	0.092	1.101	NA
Mediannikoviicoccus	0.103	0.092	2.911	NA
Pedomonas	4.318	0.092	1.071	NA
Type-C_symbiont_of_Plautia_stali	0.212	0.094	2.743	NA
Propionibacterium_phage_PHL117M01_virus	0.268	0.094	2.909	NA
Naumannella	5.114	0.095	0.849	NA
Shewanella	593.636	0.095	0.146	8.558e-01
Lentibacillus	34.801	0.096	0.264	9.036e-01
Sphingobium	158.875	0.099	0.194	8.686e-01
Chromatium	57.450	0.099	0.208	8.751e-01
Williamsia	113.724	0.100	0.447	9.361e-01
Vulcaniibacterium	2.155	0.100	1.585	NA
Histoplasma	0.752	0.101	1.238	NA
Geomicrobium	0.127	0.103	2.911	NA
Acidiplasma	17.926	0.103	0.333	9.195e-01
Agrobacterium	192.449	0.106	0.190	8.558e-01
Kurthia	83.993	0.107	0.347	9.195e-01
Jeotgalicoccus	20.422	0.108	0.374	9.195e-01
Alkalicoccobacillus	0.836	0.110	0.756	NA
Comamonas	377.215	0.111	0.215	8.686e-01
Cytobacillus	13.792	0.112	1.179	9.682e-01
Aquisphaera	1.528	0.114	1.228	NA
Rhodopseudomonas	31.634	0.114	0.351	9.195e-01
Hyphomicrobium	34.815	0.116	0.292	8.960e-01
Lachnellula	2.618	0.116	0.755	NA
Aequorivita	187.348	0.117	0.193	8.558e-01
Parasutterella	0.614	0.118	1.304	NA
Nitrosomonas	5.354	0.119	0.802	9.665e-01
Sinirhodobacter	15.801	0.119	0.331	7.674e-01
Mycobacteriaceae_genus	41.727	0.121	0.226	8.558e-01
Megamonas	3.586	0.122	1.011	NA

Ruegeria	9.018	0.123	0.689	9.407e-01
Rufibacter	1.023	0.124	1.630	NA
Fimbriiglobus	2.293	0.124	1.247	NA
Diaphorobacter	35.714	0.124	0.379	9.189e-01
Beggiatoa	32.646	0.125	0.242	8.686e-01
Pectinatus	4.713	0.125	0.697	NA
Atlantibacter	14.246	0.126	0.266	8.751e-01
Microcoleus	16.615	0.128	0.551	9.195e-01
Lipomyces	0.225	0.129	2.780	NA
Acidobacteria	2.858	0.131	0.904	NA
Azospira	27.304	0.131	0.311	8.751e-01
Viridilinea	0.240	0.133	2.911	NA
Effusibacillus	0.123	0.134	2.911	NA
Herbaspirillum	591.387	0.136	0.203	8.512e-01
Proteus_phage_VB_PmiS-Isfahan_virus	1.743	0.138	0.531	NA
Janibacter	151.607	0.138	0.186	8.218e-01
Cereal_yellow_dwarf_virus	0.223	0.138	2.909	NA
Catonella	3.258	0.139	0.927	NA
Thalassiosira	9.266	0.139	0.575	9.315e-01
Methylorubrum	226.898	0.141	0.170	7.928e-01
Brachymonas	13.279	0.143	0.443	9.195e-01
Shimia	358.445	0.143	0.301	8.751e-01
Streptococcus	1476.802	0.146	0.295	8.714e-01
Solihabitans	20.875	0.147	0.262	8.378e-01
Colletotrichum	46.845	0.147	0.355	8.880e-01
Tissierella	290.882	0.149	0.190	8.110e-01
Aggregatibacter	18.736	0.151	0.427	9.108e-01
Planctopirus	0.483	0.151	1.462	NA
Pseudorhodoferax	8.912	0.151	0.650	9.345e-01
Actinomycetospora	16.751	0.152	0.510	9.195e-01
Phototrophicus	0.608	0.152	2.176	NA
Aedoeadaptatus	2.659	0.153	1.331	NA
Pinisolibacter	11.572	0.153	0.279	8.218e-01
Rubrivivax	73.057	0.154	0.340	8.784e-01
Pedosphaera	0.186	0.156	2.910	NA
Staphylococcus	2266.839	0.157	0.310	8.686e-01
Limnohabitans	19.440	0.157	0.341	8.751e-01
Enterobacteriaceae_genus	24.824	0.158	0.285	8.558e-01
Sagittula	2.597	0.160	1.212	NA
Pyrinomonas	9.505	0.160	0.879	9.419e-01
Hydrotalea	0.322	0.161	2.628	NA
Burkholderia	2610.408	0.161	0.151	6.852e-01
Rhodospirillales	0.991	0.162	1.671	NA
Rhodococcus	464.902	0.163	0.182	7.674e-01
Methylibium	9.563	0.163	0.830	9.411e-01
Thiohalocapsa	29.701	0.165	0.326	8.686e-01
Humibacter	0.118	0.166	2.911	NA
Siccibacter	0.129	0.166	2.302	NA
Chitinophaga	7.848	0.170	0.609	9.310e-01
Winslowiella	0.287	0.174	2.911	NA
Methylomonas	1.557	0.175	0.579	NA
Mycoavidus	0.989	0.176	1.741	NA
Neptuniibacter	0.092	0.177	2.911	NA
Bhargavaea	9.142	0.177	0.600	9.189e-01
Bipolaris	7.199	0.179	0.781	9.345e-01
Zoogloeaceae_genus	0.238	0.180	2.908	NA
Thioflexithrix	10.033	0.180	0.450	8.880e-01
Tricharina	1.265	0.180	1.198	NA
Cyclobacterium	9.163	0.180	0.363	8.558e-01

Salinimicrobium	20.796	0.181	0.463	8.789e-01
Liquorilactobacillus	0.992	0.183	1.643	NA
Arenimonas	1.644	0.187	1.335	NA
Aphanothece	13.799	0.187	0.427	8.852e-01
Bradyrhizobium	4376.605	0.188	0.177	6.852e-01
Demequina	2.047	0.192	1.077	NA
Paraclostridium	10.370	0.192	0.695	9.310e-01
Murine_type_C_virus	8.935	0.192	0.401	8.558e-01
Brucella	13.265	0.193	0.432	8.834e-01
Terrabacter	109.045	0.196	0.235	7.905e-01
Azospirillum	271.311	0.196	0.341	8.558e-01
Ruminococcaceae_genus	14.329	0.197	0.374	8.686e-01
Alcaligenes	8.410	0.198	0.605	9.195e-01
Leptothrix	258.760	0.200	0.333	8.558e-01
Pseudorhizobium	3.953	0.200	1.093	NA
Tumebacillus	31.849	0.201	0.384	8.686e-01
Methylobacterium	5697.290	0.201	0.182	6.715e-01
Mycoplasmopsis	1.529	0.203	1.110	NA
Lacrimispora	0.872	0.204	1.722	NA
BeAn_58058_virus	0.384	0.204	1.118	NA
Microcella	4.645	0.204	0.850	NA
Lacticaseibacillus	6.204	0.206	0.664	9.213e-01
Paraburkholderia	685.653	0.209	0.160	5.878e-01
Hydrogenophilus	2.399	0.209	1.510	NA
Leifsonia	76.614	0.209	0.381	8.686e-01
Pasteurellaceae_genus	2.823	0.210	0.906	NA
Acidaminococcus	0.185	0.210	2.910	NA
Kallotenue	98.738	0.211	0.578	9.112e-01
Atopomonas	30.225	0.214	0.279	8.151e-01
Aquabacterium	1382.076	0.215	0.243	7.697e-01
Hydrobacter	0.593	0.215	1.968	NA
Cereibacter	5.029	0.216	0.784	NA
Citreimonas	0.088	0.216	2.911	NA
Longimicrobium	6.592	0.218	1.017	9.407e-01
Lautropia	241.645	0.218	0.204	6.852e-01
Propionibacterium	5198.439	0.220	0.283	8.151e-01
Hartmannibacter	0.288	0.221	2.907	NA
Thioclava	0.294	0.222	2.319	NA
Rhizobium	451.252	0.224	0.160	5.468e-01
Neoantrodia	9.542	0.226	0.874	9.315e-01
Malassezia	1176.135	0.227	0.284	8.058e-01
Neoroseomonas	0.161	0.228	2.910	NA
Marinilabiliaceae_genus	2.283	0.229	0.781	NA
Ichthyophthirius	11.762	0.230	0.507	8.751e-01
Rhodocista	0.302	0.231	1.675	NA
Flavobacteriaceae_genus	0.778	0.232	1.418	NA
Hoaglandella	0.398	0.235	2.161	NA
Planococcus	196.283	0.237	0.194	6.326e-01
Piscinibacter	118.721	0.240	0.360	8.512e-01
Afipia	1012.669	0.241	0.243	7.161e-01
Lactococcus	112.493	0.242	0.400	8.558e-01
Gardnerella	26.116	0.242	0.583	8.880e-01
Methylobrevis	19.272	0.243	0.411	8.558e-01
Mameliella	2.131	0.243	1.124	NA
Polaromonas	23.598	0.246	0.299	7.928e-01
Spirosoma	11.388	0.249	0.611	8.916e-01
Paecilomyces	3.711	0.249	0.900	NA
Thermus	128.332	0.250	0.409	8.558e-01
Ralstonia	14254.930	0.253	0.160	4.610e-01

Azorhizobium	2.897	0.254	0.917	NA
Granulicatella	27.984	0.256	0.436	8.558e-01
Capnocytophaga	41.728	0.256	0.302	7.886e-01
Enterococcus	638.409	0.256	0.168	4.856e-01
Gallintestinimicrobium	0.837	0.259	2.604	NA
Serinicoccus	13.203	0.259	0.757	9.168e-01
Sediminibacterium	2.811	0.260	1.146	NA
Enterobacteria_phage_T4_virus	2.981	0.260	1.115	NA
Empedobacter	31.761	0.260	0.370	8.378e-01
harvey_murine_sarcoma_virus	0.947	0.263	0.459	NA
Huaxiibacter	3.774	0.264	0.922	NA
Sulfolobus	2.237	0.265	0.452	NA
Filomicrobium	2.369	0.267	1.076	NA
Winkia	10.745	0.268	0.772	9.168e-01
Morococcus	313.297	0.270	0.272	7.161e-01
Carboxylicivirga	0.110	0.270	2.912	NA
Klenkia	7.551	0.270	0.725	8.960e-01
Skermanella	18.493	0.270	0.538	8.686e-01
Rectinema	0.081	0.273	2.912	NA
Thermoanaerobacterium	21.481	0.273	0.953	9.276e-01
Laccaria	3.822	0.277	0.732	NA
Mangrovicoccus	5.222	0.278	0.898	9.195e-01
Blattabacterium	0.399	0.279	2.140	NA
Paucilactobacillus	8.021	0.281	0.552	8.686e-01
Rosenbergiella	1.613	0.284	0.697	NA
Seonamhaeicola	12.679	0.286	0.416	8.191e-01
Paracandidimonas	0.784	0.286	2.108	NA
Defluviicoccus	0.374	0.287	2.346	NA
Levilactobacillus	89.361	0.288	0.471	8.558e-01
Olsenella	7.411	0.289	0.798	9.151e-01
Pseudacidovorax	63.479	0.292	0.308	7.368e-01
Pandoraea	33.582	0.293	0.314	7.460e-01
Paludifilum	42.859	0.293	0.223	5.814e-01
Parainfluenza_virus_5	6.913	0.293	1.137	9.345e-01
Companilactobacillus	0.505	0.294	2.342	NA
Dissoconium	0.190	0.295	2.912	NA
Gilbertella	13763.122	0.295	0.232	6.057e-01
Neisseria	439.766	0.295	0.209	5.388e-01
Azohydromonas	15.191	0.296	0.480	8.558e-01
Cryptomonas	1.703	0.296	1.244	NA
Cutibacterium	314.810	0.297	0.285	6.958e-01
Corynebacterium	1916.937	0.299	0.225	5.738e-01
Azovibrio	1.264	0.299	1.452	NA
Thermomicrobium	3.852	0.303	0.854	NA
Rudaea	1.559	0.303	0.732	NA
Alistipes	1.236	0.304	1.513	NA
Vibrio	638.107	0.305	0.224	5.623e-01
Dysgonamonadaceae_genus	13.608	0.307	0.721	8.871e-01
Paenirhodobacter	23.041	0.308	0.351	7.674e-01
Sandaracinus	1.493	0.308	1.607	NA
Peptoclostridium	0.886	0.308	1.720	NA
Mus_musculus_mobilized_virus	23.218	0.314	0.324	7.151e-01
Murine_osteosarcoma_virus	2.353	0.316	0.661	NA
Delftia	511.917	0.316	0.191	4.393e-01
Luteitalea	7.610	0.317	0.901	9.168e-01
Propionimicrobium	58.601	0.317	0.452	8.378e-01
Salinisphaera	0.252	0.317	0.902	NA
Pseudomassariella	0.665	0.319	2.206	NA
Wigglesworthia	1.349	0.319	0.840	NA

Carideicomes	0.567	0.319	1.159	NA
Parabacteroides	28.004	0.319	0.340	7.416e-01
Methylococcus	3.490	0.321	0.716	NA
Pontibrevibacter	0.084	0.323	2.912	NA
Frigidibacter	67.900	0.323	0.281	6.544e-01
Bifidobacterium	47.754	0.323	0.339	7.368e-01
Lentisphaera	29.992	0.323	0.264	6.229e-01
Oceanobacillus	36.670	0.325	0.283	6.472e-01
Chryseosolibacter	0.194	0.326	2.909	NA
Miltoncostaea	4.350	0.327	1.330	NA
Aureobasidium	12.765	0.328	0.584	8.611e-01
Yonghaparkia	1.392	0.328	1.371	NA
Sulfitobacter	6.435	0.330	0.458	8.378e-01
Thalassotalea	1.950	0.330	0.944	NA
Betaproteobacterium_FWI2	1603.185	0.331	0.338	7.222e-01
Janthinobacterium	149.394	0.332	0.242	5.613e-01
Tepidimonas	39.113	0.334	0.495	8.491e-01
Cercospora	12.008	0.335	0.540	8.558e-01
Brachybacterium	121.244	0.336	0.407	7.928e-01
Larkinella	0.768	0.339	1.602	NA
Arsenophonus	1.327	0.340	0.616	NA
Saccharopolyspora	62.086	0.345	0.210	4.312e-01
Pseudokineococcus	5.519	0.354	1.238	9.315e-01
Linderina	1.155	0.357	1.056	NA
Dermacoccus	44.902	0.357	0.302	6.438e-01
Besnoitia	7.844	0.358	0.429	7.796e-01
Duganella	52.030	0.360	0.339	6.852e-01
Aggregatilinea	0.100	0.361	2.911	NA
Pseudophaeobacter	4.670	0.362	0.759	NA
Afifella	1.377	0.365	1.754	NA
Nitrotoga	4.783	0.366	0.573	NA
Mangrovibacter	0.437	0.366	0.663	NA
Parasphingopyxis	15.233	0.369	0.633	8.558e-01
Chroococcidiopsis	2.687	0.374	0.889	NA
Gallionella	0.436	0.375	0.997	NA
Anaerobiospirillum	0.376	0.379	2.611	NA
Betaproteobacterium_AAP51	11.054	0.383	0.547	8.378e-01
Orbilia	10.608	0.383	0.849	8.826e-01
Ligilactobacillus	14.208	0.384	0.636	8.558e-01
Leucobacter	21.363	0.386	0.394	7.222e-01
Ferrovum	0.569	0.387	2.388	NA
Aphanomyces	5.189	0.387	0.589	8.558e-01
Motilimonas	7.460	0.391	0.534	8.218e-01
Candidate	14.431	0.391	0.350	6.715e-01
Flintibacter	0.071	0.392	2.912	NA
Sandaracinobacteroides	6.329	0.394	0.760	8.686e-01
Niveispirillum	2.188	0.394	1.438	NA
Propioniferax	0.646	0.395	2.889	NA
Nocardiopsis	6.413	0.397	0.966	8.934e-01
Schumannella	0.440	0.398	2.096	NA
Kineococcus	53.880	0.400	0.673	8.558e-01
Roseovarius	20.641	0.401	0.352	6.479e-01
Fimbriimonas	2.579	0.402	1.145	NA
Desulfitobacterium	6.033	0.402	0.774	8.686e-01
Paenacidovorax	3.180	0.402	0.968	NA
Leptotrichia	40.411	0.404	0.329	6.308e-01
Fortiea	2.157	0.404	1.307	NA
Arthroderma	0.451	0.409	1.193	NA
Dubosiella	0.165	0.412	2.910	NA

Segetibacter	3.596	0.412	1.245	NA
Nitrosospira	2.562	0.412	1.531	NA
Bacidia	3.842	0.412	0.895	NA
Nesterenkonia	69.289	0.413	0.270	4.856e-01
Acuticoccus	38.672	0.413	0.249	4.070e-01
Ancylobacter	5.043	0.415	0.938	NA
Verticillium	0.616	0.418	1.621	NA
Allomeiothermus	372.615	0.418	0.599	8.378e-01
Leptospira	69.642	0.420	0.274	4.856e-01
Armatimonadetes	144.045	0.421	0.646	8.558e-01
Puteibacter	1.005	0.423	1.141	NA
Pichia	0.685	0.424	1.347	NA
Niallia	0.268	0.425	1.972	NA
Dongshaea	0.131	0.426	2.910	NA
Spiribacter	38.734	0.426	0.420	7.138e-01
Eremococcus	4.175	0.427	0.870	NA
Coraliihabitans	7.777	0.427	0.587	8.323e-01
Pauljensenia	155.649	0.428	0.434	7.205e-01
Mycoplasma	1.308	0.428	0.808	NA
Nanosynbacter	12.801	0.431	0.634	8.442e-01
Lelliottia	2.324	0.436	0.625	NA
Yeguia	0.064	0.437	2.912	NA
Wolinella	0.895	0.438	1.842	NA
Homoserinimonas	5.839	0.439	0.745	8.558e-01
Hoeflea	3.595	0.440	0.942	NA
Aestuariimicrobium	3.255	0.443	0.974	NA
Bifidobacteriaceae_genus	8.416	0.444	0.910	8.739e-01
Congregibacter	0.755	0.444	0.985	NA
Coniosporium	2.486	0.447	1.447	NA
Arsukibacterium	0.805	0.452	1.168	NA
Lachnoclostridium	1.068	0.452	2.191	NA
Bdellovibrio	0.865	0.453	1.323	NA
Rhizobacter	51.463	0.459	0.306	4.912e-01
Postia	8.620	0.462	0.792	8.558e-01
Youxingia	0.114	0.462	2.912	NA
Pelosinus	4.274	0.464	0.860	NA
Abditibacterium	5.942	0.464	0.961	8.751e-01
Anaerobacillus	0.305	0.465	2.907	NA
Acidocella	0.541	0.465	1.509	NA
Allorhizobium	0.157	0.465	2.910	NA
Metarhizium	16.962	0.472	0.545	7.796e-01
Peptococcus	0.489	0.472	2.905	NA
Caldicellulosiruptor	0.237	0.473	2.908	NA
Penicilliopsis	0.668	0.473	2.312	NA
Lonsdalea	0.189	0.473	0.910	NA
Dysosmobacter	0.322	0.474	2.333	NA
Naegleria	20.303	0.474	0.488	7.258e-01
Snodgrassella	4.153	0.475	1.187	NA
Burkholderiaceae_genus	42.128	0.478	0.335	5.378e-01
Methylotenera	11.956	0.481	0.648	8.218e-01
Hafnia	2.018	0.482	0.468	NA
Conchiformibius	7.052	0.486	0.786	8.558e-01
Caulobacter	1070.743	0.488	0.232	2.409e-01
Pseudoramibacter	88.151	0.491	0.507	7.302e-01
Euzebya	0.588	0.491	1.258	NA
Bergeyella	0.183	0.495	2.865	NA
Fluviicola	6.799	0.496	0.835	8.558e-01
Solimonas	5.862	0.496	0.866	8.558e-01
Pseudonocardia	81.981	0.498	0.383	5.948e-01

Halobacteriovorax	1.982	0.499	0.590	NA
Lentihominibacter	0.331	0.499	2.148	NA
Altererythrobacter	4.874	0.499	0.920	NA
Ferruginibacter	1.535	0.500	1.236	NA
Gemella	167.060	0.501	0.413	6.358e-01
Phreatobacter	3.203	0.503	0.877	NA
Isoalcanivorax	1.062	0.505	1.740	NA
Xylophilus	6.954	0.507	0.505	7.046e-01
Tetrahymena	5.684	0.508	0.665	8.218e-01
Pseudohongiella	0.062	0.510	2.912	NA
Aquitalea	0.973	0.516	0.718	NA
Mycolicibacterium	115.168	0.516	0.317	4.490e-01
Tistrella	0.240	0.517	2.909	NA
Gulosibacter	3.895	0.525	1.084	NA
Archangium	2.807	0.526	0.793	NA
Jeotgalibacillus	1.208	0.527	1.048	NA
Mesomycoplasma	0.350	0.528	2.354	NA
Peptostreptococcus	6.314	0.528	0.669	8.112e-01
Bryobacter	0.568	0.530	2.900	NA
Leishmania	4.184	0.537	0.980	NA
Chitinophagaceae_genus	0.335	0.538	2.643	NA
Desertimonas	10.044	0.540	0.887	8.558e-01
Alkaliphilus	2.459	0.543	1.497	NA
Gluconobacter	9.302	0.547	0.635	7.786e-01
Paludisphaera	3.132	0.552	0.835	NA
Negativicoccus	0.811	0.553	2.907	NA
Macrococcus	3.784	0.553	0.724	NA
Ogataea	1.444	0.554	1.314	NA
Plantactinospora	0.899	0.556	1.272	NA
Meiothermus	30.189	0.556	0.557	7.161e-01
Tolumonas	0.873	0.558	1.896	NA
Caulobacteraceae_genus	1.255	0.559	1.906	NA
Gaiella	11.733	0.560	0.963	8.558e-01
Glaesserella	0.891	0.564	0.900	NA
Fusibacter	1.572	0.564	1.684	NA
Phytoplasma	68.366	0.568	0.307	3.396e-01
Salicibibacter	0.350	0.569	2.907	NA
Fretibacterium	3.170	0.575	1.088	NA
Luteipulveratus	0.361	0.579	2.611	NA
Frisingicoccus	0.052	0.585	2.912	NA
Campylobacter	38.374	0.587	0.283	2.488e-01
Aquimarina	1.439	0.591	1.785	NA
Herminiimonas	2.008	0.592	1.293	NA
Agreia	0.629	0.593	2.010	NA
Goekera	0.470	0.594	2.600	NA
Rubellimicrobium	30.281	0.594	0.533	6.715e-01
Wenxinia	0.425	0.597	1.856	NA
Scandinavium	0.642	0.597	0.602	NA
Anaeromassilibacillus	0.075	0.609	2.912	NA
Lachnoanaerobaculum	14.139	0.610	0.399	4.856e-01
Acidothermus	1.557	0.612	1.616	NA
Dictyostelium	8.462	0.612	0.801	8.218e-01
Lichenibacterium	2.044	0.612	1.401	NA
Haematobacter	4.763	0.613	1.196	NA
Marinilactibacillus	3.912	0.615	1.067	NA
Acetobacter	16.394	0.615	0.329	3.285e-01
Alicycliphilus	12.250	0.622	0.443	5.396e-01
Megasphaera	17.752	0.623	0.666	7.460e-01
Geomonas	0.050	0.626	2.912	NA

Amygdalobacter	0.251	0.628	2.909	NA
Roseisolibacter	3.912	0.629	1.081	NA
Propionibacterium_phage_PHL041M10_virus	0.119	0.630	2.911	NA
Soleaferrea	0.148	0.631	2.910	NA
Mycetocola	0.892	0.631	1.247	NA
Idiomarina	0.506	0.635	2.617	NA
Exophiala	31.121	0.639	0.575	6.715e-01
Oligella	1.088	0.645	2.110	NA
Fervidibacillus	1.878	0.646	2.065	NA
Jiangella	1.073	0.651	1.579	NA
Rhodovulum	0.928	0.651	1.323	NA
Aggregicoccus	0.395	0.653	2.908	NA
Rhodophyticola	0.082	0.653	2.912	NA
Gynurincola	0.054	0.653	2.912	NA
Robbsia	0.852	0.654	1.318	NA
Anaerosphaera	0.551	0.654	2.179	NA
Spleen_focus-forming_virus	3.983	0.657	0.463	NA
Filifactor	2.916	0.659	0.988	NA
Geodermatophilaceae_genus	1.091	0.660	1.547	NA
Hemiselmis	0.919	0.660	1.731	NA
Aeribacillus	0.369	0.662	2.911	NA
Sordaria	1.077	0.667	1.189	NA
Erysipelothrix	0.043	0.669	2.912	NA
Curtobacterium	149.150	0.669	0.361	3.416e-01
Acidisoma	1.255	0.670	1.477	NA
Butyricicoccus	0.662	0.676	1.635	NA
Rathayibacter	27.104	0.678	0.635	6.852e-01
Belnapia	18.847	0.682	0.612	6.715e-01
Sorangium	0.948	0.683	2.191	NA
Halochromatium	4.224	0.684	0.638	NA
Roseococcus	1.779	0.686	1.349	NA
Hyphobacterium	32.142	0.686	0.295	1.661e-01
Undibacterium	178.266	0.688	0.331	2.494e-01
Tardiphaga	3.851	0.688	0.740	NA
Microbacter	0.671	0.695	1.702	NA
Mariprofundus	0.047	0.696	2.912	NA
Tepidicella	11.802	0.700	0.730	7.368e-01
Berkiella	0.057	0.700	2.912	NA
Meyerozyma	1.589	0.701	1.808	NA
Peptoniphilus	199.711	0.702	0.520	5.686e-01
Panacagrimonas	3.396	0.704	1.411	NA
Salegentibacter	0.151	0.706	2.910	NA
Limobrevibacterium	0.486	0.710	1.742	NA
Pseudoglutamicibacter	1.363	0.711	1.328	NA
Nisaea	0.072	0.722	2.912	NA
Aureispira	3.069	0.725	0.764	NA
Mobiluncus	3.137	0.725	1.034	NA
Aquabacter	1.491	0.725	1.211	NA
Enteractinococcus	1.267	0.726	2.201	NA
Alterileibacterium	0.250	0.727	2.910	NA
Teredinibacter	0.038	0.728	2.913	NA
Allofustis	0.129	0.728	2.911	NA
Parapusillimonas	0.200	0.729	2.911	NA
Chloroflexus	0.443	0.729	2.435	NA
Lawsonella	184.540	0.731	0.389	3.285e-01
Gallaecimonas	1.280	0.731	0.941	NA
Propionispora	0.201	0.731	2.909	NA
Starkeya	0.752	0.732	1.222	NA
Paraprevotella	2.071	0.734	1.136	NA

Qaidamihabitans	5.033	0.734	1.111	NA
Mediterraneibacter	9.994	0.738	0.422	3.820e-01
Motilibacter	2.373	0.738	1.451	NA
Fibrisoma	20.446	0.745	0.524	5.388e-01
Asanoa	0.412	0.747	2.907	NA
Pajaroellobacter	0.393	0.747	2.720	NA
Chelatococcus	4.743	0.752	0.909	NA
Rhodocyclus	0.572	0.752	1.518	NA
Dickeya	1.888	0.754	0.418	NA
Pyxidicoccus	1.671	0.755	1.132	NA
Amniculibacterium	0.863	0.755	2.885	NA
Streptoalloteichus	1.111	0.760	1.300	NA
Propionibacterium_phage_P100D_virus	0.085	0.764	2.912	NA
Purpureocillium	1.627	0.766	2.264	NA
Levyella	7.688	0.766	1.127	8.469e-01
Nitrosocosmicus	2.149	0.768	1.739	NA
Lederbergia	0.161	0.770	2.805	NA
Aridibaculum	0.190	0.773	2.912	NA
Methylocella	3.329	0.774	1.268	NA
Desulfoscipio	0.565	0.786	2.174	NA
Mariluticola	0.153	0.786	2.911	NA
Lawsonibacter	0.311	0.791	2.236	NA
Pannonibacter	2.009	0.793	1.350	NA
Rhodospirillum	1.216	0.795	1.343	NA
Propionibacterium_phage_PAD20_virus	0.255	0.796	2.910	NA
Extensimonas	3.609	0.797	1.008	NA
Tetragenococcus	7.371	0.797	0.837	7.416e-01
Frigoriflavimonas	0.201	0.797	2.275	NA
Neptunicoccus	0.118	0.797	2.911	NA
Crenalkalicoccus	0.619	0.798	1.981	NA
Lewinella	0.693	0.800	0.582	NA
Nanogingivalis	0.227	0.800	1.918	NA
Neurospora	0.742	0.802	2.185	NA
Arcticiflavibacter	197.563	0.809	0.316	1.068e-01
Parvimonas	4.539	0.811	0.795	NA
Frischella	1.199	0.812	0.898	NA
Clavibacter	4.588	0.812	0.911	NA
Sporomusaceae_genus	0.444	0.813	1.285	NA
Hominisplanchenecus	0.033	0.816	2.913	NA
Acytostelium	1.048	0.817	0.857	NA
Andreesenia	0.720	0.819	2.561	NA
Enterobacteria_phage_vB_EcoS_IME542_virus	2.043	0.821	0.992	NA
Micromonospora	92.552	0.822	0.426	3.068e-01
Caldibacillus	3.835	0.826	0.811	NA
Catenibacterium	0.871	0.826	1.927	NA
Parapedobacter	0.475	0.830	1.355	NA
Knoellia	9.989	0.838	0.688	6.358e-01
Minimicrobia	2.704	0.838	0.971	NA
Vibrionimonas	0.863	0.840	1.853	NA
Calothrix	3.097	0.840	0.924	NA
Mogibacterium	9.735	0.843	0.622	5.667e-01
Simian_virus_40	1.059	0.844	0.770	NA
Gammaproteobacteria	2.722	0.844	0.976	NA
Pseudogymnoascus	4.453	0.849	0.936	NA
Abyssicoccus	5.009	0.850	1.137	NA
Tahibacter	0.454	0.852	1.547	NA
Sinomonas	0.052	0.855	2.913	NA
Truncatella	0.941	0.859	1.466	NA
Paenarthrobacter	0.392	0.860	2.384	NA

Saccharimonas	0.553	0.863	1.947	NA
Neglectibacter	0.247	0.866	1.373	NA
Sutterella	3.974	0.868	0.603	NA
Desulfofundulus	0.440	0.869	2.908	NA
Moheibacter	0.373	0.873	2.632	NA
Dysgonomonas	10.780	0.876	0.434	2.702e-01
Chondromyces	1.551	0.877	1.653	NA
Halalkalibacterium	3.247	0.878	0.865	NA
Herbiconiux	5.773	0.882	0.980	7.697e-01
Grimontia	0.335	0.894	0.758	NA
Tsuneonella	2.101	0.897	1.391	NA
Cryphonectria	1.076	0.900	1.329	NA
Massilia	397.055	0.906	0.274	2.093e-02
Herbinix	4.473	0.907	1.377	NA
Occultella	0.037	0.908	2.913	NA
Pseudobdellovibrio	0.295	0.912	2.268	NA
Nanoperiomorbus	1.710	0.924	1.345	NA
Agrococcus	150.918	0.926	0.642	5.388e-01
Daldinia	6.504	0.930	1.046	7.697e-01
Pectobacterium	2.621	0.933	0.525	NA
Marinomonas	8.179	0.933	0.927	7.156e-01
Pseudocercospora	7.879	0.936	0.628	5.087e-01
Chloroflexi	0.627	0.940	1.421	NA
Type-B_symbiont_of_Plautia_stali	0.070	0.947	2.374	NA
Pseudenterobacter	0.515	0.947	0.535	NA
Lujinxingia	0.179	0.948	1.921	NA
Fomitiporia	9.122	0.948	0.785	6.378e-01
Intrasporangiaceae_genus	0.940	0.952	1.257	NA
Natronorubrum	1.146	0.954	1.406	NA
Granulicoccus	0.329	0.957	2.658	NA
Flavihumibacter	2.570	0.958	0.824	NA
Xanthocytophaga	2.896	0.959	1.142	NA
Nevskia	5.606	0.960	1.059	7.674e-01
Quatrionicoccus	0.245	0.962	2.909	NA
Calorimonas	0.022	0.962	2.913	NA
Methylovorus	2.833	0.963	0.830	NA
Nitratireductor	1.522	0.970	0.963	NA
Type-E_symbiont_of_Plautia_stali	0.036	0.975	2.913	NA
Thermopolyspora	0.034	0.975	2.913	NA
Galbitalea	2.897	0.976	1.201	NA
Aliidiomarina	6.184	0.977	0.575	4.192e-01
Botrytis	2.262	0.977	1.080	NA
Robinsoniella	0.031	0.984	2.913	NA
Thermaurantiacus	0.224	0.987	2.910	NA
Oscillochloris	0.443	0.989	2.907	NA
Caldilinea	1.616	0.991	1.735	NA
Thermaerobacter	0.441	0.991	2.394	NA
Hypoxylon	1.453	0.992	1.441	NA
Aceticella	0.332	1.000	2.909	NA
Zygosaccharomyces	0.127	1.001	2.912	NA
Roseitranquillus	0.614	1.002	2.111	NA
Brasilonema	1.390	1.003	1.488	NA
Thermalbibacter	6.902	1.006	1.000	7.156e-01
Marasmius	3.117	1.017	0.981	NA
Anaerotruncus	0.786	1.021	0.867	NA
Nannizzia	1.520	1.040	1.257	NA
Haloferax	0.284	1.041	2.711	NA
Streptacidiphilus	1.421	1.044	1.478	NA
Parageobacillus	1.709	1.047	1.399	NA

Plesiocystis	0.041	1.053	2.913	NA
Thermodesulfomicrobium	0.020	1.059	2.913	NA
Pengzhenrongella	0.868	1.059	1.528	NA
Patulibacter	9.106	1.061	0.737	5.388e-01
Aliidongia	0.759	1.064	1.591	NA
Pseudorivibacter	1.087	1.065	1.402	NA
Mycobacteroides	34.731	1.065	0.639	4.398e-01
Enterocloster	0.679	1.073	1.796	NA
Inhella	1.220	1.076	1.575	NA
Rickettsiales	0.188	1.079	2.516	NA
Cryobacterium	1.015	1.082	1.789	NA
Porphyromonas	55.787	1.087	0.350	3.332e-02
Paenisporosarcina	0.629	1.101	2.911	NA
Granulicella	0.707	1.102	2.147	NA
Marisediminicola	0.599	1.104	2.346	NA
Methanotrophic	6.831	1.106	0.778	5.388e-01
Gryllotalpicola	6.324	1.109	0.994	6.715e-01
Betaproteobacteria	0.088	1.109	2.912	NA
Pseudarthrobacter	55.707	1.114	0.446	1.295e-01
Metabacillus	4.195	1.116	1.057	NA
Paenimyroides	6.925	1.116	0.955	6.513e-01
Alloiococcus	1.164	1.125	1.275	NA
Cucumibacter	0.022	1.128	2.913	NA
Petrimonas	0.089	1.128	2.913	NA
Rhizobiaceae_genus	0.018	1.128	2.913	NA
Sulfuritalea	0.033	1.128	2.913	NA
Mangrovibacillus	2.743	1.132	1.195	NA
Anaerobutyricum	0.119	1.135	2.911	NA
Macromonas	0.175	1.140	2.910	NA
Annandia	0.084	1.144	2.073	NA
Microthrix	0.483	1.147	2.907	NA
Macellibacteroides	0.431	1.150	2.135	NA
Komagataeibacter	1.071	1.153	1.494	NA
Limnobacter	140.336	1.157	0.577	2.810e-01
Collimonas	9.925	1.159	0.701	4.397e-01
Syntrophomonas	0.225	1.160	2.909	NA
Umbelopsis	0.027	1.161	2.913	NA
Riesia	0.026	1.164	2.913	NA
Drepanopeziza	2.702	1.168	1.066	NA
Catellicoccus	0.496	1.174	2.192	NA
Urbifossiella	0.741	1.174	2.158	NA
Hansschlegelia	1.485	1.175	1.426	NA
Sclerotinia	1.417	1.176	1.259	NA
Cellvibrio	6.798	1.179	0.718	4.483e-01
Planobispora	0.083	1.186	2.912	NA
Propionicimonas	2.047	1.186	1.047	NA
Chryseolinea	0.170	1.194	2.912	NA
Bacteria	0.118	1.194	0.927	NA
Arboricoccus	0.363	1.194	2.359	NA
Enterobacteria_phage_vB_EcoS_ACG-M12_virus	1.320	1.196	1.126	NA
Propylenella	0.014	1.199	2.913	NA
Rivularia	0.796	1.199	1.852	NA
Planifilum	0.365	1.203	2.912	NA
Petrotoga	0.426	1.209	2.144	NA
Alishewanella	18.469	1.211	0.658	3.507e-01
Heterobasidion	24.973	1.214	0.851	5.388e-01
Tuwongella	0.369	1.225	2.909	NA
Paramesorhizobium	0.152	1.232	2.912	NA
Luteococcus	0.817	1.234	1.972	NA

Tepidiphilus	66.163	1.234	0.599	2.650e-01
Anaerococcus	995.459	1.235	0.549	2.016e-01
Finegoldia	126.567	1.236	0.558	2.102e-01
Fontimonas	0.265	1.236	2.787	NA
Ureibacillus	0.798	1.238	1.446	NA
Actinomarinicola	1.651	1.242	1.262	NA
Amnimonas	7.614	1.244	1.076	6.618e-01
Filobasidium	18.977	1.247	0.503	1.295e-01
Kickxella	0.434	1.253	1.986	NA
Puniceibacterium	0.603	1.256	1.674	NA
Ehrlichia	2.744	1.262	1.077	NA
Lancefieldella	4.241	1.264	1.230	NA
Oceanibium	5.401	1.264	0.639	2.790e-01
Methanothrix	0.009	1.267	2.913	NA
Tachikawaea	0.009	1.267	2.913	NA
Ruoffia	0.404	1.269	1.951	NA
Zhihengliuella	2.204	1.273	1.432	NA
Neofamilia	0.005	1.275	2.913	NA
Protaetiibacter	2.315	1.279	1.160	NA
Escherichia_phage_vB_EcoS_ESCO41_virus	1.030	1.282	1.384	NA
Faecalicatena	0.820	1.282	1.499	NA
Viridibacillus	0.463	1.289	1.978	NA
Polynucleobacter	2.817	1.292	0.908	NA
Chlorogloea	2.792	1.293	1.313	NA
Planococcaceae_genus	0.288	1.293	2.909	NA
Dactylellina	1.676	1.295	1.378	NA
Microbacteriaceae_genus	3.298	1.303	1.166	NA
Pseudaestuariivita	0.064	1.305	2.912	NA
Mesobacillus	1.096	1.307	1.358	NA
Cloacibacterium	93.517	1.307	0.439	4.751e-02
Thermicanus	22.909	1.310	0.914	5.388e-01
Arachidicoccus	0.202	1.314	2.911	NA
Phaeoacremonium	2.333	1.314	0.659	NA
Pseudidiomarina	0.129	1.316	1.832	NA
Allosphingosinicella	0.640	1.316	1.592	NA
Gemmatirosa	1.497	1.327	1.672	NA
SsRNA_phage_SRR5466369_2_virus	0.058	1.331	2.913	NA
Agathobaculum	0.011	1.332	2.913	NA
Betaproteobacterium_AAP99	0.003	1.333	2.913	NA
Doolittlea	0.005	1.333	2.913	NA
Nioella	0.006	1.333	2.913	NA
Enterobacteria_phage_f1_virus	0.008	1.333	2.913	NA
Zafaria	0.027	1.333	2.913	NA
Fibroporia	6.292	1.336	1.078	6.358e-01
Buttiauxella	1.130	1.340	0.969	NA
Enterobacteria_phage_T7_virus	0.002	1.344	2.913	NA
Oryzihumus	0.030	1.345	2.913	NA
Pelovirga	0.002	1.356	2.913	NA
Fusicatenibacter	1.772	1.359	1.362	NA
Alkalibacterium	1.404	1.361	1.575	NA
Desulfuromonas	1.691	1.367	1.462	NA
Pseudodesulfovibrio	0.057	1.384	2.913	NA
Anaeroglobus	4.318	1.387	0.750	NA
Cronobacter_phage_vB_CsaM_GAP32_virus	0.325	1.400	2.686	NA
Amnibacterium	9.282	1.400	0.779	3.750e-01
Mycoplana	0.055	1.408	2.913	NA
Endozoicomonas	0.368	1.410	1.263	NA
Desulforhabdus	2.687	1.416	1.054	NA
Provencibacterium	0.082	1.422	2.912	NA

Paeniroseomonas	0.513	1.424	1.721	NA
Plectonema	0.287	1.425	2.911	NA
Agaricicola	0.028	1.426	2.913	NA
Niabella	0.539	1.429	1.785	NA
Grosmannia	0.859	1.432	1.527	NA
Paraflavisolibacter	0.061	1.437	2.913	NA
Cnuella	0.966	1.439	1.736	NA
Phialemonium	0.746	1.442	1.994	NA
Oerskovia	0.479	1.447	2.565	NA
Pseudoflavonifractor	1.639	1.450	0.978	NA
Buchananella	0.056	1.451	2.913	NA
Punctularia	12.219	1.452	0.882	4.490e-01
Chlamydia	0.283	1.453	2.804	NA
Rehaibacterium	0.697	1.454	1.973	NA
Pseudocnuella	0.005	1.464	2.913	NA
Adhaeribacter	6.285	1.472	1.122	6.026e-01
Gemmata	0.949	1.473	1.532	NA
Idiomarinaceae_genus	0.023	1.481	2.913	NA
Falcatimonas	0.017	1.481	2.913	NA
Photodesmus	0.007	1.485	2.913	NA
Lactovum	3.125	1.485	1.040	NA
Muribaculaceae_genus	0.814	1.490	1.314	NA
Enterobacteria_phage_YYZ-2008_virus	0.009	1.491	2.913	NA
Hydromonas	0.010	1.491	2.913	NA
Thermosinus	0.296	1.492	2.909	NA
Wielerella	1.083	1.496	1.796	NA
Psilocybe	1.438	1.496	0.847	NA
Pararobbsia	0.017	1.498	2.913	NA
Usitatibacter	0.008	1.501	2.913	NA
Erysipelotrichaceae_genus	0.086	1.511	2.912	NA
Bathymodiolus	0.021	1.511	2.913	NA
Simonsiella	0.622	1.521	2.129	NA
Segnochrobactrum	1.097	1.525	2.009	NA
Evansella	0.719	1.525	2.215	NA
Emticicia	2.070	1.526	1.459	NA
Centipeda	0.032	1.529	2.913	NA
Miniphocaeibacter	0.034	1.530	2.913	NA
Eutypa	3.140	1.531	0.826	NA
Pseudescherichia	0.062	1.532	2.912	NA
Micavibrio	0.558	1.534	1.988	NA
Naasia	4.140	1.541	1.070	NA
Flavonifractor	0.146	1.544	2.912	NA
Terribacillus	0.063	1.544	2.913	NA
Variibacter	0.081	1.547	2.912	NA
Microvirgula	1.559	1.550	1.663	NA
Siphonobacter	1.123	1.562	2.100	NA
Pectobacterium_phage_CBB_virus	1.674	1.565	1.366	NA
Microvirga	140.126	1.571	0.378	2.032e-03
Sabulicella	0.024	1.582	2.913	NA
Formosimonas	0.082	1.584	2.913	NA
Hassallia	0.102	1.584	2.913	NA
Endobacter	3.493	1.592	1.154	NA
Verrucomicrobia	1.240	1.594	1.728	NA
Beutenbergia	4.200	1.596	1.087	NA
Calidifontibacter	0.577	1.601	2.133	NA
Coriobacteriales	0.250	1.603	2.910	NA
Caldifermentibacillus	8.825	1.609	1.076	5.249e-01
Geoalkalibacter	0.121	1.611	2.913	NA
Verticiella	0.592	1.629	2.364	NA

Haloechinothrix	0.147	1.631	2.737	NA
Drancourtella	0.234	1.636	2.911	NA
Tenebrionicola	0.031	1.638	2.913	NA
Cadophora	1.997	1.639	1.097	NA
Thermoleophilum	0.347	1.642	2.673	NA
Flavipsychrobacter	0.537	1.655	2.379	NA
Alkalihalophilus	0.036	1.656	2.913	NA
Aplosporella	8.752	1.667	0.747	2.102e-01
Vitreoscilla	1.803	1.667	1.449	NA
Gulbenkiania	0.803	1.668	1.737	NA
Superficieibacter	0.163	1.674	1.121	NA
Podospora	0.781	1.677	0.751	NA
Glycomyces	0.654	1.677	1.959	NA
Myroides	1.084	1.679	1.844	NA
Kordiimonas	0.368	1.682	2.323	NA
Stomatobaculum	7.662	1.685	0.758	2.102e-01
Propionibacterium_phage_SKKY_virus	0.362	1.689	2.909	NA
Cellulosimicrobium	4.141	1.691	1.241	NA
Fervidobacterium	0.190	1.692	2.910	NA
Profftia	0.070	1.695	2.912	NA
Hyaloscypha	3.026	1.702	0.842	NA
Parachlamydia	0.212	1.709	2.910	NA
Actinoallomurus	1.044	1.722	1.595	NA
Gilliamella	0.563	1.731	1.151	NA
Aff.	0.263	1.739	2.910	NA
Neoactinobaculum	0.622	1.740	2.131	NA
Coriobacteriaceae_genus	0.483	1.743	2.387	NA
Falseniella	0.389	1.748	2.339	NA
Roseibaca	0.208	1.760	2.515	NA
Limnobaculum	0.794	1.763	1.069	NA
Paenalcaligenes	0.760	1.763	0.902	NA
Trichoderma	47.821	1.768	0.653	9.398e-02
Proteiniphilum	0.111	1.771	2.912	NA
Nitrososphaera	1.719	1.781	2.017	NA
Tomitella	1.442	1.783	1.325	NA
Anoxybacillus	29.382	1.784	0.616	6.320e-02
Moniliophthora	4.340	1.788	0.875	NA
Rubricoccus	0.152	1.803	2.912	NA
Picosynechococcus	10.813	1.808	0.687	1.068e-01
Papillibacter	0.041	1.817	2.913	NA
Argonema	0.523	1.821	2.375	NA
Plantibacter	4.413	1.832	0.922	NA
Tsukamurella	5.879	1.834	1.257	5.388e-01
Faunimonas	0.206	1.836	2.760	NA
Aestuariivirga	0.693	1.846	2.151	NA
Acidiphilium	0.973	1.851	1.428	NA
Ramularia	0.158	1.868	2.913	NA
Methyloradius	0.198	1.880	2.912	NA
Saccharomyces	1.469	1.885	1.556	NA
Halovulum	0.908	1.893	1.606	NA
Pelobacter	1.522	1.911	1.571	NA
Wickerhamomyces	2.172	1.912	1.531	NA
Oleiphilus	2.361	1.931	0.732	NA
Acetivibrio	0.767	1.935	2.085	NA
Methylocaldum	0.238	1.942	1.730	NA
Solirhodobacter	0.058	1.944	2.912	NA
Mammaliicoccus	12.984	1.951	0.702	8.419e-02
Stereum	19.599	1.969	0.746	1.068e-01
Labedella	0.708	1.970	1.975	NA

Protochlamydia	0.307	1.993	2.909	NA
Propionibacterium_phage_PHL301M00_virus	0.111	1.997	2.912	NA
Parasegetibacter	0.335	2.005	2.910	NA
Arenivirga	0.339	2.010	2.910	NA
Nannochloropsis	1.721	2.016	1.244	NA
Aquirhabdus	0.124	2.019	2.912	NA
Advenella	1.895	2.022	0.937	NA
Baumannia	0.277	2.027	2.912	NA
Chloroflexales	0.074	2.051	2.912	NA
Thermacetogenium	0.690	2.058	2.675	NA
Pseudaminobacter	1.415	2.064	1.493	NA
Fuscibacter	0.854	2.068	2.074	NA
Durotheca	0.146	2.080	2.911	NA
Atopococcus	0.111	2.118	2.912	NA
Pirellulimonas	0.144	2.141	2.911	NA
Desertibacillus	1.178	2.169	1.518	NA
Plasticicumulans	0.111	2.172	2.912	NA
Shouchella	0.581	2.178	2.684	NA
Acidimicrobium	0.273	2.179	2.911	NA
Dialister	5.744	2.180	0.907	1.726e-01
Frondihabitans	4.024	2.183	1.264	NA
Flaviaesturariibacter	0.706	2.229	2.252	NA
Scardovia	0.474	2.240	2.908	NA
Jeotgalibaca	0.938	2.262	1.820	NA
Mollisia	7.237	2.274	0.796	7.367e-02
Paracaedibacter	1.572	2.275	1.556	NA
Faecalimonas	1.081	2.310	1.603	NA
Subtercola	0.944	2.314	1.823	NA
Rhodocyclales	0.737	2.362	2.276	NA
Fontibacillus	0.151	2.366	2.911	NA
Serpula	11.118	2.372	1.146	2.827e-01
Methyloglobulus	0.550	2.373	1.455	NA
Hubei_permutotetra-like_virus	2.387	2.377	1.642	NA
Roseicitreum	1.865	2.378	1.176	NA
Corticibacterium	0.584	2.378	1.975	NA
Arthromitus	0.148	2.379	2.912	NA
Microterricola	0.560	2.381	2.139	NA
Moranella	0.333	2.383	2.499	NA
Salinispora	1.822	2.402	1.343	NA
Halteromyces	0.737	2.403	1.510	NA
Desnuesiella	0.296	2.406	2.911	NA
Stenoxybacter	0.289	2.480	2.911	NA
Parvibaculum	0.863	2.483	1.310	NA
Fastidiosipila	0.389	2.492	2.911	NA
Laspinema	0.879	2.503	1.789	NA
Chloroflexia	0.169	2.520	2.911	NA
Nigerium	0.539	2.525	2.301	NA
Thiofilum	1.021	2.540	1.916	NA
Allostreptomyces	0.218	2.548	2.911	NA
Betaproteobacterium_AAP121	0.906	2.550	1.004	NA
Sulfuriferula	0.273	2.568	2.910	NA
Yoonia	0.398	2.587	2.576	NA
Heyndrickxia	3.581	2.607	1.327	NA
Chloroploca	0.537	2.652	2.908	NA
Rhodoblastus	1.061	2.686	1.498	NA
Veillonellaceae_genus	0.639	2.692	2.295	NA
Betaproteobacterium_AAP65	0.934	2.706	1.031	NA
Parachlamydiaceae_genus	0.261	2.708	2.911	NA
Falsochrobactrum	0.395	2.790	2.849	NA

Hanamia	0.253	2.802	2.911	NA
Globicatella	1.145	2.816	1.807	NA
Auritidibacter	1.274	2.828	2.728	NA
Pseudosporangium	0.338	2.891	2.911	NA
Maridesulfovibrio	0.477	2.929	2.909	NA
Neoarthrinium	0.839	2.938	1.371	NA
Coprothermobacter	0.362	2.946	2.910	NA
Apibacter	8.111	2.969	0.986	6.810e-02
Debaryomyces	1.396	2.979	1.687	NA
Uruburuella	0.311	2.981	2.910	NA
Haematomicrobium	1.237	3.051	2.092	NA
Schneideria	1.783	3.071	1.374	NA
Acidiluteibacter	0.402	3.087	2.910	NA
Thermincola	0.633	3.147	2.249	NA
Propionibacterium_phage_PHL116M00_virus	0.473	3.374	2.484	NA
Polysphondylium	0.588	3.379	2.230	NA
Bergeriella	1.836	3.530	1.464	NA
Planomicrobium	3.609	3.550	1.388	NA
Laetiporus	0.632	3.645	2.909	NA
Gellertiella	2.769	3.779	1.432	NA
Ethanoligenens	2.089	3.800	1.808	NA
Thiothrix	0.796	3.819	2.423	NA
