# Supplementary material for: Shotgun Metagenome Analysis of Two Schizaphis graminum Biotypes over Time With and Without Carried Cereal Yellow Dwarf Virus
Source: Insects. 2025 May 23;16(6):554. doi: 10.3390/insects16060554 (PMC12193481; doi:10.3390/insects16060554)
Supplement: Supplementary file 1 [file insects-16-00554-s001.zip › Table S6.pdf]

Table S6. DESeq2 results for comparison by viral carrier status, arranged by BH-adjusted p-value.

Genus	BaseMean	Log2FC	LFCSE	Padj
Blochmannia	14.483	4.997	0.563	1.120e-12
Izhakiella	17.352	5.941	0.684	1.828e-11
Microvirga	228.961	-2.462	0.419	5.661e-06
Salipaludibacillus	74.463	-2.609	0.479	4.200e-05
Chryseobacterium	293.101	0.911	0.187	2.065e-04
Letharia	101.125	-2.569	0.535	6.544e-04
Meira	37.666	-4.850	0.907	7.304e-04
Moesziomyces	19.389	-3.826	0.814	1.558e-03
Aequitasia	23.263	-3.976	0.853	3.809e-03
Macroventuria	17.481	-1.762	0.475	1.885e-02
Labrys	96.739	1.329	0.366	2.498e-02
Ascochyta	68.305	-1.694	0.491	4.130e-02
Mycotypha	24.187	4.330	1.166	4.130e-02
Aliidiomarina	6.184	1.958	0.554	4.130e-02
Vibrio	744.207	0.813	0.252	6.241e-02
Mitsuaria	151.580	-0.982	0.304	6.241e-02
Azotobacter	95.528	-1.390	0.426	6.241e-02
Chitinophaga	6.927	-1.734	0.569	9.645e-02
Pseudoalteromonas	365.269	-0.594	0.197	9.818e-02
Pedobacter	122.644	-0.784	0.258	9.818e-02
Campylobacter	39.556	0.840	0.286	1.132e-01
Agarivorans	92.107	1.718	0.567	1.132e-01
Xanthobacter	8.431	-1.937	0.660	1.132e-01
Anaerococcus	995.459	1.605	0.543	1.231e-01
Glutamicibacter	45.516	-1.265	0.439	1.284e-01
Limnobacter	116.231	1.599	0.553	1.408e-01
Dorea	18.063	-1.625	0.573	1.415e-01
Spiribacter	38.734	1.161	0.409	1.431e-01
Streptomyces	1840.873	-0.323	0.120	1.766e-01
Spizellomyces	7.358	-2.710	0.994	1.766e-01
Staphylococcus	1969.163	0.745	0.277	1.831e-01
Alishewanella	18.469	1.735	0.647	2.194e-01
Ochrobactrum	77.175	0.890	0.343	2.287e-01
Gloeophyllum	13.463	-2.460	0.939	2.329e-01
Paucibacter	116.341	-0.576	0.227	2.454e-01
Robertmurraya	10.088	-1.531	0.609	2.514e-01
Shewanella	593.636	0.353	0.142	2.542e-01
Loigolactobacillus	21.543	-2.514	0.964	2.542e-01
Edwardsiella	64.525	-0.797	0.321	2.569e-01
Bacillus	2979.064	-0.282	0.116	2.666e-01
Microbacterium	1220.119	-0.316	0.134	2.666e-01
Janibacter	177.517	0.556	0.229	2.666e-01
Undibacterium	178.266	0.784	0.329	2.666e-01
Proteus	179.703	-0.831	0.341	2.666e-01
Ornithinimicrobium	139.539	1.059	0.446	2.666e-01
Polychytrium	14.681	-1.777	0.743	2.666e-01
Exiguobacterium	51.512	0.803	0.332	2.666e-01
Acidihalobacter	28.945	1.234	0.507	2.666e-01
Aureobasidium	16.662	-1.507	0.604	2.666e-01
Orbilia	13.707	2.094	0.843	2.666e-01
Methylovulum	39.553	-1.126	0.463	2.666e-01
Methylomicrobium	16.552	-1.323	0.551	2.666e-01
Mollisia	7.237	-1.914	0.815	2.666e-01
Propionimicrobium	87.295	1.159	0.491	2.696e-01
Westeberhardia	22.020	0.941	0.406	2.696e-01

Glarea	9.663	-1.734	0.746	2.696e-01
Propioniciclava	26.542	-1.278	0.555	2.866e-01
Dictyostelium	8.462	-1.816	0.778	2.866e-01
Legionella	1332.136	0.531	0.233	2.947e-01
Reyranella	10.238	1.247	0.554	3.139e-01
Pandoraea	33.582	0.689	0.309	3.204e-01
Dietzia	166.910	0.715	0.323	3.329e-01
Peptoniphilus	199.711	-1.137	0.514	3.348e-01
Coraliihabitans	13.613	-1.432	0.650	3.348e-01
Leptosphaeria	39.541	1.644	0.738	3.348e-01
Dyella	23.540	-0.745	0.342	3.348e-01
Kinneretia	11.622	-1.084	0.495	3.348e-01
Salinimicrobium	20.796	0.991	0.455	3.445e-01
Actinomadura	901.640	-0.314	0.148	3.475e-01
Lawsonella	225.924	0.880	0.410	3.475e-01
Gleimia	229.791	-0.830	0.388	3.475e-01
Macrococcus	6.515	1.667	0.770	3.475e-01
Tumebacillus	31.849	-0.799	0.378	3.486e-01
Carnobacterium	19.889	1.002	0.473	3.486e-01
Rhodopirellula	20.297	0.866	0.413	3.645e-01
Veillonella	194.159	0.684	0.328	3.735e-01
Propionibacterium	5198.439	0.570	0.279	3.892e-01
Musicola	448.106	-0.719	0.349	3.892e-01
Gemella	167.060	0.838	0.409	3.892e-01
Rahnella	196.146	-0.598	0.295	3.892e-01
Betaproteobacterium_JGI	8.316	0.847	0.413	3.892e-01
Tamlana	6.081	-0.893	0.443	3.892e-01
Alkalispirochaeta	9160.805	0.470	0.234	4.052e-01
Myceligenerans	6.325	1.130	0.566	4.067e-01
Turicibacter	9.170	-2.544	1.181	4.106e-01
Skermanella	18.493	1.066	0.528	4.106e-01
Geobacillus	19.526	1.144	0.569	4.106e-01
Trypanosoma	18.124	1.153	0.577	4.106e-01
Okeania	8.897	-1.496	0.764	4.106e-01
Providencia	883.343	-0.303	0.156	4.135e-01
Mycobacterium	323.882	0.358	0.185	4.135e-01
Cryptococcus	7.850	1.581	0.785	4.135e-01
Singulisphaera	6.587	1.709	0.862	4.135e-01
Bacterium	36.502	0.451	0.233	4.135e-01
Hyphobacterium	32.142	0.576	0.297	4.135e-01
Xanthomonas	75.563	0.556	0.289	4.196e-01
Mycolicibacterium	115.168	0.613	0.316	4.196e-01
Winogradskyella	5.235	0.690	0.377	4.196e-01
Ilumatobacter	13.317	-2.075	1.095	4.208e-01
Winkia	10.745	-1.444	0.757	4.208e-01
Serratia	233.668	-0.457	0.246	4.223e-01
Aequorivita	187.348	-0.354	0.190	4.223e-01
Craterilacuibacter	127.435	-0.905	0.484	4.223e-01
Photorhabdus	47.052	0.685	0.363	4.223e-01
Acidipropionibacterium	24.957	1.092	0.582	4.223e-01
Ruminococcus	28.348	0.899	0.476	4.223e-01
Alloprevotella	42.819	-0.891	0.469	4.223e-01
Exophiala	38.352	1.133	0.593	4.223e-01
Filobasidium	18.977	-0.973	0.512	4.223e-01
Lutimaribacter	14.713	-1.020	0.545	4.223e-01
Loktanella	12.504	-0.478	0.258	4.223e-01
Thalassiosira	9.266	1.065	0.563	4.223e-01
Planktothrix	6.905	1.165	0.614	4.223e-01
Haemophilus	217.469	-0.499	0.270	4.239e-01

Erwinia	905.375	-0.543	0.294	4.265e-01
Agilicoccus	80.850	-0.954	0.519	4.376e-01
Rhodococcus	464.902	-0.327	0.180	4.421e-01
Frigidibacter	67.900	0.507	0.278	4.421e-01
Enterococcus	585.175	0.234	0.132	4.426e-01
Tepidiphilus	66.163	-1.074	0.603	4.426e-01
Morococcus	313.297	-0.474	0.270	4.426e-01
Roseateles	324.440	-0.309	0.171	4.426e-01
Hymenobacter	123.295	-0.572	0.325	4.426e-01
Entamoeba	45.695	-0.912	0.507	4.426e-01
Janthinobacterium	149.394	0.427	0.241	4.426e-01
Devosia	74.074	-0.593	0.340	4.426e-01
Batrachochytrium	21.736	-3.687	1.846	4.426e-01
Lobosporangium	11.067	-1.935	1.107	4.426e-01
Bowmanella	57.320	0.566	0.324	4.426e-01
Mixta	7.632	0.935	0.530	4.426e-01
Hydrogenophaga	66.646	0.667	0.376	4.426e-01
Oribacterium	16.999	-1.018	0.581	4.426e-01
Caldifermentibacillus	6.086	-1.822	1.051	4.426e-01
Alteromonas	44.223	0.867	0.484	4.426e-01
Roseburia	27.841	-0.460	0.256	4.426e-01
Cutaneotrichosporon	17.445	1.688	0.913	4.426e-01
Agromyces	13.851	0.733	0.410	4.426e-01
Xylophilus	6.954	-0.847	0.503	4.426e-01
Methanotrophic	6.831	1.367	0.776	4.426e-01
Phanerochaete	8.638	-1.310	0.773	4.502e-01
Pimelobacter	6.825	-1.201	0.698	4.502e-01
Glaciecola	6.760	0.612	0.355	4.502e-01
Besnoitia	7.844	-0.709	0.426	4.625e-01
Sphingomonas	2004.426	-0.216	0.128	4.749e-01
Fimicolochytrium	24.210	-1.904	1.097	4.749e-01
Xenophilus	37.016	-0.538	0.323	4.852e-01
Serpula	8.078	1.968	1.151	4.930e-01
Rhizorhabdus	68.045	-0.481	0.291	4.930e-01
Trichoderma	47.821	-1.115	0.667	4.930e-01
Qaidamihabitans	5.033	1.837	1.086	4.930e-01
Nitriliruptoraceae_genus	81.801	1.448	0.863	4.966e-01
Kitasatospora	20.269	0.901	0.552	5.046e-01
Lichtheimia	16.959	-0.603	0.368	5.046e-01
Paraclostridium	10.370	1.114	0.684	5.162e-01
Virgibacillus	32.734	0.389	0.246	5.162e-01
Nakamurella	30.160	-0.841	0.521	5.162e-01
Microcoleus	16.615	0.885	0.544	5.162e-01
Planctomonas	5.568	1.454	0.896	5.323e-01
Wickerhamiella	11.493	-1.164	0.729	5.334e-01
Rhizobiales	18.512	-0.720	0.451	5.334e-01
Gluconobacter	9.302	0.995	0.629	5.334e-01
Moorena	7.922	-1.269	0.826	5.371e-01
Desulfovibrio	71087.628	0.346	0.223	5.413e-01
Gilbertella	13763.122	-0.359	0.231	5.413e-01
Azospirillum	271.311	0.526	0.338	5.413e-01
Nesterenkonia	69.289	0.417	0.270	5.413e-01
Methyloversatilis	103.042	-0.424	0.273	5.413e-01
Pseudooceanicola	63.502	-0.687	0.446	5.413e-01
Didymosphaeria	28.357	-0.904	0.586	5.413e-01
Talaromyces	10.629	1.071	0.688	5.413e-01
Limnohabitans	19.440	0.522	0.338	5.413e-01
Knoellia	9.989	-1.034	0.687	5.661e-01
Mammaliicoccus	12.984	1.113	0.725	5.661e-01

Paenibacillus	158.355	0.274	0.182	5.696e-01
Aurantimonas	8.757	-0.807	0.539	5.733e-01
Eleftheria	9.525	-0.929	0.618	5.763e-01
Collinsella	12.119	-1.263	0.820	5.764e-01
Corallococcus	123.324	-0.389	0.264	5.792e-01
Aureimonas	24.555	-0.744	0.506	5.792e-01
Marinithermofilum	29.006	-0.382	0.261	5.792e-01
Ectothiorhodospira	16.455	0.522	0.357	5.792e-01
Chitinimonas	12.648	1.000	0.675	5.792e-01
Solibacillus	6.613	0.942	0.639	5.792e-01
Colwellia	7.776	-0.747	0.511	5.792e-01
Brevibacterium	83.680	0.486	0.332	5.853e-01
Ramlibacter	60.516	-0.515	0.353	5.853e-01
Fibrisoma	25.538	-0.812	0.558	5.878e-01
Lachnospiraceae_genus	22.274	0.641	0.442	5.878e-01
Dysgonomonas	10.780	0.636	0.439	5.905e-01
Methylosarcina	17.576	-0.828	0.568	5.928e-01
Amaricoccus	20.347	-0.796	0.552	5.937e-01
Salinicoccus	15.586	0.910	0.632	6.014e-01
Rhodoferax	44.739	0.445	0.315	6.075e-01
Polyangium	64.134	-0.774	0.542	6.075e-01
Pseudogymnoascus	5.785	-1.321	0.978	6.075e-01
Bipolaris	7.199	-1.051	0.772	6.075e-01
Williamsia	113.724	0.620	0.444	6.187e-01
Apiotrichum	11.988	1.075	0.767	6.187e-01
Desulfosporosinus	5.604	-0.860	0.654	6.187e-01
Toxoplasma	250.863	-0.207	0.150	6.229e-01
Isoptericola	31.305	0.319	0.235	6.229e-01
Scytonema	22.749	0.655	0.478	6.229e-01
Ligilactobacillus	14.208	0.881	0.631	6.229e-01
Stomatobaculum	7.662	-1.047	0.779	6.229e-01
Thiohalocapsa	35.128	0.485	0.355	6.281e-01
Selenomonas	7.222	0.720	0.541	6.427e-01
Leptotrichia	40.411	0.441	0.329	6.499e-01
Parasaccharibacter	82.051	-0.283	0.213	6.544e-01
Pseudarthrobacter	55.707	0.605	0.455	6.605e-01
Treponema	11.715	0.670	0.506	6.605e-01
Pseudocercospora	7.879	-0.818	0.634	6.664e-01
Qipengyuania	68.427	-0.550	0.422	6.756e-01
Porphyromonas	64.376	-0.505	0.387	6.781e-01
Chromobacterium	7.322	-0.638	0.507	6.840e-01
Seonamhaeicola	12.679	0.523	0.415	6.840e-01
Nodosilinea	21.716	0.980	0.759	6.866e-01
Moraxellaceae_genus	14.760	-0.711	0.553	6.866e-01
Abiotrophia	14.388	0.773	0.604	6.866e-01
Pinisolibacter	11.572	-0.328	0.277	6.866e-01
Diaporthe	5.499	-1.525	1.064	6.866e-01
Alcanivorax	267.141	0.291	0.229	6.896e-01
Pseudomonas	20853.250	-0.262	0.207	6.899e-01
Pseudoramibacter	88.151	0.640	0.506	6.899e-01
Pseudoroseomonas	11.878	0.748	0.589	6.899e-01
Luteolibacter	24.040	0.501	0.397	6.919e-01
Actinomyces	520.914	0.297	0.239	7.097e-01
Pusillimonas	1729.460	-0.318	0.258	7.142e-01
Marinobacterium	1297.016	-0.437	0.358	7.142e-01
Lysobacter	182.469	0.258	0.211	7.142e-01
Modestobacter	94.398	-0.375	0.309	7.142e-01
Rugamonas	25.020	-0.509	0.414	7.142e-01
Rathayibacter	27.104	0.775	0.635	7.142e-01

Acidaminobacter	5.364	0.762	0.613	7.142e-01
Domibacillus	6.862	-1.401	1.183	7.142e-01
Fibroporia	7.444	-1.319	1.096	7.142e-01
Peptostreptococcus	6.314	-0.775	0.668	7.142e-01
Lasiodiplodia	27.457	-0.424	0.350	7.152e-01
Thioflexithrix	10.033	-0.532	0.448	7.160e-01
Citrobacter	14367.687	-0.347	0.289	7.172e-01
Moraxella	437.707	0.297	0.248	7.172e-01
Arthrobacter	566.114	0.215	0.182	7.262e-01
Uncultured	130.743	0.423	0.356	7.262e-01
Phaeodactylum	12.627	-0.615	0.523	7.262e-01
Patulibacter	9.106	0.880	0.743	7.262e-01
Pseudoduganella	5.798	-0.836	0.711	7.262e-01
Variovorax	87.909	-0.231	0.197	7.356e-01
Collimonas	7.742	-0.767	0.680	7.361e-01
Enterobacter	9658.205	-0.333	0.287	7.387e-01
Arcticiflavibacter	197.563	0.375	0.323	7.387e-01
Sparassis	5.100	-0.812	0.749	7.405e-01
Sporolactobacillus	110.272	-0.180	0.159	7.535e-01
Crocosphaera	13.832	0.448	0.396	7.535e-01
Dechloromonas	6.203	0.778	0.676	7.535e-01
Duganella	51.520	-0.377	0.337	7.680e-01
Microcystis	7129.664	0.408	0.365	7.681e-01
Gordonia	74.952	-0.398	0.358	7.681e-01
Faecalibacterium	9.066	-0.884	0.805	7.681e-01
Sedimentitalea	30.138	0.280	0.267	7.681e-01
Alicycliphilus	12.250	-0.497	0.447	7.681e-01
Fredinandcohnia	6.991	0.669	0.600	7.681e-01
Psychrobacter	94.340	0.275	0.250	7.772e-01
Lentibacillus	34.801	-0.287	0.263	7.789e-01
Achromobacter	178.806	0.142	0.131	7.827e-01
Cryptosporidium	15.505	0.535	0.490	7.827e-01
Roseovarius	20.641	0.374	0.353	7.827e-01
Lamprocystis	7995.423	-0.354	0.329	7.851e-01
Nostoc	288.872	0.210	0.197	7.851e-01
Alloscardovia	67.926	-0.447	0.419	7.851e-01
Guillardia	10.200	-1.115	1.047	7.851e-01
Algoriphagus	22.246	-0.351	0.330	7.851e-01
Thiolapillus	14.428	-0.773	0.724	7.851e-01
Enterovirga	5.894	1.020	0.940	7.851e-01
Tsukamurella	6.889	1.379	1.254	7.851e-01
Thiobacillus	5.354	-0.771	0.762	7.851e-01
Bavariicoccus	65.404	-0.342	0.323	7.859e-01
Enhydrobacter	51.501	0.298	0.281	7.859e-01
Wallemia	23.482	-0.985	0.935	7.944e-01
Thermomonas	34.874	-0.272	0.261	7.944e-01
Chrysosporum	14.164	-0.756	0.726	7.944e-01
Cardiobacterium	13.923	0.585	0.562	7.957e-01
Kwoniella	6.912	0.969	0.927	7.957e-01
Glycocaulis	61.217	1.134	1.091	8.038e-01
Thauera	36.207	0.242	0.236	8.038e-01
Synechocystis	15.469	0.670	0.653	8.049e-01
Salmonella	451.456	-0.163	0.161	8.128e-01
Abditibacterium	5.942	0.979	0.954	8.128e-01
Neisseria	439.766	-0.204	0.210	8.432e-01
Prevotella	338.175	-0.294	0.303	8.432e-01
Rubellimicrobium	30.281	0.521	0.535	8.432e-01
Lactobacillus	101.572	-0.190	0.199	8.432e-01
Candida	5.852	-0.693	0.619	8.432e-01

Aspergillus	77.673	-0.251	0.265	8.432e-01
Comamonadaceae_genus	99.630	-0.224	0.238	8.432e-01
Wolbachia	68.134	-0.384	0.405	8.432e-01
Geodermatophilus	32.121	-0.527	0.535	8.432e-01
Proteobacteria	19.452	-0.611	0.643	8.432e-01
Serinicoccus	17.817	0.753	0.788	8.432e-01
Kingella	8.778	0.490	0.510	8.432e-01
Puia	26.661	-0.286	0.295	8.432e-01
Neoantrodia	9.542	-0.813	0.870	8.432e-01
Acidiplasma	17.926	0.317	0.332	8.432e-01
Agathobacter	7.239	-0.946	1.004	8.432e-01
Aurantiacibacter	6.751	-0.729	0.791	8.432e-01
Diolcogaster_facetosa_bracovirus	12.306	-0.540	0.567	8.432e-01
Gryllotalpicola	6.324	-0.914	1.001	8.432e-01
Blastomonas	151.122	0.269	0.288	8.483e-01
Sphingobium	158.875	0.181	0.194	8.483e-01
Novosphingobium	521.937	-0.271	0.292	8.533e-01
Stutzerimonas	32.547	0.410	0.440	8.533e-01
Sinorhizobium	5.189	-0.687	0.800	8.639e-01
Ralstonia	14254.930	0.146	0.162	8.678e-01
Paracoccus	735.536	-0.155	0.172	8.678e-01
Halalkalibacter	112.475	0.275	0.303	8.678e-01
Lachnospira	17.322	-0.421	0.461	8.678e-01
Lacticaseibacillus	6.204	-0.574	0.662	8.678e-01
Marmoricola	34.294	-0.488	0.543	8.703e-01
Diaphorobacter	35.714	-0.337	0.378	8.703e-01
Plasmopara	6.866	-0.587	0.680	8.703e-01
Atlantibacter	14.246	-0.237	0.266	8.709e-01
Pelomonas	2109.665	-0.158	0.179	8.763e-01
Desertimonas	10.044	-0.774	0.886	8.763e-01
Thalassobius	5.691	-0.616	0.744	8.823e-01
Oryzomicrobium	63.319	-0.358	0.413	8.842e-01
Peredibacter	28.482	0.497	0.571	8.842e-01
Aliterella	5.985	0.897	1.020	8.884e-01
Babesia	6.351	-0.522	0.646	8.970e-01
Rheinheimera	4037.545	0.221	0.261	8.970e-01
Kocuria	406.292	0.331	0.393	8.970e-01
Fusobacterium	181.144	0.176	0.211	8.970e-01
Bosea	160.841	0.151	0.189	8.970e-01
Curtobacterium	171.096	-0.318	0.383	8.970e-01
Thermus	128.332	-0.344	0.408	8.970e-01
Acanthamoeba	49.409	-0.561	0.679	8.970e-01
Saccharopolyspora	62.086	0.169	0.213	8.970e-01
Paludifilum	42.859	-0.189	0.224	8.970e-01
Telluria	16.853	0.392	0.474	8.970e-01
Dermacoccus	44.902	0.256	0.304	8.970e-01
Calidithermus	19.574	-0.443	0.537	8.970e-01
Sinirhodobacter	15.801	-0.035	0.331	8.970e-01
Amnimonas	9.718	0.949	1.110	8.970e-01
Alteribacter	6.236	0.328	0.407	8.970e-01
Dialister	5.744	0.818	0.951	8.970e-01
Yersinia	118.324	-0.314	0.384	8.997e-01
Leifsonia	76.614	-0.311	0.380	8.997e-01
Solirubrobacter	19.559	0.449	0.552	9.003e-01
Schaalia	24.329	-0.373	0.462	9.003e-01
Methylorubrum	226.898	-0.135	0.170	9.008e-01
Blastococcus	150.500	0.307	0.383	9.008e-01
Mus_musculus_mobilized_virus	23.218	0.241	0.325	9.008e-01
Neomicrococcus	5.484	-0.515	0.688	9.008e-01

Mogibacterium	9.735	-0.503	0.629	9.008e-01
Nevskia	5.606	0.875	1.061	9.008e-01
Armatimonadetes	144.045	-0.512	0.645	9.019e-01
Olsenella	7.411	-0.611	0.797	9.019e-01
Buchnera	70221.134	0.145	0.186	9.020e-01
Phycicoccus	66.900	0.201	0.256	9.020e-01
Mycobacteriaceae_genus	41.727	-0.171	0.227	9.020e-01
Acuticoccus	38.672	0.149	0.252	9.020e-01
Gemmobacter	16.490	-0.522	0.665	9.020e-01
Tetrahymena	5.684	-0.490	0.666	9.020e-01
Mannheimia	5.616	-0.391	0.526	9.020e-01
Beijerinckiaceae_genus	37.739	0.544	0.699	9.046e-01
Fonsecaea	17.304	-0.453	0.585	9.058e-01
Conexibacter	10.772	0.525	0.685	9.072e-01
Candidate	14.431	-0.269	0.352	9.072e-01
Methylopila	6.130	0.653	0.841	9.072e-01
Picosynechococcus	8.815	0.458	0.698	9.072e-01
Caballeronia	39.742	-0.173	0.229	9.089e-01
Kaistella	17.043	0.360	0.477	9.092e-01
Allomeiothermus	372.615	-0.451	0.599	9.105e-01
Luteimonas	79.273	-0.399	0.533	9.133e-01
Phocaeicola	15.829	-0.461	0.616	9.176e-01
Agrobacterium	192.449	-0.135	0.190	9.218e-01
Protofrankia	62.094	0.271	0.369	9.218e-01
Cyclobacterium	9.163	-0.233	0.364	9.218e-01
Stenotrophomonas	1057.148	0.081	0.113	9.324e-01
Pseudoxanthomonas	75.899	0.211	0.299	9.324e-01
Leptolyngbya	41.961	0.283	0.403	9.324e-01
Mucilaginibacter	56.373	-0.213	0.299	9.324e-01
Penicillium	43.107	-0.255	0.361	9.324e-01
Rhizoctonia	10.173	-0.577	0.830	9.324e-01
Aquincola	15.393	0.349	0.490	9.324e-01
Amnibacterium	7.258	-0.529	0.781	9.324e-01
Saprolegnia	7.421	-0.391	0.574	9.324e-01
Rhodoplanes	8.916	-0.388	0.554	9.324e-01
Longimicrobium	6.592	-0.683	1.014	9.324e-01
Methylobacterium	5697.290	-0.125	0.183	9.333e-01
Corynebacterium	1916.937	0.131	0.227	9.333e-01
Malassezia	1094.910	0.143	0.267	9.333e-01
Clostridium	1001.246	0.115	0.188	9.333e-01
Mucor	15.990	0.589	0.849	9.333e-01
Protomyces	14.887	0.582	0.975	9.333e-01
Parastagonospora	59.095	-0.277	0.422	9.333e-01
Finegoldia	105.875	-0.328	0.551	9.333e-01
Elizabethkingia	205.340	-0.149	0.269	9.333e-01
Planococcus	196.283	-0.120	0.195	9.333e-01
Tatumella	7.235	0.362	0.603	9.333e-01
Halomonas	158.342	0.146	0.265	9.333e-01
Leptothrix	258.760	-0.177	0.333	9.333e-01
Neofusicoccum	40.723	0.272	0.451	9.333e-01
Deinococcus	166.493	-0.089	0.155	9.333e-01
Marinobacter	150.235	-0.146	0.245	9.333e-01
Thermoanaerobacterium	17.966	-0.573	0.929	9.333e-01
Lactococcus	112.493	0.246	0.400	9.333e-01
Brachybacterium	121.244	0.254	0.408	9.333e-01
Falsiroseomonas	137.429	-0.140	0.254	9.333e-01
Kallotenue	98.738	-0.367	0.578	9.333e-01
Mitosporidium	25.270	-0.877	1.409	9.333e-01
Agrococcus	150.918	0.384	0.647	9.333e-01

Ruficoccus	65.274	0.197	0.351	9.333e-01
Pseudonocardia	81.981	-0.255	0.385	9.333e-01
Cedecea	62.532	-0.261	0.403	9.333e-01
Chromohalobacter	65.014	-0.136	0.210	9.333e-01
Gardnerella	33.910	-0.329	0.616	9.333e-01
Metarhizium	16.962	0.372	0.547	9.333e-01
Chromatium	57.450	-0.135	0.208	9.333e-01
Rubrivivax	73.057	-0.216	0.340	9.333e-01
Sphingopyxis	66.886	-0.246	0.395	9.333e-01
Salipiger	55.657	-0.178	0.294	9.333e-01
Ensifer	36.688	0.159	0.309	9.333e-01
Pseudochrobactrum	75.819	-0.259	0.452	9.333e-01
Clostridiales	35.068	-0.209	0.385	9.333e-01
Actinomycetospora	19.821	-0.301	0.534	9.333e-01
Rhizobacter	51.463	0.138	0.309	9.333e-01
Noviherbaspirillum	23.982	-0.426	0.684	9.333e-01
Rhizorhapis	20.990	-0.306	0.462	9.333e-01
Rubrobacter	16.449	-0.369	0.634	9.333e-01
Hyphomicrobium	34.815	0.191	0.292	9.333e-01
Empedobacter	31.761	-0.241	0.370	9.333e-01
Pseudozyma	9.591	0.402	0.716	9.333e-01
Kytococcus	23.988	0.293	0.491	9.333e-01
Postia	8.620	0.484	0.792	9.333e-01
Parabacteroides	28.004	-0.207	0.341	9.333e-01
Polaromonas	23.598	0.178	0.300	9.333e-01
Aliiruegeria	16.063	0.304	0.576	9.333e-01
Lentzea	21.126	-0.173	0.300	9.333e-01
Sphaerulina	8.660	0.421	0.653	9.333e-01
Solihabitans	20.875	0.111	0.262	9.333e-01
Naegleria	20.303	-0.259	0.490	9.333e-01
Aggregatibacter	18.736	0.236	0.427	9.333e-01
Pestalotiopsis	18.985	-0.290	0.520	9.333e-01
Glaciibacter	18.801	-0.357	0.655	9.333e-01
Luteitalea	11.215	-0.569	0.944	9.333e-01
Halorubrum	12.131	-0.248	0.426	9.333e-01
Pararhizobium	9.293	-0.418	0.649	9.333e-01
Arachnia	16.113	-0.365	0.550	9.333e-01
Thermococcus	14.111	0.215	0.408	9.333e-01
Rhabdothermincola	8.157	-0.557	0.855	9.333e-01
Porphyromonadaceae_genus	12.235	-0.383	0.624	9.333e-01
Parainfluenza_virus_5	6.913	-0.579	1.136	9.333e-01
Terracoccus	15.641	0.454	0.780	9.333e-01
Ruminococcaceae_genus	14.329	-0.228	0.374	9.333e-01
Ichthyophthirius	11.762	-0.309	0.508	9.333e-01
Methylotenera	11.956	0.452	0.649	9.333e-01
Didymella	13.775	0.598	1.021	9.333e-01
Ottowia	13.988	0.300	0.476	9.333e-01
Dermabacter	7.786	-0.493	0.926	9.333e-01
Herbiconiux	5.773	0.666	0.985	9.333e-01
Seramator	7.459	0.432	0.733	9.333e-01
Methylibium	9.563	-0.478	0.828	9.333e-01
Hammondia	7.312	0.310	0.475	9.333e-01
Zimmermannella	7.604	0.419	0.766	9.333e-01
Georgenia	10.823	0.399	0.640	9.333e-01
Amorphotheca	6.328	0.364	0.670	9.333e-01
TM7	7.135	-0.421	0.646	9.333e-01
Caldimonas	5.324	-0.493	0.852	9.333e-01
Pedococcus	6.046	0.417	0.702	9.333e-01
Pneumocystis	5.632	0.692	1.258	9.333e-01

Gluconacetobacter	6.748	-0.314	0.573	9.333e-01
Kineosporia	6.745	-0.628	1.016	9.333e-01
Nitrobacter	5.995	-0.356	0.657	9.333e-01
Clavispora	9.617	-0.889	1.503	9.333e-01
Naumannella	5.114	-0.479	0.848	9.333e-01
Human_endogenous_retrovirus	5.191	0.307	0.536	9.333e-01
Perkinsus	5.227	-0.303	0.583	9.333e-01
Thermicanus	28.314	-0.501	0.947	9.372e-01
Phytobacter	11.988	-0.236	0.450	9.372e-01
Herbaspirillum	591.387	-0.105	0.203	9.378e-01
Colletotrichum	46.845	0.184	0.355	9.378e-01
Polaribacter	11.410	-0.229	0.444	9.378e-01
Myxococcus	31.942	-0.113	0.261	9.378e-01
Rhabdonatronobacter	170.476	0.124	0.242	9.387e-01
Alpha	67.476	0.145	0.284	9.388e-01
Pseudopropionibacterium	6.216	0.350	0.680	9.388e-01
Thermalbibacter	6.902	-0.487	1.011	9.388e-01
Aphanizomenon	21.520	0.043	0.263	9.394e-01
Granulicatella	27.984	0.218	0.437	9.395e-01
Leuconostoc	27.068	-0.161	0.320	9.395e-01
Dolosigranulum	11.557	0.342	0.680	9.395e-01
Shimia	358.445	-0.149	0.301	9.396e-01
Mesorhizobium	79.330	0.094	0.194	9.396e-01
Cercospora	12.008	-0.264	0.542	9.396e-01
Megasphaera	17.752	-0.332	0.670	9.396e-01
Melampsora	6.780	0.711	1.415	9.396e-01
Terrimonas	37.719	-0.235	0.482	9.439e-01
Moritella	186.504	0.076	0.166	9.497e-01
Bacteroides	124.758	-0.108	0.232	9.497e-01
Nitrosomonas	5.354	-0.348	0.802	9.497e-01
Microlunatus	62.692	0.192	0.410	9.501e-01
Bifidobacterium	47.754	0.161	0.340	9.501e-01
Burkholderiaceae_genus	42.128	-0.150	0.339	9.519e-01
Gallibacter	16.369	-0.368	0.814	9.519e-01
Prevotellaceae_genus	6.860	0.316	0.676	9.519e-01
Taibaiella	8.236	0.338	0.743	9.519e-01
Kribbella	6.342	-0.254	0.565	9.519e-01
Sandaracinobacteroides	6.329	0.336	0.761	9.519e-01
Polymorphobacter	8.366	-0.466	0.841	9.519e-01
Alcaligenes	8.410	0.284	0.605	9.519e-01
Sulfitobacter	6.435	0.209	0.460	9.519e-01
Salinibacterium	8.132	-0.257	0.621	9.562e-01
Escherichia	61277.548	0.129	0.298	9.577e-01
Brevundimonas	1147.754	0.101	0.236	9.577e-01
Cupriavidus	310.513	0.082	0.202	9.577e-01
Acaromyces	20.247	-0.361	0.877	9.577e-01
Jeotgalicoccus	20.422	-0.148	0.374	9.577e-01
Actinotalea	12.237	-0.294	0.672	9.577e-01
Leclercia	50.086	-0.095	0.234	9.577e-01
Kosakonia	17.606	-0.137	0.335	9.577e-01
Blautia	17.234	0.226	0.548	9.577e-01
Streptosporangium	49.775	-0.095	0.237	9.577e-01
Fomitiporia	7.709	-0.335	0.771	9.577e-01
Beggiatoa	32.646	-0.097	0.243	9.577e-01
Meiothermus	30.189	-0.231	0.561	9.577e-01
Oceanobacillus	36.670	0.096	0.285	9.577e-01
Tessaracoccus	17.444	0.285	0.642	9.577e-01
Acetobacter	16.394	0.119	0.335	9.577e-01
Azohydromonas	15.191	0.199	0.481	9.577e-01

Tetrasphaera	10.317	-0.278	0.666	9.577e-01
Brachymonas	13.279	0.181	0.443	9.577e-01
Gaiella	11.733	-0.401	0.965	9.577e-01
Belnapia	18.847	0.259	0.616	9.577e-01
Mediterraneibacter	9.994	-0.121	0.429	9.577e-01
Micropruina	6.588	-0.392	0.968	9.577e-01
Rhodomicrobium	7.805	-0.201	0.459	9.577e-01
Afipia	1012.669	-0.096	0.244	9.590e-01
Kushneria	83.057	-0.101	0.276	9.590e-01
Curvibacter	191.180	-0.058	0.148	9.590e-01
Zoogloea	42.077	0.134	0.350	9.590e-01
Jatrophihabitans	6.706	-0.389	1.031	9.590e-01
Adhaeribacter	5.729	0.403	1.090	9.590e-01
Enterobacteriaceae_genus	24.824	-0.087	0.286	9.591e-01
Fluoribacter	203.940	-0.105	0.274	9.614e-01
Phaeovulum	30.793	0.117	0.313	9.614e-01
Nocardiopsis	6.413	-0.343	0.968	9.614e-01
Shigella	210548.549	0.114	0.302	9.638e-01
Rhodotorula	31.421	0.207	0.545	9.638e-01
Nanosynbacter	12.801	-0.237	0.636	9.638e-01
Nocardioides	721.043	0.057	0.156	9.656e-01
Delftia	511.917	0.064	0.194	9.656e-01
Paracidovorax	147.082	0.110	0.324	9.656e-01
Actinoplanes	35.115	-0.130	0.366	9.656e-01
Phytophthora	90.681	-0.156	0.427	9.656e-01
Anoxybacillus	29.382	-0.226	0.644	9.656e-01
Phenylobacterium	59.083	0.120	0.350	9.656e-01
Trametes	10.328	0.248	0.738	9.656e-01
Piscinibacter	118.721	0.125	0.360	9.656e-01
Neobacillus	44.891	-0.084	0.242	9.656e-01
Rhodobacteraceae_genus	44.452	-0.156	0.474	9.656e-01
Atopomonas	30.225	-0.081	0.280	9.656e-01
Pseudomicrostroma	29.079	-0.291	0.853	9.656e-01
Lentilactobacillus	12.683	0.413	1.179	9.656e-01
Mycobacteroides	34.731	-0.237	0.648	9.656e-01
Desemzia	16.346	-0.177	0.511	9.656e-01
Burkholderiales	19.138	0.130	0.425	9.656e-01
Segatella	9.943	0.183	0.578	9.656e-01
Pyrococcus	12.784	-0.196	0.576	9.656e-01
Murine_type_C_virus	8.935	0.027	0.402	9.656e-01
Paucilactobacillus	8.021	-0.181	0.553	9.656e-01
Klenkia	7.551	-0.280	0.724	9.656e-01
Amycolatopsis	5.663	0.177	0.508	9.656e-01
Motilimonas	7.460	-0.153	0.535	9.656e-01
Paenimyroides	6.925	-0.702	0.959	9.656e-01
Caulobacter	1070.743	-0.076	0.237	9.670e-01
Cutibacterium	314.810	0.092	0.286	9.670e-01
Aureibaculum	51.891	-0.116	0.370	9.670e-01
Barnesiella	9.605	0.116	0.369	9.670e-01
Endosymbiont	5.833	-0.157	0.580	9.670e-01
Mycetohabitans	9.753	-0.088	0.450	9.670e-01
Mangrovicoccus	5.222	0.291	0.898	9.670e-01
Photobacterium	11.366	0.149	0.475	9.673e-01
Solobacterium	7.141	-0.190	0.677	9.673e-01
Cupidesulfovibrio	28.579	-0.120	0.402	9.705e-01
Oceanibium	5.401	-0.166	0.654	9.705e-01
Paraburkholderia	685.653	-0.048	0.161	9.706e-01
Fictibacillus	317.200	-0.106	0.347	9.706e-01
Aerococcus	77.226	0.085	0.287	9.706e-01

Terrisporobacter	682.815	0.096	0.323	9.717e-01
Methylobrevis	19.272	-0.099	0.412	9.717e-01
Facklamia	25.140	-0.091	0.354	9.724e-01
Klebsiella	1531.102	-0.060	0.227	9.737e-01
Aquabacterium	1382.076	0.059	0.244	9.737e-01
Massilia	397.055	0.074	0.286	9.737e-01
Kluyvera	247.288	0.043	0.189	9.737e-01
Roseibacterium	113.257	0.131	0.460	9.737e-01
Levilactobacillus	89.361	-0.136	0.472	9.737e-01
Roseomonas	144.562	0.110	0.387	9.737e-01
Aeromonas	84.822	-0.051	0.227	9.737e-01
Terrabacter	109.045	0.047	0.236	9.737e-01
Baudoinia	11.960	0.133	0.469	9.737e-01
Geminicoccus	65.368	-0.045	0.232	9.737e-01
Aquamicrobium	62.955	-0.089	0.335	9.737e-01
Allomuricauda	54.976	-0.080	0.344	9.737e-01
Maribellus	40.625	0.080	0.346	9.737e-01
Azospira	27.304	-0.053	0.312	9.737e-01
Rhodopseudomonas	31.634	0.081	0.352	9.737e-01
Aplosporella	8.752	-0.189	0.774	9.737e-01
Mycena	39.124	-0.199	0.827	9.737e-01
Fluviicola	6.799	-0.196	0.839	9.737e-01
Spirosoma	11.388	-0.158	0.612	9.737e-01
Sphingosinicella	7.978	-0.183	0.779	9.737e-01
Pseudoclavibacter	10.937	0.161	0.612	9.737e-01
Tepidicella	11.802	0.185	0.735	9.737e-01
Halovibrio	10.136	-0.138	0.623	9.737e-01
Bifidobacteriaceae_genus	8.416	0.257	0.912	9.737e-01
Alkanindiges	5.454	0.164	0.706	9.737e-01
Pseudorhodoferax	8.912	-0.182	0.650	9.737e-01
Bhargavaea	9.142	-0.128	0.600	9.737e-01
Tetragenococcus	9.450	-0.203	0.873	9.737e-01
Pigmentiphaga	5.865	0.199	0.739	9.737e-01
Pleionea	5.060	0.234	0.761	9.737e-01
Desulfitobacterium	6.033	-0.178	0.776	9.737e-01
Solimonas	5.862	-0.261	0.869	9.737e-01
Human_adenovirus_2	5.562	0.213	0.748	9.737e-01
Cereibacter	5.029	-0.180	0.786	9.737e-01
Trematosphaeria	5.185	-0.233	1.017	9.737e-01
Paeniglutamicibacter	17.218	0.001	0.374	9.745e-01
UNVERIFIED_ORG:	13.458	0.103	0.482	9.745e-01
Pantoea	228.056	-0.024	0.146	9.776e-01
Tannerella	323.303	0.062	0.322	9.776e-01
Fusarium	142.957	-0.100	0.575	9.776e-01
Tissierella	290.882	0.033	0.190	9.776e-01
Pauljensenia	155.649	-0.075	0.436	9.776e-01
Lysinibacillus	127.976	-0.061	0.370	9.776e-01
Stereum	17.542	0.128	0.775	9.776e-01
Micromonospora	101.944	-0.093	0.441	9.776e-01
Asticcacaulis	114.652	-0.080	0.404	9.776e-01
Erythrobacter	135.550	-0.020	0.227	9.776e-01
Trichosporon	103.764	-0.117	0.718	9.776e-01
Acidithiobacillus	129.947	0.079	0.475	9.776e-01
Dyadobacter	16.940	0.075	0.468	9.776e-01
Raoultella	68.520	-0.065	0.319	9.776e-01
Nocardia	68.412	-0.044	0.226	9.776e-01
Leptospira	69.642	-0.043	0.277	9.776e-01
Pseudacidovorax	63.479	0.049	0.310	9.776e-01
Phyllobacterium	31.179	0.087	0.503	9.776e-01

Niastella	28.805	0.246	1.215	9.776e-01
Dysgonamonadaceae_genus	13.608	0.114	0.722	9.776e-01
Quadrisphaera	9.631	0.135	0.827	9.776e-01
Paenirhodobacter	23.041	0.058	0.353	9.776e-01
Bordetella	18.960	0.080	0.384	9.776e-01
Limosilactobacillus	14.701	-0.061	0.434	9.776e-01
Ideonella	12.997	-0.095	0.561	9.776e-01
Psychromicrobium	11.336	0.005	0.299	9.776e-01
Actinobaculum	5.253	0.158	0.788	9.776e-01
Pyrinomonas	9.505	0.177	0.879	9.776e-01
Bartonella	9.625	0.112	0.557	9.776e-01
Ancylomarina	7.570	-0.068	0.560	9.776e-01
Solemya	6.351	-0.115	0.672	9.776e-01
Zychaea	6.500	0.285	1.374	9.776e-01
Cellvibrio	6.798	-0.158	0.730	9.776e-01
Algibacter	5.117	-0.100	0.550	9.776e-01
Aphanomyces	5.189	0.119	0.591	9.776e-01
Labilibaculum	7.087	-0.036	0.489	9.776e-01
Cytobacillus	13.792	0.215	1.179	9.776e-01
Cellulomonas	41.380	0.052	0.339	9.779e-01
Kurthia	83.993	-0.054	0.348	9.779e-01
Schizophyllum	63.317	-0.140	0.886	9.779e-01
Sphaerobacter	6.365	-0.180	1.178	9.779e-01
Lautropia	241.645	0.028	0.205	9.780e-01
Lentisphaera	29.992	0.015	0.267	9.780e-01
Betaproteobacterium_AAP51	11.054	0.050	0.549	9.780e-01
Acinetobacter	11818.492	0.014	0.154	9.785e-01
Bradyrhizobium	4376.605	0.017	0.178	9.785e-01
Burkholderia	2610.408	0.018	0.152	9.785e-01
Streptococcus	1476.802	-0.022	0.295	9.785e-01
Micrococcus	532.483	-0.029	0.273	9.785e-01
Acidovorax	744.728	-0.000	0.109	9.785e-01
Flavobacterium	706.963	0.021	0.155	9.785e-01
Rhizobium	451.252	0.010	0.162	9.785e-01
Comamonas	377.215	-0.020	0.215	9.785e-01
Plasmodium	260.330	0.015	0.167	9.785e-01
Zymoseptoria	34.389	-0.043	0.366	9.785e-01
Tepidimonas	39.113	-0.041	0.497	9.785e-01
Frankia	114.940	-0.034	0.284	9.785e-01
Allobacillus	31.886	-0.014	0.362	9.785e-01
Rhodobacter	87.074	-0.019	0.250	9.785e-01
Frigoribacterium	15.155	-0.065	0.630	9.785e-01
Microbispora	62.361	-0.023	0.302	9.785e-01
Hoylesella	15.245	0.059	0.557	9.785e-01
Actinokineospora	43.083	0.048	0.529	9.785e-01
Citricoccus	24.500	0.063	0.476	9.785e-01
Epilithonimonas	41.936	-0.017	0.362	9.785e-01
Chelatococcus	5.462	0.143	0.929	9.785e-01
Capnocytophaga	41.728	-0.029	0.303	9.785e-01
Saitoella	9.414	0.110	1.673	9.785e-01
Punctularia	10.023	0.123	0.882	9.785e-01
Suillus	20.978	-0.108	0.933	9.785e-01
Marinomonas	6.156	0.102	0.930	9.785e-01
Parasphingopyxis	15.233	0.022	0.635	9.785e-01
Kalmanozyma	5.083	-0.059	0.825	9.785e-01
Tolypothrix	6.280	0.028	0.815	9.785e-01
Coniophora	9.959	-0.130	1.023	9.785e-01
Eubacterium	18.755	-0.049	0.399	9.785e-01
Dioszegia	17.319	-0.084	0.940	9.785e-01

Brucella	13.265	0.027	0.433	9.785e-01
Eikenella	9.058	0.056	0.566	9.785e-01
Boeremia	20.842	0.077	0.841	9.785e-01
Shinella	19.684	0.048	0.542	9.785e-01
Lachnoanaerobaculum	14.139	0.028	0.406	9.785e-01
Saccharothrix	13.589	0.033	0.549	9.785e-01
Levyella	7.688	0.105	1.134	9.785e-01
Mobilicoccus	15.933	0.051	0.572	9.785e-01
Leucothrix	10.440	-0.023	0.474	9.785e-01
Methylophilus	8.689	0.019	0.539	9.785e-01
Weissella	5.780	-0.034	0.791	9.785e-01
Endocarpon	5.929	-0.079	0.659	9.785e-01
Yarrowia	13.249	0.105	0.776	9.785e-01
Yimella	7.431	0.042	0.729	9.785e-01
Ruegeria	9.018	0.002	0.690	9.785e-01
Renibacterium	7.693	0.092	0.785	9.785e-01
Homoserinimonas	5.839	0.102	0.748	9.785e-01
Barrientosiimonas	8.157	0.053	1.041	9.785e-01
Rhodanobacter	6.156	-0.089	0.631	9.785e-01
Ectobacillus	119.344	-0.015	0.355	9.799e-01
Atopobium	11.220	-0.026	0.646	9.799e-01
Rothia	240.951	-0.014	0.257	9.807e-01
Leucobacter	21.363	-0.015	0.397	9.807e-01
Alternaria	98.393	0.012	0.229	9.815e-01
Schlegelella	25.036	-0.022	0.436	9.820e-01
Cloacibacterium	73.077	-0.009	0.419	9.835e-01
Actinobacillus	273.019	0.013	0.342	9.849e-01
Marinifilum	38.998	-0.005	0.259	9.849e-01
Pontibacillus	7.036	0.062	1.264	9.874e-01
Dichomitus	29.347	-0.020	0.689	9.880e-01
Phytoplasma	68.366	0.008	0.312	9.880e-01
Heterobasidion	20.601	0.022	0.846	9.880e-01
Sphingobacterium	129.321	0.004	0.308	9.880e-01
Kineococcus	53.880	-0.004	0.675	9.880e-01
Citromicrobium	6.385	0.004	0.826	9.880e-01
Chelativorans	10.320	-0.020	0.809	9.880e-01
Betaproteobacterium_FWI2	1603.185	-0.008	0.339	9.885e-01
Aphanothece	13.799	-0.004	0.427	9.917e-01
Aeromicrobium	36.608	0.001	0.347	1.000e+00
Conchiformibius	7.052	0.021	0.789	1.000e+00
Pseudokineococcus	5.519	0.019	1.241	1.000e+00
Ancylobacter	5.043	0.004	0.939	1.000e+00
Arsenophonus	1.327	0.990	0.609	NA
Type-C_symbiont_of_Plautia_stali	0.212	1.245	2.754	NA
Sodalis-like	0.331	1.215	0.997	NA
Bacteria	0.118	-0.105	0.928	NA
Rhizopus	1.208	-0.156	1.506	NA
Type-D_symbiont_of_Plautia_stali	2.107	4.273	2.903	NA
Motilibacter	2.373	-1.532	1.424	NA
Type-F_symbiont_of_Plautia_stali	0.110	0.481	2.913	NA
Chaetomium	2.688	0.283	1.049	NA
Endozoicomonas	0.368	-0.575	1.254	NA
Profftia	0.070	0.429	2.912	NA
Plautia	0.323	1.504	1.596	NA
Type-E_symbiont_of_Plautia_stali	0.036	0.494	2.913	NA
Annandia	0.084	0.369	2.076	NA
Schizosaccharomyces	2.310	0.652	1.100	NA
Aedoeadaptatus	2.659	-0.330	1.330	NA
Desarmillaria	3.626	-0.485	0.809	NA

Flavisolibacter	0.705	-0.097	1.200	NA
Cronobacter	1.817	-1.471	0.926	NA
Emericellopsis	3.316	-3.420	1.363	NA
Hafnia	2.018	-0.087	0.470	NA
Sodalis	1.787	0.591	0.401	NA
Paludisphaera	3.132	-0.078	0.840	NA
Trabulsiella	0.462	0.481	0.604	NA
Phreatobacter	3.203	0.195	0.880	NA
Tissierellia	3.801	2.549	1.093	NA
Ethanoligenens	3.606	2.162	1.948	NA
Phycomyces	1.988	1.127	1.140	NA
Lelliottia	2.324	0.103	0.627	NA
Cnuella	0.966	0.677	1.744	NA
Weizmannia	1.405	-1.019	2.189	NA
Aquisphaera	2.453	0.935	1.240	NA
Radiomyces	1.508	-0.902	1.259	NA
Tautonia	1.653	1.504	1.503	NA
Lactiplantibacillus	4.188	-0.066	0.534	NA
Scedosporium	1.160	2.395	1.736	NA
Thermothelomyces	0.628	0.175	1.369	NA
Paracnuella	0.644	1.312	1.257	NA
Herbinix	3.173	-1.312	1.377	NA
Latilactobacillus	2.108	-0.726	1.309	NA
Sugiyamaella	3.235	-0.040	1.243	NA
Pseudactinotalea	0.679	-2.872	2.906	NA
Mangrovibacter	0.437	-0.743	0.648	NA
Fenollaria	1.816	-1.722	1.510	NA
Thiothrix	0.682	0.127	2.377	NA
Calothrix	3.097	1.153	0.920	NA
Pleurotus	2.254	-0.714	1.091	NA
Mikella	0.000	0.000	0.000	NA
Gullanella	1.395	3.244	0.899	NA
Fulvia	4.128	0.085	0.701	NA
Promicromonospora	1.177	-0.718	1.676	NA
Flaviaesturariibacter	0.706	-0.983	2.277	NA
Wenzhouxiangella	3.178	-0.233	0.815	NA
Phialophora	4.587	0.891	0.945	NA
Dacryopinax	2.313	-0.513	1.329	NA
Heyndrickxia	3.581	-2.388	1.347	NA
Ogataea	1.444	1.129	1.312	NA
Humisphaera	4.027	-0.637	1.315	NA
Planctomyces	2.081	0.232	1.042	NA
Apibacter	4.693	-0.298	0.995	NA
Caulobacteraceae_genus	0.901	0.377	2.003	NA
Prosthecobacter	4.698	1.440	0.983	NA
Ishikawaella	0.000	0.000	0.000	NA
Paracoccidioides	3.115	-0.839	0.809	NA
Haliangium	2.455	0.652	1.464	NA
Xenorhabdus	3.486	0.342	0.420	NA
Dickeya	1.888	1.297	0.421	NA
Phaeoacremonium	2.333	1.886	0.649	NA
Algiphilus	4.759	1.064	0.844	NA
Halopseudomonas	3.486	1.704	0.858	NA
Hafniaceae_genus	0.000	0.000	0.000	NA
Gammaproteobacteria	2.722	0.105	0.986	NA
Oceanospirillum	4.513	-0.181	0.622	NA
Varibaculum	3.474	-0.384	0.970	NA
Lachnellula	3.432	-0.021	0.797	NA
Pleomorphomonas	3.840	-0.637	1.123	NA

Labilithrix	4.216	-0.478	1.035	NA
Methylosinus	0.957	-0.046	1.081	NA
Caldibacillus	3.835	-1.486	0.798	NA
Lacrimispora	0.872	-0.543	1.734	NA
Grosmannia	2.456	2.528	1.465	NA
Pluralibacter	1.512	-1.991	1.279	NA
Flavihumibacter	2.570	-0.671	0.828	NA
Pectobacterium	2.621	-0.459	0.530	NA
Putridiphycobacter	2.985	0.517	0.762	NA
Brettanomyces	1.793	0.787	1.550	NA
Bathymodiolus	0.021	-0.132	2.913	NA
Segetibacter	3.587	-0.811	1.232	NA
Arsukibacterium	2.520	1.992	1.248	NA
Daldinia	4.862	-1.012	1.041	NA
Hoaglandella	0.398	2.243	2.102	NA
Microdochium	4.060	0.615	0.752	NA
Gilliamella	0.563	-0.079	1.188	NA
Aquihabitans	4.124	-1.251	1.059	NA
Rhodofomes	4.456	0.372	0.805	NA
Enteractinococcus	1.851	3.458	2.069	NA
Pediococcus	0.807	0.125	1.055	NA
Terrihabitans	1.488	-0.222	1.911	NA
Xylanimonas	2.036	0.340	0.910	NA
Superficieibacter	0.163	0.416	1.132	NA
Rosenbergiella	1.613	0.226	0.700	NA
Sporichthya	4.613	0.638	1.036	NA
Intestinirhabdus	0.996	0.068	0.421	NA
Alicyclobacillus	0.659	0.614	2.000	NA
Shigella_phage_SfIV_virus	0.000	0.000	0.000	NA
Limimaricola	2.701	0.278	1.413	NA
Jeotgalibacillus	1.068	-0.142	1.070	NA
Oceaniovalibus	4.526	-0.113	0.713	NA
Moranella	0.333	0.622	2.541	NA
Botrytis	2.262	2.553	1.024	NA
Pleomorpha	3.572	-0.021	0.885	NA
Laccaria	4.694	-0.056	0.747	NA
Blastomyces	1.591	-0.225	0.866	NA
Glaciihabitans	0.993	1.791	1.436	NA
Aureococcus	4.750	1.003	0.824	NA
Zygosaccharomyces	0.127	-0.720	2.913	NA
Bacidia	3.842	-0.024	0.899	NA
Oxalobacteraceae_genus	2.110	0.053	0.860	NA
Vallicoccus	3.729	-0.470	1.249	NA
Gemmatimonas	2.554	0.444	1.312	NA
Thyridium	3.802	-0.606	1.042	NA
Naasia	4.140	0.873	1.088	NA
Kuraishia	0.066	-0.219	2.913	NA
Chthonobacter	0.205	-1.156	2.911	NA
Baekduia	2.199	0.233	1.096	NA
Mediannikoviicoccus	0.000	0.000	0.000	NA
Isosphaera	0.330	1.702	2.908	NA
Thermohydrogenium	0.891	-0.858	1.973	NA
Aquimarina	0.749	1.117	1.941	NA
Tachikawaea	0.009	0.023	2.914	NA
Vagococcus	0.603	1.342	2.358	NA
Frondihabitans	4.024	-1.047	1.306	NA
Aggregicoccus	0.236	1.040	2.911	NA
Pontibacter	3.192	1.171	1.417	NA
Psilocybe	1.438	0.047	0.873	NA

Brevibacillus	1.944	1.443	0.907	NA
Conyzicola	1.722	-0.211	1.213	NA
Acidobacteria	2.858	0.920	0.897	NA
Bdellovibrio	0.992	0.310	1.273	NA
Miltoncostaea	4.350	0.292	1.331	NA
Eutypa	3.140	-0.695	0.838	NA
Niabella	0.539	0.079	1.812	NA
Pseudogemmobacter	0.912	1.417	2.181	NA
Truepera	3.557	0.052	1.075	NA
Pyrenophora	4.539	-1.123	0.846	NA
Roseisolibacter	3.912	-0.412	1.083	NA
Iamia	3.587	0.198	1.094	NA
Oscillochloris	0.443	-1.008	2.907	NA
Diplodia	4.466	-0.204	0.826	NA
Chlorogloea	3.479	1.290	1.293	NA
Faecalibacillus	0.089	-0.484	2.913	NA
Advenella	2.826	-1.080	1.003	NA
Altererythrobacter	4.874	0.155	0.924	NA
Capillimicrobium	2.546	1.764	1.234	NA
Propionicimonas	2.047	-0.246	1.065	NA
Gamsiella	4.971	1.029	1.107	NA
Microbacteriaceae_genus	4.620	3.320	1.098	NA
Pelagibacterium	3.573	-0.145	1.111	NA
Cadophora	1.997	-0.625	1.122	NA
Ilyonectria	3.065	1.201	1.078	NA
Actinomarinicola	1.651	-0.648	1.273	NA
Escherichia_phage_500465-1_virus	0.000	0.000	0.000	NA
Sporothrix	1.005	0.587	1.569	NA
Pyxidicoccus	1.671	-1.243	1.122	NA
Euzebya	0.588	0.547	1.253	NA
Leyella	2.026	0.935	1.218	NA
Coprococcus	3.166	-1.151	1.334	NA
Salinispora	1.822	-0.409	1.403	NA
Lacibacter	0.974	0.470	2.319	NA
Pararhodobacter	4.208	-0.685	0.758	NA
Emticicia	2.634	2.185	1.391	NA
Gemmiger	2.131	-2.774	1.360	NA
Coleofasciculus	1.654	1.520	1.276	NA
Mixia	2.238	1.059	1.186	NA
Tolumonas	0.873	-1.064	1.877	NA
Frankineae	1.636	-1.801	2.023	NA
Paraconexibacter	1.837	-0.453	1.110	NA
Niallia	0.268	0.475	1.971	NA
Umbelopsis	0.027	-0.247	2.913	NA
Minicystis	1.137	-0.114	1.776	NA
Fimbriimonas	4.578	-1.762	1.120	NA
Hydrogenophilus	2.886	-1.863	1.422	NA
Brenneria	3.075	-1.013	0.400	NA
Buttiauxella	1.130	0.739	0.984	NA
Exserohilum	2.273	-0.644	1.267	NA
Sanguibacter	2.434	-0.221	1.229	NA
Ferruginibacter	1.535	0.713	1.234	NA
Pengzhenrongella	1.275	0.596	1.497	NA
Enterobacteria_phage_DE3_virus	0.000	0.000	0.000	NA
Sediminibacterium	2.811	-0.111	1.148	NA
Riemerella	4.363	2.294	0.856	NA
Saccharibacteria	4.408	-0.659	0.987	NA
Fannyhessea	3.108	0.467	1.170	NA
Angustibacter	3.966	-0.127	1.148	NA

Entotheonella	3.765	0.271	0.682	NA
Pedomonas	4.318	0.474	1.070	NA
Duffyella	4.145	-0.799	0.733	NA
Cokeromyces	2.224	-0.951	0.475	NA
Roseobacter	0.876	-1.824	1.267	NA
Sporisorium	1.195	-0.023	1.312	NA
Chroococcidiopsis	2.687	-0.986	0.880	NA
Chryseomicrobium	0.799	-0.970	1.593	NA
Clavibacter	4.588	0.820	0.913	NA
Viridilinea	0.240	-0.179	2.911	NA
Terribacillus	0.063	0.115	2.913	NA
Pelorhabdus	4.813	0.344	0.712	NA
Arthroderma	0.451	-0.080	1.192	NA
Tardiphaga	3.851	0.624	0.743	NA
Paraferrimonas	4.903	0.106	0.652	NA
Tenebrionibacter	0.381	0.750	0.980	NA
Anatilimnocola	0.489	0.738	2.908	NA
Fortiea	2.790	-0.389	1.287	NA
Oleiphilus	1.965	-0.323	0.759	NA
Luteibacter	4.401	0.409	0.809	NA
Zafaria	0.027	0.076	2.914	NA
Polynucleobacter	1.891	1.252	0.913	NA
Winslowiella	0.065	-0.253	2.913	NA
Parerythrobacter	4.100	1.289	1.297	NA
Microcella	4.645	-0.098	0.851	NA
Myxococcales	1.509	0.630	1.927	NA
Coprobacillus	1.834	-0.932	1.198	NA
Pseudophaeobacter	4.670	-0.710	0.754	NA
Roseicella	3.466	0.728	0.948	NA
Azonexus	3.481	0.475	1.073	NA
Parageobacillus	1.709	0.442	1.417	NA
Leptomonas	2.477	0.571	1.066	NA
Oryzihumus	0.030	0.106	2.913	NA
Cobetia	0.433	0.411	1.432	NA
Paraprevotella	2.071	0.872	1.133	NA
Fervidibacillus	1.551	1.019	2.031	NA
Thermobacillus	0.214	-0.397	2.910	NA
Chondromyces	2.094	0.038	1.616	NA
Panacagrimonas	3.396	3.468	1.294	NA
Negativicoccus	1.337	3.641	2.904	NA
Chloroploca	0.537	-1.426	2.907	NA
Chitinophagaceae_genus	0.534	-1.407	2.292	NA
Actinobacteria	2.223	-0.050	1.630	NA
Yaniella	1.655	-0.284	1.248	NA
Berkiella	0.057	0.532	2.912	NA
Hephaestia	2.393	-0.286	1.200	NA
Parvimonas	4.539	0.090	0.806	NA
Rubrivirga	1.040	-2.252	2.099	NA
Zasmidium	1.710	-1.531	0.851	NA
Yoonia	0.283	-0.631	2.909	NA
Nitrosospira	1.058	0.382	1.572	NA
Porphyrobacter	4.254	0.234	0.623	NA
Fervidobacterium	0.190	-0.313	2.909	NA
Hallella	4.170	0.374	0.905	NA
Nitrotoga	4.783	-0.202	0.575	NA
Paecilomyces	3.711	0.497	0.899	NA
Sporosarcina	3.898	-0.207	0.743	NA
Puccinia	4.914	-0.179	0.786	NA
Pseudorhizobium	3.035	0.530	1.085	NA

Beutenbergia	4.200	0.077	1.107	NA
Enterobacteria_phage_P7_virus	0.000	0.000	0.000	NA
Pirellula	0.574	0.024	1.981	NA
Croceibacterium	2.842	-2.328	1.202	NA
Peptostreptococcaceae_genus	4.076	-0.338	0.879	NA
Halococcus	1.219	-2.341	0.803	NA
Thecamonas	1.583	0.588	1.341	NA
Galbitalea	3.500	-0.413	1.191	NA
Vogesella	3.725	0.911	0.906	NA
Moorella	0.192	-1.496	2.910	NA
Acidiferrimicrobium	0.646	-0.553	1.633	NA
Tepidiforma	3.376	-1.468	1.412	NA
Agreia	0.629	-0.609	2.006	NA
Plantibacter	4.413	-0.322	0.960	NA
Cohnella	3.253	1.683	0.665	NA
Westerdykella	3.310	0.068	0.897	NA
Microsporum	3.527	-0.402	1.039	NA
Oceanitalea	1.246	1.083	1.583	NA
Gloeocapsa	4.503	0.148	0.976	NA
Gulosibacter	3.895	0.998	1.078	NA
Flectobacillus	1.269	0.478	1.295	NA
Glaciimonas	3.779	-0.777	0.979	NA
Ezakiella	1.319	2.250	1.801	NA
Catenulispora	1.103	-1.414	2.334	NA
Peribacillus	4.798	0.674	0.919	NA
Acidothermus	1.557	-0.419	1.622	NA
Pectinatus	4.713	-0.166	0.697	NA
Pannonibacter	2.632	1.797	1.299	NA
Mangrovibacillus	2.743	-0.693	1.198	NA
Xanthocytophaga	2.896	-0.981	1.133	NA
Xanthomonadaceae_genus	3.912	-0.365	0.866	NA
Labilibacter	4.141	-0.250	0.840	NA
Azomonas	2.713	0.151	0.887	NA
Paludibacterium	3.956	-1.172	0.753	NA
Azoarcus	4.010	0.177	0.841	NA
Pelosinus	4.274	-0.344	0.863	NA
Escherichia_phage_RCS47_virus	0.000	0.000	0.000	NA
Acetobacterium	3.427	-0.297	0.956	NA
Acetivibrio	0.767	0.151	2.074	NA
Falsirhodobacter	4.108	-0.829	1.125	NA
Thermomicrobium	3.852	-0.801	0.844	NA
Neohortaea	3.383	-0.647	1.020	NA
Austwickia	2.277	0.025	1.154	NA
Saccharibacillus	2.894	0.654	1.316	NA
Synechococcus	0.899	1.378	1.397	NA
Pisolithus	0.864	0.232	0.897	NA
Archangium	2.807	0.911	0.788	NA
Sphaerotilus	3.359	-0.048	0.962	NA
Marinilactibacillus	3.912	0.316	1.072	NA
Enterobacteria_phage_phi80_virus	4.380	-0.157	0.881	NA
Firmicutes	1.284	0.642	1.199	NA
Aliarcobacter	1.118	-0.013	2.346	NA
Actinobacterium	1.502	0.446	1.610	NA
Flavimobilis	2.525	-0.222	1.064	NA
Salinicola	3.874	-0.123	0.551	NA
Azorhizobium	2.897	0.093	0.918	NA
Anaeroglobus	4.318	-0.069	0.776	NA
Fusibacter	1.384	2.048	1.722	NA
Megamonas	3.586	-0.606	1.005	NA

Armatimonas	0.268	-1.113	2.639	NA
Huaxiibacter	3.774	-0.481	0.921	NA
Ilyomonas	0.134	-1.260	2.911	NA
Cellulosimicrobium	4.141	1.260	1.256	NA
Nosocomiicoccus	1.257	-1.506	1.637	NA
Rhodocyclaceae_genus	4.038	0.568	0.883	NA
Roseococcus	1.779	1.312	1.335	NA
Sagittula	3.462	2.115	1.187	NA
Evansella	0.869	-0.043	2.113	NA
Pseudonocardiaceae_genus	1.243	1.522	1.624	NA
Salinarimonas	4.201	0.278	0.367	NA
Tricharina	1.265	0.731	1.202	NA
Methylovorus	3.354	-0.674	0.837	NA
Yonghaparkia	1.392	0.452	1.371	NA
Geodermatophilaceae_genus	1.281	-1.303	1.559	NA
Synchytrium	4.292	1.966	1.289	NA
Thermodesulfomicrobium	0.020	-0.312	2.913	NA
Eremococcus	4.175	0.247	0.873	NA
Neorhizobium	3.954	-0.118	0.974	NA
Prolinoborus	3.096	1.350	0.810	NA
Planifilum	0.365	-0.760	2.911	NA
Sutterella	3.974	-0.238	0.614	NA
SsRNA_phage_SRR5466369_2_virus	0.058	0.085	2.913	NA
Sandaracinus	1.493	-0.632	1.599	NA
Hyalangium	1.398	-0.505	2.052	NA
Catenibacterium	1.044	1.187	1.834	NA
Aciditerrimonas	0.568	2.386	1.641	NA
Collibacillus	0.630	-0.601	2.588	NA
Carbonactinospora	0.476	0.180	2.031	NA
Pseudofrankia	0.261	0.169	2.454	NA
Extensimonas	3.609	-0.603	1.014	NA
Hoyosella	0.809	-0.250	2.371	NA
Marisediminicola	1.015	2.983	2.093	NA
Ruania	2.191	-0.137	1.104	NA
Mongoliimonas	0.271	-1.798	2.909	NA
Snodgrassella	4.153	2.174	1.145	NA
Acidisphaera	1.711	0.177	1.528	NA
Fimbriiglobus	2.293	0.015	1.247	NA
Rhizophagus	3.679	1.005	1.040	NA
Moniliophthora	4.378	-1.023	0.900	NA
Fusicatenibacter	1.772	1.446	1.351	NA
Halalkalibacterium	3.247	-0.133	0.877	NA
Marinilabiliaceae_genus	2.283	0.204	0.780	NA
Halochromatium	4.224	-0.559	0.641	NA
Paracaedibacter	1.572	0.888	1.619	NA
Haladaptatus	2.468	1.204	0.776	NA
Lonsdalea	0.189	-0.346	0.901	NA
Protaetiibacter	2.315	-1.096	1.155	NA
Cryptosporangium	2.818	0.458	1.296	NA
Streptacidiphilus	2.022	-0.481	1.515	NA
Tsuneonella	2.101	0.554	1.400	NA
Zhihengliuella	1.833	-0.173	1.499	NA
Parasegetibacter	0.335	-1.315	2.910	NA
Oceanicola	3.328	-0.243	1.041	NA
Cryphonectria	1.076	-0.850	1.304	NA
Thermosipho	4.351	-0.346	0.418	NA
Alterileibacterium	0.250	-1.403	2.911	NA
Delta	4.266	0.132	0.590	NA
Alkalihalobacillus	4.260	1.764	0.562	NA

Parafrankia	2.012	-0.676	1.070	NA
Fonticella	0.702	-0.131	2.368	NA
Actinosynnema	3.106	-0.363	0.847	NA
Tabrizicola	4.228	1.994	0.823	NA
Grimontella	3.770	0.402	0.730	NA
Ehrlichia	3.296	-0.760	1.078	NA
Alkaliphilus	2.898	-2.002	1.405	NA
Leishmania	4.184	0.621	0.980	NA
Lagierella	0.000	0.000	0.000	NA
Ciceribacter	3.850	0.488	0.806	NA
Jannaschia	2.279	0.204	0.407	NA
Filifactor	2.916	-0.206	0.995	NA
Sorangium	1.090	-0.755	2.188	NA
Arcobacter	3.620	-0.631	0.820	NA
Aureispira	3.069	-0.727	0.765	NA
Gemmata	0.949	1.360	1.528	NA
Parasphingorhabdus	1.852	0.217	1.153	NA
Caenimonas	0.820	0.094	1.300	NA
Wolinella	1.486	2.612	1.614	NA
Herpetosiphon	1.350	-0.338	1.637	NA
Fretibacterium	3.170	-1.229	1.076	NA
Flaviflexus	0.626	1.230	1.671	NA
PreXMRV-1_provirus_complete	4.785	0.418	0.492	NA
Romboutsia	3.447	0.396	0.741	NA
Stappia	1.931	-1.409	1.287	NA
Asinibacterium	2.381	-0.913	0.905	NA
Uruburuella	0.038	0.550	2.913	NA
Acidiluteibacter	0.200	0.652	2.911	NA
Chthoniobacter	2.198	-1.433	1.450	NA
Trichophyton	4.105	1.174	1.387	NA
Psychrosphaera	4.364	0.887	0.623	NA
Catonella	3.258	-0.591	0.923	NA
Enterobacteria_phage_RTP_virus	2.620	0.269	0.907	NA
Aestuariimicrobium	3.255	0.823	0.972	NA
Brasilonema	1.390	0.965	1.497	NA
Enterobacteria_phage_T4_virus	2.981	-0.042	1.117	NA
Neofamilia	0.005	0.093	2.914	NA
Laetiporus	0.632	0.485	2.904	NA
Mycosynbacter	1.754	-0.594	1.260	NA
Terriglobus	1.251	0.389	1.215	NA
Enterobacteria_phage_vB_EcoS_IME542_virus	2.844	1.229	1.018	NA
Flavipsychrobacter	1.147	1.240	2.181	NA
Aliicoccus	2.437	4.058	1.668	NA
Kockovaella	3.506	0.947	1.009	NA
Epithele	1.083	-0.258	1.176	NA
Spleen_focus-forming_virus	3.983	0.758	0.465	NA
Cyberlindnera	2.022	-1.183	1.214	NA
Pseudoglutamicibacter	1.363	2.333	1.265	NA
Herminiimonas	2.008	-0.835	1.285	NA
Franconibacter	0.683	-0.777	1.241	NA
Paenacidovorax	3.180	-0.977	0.959	NA
Aquitalea	1.173	0.745	0.735	NA
Aliihoeflea	2.600	0.590	1.420	NA
Xinfangfangia	1.289	2.276	1.364	NA
Riesia	0.026	0.073	2.913	NA
Hirsutella	0.614	2.599	2.808	NA
Magnetospirillum	1.981	0.563	0.941	NA
Filomicrobium	2.369	0.831	1.071	NA
Escherichia_phage_phiV10_virus	2.464	0.399	0.983	NA

Companilactobacillus	0.662	-2.195	2.283	NA
Lancefieldella	4.241	2.049	1.205	NA
Secondary	3.884	-0.351	0.457	NA
Pseudosporangium	0.338	-0.444	2.910	NA
Cereal_yellow_dwarf_virus	0.223	1.693	2.909	NA
Tenebrionicola	0.031	0.468	2.913	NA
Anaerotruncus	0.786	0.504	0.869	NA
Acetanaerobacterium	0.863	0.449	1.632	NA
Sphingomonadaceae_genus	2.729	-0.106	1.420	NA
Paraglaciecola	2.599	0.672	0.643	NA
Vitreoscilla	2.267	3.525	1.400	NA
Rummeliibacillus	3.322	-0.033	0.831	NA
Lactovum	3.125	-0.354	1.066	NA
Pseudomassariella	0.846	0.276	2.026	NA
Friedmanniella	3.135	0.451	1.175	NA
Oscillibacter	0.819	0.852	2.379	NA
Desulforhabdus	2.687	1.588	1.051	NA
Alloiococcus	1.164	-0.086	1.293	NA
Minimicrobia	2.704	-0.923	0.970	NA
Rhizobiaceae_genus	0.018	-0.168	2.913	NA
Rhabdobacter	0.000	0.000	0.000	NA
Saccharomonospora	2.446	0.191	0.958	NA
Kallipyga	0.000	0.000	0.000	NA
Xylella	2.546	-0.337	0.607	NA
Kaistia	3.343	0.251	0.972	NA
Phascolarctobacterium	0.264	1.953	2.909	NA
Drepanopeziza	2.702	-0.522	1.081	NA
Mobiluncus	3.137	-0.944	1.024	NA
Wielerella	1.083	1.691	1.781	NA
Perlucidibaca	1.327	0.530	0.961	NA
Anaeromyxobacter	2.178	1.117	1.246	NA
Hoeflea	3.595	0.535	0.942	NA
Inhella	1.220	-0.896	1.577	NA
Gemmatirosa	2.401	-1.554	1.545	NA
Adlercreutzia	0.609	-0.261	1.882	NA
Brochothrix	2.027	-1.321	1.312	NA
Alsobacter	2.441	1.980	1.183	NA
Cystobacter	1.159	0.203	1.632	NA
Saccharomyces	2.333	-1.664	1.464	NA
Abelson	3.843	0.529	0.474	NA
Methylocystis	2.390	1.716	0.836	NA
Candidatus	2.663	0.111	1.355	NA
Abyssicoccus	5.009	-1.067	1.136	NA
Nitrolancea	0.665	0.453	1.720	NA
Tuber	2.943	-0.104	1.243	NA
Actibacterium	1.342	-0.471	1.295	NA
Propionibacteriaceae_genus	1.387	0.331	1.346	NA
Sordaria	1.077	0.897	1.186	NA
Pasteurellaceae_genus	2.823	0.162	0.907	NA
Oxalicibacterium	1.142	-1.248	1.397	NA
Aminobacter	2.429	1.623	0.916	NA
Pajaroellobacter	0.393	-0.918	2.693	NA
Flexivirga	2.311	-0.199	1.100	NA
Nannochloropsis	1.567	1.528	1.253	NA
Verrucosispora	2.100	1.082	0.952	NA
Escherichia_phage_500465-2_virus	0.000	0.000	0.000	NA
Microthrix	0.316	-1.773	2.910	NA
Methylocella	3.834	-0.470	1.246	NA
Oceanicella	2.913	0.899	0.870	NA

Luteococcus	0.817	0.637	1.983	NA
Methylococcus	3.490	-1.198	0.701	NA
Escherichia_virus_Lambda_2G7b	0.000	0.000	0.000	NA
Hahella	2.380	-0.251	0.743	NA
Sphingorhabdus	2.750	1.908	1.200	NA
Demequina	2.940	-0.973	1.053	NA
Type-B_symbiont_of_Plautia_stali	0.070	0.351	2.376	NA
Doolittlea	0.005	-0.080	2.914	NA
Zymomonas	0.330	1.579	2.909	NA
Photodesmus	0.007	-0.080	2.914	NA
Parachlamydia	0.456	2.546	2.874	NA
Komagataeibacter	1.071	0.327	1.522	NA
Starkeya	1.361	2.150	1.150	NA
Marasmius	3.117	-0.639	0.986	NA
Auritidibacter	1.179	-0.054	2.789	NA
Thalassotalea	1.950	-0.421	0.945	NA
Gallaecimonas	1.455	0.643	0.928	NA
Pseudoflavonifractor	1.265	1.115	0.986	NA
Dubosiella	0.165	0.859	2.866	NA
Amniculibacterium	0.863	-0.084	2.889	NA
Jiangella	1.425	-1.229	1.505	NA
Dactylosporangium	0.221	-0.393	2.909	NA
Dactylellina	2.854	3.434	1.286	NA
Alistipes	1.076	1.120	1.574	NA
Thermacetogenium	0.831	0.694	2.466	NA
Nitratireductor	1.522	0.570	0.970	NA
Pseudohongiella	0.062	0.798	2.912	NA
Richelia	0.517	2.124	1.653	NA
Lacipirellula	2.490	3.222	1.522	NA
Gallintestinimicrobium	0.837	1.165	2.604	NA
Drechmeria	0.424	-0.070	1.699	NA
Goekera	0.470	-1.476	2.561	NA
Agaricicola	0.028	0.157	2.914	NA
Pectobacterium_phage_CBB_virus	1.397	1.458	1.420	NA
Bacteriovorax	1.351	0.098	1.420	NA
Phaeosphaeria	2.821	0.716	1.533	NA
Methanothermobacter	0.156	-0.478	2.910	NA
Pedosphaera	0.186	1.519	2.910	NA
Acaricomes	2.228	-0.487	0.852	NA
Erysipelothrix	0.043	0.357	2.913	NA
Pseudorivibacter	1.087	-1.166	1.365	NA
Actinophytocola	1.418	0.425	1.612	NA
Desulfogranum	1.068	0.293	1.470	NA
Hyaloscypha	2.005	0.552	0.835	NA
Neoarthrinium	0.677	0.029	1.523	NA
Mycetocola	0.892	0.661	1.245	NA
Metabacillus	4.195	0.478	1.066	NA
Flintibacter	0.137	-0.864	2.911	NA
Anabaena	1.596	-0.005	1.203	NA
Gemmataceae_genus	0.813	1.177	1.852	NA
Cryptomonas	1.703	1.640	1.220	NA
Hansschlegelia	2.156	-0.274	1.357	NA
Peptococcus	0.662	-2.048	2.517	NA
Lichenibacterium	2.044	0.113	1.405	NA
Alloalcanivorax	2.425	-0.663	0.973	NA
Zavarzinella	0.480	-0.072	2.687	NA
Saccharophagus	0.820	0.387	1.790	NA
Pinibacter	0.395	-1.414	2.384	NA
Escherichia_phage_TL-2011b_virus	0.000	0.000	0.000	NA

Enterobacteria_phage_f1_virus	0.008	-0.080	2.914	NA
Pseudobdellovibrio	0.385	-0.618	2.039	NA
Sandaracinobacter	0.244	0.471	2.912	NA
Pelagivirga	2.684	-1.330	0.604	NA
Proteus_phage_VB_PmiS-Isfahan_virus	1.743	0.702	0.526	NA
Hydrocarboniphaga	1.904	-0.270	1.185	NA
Roseitranquillus	0.614	-0.491	2.088	NA
Halomicroarcula	0.504	2.366	0.897	NA
Calidifontibacter	0.577	1.100	2.193	NA
Hassallia	0.102	-0.158	2.914	NA
Siccibacter	0.129	-1.049	2.290	NA
Dissoconium	0.190	-0.141	2.911	NA
Quisquiliibacterium	0.626	1.500	1.957	NA
Halobacteriovorax	1.982	-0.532	0.585	NA
Pasteurella	1.606	-0.638	0.635	NA
Endobacter	3.493	0.291	1.179	NA
Defluviicoccus	0.374	-1.138	2.313	NA
Propionicicella	1.665	0.516	1.272	NA
Arthromitus	0.148	-0.866	2.912	NA
Melaminivora	2.129	-1.460	1.327	NA
Thermoanaerobacter	0.462	0.959	2.907	NA
Mycoavidus	1.599	2.510	1.560	NA
Thiomonas	1.348	-0.860	1.236	NA
Hemiselmis	0.919	-0.096	1.728	NA
Millisia	1.115	2.064	1.967	NA
Qingrenia	0.304	-1.978	2.909	NA
Piscicoccus	1.787	1.379	1.514	NA
Oceanimonas	1.818	-0.796	0.653	NA
Desulfocarbo	2.982	0.536	0.929	NA
Faecalicatena	0.820	-0.589	1.509	NA
Flavobacteriaceae_genus	0.949	3.053	1.305	NA
Sphingosinithalassobacter	0.506	-1.452	1.511	NA
Tychonema	1.638	0.785	1.130	NA
Nannocystis	1.346	-0.950	1.752	NA
Rhodovulum	0.928	-0.101	1.325	NA
Escherichia_virus_Lambda_4A7	0.000	0.000	0.000	NA
Macromonas	0.175	-0.136	2.909	NA
Coprinopsis	1.018	-1.466	1.550	NA
Micrococcaceae_genus	0.443	0.744	1.850	NA
Arsenicicoccus	2.165	-0.813	0.994	NA
Mameliella	2.131	-0.974	1.110	NA
Wickerhamomyces	2.456	-1.513	1.487	NA
Bergeriella	1.836	0.203	1.577	NA
Penaeicola	2.047	-0.001	0.899	NA
Sclerotinia	1.417	-0.616	1.265	NA
Verticillium	0.616	0.699	1.636	NA
harvey_murine_sarcoma_virus	0.947	0.044	0.470	NA
Rufibacter	1.318	-1.399	1.547	NA
Frateuria	2.007	1.811	0.731	NA
Halobacillus	0.907	0.995	1.169	NA
Sneathia	1.255	-0.734	2.166	NA
Cryobacterium	1.333	-1.026	1.721	NA
Sinisalibacter	0.394	0.050	1.946	NA
Centipeda	0.032	0.149	2.913	NA
Paraflavisolibacter	0.061	-0.038	2.914	NA
Gayadomonas	2.348	-1.033	0.523	NA
Sulfolobus	2.237	0.709	0.450	NA
Prauserella	0.186	1.610	2.910	NA
Siccirubricoccus	1.651	1.038	1.379	NA

Aquabacter	1.215	1.178	1.235	NA
Verrucomicrobia	1.426	1.553	1.637	NA
Thermincola	0.633	-1.654	2.355	NA
Purpureocillium	0.435	-2.164	2.881	NA
Haematobacter	4.763	-0.298	1.196	NA
Alkalicoccobacillus	0.836	-0.202	0.760	NA
Rhodospirillaceae_genus	1.267	1.310	1.339	NA
Paramagnetospirillum	1.385	-0.178	1.446	NA
Roseicitreum	2.086	0.863	1.196	NA
Robbsia	0.852	0.403	1.316	NA
Neurospora	0.942	-2.374	2.074	NA
Arenimonas	1.644	-0.731	1.325	NA
Anaerotardibacter	0.590	-2.115	1.805	NA
UNVERIFIED_CONTAM:	2.089	-1.848	1.200	NA
Pilimelia	1.968	-2.616	1.281	NA
Ustilago	1.167	1.286	1.328	NA
Schneideria	1.354	3.735	1.348	NA
Thermogemmata	1.690	-1.992	1.738	NA
Caldilinea	1.616	-1.771	1.729	NA
Streptoalloteichus	1.111	0.746	1.318	NA
Azovibrio	1.050	1.291	1.496	NA
Minwuia	0.831	1.935	2.595	NA
Methylomonas	1.557	-0.218	0.577	NA
Ignavibacterium	0.501	-0.951	2.906	NA
Scandinavium	0.642	1.109	0.609	NA
Veillonellaceae_genus	0.948	-0.821	2.015	NA
Intrasporangium	1.104	0.042	1.775	NA
Rhodospirillum	1.216	2.036	1.288	NA
Desulfotomaculum	0.503	-2.079	2.248	NA
Marichromatium	2.010	-0.264	0.587	NA
Desertibacillus	0.917	0.255	1.626	NA
Planctomycetes	0.709	2.133	1.666	NA
Propioniferax	0.646	-0.621	2.862	NA
Meyerozyma	1.925	-2.290	1.658	NA
Beijerinckia	0.400	0.036	2.207	NA
Gamma	2.482	1.454	1.062	NA
Myroides	1.389	-1.654	1.740	NA
Mariluticola	0.153	-1.209	2.911	NA
Garicola	1.949	-0.634	1.465	NA
Afifella	1.377	-1.972	1.732	NA
Coniosporium	2.486	-0.476	1.446	NA
Pusillibacter	0.000	0.000	0.000	NA
Simplicispira	1.925	0.527	0.934	NA
Psychromonas	1.967	0.318	0.654	NA
Stigmatella	0.992	-1.075	2.336	NA
Saezia	2.274	0.423	0.862	NA
Rectinema	0.081	0.963	2.912	NA
Ewingella	0.845	1.126	2.338	NA
Anaerobutyricum	0.119	0.149	2.911	NA
Klugiella	0.423	0.246	2.905	NA
Arcticibacter	0.736	-0.713	1.364	NA
Propylenella	0.014	-0.231	2.914	NA
Proteiniclasticum	0.907	0.875	0.826	NA
Seohaeicola	2.294	0.118	1.308	NA
Enterobacteria_phage_vB_EcoS_ACG-M12_virus	1.666	-1.293	1.110	NA
Phototrophicus	0.409	0.124	2.402	NA
Geoalkalibacter	0.121	-0.179	2.913	NA
Tomitella	1.308	1.488	1.325	NA
Pochonia	0.554	0.453	1.521	NA

Plastoroseomonas	0.681	1.015	2.006	NA
Lawsonibacter	0.311	0.279	2.232	NA
Globicatella	1.277	-0.929	1.769	NA
Plectonema	0.548	2.757	2.906	NA
Murine_osteosarcoma_virus	2.353	1.563	0.645	NA
Branchiibius	0.731	-0.454	2.318	NA
Parasutterella	0.614	1.411	1.300	NA
Plantactinospora	1.201	-1.692	1.218	NA
Insolitispirillum	2.216	-0.321	1.122	NA
Truncatella	0.941	2.415	1.399	NA
Mesonia	2.526	0.720	0.677	NA
Aaosphaeria	0.407	-0.440	2.309	NA
Bryobacter	0.568	-0.912	2.905	NA
Puniceibacterium	0.603	0.378	1.666	NA
Oscillatoria	1.378	-0.341	1.151	NA
Immundisolibacter	1.554	1.699	1.176	NA
Rhodoligotrophos	1.096	-0.529	1.653	NA
Escherichia_phage_Lambda_ev099_virus	0.000	0.000	0.000	NA
Bacteroidetes	1.184	1.916	1.826	NA
Crenalkalicoccus	0.463	1.147	2.059	NA
Andreesenia	0.720	0.815	2.568	NA
Klebsiella_phage_4_virus	0.000	0.000	0.000	NA
Catellicoccus	0.496	1.992	2.089	NA
Microvirgula	1.737	-0.240	1.610	NA
Rhodoblastus	1.061	0.468	1.557	NA
Wenxinia	0.425	0.213	1.863	NA
Planobispora	0.083	0.418	2.911	NA
Pyruvatibacter	1.367	0.793	0.753	NA
Mycoplana	0.055	-0.076	2.914	NA
Peptoclostridium	0.886	-0.248	1.730	NA
Aestuariibaculum	1.315	-0.281	1.146	NA
Kordiimonas	0.472	-0.143	2.119	NA
Maridesulfovibrio	0.637	2.634	2.906	NA
Neptuniibacter	0.092	0.681	2.911	NA
Stx2-converting_phage_1717_virus	0.000	0.000	0.000	NA
Planomonospora	0.303	-0.692	2.335	NA
Fischerella	0.513	2.683	2.518	NA
Theileria	0.473	-1.631	1.107	NA
Anaeromassilibacillus	0.075	-0.443	2.912	NA
Oerskovia	0.479	-0.210	2.525	NA
Rudaea	1.559	0.801	0.728	NA
Phormidium	0.945	-0.480	1.500	NA
Helcococcus	0.691	-0.573	2.347	NA
Glycomyces	0.831	1.628	1.807	NA
Cetobacterium	0.000	0.000	0.000	NA
Acidiphilium	0.973	-0.448	1.459	NA
Steroidobacter	1.182	1.738	1.304	NA
Hankyongella	1.915	-1.736	1.892	NA
Parachlamydiaceae_genus	0.346	1.148	2.907	NA
Actirhodobacter	0.195	1.414	2.910	NA
Methylocapsa	1.039	-0.452	1.167	NA
Escherichia_phage_Cartapus_virus	0.000	0.000	0.000	NA
Rhodocista	0.302	1.376	1.643	NA
Hubei_permutotetra-like_virus	2.387	4.378	1.506	NA
Coprothermobacter	0.362	-1.434	2.908	NA
Oligoflexus	0.666	-0.467	2.902	NA
Dothidotthia	2.037	-0.488	1.212	NA
Nonomuraea	1.439	-1.337	1.143	NA
Allosphingosinicella	0.640	-0.126	1.559	NA

Schumannella	0.440	-1.060	2.048	NA
Coriobacteriaceae_genus	0.483	0.050	2.380	NA
Nanoperiomorbus	1.710	0.075	1.362	NA
Pseudolabrys	0.793	1.301	1.529	NA
Castellaniella	1.341	0.493	1.493	NA
Baumannia	0.002	-0.034	2.914	NA
Erythrobacteraceae_genus	1.392	-0.603	1.313	NA
Phytohabitans	0.667	0.251	2.644	NA
Parvularcula	2.074	1.448	1.400	NA
Halophilic	1.709	-1.526	1.442	NA
Alkalibacterium	1.571	-1.681	1.491	NA
Salifodinibacter	1.550	-0.026	1.707	NA
Cecembia	0.302	-0.614	2.210	NA
Catellatospora	0.735	-1.363	2.812	NA
Xylaria	0.604	0.774	1.727	NA
Stakelama	0.615	0.964	1.562	NA
Polymorphum	0.458	0.503	1.741	NA
Reticulibacter	0.388	-0.390	2.906	NA
Rickettsiella	1.563	-1.092	0.761	NA
Nitrosocosmicus	0.792	0.710	1.862	NA
Planctopirus	0.379	0.596	1.506	NA
Ureibacillus	0.798	0.993	1.451	NA
Fastidiosipila	0.389	-0.319	2.910	NA
Carideicomes	0.567	-0.071	1.149	NA
Aliidongia	0.759	0.333	1.597	NA
Caldovatus	0.480	0.124	1.723	NA
Butyricicoccus	0.662	1.113	1.621	NA
Bacteroidales	0.562	0.048	1.729	NA
Emergencia	2.121	0.775	1.376	NA
Thermorudis	0.331	-0.021	2.759	NA
Rickettsia	2.419	2.289	1.286	NA
Granulicoccus	0.329	0.680	2.651	NA
Granulicella	0.978	-0.585	2.009	NA
Dinghuibacter	0.335	1.355	2.909	NA
SsRNA_phage_SRR5466337_3_virus	0.000	0.000	0.000	NA
Gregarina	0.341	-0.745	2.648	NA
Zeimonas	0.839	0.826	1.741	NA
Dokdonella	1.249	-2.801	1.548	NA
Kickxella	0.434	-0.358	1.982	NA
Lamprobacter	0.682	-1.057	1.306	NA
Escherichia_phage_Lambda_ev207_virus	0.000	0.000	0.000	NA
Paracandidimonas	0.784	0.348	2.133	NA
Aromatoleum	0.450	1.175	2.178	NA
Escherichia_virus_Lambda_1H12	0.000	0.000	0.000	NA
Solirhodobacter	0.058	0.761	2.912	NA
Lampropedia	1.172	0.029	1.552	NA
Niveispirillum	1.564	1.804	1.419	NA
FBR_murine_osteosarcoma_virus	1.393	1.279	0.593	NA
Tropicibacter	0.000	0.000	0.000	NA
Rivularia	0.632	1.139	1.992	NA
Nanosynsacchari	0.734	1.112	1.490	NA
Nitrososphaera	1.504	-1.519	2.175	NA
Actinoallomurus	1.044	-0.506	1.615	NA
Proteiniphilum	0.183	1.208	2.910	NA
Ramularia	0.158	0.423	2.913	NA
Glaesserella	0.891	0.954	0.900	NA
Rehaibacterium	0.368	0.846	1.985	NA
Auraticoccus	1.587	-0.949	1.998	NA
Halteromyces	0.737	-1.451	1.584	NA

Ustilaginoidea	1.194	-0.847	1.676	NA
Nocardioidaceae_genus	0.542	-0.955	2.638	NA
Cordyceps	1.093	1.465	1.613	NA
Micavibrio	0.689	1.623	1.791	NA
Variibacter	0.250	-1.764	2.910	NA
Enterobacteria_phage_T7_virus	0.002	0.039	2.914	NA
Methyloglobulus	0.637	-0.952	1.429	NA
Emiliania	0.907	0.339	1.183	NA
Oligella	1.184	3.463	1.973	NA
Dermatobacter	0.611	0.806	1.700	NA
Pseudocnuella	0.005	0.088	2.914	NA
Lachnoclostridium	1.068	-0.098	2.188	NA
Anaerobacillus	0.144	-0.476	2.910	NA
Formosimonas	0.082	-0.158	2.914	NA
Dongshaea	0.131	0.404	2.910	NA
Limobrevibacterium	0.486	-0.358	1.738	NA
Embleya	1.575	0.488	1.423	NA
Desulfofundulus	0.368	0.082	2.910	NA
Thermosinus	0.296	-0.634	2.908	NA
Idiomarina	0.506	2.402	2.553	NA
Youxingia	0.215	0.810	2.491	NA
Desulfuromonas	1.691	1.510	1.449	NA
Stx2-converting_phage_Stx2a_WGPS2_virus	0.000	0.000	0.000	NA
Flavonifractor	0.231	1.009	2.910	NA
Paeniroseomonas	0.513	1.602	1.681	NA
Phaeobacter	1.043	0.692	0.833	NA
Paraphaeosphaeria	0.868	-0.851	2.351	NA
Thioalkalivibrio	1.056	1.974	1.285	NA
Subtercola	0.858	2.741	1.738	NA
Ruoffia	0.404	-0.131	1.986	NA
Paraflavitalea	0.310	-0.741	2.909	NA
Crenobacter	1.097	0.765	0.886	NA
Methylophaga	0.781	0.497	1.190	NA
Eremomyces	0.488	0.191	2.202	NA
Paracraurococcus	0.696	0.568	1.732	NA
Hyphomonas	1.185	-0.015	1.689	NA
Faecalimonas	0.765	1.454	1.578	NA
Actinoalloteichus	0.689	-0.016	1.343	NA
Durotheca	0.146	1.284	2.910	NA
Rhodospirillales	0.991	1.697	1.627	NA
Holdemania	0.350	1.645	2.907	NA
Butyrivibrio	0.850	-1.077	1.751	NA
Moheibacter	0.373	1.086	2.622	NA
Sabulicella	0.126	-0.984	2.911	NA
Agathobaculum	0.083	0.975	2.912	NA
Escherichia_phage_Lambda_ev243_virus	0.000	0.000	0.000	NA
Petrimonas	0.193	0.819	2.911	NA
Pseudorhodoplanes	0.962	1.122	1.224	NA
Geomicrobium	0.127	-0.372	2.911	NA
Coriobacteriales	0.346	1.669	2.660	NA
Mumia	1.203	-1.577	1.770	NA
Laspinema	0.879	0.903	1.847	NA
Natronorubrum	1.279	0.687	1.378	NA
Aeribacillus	0.369	-0.569	2.912	NA
Aquimonas	1.549	1.470	1.935	NA
Gellertiella	2.945	-0.547	1.511	NA
Isoalcanivorax	1.062	0.903	1.734	NA
Neoactinobaculum	0.622	1.677	2.102	NA
Eimeria	0.728	0.571	1.007	NA

Aquibium	0.920	0.784	1.667	NA
Dankookia	1.052	-1.925	1.367	NA
Falsochrobactrum	0.107	0.115	2.912	NA
Acidisoma	1.255	-0.272	1.486	NA
Couchioplanes	0.000	0.000	0.000	NA
Yeguia	0.064	0.042	2.912	NA
Variimorphobacter	0.014	0.226	2.913	NA
Citreicoccus	0.220	1.677	2.909	NA
Serpentinimonas	0.193	0.881	2.909	NA
Ferribacterium	0.267	0.603	2.908	NA
Xanthomarina	0.828	1.504	1.547	NA
Miniimonas	0.445	0.703	2.405	NA
Pelagerythrobacter	0.521	-1.691	2.561	NA
Allocoleopsis	0.836	1.295	1.735	NA
Parvibaculum	1.055	-1.706	1.296	NA
Microterricola	0.560	2.221	2.181	NA
Algibacillus	1.251	0.370	0.931	NA
Simian_virus_40	1.059	-0.214	0.771	NA
Anaerosphaera	0.551	0.323	2.148	NA
Saliphagus	1.228	-1.259	1.130	NA
Labedella	0.708	1.466	2.012	NA
Erysipelotrichaceae_genus	0.333	1.681	2.908	NA
Frischella	1.199	-1.343	0.871	NA
Pichia	0.685	0.304	1.355	NA
Morganella	1.022	0.218	0.571	NA
Chlamydia	0.283	1.929	2.677	NA
Aridibaculum	0.190	0.561	2.912	NA
Allostreptomyces	0.466	2.516	2.554	NA
Lentihominibacter	0.331	-0.530	2.158	NA
Wigglesworthia	1.349	0.437	0.842	NA
Escherichia_virus_Lambda_2H10	0.000	0.000	0.000	NA
Hypoxylon	1.453	-1.693	1.419	NA
Chiayiivirga	0.821	-2.537	2.071	NA
Vibrionimonas	0.863	-0.191	1.858	NA
Krasilnikoviella	0.109	0.339	2.911	NA
Lederbergia	0.161	1.047	2.794	NA
Gulbenkiania	0.803	-0.912	1.773	NA
Methanothrix	0.009	-0.148	2.914	NA
Catenuloplanes	0.057	-0.698	2.913	NA
Scardovia	0.267	-1.155	2.910	NA
Acidocella	0.541	0.245	1.509	NA
Chloroflexi	0.505	-0.044	1.490	NA
Linderina	1.155	-0.285	1.070	NA
Rouxiella	0.547	-1.436	1.820	NA
Faunimonas	0.206	0.284	2.825	NA
Zobellella	1.254	0.167	1.632	NA
Protochlamydia	0.307	-0.697	2.907	NA
Mycoplasmopsis	1.529	-0.478	1.103	NA
Tuwongella	0.369	0.625	2.908	NA
Hydromonas	0.010	0.076	2.914	NA
Soleaferrea	0.148	0.954	2.910	NA
Liquorilactobacillus	0.655	1.871	1.658	NA
Thermobrachium	0.000	0.000	0.000	NA
Fuscibacter	0.854	0.319	2.071	NA
Amygdalobacter	0.251	0.231	2.909	NA
Rodentibacter	0.334	1.750	1.787	NA
Sphingomonas-like	0.813	-2.171	1.150	NA
Intrasporangiaceae_genus	0.940	-0.270	1.266	NA
Gynurincola	0.054	0.719	2.912	NA

Sandarakinorhabdus	1.376	-0.237	1.901	NA
Mycoplasma	1.308	1.249	0.791	NA
Lipomyces	0.225	-0.847	2.756	NA
Mariprofundus	0.047	-0.352	2.913	NA
Salicibibacter	0.350	0.952	2.907	NA
Urbifossiella	0.741	0.091	2.168	NA
Metasolibacillus	1.157	0.969	1.243	NA
Lignipirellula	0.509	2.413	2.556	NA
Slackia	0.721	2.064	1.803	NA
Rhodovibrio	0.588	1.356	1.962	NA
Acidobacteriaceae_genus	0.311	1.027	2.443	NA
Nanogingivalis	0.227	0.617	1.911	NA
Aquariibacter	0.913	0.399	1.281	NA
Haematomicrobium	1.237	-2.801	2.091	NA
Acidimicrobium	0.273	-0.899	2.912	NA
Kirsten_murine_sarcoma_virus	1.366	2.368	1.522	NA
Runella	0.324	1.276	2.119	NA
Lindgomyces	0.333	-1.103	2.706	NA
Gallionella	0.539	0.777	1.002	NA
Fodinicola	0.339	1.234	2.673	NA
Fontibacillus	0.151	0.078	2.910	NA
Puteibacter	1.005	-0.046	1.139	NA
Mesobacillus	1.096	-0.201	1.371	NA
Enterobacteria_phage_YYZ-2008_virus	0.009	0.076	2.914	NA
Vulcaniibacterium	2.155	-0.889	1.571	NA
Calidifontimicrobium	0.381	0.300	2.910	NA
Elioraea	0.452	-0.218	2.025	NA
Quatrionicoccus	0.245	1.757	2.909	NA
Thiofilum	1.021	-1.362	1.960	NA
Virgisporangium	0.313	0.371	2.056	NA
Capsulimonas	0.344	0.377	2.020	NA
Nisaea	0.072	0.886	2.912	NA
Oryzibacter	0.481	0.135	2.210	NA
Segnochrobactrum	1.097	2.590	1.969	NA
Debaryomyces	1.098	-2.057	1.843	NA
Spirilliplanes	0.536	-2.237	2.908	NA
Malikia	0.578	1.345	1.964	NA
Nigerium	0.251	-0.766	2.613	NA
Pleurocapsa	1.217	-1.188	1.641	NA
Thalassolituus	1.194	-0.706	1.062	NA
Pseudovibrio	0.453	0.265	2.119	NA
Histoplasma	0.752	-0.110	1.228	NA
Deferrisoma	0.000	0.000	0.000	NA
Pelobacter	1.260	-1.743	1.626	NA
Escherichia_phage_vB_EcoS_ESCO41_virus	1.030	0.034	1.405	NA
Papillibacter	0.041	0.584	2.913	NA
Acytostelium	0.807	-0.287	0.832	NA
Rickettsiales	0.188	-0.565	2.501	NA
Dysosmobacter	0.526	1.616	2.108	NA
Morchella	0.641	-0.294	2.384	NA
Neoroseomonas	0.161	0.209	2.910	NA
Tahibacter	0.454	0.996	1.560	NA
Propionibacterium_phage_PHL041M10_virus	0.037	0.330	2.913	NA
Rugosimonospora	0.425	-0.855	2.908	NA
Aff.	0.263	1.222	2.908	NA
Pseudaminobacter	1.415	-0.721	1.537	NA
Sporomusaceae_genus	0.444	1.091	1.286	NA
Anaerostipes	0.192	0.096	1.879	NA
Chloroflexia	0.169	-0.236	2.910	NA

Mitsuokella	0.467	-0.299	2.688	NA
Propionibacterium_phage_PHL301M00_virus	0.111	-0.294	2.911	NA
Pseudenterobacter	0.515	-0.433	0.530	NA
Trujillella	0.289	1.920	2.370	NA
Atlanticothrix	0.252	0.193	2.510	NA
Hominisplanchenecus	0.033	-0.463	2.913	NA
Vescimonas	0.025	-0.259	2.913	NA
Thermaurantiacus	0.224	1.368	2.909	NA
Provencibacterium	0.082	0.357	2.912	NA
Teredinibacter	0.038	0.548	2.913	NA
Pseudidiomarina	0.129	0.397	1.820	NA
Polysphondylium	0.471	-0.101	2.521	NA
Rhodophyticola	0.237	0.583	2.908	NA
Cucumibacter	0.022	0.326	2.913	NA
Planomicrobium	4.483	2.335	1.436	NA
Scleromatobacter	0.808	0.126	2.376	NA
Cucurbitaria	0.669	0.676	1.580	NA
Falseniella	0.389	1.611	2.323	NA
Parapusillimonas	0.200	1.198	2.911	NA
Falcatimonas	0.017	-0.267	2.913	NA
Lujinxingia	0.179	1.513	1.821	NA
Occultella	0.037	-0.412	2.913	NA
Nitrospira	0.501	1.421	2.052	NA
Ornithinicoccus	0.159	1.464	2.816	NA
Halovulum	1.008	0.261	1.556	NA
Microbacter	0.671	0.085	1.708	NA
Croceicoccus	0.401	-1.762	1.505	NA
Parapedobacter	0.475	1.198	1.322	NA
Acidaminococcus	0.185	1.043	2.909	NA
Frigoriflavimonas	0.492	2.263	1.911	NA
Geomonas	0.050	0.675	2.912	NA
Alkalihalophilus	0.036	0.521	2.913	NA
Hydrobacter	0.593	1.056	2.000	NA
Thermoleophilum	0.477	0.058	2.355	NA
Pyricularia	0.300	1.362	2.378	NA
Humibacter	0.118	1.061	2.911	NA
Phytoactinopolyspora	0.463	-0.521	2.405	NA
Paenalcaligenes	0.760	1.435	0.906	NA
Neokomagataea	0.142	0.770	2.910	NA
Drancourtella	0.294	-0.287	2.673	NA
Larkinella	0.768	-1.494	1.610	NA
Escherichia_phage_D6_virus	0.000	0.000	0.000	NA
Desulfoscipio	0.435	0.608	2.377	NA
Saccharimonas	0.553	-0.504	1.958	NA
Neglectibacter	0.247	0.147	1.381	NA
Sinomonas	0.642	-0.117	2.736	NA
Zoogloeaceae_genus	0.238	1.216	2.909	NA
Escherichia_phage_DTL_virus	0.756	0.390	1.877	NA
Vampirovibrio	0.259	1.241	2.908	NA
Coccidioides	0.857	0.313	1.201	NA
Gramella	1.113	-0.695	0.812	NA
Pseudodesulfovibrio	0.057	-0.119	2.914	NA
Simonsiella	0.622	-0.326	2.073	NA
Allorhizobium	0.157	0.549	2.910	NA
Pseudaestuariivita	0.064	0.821	2.912	NA
Macellibacteroides	0.431	0.973	2.117	NA
Caenispirillum	0.299	-1.963	2.909	NA
Jeotgalibaca	0.592	0.647	1.875	NA
Hydrotalea	0.322	0.377	2.667	NA

Desnuesiella	0.296	1.330	2.911	NA
BeAn_58058_virus	0.384	0.151	1.138	NA
Yinghuangia	0.547	-0.258	1.527	NA
Congregibacter	0.755	-1.445	0.928	NA
Enterobacteria_phage_Sf6_virus	0.000	0.000	0.000	NA
Methyloradius	0.301	-1.025	2.594	NA
Robiginitalea	0.271	-0.913	2.654	NA
Usitatibacter	0.008	-0.140	2.914	NA
Haloechinothrix	0.329	1.292	2.370	NA
Calorimonas	0.022	-0.332	2.913	NA
Aestuariivirga	0.428	-0.627	2.400	NA
Tistrella	0.157	0.273	2.910	NA
Sphaerisporangium	0.405	2.181	2.595	NA
Nannizzia	1.520	-2.106	1.221	NA
Haliea	0.570	0.077	1.371	NA
Siphonobacter	1.123	3.348	1.978	NA
Nioella	0.006	0.076	2.914	NA
Shouchella	0.581	0.813	2.727	NA
Chloroflexales	0.074	0.901	2.912	NA
Arenivirga	0.339	0.274	2.908	NA
Ktedonobacter	0.238	0.354	2.908	NA
Anaerobiospirillum	0.687	2.712	2.231	NA
Pelistega	0.345	1.749	2.909	NA
Arboricoccus	0.363	1.155	2.306	NA
Roseibium	0.641	0.803	1.624	NA
Propionispora	0.201	1.564	2.909	NA
Odoribacter	0.406	2.411	2.574	NA
Miniphocaeibacter	0.034	0.215	2.913	NA
Paenarthrobacter	0.311	-0.522	2.653	NA
Betaproteobacteria	0.088	0.156	2.912	NA
Betaproteobacterium_AAP65	0.934	0.368	1.094	NA
Albitalea	0.203	0.589	2.824	NA
Trichococcus	0.181	1.490	2.801	NA
Rhodocyclus	0.572	1.295	1.508	NA
Tetzosporium	0.121	-0.674	2.912	NA
Mesomycoplasma	0.567	-1.432	2.071	NA
Elstera	0.783	-0.700	2.170	NA
Haloferax	0.284	-0.246	2.685	NA
Brooklawnia	0.606	1.856	2.088	NA
Aquirhabdus	0.376	-1.499	2.910	NA
Neptunicoccus	0.253	-0.998	2.909	NA
Methylocaldum	0.238	0.548	1.757	NA
Limnobaculum	0.794	-1.533	1.085	NA
Viridibacillus	0.463	-0.745	2.026	NA
Aggregatilinea	0.220	0.456	2.909	NA
Primorskyibacter	0.613	0.063	0.939	NA
Fontimonas	0.265	0.286	2.774	NA
Aceticella	0.332	-1.663	2.910	NA
Argonema	0.623	-0.035	2.156	NA
Flagellatimonas	0.126	1.279	2.911	NA
Weeksella	0.444	0.886	2.912	NA
Gloeothece	0.000	0.000	0.000	NA
Camelimonas	0.584	0.475	2.193	NA
Silanimonas	0.443	-2.261	2.538	NA
Inquilinus	0.544	0.645	1.432	NA
Atopococcus	0.184	1.462	2.910	NA
Prosthecomicrobium	0.331	0.036	2.355	NA
Propionibacterium_phage_PHL117M01_virus	0.268	-1.421	2.910	NA
Carboxylicivirga	0.110	1.166	2.911	NA

Thermopolyspora	0.034	-0.470	2.913	NA
Filamentous	0.701	1.372	1.777	NA
Rubricoccus	0.152	0.351	2.913	NA
Ferrovum	0.569	1.634	2.323	NA
Tepidanaerobacter	0.074	-0.850	2.912	NA
Sulfuriferula	0.013	0.209	2.914	NA
Idiomarinaceae_genus	0.023	0.276	2.913	NA
Chryseosolibacter	0.194	0.709	2.909	NA
Komarekiella	0.170	1.432	2.910	NA
Pelovirga	0.002	0.030	2.914	NA
Pirellulimonas	0.064	0.813	2.912	NA
Propionibacterium_phage_PHL116M00_virus	0.473	1.313	2.655	NA
Paenisporosarcina	0.088	-0.834	2.912	NA
Salegentibacter	0.151	0.274	2.910	NA
Muribaculaceae_genus	0.949	-1.209	1.260	NA
Arachidicoccus	0.202	0.818	2.911	NA
Sulfuricystis	0.442	1.520	2.608	NA
Luteipulveratus	0.361	0.063	2.609	NA
Chryseolinea	0.170	0.338	2.912	NA
Pontibrevibacter	0.195	1.655	2.910	NA
Hanamia	0.253	1.504	2.910	NA
Penicilliopsis	0.668	0.840	2.322	NA
Podospora	0.781	-0.175	0.807	NA
Grimontia	0.335	-0.285	0.781	NA
Thioclava	0.294	1.172	2.314	NA
Propionibacterium_phage_SKKY_virus	0.362	-0.876	2.908	NA
Citreimonas	0.088	1.014	2.911	NA
Propionibacterium_phage_PAD20_virus	0.688	-1.117	2.904	NA
Planococcaceae_genus	0.288	0.064	2.907	NA
Caldicellulosiruptor	0.237	0.876	2.908	NA
Hartmannibacter	0.288	0.758	2.907	NA
Helicobacter	0.471	0.419	2.182	NA
Verticiella	0.592	-0.455	2.351	NA
Pseudescherichia	0.152	0.610	2.557	NA
Akanthomyces	0.281	-0.364	2.908	NA
Plasticicumulans	0.289	-1.925	2.909	NA
Dongia	0.909	-0.300	1.667	NA
Escherichia_phage_520873_virus	0.000	0.000	0.000	NA
Bergeyella	0.183	1.597	2.786	NA
Tersicoccus	1.189	-2.016	2.147	NA
Stenoxybacter	0.289	-0.316	2.908	NA
Cronobacter_phage_vB_CsaM_GAP32_virus	0.325	-1.037	2.624	NA
Plesiocystis	0.041	-0.397	2.913	NA
Holdemanella	0.771	1.808	1.836	NA
Torulaspora	0.428	-0.331	2.905	NA
Sulfuritalea	0.539	-2.593	2.907	NA
Streptobacillus	0.298	0.515	2.907	NA
Viadribacter	0.000	0.000	0.000	NA
Betaproteobacterium_AAP121	0.906	0.206	1.097	NA
Jaminaea	0.197	-0.152	2.910	NA
Propionibacterium_phage_P100D_virus	0.085	0.991	2.911	NA
Asanoa	0.412	-1.233	2.908	NA
Enterocloster	0.778	-1.983	1.638	NA
Petrotoga	0.426	0.596	2.150	NA
Wenjunlia	0.648	0.175	2.131	NA
Salinisphaera	0.252	-0.917	0.890	NA
Lewinella	0.693	0.059	0.581	NA
Chloroflexus	0.443	1.418	2.369	NA
Buchananella	0.056	0.003	2.913	NA

Rhodocyclales	0.737	-0.899	2.291	NA
Frisingicoccus	0.052	-0.653	2.913	NA
Pseudanabaena	0.202	1.686	2.909	NA
Effusibacillus	0.123	-1.191	2.911	NA
Blattabacterium	0.399	0.500	2.167	NA
Corticibacterium	0.417	-0.482	2.113	NA
Paramesorhizobium	0.152	0.117	2.912	NA
Betaproteobacterium_AAP99	0.003	0.054	2.914	NA
Oceanotoga	0.000	0.000	0.000	NA
Salmonella_phage_SJ46_virus	0.000	0.000	0.000	NA
Roseibaca	0.208	1.037	2.446	NA
Allofustis	0.129	1.197	2.911	NA
Phialemonium	0.746	0.554	2.020	NA
Thermaerobacter	0.441	0.558	2.406	NA
Phaseolus_vulgaris_endornavirus	0.000	0.000	0.000	NA
Defluviimonas	0.700	1.344	1.549	NA
Syntrophomonas	0.225	0.891	2.909	NA
Paludicola	0.106	1.144	2.911	NA
Kaustia	0.000	0.000	0.000	NA
Pararobbsia	0.255	1.713	2.909	NA
Melittangium	0.241	0.447	2.793	NA
Robinsoniella	0.031	0.361	2.913	NA
