# Supplementary material for: Shotgun Metagenome Analysis of Two Schizaphis graminum Biotypes over Time With and Without Carried Cereal Yellow Dwarf Virus
Source: Insects. 2025 May 23;16(6):554. doi: 10.3390/insects16060554 (PMC12193481; doi:10.3390/insects16060554)
Supplement: Supplementary file 1 [file insects-16-00554-s001.zip › Table S7.pdf]

Table S7. DESeq2 results for comparison by viral carrier status, arranged by log_2 fold change.

Genus	BaseMean	Log2FC	LFCSE	Padj
Meira	37.666	-4.850	0.907	7.304e-04
Aequitasia	23.263	-3.976	0.853	3.809e-03
Moesziomyces	19.389	-3.826	0.814	1.558e-03
Batrachochytrium	21.736	-3.687	1.846	4.426e-01
Emericellopsis	3.316	-3.420	1.363	NA
Pseudactinotalea	0.679	-2.872	2.906	NA
Haematomicrobium	1.237	-2.801	2.091	NA
Dokdonella	1.249	-2.801	1.548	NA
Gemmiger	2.131	-2.774	1.360	NA
Spizellomyces	7.358	-2.710	0.994	1.766e-01
Pilimelia	1.968	-2.616	1.281	NA
Salipaludibacillus	74.463	-2.609	0.479	4.200e-05
Sulfuritalea	0.539	-2.593	2.907	NA
Letharia	101.125	-2.569	0.535	6.544e-04
Turicibacter	9.170	-2.544	1.181	4.106e-01
Chiayiivirga	0.821	-2.537	2.071	NA
Loigolactobacillus	21.543	-2.514	0.964	2.542e-01
Microvirga	228.961	-2.462	0.419	5.661e-06
Gloeophyllum	13.463	-2.460	0.939	2.329e-01
Heyndrickxia	3.581	-2.388	1.347	NA
Neurospora	0.942	-2.374	2.074	NA
Halococcus	1.219	-2.341	0.803	NA
Croceibacterium	2.842	-2.328	1.202	NA
Meyerozyma	1.925	-2.290	1.658	NA
Silanimonas	0.443	-2.261	2.538	NA
Rubrivirga	1.040	-2.252	2.099	NA
Spirilliplanes	0.536	-2.237	2.908	NA
Companilactobacillus	0.662	-2.195	2.283	NA
Sphingomonas-like	0.813	-2.171	1.150	NA
Purpureocillium	0.435	-2.164	2.881	NA
Anaerotardibacter	0.590	-2.115	1.805	NA
Nannizzia	1.520	-2.106	1.221	NA
Desulfotomaculum	0.503	-2.079	2.248	NA
Ilumatobacter	13.317	-2.075	1.095	4.208e-01
Debaryomyces	1.098	-2.057	1.843	NA
Peptococcus	0.662	-2.048	2.517	NA
Tersicoccus	1.189	-2.016	2.147	NA
Alkaliphilus	2.898	-2.002	1.405	NA
Thermogemmata	1.690	-1.992	1.738	NA
Pluralibacter	1.512	-1.991	1.279	NA
Enterocloster	0.778	-1.983	1.638	NA
Qingrenia	0.304	-1.978	2.909	NA
Afifella	1.377	-1.972	1.732	NA
Caenispirillum	0.299	-1.963	2.909	NA
Xanthobacter	8.431	-1.937	0.660	1.132e-01
Lobosporangium	11.067	-1.935	1.107	4.426e-01
Plasticicumulans	0.289	-1.925	2.909	NA
Dankookia	1.052	-1.925	1.367	NA
Mollisia	7.237	-1.914	0.815	2.666e-01
Fimicolochytrium	24.210	-1.904	1.097	4.749e-01
Hydrogenophilus	2.886	-1.863	1.422	NA
UNVERIFIED_CONTAM:	2.089	-1.848	1.200	NA
Roseobacter	0.876	-1.824	1.267	NA
Caldifermentibacillus	6.086	-1.822	1.051	4.426e-01
Dictyostelium	8.462	-1.816	0.778	2.866e-01

Frankineae	1.636	-1.801	2.023	NA
Mongoliimonas	0.271	-1.798	2.909	NA
Polychytrium	14.681	-1.777	0.743	2.666e-01
Microthrix	0.316	-1.773	2.910	NA
Caldilinea	1.616	-1.771	1.729	NA
Variibacter	0.250	-1.764	2.910	NA
Macroventuria	17.481	-1.762	0.475	1.885e-02
Fimbriimonas	4.578	-1.762	1.120	NA
Croceicoccus	0.401	-1.762	1.505	NA
Pelobacter	1.260	-1.743	1.626	NA
Hankyongella	1.915	-1.736	1.892	NA
Chitinophaga	6.927	-1.734	0.569	9.645e-02
Glarea	9.663	-1.734	0.746	2.696e-01
Fenollaria	1.816	-1.722	1.510	NA
Parvibaculum	1.055	-1.706	1.296	NA
Ascochyta	68.305	-1.694	0.491	4.130e-02
Hypoxylon	1.453	-1.693	1.419	NA
Plantactinospora	1.201	-1.692	1.218	NA
Pelagerythrobacter	0.521	-1.691	2.561	NA
Alkalibacterium	1.571	-1.681	1.491	NA
Saccharomyces	2.333	-1.664	1.464	NA
Aceticella	0.332	-1.663	2.910	NA
Myroides	1.389	-1.654	1.740	NA
Thermincola	0.633	-1.654	2.355	NA
Theileria	0.473	-1.631	1.107	NA
Dorea	18.063	-1.625	0.573	1.415e-01
Mumia	1.203	-1.577	1.770	NA
Gemmatirosa	2.401	-1.554	1.545	NA
Limnobaculum	0.794	-1.533	1.085	NA
Motilibacter	2.373	-1.532	1.424	NA
Zasmidium	1.710	-1.531	0.851	NA
Robertmurraya	10.088	-1.531	0.609	2.514e-01
Halophilic	1.709	-1.526	1.442	NA
Diaporthe	5.499	-1.525	1.064	6.866e-01
Nitrososphaera	1.504	-1.519	2.175	NA
Wickerhamomyces	2.456	-1.513	1.487	NA
Aureobasidium	16.662	-1.507	0.604	2.666e-01
Nosocomiicoccus	1.257	-1.506	1.637	NA
Aquirhabdus	0.376	-1.499	2.910	NA
Okeania	8.897	-1.496	0.764	4.106e-01
Moorella	0.192	-1.496	2.910	NA
Larkinella	0.768	-1.494	1.610	NA
Caldibacillus	3.835	-1.486	0.798	NA
Goekera	0.470	-1.476	2.561	NA
Cronobacter	1.817	-1.471	0.926	NA
Tepidiforma	3.376	-1.468	1.412	NA
Coprinopsis	1.018	-1.466	1.550	NA
Melaminivora	2.129	-1.460	1.327	NA
Sphingosinithalassobacter	0.506	-1.452	1.511	NA
Halteromyces	0.737	-1.451	1.584	NA
Congregibacter	0.755	-1.445	0.928	NA
Winkia	10.745	-1.444	0.757	4.208e-01
Rouxiella	0.547	-1.436	1.820	NA
Coprothermobacter	0.362	-1.434	2.908	NA
Chthoniobacter	2.198	-1.433	1.450	NA
Mesomycoplasma	0.567	-1.432	2.071	NA
Coraliihabitans	13.613	-1.432	0.650	3.348e-01
Chloroploca	0.537	-1.426	2.907	NA
Propionibacterium_phage_PHL117M01_virus	0.268	-1.421	2.910	NA

Pinibacter	0.395	-1.414	2.384	NA
Catenulispora	1.103	-1.414	2.334	NA
Stappia	1.931	-1.409	1.287	NA
Chitinophagaceae_genus	0.534	-1.407	2.292	NA
Alterileibacterium	0.250	-1.403	2.911	NA
Domibacillus	6.862	-1.401	1.183	7.142e-01
Rufibacter	1.318	-1.399	1.547	NA
Azotobacter	95.528	-1.390	0.426	6.241e-02
Catellatospora	0.735	-1.363	2.812	NA
Thiofilum	1.021	-1.362	1.960	NA
Frischella	1.199	-1.343	0.871	NA
Nonomuraea	1.439	-1.337	1.143	NA
Pelagivirga	2.684	-1.330	0.604	NA
Methylomicrobium	16.552	-1.323	0.551	2.666e-01
Brochothrix	2.027	-1.321	1.312	NA
Pseudogymnoascus	5.785	-1.321	0.978	6.075e-01
Fibroporia	7.444	-1.319	1.096	7.142e-01
Parasegetibacter	0.335	-1.315	2.910	NA
Herbinix	3.173	-1.312	1.377	NA
Phanerochaete	8.638	-1.310	0.773	4.502e-01
Geodermatophilaceae_genus	1.281	-1.303	1.559	NA
Enterobacteria_phage_vB_EcoS_ACG-M12_virus	1.666	-1.293	1.110	NA
Propioniciclava	26.542	-1.278	0.555	2.866e-01
Moorena	7.922	-1.269	0.826	5.371e-01
Glutamicibacter	45.516	-1.265	0.439	1.284e-01
Collinsella	12.119	-1.263	0.820	5.764e-01
Ilyomonas	0.134	-1.260	2.911	NA
Saliphagus	1.228	-1.259	1.130	NA
Aquihabitans	4.124	-1.251	1.059	NA
Oxalicibacterium	1.142	-1.248	1.397	NA
Pyxidicoccus	1.671	-1.243	1.122	NA
Asanoa	0.412	-1.233	2.908	NA
Fretibacterium	3.170	-1.229	1.076	NA
Jiangella	1.425	-1.229	1.505	NA
Muribaculaceae_genus	0.949	-1.209	1.260	NA
Mariluticola	0.153	-1.209	2.911	NA
Pimelobacter	6.825	-1.201	0.698	4.502e-01
Methylococcus	3.490	-1.198	0.701	NA
Effusibacillus	0.123	-1.191	2.911	NA
Pleurocapsa	1.217	-1.188	1.641	NA
Cyberlindnera	2.022	-1.183	1.214	NA
Paludibacterium	3.956	-1.172	0.753	NA
Pseudorivibacter	1.087	-1.166	1.365	NA
Wickerhamiella	11.493	-1.164	0.729	5.334e-01
Chthonobacter	0.205	-1.156	2.911	NA
Scardovia	0.267	-1.155	2.910	NA
Coprococcus	3.166	-1.151	1.334	NA
Defluviicoccus	0.374	-1.138	2.313	NA
Peptoniphilus	199.711	-1.137	0.514	3.348e-01
Methylovulum	39.553	-1.126	0.463	2.666e-01
Pyrenophora	4.539	-1.123	0.846	NA
Propionibacterium_phage_PAD20_virus	0.688	-1.117	2.904	NA
Guillardia	10.200	-1.115	1.047	7.851e-01
Trichoderma	47.821	-1.115	0.667	4.930e-01
Armatimonas	0.268	-1.113	2.639	NA
Lindgomyces	0.333	-1.103	2.706	NA
Protaetiibacter	2.315	-1.096	1.155	NA
Rickettsiella	1.563	-1.092	0.761	NA
Kinneretia	11.622	-1.084	0.495	3.348e-01

Advenella	2.826	-1.080	1.003	NA
Butyrivibrio	0.850	-1.077	1.751	NA
Stigmatella	0.992	-1.075	2.336	NA
Tepidiphilus	66.163	-1.074	0.603	4.426e-01
Abyssicoccus	5.009	-1.067	1.136	NA
Tolumonas	0.873	-1.064	1.877	NA
Schumannella	0.440	-1.060	2.048	NA
Lamprobacter	0.682	-1.057	1.306	NA
Bipolaris	7.199	-1.051	0.772	6.075e-01
Siccibacter	0.129	-1.049	2.290	NA
Stomatobaculum	7.662	-1.047	0.779	6.229e-01
Frondihabitans	4.024	-1.047	1.306	NA
Cronobacter_phage_vB_CsaM_GAP32_virus	0.325	-1.037	2.624	NA
Knoellia	9.989	-1.034	0.687	5.661e-01
Gayadomonas	2.348	-1.033	0.523	NA
Cryobacterium	1.333	-1.026	1.721	NA
Methyloradius	0.301	-1.025	2.594	NA
Moniliophthora	4.378	-1.023	0.900	NA
Lutimaribacter	14.713	-1.020	0.545	4.223e-01
Weizmannia	1.405	-1.019	2.189	NA
Oribacterium	16.999	-1.018	0.581	4.426e-01
Brenneria	3.075	-1.013	0.400	NA
Daldinia	4.862	-1.012	1.041	NA
Oscillochloris	0.443	-1.008	2.907	NA
Neptunicoccus	0.253	-0.998	2.909	NA
Chroococcidiopsis	2.687	-0.986	0.880	NA
Wallemia	23.482	-0.985	0.935	7.944e-01
Sabulicella	0.126	-0.984	2.911	NA
Flaviaesturariibacter	0.706	-0.983	2.277	NA
Mitsuaria	151.580	-0.982	0.304	6.241e-02
Xanthocytophaga	2.896	-0.981	1.133	NA
Paenacidovorax	3.180	-0.977	0.959	NA
Mameliella	2.131	-0.974	1.110	NA
Demequina	2.940	-0.973	1.053	NA
Filobasidium	18.977	-0.973	0.512	4.223e-01
Chryseomicrobium	0.799	-0.970	1.593	NA
Nocardioidaceae_genus	0.542	-0.955	2.638	NA
Agilicoccus	80.850	-0.954	0.519	4.376e-01
Methyloglobulus	0.637	-0.952	1.429	NA
Cokeromyces	2.224	-0.951	0.475	NA
Ignavibacterium	0.501	-0.951	2.906	NA
Nannocystis	1.346	-0.950	1.752	NA
Auraticoccus	1.587	-0.949	1.998	NA
Agathobacter	7.239	-0.946	1.004	8.432e-01
Mobiluncus	3.137	-0.944	1.024	NA
Coprobacillus	1.834	-0.932	1.198	NA
Globicatella	1.277	-0.929	1.769	NA
Eleftheria	9.525	-0.929	0.618	5.763e-01
Minimicrobia	2.704	-0.923	0.970	NA
Pajaroellobacter	0.393	-0.918	2.693	NA
Salinisphaera	0.252	-0.917	0.890	NA
Gryllotalpicola	6.324	-0.914	1.001	8.432e-01
Asinibacterium	2.381	-0.913	0.905	NA
Robiginitalea	0.271	-0.913	2.654	NA
Bryobacter	0.568	-0.912	2.905	NA
Entamoeba	45.695	-0.912	0.507	4.426e-01
Gulbenkiania	0.803	-0.912	1.773	NA
Craterilacuibacter	127.435	-0.905	0.484	4.223e-01
Didymosphaeria	28.357	-0.904	0.586	5.413e-01

Radiomyces	1.508	-0.902	1.259	NA
Acidimicrobium	0.273	-0.899	2.912	NA
Rhodocyclales	0.737	-0.899	2.291	NA
Inhella	1.220	-0.896	1.577	NA
Tamlana	6.081	-0.893	0.443	3.892e-01
Alloprevotella	42.819	-0.891	0.469	4.223e-01
Vulcaniibacterium	2.155	-0.889	1.571	NA
Clavispora	9.617	-0.889	1.503	9.333e-01
Faecalibacterium	9.066	-0.884	0.805	7.681e-01
Mitosporidium	25.270	-0.877	1.409	9.333e-01
Propionibacterium_phage_SKKY_virus	0.362	-0.876	2.908	NA
Arthromitus	0.148	-0.866	2.912	NA
Flintibacter	0.137	-0.864	2.911	NA
Desulfosporosinus	5.604	-0.860	0.654	6.187e-01
Thiomonas	1.348	-0.860	1.236	NA
Thermohydrogenium	0.891	-0.858	1.973	NA
Rugosimonospora	0.425	-0.855	2.908	NA
Paraphaeosphaeria	0.868	-0.851	2.351	NA
Tepidanaerobacter	0.074	-0.850	2.912	NA
Cryphonectria	1.076	-0.850	1.304	NA
Ustilaginoidea	1.194	-0.847	1.676	NA
Xylophilus	6.954	-0.847	0.503	4.426e-01
Lipomyces	0.225	-0.847	2.756	NA
Nakamurella	30.160	-0.841	0.521	5.162e-01
Paracoccidioides	3.115	-0.839	0.809	NA
Pseudoduganella	5.798	-0.836	0.711	7.262e-01
Herminiimonas	2.008	-0.835	1.285	NA
Paenisporosarcina	0.088	-0.834	2.912	NA
Proteus	179.703	-0.831	0.341	2.666e-01
Gleimia	229.791	-0.830	0.388	3.475e-01
Falsirhodobacter	4.108	-0.829	1.125	NA
Methylosarcina	17.576	-0.828	0.568	5.928e-01
Veillonellaceae_genus	0.948	-0.821	2.015	NA
Pseudocercospora	7.879	-0.818	0.634	6.664e-01
Neoantrodia	9.542	-0.813	0.870	8.432e-01
Arsenicicoccus	2.165	-0.813	0.994	NA
Sparassis	5.100	-0.812	0.749	7.405e-01
Fibrisoma	25.538	-0.812	0.558	5.878e-01
Segetibacter	3.587	-0.811	1.232	NA
Aurantimonas	8.757	-0.807	0.539	5.733e-01
Thermomicrobium	3.852	-0.801	0.844	NA
Tumebacillus	31.849	-0.799	0.378	3.486e-01
Duffyella	4.145	-0.799	0.733	NA
Edwardsiella	64.525	-0.797	0.321	2.569e-01
Oceanimonas	1.818	-0.796	0.653	NA
Amaricoccus	20.347	-0.796	0.552	5.937e-01
Pedobacter	122.644	-0.784	0.258	9.818e-02
Glaciimonas	3.779	-0.777	0.979	NA
Franconibacter	0.683	-0.777	1.241	NA
Peptostreptococcus	6.314	-0.775	0.668	7.142e-01
Desertimonas	10.044	-0.774	0.886	8.763e-01
Polyangium	64.134	-0.774	0.542	6.075e-01
Thiolapillus	14.428	-0.773	0.724	7.851e-01
Thiobacillus	5.354	-0.771	0.762	7.851e-01
Collimonas	7.742	-0.767	0.680	7.361e-01
Nigerium	0.251	-0.766	2.613	NA
Ehrlichia	3.296	-0.760	1.078	NA
Planifilum	0.365	-0.760	2.911	NA
Chrysosporum	14.164	-0.756	0.726	7.944e-01

Sorangium	1.090	-0.755	2.188	NA
Colwellia	7.776	-0.747	0.511	5.792e-01
Viridibacillus	0.463	-0.745	2.026	NA
Dyella	23.540	-0.745	0.342	3.348e-01
Gregarina	0.341	-0.745	2.648	NA
Aureimonas	24.555	-0.744	0.506	5.792e-01
Mangrovibacter	0.437	-0.743	0.648	NA
Paraflavitalea	0.310	-0.741	2.909	NA
Sneathia	1.255	-0.734	2.166	NA
Arenimonas	1.644	-0.731	1.325	NA
Aurantiacibacter	6.751	-0.729	0.791	8.432e-01
Aureispira	3.069	-0.727	0.765	NA
Latilactobacillus	2.108	-0.726	1.309	NA
Pseudaminobacter	1.415	-0.721	1.537	NA
Zygosaccharomyces	0.127	-0.720	2.913	NA
Rhizobiales	18.512	-0.720	0.451	5.334e-01
Musicola	448.106	-0.719	0.349	3.892e-01
Promicromonospora	1.177	-0.718	1.676	NA
Pleurotus	2.254	-0.714	1.091	NA
Arcticibacter	0.736	-0.713	1.364	NA
Moraxellaceae_genus	14.760	-0.711	0.553	6.866e-01
Pseudophaeobacter	4.670	-0.710	0.754	NA
Besnoitia	7.844	-0.709	0.426	4.625e-01
Thalassolituus	1.194	-0.706	1.062	NA
Paenimyroides	6.925	-0.702	0.959	9.656e-01
Elstera	0.783	-0.700	2.170	NA
Catenuloplanes	0.057	-0.698	2.913	NA
Protochlamydia	0.307	-0.697	2.907	NA
Gramella	1.113	-0.695	0.812	NA
Eutypa	3.140	-0.695	0.838	NA
Mangrovibacillus	2.743	-0.693	1.198	NA
Candida	5.852	-0.693	0.619	8.432e-01
Planomonospora	0.303	-0.692	2.335	NA
Pseudooceanicola	63.502	-0.687	0.446	5.413e-01
Sinorhizobium	5.189	-0.687	0.800	8.639e-01
Pararhodobacter	4.208	-0.685	0.758	NA
Longimicrobium	6.592	-0.683	1.014	9.324e-01
Parafrankia	2.012	-0.676	1.070	NA
Methylovorus	3.354	-0.674	0.837	NA
Tetzosporium	0.121	-0.674	2.912	NA
Flavihumibacter	2.570	-0.671	0.828	NA
Alloalcanivorax	2.425	-0.663	0.973	NA
Saccharibacteria	4.408	-0.659	0.987	NA
Frisingicoccus	0.052	-0.653	2.913	NA
Actinomarinicola	1.651	-0.648	1.273	NA
Neohortaea	3.383	-0.647	1.020	NA
Exserohilum	2.273	-0.644	1.267	NA
Marasmius	3.117	-0.639	0.986	NA
Pasteurella	1.606	-0.638	0.635	NA
Chromobacterium	7.322	-0.638	0.507	6.840e-01
Pleomorphomonas	3.840	-0.637	1.123	NA
Humisphaera	4.027	-0.637	1.315	NA
Thermosinus	0.296	-0.634	2.908	NA
Garicola	1.949	-0.634	1.465	NA
Sandaracinus	1.493	-0.632	1.599	NA
Arcobacter	3.620	-0.631	0.820	NA
Yoonia	0.283	-0.631	2.909	NA
Kineosporia	6.745	-0.628	1.016	9.333e-01
Aestuariivirga	0.428	-0.627	2.400	NA

Cadophora	1.997	-0.625	1.122	NA
Propioniferax	0.646	-0.621	2.862	NA
Pseudobdellovibrio	0.385	-0.618	2.039	NA
Thalassobius	5.691	-0.616	0.744	8.823e-01
Sclerotinia	1.417	-0.616	1.265	NA
Phaeodactylum	12.627	-0.615	0.523	7.262e-01
Cecembia	0.302	-0.614	2.210	NA
Proteobacteria	19.452	-0.611	0.643	8.432e-01
Olsenella	7.411	-0.611	0.797	9.019e-01
Agreia	0.629	-0.609	2.006	NA
Megamonas	3.586	-0.606	1.005	NA
Thyridium	3.802	-0.606	1.042	NA
Lichtheimia	16.959	-0.603	0.368	5.046e-01
Extensimonas	3.609	-0.603	1.014	NA
Erythrobacteraceae_genus	1.392	-0.603	1.313	NA
Collibacillus	0.630	-0.601	2.588	NA
Rahnella	196.146	-0.598	0.295	3.892e-01
Pseudoalteromonas	365.269	-0.594	0.197	9.818e-02
Mycosynbacter	1.754	-0.594	1.260	NA
Devosia	74.074	-0.593	0.340	4.426e-01
Catonella	3.258	-0.591	0.923	NA
Faecalicatena	0.820	-0.589	1.509	NA
Plasmopara	6.866	-0.587	0.680	8.703e-01
Granulicella	0.978	-0.585	2.009	NA
Parainfluenza_virus_5	6.913	-0.579	1.136	9.333e-01
Rhizoctonia	10.173	-0.577	0.830	9.324e-01
Paucibacter	116.341	-0.576	0.227	2.454e-01
Endozoicomonas	0.368	-0.575	1.254	NA
Lacticaseibacillus	6.204	-0.574	0.662	8.678e-01
Helcococcus	0.691	-0.573	2.347	NA
Thermoanaerobacterium	17.966	-0.573	0.929	9.333e-01
Hymenobacter	123.295	-0.572	0.325	4.426e-01
Aeribacillus	0.369	-0.569	2.912	NA
Luteitalea	11.215	-0.569	0.944	9.333e-01
Rickettsiales	0.188	-0.565	2.501	NA
Acanthamoeba	49.409	-0.561	0.679	8.970e-01
Halochromatium	4.224	-0.559	0.641	NA
Rhabdothermincola	8.157	-0.557	0.855	9.333e-01
Acidiferrimicrobium	0.646	-0.553	1.633	NA
Qipengyuania	68.427	-0.550	0.422	6.756e-01
Gellertiella	2.945	-0.547	1.511	NA
Lacrimispora	0.872	-0.543	1.734	NA
Erwinia	905.375	-0.543	0.294	4.265e-01
Diolcogaster_facetosa_bracovirus	12.306	-0.540	0.567	8.432e-01
Xenophilus	37.016	-0.538	0.323	4.852e-01
Thioflexithrix	10.033	-0.532	0.448	7.160e-01
Halobacteriovorax	1.982	-0.532	0.585	NA
Lentihominibacter	0.331	-0.530	2.158	NA
Rhodoligotrophos	1.096	-0.529	1.653	NA
Amnibacterium	7.258	-0.529	0.781	9.324e-01
Geodermatophilus	32.121	-0.527	0.535	8.432e-01
Babesia	6.351	-0.522	0.646	8.970e-01
Gemmobacter	16.490	-0.522	0.665	9.020e-01
Drepanopeziza	2.702	-0.522	1.081	NA
Paenarthrobacter	0.311	-0.522	2.653	NA
Phytoactinopolyspora	0.463	-0.521	2.405	NA
Ramlibacter	60.516	-0.515	0.353	5.853e-01
Neomicrococcus	5.484	-0.515	0.688	9.008e-01
Dacryopinax	2.313	-0.513	1.329	NA

Armatimonadetes	144.045	-0.512	0.645	9.019e-01
Rugamonas	25.020	-0.509	0.414	7.142e-01
Actinoallomurus	1.044	-0.506	1.615	NA
Porphyromonas	64.376	-0.505	0.387	6.781e-01
Hyalangium	1.398	-0.505	2.052	NA
Saccharimonas	0.553	-0.504	1.958	NA
Mogibacterium	9.735	-0.503	0.629	9.008e-01
Thermicanus	28.314	-0.501	0.947	9.372e-01
Haemophilus	217.469	-0.499	0.270	4.239e-01
Alicycliphilus	12.250	-0.497	0.447	7.681e-01
Dermabacter	7.786	-0.493	0.926	9.333e-01
Caldimonas	5.324	-0.493	0.852	9.333e-01
Roseitranquillus	0.614	-0.491	2.088	NA
Tetrahymena	5.684	-0.490	0.666	9.020e-01
Marmoricola	34.294	-0.488	0.543	8.703e-01
Dothidotthia	2.037	-0.488	1.212	NA
Acaricomes	2.228	-0.487	0.852	NA
Thermalbibacter	6.902	-0.487	1.011	9.388e-01
Desarmillaria	3.626	-0.485	0.809	NA
Faecalibacillus	0.089	-0.484	2.913	NA
Corticibacterium	0.417	-0.482	2.113	NA
Rhizorhabdus	68.045	-0.481	0.291	4.930e-01
Huaxiibacter	3.774	-0.481	0.921	NA
Streptacidiphilus	2.022	-0.481	1.515	NA
Phormidium	0.945	-0.480	1.500	NA
Naumannella	5.114	-0.479	0.848	9.333e-01
Methanothermobacter	0.156	-0.478	2.910	NA
Mycoplasmopsis	1.529	-0.478	1.103	NA
Loktanella	12.504	-0.478	0.258	4.223e-01
Methylibium	9.563	-0.478	0.828	9.333e-01
Labilithrix	4.216	-0.478	1.035	NA
Coniosporium	2.486	-0.476	1.446	NA
Anaerobacillus	0.144	-0.476	2.910	NA
Morococcus	313.297	-0.474	0.270	4.426e-01
Actibacterium	1.342	-0.471	1.295	NA
Vallicoccus	3.729	-0.470	1.249	NA
Methylocella	3.834	-0.470	1.246	NA
Thermopolyspora	0.034	-0.470	2.913	NA
Oligoflexus	0.666	-0.467	2.902	NA
Polymorphobacter	8.366	-0.466	0.841	9.519e-01
Hominisplanchenecus	0.033	-0.463	2.913	NA
Phocaeicola	15.829	-0.461	0.616	9.176e-01
Roseburia	27.841	-0.460	0.256	4.426e-01
Pectobacterium	2.621	-0.459	0.530	NA
Serratia	233.668	-0.457	0.246	4.223e-01
Verticiella	0.592	-0.455	2.351	NA
Branchiibius	0.731	-0.454	2.318	NA
Fonsecaea	17.304	-0.453	0.585	9.058e-01
Paraconexibacter	1.837	-0.453	1.110	NA
Methylocapsa	1.039	-0.452	1.167	NA
Allomeiothermus	372.615	-0.451	0.599	9.105e-01
Acidiphilium	0.973	-0.448	1.459	NA
Alloscardovia	67.926	-0.447	0.419	7.851e-01
Pseudosporangium	0.338	-0.444	2.910	NA
Anaeromassilibacillus	0.075	-0.443	2.912	NA
Calidithermus	19.574	-0.443	0.537	8.970e-01
Aaosphaeria	0.407	-0.440	2.309	NA
Marinobacterium	1297.016	-0.437	0.358	7.142e-01
Pseudenterobacter	0.515	-0.433	0.530	NA

Noviherbaspirillum	23.982	-0.426	0.684	9.333e-01
Lasiodiplodia	27.457	-0.424	0.350	7.152e-01
Methyloversatilis	103.042	-0.424	0.273	5.413e-01
Lachnospira	17.322	-0.421	0.461	8.678e-01
TM7	7.135	-0.421	0.646	9.333e-01
Thalassotalea	1.950	-0.421	0.945	NA
Acidothermus	1.557	-0.419	1.622	NA
Pararhizobium	9.293	-0.418	0.649	9.333e-01
Galbitalea	3.500	-0.413	1.191	NA
Occultella	0.037	-0.412	2.913	NA
Roseisolibacter	3.912	-0.412	1.083	NA
Salinispora	1.822	-0.409	1.403	NA
Microsporum	3.527	-0.402	1.039	NA
Gaiella	11.733	-0.401	0.965	9.577e-01
Luteimonas	79.273	-0.399	0.533	9.133e-01
Gordonia	74.952	-0.398	0.358	7.681e-01
Plesiocystis	0.041	-0.397	2.913	NA
Thermobacillus	0.214	-0.397	2.910	NA
Dactylosporangium	0.221	-0.393	2.909	NA
Micropruina	6.588	-0.392	0.968	9.577e-01
Mannheimia	5.616	-0.391	0.526	9.020e-01
Saprolegnia	7.421	-0.391	0.574	9.324e-01
Reticulibacter	0.388	-0.390	2.906	NA
Corallococcus	123.324	-0.389	0.264	5.792e-01
Fortiea	2.790	-0.389	1.287	NA
Jatrophihabitans	6.706	-0.389	1.031	9.590e-01
Rhodoplanes	8.916	-0.388	0.554	9.324e-01
Varibaculum	3.474	-0.384	0.970	NA
Wolbachia	68.134	-0.384	0.405	8.432e-01
Porphyromonadaceae_genus	12.235	-0.383	0.624	9.333e-01
Marinithermofilum	29.006	-0.382	0.261	5.792e-01
Duganella	51.520	-0.377	0.337	7.680e-01
Modestobacter	94.398	-0.375	0.309	7.142e-01
Schaalia	24.329	-0.373	0.462	9.003e-01
Geomicrobium	0.127	-0.372	2.911	NA
Rubrobacter	16.449	-0.369	0.634	9.333e-01
Gallibacter	16.369	-0.368	0.814	9.519e-01
Kallotenue	98.738	-0.367	0.578	9.333e-01
Xanthomonadaceae_genus	3.912	-0.365	0.866	NA
Arachnia	16.113	-0.365	0.550	9.333e-01
Akanthomyces	0.281	-0.364	2.908	NA
Actinosynnema	3.106	-0.363	0.847	NA
Acaromyces	20.247	-0.361	0.877	9.577e-01
Gilbertella	13763.122	-0.359	0.231	5.413e-01
Kickxella	0.434	-0.358	1.982	NA
Limobrevibacterium	0.486	-0.358	1.738	NA
Oryzomicrobium	63.319	-0.358	0.413	8.842e-01
Glaciibacter	18.801	-0.357	0.655	9.333e-01
Nitrobacter	5.995	-0.356	0.657	9.333e-01
Aequorivita	187.348	-0.354	0.190	4.223e-01
Lactovum	3.125	-0.354	1.066	NA
Lamprocystis	7995.423	-0.354	0.329	7.851e-01
Mariprofundus	0.047	-0.352	2.913	NA
Secondary	3.884	-0.351	0.457	NA
Algoriphagus	22.246	-0.351	0.330	7.851e-01
Nitrosomonas	5.354	-0.348	0.802	9.497e-01
Citrobacter	14367.687	-0.347	0.289	7.172e-01
Lonsdalea	0.189	-0.346	0.901	NA
Thermosipho	4.351	-0.346	0.418	NA

Pelosinus	4.274	-0.344	0.863	NA
Thermus	128.332	-0.344	0.408	8.970e-01
Nocardiopsis	6.413	-0.343	0.968	9.614e-01
Bavariicoccus	65.404	-0.342	0.323	7.859e-01
Oscillatoria	1.378	-0.341	1.151	NA
Peptostreptococcaceae_genus	4.076	-0.338	0.879	NA
Herpetosiphon	1.350	-0.338	1.637	NA
Diaphorobacter	35.714	-0.337	0.378	8.703e-01
Xylella	2.546	-0.337	0.607	NA
Fomitiporia	7.709	-0.335	0.771	9.577e-01
Enterobacter	9658.205	-0.333	0.287	7.387e-01
Megasphaera	17.752	-0.332	0.670	9.396e-01
Calorimonas	0.022	-0.332	2.913	NA
Torulaspora	0.428	-0.331	2.905	NA
Aedoeadaptatus	2.659	-0.330	1.330	NA
Gardnerella	33.910	-0.329	0.616	9.333e-01
Finegoldia	105.875	-0.328	0.551	9.333e-01
Pinisolibacter	11.572	-0.328	0.277	6.866e-01
Rhodococcus	464.902	-0.327	0.180	4.421e-01
Simonsiella	0.622	-0.326	2.073	NA
Oleiphilus	1.965	-0.323	0.759	NA
Streptomyces	1840.873	-0.323	0.120	1.766e-01
Plantibacter	4.413	-0.322	0.960	NA
Insolitispirillum	2.216	-0.321	1.122	NA
Fastidiosipila	0.389	-0.319	2.910	NA
Pusillimonas	1729.460	-0.318	0.258	7.142e-01
Curtobacterium	171.096	-0.318	0.383	8.970e-01
Stenoxybacter	0.289	-0.316	2.908	NA
Microbacterium	1220.119	-0.316	0.134	2.666e-01
Actinomadura	901.640	-0.314	0.148	3.475e-01
Yersinia	118.324	-0.314	0.384	8.997e-01
Gluconacetobacter	6.748	-0.314	0.573	9.333e-01
Fervidobacterium	0.190	-0.313	2.909	NA
Thermodesulfomicrobium	0.020	-0.312	2.913	NA
Leifsonia	76.614	-0.311	0.380	8.997e-01
Roseateles	324.440	-0.309	0.171	4.426e-01
Ichthyophthirius	11.762	-0.309	0.508	9.333e-01
Rhizorhapis	20.990	-0.306	0.462	9.333e-01
Perkinsus	5.227	-0.303	0.583	9.333e-01
Providencia	883.343	-0.303	0.156	4.135e-01
Actinomycetospora	19.821	-0.301	0.534	9.333e-01
Dongia	0.909	-0.300	1.667	NA
Mitsuokella	0.467	-0.299	2.688	NA
Apibacter	4.693	-0.298	0.995	NA
Haematobacter	4.763	-0.298	1.196	NA
Acetobacterium	3.427	-0.297	0.956	NA
Propionibacterium_phage_PHL301M00_virus	0.111	-0.294	2.911	NA
Morchella	0.641	-0.294	2.384	NA
Actinotalea	12.237	-0.294	0.672	9.577e-01
Prevotella	338.175	-0.294	0.303	8.432e-01
Pseudomicrostroma	29.079	-0.291	0.853	9.656e-01
Pestalotiopsis	18.985	-0.290	0.520	9.333e-01
Lentibacillus	34.801	-0.287	0.263	7.789e-01
Acytostelium	0.807	-0.287	0.832	NA
Drancourtella	0.294	-0.287	2.673	NA
Puia	26.661	-0.286	0.295	8.432e-01
Hephaestia	2.393	-0.286	1.200	NA
Linderina	1.155	-0.285	1.070	NA
Grimontia	0.335	-0.285	0.781	NA

Yaniella	1.655	-0.284	1.248	NA
Parasaccharibacter	82.051	-0.283	0.213	6.544e-01
Bacillus	2979.064	-0.282	0.116	2.666e-01
Aestuariibaculum	1.315	-0.281	1.146	NA
Klenkia	7.551	-0.280	0.724	9.656e-01
Tetrasphaera	10.317	-0.278	0.666	9.577e-01
Parastagonospora	59.095	-0.277	0.422	9.333e-01
Hansschlegelia	2.156	-0.274	1.357	NA
Acidisoma	1.255	-0.272	1.486	NA
Thermomonas	34.874	-0.272	0.261	7.944e-01
Novosphingobium	521.937	-0.271	0.292	8.533e-01
Intrasporangiaceae_genus	0.940	-0.270	1.266	NA
Hydrocarboniphaga	1.904	-0.270	1.185	NA
Candidate	14.431	-0.269	0.352	9.072e-01
Falcatimonas	0.017	-0.267	2.913	NA
Marichromatium	2.010	-0.264	0.587	NA
Cercospora	12.008	-0.264	0.542	9.396e-01
Pseudomonas	20853.250	-0.262	0.207	6.899e-01
Solimonas	5.862	-0.261	0.869	9.737e-01
Cedecea	62.532	-0.261	0.403	9.333e-01
Adlercreutzia	0.609	-0.261	1.882	NA
Naegleria	20.303	-0.259	0.490	9.333e-01
Vescimonas	0.025	-0.259	2.913	NA
Pseudochrobactrum	75.819	-0.259	0.452	9.333e-01
Yinghuangia	0.547	-0.258	1.527	NA
Epithele	1.083	-0.258	1.176	NA
Salinibacterium	8.132	-0.257	0.621	9.562e-01
Pseudonocardia	81.981	-0.255	0.385	9.333e-01
Penicillium	43.107	-0.255	0.361	9.324e-01
Kribbella	6.342	-0.254	0.565	9.519e-01
Winslowiella	0.065	-0.253	2.913	NA
Aspergillus	77.673	-0.251	0.265	8.432e-01
Hahella	2.380	-0.251	0.743	NA
Hoyosella	0.809	-0.250	2.371	NA
Labilibacter	4.141	-0.250	0.840	NA
Halorubrum	12.131	-0.248	0.426	9.333e-01
Peptoclostridium	0.886	-0.248	1.730	NA
Umbelopsis	0.027	-0.247	2.913	NA
Propionicimonas	2.047	-0.246	1.065	NA
Sphingopyxis	66.886	-0.246	0.395	9.333e-01
Haloferax	0.284	-0.246	2.685	NA
Oceanicola	3.328	-0.243	1.041	NA
Empedobacter	31.761	-0.241	0.370	9.333e-01
Microvirgula	1.737	-0.240	1.610	NA
Sutterella	3.974	-0.238	0.614	NA
Atlantibacter	14.246	-0.237	0.266	8.709e-01
Sandarakinorhabdus	1.376	-0.237	1.901	NA
Nanosynbacter	12.801	-0.237	0.636	9.638e-01
Mycobacteroides	34.731	-0.237	0.648	9.656e-01
Phytobacter	11.988	-0.236	0.450	9.372e-01
Chloroflexia	0.169	-0.236	2.910	NA
Terrimonas	37.719	-0.235	0.482	9.439e-01
Trematosphaeria	5.185	-0.233	1.017	9.737e-01
Cyclobacterium	9.163	-0.233	0.364	9.218e-01
Wenzhouxiangella	3.178	-0.233	0.815	NA
Meiothermus	30.189	-0.231	0.561	9.577e-01
Variovorax	87.909	-0.231	0.197	7.356e-01
Propylenella	0.014	-0.231	2.914	NA
Polaribacter	11.410	-0.229	0.444	9.378e-01

Ruminococcaceae_genus	14.329	-0.228	0.374	9.333e-01
Anoxybacillus	29.382	-0.226	0.644	9.656e-01
Blastomyces	1.591	-0.225	0.866	NA
Comamonadaceae_genus	99.630	-0.224	0.238	8.432e-01
Flavimobilis	2.525	-0.222	1.064	NA
Terrihabitans	1.488	-0.222	1.911	NA
Sanguibacter	2.434	-0.221	1.229	NA
Kuraishia	0.066	-0.219	2.913	NA
Elioraea	0.452	-0.218	2.025	NA
Methylomonas	1.557	-0.218	0.577	NA
Rubrivivax	73.057	-0.216	0.340	9.333e-01
Sphingomonas	2004.426	-0.216	0.128	4.749e-01
Simian_virus_40	1.059	-0.214	0.771	NA
Mucilaginibacter	56.373	-0.213	0.299	9.324e-01
Conyzicola	1.722	-0.211	1.213	NA
Oerskovia	0.479	-0.210	2.525	NA
Clostridiales	35.068	-0.209	0.385	9.333e-01
Parabacteroides	28.004	-0.207	0.341	9.333e-01
Sporosarcina	3.898	-0.207	0.743	NA
Toxoplasma	250.863	-0.207	0.150	6.229e-01
Filifactor	2.916	-0.206	0.995	NA
Diplodia	4.466	-0.204	0.826	NA
Neisseria	439.766	-0.204	0.210	8.432e-01
Tetragenococcus	9.450	-0.203	0.873	9.737e-01
Nitrotoga	4.783	-0.202	0.575	NA
Alkalicoccobacillus	0.836	-0.202	0.760	NA
Rhodomicrobium	7.805	-0.201	0.459	9.577e-01
Mesobacillus	1.096	-0.201	1.371	NA
Flexivirga	2.311	-0.199	1.100	NA
Mycena	39.124	-0.199	0.827	9.737e-01
Fluviicola	6.799	-0.196	0.839	9.737e-01
Pyrococcus	12.784	-0.196	0.576	9.656e-01
Vibrionimonas	0.863	-0.191	1.858	NA
Solobacterium	7.141	-0.190	0.677	9.673e-01
Lactobacillus	101.572	-0.190	0.199	8.432e-01
Aplosporella	8.752	-0.189	0.774	9.737e-01
Paludifilum	42.859	-0.189	0.224	8.970e-01
Sphingosinicella	7.978	-0.183	0.779	9.737e-01
Pseudorhodoferax	8.912	-0.182	0.650	9.737e-01
Oceanospirillum	4.513	-0.181	0.622	NA
Paucilactobacillus	8.021	-0.181	0.553	9.656e-01
Cereibacter	5.029	-0.180	0.786	9.737e-01
Sphaerobacter	6.365	-0.180	1.178	9.779e-01
Sporolactobacillus	110.272	-0.180	0.159	7.535e-01
Viridilinea	0.240	-0.179	2.911	NA
Puccinia	4.914	-0.179	0.786	NA
Geoalkalibacter	0.121	-0.179	2.913	NA
Salipiger	55.657	-0.178	0.294	9.333e-01
Paramagnetospirillum	1.385	-0.178	1.446	NA
Desulfitobacterium	6.033	-0.178	0.776	9.737e-01
Desemzia	16.346	-0.177	0.511	9.656e-01
Leptothrix	258.760	-0.177	0.333	9.333e-01
Podospora	0.781	-0.175	0.807	NA
Caballeronia	39.742	-0.173	0.229	9.089e-01
Zhihengliuella	1.833	-0.173	1.499	NA
Lentzea	21.126	-0.173	0.300	9.333e-01
Mycobacteriaceae_genus	41.727	-0.171	0.227	9.020e-01
Rhizobiaceae_genus	0.018	-0.168	2.913	NA
Pectinatus	4.713	-0.166	0.697	NA

Oceanibium	5.401	-0.166	0.654	9.705e-01
Salmonella	451.456	-0.163	0.161	8.128e-01
Leuconostoc	27.068	-0.161	0.320	9.395e-01
Cellvibrio	6.798	-0.158	0.730	9.776e-01
Spirosoma	11.388	-0.158	0.612	9.737e-01
Pelomonas	2109.665	-0.158	0.179	8.763e-01
Hassallia	0.102	-0.158	2.914	NA
Formosimonas	0.082	-0.158	2.914	NA
Endosymbiont	5.833	-0.157	0.580	9.670e-01
Enterobacteria_phage_phi80_virus	4.380	-0.157	0.881	NA
Rhodobacteraceae_genus	44.452	-0.156	0.474	9.656e-01
Phytophthora	90.681	-0.156	0.427	9.656e-01
Rhizopus	1.208	-0.156	1.506	NA
Paracoccus	735.536	-0.155	0.172	8.678e-01
Motilimonas	7.460	-0.153	0.535	9.656e-01
Jaminaea	0.197	-0.152	2.910	NA
Burkholderiaceae_genus	42.128	-0.150	0.339	9.519e-01
Shimia	358.445	-0.149	0.301	9.396e-01
Elizabethkingia	205.340	-0.149	0.269	9.333e-01
Jeotgalicoccus	20.422	-0.148	0.374	9.577e-01
Methanothrix	0.009	-0.148	2.914	NA
Marinobacter	150.235	-0.146	0.245	9.333e-01
Pelagibacterium	3.573	-0.145	1.111	NA
Kordiimonas	0.472	-0.143	2.119	NA
Jeotgalibacillus	1.068	-0.142	1.070	NA
Dissoconium	0.190	-0.141	2.911	NA
Falsiroseomonas	137.429	-0.140	0.254	9.333e-01
Usitatibacter	0.008	-0.140	2.914	NA
Schizophyllum	63.317	-0.140	0.886	9.779e-01
Halovibrio	10.136	-0.138	0.623	9.737e-01
Ruania	2.191	-0.137	1.104	NA
Kosakonia	17.606	-0.137	0.335	9.577e-01
Levilactobacillus	89.361	-0.136	0.472	9.737e-01
Chromohalobacter	65.014	-0.136	0.210	9.333e-01
Macromonas	0.175	-0.136	2.909	NA
Methylorubrum	226.898	-0.135	0.170	9.008e-01
Chromatium	57.450	-0.135	0.208	9.333e-01
Agrobacterium	192.449	-0.135	0.190	9.218e-01
Halalkalibacterium	3.247	-0.133	0.877	NA
Bathymodiolus	0.021	-0.132	2.913	NA
Fonticella	0.702	-0.131	2.368	NA
Ruoffia	0.404	-0.131	1.986	NA
Coniophora	9.959	-0.130	1.023	9.785e-01
Actinoplanes	35.115	-0.130	0.366	9.656e-01
Bhargavaea	9.142	-0.128	0.600	9.737e-01
Angustibacter	3.966	-0.127	1.148	NA
Allosphingosinicella	0.640	-0.126	1.559	NA
Methylobacterium	5697.290	-0.125	0.183	9.333e-01
Salinicola	3.874	-0.123	0.551	NA
Mediterraneibacter	9.994	-0.121	0.429	9.577e-01
Cupidesulfovibrio	28.579	-0.120	0.402	9.705e-01
Planococcus	196.283	-0.120	0.195	9.333e-01
Pseudodesulfovibrio	0.057	-0.119	2.914	NA
Neorhizobium	3.954	-0.118	0.974	NA
Sinomonas	0.642	-0.117	2.736	NA
Trichosporon	103.764	-0.117	0.718	9.776e-01
Aureibaculum	51.891	-0.116	0.370	9.670e-01
Solemya	6.351	-0.115	0.672	9.776e-01
Minicystis	1.137	-0.114	1.776	NA

Oceaniovalibus	4.526	-0.113	0.713	NA
Myxococcus	31.942	-0.113	0.261	9.378e-01
Sediminibacterium	2.811	-0.111	1.148	NA
Histoplasma	0.752	-0.110	1.228	NA
Bacteroides	124.758	-0.108	0.232	9.497e-01
Suillus	20.978	-0.108	0.933	9.785e-01
Fictibacillus	317.200	-0.106	0.347	9.706e-01
Sphingomonadaceae_genus	2.729	-0.106	1.420	NA
Herbaspirillum	591.387	-0.105	0.203	9.378e-01
Bacteria	0.118	-0.105	0.928	NA
Fluoribacter	203.940	-0.105	0.274	9.614e-01
Tuber	2.943	-0.104	1.243	NA
Rhodovulum	0.928	-0.101	1.325	NA
Kushneria	83.057	-0.101	0.276	9.590e-01
Polysphondylium	0.471	-0.101	2.521	NA
Algibacter	5.117	-0.100	0.550	9.776e-01
Fusarium	142.957	-0.100	0.575	9.776e-01
Methylobrevis	19.272	-0.099	0.412	9.717e-01
Microcella	4.645	-0.098	0.851	NA
Lachnoclostridium	1.068	-0.098	2.188	NA
Beggiatoa	32.646	-0.097	0.243	9.577e-01
Flavisolibacter	0.705	-0.097	1.200	NA
Afipia	1012.669	-0.096	0.244	9.590e-01
Hemiselmis	0.919	-0.096	1.728	NA
Streptosporangium	49.775	-0.095	0.237	9.577e-01
Ideonella	12.997	-0.095	0.561	9.776e-01
Leclercia	50.086	-0.095	0.234	9.577e-01
Micromonospora	101.944	-0.093	0.441	9.776e-01
Facklamia	25.140	-0.091	0.354	9.724e-01
Rhodanobacter	6.156	-0.089	0.631	9.785e-01
Deinococcus	166.493	-0.089	0.155	9.333e-01
Aquamicrobium	62.955	-0.089	0.335	9.737e-01
Mycetohabitans	9.753	-0.088	0.450	9.670e-01
Enterobacteriaceae_genus	24.824	-0.087	0.286	9.591e-01
Hafnia	2.018	-0.087	0.470	NA
Alloiococcus	1.164	-0.086	1.293	NA
Dioszegia	17.319	-0.084	0.940	9.785e-01
Amniculibacterium	0.863	-0.084	2.889	NA
Neobacillus	44.891	-0.084	0.242	9.656e-01
Atopomonas	30.225	-0.081	0.280	9.656e-01
Allomuricauda	54.976	-0.080	0.344	9.737e-01
Asticcacaulis	114.652	-0.080	0.404	9.776e-01
Enterobacteria_phage_f1_virus	0.008	-0.080	2.914	NA
Photodesmus	0.007	-0.080	2.914	NA
Doolittlea	0.005	-0.080	2.914	NA
Arthroderma	0.451	-0.080	1.192	NA
Gilliamella	0.563	-0.079	1.188	NA
Endocarpon	5.929	-0.079	0.659	9.785e-01
Paludisphaera	3.132	-0.078	0.840	NA
Mycoplana	0.055	-0.076	2.914	NA
Caulobacter	1070.743	-0.076	0.237	9.670e-01
Pauljensenia	155.649	-0.075	0.436	9.776e-01
Zavarzinella	0.480	-0.072	2.687	NA
Carideicomes	0.567	-0.071	1.149	NA
Drechmeria	0.424	-0.070	1.699	NA
Anaeroglobus	4.318	-0.069	0.776	NA
Ancylomarina	7.570	-0.068	0.560	9.776e-01
Lactiplantibacillus	4.188	-0.066	0.534	NA
Raoultella	68.520	-0.065	0.319	9.776e-01

Frigoribacterium	15.155	-0.065	0.630	9.785e-01
Lysinibacillus	127.976	-0.061	0.370	9.776e-01
Limosilactobacillus	14.701	-0.061	0.434	9.776e-01
Klebsiella	1531.102	-0.060	0.227	9.737e-01
Kalmanozyma	5.083	-0.059	0.825	9.785e-01
Curvibacter	191.180	-0.058	0.148	9.590e-01
Laccaria	4.694	-0.056	0.747	NA
Kurthia	83.993	-0.054	0.348	9.779e-01
Auritidibacter	1.179	-0.054	2.789	NA
Azospira	27.304	-0.053	0.312	9.737e-01
Aeromonas	84.822	-0.051	0.227	9.737e-01
Actinobacteria	2.223	-0.050	1.630	NA
Eubacterium	18.755	-0.049	0.399	9.785e-01
Paraburkholderia	685.653	-0.048	0.161	9.706e-01
Sphaerotilus	3.359	-0.048	0.962	NA
Methylosinus	0.957	-0.046	1.081	NA
Puteibacter	1.005	-0.046	1.139	NA
Geminicoccus	65.368	-0.045	0.232	9.737e-01
Chloroflexi	0.505	-0.044	1.490	NA
Nocardia	68.412	-0.044	0.226	9.776e-01
Zymoseptoria	34.389	-0.043	0.366	9.785e-01
Leptospira	69.642	-0.043	0.277	9.776e-01
Evansella	0.869	-0.043	2.113	NA
Enterobacteria_phage_T4_virus	2.981	-0.042	1.117	NA
Tepidimonas	39.113	-0.041	0.497	9.785e-01
Sugiyamaella	3.235	-0.040	1.243	NA
Paraflavisolibacter	0.061	-0.038	2.914	NA
Labilibaculum	7.087	-0.036	0.489	9.776e-01
Argonema	0.623	-0.035	2.156	NA
Sinirhodobacter	15.801	-0.035	0.331	8.970e-01
Baumannia	0.002	-0.034	2.914	NA
Weissella	5.780	-0.034	0.791	9.785e-01
Frankia	114.940	-0.034	0.284	9.785e-01
Rummeliibacillus	3.322	-0.033	0.831	NA
Capnocytophaga	41.728	-0.029	0.303	9.785e-01
Micrococcus	532.483	-0.029	0.273	9.785e-01
Salifodinibacter	1.550	-0.026	1.707	NA
Atopobium	11.220	-0.026	0.646	9.799e-01
Pantoea	228.056	-0.024	0.146	9.776e-01
Bacidia	3.842	-0.024	0.899	NA
Sporisorium	1.195	-0.023	1.312	NA
Leucothrix	10.440	-0.023	0.474	9.785e-01
Microbispora	62.361	-0.023	0.302	9.785e-01
Streptococcus	1476.802	-0.022	0.295	9.785e-01
Schlegelella	25.036	-0.022	0.436	9.820e-01
Pleomorpha	3.572	-0.021	0.885	NA
Lachnellula	3.432	-0.021	0.797	NA
Thermorudis	0.331	-0.021	2.759	NA
Erythrobacter	135.550	-0.020	0.227	9.776e-01
Comamonas	377.215	-0.020	0.215	9.785e-01
Chelativorans	10.320	-0.020	0.809	9.880e-01
Dichomitus	29.347	-0.020	0.689	9.880e-01
Rhodobacter	87.074	-0.019	0.250	9.785e-01
Epilithonimonas	41.936	-0.017	0.362	9.785e-01
Actinoalloteichus	0.689	-0.016	1.343	NA
Leucobacter	21.363	-0.015	0.397	9.807e-01
Hyphomonas	1.185	-0.015	1.689	NA
Ectobacillus	119.344	-0.015	0.355	9.799e-01
Allobacillus	31.886	-0.014	0.362	9.785e-01

Rothia	240.951	-0.014	0.257	9.807e-01
Aliarcobacter	1.118	-0.013	2.346	NA
Cloacibacterium	73.077	-0.009	0.419	9.835e-01
Betaproteobacterium_FWI2	1603.185	-0.008	0.339	9.885e-01
Anabaena	1.596	-0.005	1.203	NA
Marinifilum	38.998	-0.005	0.259	9.849e-01
Kineococcus	53.880	-0.004	0.675	9.880e-01
Aphanothece	13.799	-0.004	0.427	9.917e-01
Penaeicola	2.047	-0.001	0.899	NA
Acidovorax	744.728	-0.000	0.109	9.785e-01
Mikella	0.000	0.000	0.000	NA
Ishikawaella	0.000	0.000	0.000	NA
Hafniaceae_genus	0.000	0.000	0.000	NA
Shigella_phage_SfIV_virus	0.000	0.000	0.000	NA
Mediannikoviicoccus	0.000	0.000	0.000	NA
Escherichia_phage_500465-1_virus	0.000	0.000	0.000	NA
Enterobacteria_phage_DE3_virus	0.000	0.000	0.000	NA
Enterobacteria_phage_P7_virus	0.000	0.000	0.000	NA
Escherichia_phage_RCS47_virus	0.000	0.000	0.000	NA
Lagierella	0.000	0.000	0.000	NA
Rhabdobacter	0.000	0.000	0.000	NA
Kallipyga	0.000	0.000	0.000	NA
Escherichia_phage_500465-2_virus	0.000	0.000	0.000	NA
Escherichia_virus_Lambda_2G7b	0.000	0.000	0.000	NA
Escherichia_phage_TL-2011b_virus	0.000	0.000	0.000	NA
Escherichia_virus_Lambda_4A7	0.000	0.000	0.000	NA
Pusillibacter	0.000	0.000	0.000	NA
Escherichia_phage_Lambda_ev099_virus	0.000	0.000	0.000	NA
Klebsiella_phage_4_virus	0.000	0.000	0.000	NA
Stx2-converting_phage_1717_virus	0.000	0.000	0.000	NA
Cetobacterium	0.000	0.000	0.000	NA
Escherichia_phage_Cartapus_virus	0.000	0.000	0.000	NA
SsRNA_phage_SRR5466337_3_virus	0.000	0.000	0.000	NA
Escherichia_phage_Lambda_ev207_virus	0.000	0.000	0.000	NA
Escherichia_virus_Lambda_1H12	0.000	0.000	0.000	NA
Tropicibacter	0.000	0.000	0.000	NA
Stx2-converting_phage_Stx2a_WGPS2_virus	0.000	0.000	0.000	NA
Escherichia_phage_Lambda_ev243_virus	0.000	0.000	0.000	NA
Couchioplanes	0.000	0.000	0.000	NA
Escherichia_virus_Lambda_2H10	0.000	0.000	0.000	NA
Thermobrachium	0.000	0.000	0.000	NA
Deferrisoma	0.000	0.000	0.000	NA
Escherichia_phage_D6_virus	0.000	0.000	0.000	NA
Enterobacteria_phage_Sf6_virus	0.000	0.000	0.000	NA
Gloeothece	0.000	0.000	0.000	NA
Escherichia_phage_520873_virus	0.000	0.000	0.000	NA
Viadribacter	0.000	0.000	0.000	NA
Oceanotoga	0.000	0.000	0.000	NA
Salmonella_phage_SJ46_virus	0.000	0.000	0.000	NA
Phaseolus_vulgaris_endornavirus	0.000	0.000	0.000	NA
Kaustia	0.000	0.000	0.000	NA
Aeromicrobium	36.608	0.001	0.347	1.000e+00
Paeniglutamicibacter	17.218	0.001	0.374	9.745e-01
Ruegeria	9.018	0.002	0.690	9.785e-01
Buchananella	0.056	0.003	2.913	NA
Ancylobacter	5.043	0.004	0.939	1.000e+00
Sphingobacterium	129.321	0.004	0.308	9.880e-01
Citromicrobium	6.385	0.004	0.826	9.880e-01
Psychromicrobium	11.336	0.005	0.299	9.776e-01

Phytoplasma	68.366	0.008	0.312	9.880e-01
Rhizobium	451.252	0.010	0.162	9.785e-01
Alternaria	98.393	0.012	0.229	9.815e-01
Actinobacillus	273.019	0.013	0.342	9.849e-01
Acinetobacter	11818.492	0.014	0.154	9.785e-01
Lentisphaera	29.992	0.015	0.267	9.780e-01
Fimbriiglobus	2.293	0.015	1.247	NA
Plasmodium	260.330	0.015	0.167	9.785e-01
Bradyrhizobium	4376.605	0.017	0.178	9.785e-01
Burkholderia	2610.408	0.018	0.152	9.785e-01
Pseudokineococcus	5.519	0.019	1.241	1.000e+00
Methylophilus	8.689	0.019	0.539	9.785e-01
Flavobacterium	706.963	0.021	0.155	9.785e-01
Conchiformibius	7.052	0.021	0.789	1.000e+00
Parasphingopyxis	15.233	0.022	0.635	9.785e-01
Heterobasidion	20.601	0.022	0.846	9.880e-01
Tachikawaea	0.009	0.023	2.914	NA
Pirellula	0.574	0.024	1.981	NA
Austwickia	2.277	0.025	1.154	NA
Brucella	13.265	0.027	0.433	9.785e-01
Murine_type_C_virus	8.935	0.027	0.402	9.656e-01
Lachnoanaerobaculum	14.139	0.028	0.406	9.785e-01
Lautropia	241.645	0.028	0.205	9.780e-01
Tolypothrix	6.280	0.028	0.815	9.785e-01
Lampropedia	1.172	0.029	1.552	NA
Neoarthrinium	0.677	0.029	1.523	NA
Pelovirga	0.002	0.030	2.914	NA
Saccharothrix	13.589	0.033	0.549	9.785e-01
Tissierella	290.882	0.033	0.190	9.776e-01
Escherichia_phage_vB_EcoS_ESCO41_virus	1.030	0.034	1.405	NA
Beijerinckia	0.400	0.036	2.207	NA
Prosthecomicrobium	0.331	0.036	2.355	NA
Chondromyces	2.094	0.038	1.616	NA
Enterobacteria_phage_T7_virus	0.002	0.039	2.914	NA
Intrasporangium	1.104	0.042	1.775	NA
Yeguia	0.064	0.042	2.912	NA
Yimella	7.431	0.042	0.729	9.785e-01
Aphanizomenon	21.520	0.043	0.263	9.394e-01
Kluyvera	247.288	0.043	0.189	9.737e-01
harvey_murine_sarcoma_virus	0.947	0.044	0.470	NA
Terrabacter	109.045	0.047	0.236	9.737e-01
Psilocybe	1.438	0.047	0.873	NA
Actinokineospora	43.083	0.048	0.529	9.785e-01
Bacteroidales	0.562	0.048	1.729	NA
Shinella	19.684	0.048	0.542	9.785e-01
Pseudacidovorax	63.479	0.049	0.310	9.776e-01
Coriobacteriaceae_genus	0.483	0.050	2.380	NA
Sinisalibacter	0.394	0.050	1.946	NA
Betaproteobacterium_AAP51	11.054	0.050	0.549	9.780e-01
Mobilicoccus	15.933	0.051	0.572	9.785e-01
Truepera	3.557	0.052	1.075	NA
Cellulomonas	41.380	0.052	0.339	9.779e-01
Oxalobacteraceae_genus	2.110	0.053	0.860	NA
Barrientosiimonas	8.157	0.053	1.041	9.785e-01
Betaproteobacterium_AAP99	0.003	0.054	2.914	NA
Eikenella	9.058	0.056	0.566	9.785e-01
Nocardioides	721.043	0.057	0.156	9.656e-01
Paenirhodobacter	23.041	0.058	0.353	9.776e-01
Thermoleophilum	0.477	0.058	2.355	NA

Lewinella	0.693	0.059	0.581	NA
Hoylesella	15.245	0.059	0.557	9.785e-01
Aquabacterium	1382.076	0.059	0.244	9.737e-01
Tannerella	323.303	0.062	0.322	9.776e-01
Pontibacillus	7.036	0.062	1.264	9.874e-01
Primorskyibacter	0.613	0.063	0.939	NA
Luteipulveratus	0.361	0.063	2.609	NA
Citricoccus	24.500	0.063	0.476	9.785e-01
Delftia	511.917	0.064	0.194	9.656e-01
Planococcaceae_genus	0.288	0.064	2.907	NA
Intestinirhabdus	0.996	0.068	0.421	NA
Westerdykella	3.310	0.068	0.897	NA
Riesia	0.026	0.073	2.913	NA
Massilia	397.055	0.074	0.286	9.737e-01
Nanoperiomorbus	1.710	0.075	1.362	NA
Dyadobacter	16.940	0.075	0.468	9.776e-01
Moritella	186.504	0.076	0.166	9.497e-01
Nioella	0.006	0.076	2.914	NA
Enterobacteria_phage_YYZ-2008_virus	0.009	0.076	2.914	NA
Hydromonas	0.010	0.076	2.914	NA
Zafaria	0.027	0.076	2.914	NA
Boeremia	20.842	0.077	0.841	9.785e-01
Haliea	0.570	0.077	1.371	NA
Beutenbergia	4.200	0.077	1.107	NA
Fontibacillus	0.151	0.078	2.910	NA
Acidithiobacillus	129.947	0.079	0.475	9.776e-01
Niabella	0.539	0.079	1.812	NA
Maribellus	40.625	0.080	0.346	9.737e-01
Bordetella	18.960	0.080	0.384	9.776e-01
Stenotrophomonas	1057.148	0.081	0.113	9.324e-01
Rhodopseudomonas	31.634	0.081	0.352	9.737e-01
Desulfofundulus	0.368	0.082	2.910	NA
Cupriavidus	310.513	0.082	0.202	9.577e-01
Aerococcus	77.226	0.085	0.287	9.706e-01
SsRNA_phage_SRR5466369_2_virus	0.058	0.085	2.913	NA
Fulvia	4.128	0.085	0.701	NA
Microbacter	0.671	0.085	1.708	NA
Phyllobacterium	31.179	0.087	0.503	9.776e-01
Pseudocnuella	0.005	0.088	2.914	NA
Parvimonas	4.539	0.090	0.806	NA
Urbifossiella	0.741	0.091	2.168	NA
Renibacterium	7.693	0.092	0.785	9.785e-01
Cutibacterium	314.810	0.092	0.286	9.670e-01
Neofamilia	0.005	0.093	2.914	NA
Azorhizobium	2.897	0.093	0.918	NA
Caenimonas	0.820	0.094	1.300	NA
Mesorhizobium	79.330	0.094	0.194	9.396e-01
Oceanobacillus	36.670	0.096	0.285	9.577e-01
Terrisporobacter	682.815	0.096	0.323	9.717e-01
Anaerostipes	0.192	0.096	1.879	NA
Bacteriovorax	1.351	0.098	1.420	NA
Brevundimonas	1147.754	0.101	0.236	9.577e-01
Marinomonas	6.156	0.102	0.930	9.785e-01
Homoserinimonas	5.839	0.102	0.748	9.785e-01
Lelliottia	2.324	0.103	0.627	NA
UNVERIFIED_ORG:	13.458	0.103	0.482	9.745e-01
Gammaproteobacteria	2.722	0.105	0.986	NA
Levyella	7.688	0.105	1.134	9.785e-01
Yarrowia	13.249	0.105	0.776	9.785e-01

Paraferrimonas	4.903	0.106	0.652	NA
Oryzihumus	0.030	0.106	2.913	NA
Saitoella	9.414	0.110	1.673	9.785e-01
Roseomonas	144.562	0.110	0.387	9.737e-01
Paracidovorax	147.082	0.110	0.324	9.656e-01
Solihabitans	20.875	0.111	0.262	9.333e-01
Candidatus	2.663	0.111	1.355	NA
Bartonella	9.625	0.112	0.557	9.776e-01
Lichenibacterium	2.044	0.113	1.405	NA
Shigella	210548.549	0.114	0.302	9.638e-01
Dysgonamonadaceae_genus	13.608	0.114	0.722	9.776e-01
Clostridium	1001.246	0.115	0.188	9.333e-01
Falsochrobactrum	0.107	0.115	2.912	NA
Terribacillus	0.063	0.115	2.913	NA
Barnesiella	9.605	0.116	0.369	9.670e-01
Paramesorhizobium	0.152	0.117	2.912	NA
Phaeovulum	30.793	0.117	0.313	9.614e-01
Seohaeicola	2.294	0.118	1.308	NA
Aphanomyces	5.189	0.119	0.591	9.776e-01
Acetobacter	16.394	0.119	0.335	9.577e-01
Phenylobacterium	59.083	0.120	0.350	9.656e-01
Punctularia	10.023	0.123	0.882	9.785e-01
Phototrophicus	0.409	0.124	2.402	NA
Rhabdonatronobacter	170.476	0.124	0.242	9.387e-01
Caldovatus	0.480	0.124	1.723	NA
Pediococcus	0.807	0.125	1.055	NA
Piscinibacter	118.721	0.125	0.360	9.656e-01
Scleromatobacter	0.808	0.126	2.376	NA
Thiothrix	0.682	0.127	2.377	NA
Stereum	17.542	0.128	0.775	9.776e-01
Escherichia	61277.548	0.129	0.298	9.577e-01
Burkholderiales	19.138	0.130	0.425	9.656e-01
Corynebacterium	1916.937	0.131	0.227	9.333e-01
Roseibacterium	113.257	0.131	0.460	9.737e-01
Delta	4.266	0.132	0.590	NA
Baudoinia	11.960	0.133	0.469	9.737e-01
Zoogloea	42.077	0.134	0.350	9.590e-01
Oryzibacter	0.481	0.135	2.210	NA
Quadrisphaera	9.631	0.135	0.827	9.776e-01
Rhizobacter	51.463	0.138	0.309	9.333e-01
Achromobacter	178.806	0.142	0.131	7.827e-01
Chelatococcus	5.462	0.143	0.929	9.785e-01
Malassezia	1094.910	0.143	0.267	9.333e-01
Alpha	67.476	0.145	0.284	9.388e-01
Buchnera	70221.134	0.145	0.186	9.020e-01
Ralstonia	14254.930	0.146	0.162	8.678e-01
Halomonas	158.342	0.146	0.265	9.333e-01
Neglectibacter	0.247	0.147	1.381	NA
Gloeocapsa	4.503	0.148	0.976	NA
Centipeda	0.032	0.149	2.913	NA
Photobacterium	11.366	0.149	0.475	9.673e-01
Acuticoccus	38.672	0.149	0.252	9.020e-01
Anaerobutyricum	0.119	0.149	2.911	NA
Azomonas	2.713	0.151	0.887	NA
BeAn_58058_virus	0.384	0.151	1.138	NA
Bosea	160.841	0.151	0.189	8.970e-01
Acetivibrio	0.767	0.151	2.074	NA
Altererythrobacter	4.874	0.155	0.924	NA
Betaproteobacteria	0.088	0.156	2.912	NA

Agaricicola	0.028	0.157	2.914	NA
Actinobaculum	5.253	0.158	0.788	9.776e-01
Ensifer	36.688	0.159	0.309	9.333e-01
Bifidobacterium	47.754	0.161	0.340	9.501e-01
Pseudoclavibacter	10.937	0.161	0.612	9.737e-01
Pasteurellaceae_genus	2.823	0.162	0.907	NA
Alkanindiges	5.454	0.164	0.706	9.737e-01
Zobellella	1.254	0.167	1.632	NA
Pseudofrankia	0.261	0.169	2.454	NA
Saccharopolyspora	62.086	0.169	0.213	8.970e-01
Wenjunlia	0.648	0.175	2.131	NA
Thermothelomyces	0.628	0.175	1.369	NA
Fusobacterium	181.144	0.176	0.211	8.970e-01
Acidisphaera	1.711	0.177	1.528	NA
Pyrinomonas	9.505	0.177	0.879	9.776e-01
Azoarcus	4.010	0.177	0.841	NA
Amycolatopsis	5.663	0.177	0.508	9.656e-01
Polaromonas	23.598	0.178	0.300	9.333e-01
Carbonactinospora	0.476	0.180	2.031	NA
Brachymonas	13.279	0.181	0.443	9.577e-01
Sphingobium	158.875	0.181	0.194	8.483e-01
Segatella	9.943	0.183	0.578	9.656e-01
Colletotrichum	46.845	0.184	0.355	9.378e-01
Tepidicella	11.802	0.185	0.735	9.737e-01
Eremomyces	0.488	0.191	2.202	NA
Saccharomonospora	2.446	0.191	0.958	NA
Hyphomicrobium	34.815	0.191	0.292	9.333e-01
Microlunatus	62.692	0.192	0.410	9.501e-01
Atlanticothrix	0.252	0.193	2.510	NA
Phreatobacter	3.203	0.195	0.880	NA
Ruficoccus	65.274	0.197	0.351	9.333e-01
Iamia	3.587	0.198	1.094	NA
Azohydromonas	15.191	0.199	0.481	9.577e-01
Pigmentiphaga	5.865	0.199	0.739	9.737e-01
Phycicoccus	66.900	0.201	0.256	9.020e-01
Cystobacter	1.159	0.203	1.632	NA
Bergeriella	1.836	0.203	1.577	NA
Marinilabiliaceae_genus	2.283	0.204	0.780	NA
Jannaschia	2.279	0.204	0.407	NA
Betaproteobacterium_AAP121	0.906	0.206	1.097	NA
Rhodotorula	31.421	0.207	0.545	9.638e-01
Neoroseomonas	0.161	0.209	2.910	NA
Sulfuriferula	0.013	0.209	2.914	NA
Sulfitobacter	6.435	0.209	0.460	9.519e-01
Nostoc	288.872	0.210	0.197	7.851e-01
Pseudoxanthomonas	75.899	0.211	0.299	9.324e-01
Wenxinia	0.425	0.213	1.863	NA
Human_adenovirus_2	5.562	0.213	0.748	9.737e-01
Arthrobacter	566.114	0.215	0.182	7.262e-01
Miniphocaeibacter	0.034	0.215	2.913	NA
Cytobacillus	13.792	0.215	1.179	9.776e-01
Thermococcus	14.111	0.215	0.408	9.333e-01
Parasphingorhabdus	1.852	0.217	1.153	NA
Morganella	1.022	0.218	0.571	NA
Granulicatella	27.984	0.218	0.437	9.395e-01
Rheinheimera	4037.545	0.221	0.261	8.970e-01
Variimorphobacter	0.014	0.226	2.913	NA
Rosenbergiella	1.613	0.226	0.700	NA
Blautia	17.234	0.226	0.548	9.577e-01

Amygdalobacter	0.251	0.231	2.909	NA
Planctomyces	2.081	0.232	1.042	NA
Pisolithus	0.864	0.232	0.897	NA
Baekduia	2.199	0.233	1.096	NA
Porphyrobacter	4.254	0.234	0.623	NA
Enterococcus	585.175	0.234	0.132	4.426e-01
Pleionea	5.060	0.234	0.761	9.737e-01
Aggregatibacter	18.736	0.236	0.427	9.333e-01
Mus_musculus_mobilized_virus	23.218	0.241	0.325	9.008e-01
Thauera	36.207	0.242	0.236	8.038e-01
Acidocella	0.541	0.245	1.509	NA
Lactococcus	112.493	0.246	0.400	9.333e-01
Niastella	28.805	0.246	1.215	9.776e-01
Klugiella	0.423	0.246	2.905	NA
Eremococcus	4.175	0.247	0.873	NA
Trametes	10.328	0.248	0.738	9.656e-01
Phytohabitans	0.667	0.251	2.644	NA
Kaistia	3.343	0.251	0.972	NA
Brachybacterium	121.244	0.254	0.408	9.333e-01
Desertibacillus	0.917	0.255	1.626	NA
Dermacoccus	44.902	0.256	0.304	8.970e-01
Bifidobacteriaceae_genus	8.416	0.257	0.912	9.737e-01
Lysobacter	182.469	0.258	0.211	7.142e-01
Belnapia	18.847	0.259	0.616	9.577e-01
Halovulum	1.008	0.261	1.556	NA
Pseudovibrio	0.453	0.265	2.119	NA
Enterobacteria_phage_RTP_virus	2.620	0.269	0.907	NA
Blastomonas	151.122	0.269	0.288	8.483e-01
Entotheonella	3.765	0.271	0.682	NA
Protofrankia	62.094	0.271	0.369	9.218e-01
Neofusicoccum	40.723	0.272	0.451	9.333e-01
Tistrella	0.157	0.273	2.910	NA
Salegentibacter	0.151	0.274	2.910	NA
Paenibacillus	158.355	0.274	0.182	5.696e-01
Arenivirga	0.339	0.274	2.908	NA
Psychrobacter	94.340	0.275	0.250	7.772e-01
Halalkalibacter	112.475	0.275	0.303	8.678e-01
Pseudomassariella	0.846	0.276	2.026	NA
Idiomarinaceae_genus	0.023	0.276	2.913	NA
Limimaricola	2.701	0.278	1.413	NA
Salinarimonas	4.201	0.278	0.367	NA
Lawsonibacter	0.311	0.279	2.232	NA
Sedimentitalea	30.138	0.280	0.267	7.681e-01
Chaetomium	2.688	0.283	1.049	NA
Leptolyngbya	41.961	0.283	0.403	9.324e-01
Faunimonas	0.206	0.284	2.825	NA
Alcaligenes	8.410	0.284	0.605	9.519e-01
Zychaea	6.500	0.285	1.374	9.776e-01
Tessaracoccus	17.444	0.285	0.642	9.577e-01
Fontimonas	0.265	0.286	2.774	NA
Mangrovicoccus	5.222	0.291	0.898	9.670e-01
Alcanivorax	267.141	0.291	0.229	6.896e-01
Endobacter	3.493	0.291	1.179	NA
Miltoncostaea	4.350	0.292	1.331	NA
Desulfogranum	1.068	0.293	1.470	NA
Kytococcus	23.988	0.293	0.491	9.333e-01
Actinomyces	520.914	0.297	0.239	7.097e-01
Moraxella	437.707	0.297	0.248	7.172e-01
Enhydrobacter	51.501	0.298	0.281	7.859e-01

Calidifontimicrobium	0.381	0.300	2.910	NA
Ottowia	13.988	0.300	0.476	9.333e-01
Aliiruegeria	16.063	0.304	0.576	9.333e-01
Pichia	0.685	0.304	1.355	NA
Blastococcus	150.500	0.307	0.383	9.008e-01
Human_endogenous_retrovirus	5.191	0.307	0.536	9.333e-01
Hammondia	7.312	0.310	0.475	9.333e-01
Bdellovibrio	0.992	0.310	1.273	NA
Coccidioides	0.857	0.313	1.201	NA
Marinilactibacillus	3.912	0.316	1.072	NA
Prevotellaceae_genus	6.860	0.316	0.676	9.519e-01
Acidiplasma	17.926	0.317	0.332	8.432e-01
Psychromonas	1.967	0.318	0.654	NA
Isoptericola	31.305	0.319	0.235	6.229e-01
Fuscibacter	0.854	0.319	2.071	NA
Anaerosphaera	0.551	0.323	2.148	NA
Cucumibacter	0.022	0.326	2.913	NA
Komagataeibacter	1.071	0.327	1.522	NA
Alteribacter	6.236	0.328	0.407	8.970e-01
Propionibacterium_phage_PHL041M10_virus	0.037	0.330	2.913	NA
Propionibacteriaceae_genus	1.387	0.331	1.346	NA
Kocuria	406.292	0.331	0.393	8.970e-01
Aliidongia	0.759	0.333	1.597	NA
Sandaracinobacteroides	6.329	0.336	0.761	9.519e-01
Chryseolinea	0.170	0.338	2.912	NA
Taibaiella	8.236	0.338	0.743	9.519e-01
Krasilnikoviella	0.109	0.339	2.911	NA
Emiliania	0.907	0.339	1.183	NA
Xylanimonas	2.036	0.340	0.910	NA
Xenorhabdus	3.486	0.342	0.420	NA
Dolosigranulum	11.557	0.342	0.680	9.395e-01
Pelorhabdus	4.813	0.344	0.712	NA
Desulfovibrio	71087.628	0.346	0.223	5.413e-01
Paracandidimonas	0.784	0.348	2.133	NA
Aquincola	15.393	0.349	0.490	9.324e-01
Pseudopropionibacterium	6.216	0.350	0.680	9.388e-01
Type-B_symbiont_of_Plautia_stali	0.070	0.351	2.376	NA
Rubricoccus	0.152	0.351	2.913	NA
Shewanella	593.636	0.353	0.142	2.542e-01
Ktedonobacter	0.238	0.354	2.908	NA
Provencibacterium	0.082	0.357	2.912	NA
Erysipelothrix	0.043	0.357	2.913	NA
Mycobacterium	323.882	0.358	0.185	4.135e-01
Kaistella	17.043	0.360	0.477	9.092e-01
Robinsoniella	0.031	0.361	2.913	NA
Tatumella	7.235	0.362	0.603	9.333e-01
Amorphotheca	6.328	0.364	0.670	9.333e-01
Betaproteobacterium_AAP65	0.934	0.368	1.094	NA
Annandia	0.084	0.369	2.076	NA
Algibacillus	1.251	0.370	0.931	NA
Virgisporangium	0.313	0.371	2.056	NA
Rhodofomes	4.456	0.372	0.805	NA
Metarhizium	16.962	0.372	0.547	9.333e-01
Hallella	4.170	0.374	0.905	NA
Roseovarius	20.641	0.374	0.353	7.827e-01
Arcticiflavibacter	197.563	0.375	0.323	7.387e-01
Capsulimonas	0.344	0.377	2.020	NA
Caulobacteraceae_genus	0.901	0.377	2.003	NA
Hydrotalea	0.322	0.377	2.667	NA

Puniceibacterium	0.603	0.378	1.666	NA
Nitrosospira	1.058	0.382	1.572	NA
Agrococcus	150.918	0.384	0.647	9.333e-01
Saccharophagus	0.820	0.387	1.790	NA
Virgibacillus	32.734	0.389	0.246	5.162e-01
Terriglobus	1.251	0.389	1.215	NA
Escherichia_phage_DTL_virus	0.756	0.390	1.877	NA
Telluria	16.853	0.392	0.474	8.970e-01
Romboutsia	3.447	0.396	0.741	NA
Pseudidiomarina	0.129	0.397	1.820	NA
Escherichia_phage_phiV10_virus	2.464	0.399	0.983	NA
Aquariibacter	0.913	0.399	1.281	NA
Georgenia	10.823	0.399	0.640	9.333e-01
Grimontella	3.770	0.402	0.730	NA
Pseudozyma	9.591	0.402	0.716	9.333e-01
Adhaeribacter	5.729	0.403	1.090	9.590e-01
Robbsia	0.852	0.403	1.316	NA
Dongshaea	0.131	0.404	2.910	NA
Microcystis	7129.664	0.408	0.365	7.681e-01
Luteibacter	4.401	0.409	0.809	NA
Stutzerimonas	32.547	0.410	0.440	8.533e-01
Cobetia	0.433	0.411	1.432	NA
Lentilactobacillus	12.683	0.413	1.179	9.656e-01
Superficieibacter	0.163	0.416	1.132	NA
Pedococcus	6.046	0.417	0.702	9.333e-01
Nesterenkonia	69.289	0.417	0.270	5.413e-01
Planobispora	0.083	0.418	2.911	NA
PreXMRV-1_provirus_complete	4.785	0.418	0.492	NA
Helicobacter	0.471	0.419	2.182	NA
Zimmermannella	7.604	0.419	0.766	9.333e-01
Sphaerulina	8.660	0.421	0.653	9.333e-01
Uncultured	130.743	0.423	0.356	7.262e-01
Saezia	2.274	0.423	0.862	NA
Ramularia	0.158	0.423	2.913	NA
Actinophytocola	1.418	0.425	1.612	NA
Janthinobacterium	149.394	0.427	0.241	4.426e-01
Profftia	0.070	0.429	2.912	NA
Seramator	7.459	0.432	0.733	9.333e-01
Wigglesworthia	1.349	0.437	0.842	NA
Leptotrichia	40.411	0.441	0.329	6.499e-01
Parageobacillus	1.709	0.442	1.417	NA
Gemmatimonas	2.554	0.444	1.312	NA
Rhodoferax	44.739	0.445	0.315	6.075e-01
Actinobacterium	1.502	0.446	1.610	NA
Melittangium	0.241	0.447	2.793	NA
Crocosphaera	13.832	0.448	0.396	7.535e-01
Solirubrobacter	19.559	0.449	0.552	9.003e-01
Acetanaerobacterium	0.863	0.449	1.632	NA
Bacterium	36.502	0.451	0.233	4.135e-01
Friedmanniella	3.135	0.451	1.175	NA
Methylotenera	11.956	0.452	0.649	9.333e-01
Yonghaparkia	1.392	0.452	1.371	NA
Pochonia	0.554	0.453	1.521	NA
Nitrolancea	0.665	0.453	1.720	NA
Terracoccus	15.641	0.454	0.780	9.333e-01
Aggregatilinea	0.220	0.456	2.909	NA
Picosynechococcus	8.815	0.458	0.698	9.072e-01
Cryptosporangium	2.818	0.458	1.296	NA
Fannyhessea	3.108	0.467	1.170	NA

Rhodoblastus	1.061	0.468	1.557	NA
Tenebrionicola	0.031	0.468	2.913	NA
Alkalispirochaeta	9160.805	0.470	0.234	4.052e-01
Lacibacter	0.974	0.470	2.319	NA
Sandaracinobacter	0.244	0.471	2.912	NA
Pedomonas	4.318	0.474	1.070	NA
Camelimonas	0.584	0.475	2.193	NA
Niallia	0.268	0.475	1.971	NA
Azonexus	3.481	0.475	1.073	NA
Flectobacillus	1.269	0.478	1.295	NA
Metabacillus	4.195	0.478	1.066	NA
Trabulsiella	0.462	0.481	0.604	NA
Type-F_symbiont_of_Plautia_stali	0.110	0.481	2.913	NA
Postia	8.620	0.484	0.792	9.333e-01
Thiohalocapsa	35.128	0.485	0.355	6.281e-01
Laetiporus	0.632	0.485	2.904	NA
Brevibacterium	83.680	0.486	0.332	5.853e-01
Embleya	1.575	0.488	1.423	NA
Ciceribacter	3.850	0.488	0.806	NA
Kingella	8.778	0.490	0.510	8.432e-01
Castellaniella	1.341	0.493	1.493	NA
Type-E_symbiont_of_Plautia_stali	0.036	0.494	2.913	NA
Methylophaga	0.781	0.497	1.190	NA
Paecilomyces	3.711	0.497	0.899	NA
Peredibacter	28.482	0.497	0.571	8.842e-01
Blattabacterium	0.399	0.500	2.167	NA
Luteolibacter	24.040	0.501	0.397	6.919e-01
Polymorphum	0.458	0.503	1.741	NA
Anaerotruncus	0.786	0.504	0.869	NA
Frigidibacter	67.900	0.507	0.278	4.421e-01
Streptobacillus	0.298	0.515	2.907	NA
Propionicicella	1.665	0.516	1.272	NA
Putridiphycobacter	2.985	0.517	0.762	NA
Rubellimicrobium	30.281	0.521	0.535	8.432e-01
Alkalihalophilus	0.036	0.521	2.913	NA
Ectothiorhodospira	16.455	0.522	0.357	5.792e-01
Limnohabitans	19.440	0.522	0.338	5.413e-01
Seonamhaeicola	12.679	0.523	0.415	6.840e-01
Conexibacter	10.772	0.525	0.685	9.072e-01
Azospirillum	271.311	0.526	0.338	5.413e-01
Simplicispira	1.925	0.527	0.934	NA
Abelson	3.843	0.529	0.474	NA
Pseudorhizobium	3.035	0.530	1.085	NA
Perlucidibaca	1.327	0.530	0.961	NA
Legionella	1332.136	0.531	0.233	2.947e-01
Berkiella	0.057	0.532	2.912	NA
Cryptosporidium	15.505	0.535	0.490	7.827e-01
Hoeflea	3.595	0.535	0.942	NA
Desulfocarbo	2.982	0.536	0.929	NA
Beijerinckiaceae_genus	37.739	0.544	0.699	9.046e-01
Euzebya	0.588	0.547	1.253	NA
Teredinibacter	0.038	0.548	2.913	NA
Methylocaldum	0.238	0.548	1.757	NA
Allorhizobium	0.157	0.549	2.910	NA
Uruburuella	0.038	0.550	2.913	NA
Hyaloscypha	2.005	0.552	0.835	NA
Phialemonium	0.746	0.554	2.020	NA
Tsuneonella	2.101	0.554	1.400	NA
Xanthomonas	75.563	0.556	0.289	4.196e-01

Janibacter	177.517	0.556	0.229	2.666e-01
Thermaerobacter	0.441	0.558	2.406	NA
Aridibaculum	0.190	0.561	2.912	NA
Magnetospirillum	1.981	0.563	0.941	NA
Bowmanella	57.320	0.566	0.324	4.426e-01
Rhodocyclaceae_genus	4.038	0.568	0.883	NA
Paracraurococcus	0.696	0.568	1.732	NA
Nitratireductor	1.522	0.570	0.970	NA
Propionibacterium	5198.439	0.570	0.279	3.892e-01
Leptomonas	2.477	0.571	1.066	NA
Eimeria	0.728	0.571	1.007	NA
Hyphobacterium	32.142	0.576	0.297	4.135e-01
Protomyces	14.887	0.582	0.975	9.333e-01
Rhodophyticola	0.237	0.583	2.908	NA
Papillibacter	0.041	0.584	2.913	NA
Cardiobacterium	13.923	0.585	0.562	7.957e-01
Sporothrix	1.005	0.587	1.569	NA
Thecamonas	1.583	0.588	1.341	NA
Albitalea	0.203	0.589	2.824	NA
Mucor	15.990	0.589	0.849	9.333e-01
Aliihoeflea	2.600	0.590	1.420	NA
Sodalis	1.787	0.591	0.401	NA
Pengzhenrongella	1.275	0.596	1.497	NA
Petrotoga	0.426	0.596	2.150	NA
Planctopirus	0.379	0.596	1.506	NA
Didymella	13.775	0.598	1.021	9.333e-01
Ferribacterium	0.267	0.603	2.908	NA
Pseudarthrobacter	55.707	0.605	0.455	6.605e-01
Desulfoscipio	0.435	0.608	2.377	NA
Pseudescherichia	0.152	0.610	2.557	NA
Glaciecola	6.760	0.612	0.355	4.502e-01
Mycolicibacterium	115.168	0.613	0.316	4.196e-01
Alicyclobacillus	0.659	0.614	2.000	NA
Microdochium	4.060	0.615	0.752	NA
Nanogingivalis	0.227	0.617	1.911	NA
Williamsia	113.724	0.620	0.444	6.187e-01
Leishmania	4.184	0.621	0.980	NA
Moranella	0.333	0.622	2.541	NA
Tardiphaga	3.851	0.624	0.743	NA
Tuwongella	0.369	0.625	2.908	NA
Myxococcales	1.509	0.630	1.927	NA
Dysgonomonas	10.780	0.636	0.439	5.905e-01
Luteococcus	0.817	0.637	1.983	NA
Sporichthya	4.613	0.638	1.036	NA
Pseudoramibacter	88.151	0.640	0.506	6.899e-01
Lachnospiraceae_genus	22.274	0.641	0.442	5.878e-01
Firmicutes	1.284	0.642	1.199	NA
Gallaecimonas	1.455	0.643	0.928	NA
Inquilinus	0.544	0.645	1.432	NA
Jeotgalibaca	0.592	0.647	1.875	NA
Haliangium	2.455	0.652	1.464	NA
Schizosaccharomyces	2.310	0.652	1.100	NA
Acidiluteibacter	0.200	0.652	2.911	NA
Methylopila	6.130	0.653	0.841	9.072e-01
Saccharibacillus	2.894	0.654	1.316	NA
Scytonema	22.749	0.655	0.478	6.229e-01
Mycetocola	0.892	0.661	1.245	NA
Herbiconiux	5.773	0.666	0.985	9.333e-01
Hydrogenophaga	66.646	0.667	0.376	4.426e-01

Fredinandcohnia	6.991	0.669	0.600	7.681e-01
Synechocystis	15.469	0.670	0.653	8.049e-01
Treponema	11.715	0.670	0.506	6.605e-01
Paraglaciecola	2.599	0.672	0.643	NA
Peribacillus	4.798	0.674	0.919	NA
Geomonas	0.050	0.675	2.912	NA
Cucurbitaria	0.669	0.676	1.580	NA
Cnuella	0.966	0.677	1.744	NA
Granulicoccus	0.329	0.680	2.651	NA
Neptuniibacter	0.092	0.681	2.911	NA
Veillonella	194.159	0.684	0.328	3.735e-01
Photorhabdus	47.052	0.685	0.363	4.223e-01
Natronorubrum	1.279	0.687	1.378	NA
Pandoraea	33.582	0.689	0.309	3.204e-01
Winogradskyella	5.235	0.690	0.377	4.196e-01
Phaeobacter	1.043	0.692	0.833	NA
Pneumocystis	5.632	0.692	1.258	9.333e-01
Thermacetogenium	0.831	0.694	2.466	NA
Verticillium	0.616	0.699	1.636	NA
Proteus_phage_VB_PmiS-Isfahan_virus	1.743	0.702	0.526	NA
Miniimonas	0.445	0.703	2.405	NA
Sulfolobus	2.237	0.709	0.450	NA
Chryseosolibacter	0.194	0.709	2.909	NA
Nitrosocosmicus	0.792	0.710	1.862	NA
Melampsora	6.780	0.711	1.415	9.396e-01
Ferruginibacter	1.535	0.713	1.234	NA
Dietzia	166.910	0.715	0.323	3.329e-01
Phaeosphaeria	2.821	0.716	1.533	NA
Gynurincola	0.054	0.719	2.912	NA
Selenomonas	7.222	0.720	0.541	6.427e-01
Mesonia	2.526	0.720	0.677	NA
Roseicella	3.466	0.728	0.948	NA
Tricharina	1.265	0.731	1.202	NA
Agromyces	13.851	0.733	0.410	4.426e-01
Anatilimnocola	0.489	0.738	2.908	NA
Buttiauxella	1.130	0.739	0.984	NA
Micrococcaceae_genus	0.443	0.744	1.850	NA
Staphylococcus	1969.163	0.745	0.277	1.831e-01
Aquitalea	1.173	0.745	0.735	NA
Streptoalloteichus	1.111	0.746	1.318	NA
Pseudoroseomonas	11.878	0.748	0.589	6.899e-01
Tenebrionibacter	0.381	0.750	0.980	NA
Serinicoccus	17.817	0.753	0.788	8.432e-01
Hartmannibacter	0.288	0.758	2.907	NA
Spleen_focus-forming_virus	3.983	0.758	0.465	NA
Solirhodobacter	0.058	0.761	2.912	NA
Acidaminobacter	5.364	0.762	0.613	7.142e-01
Crenobacter	1.097	0.765	0.886	NA
Neokomagataea	0.142	0.770	2.910	NA
Abiotrophia	14.388	0.773	0.604	6.866e-01
Xylaria	0.604	0.774	1.727	NA
Rathayibacter	27.104	0.775	0.635	7.142e-01
Emergencia	2.121	0.775	1.376	NA
Gallionella	0.539	0.777	1.002	NA
Dechloromonas	6.203	0.778	0.676	7.535e-01
Aquibium	0.920	0.784	1.667	NA
Undibacterium	178.266	0.784	0.329	2.666e-01
Tychonema	1.638	0.785	1.130	NA
Brettanomyces	1.793	0.787	1.550	NA

Pyruvatibacter	1.367	0.793	0.753	NA
Pseudohongiella	0.062	0.798	2.912	NA
Rudaea	1.559	0.801	0.728	NA
Exiguobacterium	51.512	0.803	0.332	2.666e-01
Roseibium	0.641	0.803	1.624	NA
Dermatobacter	0.611	0.806	1.700	NA
Youxingia	0.215	0.810	2.491	NA
Shouchella	0.581	0.813	2.727	NA
Vibrio	744.207	0.813	0.252	6.241e-02
Pirellulimonas	0.064	0.813	2.912	NA
Andreesenia	0.720	0.815	2.568	NA
Arachidicoccus	0.202	0.818	2.911	NA
Dialister	5.744	0.818	0.951	8.970e-01
Petrimonas	0.193	0.819	2.911	NA
Clavibacter	4.588	0.820	0.913	NA
Pseudaestuariivita	0.064	0.821	2.912	NA
Aestuariimicrobium	3.255	0.823	0.972	NA
Zeimonas	0.839	0.826	1.741	NA
Filomicrobium	2.369	0.831	1.071	NA
Gemella	167.060	0.838	0.409	3.892e-01
Penicilliopsis	0.668	0.840	2.322	NA
Campylobacter	39.556	0.840	0.286	1.132e-01
Rehaibacterium	0.368	0.846	1.985	NA
Betaproteobacterium_JGI	8.316	0.847	0.413	3.892e-01
Oscillibacter	0.819	0.852	2.379	NA
Dubosiella	0.165	0.859	2.866	NA
Roseicitreum	2.086	0.863	1.196	NA
Rhodopirellula	20.297	0.866	0.413	3.645e-01
Alteromonas	44.223	0.867	0.484	4.426e-01
Paraprevotella	2.071	0.872	1.133	NA
Naasia	4.140	0.873	1.088	NA
Nevskia	5.606	0.875	1.061	9.008e-01
Proteiniclasticum	0.907	0.875	0.826	NA
Caldicellulosiruptor	0.237	0.876	2.908	NA
Lawsonella	225.924	0.880	0.410	3.475e-01
Patulibacter	9.106	0.880	0.743	7.262e-01
Ligilactobacillus	14.208	0.881	0.631	6.229e-01
Serpentinimonas	0.193	0.881	2.909	NA
Microcoleus	16.615	0.885	0.544	5.162e-01
Nisaea	0.072	0.886	2.912	NA
Weeksella	0.444	0.886	2.912	NA
Psychrosphaera	4.364	0.887	0.623	NA
Paracaedibacter	1.572	0.888	1.619	NA
Ochrobactrum	77.175	0.890	0.343	2.287e-01
Phialophora	4.587	0.891	0.945	NA
Syntrophomonas	0.225	0.891	2.909	NA
Aliterella	5.985	0.897	1.020	8.884e-01
Sordaria	1.077	0.897	1.186	NA
Ruminococcus	28.348	0.899	0.476	4.223e-01
Oceanicella	2.913	0.899	0.870	NA
Kitasatospora	20.269	0.901	0.552	5.046e-01
Chloroflexales	0.074	0.901	2.912	NA
Isoalcanivorax	1.062	0.903	1.734	NA
Laspinema	0.879	0.903	1.847	NA
Salinicoccus	15.586	0.910	0.632	6.014e-01
Vogesella	3.725	0.911	0.906	NA
Chryseobacterium	293.101	0.911	0.187	2.065e-04
Archangium	2.807	0.911	0.788	NA
Acidobacteria	2.858	0.920	0.897	NA

Mixta	7.632	0.935	0.530	4.426e-01
Leyella	2.026	0.935	1.218	NA
Aquisphaera	2.453	0.935	1.240	NA
Westeberhardia	22.020	0.941	0.406	2.696e-01
Solibacillus	6.613	0.942	0.639	5.792e-01
Kockovaella	3.506	0.947	1.009	NA
Amnimonas	9.718	0.949	1.110	8.970e-01
Salicibibacter	0.350	0.952	2.907	NA
Glaesserella	0.891	0.954	0.900	NA
Soleaferrea	0.148	0.954	2.910	NA
Thermoanaerobacter	0.462	0.959	2.907	NA
Rectinema	0.081	0.963	2.912	NA
Stakelama	0.615	0.964	1.562	NA
Brasilonema	1.390	0.965	1.497	NA
Kwoniella	6.912	0.969	0.927	7.957e-01
Metasolibacillus	1.157	0.969	1.243	NA
Macellibacteroides	0.431	0.973	2.117	NA
Agathobaculum	0.083	0.975	2.912	NA
Abditibacterium	5.942	0.979	0.954	8.128e-01
Nodosilinea	21.716	0.980	0.759	6.866e-01
Arsenophonus	1.327	0.990	0.609	NA
Propionibacterium_phage_P100D_virus	0.085	0.991	2.911	NA
Salinimicrobium	20.796	0.991	0.455	3.445e-01
Ureibacillus	0.798	0.993	1.451	NA
Gluconobacter	9.302	0.995	0.629	5.334e-01
Halobacillus	0.907	0.995	1.169	NA
Tahibacter	0.454	0.996	1.560	NA
Gulosibacter	3.895	0.998	1.078	NA
Chitinimonas	12.648	1.000	0.675	5.792e-01
Carnobacterium	19.889	1.002	0.473	3.486e-01
Aureococcus	4.750	1.003	0.824	NA
Rhizophagus	3.679	1.005	1.040	NA
Flavonifractor	0.231	1.009	2.910	NA
Citreimonas	0.088	1.014	2.911	NA
Plastoroseomonas	0.681	1.015	2.006	NA
Fervidibacillus	1.551	1.019	2.031	NA
Enterovirga	5.894	1.020	0.940	7.851e-01
Acidobacteriaceae_genus	0.311	1.027	2.443	NA
Gamsiella	4.971	1.029	1.107	NA
Roseibaca	0.208	1.037	2.446	NA
Siccirubricoccus	1.651	1.038	1.379	NA
Aggregicoccus	0.236	1.040	2.911	NA
Acidaminococcus	0.185	1.043	2.909	NA
Lederbergia	0.161	1.047	2.794	NA
Hydrobacter	0.593	1.056	2.000	NA
Mixia	2.238	1.059	1.186	NA
Ornithinimicrobium	139.539	1.059	0.446	2.666e-01
Humibacter	0.118	1.061	2.911	NA
Algiphilus	4.759	1.064	0.844	NA
Thalassiosira	9.266	1.065	0.563	4.223e-01
Skermanella	18.493	1.066	0.528	4.106e-01
Talaromyces	10.629	1.071	0.688	5.413e-01
Apiotrichum	11.988	1.075	0.767	6.187e-01
Verrucosispora	2.100	1.082	0.952	NA
Oceanitalea	1.246	1.083	1.583	NA
Moheibacter	0.373	1.086	2.622	NA
Sporomusaceae_genus	0.444	1.091	1.286	NA
Acidipropionibacterium	24.957	1.092	0.582	4.223e-01
Calidifontibacter	0.577	1.100	2.193	NA

Scandinavium	0.642	1.109	0.609	NA
Nanosynsacchari	0.734	1.112	1.490	NA
Mammaliicoccus	12.984	1.113	0.725	5.661e-01
Butyricicoccus	0.662	1.113	1.621	NA
Paraclostridium	10.370	1.114	0.684	5.162e-01
Pseudoflavonifractor	1.265	1.115	0.986	NA
Aquimarina	0.749	1.117	1.941	NA
Anaeromyxobacter	2.178	1.117	1.246	NA
Alistipes	1.076	1.120	1.574	NA
Pseudorhodoplanes	0.962	1.122	1.224	NA
Ewingella	0.845	1.126	2.338	NA
Phycomyces	1.988	1.127	1.140	NA
Ogataea	1.444	1.129	1.312	NA
Myceligenerans	6.325	1.130	0.566	4.067e-01
Exophiala	38.352	1.133	0.593	4.223e-01
Glycocaulis	61.217	1.134	1.091	8.038e-01
Rivularia	0.632	1.139	1.992	NA
Geobacillus	19.526	1.144	0.569	4.106e-01
Paludicola	0.106	1.144	2.911	NA
Crenalkalicoccus	0.463	1.147	2.059	NA
Parachlamydiaceae_genus	0.346	1.148	2.907	NA
Trypanosoma	18.124	1.153	0.577	4.106e-01
Calothrix	3.097	1.153	0.920	NA
Arboricoccus	0.363	1.155	2.306	NA
Propionimicrobium	87.295	1.159	0.491	2.696e-01
Spiribacter	38.734	1.161	0.409	1.431e-01
Planktothrix	6.905	1.165	0.614	4.223e-01
Gallintestinimicrobium	0.837	1.165	2.604	NA
Carboxylicivirga	0.110	1.166	2.911	NA
Pontibacter	3.192	1.171	1.417	NA
Thioclava	0.294	1.172	2.314	NA
Trichophyton	4.105	1.174	1.387	NA
Aromatoleum	0.450	1.175	2.178	NA
Gemmataceae_genus	0.813	1.177	1.852	NA
Aquabacter	1.215	1.178	1.235	NA
Catenibacterium	1.044	1.187	1.834	NA
Allofustis	0.129	1.197	2.911	NA
Parapedobacter	0.475	1.198	1.322	NA
Parapusillimonas	0.200	1.198	2.911	NA
Ilyonectria	3.065	1.201	1.078	NA
Haladaptatus	2.468	1.204	0.776	NA
Proteiniphilum	0.183	1.208	2.910	NA
Sodalis-like	0.331	1.215	0.997	NA
Zoogloeaceae_genus	0.238	1.216	2.909	NA
Aff.	0.263	1.222	2.908	NA
Enterobacteria_phage_vB_EcoS_IME542_virus	2.844	1.229	1.018	NA
Flaviflexus	0.626	1.230	1.671	NA
Fodinicola	0.339	1.234	2.673	NA
Acidihalobacter	28.945	1.234	0.507	2.666e-01
Flavipsychrobacter	1.147	1.240	2.181	NA
Vampirovibrio	0.259	1.241	2.908	NA
Type-C_symbiont_of_Plautia_stali	0.212	1.245	2.754	NA
Reyranella	10.238	1.247	0.554	3.139e-01
Mycoplasma	1.308	1.249	0.791	NA
Polynucleobacter	1.891	1.252	0.913	NA
Cellulosimicrobium	4.141	1.260	1.256	NA
Runella	0.324	1.276	2.119	NA
FBR_murine_osteosarcoma_virus	1.393	1.279	0.593	NA
Flagellatimonas	0.126	1.279	2.911	NA

Durotheca	0.146	1.284	2.910	NA
Ustilago	1.167	1.286	1.328	NA
Parerythrobacter	4.100	1.289	1.297	NA
Chlorogloea	3.479	1.290	1.293	NA
Azovibrio	1.050	1.291	1.496	NA
Haloechinothrix	0.329	1.292	2.370	NA
Rhodocyclus	0.572	1.295	1.508	NA
Allocoleopsis	0.836	1.295	1.735	NA
Dickeya	1.888	1.297	0.421	NA
Pseudolabrys	0.793	1.301	1.529	NA
Rhodospirillaceae_genus	1.267	1.310	1.339	NA
Roseococcus	1.779	1.312	1.335	NA
Paracnuella	0.644	1.312	1.257	NA
Propionibacterium_phage_PHL116M00_virus	0.473	1.313	2.655	NA
Labrys	96.739	1.329	0.366	2.498e-02
Desnuesiella	0.296	1.330	2.911	NA
Vagococcus	0.603	1.342	2.358	NA
Defluviimonas	0.700	1.344	1.549	NA
Malikia	0.578	1.345	1.964	NA
Prolinoborus	3.096	1.350	0.810	NA
Dinghuibacter	0.335	1.355	2.909	NA
Rhodovibrio	0.588	1.356	1.962	NA
Gemmata	0.949	1.360	1.528	NA
Pyricularia	0.300	1.362	2.378	NA
Methanotrophic	6.831	1.367	0.776	4.426e-01
Thermaurantiacus	0.224	1.368	2.909	NA
Filamentous	0.701	1.372	1.777	NA
Rhodocista	0.302	1.376	1.643	NA
Synechococcus	0.899	1.378	1.397	NA
Tsukamurella	6.889	1.379	1.254	7.851e-01
Piscicoccus	1.787	1.379	1.514	NA
Parasutterella	0.614	1.411	1.300	NA
Actirhodobacter	0.195	1.414	2.910	NA
Pseudogemmobacter	0.912	1.417	2.181	NA
Chloroflexus	0.443	1.418	2.369	NA
Nitrospira	0.501	1.421	2.052	NA
Komarekiella	0.170	1.432	2.910	NA
Paenalcaligenes	0.760	1.435	0.906	NA
Prosthecobacter	4.698	1.440	0.983	NA
Brevibacillus	1.944	1.443	0.907	NA
Fusicatenibacter	1.772	1.446	1.351	NA
Nitriliruptoraceae_genus	81.801	1.448	0.863	4.966e-01
Parvularcula	2.074	1.448	1.400	NA
Planctomonas	5.568	1.454	0.896	5.323e-01
Gamma	2.482	1.454	1.062	NA
Faecalimonas	0.765	1.454	1.578	NA
Pectobacterium_phage_CBB_virus	1.397	1.458	1.420	NA
Atopococcus	0.184	1.462	2.910	NA
Ornithinicoccus	0.159	1.464	2.816	NA
Cordyceps	1.093	1.465	1.613	NA
Labedella	0.708	1.466	2.012	NA
Aquimonas	1.549	1.470	1.935	NA
Tomitella	1.308	1.488	1.325	NA
Trichococcus	0.181	1.490	2.801	NA
Quisquiliibacterium	0.626	1.500	1.957	NA
Hanamia	0.253	1.504	2.910	NA
Xanthomarina	0.828	1.504	1.547	NA
Tautonia	1.653	1.504	1.503	NA
Plautia	0.323	1.504	1.596	NA

Desulfuromonas	1.691	1.510	1.449	NA
Lujinxingia	0.179	1.513	1.821	NA
Pedosphaera	0.186	1.519	2.910	NA
Coleofasciculus	1.654	1.520	1.276	NA
Sulfuricystis	0.442	1.520	2.608	NA
Pseudonocardiaceae_genus	1.243	1.522	1.624	NA
Nannochloropsis	1.567	1.528	1.253	NA
Verrucomicrobia	1.426	1.553	1.637	NA
Murine_osteosarcoma_virus	2.353	1.563	0.645	NA
Propionispora	0.201	1.564	2.909	NA
Zymomonas	0.330	1.579	2.909	NA
Cryptococcus	7.850	1.581	0.785	4.135e-01
Desulforhabdus	2.687	1.588	1.051	NA
Bergeyella	0.183	1.597	2.786	NA
Limnobacter	116.231	1.599	0.553	1.408e-01
Paeniroseomonas	0.513	1.602	1.681	NA
Anaerococcus	995.459	1.605	0.543	1.231e-01
Prauserella	0.186	1.610	2.910	NA
Falseniella	0.389	1.611	2.323	NA
Dysosmobacter	0.526	1.616	2.108	NA
Micavibrio	0.689	1.623	1.791	NA
Aminobacter	2.429	1.623	0.916	NA
Glycomyces	0.831	1.628	1.807	NA
Ferrovum	0.569	1.634	2.323	NA
Cryptomonas	1.703	1.640	1.220	NA
Leptosphaeria	39.541	1.644	0.738	3.348e-01
Holdemania	0.350	1.645	2.907	NA
Pontibrevibacter	0.195	1.655	2.910	NA
Macrococcus	6.515	1.667	0.770	3.475e-01
Coriobacteriales	0.346	1.669	2.660	NA
Citreicoccus	0.220	1.677	2.909	NA
Neoactinobaculum	0.622	1.677	2.102	NA
Erysipelotrichaceae_genus	0.333	1.681	2.908	NA
Cohnella	3.253	1.683	0.665	NA
Pseudanabaena	0.202	1.686	2.909	NA
Cutaneotrichosporon	17.445	1.688	0.913	4.426e-01
Wielerella	1.083	1.691	1.781	NA
Cereal_yellow_dwarf_virus	0.223	1.693	2.909	NA
Rhodospirillales	0.991	1.697	1.627	NA
Immundisolibacter	1.554	1.699	1.176	NA
Isosphaera	0.330	1.702	2.908	NA
Halopseudomonas	3.486	1.704	0.858	NA
Singulisphaera	6.587	1.709	0.862	4.135e-01
Pararobbsia	0.255	1.713	2.909	NA
Methylocystis	2.390	1.716	0.836	NA
Agarivorans	92.107	1.718	0.567	1.132e-01
Alishewanella	18.469	1.735	0.647	2.194e-01
Steroidobacter	1.182	1.738	1.304	NA
Pelistega	0.345	1.749	2.909	NA
Rodentibacter	0.334	1.750	1.787	NA
Quatrionicoccus	0.245	1.757	2.909	NA
Alkalihalobacillus	4.260	1.764	0.562	NA
Capillimicrobium	2.546	1.764	1.234	NA
Glaciihabitans	0.993	1.791	1.436	NA
Pannonibacter	2.632	1.797	1.299	NA
Niveispirillum	1.564	1.804	1.419	NA
Holdemanella	0.771	1.808	1.836	NA
Frateuria	2.007	1.811	0.731	NA
Qaidamihabitans	5.033	1.837	1.086	4.930e-01

Brooklawnia	0.606	1.856	2.088	NA
Liquorilactobacillus	0.655	1.871	1.658	NA
Phaeoacremonium	2.333	1.886	0.649	NA
Sphingorhabdus	2.750	1.908	1.200	NA
Bacteroidetes	1.184	1.916	1.826	NA
Trujillella	0.289	1.920	2.370	NA
Chlamydia	0.283	1.929	2.677	NA
Minwuia	0.831	1.935	2.595	NA
Phascolarctobacterium	0.264	1.953	2.909	NA
Aliidiomarina	6.184	1.958	0.554	4.130e-02
Synchytrium	4.292	1.966	1.289	NA
Serpula	8.078	1.968	1.151	4.930e-01
Thioalkalivibrio	1.056	1.974	1.285	NA
Alsobacter	2.441	1.980	1.183	NA
Arsukibacterium	2.520	1.992	1.248	NA
Catellicoccus	0.496	1.992	2.089	NA
Tabrizicola	4.228	1.994	0.823	NA
Rhodospirillum	1.216	2.036	1.288	NA
Fusibacter	1.384	2.048	1.722	NA
Lancefieldella	4.241	2.049	1.205	NA
Millisia	1.115	2.064	1.967	NA
Slackia	0.721	2.064	1.803	NA
Orbilia	13.707	2.094	0.843	2.666e-01
Sagittula	3.462	2.115	1.187	NA
Richelia	0.517	2.124	1.653	NA
Planctomycetes	0.709	2.133	1.666	NA
Starkeya	1.361	2.150	1.150	NA
Ethanoligenens	3.606	2.162	1.948	NA
Snodgrassella	4.153	2.174	1.145	NA
Sphaerisporangium	0.405	2.181	2.595	NA
Emticicia	2.634	2.185	1.391	NA
Microterricola	0.560	2.221	2.181	NA
Hoaglandella	0.398	2.243	2.102	NA
Ezakiella	1.319	2.250	1.801	NA
Frigoriflavimonas	0.492	2.263	1.911	NA
Xinfangfangia	1.289	2.276	1.364	NA
Rickettsia	2.419	2.289	1.286	NA
Riemerella	4.363	2.294	0.856	NA
Pseudoglutamicibacter	1.363	2.333	1.265	NA
Planomicrobium	4.483	2.335	1.436	NA
Halomicroarcula	0.504	2.366	0.897	NA
Kirsten_murine_sarcoma_virus	1.366	2.368	1.522	NA
Aciditerrimonas	0.568	2.386	1.641	NA
Scedosporium	1.160	2.395	1.736	NA
Idiomarina	0.506	2.402	2.553	NA
Odoribacter	0.406	2.411	2.574	NA
Lignipirellula	0.509	2.413	2.556	NA
Truncatella	0.941	2.415	1.399	NA
Mycoavidus	1.599	2.510	1.560	NA
Allostreptomyces	0.466	2.516	2.554	NA
Grosmannia	2.456	2.528	1.465	NA
Parachlamydia	0.456	2.546	2.874	NA
Tissierellia	3.801	2.549	1.093	NA
Botrytis	2.262	2.553	1.024	NA
Segnochrobactrum	1.097	2.590	1.969	NA
Hirsutella	0.614	2.599	2.808	NA
Wolinella	1.486	2.612	1.614	NA
Maridesulfovibrio	0.637	2.634	2.906	NA
Fischerella	0.513	2.683	2.518	NA

Anaerobiospirillum	0.687	2.712	2.231	NA
Subtercola	0.858	2.741	1.738	NA
Plectonema	0.548	2.757	2.906	NA
Marisediminicola	1.015	2.983	2.093	NA
Flavobacteriaceae_genus	0.949	3.053	1.305	NA
Lacipirellula	2.490	3.222	1.522	NA
Gullanella	1.395	3.244	0.899	NA
Microbacteriaceae_genus	4.620	3.320	1.098	NA
Siphonobacter	1.123	3.348	1.978	NA
Dactylellina	2.854	3.434	1.286	NA
Enteractinococcus	1.851	3.458	2.069	NA
Oligella	1.184	3.463	1.973	NA
Panacagrimonas	3.396	3.468	1.294	NA
Vitreoscilla	2.267	3.525	1.400	NA
Negativicoccus	1.337	3.641	2.904	NA
Schneideria	1.354	3.735	1.348	NA
Aliicoccus	2.437	4.058	1.668	NA
Type-D_symbiont_of_Plautia_stali	2.107	4.273	2.903	NA
Mycotypha	24.187	4.330	1.166	4.130e-02
Hubei_permutotetra-like_virus	2.387	4.378	1.506	NA
Blochmannia	14.483	4.997	0.563	1.120e-12
Izhakiella	17.352	5.941	0.684	1.828e-11
