# Supplementary material for: Shotgun Metagenome Analysis of Two Schizaphis graminum Biotypes over Time With and Without Carried Cereal Yellow Dwarf Virus
Source: Insects. 2025 May 23;16(6):554. doi: 10.3390/insects16060554 (PMC12193481; doi:10.3390/insects16060554)
Supplement: Supplementary file 1 [file insects-16-00554-s001.zip › Table S8.pdf]

Table S8. DESeq2 results for comparison by early versus late relative time, arranged by BH-adjusted p-value.

Genus	BaseMean	Log2FC	LFCSE	Padj
Shigella	210548.549	-4.600	0.130	1.309e-146
Escherichia	61277.548	-4.484	0.134	2.384e-134
Citrobacter	14367.687	-4.073	0.160	1.989e-82
Aquabacterium	1382.076	2.297	0.137	9.231e-73
Klebsiella	1531.102	-2.829	0.131	2.295e-72
Terrisporobacter	682.815	-4.547	0.200	2.603e-70
Lamprocystis	7995.423	-4.828	0.201	3.554e-67
Herbaspirillum	591.387	1.852	0.123	1.199e-57
Delftia	511.917	1.747	0.118	9.198e-55
Enterobacter	9658.205	-3.768	0.186	1.803e-54
Oryzomicrobium	63.319	-6.526	0.434	5.465e-54
Pusillimonas	1729.460	-3.261	0.169	9.476e-54
Cedecea	62.532	-5.928	0.409	2.700e-46
Lentisphaera	29.992	-3.778	0.288	6.220e-43
Tannerella	323.303	-3.882	0.231	3.066e-42
Actinobacillus	273.019	-4.205	0.253	1.251e-39
Marinobacterium	1297.016	-4.959	0.275	3.670e-39
Elizabethkingia	205.340	-3.015	0.204	8.493e-36
Leptothrix	258.760	2.859	0.252	3.275e-34
Rheinheimera	4037.545	-2.975	0.196	4.079e-34
Phytophthora	90.681	-6.197	0.440	1.074e-33
Raoultella	68.520	-3.763	0.286	1.435e-30
Musicola	448.106	-4.515	0.304	1.128e-28
Mycolicibacterium	115.168	2.556	0.261	2.715e-25
Bacteroides	124.758	-2.409	0.203	1.089e-24
Craterilacuibacter	127.435	-6.634	0.499	2.151e-24
Burkholderia	2610.408	1.188	0.121	6.774e-24
Marinobacter	150.235	-2.572	0.217	9.748e-24
Comamonas	377.215	1.683	0.175	1.578e-23
Ruficoccus	65.274	-4.028	0.334	1.181e-22
Gleimia	229.791	-4.637	0.366	4.646e-22
Parastagonospora	616.798	5.222	0.580	7.941e-21
Paracidovorax	147.082	2.440	0.277	1.159e-20
Erwinia	905.375	-3.074	0.264	3.106e-20
Labrys	177.824	3.547	0.411	3.314e-20
Ochrobactrum	63.445	2.386	0.274	4.480e-20
Seonamhaeicola	12.679	3.016	0.345	5.189e-20
Moritella	186.504	-1.503	0.148	5.831e-20
Pseudochrobactrum	75.819	3.335	0.391	1.003e-19
Paraburkholderia	685.653	1.187	0.137	1.353e-18
Afipia	1012.669	-2.270	0.215	1.635e-18
Acinetobacter	11818.492	1.130	0.131	1.796e-18
Allomeiothermus	372.615	-7.886	0.603	5.037e-18
Rhizobacter	51.463	2.270	0.274	5.826e-18
Ascochyta	1160.970	5.781	0.685	6.100e-18
Kurthia	83.993	-3.526	0.330	7.456e-18
Fusobacterium	181.144	-1.871	0.192	1.614e-17
Serratia	233.668	-2.344	0.231	1.922e-17
Macroventuria	247.054	5.455	0.669	4.010e-17
Microbispora	62.361	-2.777	0.288	1.028e-16
Piscinibacter	118.721	2.507	0.331	1.735e-15
Facklamia	31.337	-4.277	0.465	2.380e-15
Agrobacterium	192.449	1.329	0.172	6.083e-15
Betaproteobacterium_FWI2	1603.185	-3.177	0.316	7.136e-15

Kallotenue	98.738	-6.624	0.636	1.277e-14
Allomuricauda	54.976	-3.274	0.349	1.305e-14
Rahnella	196.146	-2.689	0.289	3.862e-14
Bradyrhizobium	4376.605	-1.407	0.162	4.729e-14
Armatimonadetes	144.045	-7.737	0.697	9.626e-14
Clostridium	979.039	-1.451	0.171	1.711e-13
Murine_type_C_virus	17.101	3.211	0.464	3.645e-13
Lautropia	241.645	-1.633	0.196	3.884e-13
Spiribacter	38.734	2.745	0.395	4.092e-13
Nitriliruptoraceae_genus	156.786	5.769	0.831	2.294e-12
Beijerinckiaceae_genus	37.739	4.236	0.637	4.490e-12
Cloacibacterium	69.593	-3.752	0.433	6.848e-12
Novosphingobium	579.919	1.983	0.300	8.900e-12
Ichthyophthirius	11.762	-4.898	0.614	9.867e-12
Proteus	179.703	-3.054	0.353	1.116e-11
Azospirillum	271.311	2.179	0.333	1.176e-11
Alteromonas	44.223	3.016	0.470	1.575e-11
Enterobacteriaceae_genus	34.309	-3.251	0.405	2.054e-11
Aerococcus	77.226	-2.279	0.289	2.161e-11
Ensifer	36.688	-2.548	0.330	4.298e-11
Tumebacillus	31.849	-3.440	0.435	5.017e-11
Lysinibacillus	127.976	-3.065	0.373	1.184e-10
Edwardsiella	64.525	-2.811	0.353	1.265e-10
Brevundimonas	1147.754	1.464	0.230	1.267e-10
Rubrivivax	73.057	2.086	0.335	1.737e-10
Diaphorobacter	35.714	-3.212	0.416	2.114e-10
Zoogloea	42.077	-2.850	0.374	2.460e-10
Shewanella	593.636	-1.022	0.143	2.673e-10
Cytobacillus	13.792	6.010	0.970	2.698e-10
Betaproteobacterium_AAP51	11.054	3.136	0.519	4.005e-10
Leptosphaeria	79.063	4.657	0.773	5.707e-10
Mycobacteroides	34.731	3.653	0.616	6.837e-10
Acidithiobacillus	129.947	2.781	0.474	1.084e-09
Bordetella	34.897	2.789	0.476	1.114e-09
Rhodobacter	84.201	-1.692	0.244	1.790e-09
Pelomonas	2109.665	-1.222	0.177	1.974e-09
Morococcus	313.297	-1.992	0.278	2.451e-09
Wolbachia	62.605	-3.111	0.419	2.638e-09
Polyangium	64.134	3.147	0.549	2.684e-09
Fimicolochytrium	46.223	5.905	1.001	4.498e-09
Sinirhodobacter	15.801	1.919	0.329	6.142e-09
Pseudarthrobacter	55.707	2.601	0.461	8.020e-09
Anaerococcus	995.459	-4.919	0.584	8.874e-09
Bacillus	2815.520	-0.545	0.086	9.730e-09
Paucilactobacillus	8.021	-4.454	0.664	9.856e-09
Yersinia	118.324	-3.025	0.412	1.120e-08
Mitsuaria	119.372	1.530	0.270	1.307e-08
Caulobacter	1070.743	-1.605	0.241	1.988e-08
Stenotrophomonas	1057.148	0.644	0.112	3.521e-08
Meira	102.335	5.674	1.022	3.536e-08
Pseudacidovorax	63.479	-2.174	0.330	4.633e-08
Uncultured	130.743	1.987	0.370	5.045e-08
Agrococcus	150.918	3.441	0.659	7.759e-08
Prevotella	338.175	-2.080	0.317	8.113e-08
Limnobacter	140.336	3.150	0.607	9.544e-08
Providencia	883.343	-0.978	0.162	1.320e-07
Sphingopyxis	66.886	2.116	0.406	1.426e-07
Azotobacter	93.627	-3.272	0.485	2.267e-07
Nanosynbacter	12.801	-4.984	0.742	3.101e-07

Dyella	23.540	-2.476	0.403	3.370e-07
Microcystis	6710.710	-2.446	0.378	3.956e-07
Saccharopolyspora	62.086	-1.354	0.235	5.843e-07
Westeberhardia	22.020	-2.886	0.484	8.453e-07
Enterococcus	585.175	-0.775	0.139	8.905e-07
Myceligenerans	6.325	-4.000	0.704	1.111e-06
Thalassiosira	9.266	-4.240	0.696	1.523e-06
Exophiala	38.352	2.937	0.623	1.693e-06
Polychytrium	132.053	4.570	0.962	1.818e-06
Mycotypha	40.609	5.599	1.150	1.955e-06
Neisseria	439.766	-1.240	0.222	2.029e-06
Kosakonia	21.067	-2.470	0.437	2.239e-06
Perkinsus	5.227	-3.812	0.705	2.488e-06
Aureibaculum	51.891	-2.339	0.406	3.243e-06
Didymella	21.350	4.526	0.991	4.617e-06
Salinimicrobium	20.796	2.230	0.485	5.522e-06
Frankia	114.940	-1.696	0.309	5.535e-06
Methylobacterium	5697.290	-1.014	0.191	5.928e-06
Paracoccus	735.536	0.844	0.180	8.826e-06
Dermacoccus	44.902	1.462	0.322	9.299e-06
Finegoldia	162.375	-4.092	0.656	9.412e-06
Cupidesulfovibrio	28.579	-2.476	0.452	1.083e-05
Selenomonas	6.804	-3.421	0.673	1.150e-05
Mycetohabitans	9.753	2.081	0.468	1.380e-05
Glycocaulis	61.217	4.790	1.097	1.527e-05
Desulfosporosinus	5.604	-3.959	0.776	1.527e-05
Acaromyces	28.763	3.917	0.916	1.835e-05
Heterobasidion	23.873	-5.876	0.973	1.919e-05
Alkalihalobacillus	4.260	2.582	0.606	1.941e-05
Azospira	27.304	-1.810	0.358	2.075e-05
Thermus	128.332	-2.411	0.446	2.227e-05
Roseomonas	144.562	1.801	0.417	2.227e-05
Lentilactobacillus	33.253	4.814	1.121	2.295e-05
Melampsora	15.381	5.638	1.281	2.295e-05
Pauljensenia	148.330	-2.603	0.474	2.342e-05
Tetrahymena	5.684	-3.938	0.789	2.425e-05
Lichtheimia	16.959	1.704	0.396	3.026e-05
Kineococcus	53.880	2.971	0.713	3.080e-05
Lobosporangium	154.598	5.214	1.224	3.122e-05
Seramator	7.459	-4.362	0.851	3.164e-05
Phaeodactylum	12.627	-3.165	0.628	3.890e-05
Blastomonas	151.122	-1.582	0.317	3.930e-05
Yarrowia	13.249	3.234	0.791	4.245e-05
Naegleria	20.303	-3.099	0.578	5.061e-05
Janthinobacterium	149.394	1.111	0.261	5.456e-05
Micrococcus	532.483	1.227	0.293	6.212e-05
Postia	14.857	-5.275	0.981	6.212e-05
Picosynechococcus	8.815	2.974	0.729	6.318e-05
Hafnia	2.018	-2.468	0.609	6.374e-05
Kitasatospora	19.546	-3.300	0.647	6.450e-05
Propionimicrobium	84.480	-2.953	0.560	6.536e-05
Nitrosospira	14.651	6.210	1.441	6.617e-05
Xenorhabdus	3.486	-2.361	0.560	7.713e-05
Lachnoanaerobaculum	14.139	-2.297	0.482	7.905e-05
Pseudoroseomonas	11.878	2.480	0.620	8.720e-05
Sedimentitalea	30.138	1.198	0.289	9.380e-05
Chelativorans	9.704	-4.726	0.944	9.610e-05
Neofusicoccum	58.401	2.112	0.534	1.005e-04
Apibacter	4.693	3.828	1.008	1.351e-04

Alcaligenes	8.410	-3.416	0.728	1.351e-04
Hyphobacterium	32.142	1.317	0.327	1.424e-04
Belnapia	18.847	2.543	0.654	1.466e-04
Bipolaris	9.251	-4.524	0.934	1.484e-04
Trematosphaeria	6.493	3.804	1.002	1.580e-04
Secondary	3.884	-2.621	0.600	1.774e-04
Glarea	5.933	-3.760	0.838	1.893e-04
Acetobacter	16.394	1.425	0.363	1.993e-04
Stomatobaculum	6.438	-4.135	0.908	2.066e-04
Fibrisoma	27.200	-3.691	0.660	2.090e-04
Neoantrodia	12.936	-5.135	1.035	2.090e-04
PreXMRV-1_provirus_complete	4.785	2.008	0.518	2.090e-04
Dysgonamonadaceae_genus	19.860	-4.807	0.890	2.111e-04
Aphanomyces	5.189	-3.157	0.718	2.154e-04
Human_adenovirus_2	5.562	-3.892	0.878	2.159e-04
Desulfitobacterium	6.033	-4.029	0.902	2.389e-04
Pyrinomonas	9.505	-4.692	0.996	2.672e-04
Phytoplasma	62.472	-1.469	0.334	2.803e-04
Pectobacterium	3.282	-2.957	0.727	2.803e-04
Mycena	39.124	3.208	0.873	2.848e-04
Trichosporon	145.713	2.937	0.805	3.150e-04
Wickerhamiella	9.411	2.739	0.750	3.150e-04
Trametes	13.922	-4.291	0.899	3.655e-04
Sodalis	1.787	-1.930	0.544	3.863e-04
Alloscardovia	56.324	-2.026	0.456	3.887e-04
Pseudoramibacter	88.151	-2.650	0.570	4.228e-04
Salmonella	451.456	-0.733	0.178	4.304e-04
Salipaludibacillus	45.569	-2.411	0.539	4.841e-04
Porphyromonadaceae_genus	10.907	-3.178	0.726	5.020e-04
Pantoea	249.985	-0.828	0.201	5.054e-04
Paenimyroides	6.925	3.580	0.972	5.979e-04
Peptoniphilus	189.828	-2.677	0.583	5.992e-04
Chromohalobacter	65.014	-0.967	0.240	6.218e-04
Massilia	397.055	1.143	0.314	6.813e-04
Kocuria	405.951	1.547	0.435	7.336e-04
Gemella	167.060	-2.010	0.464	7.336e-04
Rhodofomes	5.979	-4.033	0.975	7.783e-04
Polaribacter	15.580	-2.433	0.584	8.207e-04
Clostridiales	35.068	1.495	0.424	8.288e-04
Gloeophyllum	12.295	-5.057	1.129	9.261e-04
Paludibacterium	3.956	-3.439	0.896	9.465e-04
Letharia	140.804	2.247	0.658	9.629e-04
Levilactobacillus	84.614	-2.264	0.530	9.921e-04
Lutimaribacter	14.713	-2.868	0.654	1.005e-03
Clavispora	11.624	5.259	1.419	1.028e-03
Ornithinimicrobium	139.539	1.735	0.505	1.059e-03
Aureococcus	5.453	-3.896	0.977	1.059e-03
Luteibacter	4.401	-3.605	0.932	1.059e-03
Bosea	160.841	0.753	0.209	1.062e-03
Phytobacter	11.988	-2.676	0.553	1.110e-03
Phaeosphaeria	3.618	4.650	1.421	1.152e-03
Saprolegnia	9.754	-2.973	0.734	1.332e-03
Microdochium	3.486	-3.248	0.888	1.348e-03
Bacterium	36.502	0.932	0.264	1.359e-03
Pestalotiopsis	18.985	-2.439	0.607	1.364e-03
Reyranella	10.238	-2.712	0.682	1.384e-03
Epilithonimonas	41.936	1.337	0.399	1.644e-03
Marinifilum	38.998	0.977	0.287	1.658e-03
Pseudomicrostroma	29.079	2.952	0.919	1.850e-03

Undibacterium	178.266	1.250	0.373	1.897e-03
Roseateles	324.440	0.653	0.191	1.963e-03
Leptolyngbya	41.961	-1.809	0.464	2.016e-03
Deinococcus	166.493	0.586	0.172	2.115e-03
Boeremia	20.842	2.856	0.900	2.140e-03
Gamsiella	9.097	3.672	1.170	2.241e-03
Propionibacterium	4137.128	0.870	0.262	2.446e-03
Microbacterium	1220.119	-0.545	0.151	2.463e-03
Gallibacter	20.094	-4.046	0.970	2.463e-03
Aureispira	3.069	-3.076	0.903	2.514e-03
Empedobacter	31.761	1.336	0.410	2.517e-03
Corallococcus	123.324	0.969	0.295	2.611e-03
Protomyces	16.751	3.244	1.043	2.626e-03
Zychaea	9.011	4.159	1.346	2.626e-03
Pectinatus	4.713	-3.200	0.825	2.730e-03
Lawsonella	200.793	-1.732	0.455	3.009e-03
Enterobacteria_phage_phi80_virus	4.380	-3.563	1.015	3.291e-03
Aquamicrobium	62.955	-1.427	0.376	3.350e-03
Spizellomyces	92.702	3.735	1.226	3.446e-03
Thermomicrobium	3.852	-3.384	0.987	3.446e-03
Caballeronia	39.742	0.830	0.257	3.465e-03
Hymenobacter	123.295	1.164	0.368	3.598e-03
Fonsecaea	17.304	1.988	0.645	3.649e-03
Clavibacter	5.527	-3.912	1.087	3.649e-03
Shinella	19.684	1.847	0.595	3.708e-03
Caldibacillus	3.114	-3.112	0.947	3.753e-03
Roseovarius	20.641	1.249	0.394	3.768e-03
Micromonospora	101.944	-1.871	0.503	3.906e-03
Mitosporidium	133.751	4.335	1.433	3.928e-03
Pseudonocardia	81.981	1.344	0.431	3.962e-03
Murine_osteosarcoma_virus	2.353	2.203	0.712	3.962e-03
Vibrio	744.207	0.928	0.293	3.974e-03
Microcoleus	16.615	1.878	0.606	3.974e-03
Thermalbibacter	6.902	-4.229	1.150	3.974e-03
Aliiruegeria	16.063	-2.472	0.673	4.138e-03
Saitoella	68.439	4.718	1.562	4.202e-03
Aureimonas	21.201	-2.089	0.575	4.254e-03
Rummeliibacillus	3.322	-3.156	0.967	4.254e-03
Emergencia	2.121	3.828	1.365	4.254e-03
Desulfocarbo	2.982	2.870	0.973	4.531e-03
Mus_musculus_mobilized_virus	23.218	1.128	0.362	4.596e-03
Anaeroglobus	4.318	-3.239	0.905	4.627e-03
Erythrobacter	135.550	0.802	0.254	4.763e-03
Leptospira	69.642	-1.102	0.317	4.854e-03
Corynebacterium	2249.564	-1.029	0.297	4.894e-03
Georgenia	10.823	2.099	0.702	4.922e-03
Dorea	12.831	-2.250	0.644	4.988e-03
Sugiyamaella	2.101	3.466	1.237	5.043e-03
Planococcus	196.283	-0.750	0.220	5.162e-03
Phanerochaete	10.953	-3.524	0.955	5.317e-03
Dietzia	166.910	1.119	0.369	5.535e-03
Roseibacterium	113.257	-1.884	0.520	5.648e-03
Protofrankia	75.565	-1.616	0.457	5.740e-03
Laccaria	4.694	-3.192	0.880	6.312e-03
Ruminococcus	45.967	-2.299	0.632	6.402e-03
Synchytrium	3.426	3.820	1.362	6.608e-03
Hallella	3.747	-3.288	1.041	7.022e-03
Levyella	9.603	-4.638	1.290	7.455e-03
Cryptosporidium	15.505	-2.197	0.583	7.675e-03

Gilbertella	13763.122	0.776	0.261	7.918e-03
Dioszegia	14.284	-3.974	1.088	7.918e-03
Wenzhouxiangella	3.178	-2.946	0.955	7.920e-03
Delta	4.266	1.854	0.652	8.152e-03
Oceanobacillus	36.670	0.946	0.321	8.313e-03
Saccharibacteria	4.408	-3.593	1.132	8.721e-03
Plasmodium	260.330	0.557	0.187	8.900e-03
Cutaneotrichosporon	11.862	-3.639	1.030	8.924e-03
Putridiphycobacter	3.734	-2.963	0.976	9.023e-03
Pedobacter	122.644	-0.984	0.307	9.111e-03
Rubellimicrobium	30.281	1.696	0.598	9.320e-03
Haematobacter	4.763	3.455	1.244	9.852e-03
Betaproteobacterium_JGI	8.316	1.337	0.473	1.001e-02
Oceanimonas	1.818	-2.128	0.805	1.065e-02
Rhodobacteraceae_genus	44.452	1.478	0.534	1.105e-02
Leifsonia	76.614	1.200	0.429	1.120e-02
Schlegelella	25.036	1.347	0.486	1.160e-02
Rhizophagus	4.450	2.903	1.109	1.160e-02
Punctularia	16.189	-4.794	1.082	1.164e-02
Coraliihabitans	13.216	-2.554	0.785	1.186e-02
Parainfluenza_virus_5	6.913	-4.210	1.295	1.220e-02
Phreatobacter	2.827	-2.890	1.031	1.221e-02
Nodosilinea	25.845	2.307	0.865	1.231e-02
Mucor	14.971	2.466	0.922	1.246e-02
Serpula	14.437	-4.749	1.317	1.259e-02
Guillardia	48.159	3.066	1.162	1.268e-02
Phyllobacterium	39.527	1.625	0.599	1.268e-02
Qipengyuania	66.264	-1.550	0.486	1.317e-02
Williamsia	113.724	-1.649	0.512	1.335e-02
Oceanospirillum	5.544	-2.545	0.786	1.335e-02
Granulicatella	30.965	-1.721	0.536	1.349e-02
Lachnospiraceae_genus	22.274	-1.808	0.527	1.349e-02
Dialister	4.741	-3.329	1.115	1.365e-02
Aspergillus	116.582	-1.212	0.390	1.384e-02
Eikenella	8.680	-2.077	0.673	1.428e-02
Enterobacteria_phage_RTP_virus	2.620	-2.856	1.050	1.498e-02
Spleen_focus-forming_virus	3.983	1.416	0.527	1.557e-02
Abelson	3.843	1.419	0.535	1.560e-02
Leptotrichia	40.411	-1.167	0.386	1.573e-02
Rhabdonatronobacter	170.476	-0.821	0.277	1.624e-02
Pararhizobium	8.228	-2.317	0.762	1.624e-02
Planomicrobium	4.483	4.526	1.491	1.624e-02
Domibacillus	6.862	-4.216	1.356	1.708e-02
Homoserinimonas	5.839	-2.840	0.887	1.786e-02
Entamoeba	57.649	-1.958	0.624	1.797e-02
Serinicoccus	17.817	2.235	0.878	1.845e-02
Acidihalobacter	28.945	-1.887	0.608	1.892e-02
Saccharothrix	13.589	1.571	0.616	1.930e-02
Lachnospira	17.322	1.351	0.523	1.972e-02
Neomicrococcus	4.911	-2.317	0.815	1.979e-02
Colletotrichum	43.728	-1.172	0.402	2.000e-02
Neohortaea	3.383	-3.204	1.175	2.000e-02
Babesia	5.122	-2.107	0.756	2.044e-02
Dictyostelium	8.462	-3.153	0.951	2.142e-02
Kirsten_murine_sarcoma_virus	1.366	3.750	1.644	2.206e-02
Eutypa	3.140	-2.717	0.996	2.209e-02
Burkholderiaceae_genus	42.128	-1.130	0.394	2.226e-02
Paraferrimonas	4.903	-2.179	0.786	2.258e-02
Pleionea	5.060	-2.563	0.917	2.269e-02

Staphylococcus	2266.839	-1.016	0.352	2.289e-02
Caldifermentibacillus	6.086	-3.730	1.239	2.302e-02
Pimelobacter	6.125	-2.605	0.838	2.302e-02
Lentibacillus	34.801	-0.885	0.313	2.350e-02
Acidovorax	744.728	0.326	0.123	2.358e-02
Sulfolobus	2.237	1.335	0.512	2.360e-02
Beutenbergia	4.200	2.916	1.197	2.393e-02
Talaromyces	10.629	-2.386	0.825	2.421e-02
Thiolapillus	14.428	-2.728	0.850	2.431e-02
Mogibacterium	9.735	-2.222	0.744	2.431e-02
Minimicrobia	2.704	-2.880	1.132	2.431e-02
Methylocystis	2.390	-2.570	1.013	2.431e-02
Rhodococcus	464.902	-0.575	0.207	2.459e-02
Toxoplasma	250.863	0.446	0.172	2.466e-02
Plasmopara	6.866	-2.287	0.819	2.473e-02
Propioniciclava	26.542	-1.946	0.665	2.532e-02
Aliidiomarina	6.184	-1.922	0.718	2.532e-02
FBR_murine_osteosarcoma_virus	1.393	1.749	0.674	2.585e-02
Acidaminobacter	5.035	-2.320	0.753	2.644e-02
Cellvibrio	9.111	2.079	0.857	2.688e-02
Mannheimia	5.616	-1.716	0.649	2.711e-02
Flavobacterium	706.963	-0.472	0.176	2.853e-02
Pelorhabdus	4.813	-2.559	0.848	2.853e-02
Fannyhessea	5.504	-3.754	1.332	2.973e-02
Penaeicola	1.825	2.421	0.966	2.977e-02
Escherichia_phage_phiV10_virus	2.464	-2.729	1.139	3.103e-02
Alloprevotella	38.025	-1.487	0.535	3.146e-02
Ruminococcaceae_genus	14.329	1.044	0.426	3.146e-02
Robertmurraya	9.332	-2.021	0.743	3.146e-02
Rhizoctonia	10.173	-2.768	0.973	3.160e-02
Salinibacterium	8.132	-1.986	0.741	3.232e-02
Isoptericola	31.305	0.678	0.273	3.238e-02
Amaricoccus	20.347	1.494	0.628	3.268e-02
Pasteurella	1.606	-1.703	0.785	3.334e-02
Aurantimonas	8.757	-1.987	0.657	3.387e-02
Acuticoccus	38.672	0.708	0.289	3.397e-02
Xanthomonadaceae_genus	3.316	-2.558	1.037	3.399e-02
Aggregatibacter	18.736	-1.344	0.509	3.503e-02
Frigidibacter	67.900	0.780	0.321	3.545e-02
Desarmillaria	3.332	-2.378	0.976	3.595e-02
Proteobacteria	45.108	1.878	0.814	3.595e-02
Singulisphaera	6.434	-2.791	1.044	3.769e-02
Azomonas	2.339	-2.359	1.022	3.948e-02
Seohaeicola	2.294	3.032	1.386	3.965e-02
Halovibrio	10.136	-2.393	0.740	4.029e-02
Luteolibacter	24.040	-1.334	0.476	4.043e-02
Gaiella	11.733	-3.141	1.120	4.043e-02
Brevibacterium	83.680	0.897	0.382	4.143e-02
Lactococcus	112.493	-1.220	0.460	4.143e-02
Tepidicella	11.802	1.864	0.823	4.143e-02
Algoriphagus	22.246	0.883	0.378	4.198e-02
Enterobacteria_phage_T4_virus	2.981	-3.015	1.279	4.221e-02
Drepanopeziza	2.702	-2.884	1.246	4.253e-02
Sagittula	3.462	2.904	1.336	4.351e-02
Trichophyton	4.702	3.149	1.458	4.369e-02
Alcanivorax	267.141	0.619	0.263	4.388e-02
Parvimonas	4.539	-2.359	0.959	4.392e-02
Solobacterium	7.141	-2.004	0.807	4.497e-02
Stereum	22.521	-2.545	0.916	4.519e-02

TM7	7.135	-2.064	0.771	4.519e-02
Schaalia	24.329	-1.407	0.546	4.530e-02
Leptomonas	2.477	-2.765	1.230	4.530e-02
Okeania	8.897	-2.378	0.923	4.590e-02
Siccirubricoccus	2.442	3.057	1.465	4.592e-02
Paracoccidioides	3.608	-2.361	0.994	4.769e-02
Prevotellaceae_genus	6.860	-1.983	0.810	4.927e-02
Pleomorphomonas	5.725	-3.315	1.306	5.099e-02
Dothidotthia	2.037	2.748	1.298	5.099e-02
Haladaptatus	2.468	-2.087	0.933	5.102e-02
Adhaeribacter	6.285	2.667	1.253	5.182e-02
Simian_virus_40	1.423	-1.824	0.975	5.213e-02
Chrysosporum	12.790	-2.214	0.846	5.312e-02
Rhodoferax	44.739	0.823	0.365	5.365e-02
Marichromatium	2.010	-1.513	0.730	5.394e-02
Trypanosoma	18.124	1.456	0.667	5.441e-02
Exserohilum	3.437	-3.226	1.403	5.442e-02
Alicycliphilus	12.250	-1.330	0.539	5.511e-02
Alloalcanivorax	2.215	-2.454	1.163	5.547e-02
Lactobacillus	101.572	0.516	0.229	5.607e-02
Hydrogenophaga	66.646	-1.100	0.443	5.699e-02
Glaciibacter	16.545	-2.167	0.756	6.022e-02
Alkalispirochaeta	9160.805	-0.640	0.271	6.393e-02
Westerdykella	3.310	2.028	0.993	6.422e-02
Sphingomonadaceae_genus	2.729	3.012	1.553	6.704e-02
Thalassotalea	1.950	-2.258	1.101	7.070e-02
Vulcaniibacterium	1.978	3.403	1.755	7.329e-02
Methylosinus	1.999	-2.487	1.264	7.382e-02
Algiphilus	5.689	-2.355	1.023	7.414e-02
Photobacterium	11.366	-1.337	0.572	7.414e-02
Insolitispirillum	2.216	-2.578	1.294	7.505e-02
Pseudomonas	20853.250	-0.538	0.238	7.681e-02
Turicibacter	17.622	-3.478	1.379	7.701e-02
Tuber	2.943	-2.948	1.430	7.744e-02
Halalkalibacterium	3.247	-2.290	1.025	7.760e-02
Thermicanus	19.990	-2.673	1.080	7.812e-02
Human_endogenous_retrovirus	5.191	1.254	0.613	7.972e-02
Curtobacterium	171.096	0.910	0.440	8.038e-02
Fibroporia	7.444	-3.076	1.288	8.094e-02
Rosenbergiella	1.613	-1.559	0.839	8.150e-02
Niastella	47.268	2.684	1.379	8.196e-02
Winkia	9.874	-2.071	0.901	8.324e-02
Aphanizomenon	21.520	0.634	0.307	8.329e-02
Brachybacterium	121.244	0.952	0.468	8.371e-02
Paludisphaera	5.695	-2.377	1.066	8.431e-02
Agarivorans	68.212	-1.514	0.641	8.461e-02
Psilocybe	1.438	-1.857	1.023	8.535e-02
Loigolactobacillus	19.382	-2.883	1.130	8.693e-02
Cadophora	1.997	-2.459	1.303	8.693e-02
Methylomonas	1.727	1.414	0.673	8.711e-02
Gammaproteobacteria	3.436	-2.457	1.148	8.828e-02
Moorena	7.922	-2.188	0.988	8.828e-02
Rubrobacter	16.449	-1.720	0.746	8.840e-02
Atlantibacter	14.246	0.642	0.312	8.931e-02
Abyssicoccus	5.009	2.397	1.266	9.232e-02
Hahella	2.380	-1.981	0.893	9.258e-02
Aequitasia	117.988	2.254	1.187	9.366e-02
Virgibacillus	35.407	-0.676	0.320	9.469e-02
Microsporum	6.726	2.238	1.188	9.505e-02

Halomonas	148.408	-0.633	0.293	9.540e-02
Duffyella	4.145	-2.014	0.892	9.559e-02
Conyzicola	2.118	-2.550	1.371	9.607e-02
Suillus	38.317	2.109	1.117	9.629e-02
Chromobacterium	7.322	-1.307	0.618	9.629e-02
Enterobacteria_phage_vB_EcoS_IME542_virus	2.043	-2.065	1.183	9.647e-02
Atopobium	11.220	-1.751	0.759	9.663e-02
Herbinix	3.656	-3.300	1.613	9.664e-02
Porphyromonas	64.376	-0.991	0.454	9.770e-02
Panacagrimonas	4.713	-3.517	1.597	9.770e-02
Schizophyllum	63.317	1.893	1.007	9.958e-02
Meiothermus	30.189	-1.453	0.655	9.958e-02
Bacidia	3.442	-2.132	1.080	9.964e-02
Chelatococcus	6.249	-2.359	1.101	1.006e-01
Actinoplanes	35.115	0.822	0.420	1.011e-01
Mollisia	4.514	-1.926	0.942	1.011e-01
Moniliophthora	4.949	-2.206	1.064	1.011e-01
Vogesella	4.180	-2.254	1.092	1.014e-01
Fomitiporia	9.334	-2.176	0.939	1.018e-01
Thermothelomyces	6.239	-3.906	1.753	1.018e-01
Xanthocytophaga	2.896	-2.531	1.338	1.020e-01
Methanotrophic	6.831	-1.952	0.939	1.025e-01
Tissierellia	8.196	-3.197	1.411	1.035e-01
Legionella	1332.136	-0.572	0.274	1.042e-01
Paeniglutamicibacter	17.218	0.847	0.433	1.042e-01
Apiotrichum	11.988	-1.961	0.913	1.047e-01
Collinsella	10.764	-2.822	0.982	1.061e-01
Gemmatimonas	2.580	-2.739	1.505	1.084e-01
Sphingobium	158.875	-0.463	0.226	1.101e-01
Leclercia	58.127	0.610	0.317	1.101e-01
Tepidiforma	3.376	2.745	1.571	1.101e-01
Microbacteriaceae_genus	2.390	-2.552	1.362	1.103e-01
Faecalibacterium	8.180	-1.977	0.954	1.115e-01
Gayadomonas	2.348	-1.206	0.672	1.115e-01
Aquisphaera	2.365	-2.691	1.486	1.119e-01
Glutamicibacter	45.516	-1.114	0.530	1.121e-01
Magnetospirillum	1.714	-1.870	1.116	1.126e-01
Enterobacteria_phage_vB_EcoS_ACG-M12_virus	1.666	-2.177	1.311	1.141e-01
Streptococcus	1476.802	-0.696	0.339	1.145e-01
Aquabacter	1.821	-2.339	1.375	1.169e-01
Acaricomes	2.882	1.760	0.978	1.170e-01
UNVERIFIED_CONTAM:	2.089	-2.506	1.442	1.183e-01
Sanguibacter	2.037	-2.494	1.443	1.191e-01
Candida	5.852	1.331	0.719	1.197e-01
Afifella	1.377	3.070	1.936	1.201e-01
Tychonema	1.638	-2.118	1.318	1.205e-01
Rhodotorula	32.855	-1.355	0.645	1.214e-01
Thalassobius	5.691	1.511	0.844	1.214e-01
Sphaerulina	10.884	1.391	0.778	1.246e-01
Maribellus	40.625	-0.806	0.406	1.261e-01
Duganella	59.734	-0.850	0.425	1.268e-01
Comamonadaceae_genus	99.630	0.507	0.274	1.271e-01
Metabacillus	4.195	2.109	1.209	1.294e-01
Gullanella	1.395	2.032	1.128	1.299e-01
Mycoplasmopsis	1.529	-1.963	1.291	1.307e-01
Veillonella	194.159	-0.765	0.387	1.320e-01
Kalmanozyma	5.083	1.615	0.932	1.320e-01
Alkaliphilus	2.898	-2.969	1.659	1.320e-01

Anaeromyxobacter	2.178	-2.435	1.445	1.366e-01
Plantibacter	3.609	-2.076	1.123	1.369e-01
Aedoeadaptatus	2.336	-2.673	1.574	1.379e-01
Sphingobacterium	129.321	-0.695	0.357	1.386e-01
Salipiger	55.657	0.609	0.340	1.391e-01
Marmoricola	34.294	1.099	0.625	1.393e-01
Geobacillus	32.342	1.269	0.727	1.393e-01
Coniophora	9.959	1.961	1.154	1.393e-01
Proteus_phage_VB_PmiS-Isfahan_virus	1.743	-1.010	0.673	1.393e-01
Rhodopirellula	20.297	-0.971	0.503	1.396e-01
Leyella	1.814	-2.336	1.454	1.396e-01
Rathayibacter	27.104	-1.505	0.749	1.412e-01
Amycolatopsis	5.663	-1.212	0.616	1.412e-01
Emticicia	2.634	-2.843	1.654	1.414e-01
Capillimicrobium	2.546	2.353	1.416	1.421e-01
Acidisphaera	2.480	-2.765	1.634	1.434e-01
Rothia	240.951	-0.563	0.297	1.438e-01
Arsukibacterium	2.520	-2.122	1.478	1.438e-01
Erythrobacteraceae_genus	1.717	2.356	1.422	1.438e-01
Gellertiella	2.945	2.737	1.700	1.438e-01
Mediterraneibacter	9.994	0.887	0.499	1.443e-01
Aureobasidium	16.662	-1.496	0.735	1.451e-01
Desemzia	16.346	-1.232	0.606	1.506e-01
Batrachochytrium	121.131	3.104	1.903	1.513e-01
Cryptomonas	1.703	-2.101	1.440	1.545e-01
Modestobacter	77.884	-0.590	0.317	1.547e-01
Hyaloscypha	3.026	-1.751	1.036	1.547e-01
Terrabacter	109.045	0.480	0.273	1.549e-01
Brevibacillus	1.944	-1.728	1.108	1.554e-01
Arcticiflavibacter	197.563	0.648	0.374	1.557e-01
Burkholderiales	19.138	0.847	0.492	1.601e-01
Flavisolibacter	1.622	-2.138	1.457	1.646e-01
Tsukamurella	6.889	2.469	1.412	1.646e-01
Nannochloropsis	1.981	-2.279	1.449	1.677e-01
Brachymonas	13.279	0.862	0.515	1.689e-01
Friedmanniella	3.135	2.089	1.319	1.692e-01
Kineosporia	6.745	1.846	1.156	1.714e-01
Agilicoccus	70.829	-1.115	0.597	1.715e-01
Hammondia	7.312	-0.991	0.578	1.715e-01
Winogradskyella	5.235	0.710	0.448	1.716e-01
Zymoseptoria	45.638	0.786	0.475	1.718e-01
Lichenibacterium	2.044	-2.470	1.617	1.718e-01
Sulfitobacter	6.435	0.900	0.536	1.722e-01
Caldilinea	1.616	2.906	1.893	1.735e-01
Mixia	2.238	2.087	1.332	1.740e-01
Rhodomicrobium	7.805	0.884	0.534	1.740e-01
Grosmannia	2.991	-2.867	1.712	1.768e-01
Streptosporangium	49.775	0.460	0.276	1.781e-01
Pneumocystis	8.386	2.162	1.387	1.783e-01
Paecilomyces	3.711	-1.756	1.062	1.783e-01
Gloeocapsa	4.503	-2.333	1.147	1.786e-01
Coleofasciculus	1.811	-2.175	1.448	1.802e-01
Sporisorium	1.536	-2.099	1.487	1.831e-01
Nitrosomonas	5.354	1.431	0.914	1.845e-01
Melaminivora	1.837	-2.348	1.608	1.845e-01
Limimaricola	3.153	2.410	1.524	1.925e-01
Labilibaculum	7.087	0.914	0.569	1.925e-01
Peptostreptococcus	6.314	-1.315	0.804	1.936e-01
Bacteriovorax	1.730	-2.248	1.594	1.936e-01

Methylovulum	39.553	0.872	0.547	1.940e-01
Buchnera	70221.134	-0.363	0.214	1.954e-01
Chromatium	57.450	0.393	0.243	1.987e-01
Haematomicrobium	1.630	3.955	2.278	1.999e-01
Fluviicola	6.799	-1.632	0.989	2.020e-01
Endosymbiont	5.833	-1.121	0.698	2.032e-01
Pectobacterium_phage_CBB_virus	1.674	-2.223	1.609	2.039e-01
Terrimonas	56.451	0.948	0.612	2.039e-01
Filobasidium	15.320	-1.050	0.585	2.045e-01
Oribacterium	16.999	-1.200	0.698	2.050e-01
Trichoderma	47.821	-1.394	0.787	2.055e-01
Jatrophihabitans	10.131	-2.278	1.233	2.055e-01
Naasia	3.443	-1.981	1.284	2.055e-01
Methylomicrobium	16.552	1.010	0.652	2.055e-01
Propionibacteriaceae_genus	1.513	-2.096	1.559	2.055e-01
Embleya	1.575	2.124	1.606	2.055e-01
Dyadobacter	16.940	0.828	0.540	2.069e-01
Botrytis	2.262	1.711	1.236	2.078e-01
Nocardia	68.412	0.416	0.263	2.092e-01
Endobacter	3.493	1.923	1.347	2.094e-01
Wickerhamomyces	2.767	2.569	1.616	2.094e-01
Qaidamihabitans	4.779	-2.160	1.335	2.122e-01
Brasilonema	1.716	-2.261	1.662	2.122e-01
Citromicrobium	6.385	-1.562	0.975	2.124e-01
Tautonia	3.902	-2.904	1.850	2.135e-01
Brucella	13.265	0.769	0.503	2.135e-01
Chroococcidiopsis	2.325	-1.605	1.055	2.146e-01
Alpha	67.476	0.512	0.330	2.155e-01
Conexibacter	13.570	-1.479	0.854	2.155e-01
Psychromicrobium	11.336	-0.611	0.371	2.155e-01
Sordaria	1.433	-1.766	1.383	2.177e-01
Rhizopus	1.764	-2.288	1.697	2.180e-01
Jeotgalicoccus	20.422	0.664	0.436	2.205e-01
Extensimonas	2.701	-1.831	1.176	2.207e-01
Tamlana	6.081	0.786	0.529	2.215e-01
Salinispora	1.822	2.370	1.550	2.228e-01
Halophilic	1.709	-2.228	1.694	2.254e-01
Orbilia	13.707	-1.708	1.029	2.262e-01
Moraxella	437.707	-0.461	0.288	2.270e-01
Paraglaciecola	2.599	1.124	0.743	2.286e-01
Castellaniella	1.632	-2.173	1.674	2.289e-01
Peredibacter	28.482	-1.100	0.673	2.318e-01
Pseudoflavonifractor	1.639	-1.582	1.172	2.318e-01
Pseudorhodoferax	8.912	1.093	0.750	2.326e-01
Riemerella	4.363	-1.581	1.072	2.375e-01
Gluconacetobacter	6.748	-1.024	0.689	2.395e-01
Luteitalea	11.215	-1.768	1.108	2.400e-01
Eremococcus	4.175	-1.494	1.040	2.403e-01
Tardiphaga	3.851	-1.266	0.892	2.439e-01
Actinobacteria	1.870	-2.375	1.838	2.482e-01
Auraticoccus	1.587	2.819	2.255	2.482e-01
Coniosporium	2.486	2.183	1.623	2.506e-01
Actinophytocola	1.747	-2.266	1.791	2.513e-01
Amorphotheca	7.049	-1.205	0.819	2.516e-01
Ethanoligenens	3.764	-3.344	2.288	2.536e-01
Marinomonas	8.179	-1.669	1.100	2.536e-01
Sinorhizobium	5.189	1.277	0.923	2.536e-01
Romboutsia	3.047	1.248	0.839	2.536e-01
Paenirhodobacter	23.041	-0.637	0.421	2.550e-01

Rhodoplanes	8.916	-0.982	0.666	2.578e-01
Kribbella	6.342	0.908	0.657	2.643e-01
Daldinia	4.862	-1.766	1.236	2.646e-01
Paramagnetospirillum	1.385	-1.959	1.669	2.654e-01
Amnimonas	9.718	1.713	1.275	2.666e-01
Leishmania	4.184	-1.817	1.158	2.686e-01
Salinicoccus	15.586	1.018	0.739	2.688e-01
Motilimonas	7.460	-0.955	0.646	2.688e-01
Microlunatus	62.692	0.669	0.477	2.689e-01
Wallemia	78.999	1.607	1.203	2.691e-01
Halochromatium	4.224	1.033	0.745	2.695e-01
Hemiselmis	1.714	-2.247	1.852	2.706e-01
Pseudaminobacter	1.415	1.885	1.732	2.706e-01
Sandarakinorhabdus	1.376	2.679	2.118	2.728e-01
Pseudogymnoascus	6.848	-1.653	1.150	2.746e-01
Zhihengliuella	1.833	2.186	1.682	2.778e-01
Methylotenera	10.684	-1.233	0.759	2.785e-01
Halorubrum	12.131	0.673	0.498	2.812e-01
Acidipropionibacterium	30.097	-1.078	0.723	2.815e-01
Pelagivirga	2.684	1.037	0.722	2.815e-01
Oceanibium	5.401	1.018	0.762	2.816e-01
Geodermatophilaceae_genus	1.390	-1.932	1.732	2.816e-01
Sparassis	5.100	-1.221	0.896	2.818e-01
Luteimonas	79.273	-0.926	0.622	2.819e-01
Phenylobacterium	59.083	-0.603	0.411	2.819e-01
Bavariicoccus	65.404	-0.543	0.381	2.918e-01
Sporosarcina	3.898	-1.145	0.891	2.920e-01
Yonghaparkia	1.392	-1.813	1.579	2.938e-01
Halobacteriovorax	2.262	-0.844	0.755	2.938e-01
Brettanomyces	5.223	2.100	1.687	2.947e-01
Rhabdothermincola	8.157	1.264	0.985	2.954e-01
Hoylesella	13.883	-0.912	0.650	2.991e-01
Methylibium	8.094	1.204	0.934	2.991e-01
Mangrovibacillus	2.576	-1.693	1.427	2.991e-01
Dolosigranulum	11.557	1.022	0.788	3.000e-01
Colwellia	7.776	-0.862	0.623	3.043e-01
Parasaccharibacter	82.051	0.329	0.251	3.078e-01
Salinarimonas	4.201	0.584	0.439	3.078e-01
Phycomyces	7.189	-2.085	1.508	3.100e-01
Calothrix	3.536	-1.385	1.088	3.100e-01
Malassezia	1277.950	-0.476	0.345	3.102e-01
Pelagibacterium	4.466	-1.674	1.281	3.102e-01
Rhizobiales	18.512	0.693	0.533	3.138e-01
Nocardioides	721.043	0.237	0.181	3.169e-01
Loktanella	12.504	-0.438	0.326	3.169e-01
Mammaliicoccus	9.464	1.044	0.817	3.179e-01
Mycobacteriaceae_genus	41.727	0.336	0.267	3.206e-01
Fulvia	3.759	-1.004	0.833	3.206e-01
Fimbriimonas	2.579	1.809	1.288	3.206e-01
Gemmatirosa	1.806	-2.133	1.869	3.206e-01
Mixta	11.107	-0.977	0.709	3.208e-01
Nitrobacter	5.995	0.942	0.762	3.208e-01
Pyrococcus	12.784	0.829	0.668	3.235e-01
Pelosinus	4.274	-1.247	1.025	3.241e-01
Metarhizium	16.962	-0.908	0.649	3.256e-01
Arcobacter	3.620	1.172	0.948	3.268e-01
Diolcogaster_facetosa_bracovirus	12.306	-0.940	0.679	3.279e-01
Meyerozyma	1.367	2.636	2.107	3.279e-01
Cokeromyces	2.224	-0.660	0.616	3.288e-01

Salifodinibacter	1.493	-2.063	1.958	3.288e-01
Huaxiibacter	3.774	-1.309	1.090	3.290e-01
Gardnerella	33.910	-0.983	0.722	3.312e-01
Saccharibacillus	2.894	-1.836	1.558	3.313e-01
Wolinella	1.486	-1.965	1.956	3.313e-01
Oceanicella	2.913	-1.226	1.044	3.313e-01
Streptacidiphilus	1.421	1.014	1.706	3.336e-01
Phycicoccus	66.900	0.375	0.300	3.370e-01
Sandaracinobacteroides	6.329	-1.874	0.909	3.370e-01
Paucibacter	116.341	0.337	0.272	3.402e-01
Pseudoglutamicibacter	1.363	1.921	1.494	3.402e-01
Cercospora	12.008	-0.834	0.647	3.438e-01
Anabaena	1.596	1.818	1.353	3.447e-01
Lasiodiplodia	27.457	0.503	0.412	3.447e-01
Segatella	9.943	-0.877	0.691	3.447e-01
Frankineae	1.968	-2.417	2.243	3.464e-01
Phaeovulum	30.793	0.448	0.367	3.496e-01
Sphaerotilus	3.359	-1.269	1.139	3.600e-01
Lelliottia	2.324	-0.775	0.763	3.637e-01
Thermosipho	4.351	-0.610	0.526	3.637e-01
Paludifilum	42.859	0.314	0.265	3.692e-01
Rhodocyclaceae_genus	4.038	-1.172	1.049	3.708e-01
UNVERIFIED_ORG:	13.458	0.659	0.563	3.727e-01
Nevskia	5.606	-1.463	1.256	3.741e-01
Pleurotus	2.527	-1.330	1.260	3.760e-01
Polymorphobacter	6.803	1.098	0.948	3.765e-01
Aeromonas	84.822	0.308	0.266	3.788e-01
Psychrobacter	113.554	-0.417	0.344	3.821e-01
Crocosphaera	13.832	0.530	0.468	3.821e-01
Alkanindiges	7.099	0.975	0.865	3.821e-01
Aplosporella	8.752	1.010	0.897	3.822e-01
Aeromicrobium	36.608	0.468	0.406	3.828e-01
Chitinophaga	7.848	-0.836	0.729	3.864e-01
Alternaria	98.393	0.309	0.268	3.882e-01
Pseudooceanicola	63.502	-0.637	0.528	3.883e-01
Thermomonas	34.874	0.350	0.308	3.889e-01
Pseudokineococcus	8.737	-1.685	1.423	3.891e-01
Filomicrobium	2.369	1.321	1.245	3.891e-01
Pseudoclavibacter	10.937	0.789	0.711	3.894e-01
Mesorhizobium	79.330	0.259	0.228	3.912e-01
Scedosporium	1.465	-1.843	1.986	3.914e-01
Penicillium	43.107	0.474	0.422	3.936e-01
Candidatus	2.396	1.739	1.594	3.936e-01
Haemophilus	217.469	-0.374	0.320	3.955e-01
Methylobrevis	19.272	0.537	0.482	3.964e-01
Aquimarina	1.439	-1.804	2.075	3.964e-01
Myxococcales	1.509	-2.074	2.228	3.964e-01
Desulfovibrio	71087.628	-0.305	0.261	3.970e-01
Spirosoma	11.388	-0.852	0.729	3.977e-01
Terrihabitans	1.434	-2.011	2.196	3.986e-01
Asinibacterium	2.381	-1.052	1.091	4.004e-01
Azohydromonas	15.191	0.616	0.562	4.033e-01
Treponema	11.715	-0.699	0.613	4.033e-01
Simplicispira	1.925	-0.966	1.125	4.081e-01
Acidobacteria	2.858	-1.057	1.071	4.097e-01
Parasphingorhabdus	1.435	-1.300	1.351	4.099e-01
Oceanicola	2.846	1.271	1.215	4.102e-01
Marinithermofilum	29.006	0.336	0.312	4.105e-01
Ralstonia	14254.930	0.207	0.188	4.109e-01

Paraclostridium	10.370	-0.930	0.825	4.112e-01
Methylosarcina	17.576	0.719	0.668	4.112e-01
Pyrenophora	5.376	1.060	1.015	4.126e-01
Phialophora	4.587	1.143	1.107	4.133e-01
Myxococcus	31.942	0.330	0.309	4.138e-01
Thermoanaerobacterium	14.931	-1.297	1.091	4.176e-01
Halopseudomonas	3.486	-1.233	1.050	4.195e-01
Janibacter	177.517	0.295	0.274	4.207e-01
Actinosynnema	2.269	1.045	0.988	4.207e-01
Skermanella	24.233	-0.776	0.683	4.270e-01
Planctomonas	4.608	1.090	1.035	4.284e-01
Tissierella	290.882	0.235	0.222	4.319e-01
Ezakiella	1.567	1.500	2.025	4.319e-01
Actinobacterium	1.502	1.873	1.842	4.395e-01
Thioflexithrix	10.033	0.534	0.529	4.397e-01
Beggiatoa	32.646	0.298	0.287	4.405e-01
Blastomyces	2.516	-0.984	1.104	4.427e-01
Tetrasphaera	10.317	0.771	0.775	4.495e-01
Endocarpon	5.929	0.783	0.771	4.516e-01
Yimella	7.431	0.834	0.847	4.522e-01
Psychrosphaera	4.364	0.779	0.734	4.526e-01
Rickettsiella	1.563	-0.784	0.942	4.526e-01
Taibaiella	8.236	-1.106	0.880	4.585e-01
Porphyrobacter	4.254	0.734	0.730	4.585e-01
Chaetomium	2.688	-1.156	1.232	4.620e-01
Dactylellina	1.676	-1.526	1.621	4.620e-01
Devosia	86.884	-0.453	0.433	4.637e-01
Radiomyces	1.508	1.551	1.453	4.654e-01
Anoxybacillus	29.382	0.732	0.749	4.658e-01
Rhizobium	451.252	0.187	0.189	4.706e-01
Fluoribacter	203.940	0.315	0.319	4.706e-01
Capnocytophaga	41.728	-0.366	0.359	4.706e-01
Diplodia	4.466	0.889	0.965	4.706e-01
Thauera	36.207	0.276	0.281	4.729e-01
Ectobacillus	119.344	0.403	0.414	4.753e-01
Geodermatophilus	37.591	0.619	0.646	4.753e-01
Synechocystis	16.400	-0.800	0.782	4.786e-01
Dechloromonas	6.203	0.749	0.796	4.786e-01
Ruania	2.191	1.226	1.279	4.825e-01
Pigmentiphaga	5.865	0.799	0.861	4.826e-01
Paraprevotella	4.071	-1.261	1.406	4.826e-01
Neorhizobium	3.954	-1.011	1.153	4.826e-01
Weizmannia	1.405	-1.979	2.560	4.844e-01
Variovorax	102.492	-0.287	0.290	4.861e-01
Limnohabitans	19.440	0.382	0.403	4.911e-01
Xanthobacter	6.049	-0.687	0.775	4.920e-01
Kluyvera	247.288	-0.212	0.221	4.977e-01
Pseudophaeobacter	4.670	0.614	0.896	4.977e-01
Conchiformibius	7.052	-0.844	0.935	4.985e-01
Mucilaginibacter	56.373	-0.337	0.354	5.012e-01
Oceanitalea	1.478	1.630	1.761	5.089e-01
Carnobacterium	19.889	-0.543	0.575	5.092e-01
Lancefieldella	4.241	-1.836	1.457	5.092e-01
Fusibacter	1.572	-1.467	2.003	5.117e-01
Miltoncostaea	4.350	1.223	1.556	5.155e-01
Angustibacter	3.139	1.121	1.339	5.155e-01
Glaciimonas	3.779	0.996	1.146	5.172e-01
Aphanothece	13.799	0.448	0.503	5.194e-01
Asticcacaulis	114.652	-0.443	0.472	5.212e-01

Lachnellula	3.803	-0.759	0.961	5.221e-01
Sclerotinia	1.417	-1.001	1.488	5.228e-01
Lacrimispora	1.596	-1.547	1.866	5.235e-01
Abiotrophia	14.388	-0.669	0.724	5.235e-01
Aquincola	21.141	0.556	0.632	5.261e-01
Parageobacillus	1.709	1.003	1.653	5.261e-01
Kushneria	83.057	-0.296	0.324	5.284e-01
Cellulomonas	41.380	0.346	0.397	5.302e-01
Tetragenococcus	7.371	0.837	0.983	5.309e-01
Hubei_permutotetra-like_virus	2.387	1.281	1.997	5.315e-01
Verrucomicrobia	1.670	-1.882	1.855	5.338e-01
Tatumella	7.235	-0.765	0.720	5.349e-01
Megasphaera	17.752	-0.742	0.790	5.357e-01
Baudoinia	14.011	-0.527	0.590	5.364e-01
Aliihoeflea	2.483	-2.174	1.637	5.367e-01
Labilithrix	4.216	-0.955	1.221	5.367e-01
Fredinandcohnia	6.991	0.600	0.710	5.367e-01
Propionicimonas	2.047	-0.889	1.261	5.380e-01
Gallaecimonas	1.455	-0.750	1.092	5.380e-01
Renibacterium	7.693	0.772	0.917	5.388e-01
Coprobacillus	1.834	-1.808	1.422	5.392e-01
Sphingosinicella	7.978	0.748	0.908	5.408e-01
Glaciecola	6.760	-0.383	0.445	5.416e-01
Austwickia	2.277	1.237	1.327	5.428e-01
Marinilactibacillus	3.912	-0.956	1.266	5.428e-01
Segetibacter	2.497	-0.885	1.445	5.429e-01
Type-D_symbiont_of_Plautia_stali	2.107	-2.520	3.395	5.438e-01
Puia	26.661	-0.307	0.355	5.438e-01
Parafrankia	1.838	-0.887	1.304	5.480e-01
Actinobaculum	5.253	0.766	0.921	5.508e-01
Hydrogenophilus	2.405	-1.589	1.748	5.508e-01
Arsenicicoccus	2.165	0.871	1.175	5.508e-01
Sandaracinus	1.493	1.563	1.844	5.536e-01
Patulibacter	9.106	-0.734	0.890	5.543e-01
Naumannella	5.114	-0.759	1.005	5.549e-01
Yaniella	1.655	1.291	1.439	5.553e-01
Marinilabiliaceae_genus	2.283	0.618	0.915	5.559e-01
Brenneria	3.075	0.472	0.494	5.559e-01
Exiguobacterium	51.512	0.327	0.399	5.581e-01
Ideonella	12.997	0.522	0.657	5.581e-01
Rugamonas	22.555	-0.406	0.479	5.625e-01
Methylorubrum	226.898	-0.165	0.200	5.636e-01
Thiobacillus	5.354	-0.675	0.912	5.636e-01
Mycosynbacter	1.754	-0.967	1.481	5.688e-01
Varibaculum	3.474	-0.791	1.148	5.688e-01
Baekduia	2.199	0.792	1.282	5.688e-01
Hyalangium	1.398	1.842	2.356	5.688e-01
Xanthomonas	75.563	-0.282	0.346	5.725e-01
Frateuria	2.007	-0.562	0.919	5.756e-01
Algibacter	5.117	-0.693	0.667	5.758e-01
Sphingorhabdus	2.358	0.562	1.401	5.758e-01
Flavihumibacter	2.570	-0.770	0.996	5.767e-01
Rudaea	1.559	-0.496	0.881	5.767e-01
Gluconobacter	9.302	-0.590	0.762	5.810e-01
Puccinia	4.914	-0.646	0.933	5.810e-01
Sphingomonas	2004.426	0.118	0.152	5.813e-01
Dickeya	1.888	-0.167	0.553	5.813e-01
Barnesiella	9.605	0.337	0.441	5.813e-01
Prolinoborus	2.487	-0.604	0.974	5.813e-01

Desertimonas	11.415	-0.866	1.091	5.850e-01
Gemmiger	1.474	1.364	1.604	5.853e-01
Chthoniobacter	2.198	0.628	1.703	5.905e-01
Mobilicoccus	15.933	0.495	0.669	5.932e-01
Kinneretia	11.622	-0.466	0.607	5.962e-01
Azoarcus	3.353	0.764	0.980	5.963e-01
Catonella	3.258	-0.692	1.098	5.963e-01
Nesterenkonia	69.289	0.239	0.321	6.013e-01
Citricoccus	29.626	0.427	0.587	6.027e-01
Mesonia	2.526	-0.797	0.840	6.056e-01
Kytococcus	23.988	0.421	0.576	6.056e-01
Arthrobacter	566.114	0.157	0.213	6.065e-01
Dichomitus	29.347	0.570	0.803	6.110e-01
Actinokineospora	43.083	-0.461	0.621	6.177e-01
Actinomarinicola	1.651	1.317	1.463	6.177e-01
Gryllotalpicola	4.898	-0.755	1.181	6.177e-01
Methylocella	3.329	1.093	1.477	6.177e-01
Lysobacter	182.469	-0.182	0.250	6.182e-01
Cardiobacterium	13.923	-0.504	0.671	6.190e-01
Rhodospirillaceae_genus	1.528	-1.116	1.543	6.223e-01
Moesziomyces	19.781	-0.794	1.064	6.223e-01
Besnoitia	7.844	0.340	0.514	6.235e-01
Chryseobacterium	293.101	-0.173	0.242	6.272e-01
Motilibacter	2.793	1.223	1.632	6.272e-01
Bartonella	9.625	-0.483	0.666	6.272e-01
Advenella	1.895	0.937	1.147	6.272e-01
Nitrolancea	1.489	-1.277	1.871	6.272e-01
Truepera	3.557	-0.745	1.267	6.288e-01
Polynucleobacter	2.817	-0.810	1.100	6.288e-01
Propionicicella	1.665	1.137	1.471	6.288e-01
Psychromonas	1.967	-0.344	0.789	6.288e-01
Hoeflea	3.595	0.527	1.112	6.314e-01
Weissella	5.780	-0.569	0.937	6.379e-01
Moraxellaceae_genus	14.760	-0.456	0.662	6.405e-01
Lacibacter	2.371	-1.521	2.636	6.420e-01
Barrientosiimonas	8.157	0.791	1.215	6.420e-01
Fervidibacillus	1.551	-1.369	2.375	6.420e-01
Mangrovicoccus	4.133	-0.718	1.050	6.420e-01
Pseudorivibacter	1.485	-0.900	1.620	6.420e-01
Didymosphaeria	28.357	-0.477	0.697	6.423e-01
Pseudocercospora	7.879	-0.489	0.763	6.423e-01
Iamia	4.767	-1.011	1.292	6.423e-01
Thermococcus	14.111	0.313	0.483	6.431e-01
Acetobacterium	3.427	-0.608	1.136	6.444e-01
Aliterella	5.985	0.769	1.198	6.455e-01
Geminicoccus	65.368	-0.178	0.275	6.463e-01
Cutibacterium	314.810	0.215	0.334	6.486e-01
Achromobacter	178.806	0.101	0.156	6.486e-01
Solihabitans	20.875	0.156	0.315	6.486e-01
Ectothiorhodospira	16.455	0.273	0.428	6.486e-01
Vallicoccus	2.925	0.869	1.473	6.486e-01
Micropruina	6.588	0.711	1.132	6.486e-01
Alteribacter	6.236	0.298	0.489	6.486e-01
Cereibacter	5.029	0.607	0.919	6.486e-01
Xylella	2.546	0.562	0.717	6.486e-01
Pasteurellaceae_genus	2.823	-0.558	1.073	6.486e-01
Methylophilus	8.689	0.397	0.636	6.490e-01
Enterovirga	5.894	-0.648	1.123	6.531e-01
Hankyongella	1.915	1.441	2.236	6.575e-01

Amnibacterium	7.258	-0.547	0.928	6.618e-01
Nonomuraea	1.439	-0.587	1.360	6.649e-01
Pseudozyma	9.591	0.505	0.840	6.686e-01
Kingella	8.778	-0.364	0.616	6.691e-01
Salinicola	3.874	-0.310	0.666	6.751e-01
Roseburia	27.841	0.185	0.309	6.794e-01
Knoellia	9.989	0.481	0.816	6.794e-01
Solimonas	5.862	0.575	1.020	6.801e-01
Roseicella	3.466	0.716	1.112	6.853e-01
Nakamurella	30.160	-0.377	0.621	6.877e-01
Prosthecobacter	3.876	0.764	1.172	6.891e-01
Tabrizicola	3.196	0.382	0.972	6.891e-01
Arachnia	16.113	-0.389	0.653	6.905e-01
Auritidibacter	1.372	-1.060	2.940	6.906e-01
Frigoribacterium	15.155	-0.450	0.744	6.930e-01
Methyloversatilis	103.042	-0.188	0.324	6.946e-01
Leuconostoc	27.068	0.218	0.379	6.946e-01
Tolypothrix	6.280	-0.497	0.964	6.946e-01
Terracoccus	15.641	0.516	0.914	6.946e-01
Flexivirga	2.311	0.579	1.292	6.946e-01
Blochmannia	13.304	0.462	0.813	6.950e-01
Fusicatenibacter	1.465	-1.086	1.645	6.954e-01
Bhargavaea	9.142	0.393	0.708	6.963e-01
Parasphingopyxis	15.233	0.417	0.744	6.989e-01
Paraconexibacter	2.432	-0.572	1.310	6.997e-01
Rhodopseudomonas	31.634	-0.241	0.417	7.003e-01
Kockovaella	3.506	0.722	1.188	7.004e-01
Scytonema	22.749	0.307	0.568	7.028e-01
Frondihabitans	2.164	-0.607	1.442	7.034e-01
Planktothrix	6.905	-0.437	0.747	7.034e-01
Vitreoscilla	1.803	-0.651	1.731	7.052e-01
Phaeoacremonium	3.214	-0.410	0.841	7.067e-01
Blastococcus	150.500	0.241	0.448	7.074e-01
Rhizorhabdus	68.045	0.188	0.346	7.074e-01
Planctomyces	2.081	-0.481	1.231	7.074e-01
Xylanimonas	3.351	-0.481	1.144	7.074e-01
Cohnella	3.253	-0.312	0.828	7.074e-01
Brochothrix	1.522	-0.898	1.580	7.074e-01
Ancylobacter	5.043	0.611	1.106	7.104e-01
Ferruginibacter	1.535	-0.478	1.475	7.136e-01
Tepidiphilus	60.638	-0.388	0.708	7.146e-01
Parerythrobacter	4.100	-2.112	1.536	7.146e-01
Chitinimonas	12.648	-0.448	0.809	7.154e-01
Pinisolibacter	11.572	-0.056	0.344	7.167e-01
Xenophilus	37.016	0.197	0.385	7.169e-01
Rhizorhapis	25.863	-0.307	0.582	7.169e-01
Quadrisphaera	13.743	0.522	1.018	7.185e-01
Actinomyces	520.914	-0.148	0.281	7.198e-01
Garicola	1.949	0.853	1.717	7.198e-01
Aurantiacibacter	6.751	-0.436	0.942	7.210e-01
Paenibacillus	158.355	-0.112	0.217	7.240e-01
Nocardiopsis	6.413	-0.522	1.142	7.240e-01
Hypoxylon	1.453	-0.886	1.675	7.240e-01
Alishewanella	16.028	-0.392	0.775	7.285e-01
Nitrotoga	4.783	0.353	0.682	7.285e-01
Debaryomyces	1.396	-0.764	2.090	7.285e-01
Eleftheria	9.525	0.364	0.738	7.298e-01
Allobacillus	31.886	-0.213	0.430	7.333e-01
Phocaeicola	15.829	-0.370	0.731	7.364e-01

Altererythrobacter	4.874	0.554	1.081	7.364e-01
Peribacillus	3.761	-0.404	1.080	7.364e-01
Sediminibacterium	2.811	-0.505	1.347	7.364e-01
Microvirgula	1.737	-0.647	1.879	7.383e-01
Lactiplantibacillus	3.865	-0.325	0.631	7.389e-01
Cryptococcus	9.052	-0.482	0.973	7.404e-01
Nostoc	288.872	-0.112	0.233	7.429e-01
Atopomonas	30.225	-0.154	0.335	7.433e-01
Pedomonas	2.739	0.450	1.222	7.465e-01
Ramlibacter	69.721	0.204	0.441	7.492e-01
Parabacteroides	28.004	0.182	0.404	7.516e-01
Flavimobilis	2.525	0.702	1.244	7.549e-01
Tepidimonas	35.654	0.258	0.571	7.556e-01
Lentzea	21.126	-0.149	0.361	7.582e-01
Alkalibacterium	1.571	1.061	1.761	7.582e-01
Klenkia	7.551	0.376	0.856	7.584e-01
Cellulosimicrobium	4.141	1.240	1.464	7.603e-01
Photorhabdus	47.052	0.185	0.433	7.623e-01
Pannonibacter	2.009	-0.505	1.592	7.623e-01
Shimia	358.445	0.154	0.351	7.637e-01
Neobacillus	44.891	0.121	0.287	7.637e-01
Entotheonella	3.765	-0.268	0.814	7.637e-01
Perlucidibaca	1.576	-0.286	1.132	7.637e-01
Oceaniovalibus	4.526	0.405	0.840	7.669e-01
Filifactor	2.916	0.617	1.163	7.669e-01
Demequina	2.317	0.502	1.242	7.669e-01
Fictibacillus	317.200	-0.176	0.405	7.675e-01
Roseisolibacter	3.912	-0.415	1.280	7.704e-01
Rhodanobacter	6.156	-0.396	0.752	7.717e-01
Methylopila	6.130	0.428	0.992	7.782e-01
Ehrlichia	3.296	-0.394	1.278	7.782e-01
Olsenella	7.411	0.380	0.940	7.811e-01
Curvibacter	191.180	0.070	0.175	7.815e-01
Pseudoduganella	3.809	0.345	0.798	7.856e-01
Solirubrobacter	24.375	-0.278	0.688	7.887e-01
Aquihabitans	4.924	-0.400	1.252	7.887e-01
Paracaedibacter	1.572	0.977	1.883	7.887e-01
Megamonas	3.586	0.269	1.194	7.914e-01
Izhakiella	14.322	0.380	0.991	7.951e-01
Polaromonas	23.598	0.135	0.358	8.001e-01
Acidiplasma	17.926	0.147	0.397	8.001e-01
Caldimonas	5.324	0.374	1.005	8.001e-01
Pseudopropionibacterium	6.216	-0.257	0.811	8.001e-01
Fretibacterium	3.170	-0.347	1.288	8.002e-01
Xylophilus	6.954	-0.183	0.611	8.006e-01
Sphaerobacter	6.365	0.501	1.379	8.031e-01
Campylobacter	38.374	-0.124	0.345	8.114e-01
Parvularcula	2.074	0.777	1.663	8.122e-01
Telluria	20.994	-0.210	0.602	8.157e-01
Ciceribacter	3.850	0.401	0.950	8.212e-01
Dysgonomonas	10.780	-0.186	0.533	8.234e-01
Kwoniella	6.912	0.364	1.098	8.234e-01
Cyclobacterium	9.163	-0.021	0.441	8.234e-01
Halalkalibacter	112.475	-0.119	0.357	8.300e-01
Actinomycetospora	24.819	-0.222	0.665	8.303e-01
Dermabacter	5.640	-0.278	1.044	8.303e-01
Verrucosispora	2.100	-0.206	1.140	8.303e-01
Sporolactobacillus	110.272	0.061	0.189	8.319e-01
Methylovorus	3.354	-0.210	0.996	8.319e-01

Solemya	6.351	0.260	0.794	8.332e-01
Jannaschia	2.279	0.264	0.503	8.346e-01
Diaporthe	5.499	0.433	1.281	8.364e-01
Fusarium	142.957	0.206	0.671	8.396e-01
Mycobacterium	323.882	0.068	0.220	8.396e-01
Limosilactobacillus	14.701	-0.147	0.517	8.396e-01
Thermohydrogenium	1.914	-0.720	2.130	8.396e-01
Minicystis	1.842	-0.440	2.026	8.396e-01
Leucobacter	21.363	0.140	0.469	8.418e-01
Heyndrickxia	3.581	0.274	1.632	8.426e-01
Ruegeria	9.018	0.236	0.813	8.426e-01
Chlorogloea	3.479	-0.303	1.538	8.453e-01
Schizosaccharomyces	3.316	0.425	1.331	8.489e-01
Pseudoxanthomonas	75.899	0.102	0.352	8.497e-01
Acanthamoeba	49.409	-0.235	0.798	8.501e-01
Hyphomicrobium	34.815	-0.101	0.349	8.507e-01
Desulforhabdus	2.687	0.357	1.266	8.535e-01
Inhella	1.432	-0.347	1.777	8.535e-01
Tessaracoccus	17.444	-0.209	0.757	8.606e-01
Sutterella	3.974	0.257	0.729	8.611e-01
Enhydrobacter	51.501	-0.090	0.335	8.617e-01
Piscicoccus	2.025	-0.317	1.735	8.617e-01
Thiohalocapsa	35.128	0.109	0.423	8.635e-01
Latilactobacillus	2.108	0.717	1.528	8.635e-01
Gulosibacter	3.895	0.370	1.273	8.635e-01
Aequorivita	205.552	-0.068	0.257	8.654e-01
Solibacillus	6.613	-0.148	0.772	8.665e-01
Ottowia	13.988	0.136	0.565	8.725e-01
Abditibacterium	5.942	-0.223	1.138	8.733e-01
Snodgrassella	4.153	-0.237	1.400	8.763e-01
Oscillatoria	1.378	-0.231	1.380	8.837e-01
Pyxidicoccus	1.671	0.618	1.330	8.893e-01
Pararhodobacter	5.193	0.214	0.930	8.893e-01
Falsiroseomonas	137.429	0.061	0.298	8.933e-01
Chondromyces	1.551	-1.531	1.944	8.933e-01
Pandoraea	33.582	0.080	0.374	8.965e-01
Coprococcus	3.166	0.513	1.584	9.022e-01
Galbitalea	2.897	-0.174	1.427	9.084e-01
Microvirga	80.646	0.065	0.361	9.087e-01
Stutzerimonas	32.547	-0.102	0.521	9.087e-01
Oleiphilus	1.386	0.328	0.862	9.087e-01
Labilibacter	4.141	-0.181	0.995	9.087e-01
Cronobacter	1.421	0.591	1.100	9.176e-01
Ancylomarina	7.570	-0.076	0.668	9.197e-01
Sporichthya	4.613	-0.329	1.224	9.307e-01
Candidate	14.431	-0.068	0.424	9.339e-01
Acetivibrio	1.633	-0.600	2.231	9.401e-01
Marasmius	3.117	0.042	1.166	9.401e-01
Ligilactobacillus	12.484	0.087	0.738	9.434e-01
Blautia	17.234	-0.077	0.648	9.516e-01
Longimicrobium	4.867	0.235	1.180	9.516e-01
Lacticaseibacillus	6.204	-0.073	0.791	9.516e-01
Desulfuromonas	2.026	-0.129	1.690	9.526e-01
Actinomadura	901.640	-0.022	0.176	9.549e-01
Bowmanella	57.320	-0.045	0.386	9.549e-01
Macrococcus	4.781	-0.038	0.898	9.549e-01
Peptostreptococcaceae_genus	4.076	-0.062	1.038	9.549e-01
Gordonia	74.952	0.049	0.422	9.606e-01
Pseudoalteromonas	365.269	-0.027	0.240	9.645e-01

Bifidobacteriaceae_genus	8.416	0.119	1.073	9.671e-01
Collimonas	7.742	0.042	0.811	9.673e-01
Noviherbaspirillum	23.982	-0.085	0.805	9.673e-01
Cupriavidus	310.513	0.023	0.237	9.679e-01
Grimontella	3.770	-0.021	0.868	9.686e-01
Agathobacter	7.239	-0.090	1.191	9.688e-01
Streptomyces	1952.590	-0.016	0.173	9.721e-01
Leucothrix	10.440	-0.017	0.565	9.721e-01
Azorhizobium	2.897	0.308	1.079	9.738e-01
Zimmermannella	7.604	-0.061	0.909	9.772e-01
Microcella	4.645	-0.030	1.005	9.777e-01
Actinotalea	15.791	-0.064	0.824	9.821e-01
Bifidobacterium	47.754	0.026	0.402	9.930e-01
Thyridium	3.802	0.251	1.227	9.936e-01
Emericellopsis	3.169	0.306	1.746	1.000e+00
Dacryopinax	1.918	0.336	1.596	1.000e+00
Humisphaera	4.982	-0.533	1.530	1.000e+00
Pontibacillus	7.454	-0.017	1.493	1.000e+00
Haliangium	2.455	-1.543	1.753	1.000e+00
Gemmobacter	13.161	0.036	0.750	1.000e+00
Agromyces	13.851	0.009	0.496	1.000e+00
Eubacterium	18.755	-0.013	0.475	1.000e+00
Kaistella	17.043	-0.012	0.567	1.000e+00
Calidithermus	19.574	0.005	0.635	1.000e+00
Ilumatobacter	4.926	-0.527	1.264	1.000e+00
Pleomorpha	4.781	-0.039	1.086	1.000e+00
Oxalobacteraceae_genus	2.465	-0.138	1.041	1.000e+00
Herbiconiux	5.773	-0.757	1.163	1.000e+00
Ilyonectria	3.065	-1.620	1.285	1.000e+00
Pedococcus	6.046	0.045	0.833	1.000e+00
Fortiea	2.157	0.338	1.534	1.000e+00
Azonexus	3.481	0.215	1.260	1.000e+00
Hephaestia	1.644	0.025	1.481	1.000e+00
Pseudorhizobium	3.953	-1.543	1.299	1.000e+00
Croceibacterium	1.652	0.464	1.447	1.000e+00
Falsirhodobacter	2.150	0.268	1.369	1.000e+00
Archangium	2.807	0.176	0.942	1.000e+00
Aliarcobacter	1.698	0.081	2.388	1.000e+00
Fimbriiglobus	2.293	0.098	1.465	1.000e+00
Protaetiibacter	1.770	0.044	1.379	1.000e+00
Cryptosporangium	2.818	-0.004	1.525	1.000e+00
Tsuneonella	2.526	0.301	1.603	1.000e+00
Aestuariimicrobium	3.255	-1.040	1.179	1.000e+00
Cyberlindnera	2.022	-0.036	1.449	1.000e+00
Paenacidovorax	3.180	0.057	1.143	1.000e+00
Lactovum	3.125	0.216	1.252	1.000e+00
Saccharomonospora	2.103	0.193	1.139	1.000e+00
Kaistia	3.343	0.284	1.141	1.000e+00
Mobiluncus	3.137	0.230	1.221	1.000e+00
Alsobacter	1.691	0.050	1.436	1.000e+00
Saccharomyces	1.469	0.342	1.872	1.000e+00
Aminobacter	1.557	-0.030	1.103	1.000e+00
Methylococcus	3.490	0.133	0.851	1.000e+00
Komagataeibacter	1.616	-1.232	1.687	1.000e+00
Lacipirellula	1.709	-1.144	2.084	1.000e+00
Hansschlegelia	1.831	0.304	1.644	1.000e+00
Hydrocarboniphaga	1.904	0.032	1.395	1.000e+00
Mameliella	2.131	0.009	1.323	1.000e+00
Bergeriella	1.836	-0.219	1.856	1.000e+00

Pilimelia	1.515	0.491	1.633	1.000e+00
Gamma	2.482	-0.010	1.278	1.000e+00
Saezia	1.565	0.014	0.986	1.000e+00
Pyruvatibacter	1.367	0.246	0.902	1.000e+00
Niveispirillum	1.564	-0.083	1.715	1.000e+00
Nannizzia	1.520	0.430	1.487	1.000e+00
Arsenophonus	1.327	-1.565	0.762	NA
Type-C_symbiont_of_Plautia_stali	0.212	0.435	3.199	NA
Sodalis-like	0.331	-0.120	1.180	NA
Bacteria	0.118	0.918	1.070	NA
Type-F_symbiont_of_Plautia_stali	0.400	-0.241	3.399	NA
Endozoicomonas	0.368	1.933	1.430	NA
Profftia	0.070	1.450	3.401	NA
Plautia	0.323	-0.202	1.909	NA
Type-E_symbiont_of_Plautia_stali	0.036	2.002	3.401	NA
Annandia	0.084	1.264	2.380	NA
Trabulsiella	0.462	-0.461	0.724	NA
Cnuella	0.966	0.247	2.041	NA
Paracnuella	0.644	-0.475	1.462	NA
Pseudactinotalea	0.706	-1.086	3.397	NA
Mangrovibacter	0.437	-0.108	0.798	NA
Fenollaria	0.794	-1.209	1.796	NA
Thiothrix	1.073	-0.034	2.565	NA
Mikella	0.000	0.000	0.000	NA
Promicromonospora	1.177	0.543	1.964	NA
Flaviaesturariibacter	0.912	-0.287	2.497	NA
Ogataea	1.303	-1.485	1.588	NA
Caulobacteraceae_genus	1.255	-0.767	2.223	NA
Ishikawaella	0.000	0.000	0.000	NA
Hafniaceae_genus	0.000	0.000	0.000	NA
Pluralibacter	1.045	0.884	1.574	NA
Bathymodiolus	0.021	1.596	3.402	NA
Hoaglandella	0.398	0.192	2.505	NA
Gilliamella	0.563	1.552	1.340	NA
Enteractinococcus	1.301	0.371	2.590	NA
Pediococcus	0.807	-0.427	1.233	NA
Superficieibacter	0.163	0.513	1.316	NA
Intestinirhabdus	0.996	-1.531	0.548	NA
Alicyclobacillus	0.659	-0.612	2.315	NA
Shigella_phage_SfIV_virus	0.000	0.000	0.000	NA
Jeotgalibacillus	1.208	-1.591	1.229	NA
Moranella	0.333	0.856	2.985	NA
Glaciihabitans	1.333	-1.736	1.646	NA
Zygosaccharomyces	0.127	1.690	3.401	NA
Kuraishia	0.224	0.222	3.400	NA
Chthonobacter	0.402	-0.293	3.399	NA
Mediannikoviicoccus	0.103	0.811	3.401	NA
Isosphaera	0.503	-0.673	3.398	NA
Tachikawaea	0.009	1.697	3.402	NA
Vagococcus	0.603	-0.892	2.764	NA
Aggregicoccus	0.395	1.970	3.394	NA
Pontibacter	1.186	2.637	1.667	NA
Bdellovibrio	0.992	-1.529	1.469	NA
Niabella	0.539	-0.469	2.102	NA
Pseudogemmobacter	0.944	-1.464	2.531	NA
Oscillochloris	0.821	0.559	3.392	NA
Faecalibacillus	0.308	-0.048	3.399	NA
Escherichia_phage_500465-1_virus	0.000	0.000	0.000	NA
Sporothrix	1.005	-1.536	1.814	NA

Euzebya	0.588	1.092	1.470	NA
Tolumonas	0.515	-0.236	2.343	NA
Niallia	0.268	0.287	2.272	NA
Umbelopsis	0.027	1.558	3.402	NA
Buttiauxella	1.130	1.145	1.148	NA
Pengzhenrongella	1.275	-1.317	1.733	NA
Enterobacteria_phage_DE3_virus	0.000	0.000	0.000	NA
Roseobacter	0.876	1.844	1.495	NA
Chryseomicrobium	0.671	-0.450	1.985	NA
Viridilinea	0.240	2.667	3.396	NA
Terribacillus	0.063	1.550	3.402	NA
Arthroderma	0.586	0.139	1.382	NA
Tenebrionibacter	0.381	-0.279	1.156	NA
Anatilimnocola	0.615	2.504	3.357	NA
Zafaria	0.027	1.933	3.402	NA
Winslowiella	0.287	0.849	3.401	NA
Oryzihumus	0.119	0.794	3.401	NA
Cobetia	0.578	-0.857	1.573	NA
Thermobacillus	0.214	0.443	3.400	NA
Negativicoccus	1.337	-1.489	3.396	NA
Chloroploca	0.474	-0.607	3.398	NA
Chitinophagaceae_genus	0.534	-0.483	2.723	NA
Berkiella	0.057	1.147	3.401	NA
Rubrivirga	1.178	0.336	2.381	NA
Zasmidium	1.244	-0.541	1.023	NA
Yoonia	0.398	0.097	3.114	NA
Fervidobacterium	0.190	0.330	3.400	NA
Enterobacteria_phage_P7_virus	0.000	0.000	0.000	NA
Pirellula	0.574	-0.622	2.287	NA
Halococcus	1.219	-1.388	1.055	NA
Thecamonas	1.245	-0.308	1.609	NA
Moorella	0.340	-0.242	3.399	NA
Acidiferrimicrobium	0.990	-1.163	1.824	NA
Agreia	0.629	-0.944	2.320	NA
Flectobacillus	1.269	-0.369	1.517	NA
Catenulispora	1.103	-1.554	2.734	NA
Acidothermus	1.064	-1.372	1.914	NA
Escherichia_phage_RCS47_virus	0.000	0.000	0.000	NA
Synechococcus	1.106	-0.412	1.598	NA
Pisolithus	1.250	-0.325	1.113	NA
Firmicutes	1.281	-1.002	1.436	NA
Armatimonas	0.426	-0.243	2.750	NA
Ilyomonas	0.580	-0.703	3.398	NA
Nosocomiicoccus	1.257	1.558	1.927	NA
Roseococcus	1.344	-1.879	1.591	NA
Evansella	0.869	-0.058	2.474	NA
Pseudonocardiaceae_genus	1.243	-0.880	1.971	NA
Tricharina	1.265	-0.370	1.435	NA
Thermodesulfomicrobium	0.202	0.274	3.400	NA
Planifilum	0.365	1.071	3.401	NA
SsRNA_phage_SRR5466369_2_virus	0.058	1.550	3.402	NA
Catenibacterium	1.044	-0.450	2.156	NA
Aciditerrimonas	0.899	-1.409	1.923	NA
Collibacillus	0.630	-0.488	3.036	NA
Carbonactinospora	0.476	-0.233	2.341	NA
Pseudofrankia	0.563	-0.727	2.525	NA
Hoyosella	1.072	-1.621	2.533	NA
Marisediminicola	1.178	-1.742	2.351	NA
Mongoliimonas	0.486	-0.638	3.398	NA

Lonsdalea	0.189	0.506	1.059	NA
Parasegetibacter	0.335	3.149	3.393	NA
Cryphonectria	1.076	-0.429	1.555	NA
Alterileibacterium	0.250	1.879	3.398	NA
Fonticella	0.702	-1.015	2.769	NA
Lagierella	0.000	0.000	0.000	NA
Sorangium	0.574	-0.831	2.784	NA
Gemmata	1.307	-1.859	1.742	NA
Caenimonas	0.820	-1.234	1.509	NA
Herpetosiphon	1.350	2.706	1.817	NA
Flaviflexus	0.920	0.146	1.924	NA
Stappia	1.350	-1.351	1.527	NA
Uruburuella	0.311	-0.142	3.399	NA
Acidiluteibacter	0.466	-0.366	3.398	NA
Neofamilia	0.005	1.709	3.402	NA
Laetiporus	0.825	-1.242	3.372	NA
Terriglobus	1.251	-1.702	1.407	NA
Flavipsychrobacter	0.537	-0.747	2.774	NA
Aliicoccus	0.581	-0.009	2.488	NA
Epithele	0.901	-1.380	1.402	NA
Herminiimonas	1.197	0.455	1.550	NA
Franconibacter	0.683	0.022	1.462	NA
Aquitalea	1.173	-1.226	0.889	NA
Xinfangfangia	1.289	0.753	1.661	NA
Riesia	0.026	1.564	3.402	NA
Hirsutella	0.438	-0.374	3.398	NA
Companilactobacillus	0.377	1.700	3.040	NA
Pseudosporangium	0.338	3.046	3.393	NA
Cereal_yellow_dwarf_virus	0.223	0.372	3.400	NA
Tenebrionicola	0.031	1.385	3.401	NA
Anaerotruncus	0.786	-0.089	1.039	NA
Acetanaerobacterium	0.863	1.274	1.888	NA
Pseudomassariella	0.846	-1.323	2.338	NA
Oscillibacter	0.819	0.143	2.793	NA
Alloiococcus	1.164	0.495	1.514	NA
Rhizobiaceae_genus	0.018	1.596	3.402	NA
Rhabdobacter	0.000	0.000	0.000	NA
Kallipyga	0.000	0.000	0.000	NA
Phascolarctobacterium	0.264	0.019	3.399	NA
Wielerella	1.083	-0.297	2.107	NA
Adlercreutzia	0.443	-0.530	2.344	NA
Cystobacter	1.340	0.422	1.828	NA
Actibacterium	0.985	-1.527	1.530	NA
Oxalicibacterium	1.142	0.965	1.647	NA
Pajaroellobacter	0.393	-0.385	3.131	NA
Escherichia_phage_500465-2_virus	0.000	0.000	0.000	NA
Microthrix	0.483	0.813	3.396	NA
Luteococcus	1.362	-1.876	2.053	NA
Escherichia_virus_Lambda_2G7b	0.000	0.000	0.000	NA
Type-B_symbiont_of_Plautia_stali	0.070	1.143	2.719	NA
Doolittlea	0.005	1.743	3.402	NA
Zymomonas	0.330	0.154	3.399	NA
Photodesmus	0.007	1.743	3.402	NA
Parachlamydia	0.456	-0.519	3.398	NA
Starkeya	0.955	-0.695	1.407	NA
Dubosiella	0.165	0.450	3.362	NA
Amniculibacterium	1.183	1.968	2.990	NA
Jiangella	1.073	0.386	1.846	NA
Dactylosporangium	0.676	-1.035	3.397	NA

Alistipes	0.914	-0.216	1.834	NA
Thermacetogenium	0.258	0.046	3.399	NA
Nitratireductor	1.356	-1.650	1.158	NA
Pseudohongiella	0.062	1.171	3.401	NA
Richelia	0.740	-1.060	1.920	NA
Gallintestinimicrobium	0.837	-1.302	3.025	NA
Drechmeria	0.521	-0.369	1.851	NA
Goekera	0.329	1.788	3.355	NA
Agaricicola	0.028	1.888	3.402	NA
Methanothermobacter	0.156	0.497	3.400	NA
Pedosphaera	0.186	0.509	3.400	NA
Erysipelothrix	0.043	1.273	3.401	NA
Desulfogranum	0.927	-1.072	1.782	NA
Neoarthrinium	0.839	0.257	1.717	NA
Mycetocola	0.892	-1.120	1.451	NA
Flintibacter	0.137	0.677	3.400	NA
Gemmataceae_genus	0.813	0.026	2.191	NA
Peptococcus	0.533	-0.737	3.398	NA
Zavarzinella	0.480	-0.620	3.103	NA
Saccharophagus	0.425	1.137	2.156	NA
Pinibacter	0.395	-0.290	2.800	NA
Escherichia_phage_TL-2011b_virus	0.000	0.000	0.000	NA
Enterobacteria_phage_f1_virus	0.008	1.743	3.402	NA
Pseudobdellovibrio	0.385	-0.384	2.338	NA
Sandaracinobacter	0.418	0.098	3.399	NA
Roseitranquillus	0.932	1.087	2.330	NA
Halomicroarcula	0.764	-1.059	1.192	NA
Calidifontibacter	0.577	-0.602	2.518	NA
Hassallia	0.246	0.486	3.400	NA
Siccibacter	0.129	0.674	2.706	NA
Dissoconium	0.190	1.046	3.401	NA
Quisquiliibacterium	0.886	0.556	2.211	NA
Defluviicoccus	0.374	0.095	2.724	NA
Arthromitus	0.148	1.276	3.401	NA
Thermoanaerobacter	0.645	0.639	3.057	NA
Mycoavidus	1.273	1.626	1.933	NA
Thiomonas	1.348	-0.157	1.473	NA
Millisia	1.115	2.941	2.238	NA
Qingrenia	0.304	-0.120	3.399	NA
Faecalicatena	0.820	-0.906	1.771	NA
Flavobacteriaceae_genus	1.172	-1.741	1.601	NA
Sphingosinithalassobacter	0.506	-0.438	1.834	NA
Nannocystis	1.346	-1.736	2.053	NA
Rhodovulum	0.928	-1.436	1.535	NA
Escherichia_virus_Lambda_4A7	0.000	0.000	0.000	NA
Macromonas	0.175	0.401	3.400	NA
Coprinopsis	1.211	-1.793	1.743	NA
Micrococcaceae_genus	0.443	-0.521	2.134	NA
Verticillium	0.616	-0.902	1.882	NA
harvey_murine_sarcoma_virus	0.947	2.154	0.455	NA
Rufibacter	1.318	-1.157	1.826	NA
Halobacillus	0.907	-1.352	1.368	NA
Sneathia	0.975	-1.185	2.714	NA
Cryobacterium	1.151	-0.710	1.972	NA
Sinisalibacter	0.394	0.794	2.272	NA
Centipeda	0.032	1.568	3.402	NA
Paraflavisolibacter	0.061	1.709	3.402	NA
Prauserella	0.485	-0.629	3.398	NA
Thermincola	0.633	-0.089	2.755	NA

Purpureocillium	0.658	-0.995	3.059	NA
Alkalicoccobacillus	1.046	-1.375	0.936	NA
Roseicitreum	1.223	-0.044	1.432	NA
Robbsia	1.088	-0.104	1.511	NA
Neurospora	1.235	-0.852	2.387	NA
Arenimonas	1.067	0.855	1.638	NA
Anaerotardibacter	0.125	0.843	2.623	NA
Ustilago	1.359	-1.921	1.500	NA
Schneideria	0.970	1.194	1.801	NA
Thermogemmata	1.288	-0.331	2.249	NA
Streptoalloteichus	1.111	-1.338	1.519	NA
Azovibrio	0.915	-1.409	1.724	NA
Minwuia	0.824	-0.263	3.094	NA
Ignavibacterium	0.501	1.542	3.393	NA
Scandinavium	0.642	-0.730	0.734	NA
Veillonellaceae_genus	0.948	2.082	2.299	NA
Intrasporangium	1.015	-1.483	2.042	NA
Rhodospirillum	1.216	0.775	1.566	NA
Desulfotomaculum	0.503	-0.160	2.738	NA
Desertibacillus	1.301	-1.833	1.728	NA
Planctomycetes	0.709	-1.103	2.016	NA
Propioniferax	0.403	0.332	3.400	NA
Beijerinckia	0.593	-0.676	2.333	NA
Myroides	1.084	-1.634	2.173	NA
Mariluticola	0.153	0.782	3.401	NA
Pusillibacter	0.000	0.000	0.000	NA
Stigmatella	0.992	-1.355	2.729	NA
Rectinema	0.081	0.958	3.401	NA
Ewingella	0.845	0.213	2.761	NA
Anaerobutyricum	0.119	1.657	3.400	NA
Klugiella	0.760	-1.182	3.051	NA
Arcticibacter	0.736	-0.342	1.658	NA
Propylenella	0.014	1.646	3.402	NA
Proteiniclasticum	1.099	-0.199	1.007	NA
Phototrophicus	0.608	-0.896	2.539	NA
Geoalkalibacter	0.121	1.600	3.402	NA
Tomitella	1.159	-0.741	1.605	NA
Pochonia	0.554	-0.750	1.754	NA
Plastoroseomonas	0.822	-1.288	2.150	NA
Lawsonibacter	0.311	0.089	2.562	NA
Globicatella	1.277	-1.843	2.057	NA
Plectonema	0.287	0.779	3.401	NA
Branchiibius	0.731	1.327	2.713	NA
Parasutterella	0.614	-0.731	1.513	NA
Plantactinospora	0.899	-1.189	1.476	NA
Truncatella	1.191	-1.506	1.656	NA
Aaosphaeria	0.407	1.796	2.674	NA
Bryobacter	0.866	1.057	3.045	NA
Puniceibacterium	0.825	-1.085	1.882	NA
Immundisolibacter	1.280	0.276	1.454	NA
Rhodoligotrophos	1.096	0.857	1.928	NA
Escherichia_phage_Lambda_ev099_virus	0.000	0.000	0.000	NA
Bacteroidetes	1.190	-1.580	2.168	NA
Crenalkalicoccus	0.840	-0.728	2.176	NA
Andreesenia	0.720	-0.710	2.987	NA
Klebsiella_phage_4_virus	0.000	0.000	0.000	NA
Catellicoccus	0.837	-1.306	2.349	NA
Rhodoblastus	1.242	1.093	1.838	NA
Wenxinia	0.425	-0.283	2.150	NA

Planobispora	0.083	1.001	3.401	NA
Mycoplana	0.174	0.650	3.400	NA
Peptoclostridium	0.886	1.152	1.995	NA
Aestuariibaculum	1.315	-0.970	1.337	NA
Kordiimonas	0.472	-0.248	2.466	NA
Maridesulfovibrio	0.179	0.380	3.400	NA
Neptuniibacter	0.092	0.884	3.401	NA
Stx2-converting_phage_1717_virus	0.000	0.000	0.000	NA
Planomonospora	0.421	0.244	2.495	NA
Fischerella	0.513	-0.695	3.081	NA
Theileria	0.680	-0.276	1.389	NA
Anaeromassilibacillus	0.075	2.183	3.400	NA
Oerskovia	0.479	0.360	2.944	NA
Phormidium	0.945	0.298	1.775	NA
Helcococcus	0.826	-0.169	2.560	NA
Glycomyces	0.831	-1.198	2.152	NA
Cetobacterium	0.000	0.000	0.000	NA
Acidiphilium	1.247	-1.537	1.652	NA
Steroidobacter	1.133	-0.859	1.559	NA
Parachlamydiaceae_genus	0.346	-0.118	3.399	NA
Actirhodobacter	0.376	-0.109	3.133	NA
Methylocapsa	1.039	-1.571	1.360	NA
Escherichia_phage_Cartapus_virus	0.000	0.000	0.000	NA
Rhodocista	0.302	0.502	1.947	NA
Coprothermobacter	0.362	-0.203	3.399	NA
Oligoflexus	0.666	-1.008	3.397	NA
Allosphingosinicella	0.640	0.092	1.838	NA
Schumannella	0.649	0.285	2.264	NA
Coriobacteriaceae_genus	0.743	-1.151	2.540	NA
Nanoperiomorbus	1.137	-1.706	1.641	NA
Pseudolabrys	0.513	-0.584	1.844	NA
Baumannia	0.277	1.414	3.401	NA
Phytohabitans	0.667	-1.018	3.062	NA
Cecembia	0.302	1.038	2.586	NA
Catellatospora	0.854	-0.814	2.947	NA
Xylaria	0.604	0.477	2.003	NA
Stakelama	0.615	2.125	1.758	NA
Polymorphum	0.621	2.126	1.974	NA
Reticulibacter	0.566	-0.814	3.398	NA
Nitrosocosmicus	0.792	-1.188	2.164	NA
Planctopirus	0.483	-0.637	1.669	NA
Ureibacillus	0.798	-0.388	1.691	NA
Fastidiosipila	0.389	0.612	3.400	NA
Carideicomes	0.567	0.770	1.316	NA
Aliidongia	0.759	0.898	1.857	NA
Caldovatus	0.368	-0.190	2.118	NA
Butyricicoccus	0.662	-0.658	1.888	NA
Bacteroidales	0.732	-1.145	1.878	NA
Thermorudis	0.331	0.991	3.206	NA
Rickettsia	0.876	-1.260	1.535	NA
Granulicoccus	0.433	1.444	2.768	NA
Granulicella	1.176	-1.680	2.184	NA
Dinghuibacter	0.335	0.255	3.400	NA
SsRNA_phage_SRR5466337_3_virus	0.000	0.000	0.000	NA
Gregarina	1.006	-0.838	2.688	NA
Zeimonas	0.839	-1.174	2.038	NA
Dokdonella	1.249	1.677	1.944	NA
Kickxella	0.434	-0.347	2.299	NA
Lamprobacter	0.388	-0.178	1.555	NA

Escherichia_phage_Lambda_ev207_virus	0.000	0.000	0.000	NA
Paracandidimonas	0.784	1.714	2.498	NA
Aromatoleum	0.470	0.351	2.564	NA
Escherichia_virus_Lambda_1H12	0.000	0.000	0.000	NA
Solirhodobacter	0.058	1.139	3.401	NA
Lampropedia	1.172	1.317	1.789	NA
Tropicibacter	0.000	0.000	0.000	NA
Rivularia	0.796	-1.183	2.165	NA
Nanosynsacchari	0.896	-1.353	1.684	NA
Nitrososphaera	1.144	-1.702	2.535	NA
Actinoallomurus	1.044	2.053	1.819	NA
Proteiniphilum	0.270	-0.005	3.399	NA
Ramularia	0.158	1.544	3.402	NA
Glaesserella	0.891	-1.416	1.046	NA
Rehaibacterium	0.368	-0.214	2.300	NA
Halteromyces	1.008	0.546	1.838	NA
Ustilaginoidea	1.049	3.471	1.858	NA
Nocardioidaceae_genus	0.397	-0.408	3.398	NA
Cordyceps	0.869	2.260	1.953	NA
Micavibrio	0.862	-1.326	2.019	NA
Variibacter	0.250	0.168	3.399	NA
Enterobacteria_phage_T7_virus	0.002	1.754	3.402	NA
Methyloglobulus	0.746	-1.008	1.585	NA
Emiliania	0.907	-0.637	1.380	NA
Oligella	1.269	-1.482	2.295	NA
Dermatobacter	0.611	1.697	1.983	NA
Pseudocnuella	0.005	1.714	3.402	NA
Lachnoclostridium	1.274	-1.842	2.350	NA
Anaerobacillus	0.305	-0.127	3.399	NA
Formosimonas	0.082	2.093	3.401	NA
Dongshaea	0.131	0.639	3.400	NA
Limobrevibacterium	0.486	-0.640	1.995	NA
Desulfofundulus	0.440	2.206	3.372	NA
Thermosinus	0.439	-0.518	3.398	NA
Idiomarina	0.506	-0.583	3.064	NA
Youxingia	0.215	0.935	2.896	NA
Stx2-converting_phage_Stx2a_WGPS2_virus	0.000	0.000	0.000	NA
Flavonifractor	0.231	0.453	3.400	NA
Paeniroseomonas	0.513	-0.700	1.995	NA
Phaeobacter	1.043	-1.130	0.992	NA
Paraphaeosphaeria	0.868	-0.227	2.748	NA
Thioalkalivibrio	1.217	-1.293	1.453	NA
Subtercola	1.074	-0.653	2.051	NA
Ruoffia	0.625	-0.933	2.162	NA
Paraflavitalea	0.310	0.203	3.400	NA
Crenobacter	1.097	-1.081	1.057	NA
Methylophaga	0.781	0.352	1.400	NA
Eremomyces	0.488	-0.642	2.533	NA
Paracraurococcus	0.696	-0.642	2.043	NA
Hyphomonas	1.185	1.734	1.928	NA
Faecalimonas	1.189	-1.676	1.819	NA
Actinoalloteichus	0.568	-0.010	1.624	NA
Durotheca	0.146	0.555	3.400	NA
Rhodospirillales	0.991	1.663	1.918	NA
Holdemania	0.072	1.030	3.401	NA
Butyrivibrio	0.998	-1.357	1.932	NA
Moheibacter	0.573	-0.638	2.733	NA
Sabulicella	0.126	0.730	3.401	NA
Agathobaculum	0.083	0.946	3.401	NA

Escherichia_phage_Lambda_ev243_virus	0.000	0.000	0.000	NA
Petrimonas	0.193	1.448	3.399	NA
Pseudorhodoplanes	0.962	-1.278	1.437	NA
Geomicrobium	0.127	1.567	3.399	NA
Coriobacteriales	0.346	-0.048	3.076	NA
Mumia	0.814	-1.086	2.198	NA
Laspinema	0.879	0.182	2.172	NA
Natronorubrum	0.689	-0.665	1.716	NA
Aeribacillus	0.369	0.929	3.401	NA
Aquimonas	0.678	-1.040	2.316	NA
Isoalcanivorax	1.062	-1.441	2.024	NA
Neoactinobaculum	0.622	-0.825	2.507	NA
Eimeria	1.027	-0.565	1.208	NA
Aquibium	0.920	-0.311	1.978	NA
Dankookia	0.758	-0.992	1.657	NA
Falsochrobactrum	0.395	3.038	3.295	NA
Acidisoma	1.255	-0.246	1.736	NA
Couchioplanes	0.000	0.000	0.000	NA
Yeguia	0.064	1.090	3.401	NA
Variimorphobacter	0.190	0.338	3.400	NA
Citreicoccus	0.220	0.195	3.400	NA
Serpentinimonas	0.382	-0.360	3.138	NA
Ferribacterium	0.267	0.271	3.400	NA
Xanthomarina	0.968	-0.025	1.721	NA
Miniimonas	0.208	0.255	3.234	NA
Pelagerythrobacter	0.197	1.312	3.398	NA
Allocoleopsis	0.836	-1.309	2.011	NA
Parvibaculum	1.055	-0.160	1.561	NA
Microterricola	0.560	-0.681	2.516	NA
Algibacillus	1.251	2.564	0.973	NA
Anaerosphaera	0.551	1.997	2.479	NA
Saliphagus	1.228	-1.740	1.337	NA
Labedella	0.708	-1.080	2.329	NA
Erysipelotrichaceae_genus	0.333	-0.212	3.399	NA
Frischella	1.199	-1.059	1.061	NA
Pichia	0.685	-0.672	1.567	NA
Morganella	1.022	-0.654	0.725	NA
Chlamydia	0.283	-0.050	3.213	NA
Aridibaculum	0.190	1.201	3.401	NA
Allostreptomyces	0.611	-0.913	2.773	NA
Lentihominibacter	0.458	0.861	2.312	NA
Wigglesworthia	1.349	-0.552	1.001	NA
Escherichia_virus_Lambda_2H10	0.000	0.000	0.000	NA
Chiayiivirga	0.821	-0.148	2.585	NA
Vibrionimonas	0.863	1.451	2.142	NA
Krasilnikoviella	0.109	0.768	3.401	NA
Lederbergia	0.311	-0.023	2.808	NA
Gulbenkiania	0.803	0.378	2.056	NA
Methanothrix	0.009	1.695	3.402	NA
Catenuloplanes	0.057	1.276	3.401	NA
Scardovia	0.474	-0.570	3.398	NA
Acidocella	1.112	-1.516	1.651	NA
Chloroflexi	0.627	1.700	1.641	NA
Linderina	1.155	0.805	1.251	NA
Rouxiella	0.687	-1.063	2.026	NA
Faunimonas	0.206	0.412	3.237	NA
Zobellella	1.254	-0.399	1.924	NA
Protochlamydia	0.307	-0.134	3.399	NA
Tuwongella	0.369	0.063	3.399	NA

Hydromonas	0.010	1.743	3.402	NA
Soleaferrea	0.148	0.540	3.400	NA
Liquorilactobacillus	0.992	1.266	1.905	NA
Thermobrachium	0.000	0.000	0.000	NA
Fuscibacter	0.854	2.334	2.361	NA
Amygdalobacter	0.372	-0.191	3.399	NA
Rodentibacter	0.334	0.115	2.170	NA
Sphingomonas-like	0.813	-0.916	1.420	NA
Intrasporangiaceae_genus	0.940	-1.455	1.465	NA
Gynurincola	0.054	1.276	3.401	NA
Mycoplasma	1.308	-1.251	0.962	NA
Lipomyces	0.316	-0.062	2.839	NA
Mariprofundus	0.047	1.231	3.401	NA
Salicibibacter	0.350	-0.206	3.399	NA
Urbifossiella	0.741	-1.018	2.517	NA
Metasolibacillus	0.775	-1.150	1.452	NA
Lignipirellula	0.509	1.285	3.043	NA
Slackia	0.631	-0.933	2.182	NA
Rhodovibrio	0.526	0.644	2.356	NA
Acidobacteriaceae_genus	0.311	0.092	2.829	NA
Nanogingivalis	0.227	0.162	2.168	NA
Aquariibacter	0.913	-1.242	1.482	NA
Acidimicrobium	0.273	3.290	3.394	NA
Runella	0.324	0.172	2.468	NA
Lindgomyces	0.470	-0.600	2.788	NA
Gallionella	0.693	-0.053	1.194	NA
Fodinicola	0.339	-0.240	3.153	NA
Fontibacillus	0.151	0.523	3.400	NA
Puteibacter	1.005	2.037	1.301	NA
Mesobacillus	1.096	-0.995	1.602	NA
Enterobacteria_phage_YYZ-2008_virus	0.009	1.743	3.402	NA
Calidifontimicrobium	0.522	1.758	3.392	NA
Elioraea	0.452	0.682	2.360	NA
Quatrionicoccus	0.245	0.099	3.399	NA
Thiofilum	1.021	-1.426	2.322	NA
Virgisporangium	0.313	-0.158	2.345	NA
Capsulimonas	0.344	-0.221	2.303	NA
Nisaea	0.072	1.069	3.401	NA
Oryzibacter	0.794	-1.239	2.343	NA
Segnochrobactrum	0.379	-0.138	2.809	NA
Spirilliplanes	0.536	4.259	3.389	NA
Malikia	0.578	-0.831	2.346	NA
Nigerium	0.539	-0.502	2.750	NA
Pleurocapsa	1.217	1.453	1.937	NA
Thalassolituus	1.194	-1.326	1.252	NA
Pseudovibrio	0.453	1.230	2.454	NA
Histoplasma	0.752	0.028	1.442	NA
Deferrisoma	0.101	0.821	3.401	NA
Pelobacter	1.250	2.209	1.898	NA
Escherichia_phage_vB_EcoS_ESCO41_virus	1.030	-0.948	1.633	NA
Papillibacter	0.041	1.287	3.401	NA
Acytostelium	1.048	-1.424	1.019	NA
Rickettsiales	0.188	0.375	2.907	NA
Dysosmobacter	0.526	-0.093	2.508	NA
Morchella	0.641	-0.959	2.766	NA
Neoroseomonas	0.161	0.599	3.400	NA
Tahibacter	0.454	-0.283	1.802	NA
Propionibacterium_phage_PHL041M10_virus	0.119	0.783	3.401	NA
Rugosimonospora	0.807	3.744	3.146	NA

Aff.	0.263	0.148	3.399	NA
Sporomusaceae_genus	0.444	-0.500	1.475	NA
Anaerostipes	0.356	-0.295	2.008	NA
Chloroflexia	0.169	0.430	3.400	NA
Mitsuokella	0.263	0.048	3.399	NA
Propionibacterium_phage_PHL301M00_virus	0.111	0.768	3.401	NA
Pseudenterobacter	0.515	-0.390	0.661	NA
Trujillella	0.289	0.148	2.841	NA
Atlanticothrix	0.252	0.064	2.871	NA
Hominisplanchenecus	0.033	2.172	3.401	NA
Vescimonas	0.025	1.515	3.402	NA
Thermaurantiacus	0.224	0.563	3.400	NA
Provencibacterium	0.230	0.160	3.399	NA
Teredinibacter	0.038	1.317	3.401	NA
Pseudidiomarina	0.129	1.028	2.098	NA
Polysphondylium	0.588	1.857	2.672	NA
Rhodophyticola	0.237	0.132	3.399	NA
Cucumibacter	0.022	1.596	3.402	NA
Scleromatobacter	0.399	-0.359	3.111	NA
Cucurbitaria	0.669	1.736	1.792	NA
Falseniella	0.389	-0.143	2.758	NA
Parapusillimonas	0.200	1.539	3.399	NA
Falcatimonas	0.017	1.596	3.402	NA
Lujinxingia	0.358	-0.305	2.007	NA
Occultella	0.037	1.447	3.402	NA
Nitrospira	0.572	1.094	2.246	NA
Ornithinicoccus	0.159	0.757	3.251	NA
Halovulum	1.008	2.067	1.762	NA
Microbacter	0.671	0.152	1.988	NA
Croceicoccus	0.549	-0.792	1.774	NA
Parapedobacter	0.475	-0.576	1.559	NA
Acidaminococcus	0.185	2.259	3.398	NA
Frigoriflavimonas	0.492	-0.638	2.332	NA
Geomonas	0.050	1.243	3.401	NA
Alkalihalophilus	0.129	0.646	3.400	NA
Hydrobacter	0.593	0.038	2.299	NA
Thermoleophilum	0.477	-0.376	2.729	NA
Pyricularia	0.300	0.152	2.823	NA
Humibacter	0.118	0.756	3.401	NA
Phytoactinopolyspora	0.463	-0.530	2.773	NA
Paenalcaligenes	0.760	2.502	0.988	NA
Neokomagataea	0.593	-0.869	3.398	NA
Drancourtella	0.294	0.245	3.111	NA
Larkinella	0.768	0.582	1.868	NA
Escherichia_phage_D6_virus	0.000	0.000	0.000	NA
Desulfoscipio	0.565	-0.810	2.517	NA
Saccharimonas	0.553	-0.299	2.256	NA
Neglectibacter	0.247	0.604	1.582	NA
Sinomonas	0.642	1.932	3.067	NA
Zoogloeaceae_genus	0.238	0.122	3.399	NA
Escherichia_phage_DTL_virus	0.756	-1.170	2.172	NA
Vampirovibrio	0.259	0.042	3.399	NA
Coccidioides	0.857	0.456	1.412	NA
Gramella	1.113	-0.329	0.982	NA
Pseudodesulfovibrio	0.057	1.668	3.402	NA
Simonsiella	0.622	1.401	2.467	NA
Allorhizobium	0.361	-0.300	3.144	NA
Pseudaestuariivita	0.064	1.103	3.401	NA
Macellibacteroides	0.431	-0.180	2.463	NA

Caenispirillum	0.400	-0.415	3.398	NA
Jeotgalibaca	1.102	-1.465	2.034	NA
Hydrotalea	0.322	1.396	3.124	NA
Desnuesiella	0.296	1.106	3.401	NA
BeAn_58058_virus	0.384	0.764	1.326	NA
Yinghuangia	0.547	-0.388	1.769	NA
Congregibacter	0.755	0.755	1.150	NA
Enterobacteria_phage_Sf6_virus	0.000	0.000	0.000	NA
Methyloradius	0.301	2.899	2.910	NA
Robiginitalea	0.271	0.952	3.092	NA
Usitatibacter	0.008	1.695	3.402	NA
Haloechinothrix	0.329	0.162	2.772	NA
Calorimonas	0.022	1.491	3.402	NA
Aestuariivirga	0.693	-1.061	2.529	NA
Tistrella	0.240	0.118	3.399	NA
Sphaerisporangium	0.405	-0.354	3.128	NA
Haliea	0.570	0.613	1.602	NA
Siphonobacter	1.123	-1.162	2.471	NA
Nioella	0.094	0.867	3.401	NA
Shouchella	0.581	1.345	3.194	NA
Chloroflexales	0.074	1.015	3.401	NA
Arenivirga	0.339	1.124	3.397	NA
Ktedonobacter	0.238	0.128	3.399	NA
Anaerobiospirillum	0.687	-0.465	2.714	NA
Pelistega	0.345	0.424	3.399	NA
Arboricoccus	0.363	1.410	2.637	NA
Roseibium	0.811	-1.232	1.807	NA
Propionispora	0.201	0.326	3.400	NA
Odoribacter	0.475	-0.609	2.785	NA
Miniphocaeibacter	0.034	1.386	3.401	NA
Paenarthrobacter	0.392	0.382	2.757	NA
Betaproteobacteria	0.088	1.001	3.401	NA
Betaproteobacterium_AAP65	0.934	-1.010	1.282	NA
Albitalea	0.203	0.274	3.266	NA
Trichococcus	0.181	0.373	3.331	NA
Rhodocyclus	0.572	-0.777	1.763	NA
Tetzosporium	0.333	-0.165	3.399	NA
Mesomycoplasma	0.567	-0.467	2.490	NA
Elstera	0.451	-0.550	2.781	NA
Haloferax	0.578	-0.752	2.516	NA
Brooklawnia	0.606	0.614	2.525	NA
Aquirhabdus	0.022	1.841	3.402	NA
Neptunicoccus	0.118	0.709	3.400	NA
Methylocaldum	0.395	0.368	1.977	NA
Limnobaculum	0.794	1.220	1.278	NA
Viridibacillus	0.463	-0.565	2.326	NA
Aggregatilinea	0.220	0.196	3.400	NA
Primorskyibacter	0.613	1.170	1.094	NA
Fontimonas	0.265	0.020	3.221	NA
Aceticella	0.332	2.371	3.394	NA
Argonema	0.623	0.914	2.516	NA
Flagellatimonas	0.126	0.659	3.400	NA
Weeksella	0.627	0.426	3.399	NA
Gloeothece	0.000	0.000	0.000	NA
Camelimonas	0.584	-0.851	2.532	NA
Silanimonas	0.443	1.871	3.101	NA
Inquilinus	0.544	0.101	1.704	NA
Atopococcus	0.184	0.562	3.400	NA
Prosthecomicrobium	0.590	-0.510	2.248	NA

Propionibacterium_phage_PHL117M01_virus	0.268	0.348	3.400	NA
Carboxylicivirga	0.110	0.989	3.401	NA
Thermopolyspora	0.034	1.491	3.402	NA
Filamentous	0.701	0.037	2.101	NA
Rubricoccus	0.152	1.306	3.401	NA
Ferrovum	0.569	-0.806	2.776	NA
Tepidanaerobacter	0.405	0.012	3.399	NA
Sulfuriferula	0.273	-0.012	3.399	NA
Idiomarinaceae_genus	0.023	1.795	3.402	NA
Chryseosolibacter	0.194	0.315	3.400	NA
Komarekiella	0.170	0.649	3.400	NA
Pelovirga	0.002	1.761	3.402	NA
Pirellulimonas	0.144	0.562	3.400	NA
Propionibacterium_phage_PHL116M00_virus	0.473	-0.399	3.093	NA
Paenisporosarcina	0.629	0.981	3.400	NA
Salegentibacter	0.151	0.525	3.400	NA
Muribaculaceae_genus	0.949	1.772	1.477	NA
Arachidicoccus	0.350	0.009	3.095	NA
Sulfuricystis	0.204	1.990	3.399	NA
Luteipulveratus	0.361	1.407	2.995	NA
Chryseolinea	0.170	1.175	3.401	NA
Pontibrevibacter	0.195	0.309	3.400	NA
Hanamia	0.446	-0.340	3.398	NA
Penicilliopsis	0.668	-0.433	2.713	NA
Podospora	0.954	-0.811	0.973	NA
Grimontia	0.335	0.092	0.909	NA
Thioclava	0.294	0.599	2.720	NA
Propionibacterium_phage_SKKY_virus	0.362	1.385	3.397	NA
Citreimonas	0.088	0.922	3.401	NA
Propionibacterium_phage_PAD20_virus	0.255	1.496	3.399	NA
Planococcaceae_genus	0.288	-0.062	3.399	NA
Caldicellulosiruptor	0.237	0.127	3.399	NA
Hartmannibacter	0.288	-0.068	3.399	NA
Helicobacter	0.471	-0.396	2.510	NA
Verticiella	0.467	2.552	2.909	NA
Pseudescherichia	0.152	0.539	2.968	NA
Akanthomyces	0.437	-0.514	3.398	NA
Plasticicumulans	0.111	0.758	3.401	NA
Dongia	0.686	-1.041	2.032	NA
Escherichia_phage_520873_virus	0.000	0.000	0.000	NA
Bergeyella	0.183	0.364	3.309	NA
Tersicoccus	1.189	2.985	2.427	NA
Stenoxybacter	0.289	-0.016	3.399	NA
Cronobacter_phage_vB_CsaM_GAP32_virus	0.325	-0.123	3.139	NA
Plesiocystis	0.041	1.544	3.402	NA
Holdemanella	0.771	2.756	2.077	NA
Torulaspora	0.598	-0.880	3.071	NA
Sulfuritalea	0.539	-0.754	3.398	NA
Streptobacillus	0.298	0.775	3.398	NA
Viadribacter	0.000	0.000	0.000	NA
Betaproteobacterium_AAP121	0.906	-0.907	1.271	NA
Jaminaea	0.480	-0.576	3.398	NA
Propionibacterium_phage_P100D_virus	0.085	0.931	3.401	NA
Asanoa	0.412	-0.217	3.399	NA
Enterocloster	0.679	3.076	1.982	NA
Petrotoga	0.426	-0.300	2.493	NA
Wenjunlia	0.648	-0.687	2.475	NA
Salinisphaera	0.252	0.698	1.077	NA
Lewinella	0.693	1.668	0.662	NA

Chloroflexus	0.443	-0.532	2.798	NA
Buchananella	0.056	1.596	3.402	NA
Rhodocyclales	0.940	4.230	2.300	NA
Frisingicoccus	0.052	1.223	3.401	NA
Pseudanabaena	0.202	2.971	3.397	NA
Effusibacillus	0.123	0.934	3.401	NA
Blattabacterium	0.399	-0.217	2.502	NA
Corticibacterium	0.417	-0.062	2.481	NA
Paramesorhizobium	0.152	1.244	3.401	NA
Betaproteobacterium_AAP99	0.003	1.743	3.402	NA
Oceanotoga	0.000	0.000	0.000	NA
Salmonella_phage_SJ46_virus	0.000	0.000	0.000	NA
Roseibaca	0.208	0.815	2.880	NA
Allofustis	0.129	0.641	3.400	NA
Phialemonium	0.746	-1.149	2.333	NA
Thermaerobacter	0.642	-0.966	2.535	NA
Phaseolus_vulgaris_endornavirus	0.000	0.000	0.000	NA
Defluviimonas	0.700	-1.063	1.791	NA
Syntrophomonas	0.225	0.173	3.399	NA
Paludicola	0.106	0.788	3.401	NA
Kaustia	0.000	0.000	0.000	NA
Pararobbsia	0.017	1.602	3.402	NA
Melittangium	0.241	0.114	3.245	NA
Robinsoniella	0.201	0.286	3.400	NA
