# Supplementary material for: Shotgun Metagenome Analysis of Two Schizaphis graminum Biotypes over Time With and Without Carried Cereal Yellow Dwarf Virus
Source: Insects. 2025 May 23;16(6):554. doi: 10.3390/insects16060554 (PMC12193481; doi:10.3390/insects16060554)
Supplement: Supplementary file 1 [file insects-16-00554-s001.zip › Table S9.pdf]

Table S9. DESeq2 results for comparison of early versus late relative time, arranged by log_2 fold change.

Genus	BaseMean	Log2FC	LFCSE	Padj
Allomeiothermus	372.615	-7.886	0.603	5.037e-18
Armatimonadetes	144.045	-7.737	0.697	9.626e-14
Craterilacuibacter	127.435	-6.634	0.499	2.151e-24
Kallotenue	98.738	-6.624	0.636	1.277e-14
Oryzomicrobium	63.319	-6.526	0.434	5.465e-54
Phytophthora	90.681	-6.197	0.440	1.074e-33
Cedecea	62.532	-5.928	0.409	2.700e-46
Heterobasidion	23.873	-5.876	0.973	1.919e-05
Postia	14.857	-5.275	0.981	6.212e-05
Neoantrodia	12.936	-5.135	1.035	2.090e-04
Gloeophyllum	12.295	-5.057	1.129	9.261e-04
Nanosynbacter	12.801	-4.984	0.742	3.101e-07
Marinobacterium	1297.016	-4.959	0.275	3.670e-39
Anaerococcus	995.459	-4.919	0.584	8.874e-09
Ichthyophthirius	11.762	-4.898	0.614	9.867e-12
Lamprocystis	7995.423	-4.828	0.201	3.554e-67
Dysgonamonadaceae_genus	19.860	-4.807	0.890	2.111e-04
Punctularia	16.189	-4.794	1.082	1.164e-02
Serpula	14.437	-4.749	1.317	1.259e-02
Chelativorans	9.704	-4.726	0.944	9.610e-05
Pyrinomonas	9.505	-4.692	0.996	2.672e-04
Levyella	9.603	-4.638	1.290	7.455e-03
Gleimia	229.791	-4.637	0.366	4.646e-22
Shigella	210548.549	-4.600	0.130	1.309e-146
Terrisporobacter	682.815	-4.547	0.200	2.603e-70
Bipolaris	9.251	-4.524	0.934	1.484e-04
Musicola	448.106	-4.515	0.304	1.128e-28
Escherichia	61277.548	-4.484	0.134	2.384e-134
Paucilactobacillus	8.021	-4.454	0.664	9.856e-09
Seramator	7.459	-4.362	0.851	3.164e-05
Trametes	13.922	-4.291	0.899	3.655e-04
Facklamia	31.337	-4.277	0.465	2.380e-15
Thalassiosira	9.266	-4.240	0.696	1.523e-06
Thermalbibacter	6.902	-4.229	1.150	3.974e-03
Domibacillus	6.862	-4.216	1.356	1.708e-02
Parainfluenza_virus_5	6.913	-4.210	1.295	1.220e-02
Actinobacillus	273.019	-4.205	0.253	1.251e-39
Stomatobaculum	6.438	-4.135	0.908	2.066e-04
Finegoldia	162.375	-4.092	0.656	9.412e-06
Citrobacter	14367.687	-4.073	0.160	1.989e-82
Gallibacter	20.094	-4.046	0.970	2.463e-03
Rhodofomes	5.979	-4.033	0.975	7.783e-04
Desulfitobacterium	6.033	-4.029	0.902	2.389e-04
Ruficoccus	65.274	-4.028	0.334	1.181e-22
Myceligenerans	6.325	-4.000	0.704	1.111e-06
Dioszegia	14.284	-3.974	1.088	7.918e-03
Desulfosporosinus	5.604	-3.959	0.776	1.527e-05
Tetrahymena	5.684	-3.938	0.789	2.425e-05
Clavibacter	5.527	-3.912	1.087	3.649e-03
Thermothelomyces	6.239	-3.906	1.753	1.018e-01
Aureococcus	5.453	-3.896	0.977	1.059e-03
Human_adenovirus_2	5.562	-3.892	0.878	2.159e-04
Tannerella	323.303	-3.882	0.231	3.066e-42
Perkinsus	5.227	-3.812	0.705	2.488e-06
Lentisphaera	29.992	-3.778	0.288	6.220e-43

Enterobacter	9658.205	-3.768	0.186	1.803e-54
Raoultella	68.520	-3.763	0.286	1.435e-30
Glarea	5.933	-3.760	0.838	1.893e-04
Fannyhessea	5.504	-3.754	1.332	2.973e-02
Cloacibacterium	69.593	-3.752	0.433	6.848e-12
Caldifermentibacillus	6.086	-3.730	1.239	2.302e-02
Fibrisoma	27.200	-3.691	0.660	2.090e-04
Cutaneotrichosporon	11.862	-3.639	1.030	8.924e-03
Luteibacter	4.401	-3.605	0.932	1.059e-03
Saccharibacteria	4.408	-3.593	1.132	8.721e-03
Enterobacteria_phage_phi80_virus	4.380	-3.563	1.015	3.291e-03
Kurthia	83.993	-3.526	0.330	7.456e-18
Phanerochaete	10.953	-3.524	0.955	5.317e-03
Panacagrimonas	4.713	-3.517	1.597	9.770e-02
Turicibacter	17.622	-3.478	1.379	7.701e-02
Tumebacillus	31.849	-3.440	0.435	5.017e-11
Paludibacterium	3.956	-3.439	0.896	9.465e-04
Selenomonas	6.804	-3.421	0.673	1.150e-05
Alcaligenes	8.410	-3.416	0.728	1.351e-04
Thermomicrobium	3.852	-3.384	0.987	3.446e-03
Ethanoligenens	3.764	-3.344	2.288	2.536e-01
Dialister	4.741	-3.329	1.115	1.365e-02
Pleomorphomonas	5.725	-3.315	1.306	5.099e-02
Kitasatospora	19.546	-3.300	0.647	6.450e-05
Herbinix	3.656	-3.300	1.613	9.664e-02
Hallella	3.747	-3.288	1.041	7.022e-03
Allomuricauda	54.976	-3.274	0.349	1.305e-14
Azotobacter	93.627	-3.272	0.485	2.267e-07
Pusillimonas	1729.460	-3.261	0.169	9.476e-54
Enterobacteriaceae_genus	34.309	-3.251	0.405	2.054e-11
Microdochium	3.486	-3.248	0.888	1.348e-03
Anaeroglobus	4.318	-3.239	0.905	4.627e-03
Exserohilum	3.437	-3.226	1.403	5.442e-02
Diaphorobacter	35.714	-3.212	0.416	2.114e-10
Neohortaea	3.383	-3.204	1.175	2.000e-02
Pectinatus	4.713	-3.200	0.825	2.730e-03
Tissierellia	8.196	-3.197	1.411	1.035e-01
Laccaria	4.694	-3.192	0.880	6.312e-03
Porphyromonadaceae_genus	10.907	-3.178	0.726	5.020e-04
Betaproteobacterium_FWI2	1603.185	-3.177	0.316	7.136e-15
Phaeodactylum	12.627	-3.165	0.628	3.890e-05
Aphanomyces	5.189	-3.157	0.718	2.154e-04
Rummeliibacillus	3.322	-3.156	0.967	4.254e-03
Dictyostelium	8.462	-3.153	0.951	2.142e-02
Gaiella	11.733	-3.141	1.120	4.043e-02
Caldibacillus	3.114	-3.112	0.947	3.753e-03
Wolbachia	62.605	-3.111	0.419	2.638e-09
Naegleria	20.303	-3.099	0.578	5.061e-05
Aureispira	3.069	-3.076	0.903	2.514e-03
Fibroporia	7.444	-3.076	1.288	8.094e-02
Erwinia	905.375	-3.074	0.264	3.106e-20
Lysinibacillus	127.976	-3.065	0.373	1.184e-10
Proteus	179.703	-3.054	0.353	1.116e-11
Yersinia	118.324	-3.025	0.412	1.120e-08
Elizabethkingia	205.340	-3.015	0.204	8.493e-36
Enterobacteria_phage_T4_virus	2.981	-3.015	1.279	4.221e-02
Rheinheimera	4037.545	-2.975	0.196	4.079e-34
Saprolegnia	9.754	-2.973	0.734	1.332e-03
Alkaliphilus	2.898	-2.969	1.659	1.320e-01

Putridiphycobacter	3.734	-2.963	0.976	9.023e-03
Pectobacterium	3.282	-2.957	0.727	2.803e-04
Propionimicrobium	84.480	-2.953	0.560	6.536e-05
Tuber	2.943	-2.948	1.430	7.744e-02
Wenzhouxiangella	3.178	-2.946	0.955	7.920e-03
Tautonia	3.902	-2.904	1.850	2.135e-01
Phreatobacter	2.827	-2.890	1.031	1.221e-02
Westeberhardia	22.020	-2.886	0.484	8.453e-07
Drepanopeziza	2.702	-2.884	1.246	4.253e-02
Loigolactobacillus	19.382	-2.883	1.130	8.693e-02
Minimicrobia	2.704	-2.880	1.132	2.431e-02
Lutimaribacter	14.713	-2.868	0.654	1.005e-03
Grosmannia	2.991	-2.867	1.712	1.768e-01
Enterobacteria_phage_RTP_virus	2.620	-2.856	1.050	1.498e-02
Zoogloea	42.077	-2.850	0.374	2.460e-10
Emticicia	2.634	-2.843	1.654	1.414e-01
Homoserinimonas	5.839	-2.840	0.887	1.786e-02
Klebsiella	1531.102	-2.829	0.131	2.295e-72
Collinsella	10.764	-2.822	0.982	1.061e-01
Edwardsiella	64.525	-2.811	0.353	1.265e-10
Singulisphaera	6.434	-2.791	1.044	3.769e-02
Microbispora	62.361	-2.777	0.288	1.028e-16
Rhizoctonia	10.173	-2.768	0.973	3.160e-02
Leptomonas	2.477	-2.765	1.230	4.530e-02
Acidisphaera	2.480	-2.765	1.634	1.434e-01
Gemmatimonas	2.580	-2.739	1.505	1.084e-01
Escherichia_phage_phiV10_virus	2.464	-2.729	1.139	3.103e-02
Thiolapillus	14.428	-2.728	0.850	2.431e-02
Eutypa	3.140	-2.717	0.996	2.209e-02
Reyranella	10.238	-2.712	0.682	1.384e-03
Aquisphaera	2.365	-2.691	1.486	1.119e-01
Rahnella	196.146	-2.689	0.289	3.862e-14
Peptoniphilus	189.828	-2.677	0.583	5.992e-04
Phytobacter	11.988	-2.676	0.553	1.110e-03
Thermicanus	19.990	-2.673	1.080	7.812e-02
Aedoeadaptatus	2.336	-2.673	1.574	1.379e-01
Pseudoramibacter	88.151	-2.650	0.570	4.228e-04
Secondary	3.884	-2.621	0.600	1.774e-04
Pimelobacter	6.125	-2.605	0.838	2.302e-02
Pauljensenia	148.330	-2.603	0.474	2.342e-05
Insolitispirillum	2.216	-2.578	1.294	7.505e-02
Marinobacter	150.235	-2.572	0.217	9.748e-24
Methylocystis	2.390	-2.570	1.013	2.431e-02
Pleionea	5.060	-2.563	0.917	2.269e-02
Pelorhabdus	4.813	-2.559	0.848	2.853e-02
Xanthomonadaceae_genus	3.316	-2.558	1.037	3.399e-02
Coraliihabitans	13.216	-2.554	0.785	1.186e-02
Microbacteriaceae_genus	2.390	-2.552	1.362	1.103e-01
Conyzicola	2.118	-2.550	1.371	9.607e-02
Ensifer	36.688	-2.548	0.330	4.298e-11
Oceanospirillum	5.544	-2.545	0.786	1.335e-02
Stereum	22.521	-2.545	0.916	4.519e-02
Xanthocytophaga	2.896	-2.531	1.338	1.020e-01
Type-D_symbiont_of_Plautia_stali	2.107	-2.520	3.395	5.438e-01
UNVERIFIED_CONTAM:	2.089	-2.506	1.442	1.183e-01
Sanguibacter	2.037	-2.494	1.443	1.191e-01
Methylosinus	1.999	-2.487	1.264	7.382e-02
Cupidesulfovibrio	28.579	-2.476	0.452	1.083e-05
Dyella	23.540	-2.476	0.403	3.370e-07

Aliiruegeria	16.063	-2.472	0.673	4.138e-03
Lichenibacterium	2.044	-2.470	1.617	1.718e-01
Kosakonia	21.067	-2.470	0.437	2.239e-06
Hafnia	2.018	-2.468	0.609	6.374e-05
Cadophora	1.997	-2.459	1.303	8.693e-02
Gammaproteobacteria	3.436	-2.457	1.148	8.828e-02
Alloalcanivorax	2.215	-2.454	1.163	5.547e-02
Microcystis	6710.710	-2.446	0.378	3.956e-07
Pestalotiopsis	18.985	-2.439	0.607	1.364e-03
Anaeromyxobacter	2.178	-2.435	1.445	1.366e-01
Polaribacter	15.580	-2.433	0.584	8.207e-04
Frankineae	1.968	-2.417	2.243	3.464e-01
Salipaludibacillus	45.569	-2.411	0.539	4.841e-04
Thermus	128.332	-2.411	0.446	2.227e-05
Bacteroides	124.758	-2.409	0.203	1.089e-24
Halovibrio	10.136	-2.393	0.740	4.029e-02
Talaromyces	10.629	-2.386	0.825	2.421e-02
Desarmillaria	3.332	-2.378	0.976	3.595e-02
Okeania	8.897	-2.378	0.923	4.590e-02
Paludisphaera	5.695	-2.377	1.066	8.431e-02
Actinobacteria	1.870	-2.375	1.838	2.482e-01
Paracoccidioides	3.608	-2.361	0.994	4.769e-02
Xenorhabdus	3.486	-2.361	0.560	7.713e-05
Azomonas	2.339	-2.359	1.022	3.948e-02
Chelatococcus	6.249	-2.359	1.101	1.006e-01
Parvimonas	4.539	-2.359	0.959	4.392e-02
Algiphilus	5.689	-2.355	1.023	7.414e-02
Melaminivora	1.837	-2.348	1.608	1.845e-01
Serratia	233.668	-2.344	0.231	1.922e-17
Aureibaculum	51.891	-2.339	0.406	3.243e-06
Aquabacter	1.821	-2.339	1.375	1.169e-01
Leyella	1.814	-2.336	1.454	1.396e-01
Gloeocapsa	4.503	-2.333	1.147	1.786e-01
Acidaminobacter	5.035	-2.320	0.753	2.644e-02
Neomicrococcus	4.911	-2.317	0.815	1.979e-02
Pararhizobium	8.228	-2.317	0.762	1.624e-02
Ruminococcus	45.967	-2.299	0.632	6.402e-03
Lachnoanaerobaculum	14.139	-2.297	0.482	7.905e-05
Halalkalibacterium	3.247	-2.290	1.025	7.760e-02
Rhizopus	1.764	-2.288	1.697	2.180e-01
Plasmopara	6.866	-2.287	0.819	2.473e-02
Aerococcus	77.226	-2.279	0.289	2.161e-11
Nannochloropsis	1.981	-2.279	1.449	1.677e-01
Jatrophihabitans	10.131	-2.278	1.233	2.055e-01
Afipia	1012.669	-2.270	0.215	1.635e-18
Actinophytocola	1.747	-2.266	1.791	2.513e-01
Levilactobacillus	84.614	-2.264	0.530	9.921e-04
Brasilonema	1.716	-2.261	1.662	2.122e-01
Thalassotalea	1.950	-2.258	1.101	7.070e-02
Vogesella	4.180	-2.254	1.092	1.014e-01
Dorea	12.831	-2.250	0.644	4.988e-03
Bacteriovorax	1.730	-2.248	1.594	1.936e-01
Hemiselmis	1.714	-2.247	1.852	2.706e-01
Halophilic	1.709	-2.228	1.694	2.254e-01
Pectobacterium_phage_CBB_virus	1.674	-2.223	1.609	2.039e-01
Mogibacterium	9.735	-2.222	0.744	2.431e-02
Chrysosporum	12.790	-2.214	0.846	5.312e-02
Moniliophthora	4.949	-2.206	1.064	1.011e-01
Cryptosporidium	15.505	-2.197	0.583	7.675e-03

Moorena	7.922	-2.188	0.988	8.828e-02
Paraferrimonas	4.903	-2.179	0.786	2.258e-02
Enterobacteria_phage_vB_EcoS_ACG-M12_virus	1.666	-2.177	1.311	1.141e-01
Fomitiporia	9.334	-2.176	0.939	1.018e-01
Coleofasciculus	1.811	-2.175	1.448	1.802e-01
Aliihoeflea	2.483	-2.174	1.637	5.367e-01
Pseudacidovorax	63.479	-2.174	0.330	4.633e-08
Castellaniella	1.632	-2.173	1.674	2.289e-01
Glaciibacter	16.545	-2.167	0.756	6.022e-02
Qaidamihabitans	4.779	-2.160	1.335	2.122e-01
Flavisolibacter	1.622	-2.138	1.457	1.646e-01
Gemmatirosa	1.806	-2.133	1.869	3.206e-01
Bacidia	3.442	-2.132	1.080	9.964e-02
Oceanimonas	1.818	-2.128	0.805	1.065e-02
Arsukibacterium	2.520	-2.122	1.478	1.438e-01
Tychonema	1.638	-2.118	1.318	1.205e-01
Parerythrobacter	4.100	-2.112	1.536	7.146e-01
Babesia	5.122	-2.107	0.756	2.044e-02
Cryptomonas	1.703	-2.101	1.440	1.545e-01
Sporisorium	1.536	-2.099	1.487	1.831e-01
Propionibacteriaceae_genus	1.513	-2.096	1.559	2.055e-01
Aureimonas	21.201	-2.089	0.575	4.254e-03
Haladaptatus	2.468	-2.087	0.933	5.102e-02
Phycomyces	7.189	-2.085	1.508	3.100e-01
Prevotella	338.175	-2.080	0.317	8.113e-08
Eikenella	8.680	-2.077	0.673	1.428e-02
Plantibacter	3.609	-2.076	1.123	1.369e-01
Myxococcales	1.509	-2.074	2.228	3.964e-01
Winkia	9.874	-2.071	0.901	8.324e-02
Enterobacteria_phage_vB_EcoS_IME542_virus	2.043	-2.065	1.183	9.647e-02
TM7	7.135	-2.064	0.771	4.519e-02
Salifodinibacter	1.493	-2.063	1.958	3.288e-01
Alloscardovia	56.324	-2.026	0.456	3.887e-04
Robertmurraya	9.332	-2.021	0.743	3.146e-02
Duffyella	4.145	-2.014	0.892	9.559e-02
Terrihabitans	1.434	-2.011	2.196	3.986e-01
Gemella	167.060	-2.010	0.464	7.336e-04
Solobacterium	7.141	-2.004	0.807	4.497e-02
Morococcus	313.297	-1.992	0.278	2.451e-09
Aurantimonas	8.757	-1.987	0.657	3.387e-02
Salinibacterium	8.132	-1.986	0.741	3.232e-02
Prevotellaceae_genus	6.860	-1.983	0.810	4.927e-02
Naasia	3.443	-1.981	1.284	2.055e-01
Hahella	2.380	-1.981	0.893	9.258e-02
Weizmannia	1.405	-1.979	2.560	4.844e-01
Faecalibacterium	8.180	-1.977	0.954	1.115e-01
Wolinella	1.486	-1.965	1.956	3.313e-01
Mycoplasmopsis	1.529	-1.963	1.291	1.307e-01
Apiotrichum	11.988	-1.961	0.913	1.047e-01
Paramagnetospirillum	1.385	-1.959	1.669	2.654e-01
Entamoeba	57.649	-1.958	0.624	1.797e-02
Methanotrophic	6.831	-1.952	0.939	1.025e-01
Propioniciclava	26.542	-1.946	0.665	2.532e-02
Geodermatophilaceae_genus	1.390	-1.932	1.732	2.816e-01
Sodalis	1.787	-1.930	0.544	3.863e-04
Mollisia	4.514	-1.926	0.942	1.011e-01
Aliidiomarina	6.184	-1.922	0.718	2.532e-02
Ustilago	1.359	-1.921	1.500	NA
Acidihalobacter	28.945	-1.887	0.608	1.892e-02

Roseibacterium	113.257	-1.884	0.520	5.648e-03
Verrucomicrobia	1.670	-1.882	1.855	5.338e-01
Roseococcus	1.344	-1.879	1.591	NA
Luteococcus	1.362	-1.876	2.053	NA
Sandaracinobacteroides	6.329	-1.874	0.909	3.370e-01
Fusobacterium	181.144	-1.871	0.192	1.614e-17
Micromonospora	101.944	-1.871	0.503	3.906e-03
Magnetospirillum	1.714	-1.870	1.116	1.126e-01
Gemmata	1.307	-1.859	1.742	NA
Psilocybe	1.438	-1.857	1.023	8.535e-02
Scedosporium	1.465	-1.843	1.986	3.914e-01
Globicatella	1.277	-1.843	2.057	NA
Lachnoclostridium	1.274	-1.842	2.350	NA
Lancefieldella	4.241	-1.836	1.457	5.092e-01
Saccharibacillus	2.894	-1.836	1.558	3.313e-01
Desertibacillus	1.301	-1.833	1.728	NA
Extensimonas	2.701	-1.831	1.176	2.207e-01
Simian_virus_40	1.423	-1.824	0.975	5.213e-02
Leishmania	4.184	-1.817	1.158	2.686e-01
Yonghaparkia	1.392	-1.813	1.579	2.938e-01
Azospira	27.304	-1.810	0.358	2.075e-05
Leptolyngbya	41.961	-1.809	0.464	2.016e-03
Coprobacillus	1.834	-1.808	1.422	5.392e-01
Lachnospiraceae_genus	22.274	-1.808	0.527	1.349e-02
Aquimarina	1.439	-1.804	2.075	3.964e-01
Coprinopsis	1.211	-1.793	1.743	NA
Luteitalea	11.215	-1.768	1.108	2.400e-01
Daldinia	4.862	-1.766	1.236	2.646e-01
Sordaria	1.433	-1.766	1.383	2.177e-01
Paecilomyces	3.711	-1.756	1.062	1.783e-01
Hyaloscypha	3.026	-1.751	1.036	1.547e-01
Atopobium	11.220	-1.751	0.759	9.663e-02
Marisediminicola	1.178	-1.742	2.351	NA
Flavobacteriaceae_genus	1.172	-1.741	1.601	NA
Saliphagus	1.228	-1.740	1.337	NA
Glaciihabitans	1.333	-1.736	1.646	NA
Nannocystis	1.346	-1.736	2.053	NA
Lawsonella	200.793	-1.732	0.455	3.009e-03
Brevibacillus	1.944	-1.728	1.108	1.554e-01
Granulicatella	30.965	-1.721	0.536	1.349e-02
Rubrobacter	16.449	-1.720	0.746	8.840e-02
Mannheimia	5.616	-1.716	0.649	2.711e-02
Orbilia	13.707	-1.708	1.029	2.262e-01
Nanoperiomorbus	1.137	-1.706	1.641	NA
Pasteurella	1.606	-1.703	0.785	3.334e-02
Nitrososphaera	1.144	-1.702	2.535	NA
Terriglobus	1.251	-1.702	1.407	NA
Frankia	114.940	-1.696	0.309	5.535e-06
Mangrovibacillus	2.576	-1.693	1.427	2.991e-01
Rhodobacter	84.201	-1.692	0.244	1.790e-09
Pseudokineococcus	8.737	-1.685	1.423	3.891e-01
Granulicella	1.176	-1.680	2.184	NA
Faecalimonas	1.189	-1.676	1.819	NA
Pelagibacterium	4.466	-1.674	1.281	3.102e-01
Marinomonas	8.179	-1.669	1.100	2.536e-01
Pseudogymnoascus	6.848	-1.653	1.150	2.746e-01
Nitratireductor	1.356	-1.650	1.158	NA
Williamsia	113.724	-1.649	0.512	1.335e-02
Myroides	1.084	-1.634	2.173	NA

Lautropia	241.645	-1.633	0.196	3.884e-13
Fluviicola	6.799	-1.632	0.989	2.020e-01
Hoyosella	1.072	-1.621	2.533	NA
Ilyonectria	3.065	-1.620	1.285	1.000e+00
Protofrankia	75.565	-1.616	0.457	5.740e-03
Caulobacter	1070.743	-1.605	0.241	1.988e-08
Chroococcidiopsis	2.325	-1.605	1.055	2.146e-01
Jeotgalibacillus	1.208	-1.591	1.229	NA
Hydrogenophilus	2.405	-1.589	1.748	5.508e-01
Pseudoflavonifractor	1.639	-1.582	1.172	2.318e-01
Blastomonas	151.122	-1.582	0.317	3.930e-05
Riemerella	4.363	-1.581	1.072	2.375e-01
Bacteroidetes	1.190	-1.580	2.168	NA
Methylocapsa	1.039	-1.571	1.360	NA
Arsenophonus	1.327	-1.565	0.762	NA
Citromicrobium	6.385	-1.562	0.975	2.124e-01
Rosenbergiella	1.613	-1.559	0.839	8.150e-02
Catenulispora	1.103	-1.554	2.734	NA
Qipengyuania	66.264	-1.550	0.486	1.317e-02
Lacrimispora	1.596	-1.547	1.866	5.235e-01
Haliangium	2.455	-1.543	1.753	1.000e+00
Pseudorhizobium	3.953	-1.543	1.299	1.000e+00
Acidiphilium	1.247	-1.537	1.652	NA
Sporothrix	1.005	-1.536	1.814	NA
Chondromyces	1.551	-1.531	1.944	8.933e-01
Intestinirhabdus	0.996	-1.531	0.548	NA
Bdellovibrio	0.992	-1.529	1.469	NA
Actibacterium	0.985	-1.527	1.530	NA
Dactylellina	1.676	-1.526	1.621	4.620e-01
Lacibacter	2.371	-1.521	2.636	6.420e-01
Acidocella	1.112	-1.516	1.651	NA
Agarivorans	68.212	-1.514	0.641	8.461e-02
Marichromatium	2.010	-1.513	0.730	5.394e-02
Truncatella	1.191	-1.506	1.656	NA
Rathayibacter	27.104	-1.505	0.749	1.412e-01
Moritella	186.504	-1.503	0.148	5.831e-20
Aureobasidium	16.662	-1.496	0.735	1.451e-01
Eremococcus	4.175	-1.494	1.040	2.403e-01
Negativicoccus	1.337	-1.489	3.396	NA
Alloprevotella	38.025	-1.487	0.535	3.146e-02
Ogataea	1.303	-1.485	1.588	NA
Intrasporangium	1.015	-1.483	2.042	NA
Oligella	1.269	-1.482	2.295	NA
Conexibacter	13.570	-1.479	0.854	2.155e-01
Phytoplasma	62.472	-1.469	0.334	2.803e-04
Fusibacter	1.572	-1.467	2.003	5.117e-01
Jeotgalibaca	1.102	-1.465	2.034	NA
Pseudogemmobacter	0.944	-1.464	2.531	NA
Nevskia	5.606	-1.463	1.256	3.741e-01
Intrasporangiaceae_genus	0.940	-1.455	1.465	NA
Meiothermus	30.189	-1.453	0.655	9.958e-02
Clostridium	979.039	-1.451	0.171	1.711e-13
Isoalcanivorax	1.062	-1.441	2.024	NA
Rhodovulum	0.928	-1.436	1.535	NA
Aquamicrobium	62.955	-1.427	0.376	3.350e-03
Thiofilum	1.021	-1.426	2.322	NA
Acytostelium	1.048	-1.424	1.019	NA
Glaesserella	0.891	-1.416	1.046	NA
Azovibrio	0.915	-1.409	1.724	NA

Aciditerrimonas	0.899	-1.409	1.923	NA
Bradyrhizobium	4376.605	-1.407	0.162	4.729e-14
Schaalia	24.329	-1.407	0.546	4.530e-02
Trichoderma	47.821	-1.394	0.787	2.055e-01
Halococcus	1.219	-1.388	1.055	NA
Calothrix	3.536	-1.385	1.088	3.100e-01
Epithele	0.901	-1.380	1.402	NA
Alkalicoccobacillus	1.046	-1.375	0.936	NA
Acidothermus	1.064	-1.372	1.914	NA
Fervidibacillus	1.551	-1.369	2.375	6.420e-01
Butyrivibrio	0.998	-1.357	1.932	NA
Rhodotorula	32.855	-1.355	0.645	1.214e-01
Stigmatella	0.992	-1.355	2.729	NA
Saccharopolyspora	62.086	-1.354	0.235	5.843e-07
Nanosynsacchari	0.896	-1.353	1.684	NA
Halobacillus	0.907	-1.352	1.368	NA
Stappia	1.350	-1.351	1.527	NA
Aggregatibacter	18.736	-1.344	0.509	3.503e-02
Streptoalloteichus	1.111	-1.338	1.519	NA
Photobacterium	11.366	-1.337	0.572	7.414e-02
Luteolibacter	24.040	-1.334	0.476	4.043e-02
Pleurotus	2.527	-1.330	1.260	3.760e-01
Alicycliphilus	12.250	-1.330	0.539	5.511e-02
Micavibrio	0.862	-1.326	2.019	NA
Thalassolituus	1.194	-1.326	1.252	NA
Pseudomassariella	0.846	-1.323	2.338	NA
Pengzhenrongella	1.275	-1.317	1.733	NA
Peptostreptococcus	6.314	-1.315	0.804	1.936e-01
Allocoleopsis	0.836	-1.309	2.011	NA
Huaxiibacter	3.774	-1.309	1.090	3.290e-01
Chromobacterium	7.322	-1.307	0.618	9.629e-02
Catellicoccus	0.837	-1.306	2.349	NA
Gallintestinimicrobium	0.837	-1.302	3.025	NA
Parasphingorhabdus	1.435	-1.300	1.351	4.099e-01
Thermoanaerobacterium	14.931	-1.297	1.091	4.176e-01
Thioalkalivibrio	1.217	-1.293	1.453	NA
Plastoroseomonas	0.822	-1.288	2.150	NA
Pseudorhodoplanes	0.962	-1.278	1.437	NA
Nitrolancea	1.489	-1.277	1.871	6.272e-01
Sphaerotilus	3.359	-1.269	1.139	3.600e-01
Tardiphaga	3.851	-1.266	0.892	2.439e-01
Paraprevotella	4.071	-1.261	1.406	4.826e-01
Rickettsia	0.876	-1.260	1.535	NA
Mycoplasma	1.308	-1.251	0.962	NA
Pelosinus	4.274	-1.247	1.025	3.241e-01
Aquariibacter	0.913	-1.242	1.482	NA
Laetiporus	0.825	-1.242	3.372	NA
Neisseria	439.766	-1.240	0.222	2.029e-06
Oryzibacter	0.794	-1.239	2.343	NA
Caenimonas	0.820	-1.234	1.509	NA
Halopseudomonas	3.486	-1.233	1.050	4.195e-01
Methylotenera	10.684	-1.233	0.759	2.785e-01
Roseibium	0.811	-1.232	1.807	NA
Komagataeibacter	1.616	-1.232	1.687	1.000e+00
Desemzia	16.346	-1.232	0.606	1.506e-01
Oceanicella	2.913	-1.226	1.044	3.313e-01
Aquitalea	1.173	-1.226	0.889	NA
Pelomonas	2109.665	-1.222	0.177	1.974e-09
Sparassis	5.100	-1.221	0.896	2.818e-01

Lactococcus	112.493	-1.220	0.460	4.143e-02
Amycolatopsis	5.663	-1.212	0.616	1.412e-01
Aspergillus	116.582	-1.212	0.390	1.384e-02
Fenollaria	0.794	-1.209	1.796	NA
Gayadomonas	2.348	-1.206	0.672	1.115e-01
Amorphotheca	7.049	-1.205	0.819	2.516e-01
Oribacterium	16.999	-1.200	0.698	2.050e-01
Glycomyces	0.831	-1.198	2.152	NA
Plantactinospora	0.899	-1.189	1.476	NA
Nitrosocosmicus	0.792	-1.188	2.164	NA
Sneathia	0.975	-1.185	2.714	NA
Rivularia	0.796	-1.183	2.165	NA
Klugiella	0.760	-1.182	3.051	NA
Zeimonas	0.839	-1.174	2.038	NA
Rhodocyclaceae_genus	4.038	-1.172	1.049	3.708e-01
Colletotrichum	43.728	-1.172	0.402	2.000e-02
Escherichia_phage_DTL_virus	0.756	-1.170	2.172	NA
Leptotrichia	40.411	-1.167	0.386	1.573e-02
Acidiferrimicrobium	0.990	-1.163	1.824	NA
Siphonobacter	1.123	-1.162	2.471	NA
Rufibacter	1.318	-1.157	1.826	NA
Chaetomium	2.688	-1.156	1.232	4.620e-01
Coriobacteriaceae_genus	0.743	-1.151	2.540	NA
Metasolibacillus	0.775	-1.150	1.452	NA
Phialemonium	0.746	-1.149	2.333	NA
Sporosarcina	3.898	-1.145	0.891	2.920e-01
Bacteroidales	0.732	-1.145	1.878	NA
Lacipirellula	1.709	-1.144	2.084	1.000e+00
Phaeobacter	1.043	-1.130	0.992	NA
Burkholderiaceae_genus	42.128	-1.130	0.394	2.226e-02
Endosymbiont	5.833	-1.121	0.698	2.032e-01
Mycetocola	0.892	-1.120	1.451	NA
Rhodospirillaceae_genus	1.528	-1.116	1.543	6.223e-01
Agilicoccus	70.829	-1.115	0.597	1.715e-01
Glutamicibacter	45.516	-1.114	0.530	1.121e-01
Taibaiella	8.236	-1.106	0.880	4.585e-01
Planctomycetes	0.709	-1.103	2.016	NA
Leptospira	69.642	-1.102	0.317	4.854e-03
Peredibacter	28.482	-1.100	0.673	2.318e-01
Hydrogenophaga	66.646	-1.100	0.443	5.699e-02
Mumia	0.814	-1.086	2.198	NA
Pseudactinotalea	0.706	-1.086	3.397	NA
Fusicatenibacter	1.465	-1.086	1.645	6.954e-01
Puniceibacterium	0.825	-1.085	1.882	NA
Crenobacter	1.097	-1.081	1.057	NA
Labedella	0.708	-1.080	2.329	NA
Acidipropionibacterium	30.097	-1.078	0.723	2.815e-01
Desulfogranum	0.927	-1.072	1.782	NA
Rouxiella	0.687	-1.063	2.026	NA
Defluviimonas	0.700	-1.063	1.791	NA
Aestuariivirga	0.693	-1.061	2.529	NA
Richelia	0.740	-1.060	1.920	NA
Auritidibacter	1.372	-1.060	2.940	6.906e-01
Halomicroarcula	0.764	-1.059	1.192	NA
Frischella	1.199	-1.059	1.061	NA
Acidobacteria	2.858	-1.057	1.071	4.097e-01
Asinibacterium	2.381	-1.052	1.091	4.004e-01
Filobasidium	15.320	-1.050	0.585	2.045e-01
Dongia	0.686	-1.041	2.032	NA

Aestuariimicrobium	3.255	-1.040	1.179	1.000e+00
Aquimonas	0.678	-1.040	2.316	NA
Dactylosporangium	0.676	-1.035	3.397	NA
Corynebacterium	2249.564	-1.029	0.297	4.894e-03
Gluconacetobacter	6.748	-1.024	0.689	2.395e-01
Shewanella	593.636	-1.022	0.143	2.673e-10
Phytohabitans	0.667	-1.018	3.062	NA
Urbifossiella	0.741	-1.018	2.517	NA
Staphylococcus	2266.839	-1.016	0.352	2.289e-02
Fonticella	0.702	-1.015	2.769	NA
Methylobacterium	5697.290	-1.014	0.191	5.928e-06
Neorhizobium	3.954	-1.011	1.153	4.826e-01
Iamia	4.767	-1.011	1.292	6.423e-01
Betaproteobacterium_AAP65	0.934	-1.010	1.282	NA
Proteus_phage_VB_PmiS-Isfahan_virus	1.743	-1.010	0.673	1.393e-01
Methyloglobulus	0.746	-1.008	1.585	NA
Oligoflexus	0.666	-1.008	3.397	NA
Fulvia	3.759	-1.004	0.833	3.206e-01
Firmicutes	1.281	-1.002	1.436	NA
Sclerotinia	1.417	-1.001	1.488	5.228e-01
Mesobacillus	1.096	-0.995	1.602	NA
Purpureocillium	0.658	-0.995	3.059	NA
Dankookia	0.758	-0.992	1.657	NA
Hammondia	7.312	-0.991	0.578	1.715e-01
Porphyromonas	64.376	-0.991	0.454	9.770e-02
Pedobacter	122.644	-0.984	0.307	9.111e-03
Blastomyces	2.516	-0.984	1.104	4.427e-01
Gardnerella	33.910	-0.983	0.722	3.312e-01
Rhodoplanes	8.916	-0.982	0.666	2.578e-01
Providencia	883.343	-0.978	0.162	1.320e-07
Mixta	11.107	-0.977	0.709	3.208e-01
Rhodopirellula	20.297	-0.971	0.503	1.396e-01
Aestuariibaculum	1.315	-0.970	1.337	NA
Mycosynbacter	1.754	-0.967	1.481	5.688e-01
Chromohalobacter	65.014	-0.967	0.240	6.218e-04
Simplicispira	1.925	-0.966	1.125	4.081e-01
Thermaerobacter	0.642	-0.966	2.535	NA
Morchella	0.641	-0.959	2.766	NA
Marinilactibacillus	3.912	-0.956	1.266	5.428e-01
Motilimonas	7.460	-0.955	0.646	2.688e-01
Labilithrix	4.216	-0.955	1.221	5.367e-01
Escherichia_phage_vB_EcoS_ESCO41_virus	1.030	-0.948	1.633	NA
Agreia	0.629	-0.944	2.320	NA
Diolcogaster_facetosa_bracovirus	12.306	-0.940	0.679	3.279e-01
Ruoffia	0.625	-0.933	2.162	NA
Slackia	0.631	-0.933	2.182	NA
Paraclostridium	10.370	-0.930	0.825	4.112e-01
Luteimonas	79.273	-0.926	0.622	2.819e-01
Sphingomonas-like	0.813	-0.916	1.420	NA
Allostreptomyces	0.611	-0.913	2.773	NA
Hoylesella	13.883	-0.912	0.650	2.991e-01
Metarhizium	16.962	-0.908	0.649	3.256e-01
Betaproteobacterium_AAP121	0.906	-0.907	1.271	NA
Faecalicatena	0.820	-0.906	1.771	NA
Verticillium	0.616	-0.902	1.882	NA
Pseudorivibacter	1.485	-0.900	1.620	6.420e-01
Brochothrix	1.522	-0.898	1.580	7.074e-01
Phototrophicus	0.608	-0.896	2.539	NA
Vagococcus	0.603	-0.892	2.764	NA

Propionicimonas	2.047	-0.889	1.261	5.380e-01
Parafrankia	1.838	-0.887	1.304	5.480e-01
Hypoxylon	1.453	-0.886	1.675	7.240e-01
Segetibacter	2.497	-0.885	1.445	5.429e-01
Lentibacillus	34.801	-0.885	0.313	2.350e-02
Pseudonocardiaceae_genus	1.243	-0.880	1.971	NA
Torulaspora	0.598	-0.880	3.071	NA
Segatella	9.943	-0.877	0.691	3.447e-01
Neokomagataea	0.593	-0.869	3.398	NA
Desertimonas	11.415	-0.866	1.091	5.850e-01
Colwellia	7.776	-0.862	0.623	3.043e-01
Steroidobacter	1.133	-0.859	1.559	NA
Cobetia	0.578	-0.857	1.573	NA
Spirosoma	11.388	-0.852	0.729	3.977e-01
Neurospora	1.235	-0.852	2.387	NA
Camelimonas	0.584	-0.851	2.532	NA
Duganella	59.734	-0.850	0.425	1.268e-01
Conchiformibius	7.052	-0.844	0.935	4.985e-01
Halobacteriovorax	2.262	-0.844	0.755	2.938e-01
Gregarina	1.006	-0.838	2.688	NA
Chitinophaga	7.848	-0.836	0.729	3.864e-01
Cercospora	12.008	-0.834	0.647	3.438e-01
Sorangium	0.574	-0.831	2.784	NA
Malikia	0.578	-0.831	2.346	NA
Pantoea	249.985	-0.828	0.201	5.054e-04
Neoactinobaculum	0.622	-0.825	2.507	NA
Rhabdonatronobacter	170.476	-0.821	0.277	1.624e-02
Reticulibacter	0.566	-0.814	3.398	NA
Catellatospora	0.854	-0.814	2.947	NA
Podospora	0.954	-0.811	0.973	NA
Desulfoscipio	0.565	-0.810	2.517	NA
Polynucleobacter	2.817	-0.810	1.100	6.288e-01
Maribellus	40.625	-0.806	0.406	1.261e-01
Ferrovum	0.569	-0.806	2.776	NA
Synechocystis	16.400	-0.800	0.782	4.786e-01
Mesonia	2.526	-0.797	0.840	6.056e-01
Moesziomyces	19.781	-0.794	1.064	6.223e-01
Croceicoccus	0.549	-0.792	1.774	NA
Varibaculum	3.474	-0.791	1.148	5.688e-01
Rickettsiella	1.563	-0.784	0.942	4.526e-01
Rhodocyclus	0.572	-0.777	1.763	NA
Skermanella	24.233	-0.776	0.683	4.270e-01
Enterococcus	585.175	-0.775	0.139	8.905e-07
Lelliottia	2.324	-0.775	0.763	3.637e-01
Flavihumibacter	2.570	-0.770	0.996	5.767e-01
Caulobacteraceae_genus	1.255	-0.767	2.223	NA
Tatumella	7.235	-0.765	0.720	5.349e-01
Veillonella	194.159	-0.765	0.387	1.320e-01
Debaryomyces	1.396	-0.764	2.090	7.285e-01
Lachnellula	3.803	-0.759	0.961	5.221e-01
Naumannella	5.114	-0.759	1.005	5.549e-01
Herbiconiux	5.773	-0.757	1.163	1.000e+00
Gryllotalpicola	4.898	-0.755	1.181	6.177e-01
Sulfuritalea	0.539	-0.754	3.398	NA
Haloferax	0.578	-0.752	2.516	NA
Planococcus	196.283	-0.750	0.220	5.162e-03
Pochonia	0.554	-0.750	1.754	NA
Gallaecimonas	1.455	-0.750	1.092	5.380e-01
Flavipsychrobacter	0.537	-0.747	2.774	NA

Truepera	3.557	-0.745	1.267	6.288e-01
Megasphaera	17.752	-0.742	0.790	5.357e-01
Tomitella	1.159	-0.741	1.605	NA
Peptococcus	0.533	-0.737	3.398	NA
Patulibacter	9.106	-0.734	0.890	5.543e-01
Salmonella	451.456	-0.733	0.178	4.304e-04
Parasutterella	0.614	-0.731	1.513	NA
Scandinavium	0.642	-0.730	0.734	NA
Crenalkalicoccus	0.840	-0.728	2.176	NA
Pseudofrankia	0.563	-0.727	2.525	NA
Thermohydrogenium	1.914	-0.720	2.130	8.396e-01
Mangrovicoccus	4.133	-0.718	1.050	6.420e-01
Andreesenia	0.720	-0.710	2.987	NA
Cryobacterium	1.151	-0.710	1.972	NA
Ilyomonas	0.580	-0.703	3.398	NA
Paeniroseomonas	0.513	-0.700	1.995	NA
Treponema	11.715	-0.699	0.613	4.033e-01
Streptococcus	1476.802	-0.696	0.339	1.145e-01
Starkeya	0.955	-0.695	1.407	NA
Fischerella	0.513	-0.695	3.081	NA
Sphingobacterium	129.321	-0.695	0.357	1.386e-01
Algibacter	5.117	-0.693	0.667	5.758e-01
Catonella	3.258	-0.692	1.098	5.963e-01
Xanthobacter	6.049	-0.687	0.775	4.920e-01
Wenjunlia	0.648	-0.687	2.475	NA
Microterricola	0.560	-0.681	2.516	NA
Virgibacillus	35.407	-0.676	0.320	9.469e-02
Beijerinckia	0.593	-0.676	2.333	NA
Thiobacillus	5.354	-0.675	0.912	5.636e-01
Isosphaera	0.503	-0.673	3.398	NA
Pichia	0.685	-0.672	1.567	NA
Abiotrophia	14.388	-0.669	0.724	5.235e-01
Natronorubrum	0.689	-0.665	1.716	NA
Cokeromyces	2.224	-0.660	0.616	3.288e-01
Butyricicoccus	0.662	-0.658	1.888	NA
Morganella	1.022	-0.654	0.725	NA
Subtercola	1.074	-0.653	2.051	NA
Vitreoscilla	1.803	-0.651	1.731	7.052e-01
Enterovirga	5.894	-0.648	1.123	6.531e-01
Microvirgula	1.737	-0.647	1.879	7.383e-01
Puccinia	4.914	-0.646	0.933	5.810e-01
Eremomyces	0.488	-0.642	2.533	NA
Paracraurococcus	0.696	-0.642	2.043	NA
Alkalispirochaeta	9160.805	-0.640	0.271	6.393e-02
Limobrevibacterium	0.486	-0.640	1.995	NA
Mongoliimonas	0.486	-0.638	3.398	NA
Moheibacter	0.573	-0.638	2.733	NA
Frigoriflavimonas	0.492	-0.638	2.332	NA
Planctopirus	0.483	-0.637	1.669	NA
Emiliania	0.907	-0.637	1.380	NA
Paenirhodobacter	23.041	-0.637	0.421	2.550e-01
Pseudooceanicola	63.502	-0.637	0.528	3.883e-01
Halomonas	148.408	-0.633	0.293	9.540e-02
Prauserella	0.485	-0.629	3.398	NA
Pirellula	0.574	-0.622	2.287	NA
Zavarzinella	0.480	-0.620	3.103	NA
Alicyclobacillus	0.659	-0.612	2.315	NA
Psychromicrobium	11.336	-0.611	0.371	2.155e-01
Thermosipho	4.351	-0.610	0.526	3.637e-01

Odoribacter	0.475	-0.609	2.785	NA
Acetobacterium	3.427	-0.608	1.136	6.444e-01
Frondihabitans	2.164	-0.607	1.442	7.034e-01
Chloroploca	0.474	-0.607	3.398	NA
Prolinoborus	2.487	-0.604	0.974	5.813e-01
Phenylobacterium	59.083	-0.603	0.411	2.819e-01
Calidifontibacter	0.577	-0.602	2.518	NA
Acetivibrio	1.633	-0.600	2.231	9.401e-01
Lindgomyces	0.470	-0.600	2.788	NA
Gluconobacter	9.302	-0.590	0.762	5.810e-01
Modestobacter	77.884	-0.590	0.317	1.547e-01
Nonomuraea	1.439	-0.587	1.360	6.649e-01
Pseudolabrys	0.513	-0.584	1.844	NA
Idiomarina	0.506	-0.583	3.064	NA
Parapedobacter	0.475	-0.576	1.559	NA
Jaminaea	0.480	-0.576	3.398	NA
Rhodococcus	464.902	-0.575	0.207	2.459e-02
Legionella	1332.136	-0.572	0.274	1.042e-01
Paraconexibacter	2.432	-0.572	1.310	6.997e-01
Scardovia	0.474	-0.570	3.398	NA
Weissella	5.780	-0.569	0.937	6.379e-01
Eimeria	1.027	-0.565	1.208	NA
Viridibacillus	0.463	-0.565	2.326	NA
Rothia	240.951	-0.563	0.297	1.438e-01
Frateuria	2.007	-0.562	0.919	5.756e-01
Pasteurellaceae_genus	2.823	-0.558	1.073	6.486e-01
Wigglesworthia	1.349	-0.552	1.001	NA
Elstera	0.451	-0.550	2.781	NA
Amnibacterium	7.258	-0.547	0.928	6.618e-01
Microbacterium	1220.119	-0.545	0.151	2.463e-03
Bacillus	2815.520	-0.545	0.086	9.730e-09
Bavariicoccus	65.404	-0.543	0.381	2.918e-01
Carnobacterium	19.889	-0.543	0.575	5.092e-01
Zasmidium	1.244	-0.541	1.023	NA
Pseudomonas	20853.250	-0.538	0.238	7.681e-02
Humisphaera	4.982	-0.533	1.530	1.000e+00
Chloroflexus	0.443	-0.532	2.798	NA
Adlercreutzia	0.443	-0.530	2.344	NA
Phytoactinopolyspora	0.463	-0.530	2.773	NA
Ilumatobacter	4.926	-0.527	1.264	1.000e+00
Baudoinia	14.011	-0.527	0.590	5.364e-01
Nocardiopsis	6.413	-0.522	1.142	7.240e-01
Micrococcaceae_genus	0.443	-0.521	2.134	NA
Parachlamydia	0.456	-0.519	3.398	NA
Thermosinus	0.439	-0.518	3.398	NA
Akanthomyces	0.437	-0.514	3.398	NA
Prosthecomicrobium	0.590	-0.510	2.248	NA
Pannonibacter	2.009	-0.505	1.592	7.623e-01
Sediminibacterium	2.811	-0.505	1.347	7.364e-01
Cardiobacterium	13.923	-0.504	0.671	6.190e-01
Nigerium	0.539	-0.502	2.750	NA
Sporomusaceae_genus	0.444	-0.500	1.475	NA
Tolypothrix	6.280	-0.497	0.964	6.946e-01
Rudaea	1.559	-0.496	0.881	5.767e-01
Pseudocercospora	7.879	-0.489	0.763	6.423e-01
Collibacillus	0.630	-0.488	3.036	NA
Bartonella	9.625	-0.483	0.666	6.272e-01
Chitinophagaceae_genus	0.534	-0.483	2.723	NA
Cryptococcus	9.052	-0.482	0.973	7.404e-01

Planctomyces	2.081	-0.481	1.231	7.074e-01
Xylanimonas	3.351	-0.481	1.144	7.074e-01
Ferruginibacter	1.535	-0.478	1.475	7.136e-01
Didymosphaeria	28.357	-0.477	0.697	6.423e-01
Malassezia	1277.950	-0.476	0.345	3.102e-01
Paracnuella	0.644	-0.475	1.462	NA
Flavobacterium	706.963	-0.472	0.176	2.853e-02
Niabella	0.539	-0.469	2.102	NA
Mesomycoplasma	0.567	-0.467	2.490	NA
Kinneretia	11.622	-0.466	0.607	5.962e-01
Anaerobiospirillum	0.687	-0.465	2.714	NA
Sphingobium	158.875	-0.463	0.226	1.101e-01
Moraxella	437.707	-0.461	0.288	2.270e-01
Actinokineospora	43.083	-0.461	0.621	6.177e-01
Trabulsiella	0.462	-0.461	0.724	NA
Moraxellaceae_genus	14.760	-0.456	0.662	6.405e-01
Devosia	86.884	-0.453	0.433	4.637e-01
Chryseomicrobium	0.671	-0.450	1.985	NA
Catenibacterium	1.044	-0.450	2.156	NA
Frigoribacterium	15.155	-0.450	0.744	6.930e-01
Chitinimonas	12.648	-0.448	0.809	7.154e-01
Asticcacaulis	114.652	-0.443	0.472	5.212e-01
Minicystis	1.842	-0.440	2.026	8.396e-01
Sphingosinithalassobacter	0.506	-0.438	1.834	NA
Loktanella	12.504	-0.438	0.326	3.169e-01
Planktothrix	6.905	-0.437	0.747	7.034e-01
Aurantiacibacter	6.751	-0.436	0.942	7.210e-01
Penicilliopsis	0.668	-0.433	2.713	NA
Cryphonectria	1.076	-0.429	1.555	NA
Pediococcus	0.807	-0.427	1.233	NA
Psychrobacter	113.554	-0.417	0.344	3.821e-01
Roseisolibacter	3.912	-0.415	1.280	7.704e-01
Caenispirillum	0.400	-0.415	3.398	NA
Synechococcus	1.106	-0.412	1.598	NA
Phaeoacremonium	3.214	-0.410	0.841	7.067e-01
Nocardioidaceae_genus	0.397	-0.408	3.398	NA
Rugamonas	22.555	-0.406	0.479	5.625e-01
Peribacillus	3.761	-0.404	1.080	7.364e-01
Aquihabitans	4.924	-0.400	1.252	7.887e-01
Zobellella	1.254	-0.399	1.924	NA
Propionibacterium_phage_PHL116M00_virus	0.473	-0.399	3.093	NA
Rhodanobacter	6.156	-0.396	0.752	7.717e-01
Helicobacter	0.471	-0.396	2.510	NA
Ehrlichia	3.296	-0.394	1.278	7.782e-01
Alishewanella	16.028	-0.392	0.775	7.285e-01
Pseudenterobacter	0.515	-0.390	0.661	NA
Arachnia	16.113	-0.389	0.653	6.905e-01
Ureibacillus	0.798	-0.388	1.691	NA
Tepidiphilus	60.638	-0.388	0.708	7.146e-01
Yinghuangia	0.547	-0.388	1.769	NA
Pajaroellobacter	0.393	-0.385	3.131	NA
Pseudobdellovibrio	0.385	-0.384	2.338	NA
Glaciecola	6.760	-0.383	0.445	5.416e-01
Nakamurella	30.160	-0.377	0.621	6.877e-01
Thermoleophilum	0.477	-0.376	2.729	NA
Haemophilus	217.469	-0.374	0.320	3.955e-01
Hirsutella	0.438	-0.374	3.398	NA
Phocaeicola	15.829	-0.370	0.731	7.364e-01
Tricharina	1.265	-0.370	1.435	NA

Flectobacillus	1.269	-0.369	1.517	NA
Drechmeria	0.521	-0.369	1.851	NA
Acidiluteibacter	0.466	-0.366	3.398	NA
Capnocytophaga	41.728	-0.366	0.359	4.706e-01
Kingella	8.778	-0.364	0.616	6.691e-01
Buchnera	70221.134	-0.363	0.214	1.954e-01
Serpentinimonas	0.382	-0.360	3.138	NA
Scleromatobacter	0.399	-0.359	3.111	NA
Sphaerisporangium	0.405	-0.354	3.128	NA
Inhella	1.432	-0.347	1.777	8.535e-01
Kickxella	0.434	-0.347	2.299	NA
Fretibacterium	3.170	-0.347	1.288	8.002e-01
Psychromonas	1.967	-0.344	0.789	6.288e-01
Arcticibacter	0.736	-0.342	1.658	NA
Hanamia	0.446	-0.340	3.398	NA
Mucilaginibacter	56.373	-0.337	0.354	5.012e-01
Thermogemmata	1.288	-0.331	2.249	NA
Gramella	1.113	-0.329	0.982	NA
Sporichthya	4.613	-0.329	1.224	9.307e-01
Lactiplantibacillus	3.865	-0.325	0.631	7.389e-01
Pisolithus	1.250	-0.325	1.113	NA
Piscicoccus	2.025	-0.317	1.735	8.617e-01
Cohnella	3.253	-0.312	0.828	7.074e-01
Aquibium	0.920	-0.311	1.978	NA
Salinicola	3.874	-0.310	0.666	6.751e-01
Thecamonas	1.245	-0.308	1.609	NA
Puia	26.661	-0.307	0.355	5.438e-01
Rhizorhapis	25.863	-0.307	0.582	7.169e-01
Lujinxingia	0.358	-0.305	2.007	NA
Desulfovibrio	71087.628	-0.305	0.261	3.970e-01
Chlorogloea	3.479	-0.303	1.538	8.453e-01
Allorhizobium	0.361	-0.300	3.144	NA
Petrotoga	0.426	-0.300	2.493	NA
Saccharimonas	0.553	-0.299	2.256	NA
Wielerella	1.083	-0.297	2.107	NA
Kushneria	83.057	-0.296	0.324	5.284e-01
Anaerostipes	0.356	-0.295	2.008	NA
Chthonobacter	0.402	-0.293	3.399	NA
Pinibacter	0.395	-0.290	2.800	NA
Variovorax	102.492	-0.287	0.290	4.861e-01
Flaviaesturariibacter	0.912	-0.287	2.497	NA
Perlucidibaca	1.576	-0.286	1.132	7.637e-01
Wenxinia	0.425	-0.283	2.150	NA
Tahibacter	0.454	-0.283	1.802	NA
Xanthomonas	75.563	-0.282	0.346	5.725e-01
Tenebrionibacter	0.381	-0.279	1.156	NA
Dermabacter	5.640	-0.278	1.044	8.303e-01
Solirubrobacter	24.375	-0.278	0.688	7.887e-01
Theileria	0.680	-0.276	1.389	NA
Entotheonella	3.765	-0.268	0.814	7.637e-01
Minwuia	0.824	-0.263	3.094	NA
Pseudopropionibacterium	6.216	-0.257	0.811	8.001e-01
Kordiimonas	0.472	-0.248	2.466	NA
Acidisoma	1.255	-0.246	1.736	NA
Armatimonas	0.426	-0.243	2.750	NA
Moorella	0.340	-0.242	3.399	NA
Type-F_symbiont_of_Plautia_stali	0.400	-0.241	3.399	NA
Rhodopseudomonas	31.634	-0.241	0.417	7.003e-01
Fodinicola	0.339	-0.240	3.153	NA

Snodgrassella	4.153	-0.237	1.400	8.763e-01
Tolumonas	0.515	-0.236	2.343	NA
Acanthamoeba	49.409	-0.235	0.798	8.501e-01
Carbonactinospora	0.476	-0.233	2.341	NA
Oscillatoria	1.378	-0.231	1.380	8.837e-01
Paraphaeosphaeria	0.868	-0.227	2.748	NA
Abditibacterium	5.942	-0.223	1.138	8.733e-01
Actinomycetospora	24.819	-0.222	0.665	8.303e-01
Capsulimonas	0.344	-0.221	2.303	NA
Bergeriella	1.836	-0.219	1.856	1.000e+00
Blattabacterium	0.399	-0.217	2.502	NA
Asanoa	0.412	-0.217	3.399	NA
Alistipes	0.914	-0.216	1.834	NA
Rehaibacterium	0.368	-0.214	2.300	NA
Allobacillus	31.886	-0.213	0.430	7.333e-01
Kluyvera	247.288	-0.212	0.221	4.977e-01
Erysipelotrichaceae_genus	0.333	-0.212	3.399	NA
Methylovorus	3.354	-0.210	0.996	8.319e-01
Telluria	20.994	-0.210	0.602	8.157e-01
Tessaracoccus	17.444	-0.209	0.757	8.606e-01
Verrucosispora	2.100	-0.206	1.140	8.303e-01
Salicibibacter	0.350	-0.206	3.399	NA
Coprothermobacter	0.362	-0.203	3.399	NA
Plautia	0.323	-0.202	1.909	NA
Proteiniclasticum	1.099	-0.199	1.007	NA
Amygdalobacter	0.372	-0.191	3.399	NA
Caldovatus	0.368	-0.190	2.118	NA
Methyloversatilis	103.042	-0.188	0.324	6.946e-01
Dysgonomonas	10.780	-0.186	0.533	8.234e-01
Xylophilus	6.954	-0.183	0.611	8.006e-01
Lysobacter	182.469	-0.182	0.250	6.182e-01
Labilibacter	4.141	-0.181	0.995	9.087e-01
Macellibacteroides	0.431	-0.180	2.463	NA
Geminicoccus	65.368	-0.178	0.275	6.463e-01
Lamprobacter	0.388	-0.178	1.555	NA
Fictibacillus	317.200	-0.176	0.405	7.675e-01
Galbitalea	2.897	-0.174	1.427	9.084e-01
Chryseobacterium	293.101	-0.173	0.242	6.272e-01
Helcococcus	0.826	-0.169	2.560	NA
Dickeya	1.888	-0.167	0.553	5.813e-01
Methylorubrum	226.898	-0.165	0.200	5.636e-01
Tetzosporium	0.333	-0.165	3.399	NA
Desulfotomaculum	0.503	-0.160	2.738	NA
Parvibaculum	1.055	-0.160	1.561	NA
Virgisporangium	0.313	-0.158	2.345	NA
Thiomonas	1.348	-0.157	1.473	NA
Atopomonas	30.225	-0.154	0.335	7.433e-01
Lentzea	21.126	-0.149	0.361	7.582e-01
Solibacillus	6.613	-0.148	0.772	8.665e-01
Chiayiivirga	0.821	-0.148	2.585	NA
Actinomyces	520.914	-0.148	0.281	7.198e-01
Limosilactobacillus	14.701	-0.147	0.517	8.396e-01
Falseniella	0.389	-0.143	2.758	NA
Uruburuella	0.311	-0.142	3.399	NA
Segnochrobactrum	0.379	-0.138	2.809	NA
Oxalobacteraceae_genus	2.465	-0.138	1.041	1.000e+00
Protochlamydia	0.307	-0.134	3.399	NA
Desulfuromonas	2.026	-0.129	1.690	9.526e-01
Anaerobacillus	0.305	-0.127	3.399	NA

Campylobacter	38.374	-0.124	0.345	8.114e-01
Cronobacter_phage_vB_CsaM_GAP32_virus	0.325	-0.123	3.139	NA
Qingrenia	0.304	-0.120	3.399	NA
Sodalis-like	0.331	-0.120	1.180	NA
Halalkalibacter	112.475	-0.119	0.357	8.300e-01
Parachlamydiaceae_genus	0.346	-0.118	3.399	NA
Paenibacillus	158.355	-0.112	0.217	7.240e-01
Nostoc	288.872	-0.112	0.233	7.429e-01
Actirhodobacter	0.376	-0.109	3.133	NA
Mangrovibacter	0.437	-0.108	0.798	NA
Robbsia	1.088	-0.104	1.511	NA
Stutzerimonas	32.547	-0.102	0.521	9.087e-01
Hyphomicrobium	34.815	-0.101	0.349	8.507e-01
Dysosmobacter	0.526	-0.093	2.508	NA
Enhydrobacter	51.501	-0.090	0.335	8.617e-01
Agathobacter	7.239	-0.090	1.191	9.688e-01
Anaerotruncus	0.786	-0.089	1.039	NA
Thermincola	0.633	-0.089	2.755	NA
Noviherbaspirillum	23.982	-0.085	0.805	9.673e-01
Niveispirillum	1.564	-0.083	1.715	1.000e+00
Blautia	17.234	-0.077	0.648	9.516e-01
Ancylomarina	7.570	-0.076	0.668	9.197e-01
Lacticaseibacillus	6.204	-0.073	0.791	9.516e-01
Hartmannibacter	0.288	-0.068	3.399	NA
Candidate	14.431	-0.068	0.424	9.339e-01
Aequorivita	205.552	-0.068	0.257	8.654e-01
Actinotalea	15.791	-0.064	0.824	9.821e-01
Planococcaceae_genus	0.288	-0.062	3.399	NA
Corticibacterium	0.417	-0.062	2.481	NA
Lipomyces	0.316	-0.062	2.839	NA
Peptostreptococcaceae_genus	4.076	-0.062	1.038	9.549e-01
Zimmermannella	7.604	-0.061	0.909	9.772e-01
Evansella	0.869	-0.058	2.474	NA
Pinisolibacter	11.572	-0.056	0.344	7.167e-01
Gallionella	0.693	-0.053	1.194	NA
Chlamydia	0.283	-0.050	3.213	NA
Coriobacteriales	0.346	-0.048	3.076	NA
Faecalibacillus	0.308	-0.048	3.399	NA
Bowmanella	57.320	-0.045	0.386	9.549e-01
Roseicitreum	1.223	-0.044	1.432	NA
Pleomorpha	4.781	-0.039	1.086	1.000e+00
Macrococcus	4.781	-0.038	0.898	9.549e-01
Cyberlindnera	2.022	-0.036	1.449	1.000e+00
Thiothrix	1.073	-0.034	2.565	NA
Microcella	4.645	-0.030	1.005	9.777e-01
Aminobacter	1.557	-0.030	1.103	1.000e+00
Pseudoalteromonas	365.269	-0.027	0.240	9.645e-01
Xanthomarina	0.968	-0.025	1.721	NA
Lederbergia	0.311	-0.023	2.808	NA
Actinomadura	901.640	-0.022	0.176	9.549e-01
Cyclobacterium	9.163	-0.021	0.441	8.234e-01
Grimontella	3.770	-0.021	0.868	9.686e-01
Leucothrix	10.440	-0.017	0.565	9.721e-01
Pontibacillus	7.454	-0.017	1.493	1.000e+00
Streptomyces	1952.590	-0.016	0.173	9.721e-01
Stenoxybacter	0.289	-0.016	3.399	NA
Eubacterium	18.755	-0.013	0.475	1.000e+00
Sulfuriferula	0.273	-0.012	3.399	NA
Kaistella	17.043	-0.012	0.567	1.000e+00

Gamma	2.482	-0.010	1.278	1.000e+00
Actinoalloteichus	0.568	-0.010	1.624	NA
Aliicoccus	0.581	-0.009	2.488	NA
Proteiniphilum	0.270	-0.005	3.399	NA
Cryptosporangium	2.818	-0.004	1.525	1.000e+00
Mikella	0.000	0.000	0.000	NA
Ishikawaella	0.000	0.000	0.000	NA
Hafniaceae_genus	0.000	0.000	0.000	NA
Shigella_phage_SfIV_virus	0.000	0.000	0.000	NA
Escherichia_phage_500465-1_virus	0.000	0.000	0.000	NA
Enterobacteria_phage_DE3_virus	0.000	0.000	0.000	NA
Enterobacteria_phage_P7_virus	0.000	0.000	0.000	NA
Escherichia_phage_RCS47_virus	0.000	0.000	0.000	NA
Lagierella	0.000	0.000	0.000	NA
Rhabdobacter	0.000	0.000	0.000	NA
Kallipyga	0.000	0.000	0.000	NA
Escherichia_phage_500465-2_virus	0.000	0.000	0.000	NA
Escherichia_virus_Lambda_2G7b	0.000	0.000	0.000	NA
Escherichia_phage_TL-2011b_virus	0.000	0.000	0.000	NA
Escherichia_virus_Lambda_4A7	0.000	0.000	0.000	NA
Pusillibacter	0.000	0.000	0.000	NA
Escherichia_phage_Lambda_ev099_virus	0.000	0.000	0.000	NA
Klebsiella_phage_4_virus	0.000	0.000	0.000	NA
Stx2-converting_phage_1717_virus	0.000	0.000	0.000	NA
Cetobacterium	0.000	0.000	0.000	NA
Escherichia_phage_Cartapus_virus	0.000	0.000	0.000	NA
SsRNA_phage_SRR5466337_3_virus	0.000	0.000	0.000	NA
Escherichia_phage_Lambda_ev207_virus	0.000	0.000	0.000	NA
Escherichia_virus_Lambda_1H12	0.000	0.000	0.000	NA
Tropicibacter	0.000	0.000	0.000	NA
Stx2-converting_phage_Stx2a_WGPS2_virus	0.000	0.000	0.000	NA
Escherichia_phage_Lambda_ev243_virus	0.000	0.000	0.000	NA
Couchioplanes	0.000	0.000	0.000	NA
Escherichia_virus_Lambda_2H10	0.000	0.000	0.000	NA
Thermobrachium	0.000	0.000	0.000	NA
Escherichia_phage_D6_virus	0.000	0.000	0.000	NA
Enterobacteria_phage_Sf6_virus	0.000	0.000	0.000	NA
Gloeothece	0.000	0.000	0.000	NA
Escherichia_phage_520873_virus	0.000	0.000	0.000	NA
Viadribacter	0.000	0.000	0.000	NA
Oceanotoga	0.000	0.000	0.000	NA
Salmonella_phage_SJ46_virus	0.000	0.000	0.000	NA
Phaseolus_vulgaris_endornavirus	0.000	0.000	0.000	NA
Kaustia	0.000	0.000	0.000	NA
Calidithermus	19.574	0.005	0.635	1.000e+00
Arachidicoccus	0.350	0.009	3.095	NA
Agromyces	13.851	0.009	0.496	1.000e+00
Mameliella	2.131	0.009	1.323	1.000e+00
Tepidanaerobacter	0.405	0.012	3.399	NA
Saezia	1.565	0.014	0.986	1.000e+00
Phascolarctobacterium	0.264	0.019	3.399	NA
Fontimonas	0.265	0.020	3.221	NA
Franconibacter	0.683	0.022	1.462	NA
Cupriavidus	310.513	0.023	0.237	9.679e-01
Hephaestia	1.644	0.025	1.481	1.000e+00
Bifidobacterium	47.754	0.026	0.402	9.930e-01
Gemmataceae_genus	0.813	0.026	2.191	NA
Histoplasma	0.752	0.028	1.442	NA
Hydrocarboniphaga	1.904	0.032	1.395	1.000e+00

Gemmobacter	13.161	0.036	0.750	1.000e+00
Filamentous	0.701	0.037	2.101	NA
Hydrobacter	0.593	0.038	2.299	NA
Collimonas	7.742	0.042	0.811	9.673e-01
Vampirovibrio	0.259	0.042	3.399	NA
Marasmius	3.117	0.042	1.166	9.401e-01
Protaetiibacter	1.770	0.044	1.379	1.000e+00
Pedococcus	6.046	0.045	0.833	1.000e+00
Thermacetogenium	0.258	0.046	3.399	NA
Mitsuokella	0.263	0.048	3.399	NA
Gordonia	74.952	0.049	0.422	9.606e-01
Alsobacter	1.691	0.050	1.436	1.000e+00
Paenacidovorax	3.180	0.057	1.143	1.000e+00
Sporolactobacillus	110.272	0.061	0.189	8.319e-01
Falsiroseomonas	137.429	0.061	0.298	8.933e-01
Tuwongella	0.369	0.063	3.399	NA
Atlanticothrix	0.252	0.064	2.871	NA
Microvirga	80.646	0.065	0.361	9.087e-01
Mycobacterium	323.882	0.068	0.220	8.396e-01
Curvibacter	191.180	0.070	0.175	7.815e-01
Pandoraea	33.582	0.080	0.374	8.965e-01
Aliarcobacter	1.698	0.081	2.388	1.000e+00
Ligilactobacillus	12.484	0.087	0.738	9.434e-01
Lawsonibacter	0.311	0.089	2.562	NA
Acidobacteriaceae_genus	0.311	0.092	2.829	NA
Grimontia	0.335	0.092	0.909	NA
Allosphingosinicella	0.640	0.092	1.838	NA
Defluviicoccus	0.374	0.095	2.724	NA
Yoonia	0.398	0.097	3.114	NA
Sandaracinobacter	0.418	0.098	3.399	NA
Fimbriiglobus	2.293	0.098	1.465	1.000e+00
Quatrionicoccus	0.245	0.099	3.399	NA
Inquilinus	0.544	0.101	1.704	NA
Achromobacter	178.806	0.101	0.156	6.486e-01
Pseudoxanthomonas	75.899	0.102	0.352	8.497e-01
Thiohalocapsa	35.128	0.109	0.423	8.635e-01
Melittangium	0.241	0.114	3.245	NA
Rodentibacter	0.334	0.115	2.170	NA
Tistrella	0.240	0.118	3.399	NA
Sphingomonas	2004.426	0.118	0.152	5.813e-01
Bifidobacteriaceae_genus	8.416	0.119	1.073	9.671e-01
Neobacillus	44.891	0.121	0.287	7.637e-01
Zoogloeaceae_genus	0.238	0.122	3.399	NA
Caldicellulosiruptor	0.237	0.127	3.399	NA
Ktedonobacter	0.238	0.128	3.399	NA
Rhodophyticola	0.237	0.132	3.399	NA
Methylococcus	3.490	0.133	0.851	1.000e+00
Polaromonas	23.598	0.135	0.358	8.001e-01
Ottowia	13.988	0.136	0.565	8.725e-01
Arthroderma	0.586	0.139	1.382	NA
Leucobacter	21.363	0.140	0.469	8.418e-01
Oscillibacter	0.819	0.143	2.793	NA
Flaviflexus	0.920	0.146	1.924	NA
Acidiplasma	17.926	0.147	0.397	8.001e-01
Trujillella	0.289	0.148	2.841	NA
Aff.	0.263	0.148	3.399	NA
Pyricularia	0.300	0.152	2.823	NA
Microbacter	0.671	0.152	1.988	NA
Zymomonas	0.330	0.154	3.399	NA

Shimia	358.445	0.154	0.351	7.637e-01
Solihabitans	20.875	0.156	0.315	6.486e-01
Arthrobacter	566.114	0.157	0.213	6.065e-01
Provencibacterium	0.230	0.160	3.399	NA
Nanogingivalis	0.227	0.162	2.168	NA
Haloechinothrix	0.329	0.162	2.772	NA
Variibacter	0.250	0.168	3.399	NA
Runella	0.324	0.172	2.468	NA
Syntrophomonas	0.225	0.173	3.399	NA
Archangium	2.807	0.176	0.942	1.000e+00
Laspinema	0.879	0.182	2.172	NA
Parabacteroides	28.004	0.182	0.404	7.516e-01
Roseburia	27.841	0.185	0.309	6.794e-01
Photorhabdus	47.052	0.185	0.433	7.623e-01
Rhizobium	451.252	0.187	0.189	4.706e-01
Rhizorhabdus	68.045	0.188	0.346	7.074e-01
Hoaglandella	0.398	0.192	2.505	NA
Saccharomonospora	2.103	0.193	1.139	1.000e+00
Citreicoccus	0.220	0.195	3.400	NA
Aggregatilinea	0.220	0.196	3.400	NA
Xenophilus	37.016	0.197	0.385	7.169e-01
Paraflavitalea	0.310	0.203	3.400	NA
Ramlibacter	69.721	0.204	0.441	7.492e-01
Fusarium	142.957	0.206	0.671	8.396e-01
Ralstonia	14254.930	0.207	0.188	4.109e-01
Ewingella	0.845	0.213	2.761	NA
Pararhodobacter	5.193	0.214	0.930	8.893e-01
Azonexus	3.481	0.215	1.260	1.000e+00
Cutibacterium	314.810	0.215	0.334	6.486e-01
Lactovum	3.125	0.216	1.252	1.000e+00
Leuconostoc	27.068	0.218	0.379	6.946e-01
Kuraishia	0.224	0.222	3.400	NA
Mobiluncus	3.137	0.230	1.221	1.000e+00
Tissierella	290.882	0.235	0.222	4.319e-01
Longimicrobium	4.867	0.235	1.180	9.516e-01
Ruegeria	9.018	0.236	0.813	8.426e-01
Nocardioides	721.043	0.237	0.181	3.169e-01
Nesterenkonia	69.289	0.239	0.321	6.013e-01
Blastococcus	150.500	0.241	0.448	7.074e-01
Planomonospora	0.421	0.244	2.495	NA
Drancourtella	0.294	0.245	3.111	NA
Pyruvatibacter	1.367	0.246	0.902	1.000e+00
Cnuella	0.966	0.247	2.041	NA
Thyridium	3.802	0.251	1.227	9.936e-01
Miniimonas	0.208	0.255	3.234	NA
Dinghuibacter	0.335	0.255	3.400	NA
Neoarthrinium	0.839	0.257	1.717	NA
Sutterella	3.974	0.257	0.729	8.611e-01
Tepidimonas	35.654	0.258	0.571	7.556e-01
Mesorhizobium	79.330	0.259	0.228	3.912e-01
Solemya	6.351	0.260	0.794	8.332e-01
Jannaschia	2.279	0.264	0.503	8.346e-01
Falsirhodobacter	2.150	0.268	1.369	1.000e+00
Megamonas	3.586	0.269	1.194	7.914e-01
Ferribacterium	0.267	0.271	3.400	NA
Ectothiorhodospira	16.455	0.273	0.428	6.486e-01
Thermodesulfomicrobium	0.202	0.274	3.400	NA
Albitalea	0.203	0.274	3.266	NA
Heyndrickxia	3.581	0.274	1.632	8.426e-01

Thauera	36.207	0.276	0.281	4.729e-01
Immundisolibacter	1.280	0.276	1.454	NA
Kaistia	3.343	0.284	1.141	1.000e+00
Schumannella	0.649	0.285	2.264	NA
Robinsoniella	0.201	0.286	3.400	NA
Niallia	0.268	0.287	2.272	NA
Janibacter	177.517	0.295	0.274	4.207e-01
Phormidium	0.945	0.298	1.775	NA
Alteribacter	6.236	0.298	0.489	6.486e-01
Beggiatoa	32.646	0.298	0.287	4.405e-01
Tsuneonella	2.526	0.301	1.603	1.000e+00
Hansschlegelia	1.831	0.304	1.644	1.000e+00
Emericellopsis	3.169	0.306	1.746	1.000e+00
Scytonema	22.749	0.307	0.568	7.028e-01
Aeromonas	84.822	0.308	0.266	3.788e-01
Azorhizobium	2.897	0.308	1.079	9.738e-01
Alternaria	98.393	0.309	0.268	3.882e-01
Pontibrevibacter	0.195	0.309	3.400	NA
Thermococcus	14.111	0.313	0.483	6.431e-01
Paludifilum	42.859	0.314	0.265	3.692e-01
Fluoribacter	203.940	0.315	0.319	4.706e-01
Chryseosolibacter	0.194	0.315	3.400	NA
Propionispora	0.201	0.326	3.400	NA
Acidovorax	744.728	0.326	0.123	2.358e-02
Exiguobacterium	51.512	0.327	0.399	5.581e-01
Oleiphilus	1.386	0.328	0.862	9.087e-01
Parasaccharibacter	82.051	0.329	0.251	3.078e-01
Myxococcus	31.942	0.330	0.309	4.138e-01
Fervidobacterium	0.190	0.330	3.400	NA
Propioniferax	0.403	0.332	3.400	NA
Rubrivirga	1.178	0.336	2.381	NA
Dacryopinax	1.918	0.336	1.596	1.000e+00
Mycobacteriaceae_genus	41.727	0.336	0.267	3.206e-01
Marinithermofilum	29.006	0.336	0.312	4.105e-01
Barnesiella	9.605	0.337	0.441	5.813e-01
Paucibacter	116.341	0.337	0.272	3.402e-01
Fortiea	2.157	0.338	1.534	1.000e+00
Variimorphobacter	0.190	0.338	3.400	NA
Besnoitia	7.844	0.340	0.514	6.235e-01
Saccharomyces	1.469	0.342	1.872	1.000e+00
Pseudoduganella	3.809	0.345	0.798	7.856e-01
Cellulomonas	41.380	0.346	0.397	5.302e-01
Propionibacterium_phage_PHL117M01_virus	0.268	0.348	3.400	NA
Thermomonas	34.874	0.350	0.308	3.889e-01
Aromatoleum	0.470	0.351	2.564	NA
Methylophaga	0.781	0.352	1.400	NA
Nitrotoga	4.783	0.353	0.682	7.285e-01
Desulforhabdus	2.687	0.357	1.266	8.535e-01
Oerskovia	0.479	0.360	2.944	NA
Kwoniella	6.912	0.364	1.098	8.234e-01
Eleftheria	9.525	0.364	0.738	7.298e-01
Bergeyella	0.183	0.364	3.309	NA
Methylocaldum	0.395	0.368	1.977	NA
Gulosibacter	3.895	0.370	1.273	8.635e-01
Enteractinococcus	1.301	0.371	2.590	NA
Cereal_yellow_dwarf_virus	0.223	0.372	3.400	NA
Trichococcus	0.181	0.373	3.331	NA
Caldimonas	5.324	0.374	1.005	8.001e-01
Phycicoccus	66.900	0.375	0.300	3.370e-01

Rickettsiales	0.188	0.375	2.907	NA
Klenkia	7.551	0.376	0.856	7.584e-01
Gulbenkiania	0.803	0.378	2.056	NA
Maridesulfovibrio	0.179	0.380	3.400	NA
Olsenella	7.411	0.380	0.940	7.811e-01
Izhakiella	14.322	0.380	0.991	7.951e-01
Paenarthrobacter	0.392	0.382	2.757	NA
Limnohabitans	19.440	0.382	0.403	4.911e-01
Tabrizicola	3.196	0.382	0.972	6.891e-01
Jiangella	1.073	0.386	1.846	NA
Bhargavaea	9.142	0.393	0.708	6.963e-01
Chromatium	57.450	0.393	0.243	1.987e-01
Methylophilus	8.689	0.397	0.636	6.490e-01
Ciceribacter	3.850	0.401	0.950	8.212e-01
Macromonas	0.175	0.401	3.400	NA
Ectobacillus	119.344	0.403	0.414	4.753e-01
Oceaniovalibus	4.526	0.405	0.840	7.669e-01
Faunimonas	0.206	0.412	3.237	NA
Nocardia	68.412	0.416	0.263	2.092e-01
Parasphingopyxis	15.233	0.417	0.744	6.989e-01
Kytococcus	23.988	0.421	0.576	6.056e-01
Cystobacter	1.340	0.422	1.828	NA
Pelistega	0.345	0.424	3.399	NA
Schizosaccharomyces	3.316	0.425	1.331	8.489e-01
Weeksella	0.627	0.426	3.399	NA
Citricoccus	29.626	0.427	0.587	6.027e-01
Methylopila	6.130	0.428	0.992	7.782e-01
Chloroflexia	0.169	0.430	3.400	NA
Nannizzia	1.520	0.430	1.487	1.000e+00
Diaporthe	5.499	0.433	1.281	8.364e-01
Type-C_symbiont_of_Plautia_stali	0.212	0.435	3.199	NA
Thermobacillus	0.214	0.443	3.400	NA
Toxoplasma	250.863	0.446	0.172	2.466e-02
Phaeovulum	30.793	0.448	0.367	3.496e-01
Aphanothece	13.799	0.448	0.503	5.194e-01
Pedomonas	2.739	0.450	1.222	7.465e-01
Dubosiella	0.165	0.450	3.362	NA
Flavonifractor	0.231	0.453	3.400	NA
Herminiimonas	1.197	0.455	1.550	NA
Coccidioides	0.857	0.456	1.412	NA
Streptosporangium	49.775	0.460	0.276	1.781e-01
Blochmannia	13.304	0.462	0.813	6.950e-01
Croceibacterium	1.652	0.464	1.447	1.000e+00
Aeromicrobium	36.608	0.468	0.406	3.828e-01
Brenneria	3.075	0.472	0.494	5.559e-01
Penicillium	43.107	0.474	0.422	3.936e-01
Xylaria	0.604	0.477	2.003	NA
Terrabacter	109.045	0.480	0.273	1.549e-01
Knoellia	9.989	0.481	0.816	6.794e-01
Hassallia	0.246	0.486	3.400	NA
Pilimelia	1.515	0.491	1.633	1.000e+00
Alloiococcus	1.164	0.495	1.514	NA
Mobilicoccus	15.933	0.495	0.669	5.932e-01
Methanothermobacter	0.156	0.497	3.400	NA
Sphaerobacter	6.365	0.501	1.379	8.031e-01
Demequina	2.317	0.502	1.242	7.669e-01
Rhodocista	0.302	0.502	1.947	NA
Lasiodiplodia	27.457	0.503	0.412	3.447e-01
Pseudozyma	9.591	0.505	0.840	6.686e-01

Lonsdalea	0.189	0.506	1.059	NA
Comamonadaceae_genus	99.630	0.507	0.274	1.271e-01
Pedosphaera	0.186	0.509	3.400	NA
Alpha	67.476	0.512	0.330	2.155e-01
Coprococcus	3.166	0.513	1.584	9.022e-01
Superficieibacter	0.163	0.513	1.316	NA
Terracoccus	15.641	0.516	0.914	6.946e-01
Lactobacillus	101.572	0.516	0.229	5.607e-02
Ideonella	12.997	0.522	0.657	5.581e-01
Quadrisphaera	13.743	0.522	1.018	7.185e-01
Fontibacillus	0.151	0.523	3.400	NA
Salegentibacter	0.151	0.525	3.400	NA
Hoeflea	3.595	0.527	1.112	6.314e-01
Crocosphaera	13.832	0.530	0.468	3.821e-01
Thioflexithrix	10.033	0.534	0.529	4.397e-01
Methylobrevis	19.272	0.537	0.482	3.964e-01
Pseudescherichia	0.152	0.539	2.968	NA
Soleaferrea	0.148	0.540	3.400	NA
Promicromonospora	1.177	0.543	1.964	NA
Halteromyces	1.008	0.546	1.838	NA
Altererythrobacter	4.874	0.554	1.081	7.364e-01
Durotheca	0.146	0.555	3.400	NA
Aquincola	21.141	0.556	0.632	5.261e-01
Quisquiliibacterium	0.886	0.556	2.211	NA
Plasmodium	260.330	0.557	0.187	8.900e-03
Oscillochloris	0.821	0.559	3.392	NA
Sphingorhabdus	2.358	0.562	1.401	5.758e-01
Xylella	2.546	0.562	0.717	6.486e-01
Atopococcus	0.184	0.562	3.400	NA
Pirellulimonas	0.144	0.562	3.400	NA
Thermaurantiacus	0.224	0.563	3.400	NA
Dichomitus	29.347	0.570	0.803	6.110e-01
Solimonas	5.862	0.575	1.020	6.801e-01
Flexivirga	2.311	0.579	1.292	6.946e-01
Larkinella	0.768	0.582	1.868	NA
Salinarimonas	4.201	0.584	0.439	3.078e-01
Deinococcus	166.493	0.586	0.172	2.115e-03
Cronobacter	1.421	0.591	1.100	9.176e-01
Thioclava	0.294	0.599	2.720	NA
Neoroseomonas	0.161	0.599	3.400	NA
Fredinandcohnia	6.991	0.600	0.710	5.367e-01
Neglectibacter	0.247	0.604	1.582	NA
Cereibacter	5.029	0.607	0.919	6.486e-01
Salipiger	55.657	0.609	0.340	1.391e-01
Leclercia	58.127	0.610	0.317	1.101e-01
Ancylobacter	5.043	0.611	1.106	7.104e-01
Fastidiosipila	0.389	0.612	3.400	NA
Haliea	0.570	0.613	1.602	NA
Pseudophaeobacter	4.670	0.614	0.896	4.977e-01
Brooklawnia	0.606	0.614	2.525	NA
Azohydromonas	15.191	0.616	0.562	4.033e-01
Filifactor	2.916	0.617	1.163	7.669e-01
Marinilabiliaceae_genus	2.283	0.618	0.915	5.559e-01
Pyxidicoccus	1.671	0.618	1.330	8.893e-01
Geodermatophilus	37.591	0.619	0.646	4.753e-01
Alcanivorax	267.141	0.619	0.263	4.388e-02
Chthoniobacter	2.198	0.628	1.703	5.905e-01
Aphanizomenon	21.520	0.634	0.307	8.329e-02
Thermoanaerobacter	0.645	0.639	3.057	NA

Dongshaea	0.131	0.639	3.400	NA
Allofustis	0.129	0.641	3.400	NA
Atlantibacter	14.246	0.642	0.312	8.931e-02
Rhodovibrio	0.526	0.644	2.356	NA
Stenotrophomonas	1057.148	0.644	0.112	3.521e-08
Alkalihalophilus	0.129	0.646	3.400	NA
Arcticiflavibacter	197.563	0.648	0.374	1.557e-01
Komarekiella	0.170	0.649	3.400	NA
Mycoplana	0.174	0.650	3.400	NA
Roseateles	324.440	0.653	0.191	1.963e-03
Flagellatimonas	0.126	0.659	3.400	NA
UNVERIFIED_ORG:	13.458	0.659	0.563	3.727e-01
Jeotgalicoccus	20.422	0.664	0.436	2.205e-01
Microlunatus	62.692	0.669	0.477	2.689e-01
Halorubrum	12.131	0.673	0.498	2.812e-01
Siccibacter	0.129	0.674	2.706	NA
Flintibacter	0.137	0.677	3.400	NA
Isoptericola	31.305	0.678	0.273	3.238e-02
Elioraea	0.452	0.682	2.360	NA
Rhizobiales	18.512	0.693	0.533	3.138e-01
Salinisphaera	0.252	0.698	1.077	NA
Flavimobilis	2.525	0.702	1.244	7.549e-01
Acuticoccus	38.672	0.708	0.289	3.397e-02
Neptunicoccus	0.118	0.709	3.400	NA
Winogradskyella	5.235	0.710	0.448	1.716e-01
Micropruina	6.588	0.711	1.132	6.486e-01
Roseicella	3.466	0.716	1.112	6.853e-01
Latilactobacillus	2.108	0.717	1.528	8.635e-01
Methylosarcina	17.576	0.719	0.668	4.112e-01
Kockovaella	3.506	0.722	1.188	7.004e-01
Sabulicella	0.126	0.730	3.401	NA
Anoxybacillus	29.382	0.732	0.749	4.658e-01
Porphyrobacter	4.254	0.734	0.730	4.585e-01
Sphingosinicella	7.978	0.748	0.908	5.408e-01
Dechloromonas	6.203	0.749	0.796	4.786e-01
Xinfangfangia	1.289	0.753	1.661	NA
Bosea	160.841	0.753	0.209	1.062e-03
Congregibacter	0.755	0.755	1.150	NA
Humibacter	0.118	0.756	3.401	NA
Ornithinicoccus	0.159	0.757	3.251	NA
Plasticicumulans	0.111	0.758	3.401	NA
Prosthecobacter	3.876	0.764	1.172	6.891e-01
BeAn_58058_virus	0.384	0.764	1.326	NA
Azoarcus	3.353	0.764	0.980	5.963e-01
Actinobaculum	5.253	0.766	0.921	5.508e-01
Propionibacterium_phage_PHL301M00_virus	0.111	0.768	3.401	NA
Krasilnikoviella	0.109	0.768	3.401	NA
Aliterella	5.985	0.769	1.198	6.455e-01
Brucella	13.265	0.769	0.503	2.135e-01
Carideicomes	0.567	0.770	1.316	NA
Tetrasphaera	10.317	0.771	0.775	4.495e-01
Renibacterium	7.693	0.772	0.917	5.388e-01
Streptobacillus	0.298	0.775	3.398	NA
Rhodospirillum	1.216	0.775	1.566	NA
Gilbertella	13763.122	0.776	0.261	7.918e-03
Parvularcula	2.074	0.777	1.663	8.122e-01
Psychrosphaera	4.364	0.779	0.734	4.526e-01
Plectonema	0.287	0.779	3.401	NA
Frigidibacter	67.900	0.780	0.321	3.545e-02

Mariluticola	0.153	0.782	3.401	NA
Endocarpon	5.929	0.783	0.771	4.516e-01
Propionibacterium_phage_PHL041M10_virus	0.119	0.783	3.401	NA
Tamlana	6.081	0.786	0.529	2.215e-01
Zymoseptoria	45.638	0.786	0.475	1.718e-01
Paludicola	0.106	0.788	3.401	NA
Pseudoclavibacter	10.937	0.789	0.711	3.894e-01
Barrientosiimonas	8.157	0.791	1.215	6.420e-01
Baekduia	2.199	0.792	1.282	5.688e-01
Oryzihumus	0.119	0.794	3.401	NA
Sinisalibacter	0.394	0.794	2.272	NA
Pigmentiphaga	5.865	0.799	0.861	4.826e-01
Erythrobacter	135.550	0.802	0.254	4.763e-03
Linderina	1.155	0.805	1.251	NA
Mediannikoviicoccus	0.103	0.811	3.401	NA
Microthrix	0.483	0.813	3.396	NA
Roseibaca	0.208	0.815	2.880	NA
Deferrisoma	0.101	0.821	3.401	NA
Actinoplanes	35.115	0.822	0.420	1.011e-01
Rhodoferax	44.739	0.823	0.365	5.365e-02
Dyadobacter	16.940	0.828	0.540	2.069e-01
Pyrococcus	12.784	0.829	0.668	3.235e-01
Caballeronia	39.742	0.830	0.257	3.465e-03
Yimella	7.431	0.834	0.847	4.522e-01
Tetragenococcus	7.371	0.837	0.983	5.309e-01
Anaerotardibacter	0.125	0.843	2.623	NA
Paracoccus	735.536	0.844	0.180	8.826e-06
Paeniglutamicibacter	17.218	0.847	0.433	1.042e-01
Burkholderiales	19.138	0.847	0.492	1.601e-01
Winslowiella	0.287	0.849	3.401	NA
Garicola	1.949	0.853	1.717	7.198e-01
Arenimonas	1.067	0.855	1.638	NA
Moranella	0.333	0.856	2.985	NA
Rhodoligotrophos	1.096	0.857	1.928	NA
Lentihominibacter	0.458	0.861	2.312	NA
Brachymonas	13.279	0.862	0.515	1.689e-01
Nioella	0.094	0.867	3.401	NA
Vallicoccus	2.925	0.869	1.473	6.486e-01
Propionibacterium	4137.128	0.870	0.262	2.446e-03
Arsenicicoccus	2.165	0.871	1.175	5.508e-01
Methylovulum	39.553	0.872	0.547	1.940e-01
Algoriphagus	22.246	0.883	0.378	4.198e-02
Neptuniibacter	0.092	0.884	3.401	NA
Pluralibacter	1.045	0.884	1.574	NA
Rhodomicrobium	7.805	0.884	0.534	1.740e-01
Mediterraneibacter	9.994	0.887	0.499	1.443e-01
Diplodia	4.466	0.889	0.965	4.706e-01
Brevibacterium	83.680	0.897	0.382	4.143e-02
Aliidongia	0.759	0.898	1.857	NA
Sulfitobacter	6.435	0.900	0.536	1.722e-01
Kribbella	6.342	0.908	0.657	2.643e-01
Curtobacterium	171.096	0.910	0.440	8.038e-02
Labilibaculum	7.087	0.914	0.569	1.925e-01
Argonema	0.623	0.914	2.516	NA
Bacteria	0.118	0.918	1.070	NA
Citreimonas	0.088	0.922	3.401	NA
Vibrio	744.207	0.928	0.293	3.974e-03
Aeribacillus	0.369	0.929	3.401	NA
Propionibacterium_phage_P100D_virus	0.085	0.931	3.401	NA

Bacterium	36.502	0.932	0.264	1.359e-03
Effusibacillus	0.123	0.934	3.401	NA
Youxingia	0.215	0.935	2.896	NA
Advenella	1.895	0.937	1.147	6.272e-01
Nitrobacter	5.995	0.942	0.762	3.208e-01
Oceanobacillus	36.670	0.946	0.321	8.313e-03
Agathobaculum	0.083	0.946	3.401	NA
Terrimonas	56.451	0.948	0.612	2.039e-01
Brachybacterium	121.244	0.952	0.468	8.371e-02
Robiginitalea	0.271	0.952	3.092	NA
Rectinema	0.081	0.958	3.401	NA
Oxalicibacterium	1.142	0.965	1.647	NA
Corallococcus	123.324	0.969	0.295	2.611e-03
Alkanindiges	7.099	0.975	0.865	3.821e-01
Marinifilum	38.998	0.977	0.287	1.658e-03
Paracaedibacter	1.572	0.977	1.883	7.887e-01
Paenisporosarcina	0.629	0.981	3.400	NA
Carboxylicivirga	0.110	0.989	3.401	NA
Thermorudis	0.331	0.991	3.206	NA
Glaciimonas	3.779	0.996	1.146	5.172e-01
Planobispora	0.083	1.001	3.401	NA
Betaproteobacteria	0.088	1.001	3.401	NA
Parageobacillus	1.709	1.003	1.653	5.261e-01
Methylomicrobium	16.552	1.010	0.652	2.055e-01
Aplosporella	8.752	1.010	0.897	3.822e-01
Streptacidiphilus	1.421	1.014	1.706	3.336e-01
Chloroflexales	0.074	1.015	3.401	NA
Salinicoccus	15.586	1.018	0.739	2.688e-01
Oceanibium	5.401	1.018	0.762	2.816e-01
Dolosigranulum	11.557	1.022	0.788	3.000e-01
Pseudidiomarina	0.129	1.028	2.098	NA
Holdemania	0.072	1.030	3.401	NA
Halochromatium	4.224	1.033	0.745	2.695e-01
Pelagivirga	2.684	1.037	0.722	2.815e-01
Cecembia	0.302	1.038	2.586	NA
Ruminococcaceae_genus	14.329	1.044	0.426	3.146e-02
Mammaliicoccus	9.464	1.044	0.817	3.179e-01
Actinosynnema	2.269	1.045	0.988	4.207e-01
Dissoconium	0.190	1.046	3.401	NA
Bryobacter	0.866	1.057	3.045	NA
Pyrenophora	5.376	1.060	1.015	4.126e-01
Alkalibacterium	1.571	1.061	1.761	7.582e-01
Nisaea	0.072	1.069	3.401	NA
Planifilum	0.365	1.071	3.401	NA
Roseitranquillus	0.932	1.087	2.330	NA
Yeguia	0.064	1.090	3.401	NA
Planctomonas	4.608	1.090	1.035	4.284e-01
Euzebya	0.588	1.092	1.470	NA
Methylocella	3.329	1.093	1.477	6.177e-01
Pseudorhodoferax	8.912	1.093	0.750	2.326e-01
Rhodoblastus	1.242	1.093	1.838	NA
Nitrospira	0.572	1.094	2.246	NA
Polymorphobacter	6.803	1.098	0.948	3.765e-01
Marmoricola	34.294	1.099	0.625	1.393e-01
Pseudaestuariivita	0.064	1.103	3.401	NA
Desnuesiella	0.296	1.106	3.401	NA
Janthinobacterium	149.394	1.111	0.261	5.456e-05
Dietzia	166.910	1.119	0.369	5.535e-03
Angustibacter	3.139	1.121	1.339	5.155e-01

Paraglaciecola	2.599	1.124	0.743	2.286e-01
Arenivirga	0.339	1.124	3.397	NA
Mus_musculus_mobilized_virus	23.218	1.128	0.362	4.596e-03
Acinetobacter	11818.492	1.130	0.131	1.796e-18
Saccharophagus	0.425	1.137	2.156	NA
Propionicicella	1.665	1.137	1.471	6.288e-01
Solirhodobacter	0.058	1.139	3.401	NA
Massilia	397.055	1.143	0.314	6.813e-04
Type-B_symbiont_of_Plautia_stali	0.070	1.143	2.719	NA
Phialophora	4.587	1.143	1.107	4.133e-01
Buttiauxella	1.130	1.145	1.148	NA
Berkiella	0.057	1.147	3.401	NA
Peptoclostridium	0.886	1.152	1.995	NA
Hymenobacter	123.295	1.164	0.368	3.598e-03
Primorskyibacter	0.613	1.170	1.094	NA
Pseudohongiella	0.062	1.171	3.401	NA
Arcobacter	3.620	1.172	0.948	3.268e-01
Chryseolinea	0.170	1.175	3.401	NA
Paraburkholderia	685.653	1.187	0.137	1.353e-18
Burkholderia	2610.408	1.188	0.121	6.774e-24
Schneideria	0.970	1.194	1.801	NA
Sedimentitalea	30.138	1.198	0.289	9.380e-05
Leifsonia	76.614	1.200	0.429	1.120e-02
Aridibaculum	0.190	1.201	3.401	NA
Methylibium	8.094	1.204	0.934	2.991e-01
Limnobaculum	0.794	1.220	1.278	NA
Frisingicoccus	0.052	1.223	3.401	NA
Motilibacter	2.793	1.223	1.632	6.272e-01
Miltoncostaea	4.350	1.223	1.556	5.155e-01
Ruania	2.191	1.226	1.279	4.825e-01
Micrococcus	532.483	1.227	0.293	6.212e-05
Pseudovibrio	0.453	1.230	2.454	NA
Mariprofundus	0.047	1.231	3.401	NA
Austwickia	2.277	1.237	1.327	5.428e-01
Cellulosimicrobium	4.141	1.240	1.464	7.603e-01
Geomonas	0.050	1.243	3.401	NA
Paramesorhizobium	0.152	1.244	3.401	NA
Romboutsia	3.047	1.248	0.839	2.536e-01
Roseovarius	20.641	1.249	0.394	3.768e-03
Undibacterium	178.266	1.250	0.373	1.897e-03
Human_endogenous_retrovirus	5.191	1.254	0.613	7.972e-02
Annandia	0.084	1.264	2.380	NA
Rhabdothermincola	8.157	1.264	0.985	2.954e-01
Liquorilactobacillus	0.992	1.266	1.905	NA
Geobacillus	32.342	1.269	0.727	1.393e-01
Oceanicola	2.846	1.271	1.215	4.102e-01
Erysipelothrix	0.043	1.273	3.401	NA
Acetanaerobacterium	0.863	1.274	1.888	NA
Arthromitus	0.148	1.276	3.401	NA
Catenuloplanes	0.057	1.276	3.401	NA
Gynurincola	0.054	1.276	3.401	NA
Sinorhizobium	5.189	1.277	0.923	2.536e-01
Hubei_permutotetra-like_virus	2.387	1.281	1.997	5.315e-01
Lignipirellula	0.509	1.285	3.043	NA
Papillibacter	0.041	1.287	3.401	NA
Yaniella	1.655	1.291	1.439	5.553e-01
Rubricoccus	0.152	1.306	3.401	NA
Pelagerythrobacter	0.197	1.312	3.398	NA
Teredinibacter	0.038	1.317	3.401	NA

Lampropedia	1.172	1.317	1.789	NA
Hyphobacterium	32.142	1.317	0.327	1.424e-04
Actinomarinicola	1.651	1.317	1.463	6.177e-01
Filomicrobium	2.369	1.321	1.245	3.891e-01
Branchiibius	0.731	1.327	2.713	NA
Agrobacterium	192.449	1.329	0.172	6.083e-15
Candida	5.852	1.331	0.719	1.197e-01
Sulfolobus	2.237	1.335	0.512	2.360e-02
Empedobacter	31.761	1.336	0.410	2.517e-03
Betaproteobacterium_JGI	8.316	1.337	0.473	1.001e-02
Epilithonimonas	41.936	1.337	0.399	1.644e-03
Pseudonocardia	81.981	1.344	0.431	3.962e-03
Shouchella	0.581	1.345	3.194	NA
Schlegelella	25.036	1.347	0.486	1.160e-02
Lachnospira	17.322	1.351	0.523	1.972e-02
Gemmiger	1.474	1.364	1.604	5.853e-01
Propionibacterium_phage_SKKY_virus	0.362	1.385	3.397	NA
Tenebrionicola	0.031	1.385	3.401	NA
Miniphocaeibacter	0.034	1.386	3.401	NA
Sphaerulina	10.884	1.391	0.778	1.246e-01
Hydrotalea	0.322	1.396	3.124	NA
Simonsiella	0.622	1.401	2.467	NA
Luteipulveratus	0.361	1.407	2.995	NA
Arboricoccus	0.363	1.410	2.637	NA
Methylomonas	1.727	1.414	0.673	8.711e-02
Baumannia	0.277	1.414	3.401	NA
Spleen_focus-forming_virus	3.983	1.416	0.527	1.557e-02
Abelson	3.843	1.419	0.535	1.560e-02
Acetobacter	16.394	1.425	0.363	1.993e-04
Nitrosomonas	5.354	1.431	0.914	1.845e-01
Hankyongella	1.915	1.441	2.236	6.575e-01
Granulicoccus	0.433	1.444	2.768	NA
Occultella	0.037	1.447	3.402	NA
Petrimonas	0.193	1.448	3.399	NA
Profftia	0.070	1.450	3.401	NA
Vibrionimonas	0.863	1.451	2.142	NA
Pleurocapsa	1.217	1.453	1.937	NA
Trypanosoma	18.124	1.456	0.667	5.441e-02
Dermacoccus	44.902	1.462	0.322	9.299e-06
Brevundimonas	1147.754	1.464	0.230	1.267e-10
Rhodobacteraceae_genus	44.452	1.478	0.534	1.105e-02
Calorimonas	0.022	1.491	3.402	NA
Thermopolyspora	0.034	1.491	3.402	NA
Amaricoccus	20.347	1.494	0.628	3.268e-02
Clostridiales	35.068	1.495	0.424	8.288e-04
Propionibacterium_phage_PAD20_virus	0.255	1.496	3.399	NA
Ezakiella	1.567	1.500	2.025	4.319e-01
Thalassobius	5.691	1.511	0.844	1.214e-01
Vescimonas	0.025	1.515	3.402	NA
Mitsuaria	119.372	1.530	0.270	1.307e-08
Parapusillimonas	0.200	1.539	3.399	NA
Ignavibacterium	0.501	1.542	3.393	NA
Ramularia	0.158	1.544	3.402	NA
Plesiocystis	0.041	1.544	3.402	NA
Kocuria	405.951	1.547	0.435	7.336e-04
Terribacillus	0.063	1.550	3.402	NA
SsRNA_phage_SRR5466369_2_virus	0.058	1.550	3.402	NA
Radiomyces	1.508	1.551	1.453	4.654e-01
Gilliamella	0.563	1.552	1.340	NA

Nosocomiicoccus	1.257	1.558	1.927	NA
Umbelopsis	0.027	1.558	3.402	NA
Sandaracinus	1.493	1.563	1.844	5.536e-01
Riesia	0.026	1.564	3.402	NA
Geomicrobium	0.127	1.567	3.399	NA
Centipeda	0.032	1.568	3.402	NA
Saccharothrix	13.589	1.571	0.616	1.930e-02
Buchananella	0.056	1.596	3.402	NA
Bathymodiolus	0.021	1.596	3.402	NA
Cucumibacter	0.022	1.596	3.402	NA
Falcatimonas	0.017	1.596	3.402	NA
Rhizobiaceae_genus	0.018	1.596	3.402	NA
Geoalkalibacter	0.121	1.600	3.402	NA
Pararobbsia	0.017	1.602	3.402	NA
Wallemia	78.999	1.607	1.203	2.691e-01
Kalmanozyma	5.083	1.615	0.932	1.320e-01
Phyllobacterium	39.527	1.625	0.599	1.268e-02
Mycoavidus	1.273	1.626	1.933	NA
Oceanitalea	1.478	1.630	1.761	5.089e-01
Propylenella	0.014	1.646	3.402	NA
Anaerobutyricum	0.119	1.657	3.400	NA
Rhodospirillales	0.991	1.663	1.918	NA
Lewinella	0.693	1.668	0.662	NA
Pseudodesulfovibrio	0.057	1.668	3.402	NA
Dokdonella	1.249	1.677	1.944	NA
Comamonas	377.215	1.683	0.175	1.578e-23
Zygosaccharomyces	0.127	1.690	3.401	NA
Methanothrix	0.009	1.695	3.402	NA
Usitatibacter	0.008	1.695	3.402	NA
Rubellimicrobium	30.281	1.696	0.598	9.320e-03
Dermatobacter	0.611	1.697	1.983	NA
Tachikawaea	0.009	1.697	3.402	NA
Chloroflexi	0.627	1.700	1.641	NA
Companilactobacillus	0.377	1.700	3.040	NA
Lichtheimia	16.959	1.704	0.396	3.026e-05
Paraflavisolibacter	0.061	1.709	3.402	NA
Neofamilia	0.005	1.709	3.402	NA
Botrytis	2.262	1.711	1.236	2.078e-01
Amnimonas	9.718	1.713	1.275	2.666e-01
Pseudocnuella	0.005	1.714	3.402	NA
Paracandidimonas	0.784	1.714	2.498	NA
Hyphomonas	1.185	1.734	1.928	NA
Ornithinimicrobium	139.539	1.735	0.505	1.059e-03
Cucurbitaria	0.669	1.736	1.792	NA
Candidatus	2.396	1.739	1.594	3.936e-01
Hydromonas	0.010	1.743	3.402	NA
Enterobacteria_phage_YYZ-2008_virus	0.009	1.743	3.402	NA
Enterobacteria_phage_f1_virus	0.008	1.743	3.402	NA
Photodesmus	0.007	1.743	3.402	NA
Doolittlea	0.005	1.743	3.402	NA
Betaproteobacterium_AAP99	0.003	1.743	3.402	NA
Delftia	511.917	1.747	0.118	9.198e-55
FBR_murine_osteosarcoma_virus	1.393	1.749	0.674	2.585e-02
Enterobacteria_phage_T7_virus	0.002	1.754	3.402	NA
Calidifontimicrobium	0.522	1.758	3.392	NA
Acaricomes	2.882	1.760	0.978	1.170e-01
Pelovirga	0.002	1.761	3.402	NA
Muribaculaceae_genus	0.949	1.772	1.477	NA
Goekera	0.329	1.788	3.355	NA

Idiomarinaceae_genus	0.023	1.795	3.402	NA
Aaosphaeria	0.407	1.796	2.674	NA
Roseomonas	144.562	1.801	0.417	2.227e-05
Fimbriimonas	2.579	1.809	1.288	3.206e-01
Anabaena	1.596	1.818	1.353	3.447e-01
Aquirhabdus	0.022	1.841	3.402	NA
Hyalangium	1.398	1.842	2.356	5.688e-01
Roseobacter	0.876	1.844	1.495	NA
Kineosporia	6.745	1.846	1.156	1.714e-01
Shinella	19.684	1.847	0.595	3.708e-03
Herbaspirillum	591.387	1.852	0.123	1.199e-57
Delta	4.266	1.854	0.652	8.152e-03
Polysphondylium	0.588	1.857	2.672	NA
Tepidicella	11.802	1.864	0.823	4.143e-02
Silanimonas	0.443	1.871	3.101	NA
Actinobacterium	1.502	1.873	1.842	4.395e-01
Microcoleus	16.615	1.878	0.606	3.974e-03
Proteobacteria	45.108	1.878	0.814	3.595e-02
Alterileibacterium	0.250	1.879	3.398	NA
Pseudaminobacter	1.415	1.885	1.732	2.706e-01
Agaricicola	0.028	1.888	3.402	NA
Schizophyllum	63.317	1.893	1.007	9.958e-02
Sinirhodobacter	15.801	1.919	0.329	6.142e-09
Pseudoglutamicibacter	1.363	1.921	1.494	3.402e-01
Endobacter	3.493	1.923	1.347	2.094e-01
Sinomonas	0.642	1.932	3.067	NA
Zafaria	0.027	1.933	3.402	NA
Endozoicomonas	0.368	1.933	1.430	NA
Coniophora	9.959	1.961	1.154	1.393e-01
Amniculibacterium	1.183	1.968	2.990	NA
Aggregicoccus	0.395	1.970	3.394	NA
Novosphingobium	579.919	1.983	0.300	8.900e-12
Uncultured	130.743	1.987	0.370	5.045e-08
Fonsecaea	17.304	1.988	0.645	3.649e-03
Sulfuricystis	0.204	1.990	3.399	NA
Anaerosphaera	0.551	1.997	2.479	NA
Type-E_symbiont_of_Plautia_stali	0.036	2.002	3.401	NA
PreXMRV-1_provirus_complete	4.785	2.008	0.518	2.090e-04
Westerdykella	3.310	2.028	0.993	6.422e-02
Gullanella	1.395	2.032	1.128	1.299e-01
Puteibacter	1.005	2.037	1.301	NA
Actinoallomurus	1.044	2.053	1.819	NA
Halovulum	1.008	2.067	1.762	NA
Cellvibrio	9.111	2.079	0.857	2.688e-02
Mycetohabitans	9.753	2.081	0.468	1.380e-05
Veillonellaceae_genus	0.948	2.082	2.299	NA
Rubrivivax	73.057	2.086	0.335	1.737e-10
Mixia	2.238	2.087	1.332	1.740e-01
Friedmanniella	3.135	2.089	1.319	1.692e-01
Formosimonas	0.082	2.093	3.401	NA
Georgenia	10.823	2.099	0.702	4.922e-03
Brettanomyces	5.223	2.100	1.687	2.947e-01
Metabacillus	4.195	2.109	1.209	1.294e-01
Suillus	38.317	2.109	1.117	9.629e-02
Neofusicoccum	58.401	2.112	0.534	1.005e-04
Sphingopyxis	66.886	2.116	0.406	1.426e-07
Embleya	1.575	2.124	1.606	2.055e-01
Stakelama	0.615	2.125	1.758	NA
Polymorphum	0.621	2.126	1.974	NA

harvey_murine_sarcoma_virus	0.947	2.154	0.455	NA
Pneumocystis	8.386	2.162	1.387	1.783e-01
Hominisplanchenecus	0.033	2.172	3.401	NA
Azospirillum	271.311	2.179	0.333	1.176e-11
Anaeromassilibacillus	0.075	2.183	3.400	NA
Coniosporium	2.486	2.183	1.623	2.506e-01
Zhihengliuella	1.833	2.186	1.682	2.778e-01
Murine_osteosarcoma_virus	2.353	2.203	0.712	3.962e-03
Desulfofundulus	0.440	2.206	3.372	NA
Pelobacter	1.250	2.209	1.898	NA
Salinimicrobium	20.796	2.230	0.485	5.522e-06
Serinicoccus	17.817	2.235	0.878	1.845e-02
Microsporum	6.726	2.238	1.188	9.505e-02
Letharia	140.804	2.247	0.658	9.629e-04
Aequitasia	117.988	2.254	1.187	9.366e-02
Acidaminococcus	0.185	2.259	3.398	NA
Cordyceps	0.869	2.260	1.953	NA
Rhizobacter	51.463	2.270	0.274	5.826e-18
Aquabacterium	1382.076	2.297	0.137	9.231e-73
Nodosilinea	25.845	2.307	0.865	1.231e-02
Fuscibacter	0.854	2.334	2.361	NA
Capillimicrobium	2.546	2.353	1.416	1.421e-01
Erythrobacteraceae_genus	1.717	2.356	1.422	1.438e-01
Salinispora	1.822	2.370	1.550	2.228e-01
Aceticella	0.332	2.371	3.394	NA
Ochrobactrum	63.445	2.386	0.274	4.480e-20
Abyssicoccus	5.009	2.397	1.266	9.232e-02
Limimaricola	3.153	2.410	1.524	1.925e-01
Penaeicola	1.825	2.421	0.966	2.977e-02
Paracidovorax	147.082	2.440	0.277	1.159e-20
Mucor	14.971	2.466	0.922	1.246e-02
Tsukamurella	6.889	2.469	1.412	1.646e-01
Pseudoroseomonas	11.878	2.480	0.620	8.720e-05
Paenalcaligenes	0.760	2.502	0.988	NA
Anatilimnocola	0.615	2.504	3.357	NA
Piscinibacter	118.721	2.507	0.331	1.735e-15
Belnapia	18.847	2.543	0.654	1.466e-04
Verticiella	0.467	2.552	2.909	NA
Mycolicibacterium	115.168	2.556	0.261	2.715e-25
Algibacillus	1.251	2.564	0.973	NA
Wickerhamomyces	2.767	2.569	1.616	2.094e-01
Alkalihalobacillus	4.260	2.582	0.606	1.941e-05
Pseudarthrobacter	55.707	2.601	0.461	8.020e-09
Meyerozyma	1.367	2.636	2.107	3.279e-01
Pontibacter	1.186	2.637	1.667	NA
Adhaeribacter	6.285	2.667	1.253	5.182e-02
Viridilinea	0.240	2.667	3.396	NA
Sandarakinorhabdus	1.376	2.679	2.118	2.728e-01
Niastella	47.268	2.684	1.379	8.196e-02
Herpetosiphon	1.350	2.706	1.817	NA
Gellertiella	2.945	2.737	1.700	1.438e-01
Wickerhamiella	9.411	2.739	0.750	3.150e-04
Tepidiforma	3.376	2.745	1.571	1.101e-01
Spiribacter	38.734	2.745	0.395	4.092e-13
Dothidotthia	2.037	2.748	1.298	5.099e-02
Holdemanella	0.771	2.756	2.077	NA
Acidithiobacillus	129.947	2.781	0.474	1.084e-09
Bordetella	34.897	2.789	0.476	1.114e-09
Auraticoccus	1.587	2.819	2.255	2.482e-01

Boeremia	20.842	2.856	0.900	2.140e-03
Leptothrix	258.760	2.859	0.252	3.275e-34
Desulfocarbo	2.982	2.870	0.973	4.531e-03
Methyloradius	0.301	2.899	2.910	NA
Rhizophagus	4.450	2.903	1.109	1.160e-02
Sagittula	3.462	2.904	1.336	4.351e-02
Caldilinea	1.616	2.906	1.893	1.735e-01
Beutenbergia	4.200	2.916	1.197	2.393e-02
Trichosporon	145.713	2.937	0.805	3.150e-04
Exophiala	38.352	2.937	0.623	1.693e-06
Millisia	1.115	2.941	2.238	NA
Pseudomicrostroma	29.079	2.952	0.919	1.850e-03
Kineococcus	53.880	2.971	0.713	3.080e-05
Pseudanabaena	0.202	2.971	3.397	NA
Picosynechococcus	8.815	2.974	0.729	6.318e-05
Tersicoccus	1.189	2.985	2.427	NA
Sphingomonadaceae_genus	2.729	3.012	1.553	6.704e-02
Alteromonas	44.223	3.016	0.470	1.575e-11
Seonamhaeicola	12.679	3.016	0.345	5.189e-20
Seohaeicola	2.294	3.032	1.386	3.965e-02
Falsochrobactrum	0.395	3.038	3.295	NA
Pseudosporangium	0.338	3.046	3.393	NA
Siccirubricoccus	2.442	3.057	1.465	4.592e-02
Guillardia	48.159	3.066	1.162	1.268e-02
Afifella	1.377	3.070	1.936	1.201e-01
Enterocloster	0.679	3.076	1.982	NA
Batrachochytrium	121.131	3.104	1.903	1.513e-01
Betaproteobacterium_AAP51	11.054	3.136	0.519	4.005e-10
Polyangium	64.134	3.147	0.549	2.684e-09
Trichophyton	4.702	3.149	1.458	4.369e-02
Parasegetibacter	0.335	3.149	3.393	NA
Limnobacter	140.336	3.150	0.607	9.544e-08
Mycena	39.124	3.208	0.873	2.848e-04
Murine_type_C_virus	17.101	3.211	0.464	3.645e-13
Yarrowia	13.249	3.234	0.791	4.245e-05
Protomyces	16.751	3.244	1.043	2.626e-03
Acidimicrobium	0.273	3.290	3.394	NA
Pseudochrobactrum	75.819	3.335	0.391	1.003e-19
Vulcaniibacterium	1.978	3.403	1.755	7.329e-02
Agrococcus	150.918	3.441	0.659	7.759e-08
Haematobacter	4.763	3.455	1.244	9.852e-03
Sugiyamaella	2.101	3.466	1.237	5.043e-03
Ustilaginoidea	1.049	3.471	1.858	NA
Labrys	177.824	3.547	0.411	3.314e-20
Paenimyroides	6.925	3.580	0.972	5.979e-04
Mycobacteroides	34.731	3.653	0.616	6.837e-10
Gamsiella	9.097	3.672	1.170	2.241e-03
Spizellomyces	92.702	3.735	1.226	3.446e-03
Rugosimonospora	0.807	3.744	3.146	NA
Kirsten_murine_sarcoma_virus	1.366	3.750	1.644	2.206e-02
Trematosphaeria	6.493	3.804	1.002	1.580e-04
Synchytrium	3.426	3.820	1.362	6.608e-03
Apibacter	4.693	3.828	1.008	1.351e-04
Emergencia	2.121	3.828	1.365	4.254e-03
Acaromyces	28.763	3.917	0.916	1.835e-05
Haematomicrobium	1.630	3.955	2.278	1.999e-01
Zychaea	9.011	4.159	1.346	2.626e-03
Rhodocyclales	0.940	4.230	2.300	NA
Beijerinckiaceae_genus	37.739	4.236	0.637	4.490e-12

Spirilliplanes	0.536	4.259	3.389	NA
Mitosporidium	133.751	4.335	1.433	3.928e-03
Planomicrobium	4.483	4.526	1.491	1.624e-02
Didymella	21.350	4.526	0.991	4.617e-06
Polychytrium	132.053	4.570	0.962	1.818e-06
Phaeosphaeria	3.618	4.650	1.421	1.152e-03
Leptosphaeria	79.063	4.657	0.773	5.707e-10
Saitoella	68.439	4.718	1.562	4.202e-03
Glycocaulis	61.217	4.790	1.097	1.527e-05
Lentilactobacillus	33.253	4.814	1.121	2.295e-05
Lobosporangium	154.598	5.214	1.224	3.122e-05
Parastagonospora	616.798	5.222	0.580	7.941e-21
Clavispora	11.624	5.259	1.419	1.028e-03
Macroventuria	247.054	5.455	0.669	4.010e-17
Mycotypha	40.609	5.599	1.150	1.955e-06
Melampsora	15.381	5.638	1.281	2.295e-05
Meira	102.335	5.674	1.022	3.536e-08
Nitriliruptoraceae_genus	156.786	5.769	0.831	2.294e-12
Ascochyta	1160.970	5.781	0.685	6.100e-18
Fimicolochytrium	46.223	5.905	1.001	4.498e-09
Cytobacillus	13.792	6.010	0.970	2.698e-10
Nitrosospira	14.651	6.210	1.441	6.617e-05
