# Supplementary figures and images for: Shotgun Metagenome Analysis of Two Schizaphis graminum Biotypes over Time With and Without Carried Cereal Yellow Dwarf Virus
Source: Insects. 2025 May 23;16(6):554. doi: 10.3390/insects16060554 (PMC12193481; doi:10.3390/insects16060554)

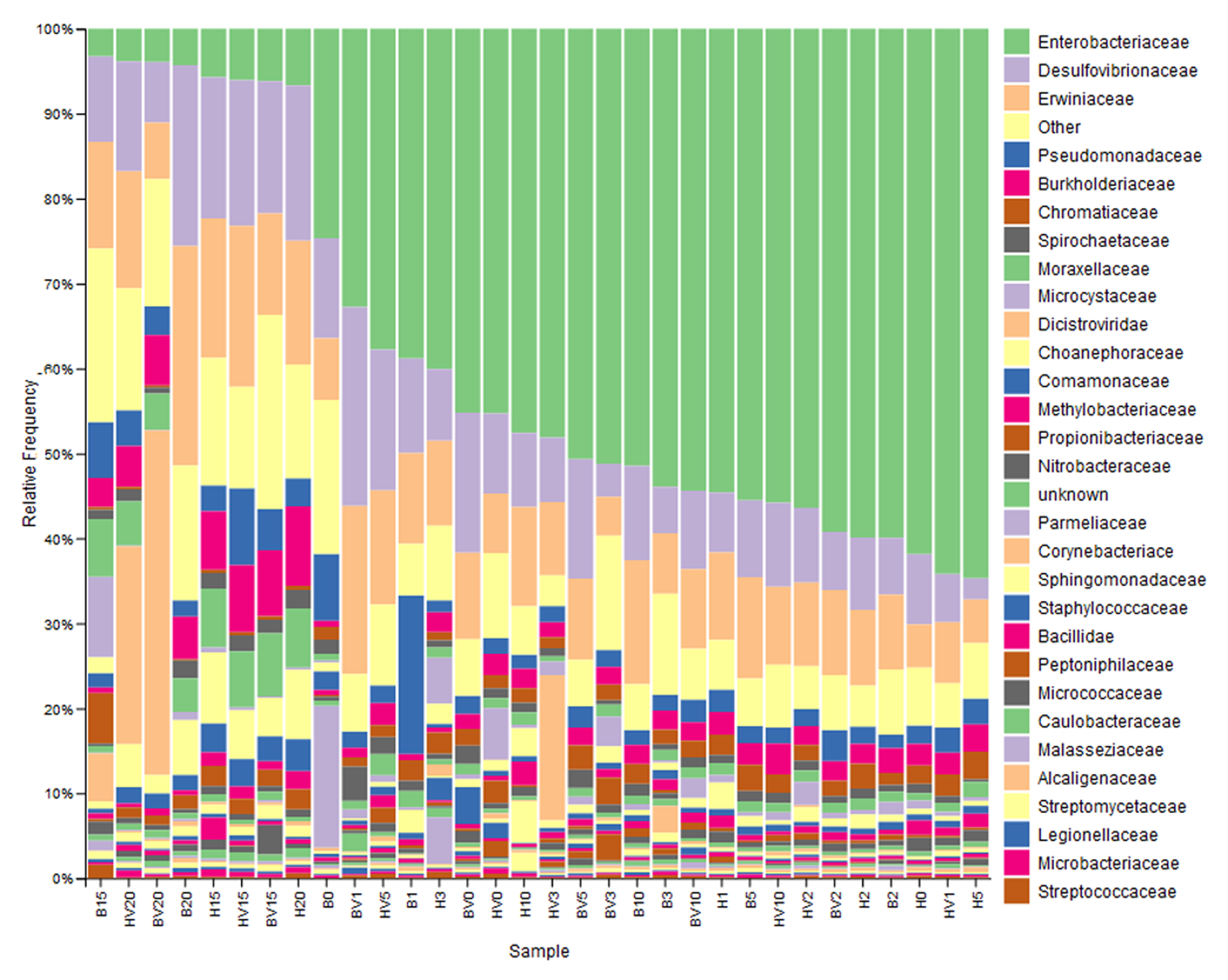

Supplement: Supplementary file 1 [file insects-16-00554-s001.zip › Figure S1-Family-byOrg.tif]

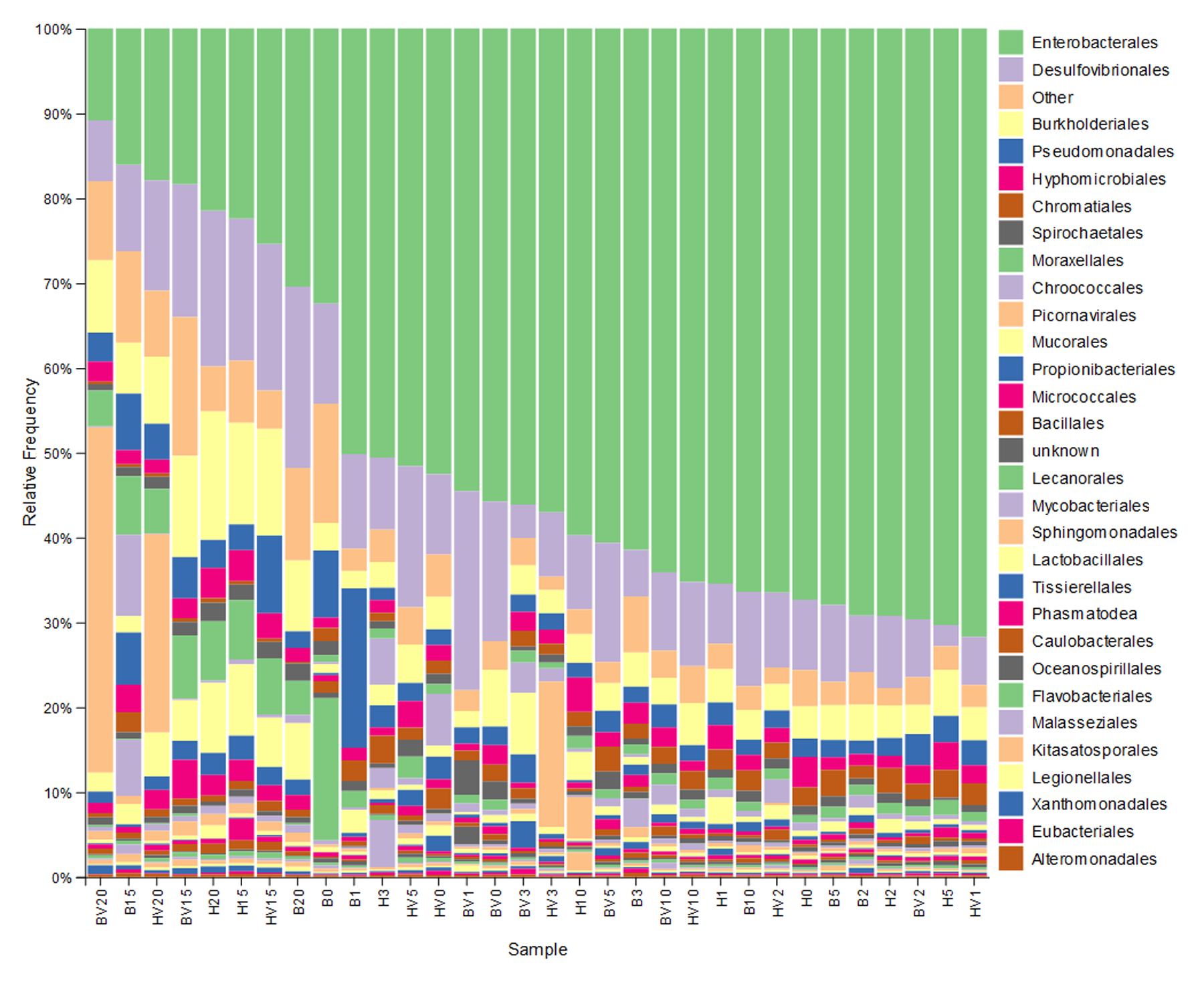

Supplement: Supplementary file 1 [file insects-16-00554-s001.zip › Figure S2-Order-byOrg.tif]

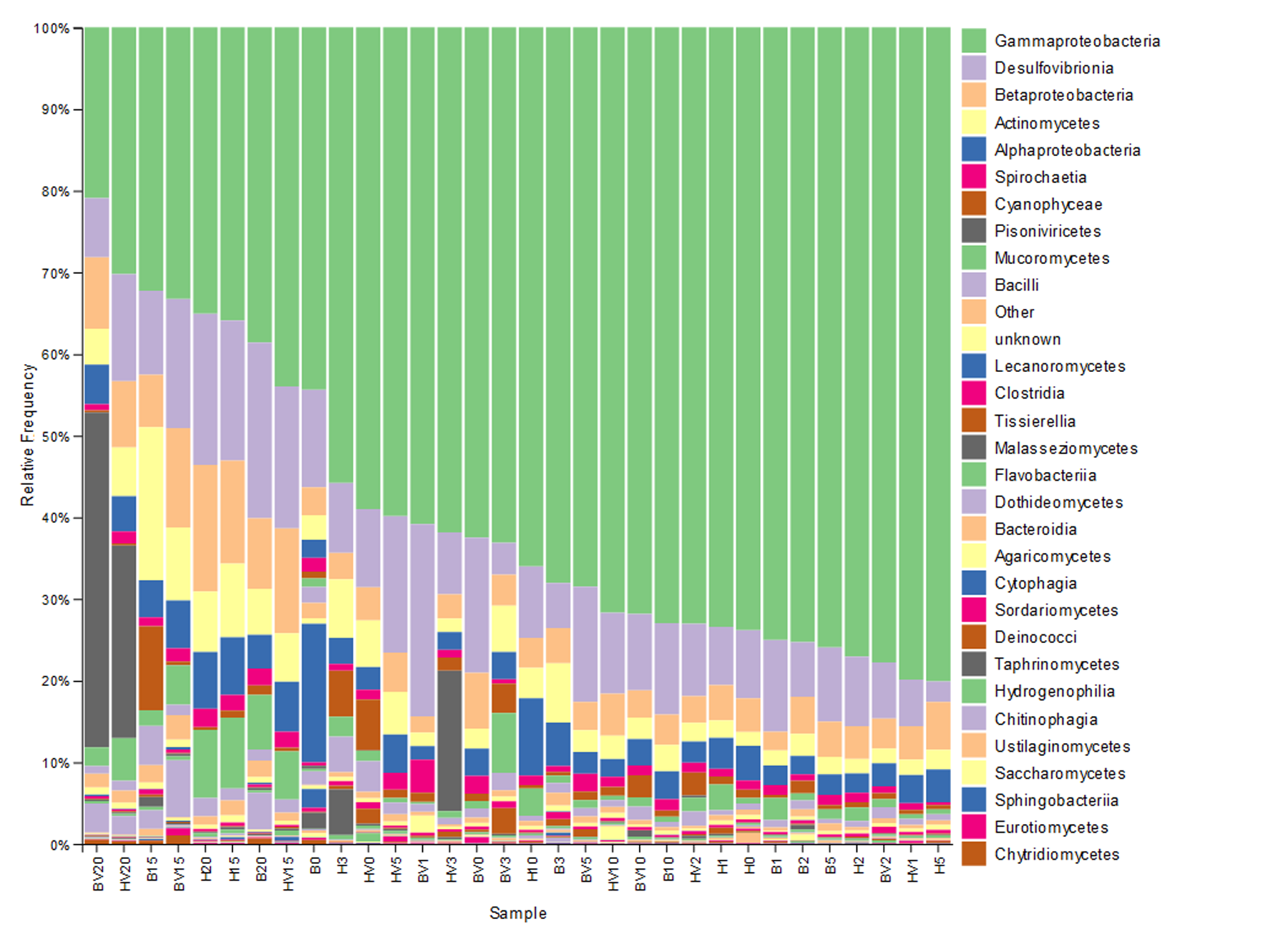

Supplement: Supplementary file 1 [file insects-16-00554-s001.zip › Figure S3-Class-byOrg.tif]

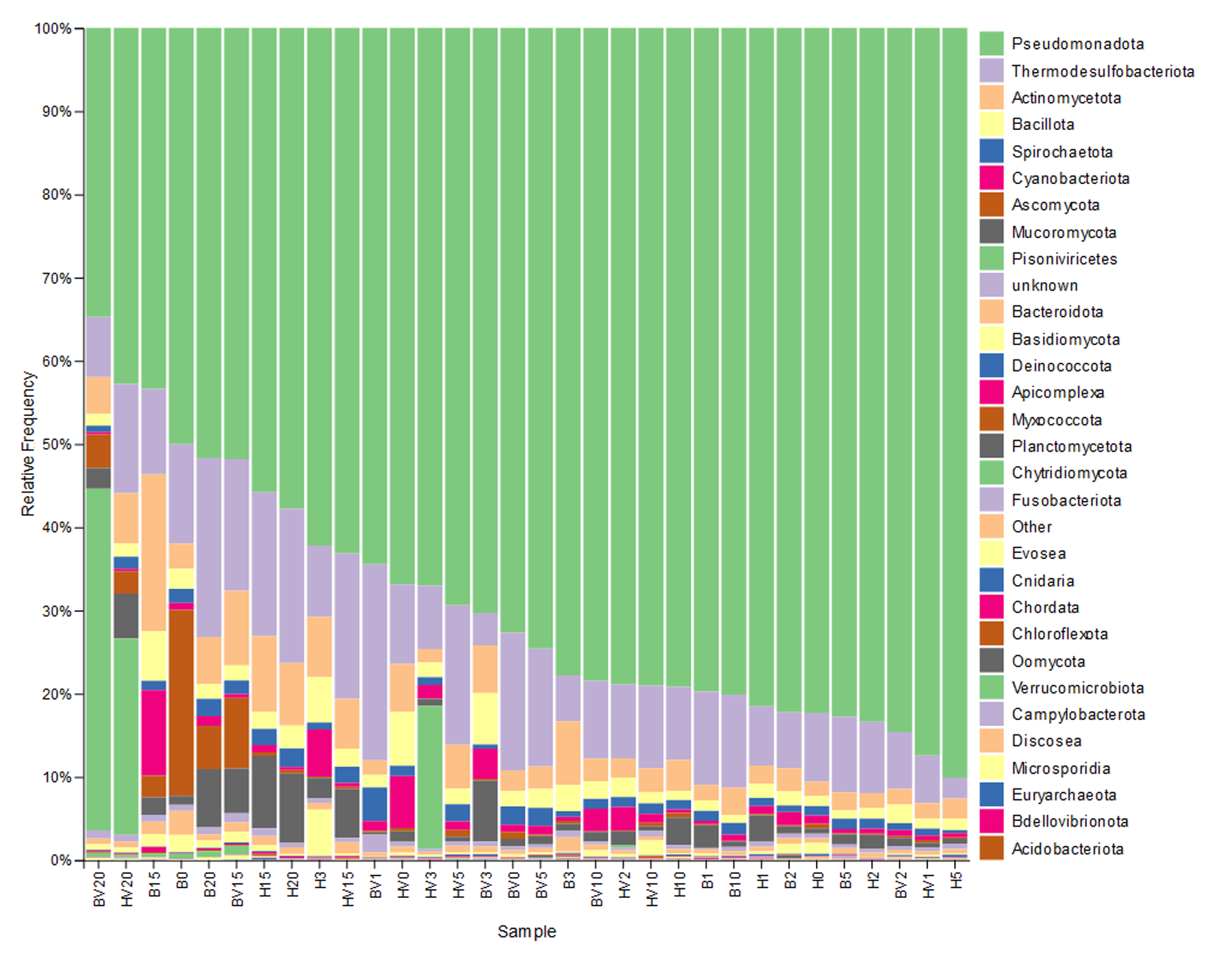

Supplement: Supplementary file 1 [file insects-16-00554-s001.zip › Figure S4-Phylum-byOrg.tif]
